# Supplementary material for: Assessing arthropod biodiversity with DNA barcoding in Jinnah Garden, Lahore, Pakistan
Source: PeerJ. 2024 May 31;12:e17420. doi: 10.7717/peerj.17420 (PMC11146329; doi:10.7717/peerj.17420)
Supplement: Supplemental Information 5 [file peerj-12-17420-s005.pdf]

BOLD TaxonID Tree

Title	:	Tree Result - DS-GMPJA
Date	:	08-Nov-2023
Data Type	:	Nucleotide
Distance Model	:	Kimura 2 Parameter
Marker	:	COI-5P
Colourization	:	[blue]=Stop Codons [red]=Contamination or misidentification
Label	:	Sample ID
Label	:	Process ID
Label	:	Taxon
Label	:	Barcode Cluster (BIN)
Filter	:	length > 400bp only
Filter	:	exclude records flagged as misidentifications
Filter	:	exclude records with stop codons
Filter	:	exclude contaminants
Sequence Count	:	8987
Species count	:	191
Genus count	:	326
Family count	:	170
Unidentified	:	7500
BIN Count	:	1361

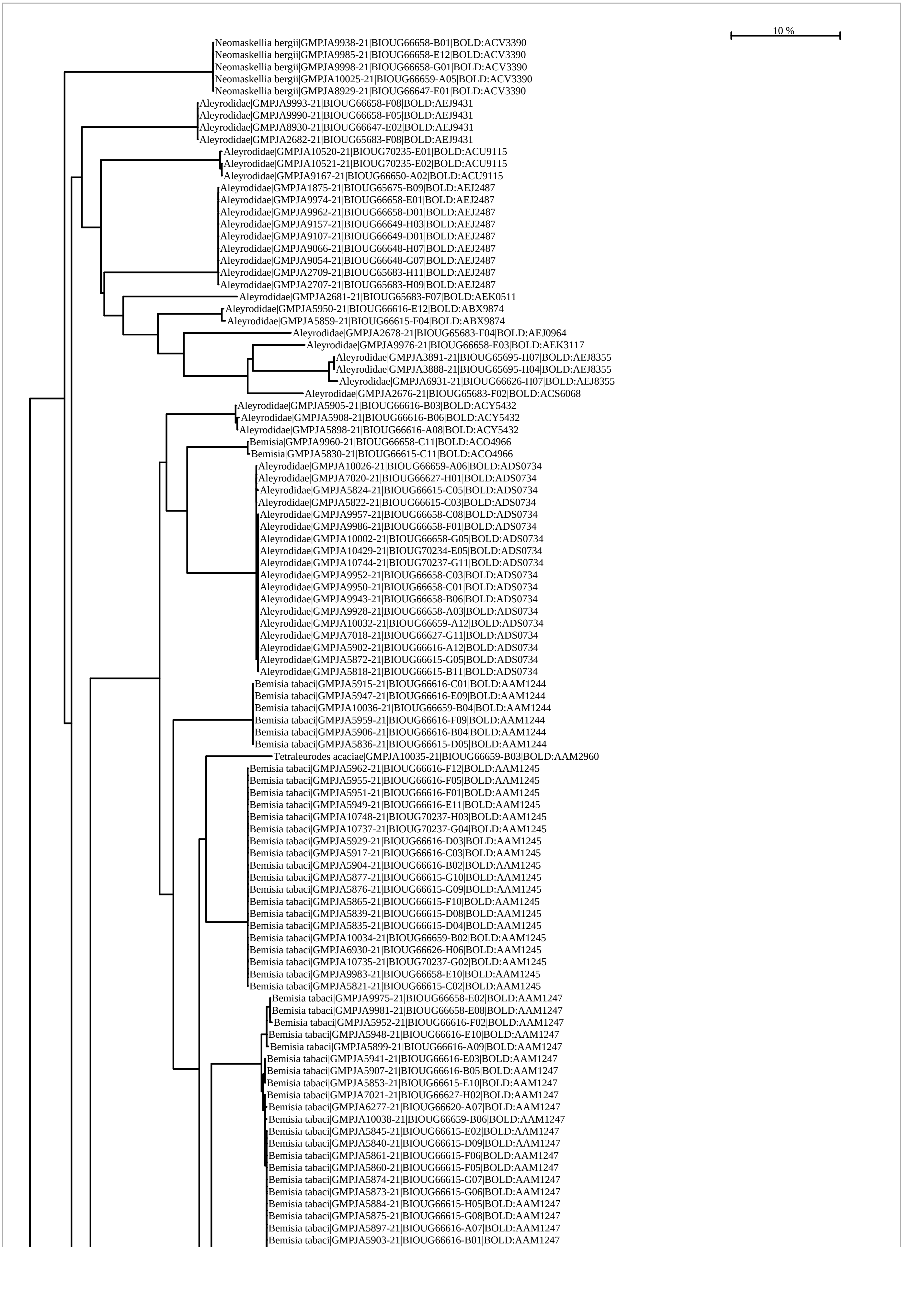
10 %
Neomaskellia bergii|GMPJA9938-21|BIOUG66658-B01|BOLD:ACV3390
Neomaskellia bergii|GMPJA9985-21|BIOUG66658-E12|BOLD:ACV3390
Neomaskellia bergii|GMPJA9998-21|BIOUG66658-G01|BOLD:ACV3390
Neomaskellia bergii|GMPJA10025-21|BIOUG66659-A05|BOLD:ACV3390
Neomaskellia bergii|GMPJA8929-21|BIOUG66647-E01|BOLD:ACV3390
Aleyrodidae|GMPJA9993-21|BIOUG66658-F08|BOLD:AEJ9431
Aleyrodidae|GMPJA9990-21|BIOUG66658-F05|BOLD:AEJ9431
Aleyrodidae|GMPJA8930-21|BIOUG66647-E02|BOLD:AEJ9431
Aleyrodidae|GMPJA2682-21|BIOUG65683-F08|BOLD:AEJ9431
Aleyrodidae|GMPJA10520-21|BIOUG70235-E01|BOLD:ACU9115
Aleyrodidae|GMPJA10521-21|BIOUG70235-E02|BOLD:ACU9115
Aleyrodidae|GMPJA9167-21|BIOUG66650-A02|BOLD:ACU9115
Aleyrodidae|GMPJA1875-21|BIOUG65675-B09|BOLD:AEJ2487
Aleyrodidae|GMPJA9974-21|BIOUG66658-E01|BOLD:AEJ2487
Aleyrodidae|GMPJA9962-21|BIOUG66658-D01|BOLD:AEJ2487
Aleyrodidae|GMPJA9157-21|BIOUG66649-H03|BOLD:AEJ2487
Aleyrodidae|GMPJA9107-21|BIOUG66649-D01|BOLD:AEJ2487
Aleyrodidae|GMPJA9066-21|BIOUG66648-H07|BOLD:AEJ2487
Aleyrodidae|GMPJA9054-21|BIOUG66648-G07|BOLD:AEJ2487
Aleyrodidae|GMPJA2709-21|BIOUG65683-H11|BOLD:AEJ2487
Aleyrodidae|GMPJA2707-21|BIOUG65683-H09|BOLD:AEJ2487
Aleyrodidae|GMPJA2681-21|BIOUG65683-F07|BOLD:AEK0511
Aleyrodidae|GMPJA5950-21|BIOUG66616-E12|BOLD:ABX9874
Aleyrodidae|GMPJA5859-21|BIOUG66615-F04|BOLD:ABX9874
Aleyrodidae|GMPJA2678-21|BIOUG65683-F04|BOLD:AEJ0964
Aleyrodidae|GMPJA9976-21|BIOUG66658-E03|BOLD:AEK3117
Aleyrodidae|GMPJA3891-21|BIOUG65695-H07|BOLD:AEJ8355
Aleyrodidae|GMPJA3888-21|BIOUG65695-H04|BOLD:AEJ8355
Aleyrodidae|GMPJA6931-21|BIOUG66626-H07|BOLD:AEJ8355
Aleyrodidae|GMPJA2676-21|BIOUG65683-F02|BOLD:ACS6068
Aleyrodidae|GMPJA5905-21|BIOUG66616-B03|BOLD:ACY5432
Aleyrodidae|GMPJA5908-21|BIOUG66616-B06|BOLD:ACY5432
Aleyrodidae|GMPJA5898-21|BIOUG66616-A08|BOLD:ACY5432
Bemisia|GMPJA9960-21|BIOUG66658-C11|BOLD:ACO4966
Bemisia|GMPJA5830-21|BIOUG66615-C11|BOLD:ACO4966
Aleyrodidae|GMPJA10026-21|BIOUG66659-A06|BOLD:ADS0734
Aleyrodidae|GMPJA7020-21|BIOUG66627-H01|BOLD:ADS0734
Aleyrodidae|GMPJA5824-21|BIOUG66615-C05|BOLD:ADS0734
Aleyrodidae|GMPJA5822-21|BIOUG66615-C03|BOLD:ADS0734
Aleyrodidae|GMPJA9957-21|BIOUG66658-C08|BOLD:ADS0734
Aleyrodidae|GMPJA9986-21|BIOUG66658-F01|BOLD:ADS0734
Aleyrodidae|GMPJA10002-21|BIOUG66658-G05|BOLD:ADS0734
Aleyrodidae|GMPJA10429-21|BIOUG70234-E05|BOLD:ADS0734
Aleyrodidae|GMPJA10744-21|BIOUG70237-G11|BOLD:ADS0734
Aleyrodidae|GMPJA9952-21|BIOUG66658-C03|BOLD:ADS0734
Aleyrodidae|GMPJA9950-21|BIOUG66658-C01|BOLD:ADS0734
Aleyrodidae|GMPJA9943-21|BIOUG66658-B06|BOLD:ADS0734
Aleyrodidae|GMPJA9928-21|BIOUG66658-A03|BOLD:ADS0734
Aleyrodidae|GMPJA10032-21|BIOUG66659-A12|BOLD:ADS0734
Aleyrodidae|GMPJA7018-21|BIOUG66627-G11|BOLD:ADS0734
Aleyrodidae|GMPJA5902-21|BIOUG66616-A12|BOLD:ADS0734
Aleyrodidae|GMPJA5872-21|BIOUG66615-G05|BOLD:ADS0734
Aleyrodidae|GMPJA5818-21|BIOUG66615-B11|BOLD:ADS0734
Bemisia tabaci|GMPJA5915-21|BIOUG66616-C01|BOLD:AAM1244
Bemisia tabaci|GMPJA5947-21|BIOUG66616-E09|BOLD:AAM1244
Bemisia tabaci|GMPJA10036-21|BIOUG66659-B04|BOLD:AAM1244
Bemisia tabaci|GMPJA5959-21|BIOUG66616-F09|BOLD:AAM1244
Bemisia tabaci|GMPJA5906-21|BIOUG66616-B04|BOLD:AAM1244
Bemisia tabaci|GMPJA5836-21|BIOUG66615-D05|BOLD:AAM1244
Tetraleurodes acaciae|GMPJA10035-21|BIOUG66659-B03|BOLD:AAM2960
Bemisia tabaci|GMPJA5962-21|BIOUG66616-F12|BOLD:AAM1245
Bemisia tabaci|GMPJA5955-21|BIOUG66616-F05|BOLD:AAM1245
Bemisia tabaci|GMPJA5951-21|BIOUG66616-F01|BOLD:AAM1245
Bemisia tabaci|GMPJA5949-21|BIOUG66616-E11|BOLD:AAM1245
Bemisia tabaci|GMPJA10748-21|BIOUG70237-H03|BOLD:AAM1245
Bemisia tabaci|GMPJA10737-21|BIOUG70237-G04|BOLD:AAM1245
Bemisia tabaci|GMPJA5929-21|BIOUG66616-D03|BOLD:AAM1245
Bemisia tabaci|GMPJA5917-21|BIOUG66616-C03|BOLD:AAM1245
Bemisia tabaci|GMPJA5904-21|BIOUG66616-B02|BOLD:AAM1245
Bemisia tabaci|GMPJA5877-21|BIOUG66615-G10|BOLD:AAM1245
Bemisia tabaci|GMPJA5876-21|BIOUG66615-G09|BOLD:AAM1245
Bemisia tabaci|GMPJA5865-21|BIOUG66615-F10|BOLD:AAM1245
Bemisia tabaci|GMPJA5839-21|BIOUG66615-D08|BOLD:AAM1245
Bemisia tabaci|GMPJA5835-21|BIOUG66615-D04|BOLD:AAM1245
Bemisia tabaci|GMPJA10034-21|BIOUG66659-B02|BOLD:AAM1245
Bemisia tabaci|GMPJA6930-21|BIOUG66626-H06|BOLD:AAM1245
Bemisia tabaci|GMPJA10735-21|BIOUG70237-G02|BOLD:AAM1245
Bemisia tabaci|GMPJA9983-21|BIOUG66658-E10|BOLD:AAM1245
Bemisia tabaci|GMPJA5821-21|BIOUG66615-C02|BOLD:AAM1245
Bemisia tabaci|GMPJA9975-21|BIOUG66658-E02|BOLD:AAM1247
Bemisia tabaci|GMPJA9981-21|BIOUG66658-E08|BOLD:AAM1247
Bemisia tabaci|GMPJA5952-21|BIOUG66616-F02|BOLD:AAM1247
Bemisia tabaci|GMPJA5948-21|BIOUG66616-E10|BOLD:AAM1247
Bemisia tabaci|GMPJA5899-21|BIOUG66616-A09|BOLD:AAM1247
Bemisia tabaci|GMPJA5941-21|BIOUG66616-E03|BOLD:AAM1247
Bemisia tabaci|GMPJA5907-21|BIOUG66616-B05|BOLD:AAM1247
Bemisia tabaci|GMPJA5853-21|BIOUG66615-E10|BOLD:AAM1247
Bemisia tabaci|GMPJA7021-21|BIOUG66627-H02|BOLD:AAM1247
Bemisia tabaci|GMPJA6277-21|BIOUG66620-A07|BOLD:AAM1247
Bemisia tabaci|GMPJA10038-21|BIOUG66659-B06|BOLD:AAM1247
Bemisia tabaci|GMPJA5845-21|BIOUG66615-E02|BOLD:AAM1247
Bemisia tabaci|GMPJA5840-21|BIOUG66615-D09|BOLD:AAM1247
Bemisia tabaci|GMPJA5861-21|BIOUG66615-F06|BOLD:AAM1247
Bemisia tabaci|GMPJA5860-21|BIOUG66615-F05|BOLD:AAM1247
Bemisia tabaci|GMPJA5874-21|BIOUG66615-G07|BOLD:AAM1247
Bemisia tabaci|GMPJA5873-21|BIOUG66615-G06|BOLD:AAM1247
Bemisia tabaci|GMPJA5884-21|BIOUG66615-H05|BOLD:AAM1247
Bemisia tabaci|GMPJA5875-21|BIOUG66615-G08|BOLD:AAM1247
Bemisia tabaci|GMPJA5897-21|BIOUG66616-A07|BOLD:AAM1247
Bemisia tabaci|GMPJA5903-21|BIOUG66616-B01|BOLD:AAM1247

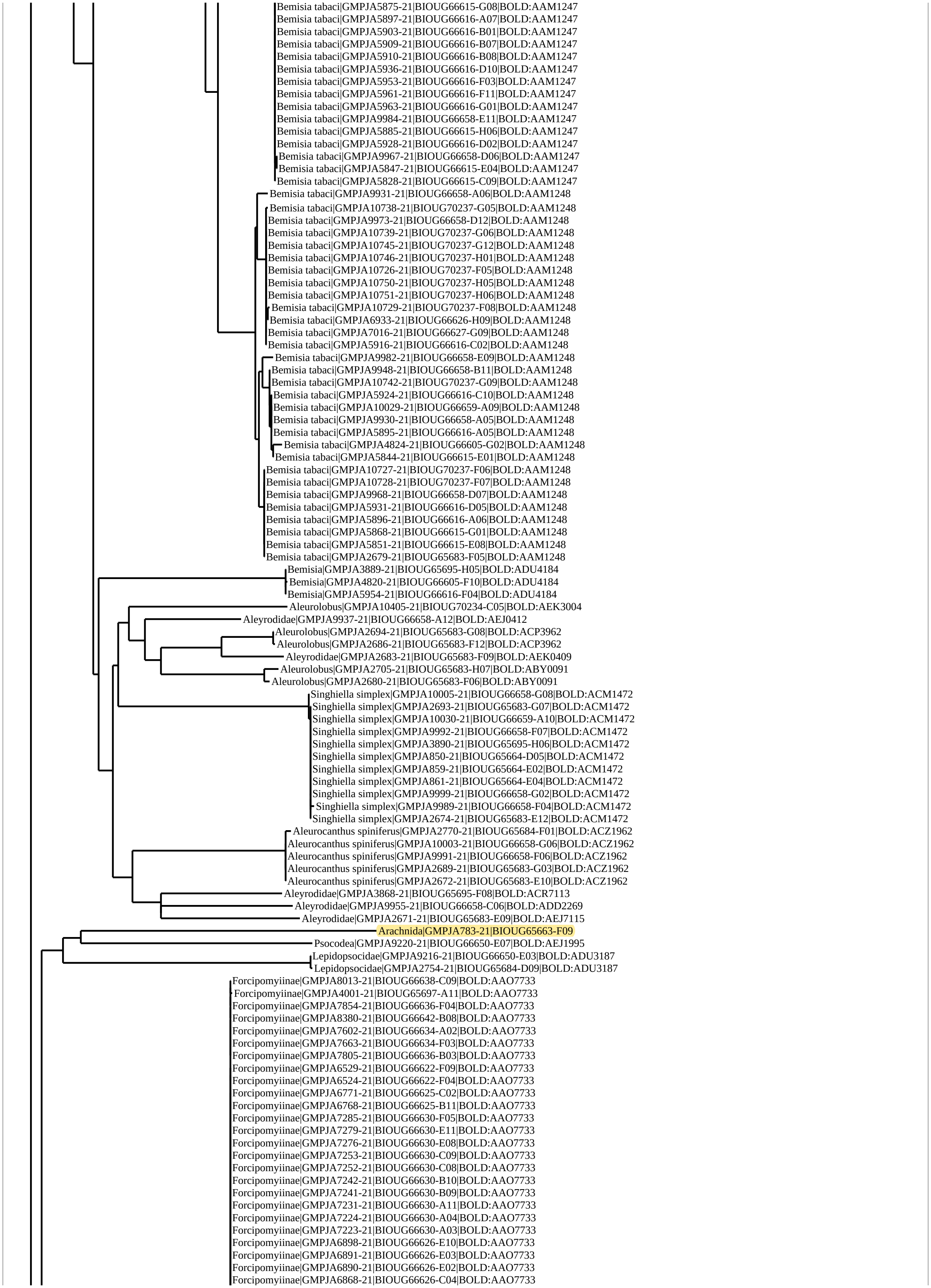

Bemisia tabaci|GMPJA5875-21|BIOUG66615-G08|BOLD:AAM1247
Bemisia tabaci|GMPJA5897-21|BIOUG66616-A07|BOLD:AAM1247
Bemisia tabaci|GMPJA5903-21|BIOUG66616-B01|BOLD:AAM1247
Bemisia tabaci|GMPJA5909-21|BIOUG66616-B07|BOLD:AAM1247
Bemisia tabaci|GMPJA5910-21|BIOUG66616-B08|BOLD:AAM1247
Bemisia tabaci|GMPJA5936-21|BIOUG66616-D10|BOLD:AAM1247
Bemisia tabaci|GMPJA5953-21|BIOUG66616-F03|BOLD:AAM1247
Bemisia tabaci|GMPJA5961-21|BIOUG66616-F11|BOLD:AAM1247
Bemisia tabaci|GMPJA5963-21|BIOUG66616-G01|BOLD:AAM1247
Bemisia tabaci|GMPJA9984-21|BIOUG66658-E11|BOLD:AAM1247
Bemisia tabaci|GMPJA5885-21|BIOUG66615-H06|BOLD:AAM1247
Bemisia tabaci|GMPJA5928-21|BIOUG66616-D02|BOLD:AAM1247
Bemisia tabaci|GMPJA9967-21|BIOUG66658-D06|BOLD:AAM1247
Bemisia tabaci|GMPJA5847-21|BIOUG66615-E04|BOLD:AAM1247
Bemisia tabaci|GMPJA5828-21|BIOUG66615-C09|BOLD:AAM1247
Bemisia tabaci|GMPJA9931-21|BIOUG66658-A06|BOLD:AAM1248
Bemisia tabaci|GMPJA10738-21|BIOUG70237-G05|BOLD:AAM1248
Bemisia tabaci|GMPJA9973-21|BIOUG66658-D12|BOLD:AAM1248
Bemisia tabaci|GMPJA10739-21|BIOUG70237-G06|BOLD:AAM1248
Bemisia tabaci|GMPJA10745-21|BIOUG70237-G12|BOLD:AAM1248
Bemisia tabaci|GMPJA10746-21|BIOUG70237-H01|BOLD:AAM1248
Bemisia tabaci|GMPJA10726-21|BIOUG70237-F05|BOLD:AAM1248
Bemisia tabaci|GMPJA10750-21|BIOUG70237-H05|BOLD:AAM1248
Bemisia tabaci|GMPJA10751-21|BIOUG70237-H06|BOLD:AAM1248
Bemisia tabaci|GMPJA10729-21|BIOUG70237-F08|BOLD:AAM1248
Bemisia tabaci|GMPJA6933-21|BIOUG66626-H09|BOLD:AAM1248
Bemisia tabaci|GMPJA7016-21|BIOUG66627-G09|BOLD:AAM1248
Bemisia tabaci|GMPJA5916-21|BIOUG66616-C02|BOLD:AAM1248
Bemisia tabaci|GMPJA9982-21|BIOUG66658-E09|BOLD:AAM1248
Bemisia tabaci|GMPJA9948-21|BIOUG66658-B11|BOLD:AAM1248
Bemisia tabaci|GMPJA10742-21|BIOUG70237-G09|BOLD:AAM1248
Bemisia tabaci|GMPJA5924-21|BIOUG66616-C10|BOLD:AAM1248
Bemisia tabaci|GMPJA10029-21|BIOUG66659-A09|BOLD:AAM1248
Bemisia tabaci|GMPJA9930-21|BIOUG66658-A05|BOLD:AAM1248
Bemisia tabaci|GMPJA5895-21|BIOUG66616-A05|BOLD:AAM1248
Bemisia tabaci|GMPJA4824-21|BIOUG66605-G02|BOLD:AAM1248
Bemisia tabaci|GMPJA5844-21|BIOUG66615-E01|BOLD:AAM1248
Bemisia tabaci|GMPJA10727-21|BIOUG70237-F06|BOLD:AAM1248
Bemisia tabaci|GMPJA10728-21|BIOUG70237-F07|BOLD:AAM1248
Bemisia tabaci|GMPJA9968-21|BIOUG66658-D07|BOLD:AAM1248
Bemisia tabaci|GMPJA5931-21|BIOUG66616-D05|BOLD:AAM1248
Bemisia tabaci|GMPJA5896-21|BIOUG66616-A06|BOLD:AAM1248
Bemisia tabaci|GMPJA5868-21|BIOUG66615-G01|BOLD:AAM1248
Bemisia tabaci|GMPJA5851-21|BIOUG66615-E08|BOLD:AAM1248
Bemisia tabaci|GMPJA2679-21|BIOUG65683-F05|BOLD:AAM1248
Bemisia|GMPJA3889-21|BIOUG65695-H05|BOLD:ADU4184
Bemisia|GMPJA4820-21|BIOUG66605-F10|BOLD:ADU4184
Bemisia|GMPJA5954-21|BIOUG66616-F04|BOLD:ADU4184
Aleurolobus|GMPJA10405-21|BIOUG70234-C05|BOLD:AEK3004
Aleyrodidae|GMPJA9937-21|BIOUG66658-A12|BOLD:AEJ0412
Aleurolobus|GMPJA2694-21|BIOUG65683-G08|BOLD:ACP3962
Aleurolobus|GMPJA2686-21|BIOUG65683-F12|BOLD:ACP3962
Aleyrodidae|GMPJA2683-21|BIOUG65683-F09|BOLD:AEK0409
Aleurolobus|GMPJA2705-21|BIOUG65683-H07|BOLD:ABY0091
Aleurolobus|GMPJA2680-21|BIOUG65683-F06|BOLD:ABY0091
Singhiella simplex|GMPJA10005-21|BIOUG66658-G08|BOLD:ACM1472
Singhiella simplex|GMPJA2693-21|BIOUG65683-G07|BOLD:ACM1472
Singhiella simplex|GMPJA10030-21|BIOUG66659-A10|BOLD:ACM1472
Singhiella simplex|GMPJA9992-21|BIOUG66658-F07|BOLD:ACM1472
Singhiella simplex|GMPJA3890-21|BIOUG65695-H06|BOLD:ACM1472
Singhiella simplex|GMPJA850-21|BIOUG65664-D05|BOLD:ACM1472
Singhiella simplex|GMPJA859-21|BIOUG65664-E02|BOLD:ACM1472
Singhiella simplex|GMPJA861-21|BIOUG65664-E04|BOLD:ACM1472
Singhiella simplex|GMPJA9999-21|BIOUG66658-G02|BOLD:ACM1472
Singhiella simplex|GMPJA9989-21|BIOUG66658-F04|BOLD:ACM1472
Singhiella simplex|GMPJA2674-21|BIOUG65683-E12|BOLD:ACM1472
Aleurocanthus spiniferus|GMPJA2770-21|BIOUG65684-F01|BOLD:ACZ1962
Aleurocanthus spiniferus|GMPJA10003-21|BIOUG66658-G06|BOLD:ACZ1962
Aleurocanthus spiniferus|GMPJA9991-21|BIOUG66658-F06|BOLD:ACZ1962
Aleurocanthus spiniferus|GMPJA2689-21|BIOUG65683-G03|BOLD:ACZ1962
Aleurocanthus spiniferus|GMPJA2672-21|BIOUG65683-E10|BOLD:ACZ1962
Aleyrodidae|GMPJA3868-21|BIOUG65695-F08|BOLD:ACR7113
Aleyrodidae|GMPJA9955-21|BIOUG66658-C06|BOLD:ADD2269
Aleyrodidae|GMPJA2671-21|BIOUG65683-E09|BOLD:AEJ7115
Arachnida|GMPJA783-21|BIOUG65663-F09
Psocodea|GMPJA9220-21|BIOUG66650-E07|BOLD:AEJ1995
Lepidopsocidae|GMPJA9216-21|BIOUG66650-E03|BOLD:ADU3187
Lepidopsocidae|GMPJA2754-21|BIOUG65684-D09|BOLD:ADU3187
Forcipomyiinae|GMPJA8013-21|BIOUG66638-C09|BOLD:AAO7733
Forcipomyiinae|GMPJA4001-21|BIOUG65697-A11|BOLD:AAO7733
Forcipomyiinae|GMPJA7854-21|BIOUG66636-F04|BOLD:AAO7733
Forcipomyiinae|GMPJA8380-21|BIOUG66642-B08|BOLD:AAO7733
Forcipomyiinae|GMPJA7602-21|BIOUG66634-A02|BOLD:AAO7733
Forcipomyiinae|GMPJA7663-21|BIOUG66634-F03|BOLD:AAO7733
Forcipomyiinae|GMPJA7805-21|BIOUG66636-B03|BOLD:AAO7733
Forcipomyiinae|GMPJA6529-21|BIOUG66622-F09|BOLD:AAO7733
Forcipomyiinae|GMPJA6524-21|BIOUG66622-F04|BOLD:AAO7733
Forcipomyiinae|GMPJA6771-21|BIOUG66625-C02|BOLD:AAO7733
Forcipomyiinae|GMPJA6768-21|BIOUG66625-B11|BOLD:AAO7733
Forcipomyiinae|GMPJA7285-21|BIOUG66630-F05|BOLD:AAO7733
Forcipomyiinae|GMPJA7279-21|BIOUG66630-E11|BOLD:AAO7733
Forcipomyiinae|GMPJA7276-21|BIOUG66630-E08|BOLD:AAO7733
Forcipomyiinae|GMPJA7253-21|BIOUG66630-C09|BOLD:AAO7733
Forcipomyiinae|GMPJA7252-21|BIOUG66630-C08|BOLD:AAO7733
Forcipomyiinae|GMPJA7242-21|BIOUG66630-B10|BOLD:AAO7733
Forcipomyiinae|GMPJA7241-21|BIOUG66630-B09|BOLD:AAO7733
Forcipomyiinae|GMPJA7231-21|BIOUG66630-A11|BOLD:AAO7733
Forcipomyiinae|GMPJA7224-21|BIOUG66630-A04|BOLD:AAO7733
Forcipomyiinae|GMPJA7223-21|BIOUG66630-A03|BOLD:AAO7733
Forcipomyiinae|GMPJA6898-21|BIOUG66626-E10|BOLD:AAO7733
Forcipomyiinae|GMPJA6891-21|BIOUG66626-E03|BOLD:AAO7733
Forcipomyiinae|GMPJA6890-21|BIOUG66626-E02|BOLD:AAO7733
Forcipomyiinae|GMPJA6868-21|BIOUG66626-C04|BOLD:AAO7733

Forcipomyiinae|GMPJA6891-21|BIOUG66626-E03|BOLD:AAO7733
Forcipomyiinae|GMPJA6890-21|BIOUG66626-E02|BOLD:AAO7733
Forcipomyiinae|GMPJA6868-21|BIOUG66626-C04|BOLD:AAO7733
Forcipomyiinae|GMPJA6861-21|BIOUG66626-B09|BOLD:AAO7733
Forcipomyiinae|GMPJA6860-21|BIOUG66626-B08|BOLD:AAO7733
Forcipomyiinae|GMPJA8176-21|BIOUG66640-A06|BOLD:AAO7733
Forcipomyiinae|GMPJA8175-21|BIOUG66640-A05|BOLD:AAO7733
Forcipomyiinae|GMPJA8612-21|BIOUG66644-F02|BOLD:AAO7733
Forcipomyiinae|GMPJA8608-21|BIOUG66644-E10|BOLD:AAO7733
Forcipomyiinae|GMPJA6847-21|BIOUG66626-A07|BOLD:AAO7733
Forcipomyiinae|GMPJA6843-21|BIOUG66626-A03|BOLD:AAO7733
Forcipomyiinae|GMPJA7452-21|BIOUG66632-D06|BOLD:AAO7733
Forcipomyiinae|GMPJA7442-21|BIOUG66632-C08|BOLD:AAO7733
Forcipomyiinae|GMPJA8302-21|BIOUG66641-D01|BOLD:AAO7733
Forcipomyiinae|GMPJA8298-21|BIOUG66641-C09|BOLD:AAO7733
Forcipomyiinae|GMPJA8291-21|BIOUG66641-C02|BOLD:AAO7733
Forcipomyiinae|GMPJA8273-21|BIOUG66641-A08|BOLD:AAO7733
Forcipomyiinae|GMPJA8033-21|BIOUG66638-E05|BOLD:AAO7733
Forcipomyiinae|GMPJA8031-21|BIOUG66638-E03|BOLD:AAO7733
Forcipomyiinae|GMPJA8027-21|BIOUG66638-D11|BOLD:AAO7733
Forcipomyiinae|GMPJA8019-21|BIOUG66638-D03|BOLD:AAO7733
Forcipomyiinae|GMPJA8193-21|BIOUG66640-B11|BOLD:AAO7733
Forcipomyiinae|GMPJA8189-21|BIOUG66640-B07|BOLD:AAO7733
Forcipomyiinae|GMPJA8184-21|BIOUG66640-B02|BOLD:AAO7733
Forcipomyiinae|GMPJA8177-21|BIOUG66640-A07|BOLD:AAO7733
Forcipomyiinae|GMPJA7521-21|BIOUG66633-B04|BOLD:AAO7733
Forcipomyiinae|GMPJA7512-21|BIOUG66633-A07|BOLD:AAO7733
Forcipomyiinae|GMPJA7291-21|BIOUG66630-F11|BOLD:AAO7733
Forcipomyiinae|GMPJA7290-21|BIOUG66630-F10|BOLD:AAO7733
Forcipomyiinae|GMPJA7733-21|BIOUG66635-D02|BOLD:AAO7733
Forcipomyiinae|GMPJA7732-21|BIOUG66635-D01|BOLD:AAO7733
Forcipomyiinae|GMPJA7714-21|BIOUG66635-B07|BOLD:AAO7733
Forcipomyiinae|GMPJA7713-21|BIOUG66635-B06|BOLD:AAO7733
Forcipomyiinae|GMPJA7709-21|BIOUG66635-B02|BOLD:AAO7733
Forcipomyiinae|GMPJA7703-21|BIOUG66635-A08|BOLD:AAO7733
Forcipomyiinae|GMPJA7699-21|BIOUG66635-A04|BOLD:AAO7733
Forcipomyiinae|GMPJA6903-21|BIOUG66626-F03|BOLD:AAO7733
Forcipomyiinae|GMPJA8008-21|BIOUG66638-C04|BOLD:AAO7733
Forcipomyiinae|GMPJA7966-21|BIOUG66637-G09|BOLD:AAO7733
Forcipomyiinae|GMPJA7525-21|BIOUG66633-B08|BOLD:AAO7733
Forcipomyiinae|GMPJA7522-21|BIOUG66633-B05|BOLD:AAO7733
Forcipomyiinae|GMPJA7510-21|BIOUG66633-A05|BOLD:AAO7733
Forcipomyiinae|GMPJA7504-21|BIOUG66632-H10|BOLD:AAO7733
Forcipomyiinae|GMPJA7502-21|BIOUG66632-H08|BOLD:AAO7733
Forcipomyiinae|GMPJA7501-21|BIOUG66632-H07|BOLD:AAO7733
Forcipomyiinae|GMPJA8423-21|BIOUG66642-F03|BOLD:AAO7733
Forcipomyiinae|GMPJA8422-21|BIOUG66642-F02|BOLD:AAO7733
Forcipomyiinae|GMPJA8258-21|BIOUG66640-H04|BOLD:AAO7733
Forcipomyiinae|GMPJA8239-21|BIOUG66640-F09|BOLD:AAO7733
Forcipomyiinae|GMPJA7494-21|BIOUG66632-G12|BOLD:AAO7733
Forcipomyiinae|GMPJA7491-21|BIOUG66632-G09|BOLD:AAO7733
Forcipomyiinae|GMPJA7490-21|BIOUG66632-G08|BOLD:AAO7733
Forcipomyiinae|GMPJA7489-21|BIOUG66632-G07|BOLD:AAO7733
Forcipomyiinae|GMPJA8238-21|BIOUG66640-F08|BOLD:AAO7733
Forcipomyiinae|GMPJA8231-21|BIOUG66640-F01|BOLD:AAO7733
Forcipomyiinae|GMPJA8203-21|BIOUG66640-C09|BOLD:AAO7733
Forcipomyiinae|GMPJA8196-21|BIOUG66640-C02|BOLD:AAO7733
Forcipomyiinae|GMPJA7303-21|BIOUG66630-G11|BOLD:AAO7733
Forcipomyiinae|GMPJA7297-21|BIOUG66630-G05|BOLD:AAO7733
Forcipomyiinae|GMPJA7296-21|BIOUG66630-G04|BOLD:AAO7733
Forcipomyiinae|GMPJA7294-21|BIOUG66630-G02|BOLD:AAO7733
Forcipomyiinae|GMPJA7599-21|BIOUG66633-H10|BOLD:AAO7733
Forcipomyiinae|GMPJA7596-21|BIOUG66633-H07|BOLD:AAO7733
Forcipomyiinae|GMPJA7590-21|BIOUG66633-H01|BOLD:AAO7733
Forcipomyiinae|GMPJA7583-21|BIOUG66633-G06|BOLD:AAO7733
Forcipomyiinae|GMPJA7575-21|BIOUG66633-F10|BOLD:AAO7733
Forcipomyiinae|GMPJA7551-21|BIOUG66633-D10|BOLD:AAO7733
Forcipomyiinae|GMPJA7543-21|BIOUG66633-D02|BOLD:AAO7733
Forcipomyiinae|GMPJA8085-21|BIOUG66639-A10|BOLD:AAO7733
Forcipomyiinae|GMPJA1931-21|BIOUG65675-G05|BOLD:AAO7733
Forcipomyiinae|GMPJA1904-21|BIOUG65675-E02|BOLD:AAO7733
Forcipomyiinae|GMPJA7656-21|BIOUG66634-E08|BOLD:AAO7733
Forcipomyiinae|GMPJA7655-21|BIOUG66634-E07|BOLD:AAO7733
Forcipomyiinae|GMPJA10297-21|BIOUG70233-B04|BOLD:AAO7733
Forcipomyiinae|GMPJA10260-21|BIOUG70232-G02|BOLD:AAO7733
Forcipomyiinae|GMPJA8653-21|BIOUG66645-A08|BOLD:AAO7733
Forcipomyiinae|GMPJA8642-21|BIOUG66644-H08|BOLD:AAO7733
Forcipomyiinae|GMPJA8084-21|BIOUG66639-A09|BOLD:AAO7733
Forcipomyiinae|GMPJA8073-21|BIOUG66638-H09|BOLD:AAO7733
Forcipomyiinae|GMPJA8067-21|BIOUG66638-H03|BOLD:AAO7733
Forcipomyiinae|GMPJA8066-21|BIOUG66638-H02|BOLD:AAO7733
Forcipomyiinae|GMPJA7487-21|BIOUG66632-G05|BOLD:AAO7733
Forcipomyiinae|GMPJA7461-21|BIOUG66632-E03|BOLD:AAO7733
Forcipomyiinae|GMPJA7460-21|BIOUG66632-E02|BOLD:AAO7733
Forcipomyiinae|GMPJA7347-21|BIOUG66631-C08|BOLD:AAO7733
Forcipomyiinae|GMPJA7329-21|BIOUG66631-B02|BOLD:AAO7733
Forcipomyiinae|GMPJA7326-21|BIOUG66631-A11|BOLD:AAO7733
Forcipomyiinae|GMPJA7317-21|BIOUG66631-A02|BOLD:AAO7733
Forcipomyiinae|GMPJA7305-21|BIOUG66630-H01|BOLD:AAO7733
Forcipomyiinae|GMPJA8173-21|BIOUG66640-A03|BOLD:AAO7733
Forcipomyiinae|GMPJA8167-21|BIOUG66639-H08|BOLD:AAO7733
Forcipomyiinae|GMPJA8164-21|BIOUG66639-H05|BOLD:AAO7733
Forcipomyiinae|GMPJA8159-21|BIOUG66639-G12|BOLD:AAO7733
Forcipomyiinae|GMPJA7402-21|BIOUG66631-H03|BOLD:AAO7733
Forcipomyiinae|GMPJA7398-21|BIOUG66631-G11|BOLD:AAO7733
Forcipomyiinae|GMPJA7360-21|BIOUG66631-D09|BOLD:AAO7733
Forcipomyiinae|GMPJA7359-21|BIOUG66631-D08|BOLD:AAO7733
Forcipomyiinae|GMPJA7389-21|BIOUG66631-G02|BOLD:AAO7733
Forcipomyiinae|GMPJA7379-21|BIOUG66631-F04|BOLD:AAO7733
Forcipomyiinae|GMPJA7357-21|BIOUG66631-D06|BOLD:AAO7733
Forcipomyiinae|GMPJA6815-21|BIOUG66625-F10|BOLD:AAO7733
Forcipomyiinae|GMPJA6808-21|BIOUG66625-F03|BOLD:AAO7733
Forcipomyiinae|GMPJA6783-21|BIOUG66625-D02|BOLD:AAO7733

Forcipomyiinae|GMPJA6815-21|BIOUG66625-F10|BOLD:AAO7733
Forcipomyiinae|GMPJA6808-21|BIOUG66625-F03|BOLD:AAO7733
Forcipomyiinae|GMPJA6783-21|BIOUG66625-D02|BOLD:AAO7733
Forcipomyiinae|GMPJA6779-21|BIOUG66625-C10|BOLD:AAO7733
Forcipomyiinae|GMPJA6773-21|BIOUG66625-C04|BOLD:AAO7733
Forcipomyiinae|GMPJA6761-21|BIOUG66625-B04|BOLD:AAO7733
Forcipomyiinae|GMPJA6712-21|BIOUG66624-F02|BOLD:AAO7733
Forcipomyiinae|GMPJA6670-21|BIOUG66624-B08|BOLD:AAO7733
Forcipomyiinae|GMPJA6666-21|BIOUG66624-B04|BOLD:AAO7733
Forcipomyiinae|GMPJA6653-21|BIOUG66624-A03|BOLD:AAO7733
Forcipomyiinae|GMPJA6621-21|BIOUG66623-F06|BOLD:AAO7733
Forcipomyiinae|GMPJA6620-21|BIOUG66623-F05|BOLD:AAO7733
Forcipomyiinae|GMPJA6607-21|BIOUG66623-E04|BOLD:AAO7733
Forcipomyiinae|GMPJA6596-21|BIOUG66623-D05|BOLD:AAO7733
Forcipomyiinae|GMPJA6589-21|BIOUG66623-C10|BOLD:AAO7733
Forcipomyiinae|GMPJA6587-21|BIOUG66623-C08|BOLD:AAO7733
Forcipomyiinae|GMPJA6584-21|BIOUG66623-C05|BOLD:AAO7733
Forcipomyiinae|GMPJA6580-21|BIOUG66623-C01|BOLD:AAO7733
Forcipomyiinae|GMPJA6565-21|BIOUG66623-A10|BOLD:AAO7733
Forcipomyiinae|GMPJA6541-21|BIOUG66622-G09|BOLD:AAO7733
Forcipomyiinae|GMPJA6537-21|BIOUG66622-G05|BOLD:AAO7733
Forcipomyiinae|GMPJA6536-21|BIOUG66622-G04|BOLD:AAO7733
Forcipomyiinae|GMPJA6531-21|BIOUG66622-F11|BOLD:AAO7733
Forcipomyiinae|GMPJA6516-21|BIOUG66622-E08|BOLD:AAO7733
Forcipomyiinae|GMPJA6511-21|BIOUG66622-E03|BOLD:AAO7733
Forcipomyiinae|GMPJA6501-21|BIOUG66622-D05|BOLD:AAO7733
Forcipomyiinae|GMPJA6499-21|BIOUG66622-D03|BOLD:AAO7733
Forcipomyiinae|GMPJA6492-21|BIOUG66622-C08|BOLD:AAO7733
Forcipomyiinae|GMPJA6406-21|BIOUG66621-D05|BOLD:AAO7733
Forcipomyiinae|GMPJA6392-21|BIOUG66621-C03|BOLD:AAO7733
Forcipomyiinae|GMPJA6387-21|BIOUG66621-B10|BOLD:AAO7733
Forcipomyiinae|GMPJA6379-21|BIOUG66621-B02|BOLD:AAO7733
Forcipomyiinae|GMPJA6376-21|BIOUG66621-A11|BOLD:AAO7733
Forcipomyiinae|GMPJA6374-21|BIOUG66621-A09|BOLD:AAO7733
Forcipomyiinae|GMPJA6739-21|BIOUG66624-H05|BOLD:AAO7733
Forcipomyiinae|GMPJA6738-21|BIOUG66624-H04|BOLD:AAO7733
Forcipomyiinae|GMPJA6736-21|BIOUG66624-H02|BOLD:AAO7733
Forcipomyiinae|GMPJA6732-21|BIOUG66624-G10|BOLD:AAO7733
Forcipomyiinae|GMPJA6720-21|BIOUG66624-F10|BOLD:AAO7733
Forcipomyiinae|GMPJA6254-21|BIOUG66619-G07|BOLD:AAO7733
Forcipomyiinae|GMPJA6193-21|BIOUG66619-B06|BOLD:AAO7733
Forcipomyiinae|GMPJA6190-21|BIOUG66619-B03|BOLD:AAO7733
Forcipomyiinae|GMPJA6481-21|BIOUG66622-B09|BOLD:AAO7733
Forcipomyiinae|GMPJA6480-21|BIOUG66622-B08|BOLD:AAO7733
Forcipomyiinae|GMPJA6477-21|BIOUG66622-B05|BOLD:AAO7733
Forcipomyiinae|GMPJA6475-21|BIOUG66622-B03|BOLD:AAO7733
Forcipomyiinae|GMPJA6459-21|BIOUG66621-H10|BOLD:AAO7733
Forcipomyiinae|GMPJA6456-21|BIOUG66621-H07|BOLD:AAO7733
Forcipomyiinae|GMPJA6448-21|BIOUG66621-G11|BOLD:AAO7733
Forcipomyiinae|GMPJA6446-21|BIOUG66621-G09|BOLD:AAO7733
Forcipomyiinae|GMPJA6439-21|BIOUG66621-G02|BOLD:AAO7733
Forcipomyiinae|GMPJA8605-21|BIOUG66644-E07|BOLD:AAO7733
Forcipomyiinae|GMPJA8604-21|BIOUG66644-E06|BOLD:AAO7733
Forcipomyiinae|GMPJA8600-21|BIOUG66644-E02|BOLD:AAO7733
Forcipomyiinae|GMPJA8597-21|BIOUG66644-D11|BOLD:AAO7733
Forcipomyiinae|GMPJA7438-21|BIOUG66632-C04|BOLD:AAO7733
Forcipomyiinae|GMPJA7437-21|BIOUG66632-C03|BOLD:AAO7733
Forcipomyiinae|GMPJA7435-21|BIOUG66632-C01|BOLD:AAO7733
Forcipomyiinae|GMPJA7430-21|BIOUG66632-B08|BOLD:AAO7733
Forcipomyiinae|GMPJA8146-21|BIOUG66639-F11|BOLD:AAO7733
Forcipomyiinae|GMPJA8142-21|BIOUG66639-F07|BOLD:AAO7733
Forcipomyiinae|GMPJA8135-21|BIOUG66639-E12|BOLD:AAO7733
Forcipomyiinae|GMPJA8130-21|BIOUG66639-E07|BOLD:AAO7733
Forcipomyiinae|GMPJA8114-21|BIOUG66639-D03|BOLD:AAO7733
Forcipomyiinae|GMPJA8106-21|BIOUG66639-C07|BOLD:AAO7733
Forcipomyiinae|GMPJA8095-21|BIOUG66639-B08|BOLD:AAO7733
Forcipomyiinae|GMPJA7954-21|BIOUG66637-F09|BOLD:AAO7733
Forcipomyiinae|GMPJA7953-21|BIOUG66637-F08|BOLD:AAO7733
Forcipomyiinae|GMPJA7952-21|BIOUG66637-F07|BOLD:AAO7733
Forcipomyiinae|GMPJA7947-21|BIOUG66637-F02|BOLD:AAO7733
Forcipomyiinae|GMPJA7946-21|BIOUG66637-F01|BOLD:AAO7733
Forcipomyiinae|GMPJA7937-21|BIOUG66637-E04|BOLD:AAO7733
Forcipomyiinae|GMPJA7935-21|BIOUG66637-E02|BOLD:AAO7733
Forcipomyiinae|GMPJA7930-21|BIOUG66637-D09|BOLD:AAO7733
Forcipomyiinae|GMPJA7928-21|BIOUG66637-D07|BOLD:AAO7733
Forcipomyiinae|GMPJA7924-21|BIOUG66637-D03|BOLD:AAO7733
Forcipomyiinae|GMPJA7918-21|BIOUG66637-C09|BOLD:AAO7733
Forcipomyiinae|GMPJA7908-21|BIOUG66637-B11|BOLD:AAO7733
Forcipomyiinae|GMPJA7905-21|BIOUG66637-B08|BOLD:AAO7733
Forcipomyiinae|GMPJA7898-21|BIOUG66637-B01|BOLD:AAO7733
Forcipomyiinae|GMPJA7895-21|BIOUG66637-A10|BOLD:AAO7733
Forcipomyiinae|GMPJA7894-21|BIOUG66637-A09|BOLD:AAO7733
Forcipomyiinae|GMPJA7876-21|BIOUG66636-H02|BOLD:AAO7733
Forcipomyiinae|GMPJA7866-21|BIOUG66636-G04|BOLD:AAO7733
Forcipomyiinae|GMPJA7845-21|BIOUG66636-E07|BOLD:AAO7733
Forcipomyiinae|GMPJA7844-21|BIOUG66636-E06|BOLD:AAO7733
Forcipomyiinae|GMPJA7837-21|BIOUG66636-D11|BOLD:AAO7733
Forcipomyiinae|GMPJA8314-21|BIOUG66641-E01|BOLD:AAO7733
Forcipomyiinae|GMPJA7815-21|BIOUG66636-C01|BOLD:AAO7733
Forcipomyiinae|GMPJA7813-21|BIOUG66636-B11|BOLD:AAO7733
Forcipomyiinae|GMPJA7812-21|BIOUG66636-B10|BOLD:AAO7733
Forcipomyiinae|GMPJA7797-21|BIOUG66636-A07|BOLD:AAO7733
Forcipomyiinae|GMPJA7795-21|BIOUG66636-A05|BOLD:AAO7733
Forcipomyiinae|GMPJA7794-21|BIOUG66636-A04|BOLD:AAO7733
Forcipomyiinae|GMPJA7787-21|BIOUG66635-H08|BOLD:AAO7733
Forcipomyiinae|GMPJA7783-21|BIOUG66635-H04|BOLD:AAO7733
Forcipomyiinae|GMPJA7782-21|BIOUG66635-H03|BOLD:AAO7733
Forcipomyiinae|GMPJA7775-21|BIOUG66635-G08|BOLD:AAO7733
Forcipomyiinae|GMPJA7768-21|BIOUG66635-G01|BOLD:AAO7733
Forcipomyiinae|GMPJA7761-21|BIOUG66635-F06|BOLD:AAO7733
Forcipomyiinae|GMPJA7760-21|BIOUG66635-F05|BOLD:AAO7733
Forcipomyiinae|GMPJA7741-21|BIOUG66635-D10|BOLD:AAO7733
Forcipomyiinae|GMPJA7739-21|BIOUG66635-D08|BOLD:AAO7733

[illegible]
Forcipomyiinae|GMPJA7760-21|BIOUG66635-F05|BOLD:AAO7733
Forcipomyiinae|GMPJA7741-21|BIOUG66635-D10|BOLD:AAO7733
Forcipomyiinae|GMPJA7739-21|BIOUG66635-D08|BOLD:AAO7733
Forcipomyiinae|GMPJA8214-21|BIOUG66640-D08|BOLD:AAO7733
Forcipomyiinae|GMPJA8205-21|BIOUG66640-C11|BOLD:AAO7733
Forcipomyiinae|GMPJA7832-21|BIOUG66636-D06|BOLD:AAO7733
Forcipomyiinae|GMPJA8321-21|BIOUG66641-E08|BOLD:AAO7733
Forcipomyiinae|GMPJA7652-21|BIOUG66634-E04|BOLD:AAO7733
Forcipomyiinae|GMPJA7651-21|BIOUG66634-E03|BOLD:AAO7733
Forcipomyiinae|GMPJA7649-21|BIOUG66634-E01|BOLD:AAO7733
Forcipomyiinae|GMPJA7638-21|BIOUG66634-D02|BOLD:AAO7733
Forcipomyiinae|GMPJA8589-21|BIOUG66644-D03|BOLD:AAO7733
Forcipomyiinae|GMPJA8588-21|BIOUG66644-D02|BOLD:AAO7733
Forcipomyiinae|GMPJA8584-21|BIOUG66644-C10|BOLD:AAO7733
Forcipomyiinae|GMPJA8583-21|BIOUG66644-C09|BOLD:AAO7733
Forcipomyiinae|GMPJA7427-21|BIOUG66632-B05|BOLD:AAO7733
Forcipomyiinae|GMPJA7421-21|BIOUG66632-A11|BOLD:AAO7733
Forcipomyiinae|GMPJA7420-21|BIOUG66632-A10|BOLD:AAO7733
Forcipomyiinae|GMPJA7418-21|BIOUG66632-A08|BOLD:AAO7733
Forcipomyiinae|GMPJA6428-21|BIOUG66621-F03|BOLD:AAO7733
Forcipomyiinae|GMPJA5200-21|BIOUG66608-F10|BOLD:AAO7733
Forcipomyiinae|GMPJA7676-21|BIOUG66634-G04|BOLD:AAO7733
Forcipomyiinae|GMPJA7665-21|BIOUG66634-F05|BOLD:AAO7733
Forcipomyiinae|GMPJA7661-21|BIOUG66634-F01|BOLD:AAO7733
Forcipomyiinae|GMPJA7657-21|BIOUG66634-E09|BOLD:AAO7733
Forcipomyiinae|GMPJA7625-21|BIOUG66634-C01|BOLD:AAO7733
Forcipomyiinae|GMPJA7623-21|BIOUG66634-B11|BOLD:AAO7733
Forcipomyiinae|GMPJA7621-21|BIOUG66634-B09|BOLD:AAO7733
Forcipomyiinae|GMPJA7620-21|BIOUG66634-B08|BOLD:AAO7733
Forcipomyiinae|GMPJA7617-21|BIOUG66634-B05|BOLD:AAO7733
Forcipomyiinae|GMPJA7615-21|BIOUG66634-B03|BOLD:AAO7733
Forcipomyiinae|GMPJA7612-21|BIOUG66634-A12|BOLD:AAO7733
Forcipomyiinae|GMPJA7611-21|BIOUG66634-A11|BOLD:AAO7733
Forcipomyiinae|GMPJA6381-21|BIOUG66621-B04|BOLD:AAO7733
Forcipomyiinae|GMPJA6380-21|BIOUG66621-B03|BOLD:AAO7733
Forcipomyiinae|GMPJA5171-21|BIOUG66608-D05|BOLD:AAO7733
Forcipomyiinae|GMPJA5162-21|BIOUG66608-C08|BOLD:AAO7733
Forcipomyiinae|GMPJA5104-21|BIOUG66607-F09|BOLD:AAO7733
Forcipomyiinae|GMPJA5103-21|BIOUG66607-F08|BOLD:AAO7733
Forcipomyiinae|GMPJA5323-21|BIOUG66610-A03|BOLD:AAO7733
Forcipomyiinae|GMPJA5050-21|BIOUG66607-B03|BOLD:AAO7733
Forcipomyiinae|GMPJA8451-21|BIOUG66642-H07|BOLD:AAO7733
Forcipomyiinae|GMPJA8443-21|BIOUG66642-G11|BOLD:AAO7733
Forcipomyiinae|GMPJA8439-21|BIOUG66642-G07|BOLD:AAO7733
Forcipomyiinae|GMPJA8424-21|BIOUG66642-F04|BOLD:AAO7733
Forcipomyiinae|GMPJA8530-21|BIOUG66643-G03|BOLD:AAO7733
Forcipomyiinae|GMPJA8529-21|BIOUG66643-G02|BOLD:AAO7733
Forcipomyiinae|GMPJA8456-21|BIOUG66643-A01|BOLD:AAO7733
Forcipomyiinae|GMPJA8453-21|BIOUG66642-H09|BOLD:AAO7733
Forcipomyiinae|GMPJA8546-21|BIOUG66643-H07|BOLD:AAO7733
Forcipomyiinae|GMPJA8541-21|BIOUG66643-H02|BOLD:AAO7733
Forcipomyiinae|GMPJA8540-21|BIOUG66643-H01|BOLD:AAO7733
Forcipomyiinae|GMPJA8537-21|BIOUG66643-G10|BOLD:AAO7733
Forcipomyiinae|GMPJA8072-21|BIOUG66638-H08|BOLD:AAO7733
Forcipomyiinae|GMPJA8071-21|BIOUG66638-H07|BOLD:AAO7733
Forcipomyiinae|GMPJA8061-21|BIOUG66638-G09|BOLD:AAO7733
Forcipomyiinae|GMPJA8056-21|BIOUG66638-G04|BOLD:AAO7733
Forcipomyiinae|GMPJA8375-21|BIOUG66642-B03|BOLD:AAO7733
Forcipomyiinae|GMPJA8369-21|BIOUG66642-A09|BOLD:AAO7733
Forcipomyiinae|GMPJA8354-21|BIOUG66641-H05|BOLD:AAO7733
Forcipomyiinae|GMPJA8307-21|BIOUG66641-D06|BOLD:AAO7733
Forcipomyiinae|GMPJA8053-21|BIOUG66638-G01|BOLD:AAO7733
Forcipomyiinae|GMPJA8051-21|BIOUG66638-F11|BOLD:AAO7733
Forcipomyiinae|GMPJA8050-21|BIOUG66638-F10|BOLD:AAO7733
Forcipomyiinae|GMPJA8039-21|BIOUG66638-E11|BOLD:AAO7733
Forcipomyiinae|GMPJA3517-21|BIOUG65692-A02|BOLD:AAO7733
Forcipomyiinae|GMPJA4380-21|BIOUG66601-A10|BOLD:AAO7733
Forcipomyiinae|GMPJA3508-21|BIOUG65691-H04|BOLD:AAO7733
Forcipomyiinae|GMPJA10327-21|BIOUG70233-D10|BOLD:AAO7733
Forcipomyiinae|GMPJA8155-21|BIOUG66639-G08|BOLD:AAO7733
Forcipomyiinae|GMPJA8154-21|BIOUG66639-G07|BOLD:AAO7733
Forcipomyiinae|GMPJA8152-21|BIOUG66639-G05|BOLD:AAO7733
Forcipomyiinae|GMPJA8150-21|BIOUG66639-G03|BOLD:AAO7733
Forcipomyiinae|GMPJA8230-21|BIOUG66640-E12|BOLD:AAO7733
Forcipomyiinae|GMPJA8224-21|BIOUG66640-E06|BOLD:AAO7733
Forcipomyiinae|GMPJA8220-21|BIOUG66640-E02|BOLD:AAO7733
Forcipomyiinae|GMPJA8219-21|BIOUG66640-E01|BOLD:AAO7733
Forcipomyiinae|GMPJA1869-21|BIOUG65675-B03|BOLD:AAO7733
Forcipomyiinae|GMPJA1360-21|BIOUG65669-G04|BOLD:AAO7733
Forcipomyiinae|GMPJA1002-21|BIOUG65666-A03|BOLD:AAO7733
Forcipomyiinae|GMPJA4025-21|BIOUG65697-C11|BOLD:AAO7733
Forcipomyiinae|GMPJA7230-21|BIOUG66630-A10|BOLD:AAO7733
Forcipomyiinae|GMPJA7227-21|BIOUG66630-A07|BOLD:AAO7733
Forcipomyiinae|GMPJA4000-21|BIOUG65697-A10|BOLD:AAO7733
Forcipomyiinae|GMPJA3548-21|BIOUG65692-C09|BOLD:AAO7733
Forcipomyiinae|GMPJA8404-21|BIOUG66642-D08|BOLD:AAO7733
Forcipomyiinae|GMPJA8376-21|BIOUG66642-B04|BOLD:AAO7733
Forcipomyiinae|GMPJA2285-21|BIOUG65679-E03|BOLD:AAO7733
Forcipomyiinae|GMPJA1613-21|BIOUG65672-D08|BOLD:AAO7733
Forcipomyiinae|GMPJA8462-21|BIOUG66643-A07|BOLD:AAO7733
Forcipomyiinae|GMPJA8457-21|BIOUG66643-A02|BOLD:AAO7733
Forcipomyiinae|GMPJA2348-21|BIOUG65680-B07|BOLD:AAO7733
Forcipomyiinae|GMPJA2314-21|BIOUG65679-G08|BOLD:AAO7733
Forcipomyiinae|GMPJA8490-21|BIOUG66643-C11|BOLD:AAO7733
Forcipomyiinae|GMPJA8483-21|BIOUG66643-C04|BOLD:AAO7733
Forcipomyiinae|GMPJA7384-21|BIOUG66631-F09|BOLD:AAO7733
Forcipomyiinae|GMPJA6506-21|BIOUG66622-D10|BOLD:AAO7733
Forcipomyiinae|GMPJA7302-21|BIOUG66630-G10|BOLD:AAO7733
Forcipomyiinae|GMPJA7422-21|BIOUG66632-A12|BOLD:AAO7733
Forcipomyiinae|GMPJA1711-21|BIOUG65673-D11|BOLD:AAO7733
Forcipomyiinae|GMPJA1662-21|BIOUG65672-H09|BOLD:AAO7733
Forcipomyiinae|GMPJA8524-21|BIOUG66643-F09|BOLD:AAO7733
Forcipomyiinae|GMPJA8519-21|BIOUG66643-F04|BOLD:AAO7733

Forcipomyiinae|GMPJA1711-21|BIOUG65673-D11|[illegible]
Forcipomyiinae|GMPJA1662-21|BIOUG65672-H09|BOLD:AAO7733
Forcipomyiinae|GMPJA8524-21|BIOUG66643-F09|BOLD:AAO7733
Forcipomyiinae|GMPJA8519-21|BIOUG66643-F04|BOLD:AAO7733
Forcipomyiinae|GMPJA8701-21|BIOUG66645-E08|BOLD:AAO7733
Forcipomyiinae|GMPJA8696-21|BIOUG66645-E03|BOLD:AAO7733
Forcipomyiinae|GMPJA8737-21|BIOUG66645-H08|BOLD:AAO7733
Forcipomyiinae|GMPJA8734-21|BIOUG66645-H05|BOLD:AAO7733
Forcipomyiinae|GMPJA8755-21|BIOUG66646-B03|BOLD:AAO7733
Forcipomyiinae|GMPJA8751-21|BIOUG66646-A11|BOLD:AAO7733
Forcipomyiinae|GMPJA8818-21|BIOUG66646-G06|BOLD:AAO7733
Forcipomyiinae|GMPJA8808-21|BIOUG66646-F08|BOLD:AAO7733
Forcipomyiinae|GMPJA10286-21|BIOUG70233-A05|BOLD:AAO7733
Forcipomyiinae|GMPJA10282-21|BIOUG70233-A01|BOLD:AAO7733
Forcipomyiinae|GMPJA10188-21|BIOUG70232-A02|BOLD:AAO7733
Forcipomyiinae|GMPJA10649-21|BIOUG70236-G11|BOLD:AAO7733
Forcipomyiinae|GMPJA2250-21|BIOUG65679-B04|BOLD:AAO7733
Forcipomyiinae|GMPJA8101-21|BIOUG66639-C02|BOLD:AAO7733
Forcipomyiinae|GMPJA8512-21|BIOUG66643-E09|BOLD:AAO7733
Forcipomyiinae|GMPJA8508-21|BIOUG66643-E05|BOLD:AAO7733
Forcipomyiinae|GMPJA8667-21|BIOUG66645-B10|BOLD:AAO7733
Forcipomyiinae|GMPJA8666-21|BIOUG66645-B09|BOLD:AAO7733
Forcipomyiinae|GMPJA8720-21|BIOUG66645-G03|BOLD:AAO7733
Forcipomyiinae|GMPJA8703-21|BIOUG66645-E10|BOLD:AAO7733
Forcipomyiinae|GMPJA8750-21|BIOUG66646-A10|BOLD:AAO7733
Forcipomyiinae|GMPJA8748-21|BIOUG66646-A08|BOLD:AAO7733
Forcipomyiinae|GMPJA8798-21|BIOUG66646-E10|BOLD:AAO7733
Forcipomyiinae|GMPJA8795-21|BIOUG66646-E07|BOLD:AAO7733
Forcipomyiinae|GMPJA10247-21|BIOUG70232-F01|BOLD:AAO7733
Forcipomyiinae|GMPJA10246-21|BIOUG70232-E12|BOLD:AAO7733
Forcipomyiinae|GMPJA2049-21|BIOUG65677-A05|BOLD:AAO7733
Forcipomyiinae|GMPJA1995-21|BIOUG65676-D10|BOLD:AAO7733
Forcipomyiinae|GMPJA3031-21|BIOUG65686-H02|BOLD:AAO7733
Forcipomyiinae|GMPJA4574-21|BIOUG66603-B02|BOLD:AAO7733
Forcipomyiinae|GMPJA8510-21|BIOUG66643-E07|BOLD:AAO7733
Forcipomyiinae|GMPJA3692-21|BIOUG65693-G10|BOLD:AAO7733
Forcipomyiinae|GMPJA6289-21|BIOUG66620-B07|BOLD:AAO7733
Forcipomyiinae|GMPJA5234-21|BIOUG66609-A09|BOLD:AAO7733
Forcipomyiinae|GMPJA8628-21|BIOUG66644-G06|BOLD:AAO7733
Forcipomyiinae|GMPJA8620-21|BIOUG66644-F10|BOLD:AAO7733
Forcipomyiinae|GMPJA1774-21|BIOUG65674-B03|BOLD:AAO7733
Forcipomyiinae|GMPJA1731-21|BIOUG65673-F07|BOLD:AAO7733
Forcipomyiinae|GMPJA8744-21|BIOUG66646-A04|BOLD:AAO7733
Forcipomyiinae|GMPJA8743-21|BIOUG66646-A03|BOLD:AAO7733
Forcipomyiinae|GMPJA8807-21|BIOUG66646-F07|BOLD:AAO7733
Forcipomyiinae|GMPJA8805-21|BIOUG66646-F05|BOLD:AAO7733
Forcipomyiinae|GMPJA10355-21|BIOUG70233-G02|BOLD:AAO7733
Forcipomyiinae|GMPJA10309-21|BIOUG70233-C04|BOLD:AAO7733
Forcipomyiinae|GMPJA10226-21|BIOUG70232-D04|BOLD:AAO7733
Forcipomyiinae|GMPJA10207-21|BIOUG70232-B09|BOLD:AAO7733
Forcipomyiinae|GMPJA1560-21|BIOUG65671-H02|BOLD:AAO7733
Forcipomyiinae|GMPJA1511-21|BIOUG65671-D01|BOLD:AAO7733
Forcipomyiinae|GMPJA7855-21|BIOUG66636-F05|BOLD:AAO7733
Forcipomyiinae|GMPJA7974-21|BIOUG66637-H05|BOLD:AAO7733
Forcipomyiinae|GMPJA8474-21|BIOUG66643-B07|BOLD:AAO7733
Forcipomyiinae|GMPJA7694-21|BIOUG66634-H10|BOLD:AAO7733
Forcipomyiinae|GMPJA7403-21|BIOUG66631-H04|BOLD:AAO7733
Forcipomyiinae|GMPJA2507-21|BIOUG65681-G11|BOLD:AAO7733
Forcipomyiinae|GMPJA7474-21|BIOUG66632-F04|BOLD:AAO7733
Forcipomyiinae|GMPJA7313-21|BIOUG66630-H09|BOLD:AAO7733
Forcipomyiinae|GMPJA7558-21|BIOUG66633-E05|BOLD:AAO7733
Forcipomyiinae|GMPJA8035-21|BIOUG66638-E07|BOLD:AAO7733
Forcipomyiinae|GMPJA8094-21|BIOUG66639-B07|BOLD:AAO7733
Forcipomyiinae|GMPJA7644-21|BIOUG66634-D08|BOLD:AAO7733
Forcipomyiinae|GMPJA8475-21|BIOUG66643-B08|BOLD:AAO7733
Forcipomyiinae|GMPJA8410-21|BIOUG66642-E02|BOLD:AAO7733
Forcipomyiinae|GMPJA8777-21|BIOUG66646-D01|BOLD:AAO7733
Forcipomyiinae|GMPJA8766-21|BIOUG66646-C02|BOLD:AAO7733
Forcipomyiinae|GMPJA10212-21|BIOUG70232-C02|BOLD:AAO7733
Forcipomyiinae|GMPJA8821-21|BIOUG66646-G09|BOLD:AAO7733
Forcipomyiinae|GMPJA10630-21|BIOUG70236-F04|BOLD:AAO7733
Forcipomyiinae|GMPJA10359-21|BIOUG70233-G06|BOLD:AAO7733
Forcipomyiinae|GMPJA2072-21|BIOUG65677-C04|BOLD:AAO7733
Forcipomyiinae|GMPJA2065-21|BIOUG65677-B09|BOLD:AAO7733
Forcipomyiinae|GMPJA7737-21|BIOUG66635-D06|BOLD:AAO7733
Forcipomyiinae|GMPJA8116-21|BIOUG66639-D05|BOLD:AAO7733
Forcipomyiinae|GMPJA8586-21|BIOUG66644-C12|BOLD:AAO7733
Forcipomyiinae|GMPJA7237-21|BIOUG66630-B05|BOLD:AAO7733
Forcipomyiinae|GMPJA7681-21|BIOUG66634-G09|BOLD:AAO7733
Forcipomyiinae|GMPJA7640-21|BIOUG66634-D04|BOLD:AAO7733
Forcipomyiinae|GMPJA8018-21|BIOUG66638-D02|BOLD:AAO7733
Forcipomyiinae|GMPJA7764-21|BIOUG66635-F09|BOLD:AAO7733
Forcipomyiinae|GMPJA7740-21|BIOUG66635-D09|BOLD:AAO7733
Forcipomyiinae|GMPJA7948-21|BIOUG66637-F03|BOLD:AAO7733
Forcipomyiinae|GMPJA7798-21|BIOUG66636-A08|BOLD:AAO7733
Forcipomyiinae|GMPJA8199-21|BIOUG66640-C05|BOLD:AAO7733
Forcipomyiinae|GMPJA8577-21|BIOUG66644-C03|BOLD:AAO7733
Forcipomyiinae|GMPJA8286-21|BIOUG66641-B09|BOLD:AAO7733
Forcipomyiinae|GMPJA8251-21|BIOUG66640-G09|BOLD:AAO7733
Forcipomyiinae|GMPJA7396-21|BIOUG66631-G09|BOLD:AAO7733
Forcipomyiinae|GMPJA7380-21|BIOUG66631-F05|BOLD:AAO7733
Forcipomyiinae|GMPJA8382-21|BIOUG66642-B10|BOLD:AAO7733
Forcipomyiinae|GMPJA8296-21|BIOUG66641-C07|BOLD:AAO7733
Forcipomyiinae|GMPJA7433-21|BIOUG66632-B11|BOLD:AAO7733
Forcipomyiinae|GMPJA7425-21|BIOUG66632-B03|BOLD:AAO7733
Forcipomyiinae|GMPJA8470-21|BIOUG66643-B03|BOLD:AAO7733
Forcipomyiinae|GMPJA8779-21|BIOUG66646-D03|BOLD:AAO7733
Forcipomyiinae|GMPJA8816-21|BIOUG66646-G04|BOLD:AAO7733
Forcipomyiinae|GMPJA3146-21|BIOUG65688-A11|BOLD:AAO7733
Forcipomyiinae|GMPJA5163-21|BIOUG66608-C09|BOLD:AAO7733
Forcipomyiinae|GMPJA10661-21|BIOUG70236-H11|BOLD:AAO7733
Forcipomyiinae|GMPJA6762-21|BIOUG66625-B05|BOLD:AAO7733
Forcipomyiinae|GMPJA8054-21|BIOUG66638-G02|BOLD:AAO7733
[illegible]

Forcipomyiinae|GMPJA10661-21|BIOUG70236-H11|BOLD:AAO7733
Forcipomyiinae|GMPJA6762-21|BIOUG66625-B05|BOLD:AAO7733
Forcipomyiinae|GMPJA8054-21|BIOUG66638-G02|BOLD:AAO7733
Forcipomyiinae|GMPJA7416-21|BIOUG66632-A06|BOLD:AAO7733
Forcipomyiinae|GMPJA7985-21|BIOUG66638-A05|BOLD:AAO7733
Forcipomyiinae|GMPJA8246-21|BIOUG66640-G04|BOLD:AAO7733
Forcipomyiinae|GMPJA8364-21|BIOUG66642-A04|BOLD:AAO7733
Forcipomyiinae|GMPJA8618-21|BIOUG66644-F08|BOLD:AAO7733
Forcipomyiinae|GMPJA8617-21|BIOUG66644-F07|BOLD:AAO7733
Forcipomyiinae|GMPJA8615-21|BIOUG66644-F05|BOLD:AAO7733
Forcipomyiinae|GMPJA7785-21|BIOUG66635-H06|BOLD:AAO7733
Forcipomyiinae|GMPJA7428-21|BIOUG66632-B06|BOLD:AAO7733
Forcipomyiinae|GMPJA5511-21|BIOUG66612-A01|BOLD:AAO7733
Forcipomyiinae|GMPJA8680-21|BIOUG66645-C11|BOLD:AAO7733
Forcipomyiinae|GMPJA8625-21|BIOUG66644-G03|BOLD:AAO7733
Forcipomyiinae|GMPJA1150-21|BIOUG65667-E08|BOLD:AAO7733
Forcipomyiinae|GMPJA8791-21|BIOUG66646-E03|BOLD:AAO7733
Forcipomyiinae|GMPJA2012-21|BIOUG65676-F03|BOLD:AAO7733
Forcipomyiinae|GMPJA2488-21|BIOUG65681-F04|BOLD:AAO7733
Forcipomyiinae|GMPJA8783-21|BIOUG66646-D07|BOLD:AAO7733
Forcipomyiinae|GMPJA8739-21|BIOUG66645-H10|BOLD:AAO7733
Forcipomyiinae|GMPJA8728-21|BIOUG66645-G11|BOLD:AAO7733
Forcipomyiinae|GMPJA8707-21|BIOUG66645-F02|BOLD:AAO7733
Forcipomyiinae|GMPJA7902-21|BIOUG66637-B05|BOLD:AAO7733
Forcipomyiinae|GMPJA7889-21|BIOUG66637-A04|BOLD:AAO7733
Forcipomyiinae|GMPJA7331-21|BIOUG66631-B04|BOLD:AAO7733
Forcipomyiinae|GMPJA7324-21|BIOUG66631-A09|BOLD:AAO7733
Forcipomyiinae|GMPJA7304-21|BIOUG66630-G12|BOLD:AAO7733
Forcipomyiinae|GMPJA7289-21|BIOUG66630-F09|BOLD:AAO7733
Forcipomyiinae|GMPJA7970-21|BIOUG66637-H01|BOLD:AAO7733
Forcipomyiinae|GMPJA7495-21|BIOUG66632-H01|BOLD:AAO7733
Forcipomyiinae|GMPJA7492-21|BIOUG66632-G10|BOLD:AAO7733
Forcipomyiinae|GMPJA7353-21|BIOUG66631-D02|BOLD:AAO7733
Forcipomyiinae|GMPJA7647-21|BIOUG66634-D11|BOLD:AAO7733
Forcipomyiinae|GMPJA7549-21|BIOUG66633-D08|BOLD:AAO7733
Forcipomyiinae|GMPJA8007-21|BIOUG66638-C03|BOLD:AAO7733
Forcipomyiinae|GMPJA7972-21|BIOUG66637-H03|BOLD:AAO7733
Forcipomyiinae|GMPJA8531-21|BIOUG66643-G04|BOLD:AAO7733
Forcipomyiinae|GMPJA8452-21|BIOUG66642-H08|BOLD:AAO7733
Forcipomyiinae|GMPJA8448-21|BIOUG66642-H04|BOLD:AAO7733
Forcipomyiinae|GMPJA8425-21|BIOUG66642-F05|BOLD:AAO7733
Forcipomyiinae|GMPJA8086-21|BIOUG66639-A11|BOLD:AAO7733
Forcipomyiinae|GMPJA8069-21|BIOUG66638-H05|BOLD:AAO7733
Forcipomyiinae|GMPJA8309-21|BIOUG66641-D08|BOLD:AAO7733
Forcipomyiinae|GMPJA7818-21|BIOUG66636-C04|BOLD:AAO7733
Forcipomyiinae|GMPJA7816-21|BIOUG66636-C02|BOLD:AAO7733
Forcipomyiinae|GMPJA7653-21|BIOUG66634-E05|BOLD:AAO7733
Forcipomyiinae|GMPJA4499-21|BIOUG66602-C10|BOLD:AAO7733
Forcipomyiinae|GMPJA10235-21|BIOUG70232-E01|BOLD:AAO7733
Forcipomyiinae|GMPJA10228-21|BIOUG70232-D06|BOLD:AAO7733
Forcipomyiinae|GMPJA3417-21|BIOUG65690-H08|BOLD:AAO7733
Forcipomyiinae|GMPJA8108-21|BIOUG66639-C09|BOLD:AAO7733
Forcipomyiinae|GMPJA8092-21|BIOUG66639-B05|BOLD:AAO7733
Forcipomyiinae|GMPJA8416-21|BIOUG66642-E08|BOLD:AAO7733
Forcipomyiinae|GMPJA8407-21|BIOUG66642-D11|BOLD:AAO7733
Forcipomyiinae|GMPJA8406-21|BIOUG66642-D10|BOLD:AAO7733
Forcipomyiinae|GMPJA8391-21|BIOUG66642-C07|BOLD:AAO7733
Forcipomyiinae|GMPJA8373-21|BIOUG66642-B01|BOLD:AAO7733
Forcipomyiinae|GMPJA8294-21|BIOUG66641-C05|BOLD:AAO7733
Forcipomyiinae|GMPJA8287-21|BIOUG66641-B10|BOLD:AAO7733
Forcipomyiinae|GMPJA8259-21|BIOUG66640-H05|BOLD:AAO7733
Forcipomyiinae|GMPJA8253-21|BIOUG66640-G11|BOLD:AAO7733
Forcipomyiinae|GMPJA8245-21|BIOUG66640-G03|BOLD:AAO7733
Forcipomyiinae|GMPJA8225-21|BIOUG66640-E07|BOLD:AAO7733
Forcipomyiinae|GMPJA8211-21|BIOUG66640-D05|BOLD:AAO7733
Forcipomyiinae|GMPJA8168-21|BIOUG66639-H09|BOLD:AAO7733
Forcipomyiinae|GMPJA8156-21|BIOUG66639-G09|BOLD:AAO7733
Forcipomyiinae|GMPJA8126-21|BIOUG66639-E03|BOLD:AAO7733
Forcipomyiinae|GMPJA8125-21|BIOUG66639-E02|BOLD:AAO7733
Forcipomyiinae|GMPJA1531-21|BIOUG65671-E09|BOLD:AAO7733
Forcipomyiinae|GMPJA1014-21|BIOUG65666-B03|BOLD:AAO7733
Forcipomyiinae|GMPJA3459-21|BIOUG65691-D03|BOLD:AAO7733
Forcipomyiinae|GMPJA4532-21|BIOUG66602-F07|BOLD:AAO7733
Forcipomyiinae|GMPJA8115-21|BIOUG66639-D04|BOLD:AAO7733
Forcipomyiinae|GMPJA8113-21|BIOUG66639-D02|BOLD:AAO7733
Forcipomyiinae|GMPJA8702-21|BIOUG66645-E09|BOLD:AAO7733
Forcipomyiinae|GMPJA8662-21|BIOUG66645-B05|BOLD:AAO7733
Forcipomyiinae|GMPJA8629-21|BIOUG66644-G07|BOLD:AAO7733
Forcipomyiinae|GMPJA8549-21|BIOUG66643-H10|BOLD:AAO7733
Forcipomyiinae|GMPJA8327-21|BIOUG66641-F02|BOLD:AAO7733
Forcipomyiinae|GMPJA8310-21|BIOUG66641-D09|BOLD:AAO7733
Forcipomyiinae|GMPJA7278-21|BIOUG66630-E10|BOLD:AAO7733
Forcipomyiinae|GMPJA7277-21|BIOUG66630-E09|BOLD:AAO7733
Forcipomyiinae|GMPJA7730-21|BIOUG66635-C11|BOLD:AAO7733
Forcipomyiinae|GMPJA7721-21|BIOUG66635-C02|BOLD:AAO7733
Forcipomyiinae|GMPJA7707-21|BIOUG66635-A12|BOLD:AAO7733
Forcipomyiinae|GMPJA7222-21|BIOUG66630-A02|BOLD:AAO7733
Forcipomyiinae|GMPJA6883-21|BIOUG66626-D07|BOLD:AAO7733
Forcipomyiinae|GMPJA7382-21|BIOUG66631-F07|BOLD:AAO7733
Forcipomyiinae|GMPJA6766-21|BIOUG66625-B09|BOLD:AAO7733
Forcipomyiinae|GMPJA6708-21|BIOUG66624-E10|BOLD:AAO7733
Forcipomyiinae|GMPJA6619-21|BIOUG66623-F04|BOLD:AAO7733
Forcipomyiinae|GMPJA6519-21|BIOUG66622-E11|BOLD:AAO7733
Forcipomyiinae|GMPJA6724-21|BIOUG66624-G02|BOLD:AAO7733
Forcipomyiinae|GMPJA6255-21|BIOUG66619-G08|BOLD:AAO7733
Forcipomyiinae|GMPJA1699-21|BIOUG65673-C11|BOLD:AAO7733
Forcipomyiinae|GMPJA2200-21|BIOUG65678-F01|BOLD:AAO7733
Forcipomyiinae|GMPJA2477-21|BIOUG65681-E05|BOLD:AAO7733
Forcipomyiinae|GMPJA8257-21|BIOUG66640-H03|BOLD:AAO7733
Forcipomyiinae|GMPJA8606-21|BIOUG66644-E08|BOLD:AAO7733
Forcipomyiinae|GMPJA8721-21|BIOUG66645-G04|BOLD:AAO7733
Forcipomyiinae|GMPJA8698-21|BIOUG66645-E05|BOLD:AAO7733
Forcipomyiinae|GMPJA2149-21|BIOUG65678-A10|BOLD:AAO7733
[illegible]

Forcipomyiinae|GMPJA8721-21|BIOUG66645-G04|BOLD:AAO7733
Forcipomyiinae|GMPJA8698-21|BIOUG66645-E05|BOLD:AAO7733
Forcipomyiinae|GMPJA2149-21|BIOUG65678-A10|BOLD:AAO7733
Forcipomyiinae|GMPJA8753-21|BIOUG66646-B01|BOLD:AAO7733
Forcipomyiinae|GMPJA8303-21|BIOUG66641-D02|BOLD:AAO7733
Forcipomyiinae|GMPJA6715-21|BIOUG66624-F05|BOLD:AAO7733
Forcipomyiinae|GMPJA7270-21|BIOUG66630-E02|BOLD:AAO7733
Forcipomyiinae|GMPJA10211-21|BIOUG70232-C01|BOLD:AAO7733
Forcipomyiinae|GMPJA8026-21|BIOUG66638-D10|BOLD:AAO7733
Forcipomyiinae|GMPJA7803-21|BIOUG66636-B01|BOLD:AAO7733
Forcipomyiinae|GMPJA4490-21|BIOUG66602-C01|BOLD:AAO7733
Syrphidae|GMPJA8878-21|BIOUG63985-F01|BOLD:ACA6567
Phoridae|GMPJA7473-21|BIOUG66632-F03|BOLD:ACG0915
Phoridae|GMPJA6900-21|BIOUG66626-E12|BOLD:ACG0915
Phoridae|GMPJA4055-21|BIOUG65697-F05|BOLD:ACG0915
Phoridae|GMPJA3440-21|BIOUG65691-B08|BOLD:ACG0915
Phoridae|GMPJA10657-21|BIOUG70236-H07|BOLD:ACG0915
Phoridae|GMPJA10572-21|BIOUG70236-A06|BOLD:ACG0915
Phoridae|GMPJA3138-21|BIOUG65688-A03|BOLD:ACG0915
Phoridae|GMPJA8670-21|BIOUG66645-C01|BOLD:ACG0915
Phoridae|GMPJA8478-21|BIOUG66643-B11|BOLD:ACG0915
Phoridae|GMPJA6646-21|BIOUG66623-H07|BOLD:ACG0915
Phoridae|GMPJA5079-21|BIOUG66607-D08|BOLD:ACG0915
Phoridae|GMPJA5064-21|BIOUG66607-C05|BOLD:ACG0915
Ceratopogonidae|GMPJA5147-21|BIOUG66608-B05|BOLD:ACP3824
Ceratopogonidae|GMPJA6554-21|BIOUG66622-H10|BOLD:ACP3824
Ceratopogonidae|GMPJA5408-21|BIOUG66610-H04|BOLD:ACP3824
Ceratopogonidae|GMPJA4172-21|BIOUG65698-H03|BOLD:ACP3824
Ceratopogonidae|GMPJA5465-21|BIOUG66611-E02|BOLD:ACP3824
Ceratopogonidae|GMPJA7351-21|BIOUG66631-C12|BOLD:ACP3824
Ceratopogonidae|GMPJA8558-21|BIOUG66644-A08|BOLD:ACP3824
Ceratopogonidae|GMPJA5308-21|BIOUG66609-G11|BOLD:ACP3824
Ceratopogonidae|GMPJA5515-21|BIOUG66612-A05|BOLD:ACP3824
Ceratopogonidae|GMPJA5598-21|BIOUG66612-H04|BOLD:ACP3824
Ceratopogonidae|GMPJA7405-21|BIOUG66631-H06|BOLD:ACP3824
Ceratopogonidae|GMPJA7419-21|BIOUG66632-A09|BOLD:ACP3824
Ceratopogonidae|GMPJA7239-21|BIOUG66630-B07|BOLD:ACP3824
Ceratopogonidae|GMPJA7318-21|BIOUG66631-A03|BOLD:ACP3824
Ceratopogonidae|GMPJA7627-21|BIOUG66634-C03|BOLD:ACP3824
Ceratopogonidae|GMPJA7910-21|BIOUG66637-C01|BOLD:ACP3824
Ceratopogonidae|GMPJA7929-21|BIOUG66637-D08|BOLD:ACP3824
Ceratopogonidae|GMPJA8132-21|BIOUG66639-E09|BOLD:ACP3824
Ceratopogonidae|GMPJA8174-21|BIOUG66640-A04|BOLD:ACP3824
Ceratopogonidae|GMPJA8412-21|BIOUG66642-E04|BOLD:ACP3824
Ceratopogonidae|GMPJA8769-21|BIOUG66646-C05|BOLD:ACP3824
Ceratopogonidae|GMPJA3277-21|BIOUG65689-D11|BOLD:ACP3824
Ceratopogonidae|GMPJA10642-21|BIOUG70236-G04|BOLD:ACP3824
Ceratopogonidae|GMPJA3133-21|BIOUG65687-H09|BOLD:ACP3824
Ceratopogonidae|GMPJA3200-21|BIOUG65688-F05|BOLD:ACP3824
Ceratopogonidae|GMPJA4554-21|BIOUG66602-H05|BOLD:ACP3824
Ceratopogonidae|GMPJA4556-21|BIOUG66602-H07|BOLD:ACP3824
Ceratopogonidae|GMPJA3514-21|BIOUG65691-H10|BOLD:ACP3824
Ceratopogonidae|GMPJA4268-21|BIOUG65699-H04|BOLD:ACP3824
Ceratopogonidae|GMPJA4346-21|BIOUG65700-F11|BOLD:ACP3824
Ceratopogonidae|GMPJA4374-21|BIOUG66601-A04|BOLD:ACP3824
Ceratopogonidae|GMPJA4457-21|BIOUG66601-H03|BOLD:ACP3824
Ceratopogonidae|GMPJA1814-21|BIOUG65674-E07|BOLD:ACP3824
Ceratopogonidae|GMPJA3328-21|BIOUG65690-A03|BOLD:ACP3824
Ceratopogonidae|GMPJA4440-21|BIOUG66601-F10|BOLD:ACP3824
Ceratopogonidae|GMPJA4150-21|BIOUG65698-F05|BOLD:ACP3824
Ceratopogonidae|GMPJA1821-21|BIOUG65674-F02|BOLD:ACP3824
Ceratopogonidae|GMPJA2108-21|BIOUG65677-F04|BOLD:ACP3824
Ceratopogonidae|GMPJA2376-21|BIOUG65680-D11|BOLD:ACP3824
Ceratopogonidae|GMPJA7673-21|BIOUG66634-G01|BOLD:ACP3824
Ceratopogonidae|GMPJA7692-21|BIOUG66634-H08|BOLD:ACP3824
Ceratopogonidae|GMPJA5303-21|BIOUG66609-G06|BOLD:ACP3824
Ceratopogonidae|GMPJA5287-21|BIOUG66609-F02|BOLD:ACP3824
Ceratopogonidae|GMPJA5409-21|BIOUG66610-H05|BOLD:ACP3824
Ceratopogonidae|GMPJA5342-21|BIOUG66610-B10|BOLD:ACP3824
Ceratopogonidae|GMPJA5046-21|BIOUG66607-A11|BOLD:ACP3824
Ceratopogonidae|GMPJA2015-21|BIOUG65676-F06|BOLD:ACV4194
Ceratopogonidae|GMPJA7582-21|BIOUG66633-G05|BOLD:ACV4194
Ceratopogonidae|GMPJA8594-21|BIOUG66644-D08|BOLD:ACA2845
Ceratopogonidae|GMPJA8522-21|BIOUG66643-F07|BOLD:ACA2845
Ceratopogonidae|GMPJA7916-21|BIOUG66637-C07|BOLD:ACA2845
Ceratopogonidae|GMPJA1550-21|BIOUG65671-G04|BOLD:ACA2845
Ceratopogonidae|GMPJA4124-21|BIOUG65698-D03|BOLD:ACA2845
Ceratopogonidae|GMPJA7762-21|BIOUG66635-F07|BOLD:ACA2845
Ceratopogonidae|GMPJA8385-21|BIOUG66642-C01|BOLD:ACA2845
Ceratopogonidae|GMPJA7444-21|BIOUG66632-C10|BOLD:ACA2845
Ceratopogonidae|GMPJA6754-21|BIOUG66625-A09|BOLD:ADS1386
Forcipomyiinae|GMPJA1910-21|BIOUG65675-E08|BOLD:ABW7946
Forcipomyiinae|GMPJA7233-21|BIOUG66630-B01|BOLD:ABW7946
Forcipomyiinae|GMPJA8627-21|BIOUG66644-G05|BOLD:ABW7946
Forcipomyiinae|GMPJA1719-21|BIOUG65673-E07|BOLD:ABW7946
Forcipomyiinae|GMPJA1447-21|BIOUG65670-F08|BOLD:ABW7946
Forcipomyiinae|GMPJA1888-21|BIOUG65675-C10|BOLD:ABW7946
Forcipomyiinae|GMPJA8097-21|BIOUG66639-B10|BOLD:ABW7946
Forcipomyiinae|GMPJA7566-21|BIOUG66633-F01|BOLD:ABW7946
Forcipomyiinae|GMPJA2416-21|BIOUG65680-H03|BOLD:ABW7946
Forcipomyiinae|GMPJA1387-21|BIOUG65670-A08|BOLD:ABW7946
Forcipomyiinae|GMPJA4220-21|BIOUG65699-D04|BOLD:ABW7946
Forcipomyiinae|GMPJA8654-21|BIOUG66645-A09|BOLD:ABW7946
Forcipomyiinae|GMPJA5543-21|BIOUG66612-C09|BOLD:ABW7946
Ceratopogonidae|GMPJA8651-21|BIOUG66645-A06|BOLD:ACO2890
Dasyhelea|GMPJA5525-21|BIOUG66612-B03|BOLD:ACP4835
Dasyhelea|GMPJA4652-21|BIOUG66603-H08|BOLD:ACP4835
Dasyhelea|GMPJA5419-21|BIOUG66611-A04|BOLD:ACP4835
Dasyhelea|GMPJA1200-21|BIOUG65668-A11|BOLD:ACP4835
Dasyhelea|GMPJA5447-21|BIOUG66611-C08|BOLD:ACP4835
Dasyhelea|GMPJA5347-21|BIOUG66610-C03|BOLD:ACP4835
Ceratopogonidae|GMPJA7631-21|BIOUG66634-C07|BOLD:AEK1168
Ceratopogonidae|GMPJA5574-21|BIOUG66612-F04|BOLD:ACP2441

Dasyhelea|GMPJA5347-21|BIOUG66610-C03|BOLD:ACP4835
Ceratopogonidae|GMPJA7631-21|BIOUG66634-C07|BOLD:AEK1168
Ceratopogonidae|GMPJA5574-21|BIOUG66612-F04|BOLD:ACP2441
Ceratopogonidae|GMPJA3044-21|BIOUG65687-A04|BOLD:ACP2441
Ceratopogonidae|GMPJA6801-21|BIOUG66625-E08|BOLD:ACP2441
Ceratopogonidae|GMPJA7462-21|BIOUG66632-E04|BOLD:ACP2441
Ceratopogonidae|GMPJA7811-21|BIOUG66636-B09|BOLD:ACP2441
Ceratopogonidae|GMPJA4082-21|BIOUG65697-H08|BOLD:ACP2441
Ceratopogonidae|GMPJA3174-21|BIOUG65688-D03|BOLD:ACP2441
Ceratopogonidae|GMPJA5112-21|BIOUG66607-G05|BOLD:ACP2441
Ceratopogonidae|GMPJA7323-21|BIOUG66631-A08|BOLD:AEJ0752
Ceratopogonidae|GMPJA8688-21|BIOUG66645-D07|BOLD:ACP4409
Ceratopogonidae|GMPJA7727-21|BIOUG66635-C08|BOLD:ACP4192
Ceratopogonidae|GMPJA7650-21|BIOUG66634-E02|BOLD:ACF8637
Ceratopogonidae|GMPJA7440-21|BIOUG66632-C06|BOLD:ACF8637
Ceratopogonidae|GMPJA8105-21|BIOUG66639-C06|BOLD:ACF8637
Ceratopogonidae|GMPJA7939-21|BIOUG66637-E06|BOLD:ACF8637
Ceratopogonidae|GMPJA7925-21|BIOUG66637-D04|BOLD:ACF8637
Ceratopogonidae|GMPJA7890-21|BIOUG66637-A05|BOLD:ACF8637
Ceratopogonidae|GMPJA7869-21|BIOUG66636-G07|BOLD:ACF8637
Ceratopogonidae|GMPJA7547-21|BIOUG66633-D06|BOLD:ACF8637
Ceratopogonidae|GMPJA7350-21|BIOUG66631-C11|BOLD:ACF8637
Ceratopogonidae|GMPJA7328-21|BIOUG66631-B01|BOLD:ACF8637
Ceratopogonidae|GMPJA7238-21|BIOUG66630-B06|BOLD:ACF8637
Ceratopogonidae|GMPJA7682-21|BIOUG66634-G10|BOLD:ACF8637
Ceratopogonidae|GMPJA8359-21|BIOUG66641-H10|BOLD:ACF8637
Ceratopogonidae|GMPJA8240-21|BIOUG66640-F10|BOLD:ACF8637
Ceratopogonidae|GMPJA8188-21|BIOUG66640-B06|BOLD:ACF8637
Ceratopogonidae|GMPJA8107-21|BIOUG66639-C08|BOLD:ACF8637
Ceratopogonidae|GMPJA8445-21|BIOUG66642-H01|BOLD:ACF8637
Ceratopogonidae|GMPJA8427-21|BIOUG66642-F07|BOLD:ACF8637
Ceratopogonidae|GMPJA8493-21|BIOUG66643-D02|BOLD:ACF8637
Ceratopogonidae|GMPJA8486-21|BIOUG66643-C07|BOLD:ACF8637
Ceratopogonidae|GMPJA8742-21|BIOUG66646-A02|BOLD:ACF8637
Ceratopogonidae|GMPJA8555-21|BIOUG66644-A05|BOLD:ACF8637
Ceratopogonidae|GMPJA8782-21|BIOUG66646-D06|BOLD:ACF8637
Ceratopogonidae|GMPJA8775-21|BIOUG66646-C11|BOLD:ACF8637
Ceratopogonidae|GMPJA9169-21|BIOUG66650-A04|BOLD:ACF8637
Ceratopogonidae|GMPJA8789-21|BIOUG66646-E01|BOLD:ACF8637
Ceratopogonidae|GMPJA10592-21|BIOUG70236-C02|BOLD:ACF8637
Ceratopogonidae|GMPJA10593-21|BIOUG70236-C03|BOLD:ACF8637
Ceratopogonidae|GMPJA203-21|BIOUG65657-E11|BOLD:ACF8637
Ceratopogonidae|GMPJA446-21|BIOUG65660-B05|BOLD:ACF8637
Ceratopogonidae|GMPJA1328-21|BIOUG65669-D08|BOLD:ACF8637
Ceratopogonidae|GMPJA1199-21|BIOUG65668-A10|BOLD:ACF8637
Ceratopogonidae|GMPJA1836-21|BIOUG65674-G05|BOLD:ACF8637
Ceratopogonidae|GMPJA1549-21|BIOUG65671-G03|BOLD:ACF8637
Ceratopogonidae|GMPJA1351-21|BIOUG65669-F07|BOLD:ACF8637
Ceratopogonidae|GMPJA1009-21|BIOUG65666-A10|BOLD:ACF8637
Ceratopogonidae|GMPJA10548-21|BIOUG70235-G05|BOLD:ACF8637
Ceratopogonidae|GMPJA8806-21|BIOUG66646-F06|BOLD:ACF8637
Ceratopogonidae|GMPJA8655-21|BIOUG66645-A10|BOLD:ACF8637
Ceratopogonidae|GMPJA7868-21|BIOUG66636-G06|BOLD:ACF8637
Ceratopogonidae|GMPJA7610-21|BIOUG66634-A10|BOLD:ACF8637
Ceratopogonidae|GMPJA7978-21|BIOUG66637-H09|BOLD:ACF8637
Ceratopogonidae|GMPJA6656-21|BIOUG66624-A06|BOLD:ACF8637
Ceratopogonidae|GMPJA7436-21|BIOUG66632-C02|BOLD:ACF8637
Ceratopogonidae|GMPJA6588-21|BIOUG66623-C09|BOLD:ACF8637
Ceratopogonidae|GMPJA6714-21|BIOUG66624-F04|BOLD:ACF8637
Ceratopogonidae|GMPJA6458-21|BIOUG66621-H09|BOLD:ACF8637
Ceratopogonidae|GMPJA6875-21|BIOUG66626-C11|BOLD:ACF8637
Ceratopogonidae|GMPJA7448-21|BIOUG66632-D02|BOLD:ACF8637
Ceratopogonidae|GMPJA6440-21|BIOUG66621-G03|BOLD:ACF8637
Ceratopogonidae|GMPJA5341-21|BIOUG66610-B09|BOLD:ACF8637
Ceratopogonidae|GMPJA154-21|BIOUG65657-A10|BOLD:AEA1475
Ceratopogonidae|GMPJA8037-21|BIOUG66638-E09|BOLD:ACV3267
Ceratopogonidae|GMPJA8489-21|BIOUG66643-C10|BOLD:ACV3267
Ceratopogonidae|GMPJA7967-21|BIOUG66637-G10|BOLD:ACV3267
Ceratopogonidae|GMPJA7386-21|BIOUG66631-F11|BOLD:ACV3267
Ceratopogonidae|GMPJA8656-21|BIOUG66645-A11|BOLD:ACR6834
Ceratopogonidae|GMPJA7500-21|BIOUG66632-H06|BOLD:ACR6834
Ceratopogonidae|GMPJA7660-21|BIOUG66634-E12|BOLD:ACR6834
Ceratopogonidae|GMPJA7884-21|BIOUG66636-H10|BOLD:ACR6834
Ceratopogonidae|GMPJA7662-21|BIOUG66634-F02|BOLD:ACR6834
Ceratopogonidae|GMPJA7879-21|BIOUG66636-H05|BOLD:ACR6834
Ceratopogonidae|GMPJA8315-21|BIOUG66641-E02|BOLD:ACR6834
Ceratopogonidae|GMPJA7254-21|BIOUG66630-C10|BOLD:ACR6834
Ceratopogonidae|GMPJA10316-21|BIOUG70233-C11|BOLD:ACR6834
Ceratopogonidae|GMPJA8405-21|BIOUG66642-D09|BOLD:ACR6834
Ceratopogonidae|GMPJA7971-21|BIOUG66637-H02|BOLD:ACR6834
Ceratopogonidae|GMPJA7320-21|BIOUG66631-A05|BOLD:ACR6834
Ceratopogonidae|GMPJA6902-21|BIOUG66626-F02|BOLD:ACR6834
Ceratopogonidae|GMPJA6856-21|BIOUG66626-B04|BOLD:ACR6834
Ceratopogonidae|GMPJA6412-21|BIOUG66621-D11|BOLD:ACR6834
Ceratopogonidae|GMPJA6479-21|BIOUG66622-B07|BOLD:ACR6834
Ceratopogonidae|GMPJA8797-21|BIOUG66646-E09|BOLD:ACR6834
Ceratopogonidae|GMPJA8222-21|BIOUG66640-E04|BOLD:ACR6834
Ceratopogonidae|GMPJA7835-21|BIOUG66636-D09|BOLD:ACR6834
Ceratopogonidae|GMPJA8096-21|BIOUG66639-B09|BOLD:ACR6834
Ceratopogonidae|GMPJA6726-21|BIOUG66624-G04|BOLD:ACR6834
Ceratopogonidae|GMPJA8569-21|BIOUG66644-B07|BOLD:ACR6834
Ceratopogonidae|GMPJA8229-21|BIOUG66640-E11|BOLD:ACR6834
Ceratopogonidae|GMPJA7477-21|BIOUG66632-F07|BOLD:ACR6834
Ceratopogonidae|GMPJA6644-21|BIOUG66623-H05|BOLD:ACR6834
Ceratopogonidae|GMPJA7892-21|BIOUG66637-A07|BOLD:ACR6834
Ceratopogonidae|GMPJA7471-21|BIOUG66632-F01|BOLD:ACR6834
Ceratopogonidae|GMPJA7486-21|BIOUG66632-G04|BOLD:ACR6834
Ceratopogonidae|GMPJA7526-21|BIOUG66633-B09|BOLD:ACR6834
Ceratopogonidae|GMPJA8036-21|BIOUG66638-E08|BOLD:ACR6834
Ceratopogonidae|GMPJA7546-21|BIOUG66633-D05|BOLD:ACR6834
Ceratopogonidae|GMPJA7604-21|BIOUG66634-A04|BOLD:ACR6834
Ceratopogonidae|GMPJA7674-21|BIOUG66634-G02|BOLD:ACR6834
Ceratopogonidae|GMPJA7756-21|BIOUG66635-F01|BOLD:ACR6834

Ceratopogonidae|GMPJA7604-21|BIOUG66634-A04|BOLD:ACR6834
Ceratopogonidae|GMPJA7674-21|BIOUG66634-G02|BOLD:ACR6834
Ceratopogonidae|GMPJA7756-21|BIOUG66635-F01|BOLD:ACR6834
Ceratopogonidae|GMPJA7938-21|BIOUG66637-E05|BOLD:ACR6834
Ceratopogonidae|GMPJA8418-21|BIOUG66642-E10|BOLD:ACR6834
Ceratopogonidae|GMPJA8434-21|BIOUG66642-G02|BOLD:ACR6834
Ceratopogonidae|GMPJA8435-21|BIOUG66642-G03|BOLD:ACR6834
Ceratopogonidae|GMPJA7464-21|BIOUG66632-E06|BOLD:ACR6834
Ceratopogonidae|GMPJA7288-21|BIOUG66630-F08|BOLD:ACR6834
Ceratopogonidae|GMPJA7229-21|BIOUG66630-A09|BOLD:ACR6834
Ceratopogonidae|GMPJA7736-21|BIOUG66635-D05|BOLD:ACR6834
Ceratopogonidae|GMPJA6447-21|BIOUG66621-G10|BOLD:ACR6834
Ceratopogonidae|GMPJA6384-21|BIOUG66621-B07|BOLD:ACR6834
Ceratopogonidae|GMPJA6618-21|BIOUG66623-F03|BOLD:ACR6834
Ceratopogonidae|GMPJA7973-21|BIOUG66637-H04|BOLD:ACR6834
Ceratopogonidae|GMPJA7901-21|BIOUG66637-B04|BOLD:ACR6834
Ceratopogonidae|GMPJA8599-21|BIOUG66644-E01|BOLD:ACR6834
Ceratopogonidae|GMPJA8191-21|BIOUG66640-B09|BOLD:ACR6834
Ceratopogonidae|GMPJA8505-21|BIOUG66643-E02|BOLD:ACR6834
Ceratopogonidae|GMPJA8547-21|BIOUG66643-H08|BOLD:ACR6834
Ceratopogonidae|GMPJA8817-21|BIOUG66646-G05|BOLD:ACR6834
Ceratopogonidae|GMPJA7559-21|BIOUG66633-E06|BOLD:ACR6834
Ceratopogonidae|GMPJA5246-21|BIOUG66609-B09|BOLD:ACR6834
Ceratopogonidae|GMPJA8520-21|BIOUG66643-F05|BOLD:AEK7036
Ceratopogonidae|GMPJA8633-21|BIOUG66644-G11|BOLD:ABY0150
Ceratopogonidae|GMPJA4588-21|BIOUG66603-C04|BOLD:ABY0150
Ceratopogonidae|GMPJA3294-21|BIOUG65689-F04|BOLD:ABY0150
Ceratopogonidae|GMPJA8814-21|BIOUG66646-G02|BOLD:ABY0150
Ceratopogonidae|GMPJA8812-21|BIOUG66646-F12|BOLD:ABY0150
Ceratopogonidae|GMPJA8726-21|BIOUG66645-G09|BOLD:ABY0150
Ceratopogonidae|GMPJA8560-21|BIOUG66644-A10|BOLD:ABY0150
Ceratopogonidae|GMPJA8553-21|BIOUG66644-A03|BOLD:ABY0150
Ceratopogonidae|GMPJA8386-21|BIOUG66642-C02|BOLD:ABY0150
Ceratopogonidae|GMPJA2341-21|BIOUG65680-A12|BOLD:ABY0150
Ceratopogonidae|GMPJA1562-21|BIOUG65671-H04|BOLD:ABY0150
Ceratopogonidae|GMPJA1443-21|BIOUG65670-F04|BOLD:ABY0150
Ceratopogonidae|GMPJA1402-21|BIOUG65670-B11|BOLD:ABY0150
Ceratopogonidae|GMPJA8215-21|BIOUG66640-D09|BOLD:ABY0150
Ceratopogonidae|GMPJA8607-21|BIOUG66644-E09|BOLD:ABY0150
Ceratopogonidae|GMPJA8151-21|BIOUG66639-G04|BOLD:ABY0150
Ceratopogonidae|GMPJA8140-21|BIOUG66639-F05|BOLD:ABY0150
Ceratopogonidae|GMPJA7919-21|BIOUG66637-C10|BOLD:ABY0150
Ceratopogonidae|GMPJA7883-21|BIOUG66636-H09|BOLD:ABY0150
Ceratopogonidae|GMPJA7864-21|BIOUG66636-G02|BOLD:ABY0150
Ceratopogonidae|GMPJA7804-21|BIOUG66636-B02|BOLD:ABY0150
Ceratopogonidae|GMPJA7639-21|BIOUG66634-D03|BOLD:ABY0150
Ceratopogonidae|GMPJA7608-21|BIOUG66634-A08|BOLD:ABY0150
Ceratopogonidae|GMPJA1350-21|BIOUG65669-F06|BOLD:ABY0150
Ceratopogonidae|GMPJA3546-21|BIOUG65692-C07|BOLD:ABY0150
Ceratopogonidae|GMPJA4508-21|BIOUG66602-D07|BOLD:ABY0150
Ceratopogonidae|GMPJA10259-21|BIOUG70232-G01|BOLD:ABY0150
Ceratopogonidae|GMPJA8048-21|BIOUG66638-F08|BOLD:ABY0150
Ceratopogonidae|GMPJA8022-21|BIOUG66638-D06|BOLD:ABY0150
Ceratopogonidae|GMPJA7537-21|BIOUG66633-C08|BOLD:ABY0150
Ceratopogonidae|GMPJA7225-21|BIOUG66630-A05|BOLD:ABY0150
Ceratopogonidae|GMPJA7365-21|BIOUG66631-E02|BOLD:ABY0150
Ceratopogonidae|GMPJA6765-21|BIOUG66625-B08|BOLD:ABY0150
Ceratopogonidae|GMPJA3333-21|BIOUG65690-A08|BOLD:ACP3998
Ceratopogonidae|GMPJA10575-21|BIOUG70236-A09|BOLD:ACP3998
Ceratopogonidae|GMPJA3127-21|BIOUG65687-H03|BOLD:ACP3998
Ceratopogonidae|GMPJA4135-21|BIOUG65698-E02|BOLD:ACP3998
Ceratopogonidae|GMPJA3490-21|BIOUG65691-F10|BOLD:ACP3998
Ceratopogonidae|GMPJA6183-21|BIOUG66619-A08|BOLD:ACP3998
Ceratopogonidae|GMPJA4582-21|BIOUG66603-B10|BOLD:ABX2129
Ceratopogonidae|GMPJA8400-21|BIOUG66642-D04|BOLD:ABX2129
Ceratopogonidae|GMPJA8133-21|BIOUG66639-E10|BOLD:ABX2129
Ceratopogonidae|GMPJA1398-21|BIOUG65670-B07|BOLD:ABX2129
Ceratopogonidae|GMPJA3096-21|BIOUG65687-E08|BOLD:ABX2129
Ceratopogonidae|GMPJA7485-21|BIOUG66632-G03|BOLD:ABX2129
Ceratopogonidae|GMPJA1388-21|BIOUG65670-A09|BOLD:ABX2129
Ceratopogonidae|GMPJA1796-21|BIOUG65674-D01|BOLD:ABX2129
Ceratopogonidae|GMPJA1299-21|BIOUG65669-B03|BOLD:ABX2129
Ceratopogonidae|GMPJA3995-21|BIOUG65697-A05|BOLD:ABX2129
Ceratopogonidae|GMPJA3632-21|BIOUG65693-B10|BOLD:ABX2129
Ceratopogonidae|GMPJA3406-21|BIOUG65690-G09|BOLD:ABX2129
Ceratopogonidae|GMPJA8181-21|BIOUG66640-A11|BOLD:ABX2129
Ceratopogonidae|GMPJA4109-21|BIOUG65698-B12|BOLD:ABX2129
Ceratopogonidae|GMPJA1465-21|BIOUG65670-H02|BOLD:ABX2129
Ceratopogonidae|GMPJA3218-21|BIOUG65688-G11|BOLD:ABX2129
Ceratopogonidae|GMPJA7443-21|BIOUG66632-C09|BOLD:ABX2129
Ceratopogonidae|GMPJA7373-21|BIOUG66631-E10|BOLD:ABX2129
Ceratopogonidae|GMPJA3997-21|BIOUG65697-A07|BOLD:ABX2129
Ceratopogonidae|GMPJA2300-21|BIOUG65679-F06|BOLD:ABX2129
Ceratopogonidae|GMPJA1798-21|BIOUG65674-D03|BOLD:ABX2129
Ceratopogonidae|GMPJA8001-21|BIOUG66638-B09|BOLD:ABX2129
Ceratopogonidae|GMPJA10213-21|BIOUG70232-C03|BOLD:ABX2129
Ceratopogonidae|GMPJA1658-21|BIOUG65672-H05|BOLD:ABX2129
Ceratopogonidae|GMPJA7301-21|BIOUG66630-G09|BOLD:ABX2129
Ceratopogonidae|GMPJA1868-21|BIOUG65675-B02|BOLD:ABX2129
Ceratopogonidae|GMPJA3235-21|BIOUG65689-A05|BOLD:ABX2129
Ceratopogonidae|GMPJA7456-21|BIOUG66632-D10|BOLD:ABX2129
Ceratopogonidae|GMPJA3567-21|BIOUG65692-E04|BOLD:ABX2129
Ceratopogonidae|GMPJA3299-21|BIOUG65689-F09|BOLD:ABX2129
Ceratopogonidae|GMPJA4235-21|BIOUG65699-E07|BOLD:ABX2129
Ceratopogonidae|GMPJA3590-21|BIOUG65692-G03|BOLD:ABX2129
Ceratopogonidae|GMPJA7401-21|BIOUG66631-H02|BOLD:ABX2129
Ceratopogonidae|GMPJA7776-21|BIOUG66635-G09|BOLD:ABX2129
Ceratopogonidae|GMPJA3190-21|BIOUG65688-E07|BOLD:ABX2129
Ceratopogonidae|GMPJA3251-21|BIOUG65689-B09|BOLD:ABX2129
Ceratopogonidae|GMPJA3581-21|BIOUG65692-F06|BOLD:ABX2129
Ceratopogonidae|GMPJA3663-21|BIOUG65693-E05|BOLD:ABX2129
Ceratopogonidae|GMPJA1346-21|BIOUG65669-F02|BOLD:ABX2129
Ceratopogonidae|GMPJA7750-21|BIOUG66635-E07|BOLD:ABX2129

Ceratopogonidae|GMPJA3663-21|BIOUG65693-E05|BOLD:ABX2129
Ceratopogonidae|GMPJA1346-21|BIOUG65669-F02|BOLD:ABX2129
Ceratopogonidae|GMPJA7750-21|BIOUG66635-E07|BOLD:ABX2129
Ceratopogonidae|GMPJA1912-21|BIOUG65675-E10|BOLD:ABX2129
Ceratopogonidae|GMPJA8065-21|BIOUG66638-H01|BOLD:ABX2129
Ceratopogonidae|GMPJA7628-21|BIOUG66634-C04|BOLD:ABX2129
Ceratopogonidae|GMPJA8345-21|BIOUG66641-G08|BOLD:ABX2129
Ceratopogonidae|GMPJA7852-21|BIOUG66636-F02|BOLD:ABX2129
Ceratopogonidae|GMPJA7950-21|BIOUG66637-F05|BOLD:ABX2129
Ceratopogonidae|GMPJA8091-21|BIOUG66639-B04|BOLD:ABX2129
Ceratopogonidae|GMPJA8216-21|BIOUG66640-D10|BOLD:ABX2129
Ceratopogonidae|GMPJA3466-21|BIOUG65691-D10|BOLD:ABX2129
Ceratopogonidae|GMPJA8559-21|BIOUG66644-A09|BOLD:ABX2129
Ceratopogonidae|GMPJA8565-21|BIOUG66644-B03|BOLD:ABX2129
Ceratopogonidae|GMPJA8616-21|BIOUG66644-F06|BOLD:ABX2129
Ceratopogonidae|GMPJA8685-21|BIOUG66645-D04|BOLD:ABX2129
Ceratopogonidae|GMPJA8738-21|BIOUG66645-H09|BOLD:ABX2129
Ceratopogonidae|GMPJA8745-21|BIOUG66646-A05|BOLD:ABX2129
Ceratopogonidae|GMPJA10285-21|BIOUG70233-A04|BOLD:ABX2129
Ceratopogonidae|GMPJA3112-21|BIOUG65687-F12|BOLD:ABX2129
Ceratopogonidae|GMPJA3125-21|BIOUG65687-H01|BOLD:ABX2129
Ceratopogonidae|GMPJA3222-21|BIOUG65688-H03|BOLD:ABX2129
Ceratopogonidae|GMPJA3293-21|BIOUG65689-F03|BOLD:ABX2129
Ceratopogonidae|GMPJA10627-21|BIOUG70236-F01|BOLD:ABX2129
Ceratopogonidae|GMPJA4576-21|BIOUG66603-B04|BOLD:ABX2129
Ceratopogonidae|GMPJA3062-21|BIOUG65687-B10|BOLD:ABX2129
Ceratopogonidae|GMPJA10323-21|BIOUG70233-D06|BOLD:ABX2129
Ceratopogonidae|GMPJA3227-21|BIOUG65688-H08|BOLD:ABX2129
Ceratopogonidae|GMPJA3666-21|BIOUG65693-E08|BOLD:ABX2129
Ceratopogonidae|GMPJA3668-21|BIOUG65693-E10|BOLD:ABX2129
Ceratopogonidae|GMPJA3672-21|BIOUG65693-F02|BOLD:ABX2129
Ceratopogonidae|GMPJA4462-21|BIOUG66601-H08|BOLD:ABX2129
Ceratopogonidae|GMPJA4468-21|BIOUG66602-A03|BOLD:ABX2129
Ceratopogonidae|GMPJA3263-21|BIOUG65689-C09|BOLD:ABX2129
Ceratopogonidae|GMPJA3297-21|BIOUG65689-F07|BOLD:ABX2129
Ceratopogonidae|GMPJA10554-21|BIOUG70235-G11|BOLD:ABX2129
Ceratopogonidae|GMPJA3341-21|BIOUG65690-B04|BOLD:ABX2129
Ceratopogonidae|GMPJA3487-21|BIOUG65691-F07|BOLD:ABX2129
Ceratopogonidae|GMPJA3491-21|BIOUG65691-F11|BOLD:ABX2129
Ceratopogonidae|GMPJA4272-21|BIOUG65699-H08|BOLD:ABX2129
Ceratopogonidae|GMPJA4280-21|BIOUG65700-A05|BOLD:ABX2129
Ceratopogonidae|GMPJA4314-21|BIOUG65700-D03|BOLD:ABX2129
Ceratopogonidae|GMPJA4393-21|BIOUG66601-B11|BOLD:ABX2129
Ceratopogonidae|GMPJA4415-21|BIOUG66601-D09|BOLD:ABX2129
Ceratopogonidae|GMPJA3525-21|BIOUG65692-A10|BOLD:ABX2129
Ceratopogonidae|GMPJA3547-21|BIOUG65692-C08|BOLD:ABX2129
Ceratopogonidae|GMPJA3566-21|BIOUG65692-E03|BOLD:ABX2129
Ceratopogonidae|GMPJA3568-21|BIOUG65692-E05|BOLD:ABX2129
Ceratopogonidae|GMPJA3572-21|BIOUG65692-E09|BOLD:ABX2129
Ceratopogonidae|GMPJA3606-21|BIOUG65692-H07|BOLD:ABX2129
Ceratopogonidae|GMPJA3608-21|BIOUG65692-H09|BOLD:ABX2129
Ceratopogonidae|GMPJA3648-21|BIOUG65693-D02|BOLD:ABX2129
Ceratopogonidae|GMPJA3661-21|BIOUG65693-E03|BOLD:ABX2129
Ceratopogonidae|GMPJA4034-21|BIOUG65697-D08|BOLD:ABX2129
Ceratopogonidae|GMPJA4036-21|BIOUG65697-D10|BOLD:ABX2129
Ceratopogonidae|GMPJA4046-21|BIOUG65697-E08|BOLD:ABX2129
Ceratopogonidae|GMPJA4106-21|BIOUG65698-B09|BOLD:ABX2129
Ceratopogonidae|GMPJA4143-21|BIOUG65698-E10|BOLD:ABX2129
Ceratopogonidae|GMPJA4169-21|BIOUG65698-G12|BOLD:ABX2129
Ceratopogonidae|GMPJA920-21|BIOUG65665-B04|BOLD:ABX2129
Ceratopogonidae|GMPJA1298-21|BIOUG65669-B02|BOLD:ABX2129
Ceratopogonidae|GMPJA1051-21|BIOUG65666-E04|BOLD:ABX2129
Ceratopogonidae|GMPJA1376-21|BIOUG65669-H08|BOLD:ABX2129
Ceratopogonidae|GMPJA1876-21|BIOUG65675-B10|BOLD:ABX2129
Ceratopogonidae|GMPJA1445-21|BIOUG65670-F06|BOLD:ABX2129
Ceratopogonidae|GMPJA1566-21|BIOUG65671-H08|BOLD:ABX2129
Ceratopogonidae|GMPJA1603-21|BIOUG65672-C10|BOLD:ABX2129
Ceratopogonidae|GMPJA2210-21|BIOUG65678-F11|BOLD:ABX2129
Ceratopogonidae|GMPJA2225-21|BIOUG65678-H02|BOLD:ABX2129
Ceratopogonidae|GMPJA2287-21|BIOUG65679-E05|BOLD:ABX2129
Ceratopogonidae|GMPJA2302-21|BIOUG65679-F08|BOLD:ABX2129
Ceratopogonidae|GMPJA2360-21|BIOUG65680-C07|BOLD:ABX2129
Ceratopogonidae|GMPJA1652-21|BIOUG65672-G11|BOLD:ABX2129
Ceratopogonidae|GMPJA1671-21|BIOUG65673-A07|BOLD:ABX2129
Ceratopogonidae|GMPJA1698-21|BIOUG65673-C10|BOLD:ABX2129
Ceratopogonidae|GMPJA1708-21|BIOUG65673-D08|BOLD:ABX2129
Ceratopogonidae|GMPJA2026-21|BIOUG65676-G05|BOLD:ABX2129
Ceratopogonidae|GMPJA2054-21|BIOUG65677-A10|BOLD:ABX2129
Ceratopogonidae|GMPJA2096-21|BIOUG65677-E04|BOLD:ABX2129
Ceratopogonidae|GMPJA2116-21|BIOUG65677-F12|BOLD:ABX2129
Ceratopogonidae|GMPJA3311-21|BIOUG65689-G09|BOLD:ABX2129
Ceratopogonidae|GMPJA7913-21|BIOUG66637-C04|BOLD:ABX2129
Ceratopogonidae|GMPJA4303-21|BIOUG65700-C04|BOLD:ABX2129
Ceratopogonidae|GMPJA2001-21|BIOUG65676-E04|BOLD:ABX2129
Ceratopogonidae|GMPJA4506-21|BIOUG66602-D05|BOLD:ABX2129
Ceratopogonidae|GMPJA8060-21|BIOUG66638-G08|BOLD:ABX2129
Ceratopogonidae|GMPJA8059-21|BIOUG66638-G07|BOLD:ABX2129
Ceratopogonidae|GMPJA7343-21|BIOUG66631-C04|BOLD:ABX2129
Ceratopogonidae|GMPJA7299-21|BIOUG66630-G07|BOLD:ABX2129
Ceratopogonidae|GMPJA7247-21|BIOUG66630-C03|BOLD:ABX2129
Ceratopogonidae|GMPJA7390-21|BIOUG66631-G03|BOLD:ABX2129
Ceratopogonidae|GMPJA5690-21|BIOUG66613-H01|BOLD:ABX2129
Ceratopogonidae|GMPJA5474-21|BIOUG66611-E11|BOLD:ABX2129
Ceratopogonidae|GMPJA5471-21|BIOUG66611-E08|BOLD:ABX2129
Ceratopogonidae|GMPJA5319-21|BIOUG66609-H10|BOLD:ABX2129
Ceratopogonidae|GMPJA5432-21|BIOUG66611-B05|BOLD:ABX2129
Ceratopogonidae|GMPJA5378-21|BIOUG66610-E10|BOLD:ABX2129
Ceratopogonidae|GMPJA1552-21|BIOUG65671-G06|BOLD:ABX2129
Ceratopogonidae|GMPJA4028-21|BIOUG65697-D02|BOLD:ABX2129
Ceratopogonidae|GMPJA8210-21|BIOUG66640-D04|BOLD:ABX2129
Ceratopogonidae|GMPJA3115-21|BIOUG65687-G03|BOLD:ABX2129
Ceratopogonidae|GMPJA2455-21|BIOUG65681-C07|BOLD:ABX2129
Ceratopogonidae|GMPJA5253-21|BIOUG66609-C04|BOLD:ABY5536

Ceratopogonidae|GMPJA3115-21|BIOUG65687-G03|BOLD:ABX2129
Ceratopogonidae|GMPJA2455-21|BIOUG65681-C07|BOLD:ABX2129
Ceratopogonidae|GMPJA5253-21|BIOUG66609-C04|BOLD:ABY5536
Ceratopogonidae|GMPJA7724-21|BIOUG66635-C05|BOLD:ABY5536
Ceratopogonidae|GMPJA8683-21|BIOUG66645-D02|BOLD:ABY5536
Ceratopogonidae|GMPJA3379-21|BIOUG65690-E06|BOLD:ABY5536
Ceratopogonidae|GMPJA10540-21|BIOUG70235-F09|BOLD:ABY5536
Ceratopogonidae|GMPJA8353-21|BIOUG66641-H04|BOLD:ABY5536
Ceratopogonidae|GMPJA8595-21|BIOUG66644-D09|BOLD:ABY5536
Ceratopogonidae|GMPJA3194-21|BIOUG65688-E11|BOLD:ABY5536
Ceratopogonidae|GMPJA4306-21|BIOUG65700-C07|BOLD:ABY5536
Ceratopogonidae|GMPJA4541-21|BIOUG66602-G04|BOLD:ABY5536
Ceratopogonidae|GMPJA3449-21|BIOUG65691-C05|BOLD:ABY5536
Ceratopogonidae|GMPJA3215-21|BIOUG65688-G08|BOLD:ABY5536
Ceratopogonidae|GMPJA3673-21|BIOUG65693-F03|BOLD:ABY5536
Ceratopogonidae|GMPJA3472-21|BIOUG65691-E04|BOLD:ABY5536
Ceratopogonidae|GMPJA1779-21|BIOUG65674-B08|BOLD:ABY5536
Ceratopogonidae|GMPJA2102-21|BIOUG65677-E10|BOLD:ABY5536
Ceratopogonidae|GMPJA2110-21|BIOUG65677-F06|BOLD:ABY5536
Ceratopogonidae|GMPJA1323-21|BIOUG65669-D03|BOLD:ABY5536
Ceratopogonidae|GMPJA4162-21|BIOUG65698-G05|BOLD:ABY5536
Ceratopogonidae|GMPJA1600-21|BIOUG65672-C07|BOLD:ABY5536
Ceratopogonidae|GMPJA2095-21|BIOUG65677-E03|BOLD:ABY5536
Ceratopogonidae|GMPJA4171-21|BIOUG65698-H02|BOLD:ABY5536
Ceratopogonidae|GMPJA4040-21|BIOUG65697-E02|BOLD:ABY5536
Ceratopogonidae|GMPJA3925-21|BIOUG65696-C06|BOLD:ABY5536
Ceratopogonidae|GMPJA3535-21|BIOUG65692-B08|BOLD:ABY5536
Ceratopogonidae|GMPJA4396-21|BIOUG66601-C02|BOLD:ABY5536
Ceratopogonidae|GMPJA4317-21|BIOUG65700-D06|BOLD:ABY5536
Ceratopogonidae|GMPJA4304-21|BIOUG65700-C05|BOLD:ABY5536
Ceratopogonidae|GMPJA4283-21|BIOUG65700-A08|BOLD:ABY5536
Ceratopogonidae|GMPJA3377-21|BIOUG65690-E04|BOLD:ABY5536
Ceratopogonidae|GMPJA3309-21|BIOUG65689-G07|BOLD:ABY5536
Ceratopogonidae|GMPJA3179-21|BIOUG65688-D08|BOLD:ABY5536
Ceratopogonidae|GMPJA3091-21|BIOUG65687-E03|BOLD:ABY5536
Ceratopogonidae|GMPJA3018-21|BIOUG65686-G01|BOLD:ABY5536
Ceratopogonidae|GMPJA4577-21|BIOUG66603-B05|BOLD:ABY5536
Ceratopogonidae|GMPJA3423-21|BIOUG65691-A03|BOLD:ABY5536
Ceratopogonidae|GMPJA3424-21|BIOUG65691-A04|BOLD:ABY5536
Ceratopogonidae|GMPJA10585-21|BIOUG70236-B07|BOLD:ABY5536
Ceratopogonidae|GMPJA3305-21|BIOUG65689-G03|BOLD:ABY5536
Ceratopogonidae|GMPJA3219-21|BIOUG65688-G12|BOLD:ABY5536
Ceratopogonidae|GMPJA3121-21|BIOUG65687-G09|BOLD:ABY5536
Ceratopogonidae|GMPJA3180-21|BIOUG65688-D09|BOLD:ABY5536
Ceratopogonidae|GMPJA8785-21|BIOUG66646-D09|BOLD:ABY5536
Ceratopogonidae|GMPJA8731-21|BIOUG66645-H02|BOLD:ABY5536
Ceratopogonidae|GMPJA8676-21|BIOUG66645-C07|BOLD:ABY5536
Ceratopogonidae|GMPJA8497-21|BIOUG66643-D06|BOLD:ABY5536
Ceratopogonidae|GMPJA8226-21|BIOUG66640-E08|BOLD:ABY5536
Ceratopogonidae|GMPJA8180-21|BIOUG66640-A10|BOLD:ABY5536
Ceratopogonidae|GMPJA8602-21|BIOUG66644-E04|BOLD:ABY5536
Ceratopogonidae|GMPJA7951-21|BIOUG66637-F06|BOLD:ABY5536
Ceratopogonidae|GMPJA7871-21|BIOUG66636-G09|BOLD:ABY5536
Ceratopogonidae|GMPJA7829-21|BIOUG66636-D03|BOLD:ABY5536
Ceratopogonidae|GMPJA7823-21|BIOUG66636-C09|BOLD:ABY5536
Ceratopogonidae|GMPJA7766-21|BIOUG66635-F11|BOLD:ABY5536
Ceratopogonidae|GMPJA7300-21|BIOUG66630-G08|BOLD:ABY5536
Ceratopogonidae|GMPJA7272-21|BIOUG66630-E04|BOLD:ABY5536
Ceratopogonidae|GMPJA7454-21|BIOUG66632-D08|BOLD:ABY5536
Ceratopogonidae|GMPJA5486-21|BIOUG66611-F11|BOLD:ABY5536
Ceratopogonidae|GMPJA5092-21|BIOUG66607-E09|BOLD:ABY5536
Ceratopogonidae|GMPJA2425-21|BIOUG65681-A01|BOLD:ABY5536
Culicoides clavipalpis|GMPJA2159-21|BIOUG65678-B08|BOLD:AEH2086
Ceratopogonidae|GMPJA8074-21|BIOUG66638-H10|BOLD:AEJ7812
Culicoides oxystoma|GMPJA1944-21|BIOUG65675-H06|BOLD:AAD1856
Culicoides oxystoma|GMPJA5608-21|BIOUG66613-A03|BOLD:AAD1856
Culicoides oxystoma|GMPJA5189-21|BIOUG66608-E11|BOLD:AAD1856
Culicoides oxystoma|GMPJA4103-21|BIOUG65698-B06|BOLD:AAD1856
Culicoides oxystoma|GMPJA5125-21|BIOUG66607-H06|BOLD:AAD1856
Culicoides oxystoma|GMPJA7877-21|BIOUG66636-H03|BOLD:AAD1856
Culicoides oxystoma|GMPJA5367-21|BIOUG66610-D11|BOLD:AAD1856
Ceratopogonidae|GMPJA7962-21|BIOUG66637-G05|BOLD:ABX9968
Ceratopogonidae|GMPJA7702-21|BIOUG66635-A07|BOLD:ACR6113
Ceratopogonidae|GMPJA6807-21|BIOUG66625-F02|BOLD:AEJ1407
Ceratopogonidae|GMPJA10014-21|BIOUG66658-H05|BOLD:ADU7747
Ceratopogonidae|GMPJA8082-21|BIOUG66639-A07|BOLD:ACI9293
Ceratopogonidae|GMPJA8075-21|BIOUG66638-H11|BOLD:ACI9293
Ceratopogonidae|GMPJA8754-21|BIOUG66646-B02|BOLD:ACW1632
Ceratopogonidae|GMPJA7645-21|BIOUG66634-D09|BOLD:ACP4280
Ceratopogonidae|GMPJA7264-21|BIOUG66630-D08|BOLD:ACP4280
Ceratopogonidae|GMPJA7772-21|BIOUG66635-G05|BOLD:ACP4280
Ceratopogonidae|GMPJA7246-21|BIOUG66630-C02|BOLD:ACP4280
Ceratopogonidae|GMPJA7385-21|BIOUG66631-F10|BOLD:ACP4280
Ceratopogonidae|GMPJA7887-21|BIOUG66637-A02|BOLD:ACP4280
Ceratopogonidae|GMPJA8317-21|BIOUG66641-E04|BOLD:ACP4280
Ceratopogonidae|GMPJA8006-21|BIOUG66638-C02|BOLD:ACP4280
Ceratopogonidae|GMPJA7262-21|BIOUG66630-D06|BOLD:ACP4280
Ceratopogonidae|GMPJA8235-21|BIOUG66640-F05|BOLD:ACP4280
Ceratopogonidae|GMPJA7482-21|BIOUG66632-F12|BOLD:ACP4280
Ceratopogonidae|GMPJA7666-21|BIOUG66634-F06|BOLD:ACP4280
Ceratopogonidae|GMPJA8446-21|BIOUG66642-H02|BOLD:ACP4280
Ceratopogonidae|GMPJA8079-21|BIOUG66639-A04|BOLD:ACP4280
Ceratopogonidae|GMPJA8513-21|BIOUG66643-E10|BOLD:ACP4280
Ceratopogonidae|GMPJA7920-21|BIOUG66637-C11|BOLD:ACP4280
Ceratopogonidae|GMPJA7975-21|BIOUG66637-H06|BOLD:ACP4280
Ceratopogonidae|GMPJA8801-21|BIOUG66646-F01|BOLD:ACP4280
Ceratopogonidae|GMPJA7550-21|BIOUG66633-D09|BOLD:ACP4280
Ceratopogonidae|GMPJA8796-21|BIOUG66646-E08|BOLD:ACP4280
Ceratopogonidae|GMPJA8665-21|BIOUG66645-B08|BOLD:ACP4280
Ceratopogonidae|GMPJA8228-21|BIOUG66640-E10|BOLD:ACP4280
Ceratopogonidae|GMPJA10270-21|BIOUG70232-G12|BOLD:ACP4280
Ceratopogonidae|GMPJA8365-21|BIOUG66642-A05|BOLD:ACP4280
Ceratopogonidae|GMPJA8370-21|BIOUG66642-A10|BOLD:ACP4280

[illegible]
Ceratopogonidae|GMPJA10270-21|BIOUG70232-G12|BOLD:ACP4280
Ceratopogonidae|GMPJA8365-21|BIOUG66642-A05|BOLD:ACP4280
Ceratopogonidae|GMPJA8370-21|BIOUG66642-A10|BOLD:ACP4280
Ceratopogonidae|GMPJA10295-21|BIOUG70233-B02|BOLD:ACP4280
Ceratopogonidae|GMPJA8015-21|BIOUG66638-C11|BOLD:ACP4280
Ceratopogonidae|GMPJA7423-21|BIOUG66632-B01|BOLD:ACP4280
Ceratopogonidae|GMPJA8221-21|BIOUG66640-E03|BOLD:ACP4280
Ceratopogonidae|GMPJA8009-21|BIOUG66638-C05|BOLD:ACP4280
Ceratopogonidae|GMPJA8339-21|BIOUG66641-G02|BOLD:ACP4280
Ceratopogonidae|GMPJA7777-21|BIOUG66635-G10|BOLD:ACP4280
Ceratopogonidae|GMPJA8166-21|BIOUG66639-H07|BOLD:ACP4280
Ceratopogonidae|GMPJA8447-21|BIOUG66642-H03|BOLD:ACP4280
Ceratopogonidae|GMPJA7745-21|BIOUG66635-E02|BOLD:ACP4280
Ceratopogonidae|GMPJA7689-21|BIOUG66634-H05|BOLD:ACP4280
Ceratopogonidae|GMPJA8715-21|BIOUG66645-F10|BOLD:ACP4280
Ceratopogonidae|GMPJA7346-21|BIOUG66631-C07|BOLD:ACP4280
Ceratopogonidae|GMPJA7614-21|BIOUG66634-B02|BOLD:ACP4280
Ceratopogonidae|GMPJA7404-21|BIOUG66631-H05|BOLD:ACP4280
Ceratopogonidae|GMPJA8603-21|BIOUG66644-E05|BOLD:ACP4280
Ceratopogonidae|GMPJA7538-21|BIOUG66633-C09|BOLD:ACP4280
Ceratopogonidae|GMPJA8328-21|BIOUG66641-F03|BOLD:ACP4280
Ceratopogonidae|GMPJA8515-21|BIOUG66643-E12|BOLD:ACP4280
Ceratopogonidae|GMPJA8241-21|BIOUG66640-F11|BOLD:ACP4280
Ceratopogonidae|GMPJA8578-21|BIOUG66644-C04|BOLD:ACP4280
Ceratopogonidae|GMPJA8102-21|BIOUG66639-C03|BOLD:ACP4280
Ceratopogonidae|GMPJA7872-21|BIOUG66636-G10|BOLD:ACP4280
Ceratopogonidae|GMPJA8509-21|BIOUG66643-E06|BOLD:ACP4280
Ceratopogonidae|GMPJA8567-21|BIOUG66644-B05|BOLD:ACP4280
Ceratopogonidae|GMPJA8774-21|BIOUG66646-C10|BOLD:ACP4280
Ceratopogonidae|GMPJA7769-21|BIOUG66635-G02|BOLD:ACP4280
Ceratopogonidae|GMPJA7635-21|BIOUG66634-C11|BOLD:ACP4280
Ceratopogonidae|GMPJA8652-21|BIOUG66645-A07|BOLD:ACP4280
Ceratopogonidae|GMPJA7273-21|BIOUG66630-E05|BOLD:ACP4280
Ceratopogonidae|GMPJA8481-21|BIOUG66643-C02|BOLD:ACP4280
Ceratopogonidae|GMPJA8449-21|BIOUG66642-H05|BOLD:ACP4280
Ceratopogonidae|GMPJA8262-21|BIOUG66640-H08|BOLD:ACP4280
Ceratopogonidae|GMPJA8187-21|BIOUG66640-B05|BOLD:ACP4280
Ceratopogonidae|GMPJA7588-21|BIOUG66633-G11|BOLD:ACP4280
Ceratopogonidae|GMPJA7255-21|BIOUG66630-C11|BOLD:ACP4280
Ceratopogonidae|GMPJA7503-21|BIOUG66632-H09|BOLD:ACP4280
Ceratopogonidae|GMPJA7392-21|BIOUG66631-G05|BOLD:ACP4280
Ceratopogonidae|GMPJA8395-21|BIOUG66642-C11|BOLD:ACP4280
Ceratopogonidae|GMPJA10283-21|BIOUG70233-A02|BOLD:ACP4280
Ceratopogonidae|GMPJA7846-21|BIOUG66636-E08|BOLD:ACP4280
Ceratopogonidae|GMPJA8316-21|BIOUG66641-E03|BOLD:ACP4280
Ceratopogonidae|GMPJA7751-21|BIOUG66635-E08|BOLD:ACP4280
Ceratopogonidae|GMPJA7753-21|BIOUG66635-E10|BOLD:ACP4280
Ceratopogonidae|GMPJA8433-21|BIOUG66642-G01|BOLD:ACP4280
Ceratopogonidae|GMPJA8104-21|BIOUG66639-C05|BOLD:ACP4280
Ceratopogonidae|GMPJA8334-21|BIOUG66641-F09|BOLD:ACP4280
Ceratopogonidae|GMPJA7834-21|BIOUG66636-D08|BOLD:ACP4280
Ceratopogonidae|GMPJA7774-21|BIOUG66635-G07|BOLD:ACP4280
Ceratopogonidae|GMPJA7881-21|BIOUG66636-H07|BOLD:ACP4280
Ceratopogonidae|GMPJA8708-21|BIOUG66645-F03|BOLD:ACP4280
Ceratopogonidae|GMPJA7524-21|BIOUG66633-B07|BOLD:ACP4280
Ceratopogonidae|GMPJA8784-21|BIOUG66646-D08|BOLD:ACP4280
Ceratopogonidae|GMPJA8517-21|BIOUG66643-F02|BOLD:ACP4280
Ceratopogonidae|GMPJA8283-21|BIOUG66641-B06|BOLD:ACP4280
Ceratopogonidae|GMPJA8758-21|BIOUG66646-B06|BOLD:ACP4280
Ceratopogonidae|GMPJA8722-21|BIOUG66645-G05|BOLD:ACP4280
Ceratopogonidae|GMPJA8601-21|BIOUG66644-E03|BOLD:ACP4280
Ceratopogonidae|GMPJA8267-21|BIOUG66641-A02|BOLD:ACP4280
Ceratopogonidae|GMPJA7527-21|BIOUG66633-B10|BOLD:ACP4280
Ceratopogonidae|GMPJA7880-21|BIOUG66636-H06|BOLD:ACP4280
Ceratopogonidae|GMPJA7555-21|BIOUG66633-E02|BOLD:ACP4280
Ceratopogonidae|GMPJA8804-21|BIOUG66646-F04|BOLD:ACP4280
Ceratopogonidae|GMPJA7697-21|BIOUG66635-A02|BOLD:ACP4280
Ceratopogonidae|GMPJA7613-21|BIOUG66634-B01|BOLD:ACP4280
Ceratopogonidae|GMPJA8378-21|BIOUG66642-B06|BOLD:ACP4280
Ceratopogonidae|GMPJA8668-21|BIOUG66645-B11|BOLD:ACP4280
Ceratopogonidae|GMPJA8394-21|BIOUG66642-C10|BOLD:ACP4280
Ceratopogonidae|GMPJA7632-21|BIOUG66634-C08|BOLD:ACP4280
Ceratopogonidae|GMPJA7983-21|BIOUG66638-A03|BOLD:ACP4280
Ceratopogonidae|GMPJA7691-21|BIOUG66634-H07|BOLD:ACP4280
Ceratopogonidae|GMPJA7752-21|BIOUG66635-E09|BOLD:ACP4280
Ceratopogonidae|GMPJA7453-21|BIOUG66632-D07|BOLD:ACP4280
Ceratopogonidae|GMPJA8593-21|BIOUG66644-D07|BOLD:ACP4280
Ceratopogonidae|GMPJA7619-21|BIOUG66634-B07|BOLD:ACP4280
Ceratopogonidae|GMPJA7424-21|BIOUG66632-B02|BOLD:ACP4280
Ceratopogonidae|GMPJA8570-21|BIOUG66644-B08|BOLD:ACP4280
Ceratopogonidae|GMPJA7476-21|BIOUG66632-F06|BOLD:ACP4280
Ceratopogonidae|GMPJA8389-21|BIOUG66642-C05|BOLD:ACP4280
Ceratopogonidae|GMPJA7609-21|BIOUG66634-A09|BOLD:ACP4280
Ceratopogonidae|GMPJA7445-21|BIOUG66632-C11|BOLD:ACP4280
Ceratopogonidae|GMPJA7963-21|BIOUG66637-G06|BOLD:ACP4280
Ceratopogonidae|GMPJA8052-21|BIOUG66638-F12|BOLD:ACP4280
Ceratopogonidae|GMPJA8145-21|BIOUG66639-F10|BOLD:ACP4280
Ceratopogonidae|GMPJA7345-21|BIOUG66631-C06|BOLD:ACP4280
Ceratopogonidae|GMPJA7875-21|BIOUG66636-H01|BOLD:ACP4280
Ceratopogonidae|GMPJA8781-21|BIOUG66646-D05|BOLD:ACP4280
Ceratopogonidae|GMPJA7949-21|BIOUG66637-F04|BOLD:ACP4280
Ceratopogonidae|GMPJA7603-21|BIOUG66634-A03|BOLD:ACP4280
Ceratopogonidae|GMPJA7341-21|BIOUG66631-C02|BOLD:ACP4280
Ceratopogonidae|GMPJA7942-21|BIOUG66637-E09|BOLD:ACP4280
Ceratopogonidae|GMPJA7927-21|BIOUG66637-D06|BOLD:ACP4280
Ceratopogonidae|GMPJA7664-21|BIOUG66634-F04|BOLD:ACP4280
Ceratopogonidae|GMPJA7900-21|BIOUG66637-B03|BOLD:ACP4280
Ceratopogonidae|GMPJA7338-21|BIOUG66631-B11|BOLD:ACP4280
Ceratopogonidae|GMPJA8661-21|BIOUG66645-B04|BOLD:ACP4280
Ceratopogonidae|GMPJA7633-21|BIOUG66634-C09|BOLD:ACP4280
Ceratopogonidae|GMPJA8573-21|BIOUG66644-B11|BOLD:ACP4280
Ceratopogonidae|GMPJA8799-21|BIOUG66646-E11|BOLD:ACP4280
Ceratopogonidae|GMPJA7597-21|BIOUG66633-H08|BOLD:ACP4280

Ceratopogonidae|GMPJA[illegible]-21|BIOUG[illegible]|BOLD:ACP4280
Ceratopogonidae|GMPJA8573-21|BIOUG66644-B11|BOLD:ACP4280
Ceratopogonidae|GMPJA8799-21|BIOUG66646-E11|BOLD:ACP4280
Ceratopogonidae|GMPJA7597-21|BIOUG66633-H08|BOLD:ACP4280
Ceratopogonidae|GMPJA7311-21|BIOUG66630-H07|BOLD:ACP4280
Ceratopogonidae|GMPJA8383-21|BIOUG66642-B11|BOLD:ACP4280
Ceratopogonidae|GMPJA8078-21|BIOUG66639-A03|BOLD:ACP4280
Ceratopogonidae|GMPJA7643-21|BIOUG66634-D07|BOLD:ACP4280
Ceratopogonidae|GMPJA8202-21|BIOUG66640-C08|BOLD:ACP4280
Ceratopogonidae|GMPJA8276-21|BIOUG66641-A11|BOLD:ACP4280
Ceratopogonidae|GMPJA7432-21|BIOUG66632-B10|BOLD:ACP4280
Ceratopogonidae|GMPJA7675-21|BIOUG66634-G03|BOLD:ACP4280
Ceratopogonidae|GMPJA8494-21|BIOUG66643-D03|BOLD:ACP4280
Ceratopogonidae|GMPJA8192-21|BIOUG66640-B10|BOLD:ACP4280
Ceratopogonidae|GMPJA8109-21|BIOUG66639-C10|BOLD:ACP4280
Ceratopogonidae|GMPJA8032-21|BIOUG66638-E04|BOLD:ACP4280
Ceratopogonidae|GMPJA8162-21|BIOUG66639-H03|BOLD:ACP4280
Ceratopogonidae|GMPJA7374-21|BIOUG66631-E11|BOLD:ACP4280
Ceratopogonidae|GMPJA8469-21|BIOUG66643-B02|BOLD:ACP4280
Ceratopogonidae|GMPJA7533-21|BIOUG66633-C04|BOLD:ACP4280
Ceratopogonidae|GMPJA7319-21|BIOUG66631-A04|BOLD:ACP4280
Ceratopogonidae|GMPJA8710-21|BIOUG66645-F05|BOLD:ACP4280
Ceratopogonidae|GMPJA8336-21|BIOUG66641-F11|BOLD:ACP4280
Ceratopogonidae|GMPJA8288-21|BIOUG66641-B11|BOLD:ACP4280
Ceratopogonidae|GMPJA8285-21|BIOUG66641-B08|BOLD:ACP4280
Ceratopogonidae|GMPJA7417-21|BIOUG66632-A07|BOLD:ACP4280
Ceratopogonidae|GMPJA7926-21|BIOUG66637-D05|BOLD:ACP4280
Ceratopogonidae|GMPJA8459-21|BIOUG66643-A04|BOLD:ACP4280
Ceratopogonidae|GMPJA7793-21|BIOUG66636-A03|BOLD:ACP4280
Ceratopogonidae|GMPJA7626-21|BIOUG66634-C02|BOLD:ACP4280
Ceratopogonidae|GMPJA7367-21|BIOUG66631-E04|BOLD:ACP4280
Forcipomyia|GMPJA8299-21|BIOUG66641-C10|BOLD:ABX4360
Forcipomyia|GMPJA512-21|BIOUG65660-G11|BOLD:ABX4360
Forcipomyia|GMPJA341-21|BIOUG65659-A07|BOLD:ABX4360
Forcipomyia|GMPJA8484-21|BIOUG66643-C05|BOLD:ABX4360
Forcipomyia|GMPJA8263-21|BIOUG66640-H09|BOLD:ABX4360
Forcipomyia|GMPJA7580-21|BIOUG66633-G03|BOLD:ABX4360
Forcipomyia|GMPJA066-21|BIOUG65656-B05|BOLD:ABX4360
Forcipomyia|GMPJA207-21|BIOUG65657-F03|BOLD:ABX4360
Forcipomyia|GMPJA463-21|BIOUG65660-C10|BOLD:ABX4360
Forcipomyia|GMPJA1564-21|BIOUG65671-H06|BOLD:ABX4360
Forcipomyia|GMPJA095-21|BIOUG65656-D10|BOLD:ABX4360
Forcipomyia|GMPJA1559-21|BIOUG65671-H01|BOLD:ABX4360
Forcipomyia|GMPJA981-21|BIOUG65665-G05|BOLD:ABX4360
Forcipomyia|GMPJA504-21|BIOUG65660-G03|BOLD:ABX4360
Forcipomyia|GMPJA448-21|BIOUG65660-B07|BOLD:ABX4360
Forcipomyia|GMPJA7780-21|BIOUG66635-H01|BOLD:ABX4360
Forcipomyia|GMPJA8282-21|BIOUG66641-B05|BOLD:ABX4360
Forcipomyia|GMPJA8518-21|BIOUG66643-F03|BOLD:ABX4360
Forcipomyia|GMPJA8143-21|BIOUG66639-F08|BOLD:ABX4360
Forcipomyia|GMPJA7838-21|BIOUG66636-D12|BOLD:ABX4360
Forcipomyia|GMPJA8351-21|BIOUG66641-H02|BOLD:ABX4360
Forcipomyia|GMPJA10614-21|BIOUG70236-D12|BOLD:ABX4360
Forcipomyia|GMPJA8770-21|BIOUG66646-C06|BOLD:ABX4360
Forcipomyia|GMPJA417-21|BIOUG65659-G11|BOLD:ABX4360
Forcipomyia|GMPJA397-21|BIOUG65659-F03|BOLD:ABX4360
Forcipomyia|GMPJA051-21|BIOUG65656-A02|BOLD:ABX4360
Forcipomyia|GMPJA445-21|BIOUG65660-B04|BOLD:ABX4360
Forcipomyia|GMPJA966-21|BIOUG65665-F02|BOLD:ABX4360
Forcipomyia|GMPJA390-21|BIOUG65659-E08|BOLD:ABX4360
Forcipomyia|GMPJA646-21|BIOUG65662-C03|BOLD:ABX4360
Forcipomyia|GMPJA652-21|BIOUG65662-C09|BOLD:ABX4360
Forcipomyia|GMPJA993-21|BIOUG65665-H05|BOLD:ABX4360
Forcipomyia|GMPJA1056-21|BIOUG65666-E09|BOLD:ABX4360
Forcipomyia|GMPJA1073-21|BIOUG65666-G02|BOLD:ABX4360
Forcipomyia|GMPJA1406-21|BIOUG65670-C03|BOLD:ABX4360
Forcipomyia|GMPJA1606-21|BIOUG65672-D01|BOLD:ABX4360
Forcipomyia|GMPJA1697-21|BIOUG65673-C09|BOLD:ABX4360
Forcipomyia|GMPJA2060-21|BIOUG65677-B04|BOLD:ABX4360
Forcipomyia|GMPJA718-21|BIOUG65663-A04|BOLD:ABX4360
Forcipomyia|GMPJA2205-21|BIOUG65678-F06|BOLD:ABX4360
Forcipomyia|GMPJA2173-21|BIOUG65678-C10|BOLD:ABX4360
Forcipomyia|GMPJA2371-21|BIOUG65680-D06|BOLD:ABX4360
Forcipomyia|GMPJA212-21|BIOUG65657-F08|BOLD:ABX4360
Forcipomyia|GMPJA3926-21|BIOUG65696-C07|BOLD:ABX4360
Forcipomyia|GMPJA8322-21|BIOUG66641-E09|BOLD:ABX4360
Forcipomyia|GMPJA7670-21|BIOUG66634-F10|BOLD:ABX4360
Forcipomyia|GMPJA7480-21|BIOUG66632-F10|BOLD:ABX4360
Forcipomyia|GMPJA7688-21|BIOUG66634-H04|BOLD:ABX4360
Forcipomyia|GMPJA7406-21|BIOUG66631-H07|BOLD:ABX4360
Ceratopogonidae_incertae_sedis|GMPJA10287-21|BIOUG70233-A06|BOLD:AFI7763
Ceratopogonidae|GMPJA8388-21|BIOUG66642-C04|BOLD:ACG0244
Ceratopogonidae|GMPJA7552-21|BIOUG66633-D11|BOLD:ACG0244
Ceratopogonidae|GMPJA7771-21|BIOUG66635-G04|BOLD:ACG0244
Ceratopogonidae|GMPJA10333-21|BIOUG70233-E04|BOLD:ACG0244
Ceratopogonidae|GMPJA7606-21|BIOUG66634-A06|BOLD:ACG0244
Ceratopogonidae|GMPJA8324-21|BIOUG66641-E11|BOLD:ACG0244
Ceratopogonidae|GMPJA8695-21|BIOUG66645-E02|BOLD:ACG0244
Ceratopogonidae|GMPJA10365-21|BIOUG70233-G12|BOLD:ACG0244
Ceratopogonidae|GMPJA8719-21|BIOUG66645-G02|BOLD:ACG0244
Ceratopogonidae|GMPJA8673-21|BIOUG66645-C04|BOLD:ACG0244
Ceratopogonidae|GMPJA8581-21|BIOUG66644-C07|BOLD:ACG0244
Ceratopogonidae|GMPJA8260-21|BIOUG66640-H06|BOLD:ACG0244
Ceratopogonidae|GMPJA10358-21|BIOUG70233-G05|BOLD:ACG0244
Ceratopogonidae|GMPJA7481-21|BIOUG66632-F11|BOLD:ACG0244
Ceratopogonidae|GMPJA7484-21|BIOUG66632-G02|BOLD:ACG0244
Ceratopogonidae|GMPJA8729-21|BIOUG66645-G12|BOLD:ACG0244
Ceratopogonidae|GMPJA077-21|BIOUG65656-C04|BOLD:ACG0244
Ceratopogonidae|GMPJA452-21|BIOUG65660-B11|BOLD:ACG0244
Ceratopogonidae|GMPJA8756-21|BIOUG66646-B04|BOLD:ACG0244
Ceratopogonidae|GMPJA7542-21|BIOUG66633-D01|BOLD:ACG0244
Ceratopogonidae|GMPJA7993-21|BIOUG66638-B01|BOLD:ACG0244
Ceratopogonidae|GMPJA7693-21|BIOUG66634-H09|BOLD:ACG0244
[illegible]|GMPJA8551-21|BIOUG66644-A01|BOLD:ACG0244

Ceratopogonidae|GMPJA7[illegible]42-21|BIOUG6663[illegible]-D01|BOLD:ACG0244
Ceratopogonidae|GMPJA7993-21|BIOUG66638-B01|BOLD:ACG0244
Ceratopogonidae|GMPJA7693-21|BIOUG66634-H09|BOLD:ACG0244
Ceratopogonidae|GMPJA8551-21|BIOUG66644-A01|BOLD:ACG0244
Ceratopogonidae|GMPJA10331-21|BIOUG70233-E02|BOLD:ACG0244
Ceratopogonidae|GMPJA7994-21|BIOUG66638-B02|BOLD:ACG0244
Ceratopogonidae|GMPJA131-21|BIOUG65656-G10|BOLD:ACG0244
Ceratopogonidae|GMPJA8585-21|BIOUG66644-C11|BOLD:ACG0244
Ceratopogonidae|GMPJA7987-21|BIOUG66638-A07|BOLD:ACG0244
Ceratopogonidae|GMPJA7705-21|BIOUG66635-A10|BOLD:ACG0244
Ceratopogonidae|GMPJA338-21|BIOUG65659-A04|BOLD:ACG0244
Ceratopogonidae|GMPJA6539-21|BIOUG66622-G07|BOLD:ACG0244
Ceratopogonidae|GMPJA1639-21|BIOUG65672-F10|BOLD:ACU8246
Ceratopogonidae|GMPJA399-21|BIOUG65659-F05|BOLD:ACU8246
Ceratopogonidae|GMPJA421-21|BIOUG65659-H03|BOLD:ACU8246
Ceratopogonidae|GMPJA428-21|BIOUG65659-H10|BOLD:ACU8246
Ceratopogonidae|GMPJA451-21|BIOUG65660-B10|BOLD:ACU8246
Ceratopogonidae|GMPJA468-21|BIOUG65660-D03|BOLD:ACU8246
Ceratopogonidae|GMPJA533-21|BIOUG65661-A09|BOLD:ACU8246
Ceratopogonidae|GMPJA151-21|BIOUG65657-A07|BOLD:ACU8246
Ceratopogonidae|GMPJA171-21|BIOUG65657-C03|BOLD:ACU8246
Ceratopogonidae|GMPJA176-21|BIOUG65657-C08|BOLD:ACU8246
Ceratopogonidae|GMPJA183-21|BIOUG65657-D03|BOLD:ACU8246
Ceratopogonidae|GMPJA194-21|BIOUG65657-E02|BOLD:ACU8246
Ceratopogonidae|GMPJA1835-21|BIOUG65674-G04|BOLD:ACU8246
Ceratopogonidae|GMPJA1425-21|BIOUG65670-D10|BOLD:ACU8246
Ceratopogonidae|GMPJA1432-21|BIOUG65670-E05|BOLD:ACU8246
Ceratopogonidae|GMPJA2236-21|BIOUG65679-A02|BOLD:ACU8246
Ceratopogonidae|GMPJA2406-21|BIOUG65680-G05|BOLD:ACU8246
Ceratopogonidae|GMPJA128-21|BIOUG65656-G07|BOLD:ACU8246
Ceratopogonidae|GMPJA3412-21|BIOUG65690-H03|BOLD:ACU8246
Ceratopogonidae|GMPJA10632-21|BIOUG70236-F06|BOLD:ACU8246
Ceratopogonidae|GMPJA10198-21|BIOUG70232-A12|BOLD:ACU8246
Ceratopogonidae|GMPJA2493-21|BIOUG65681-F09|BOLD:ACU8246
Ceratopogonidae|GMPJA2459-21|BIOUG65681-C11|BOLD:ACU8246
Ceratopogonidae|GMPJA4428-21|BIOUG66601-E10|BOLD:AEJ5998
Ceratopogonidae|GMPJA2301-21|BIOUG65679-F07|BOLD:AEJ3822
Ceratopogonidae|GMPJA2368-21|BIOUG65680-D03|BOLD:AEJ3822
Ceratopogonidae|GMPJA1755-21|BIOUG65673-H07|BOLD:AEJ3822
Ceratopogonidae|GMPJA1316-21|BIOUG65669-C08|BOLD:AEJ3822
Ceratopogonidae|GMPJA3322-21|BIOUG65689-H08|BOLD:AEJ3822
Ceratopogonidae|GMPJA2288-21|BIOUG65679-E06|BOLD:ACP4190
Ceratopogonidae|GMPJA3330-21|BIOUG65690-A05|BOLD:ACP4190
Ceratopogonidae|GMPJA3289-21|BIOUG65689-E11|BOLD:ACP4190
Ceratopogonidae|GMPJA2396-21|BIOUG65680-F07|BOLD:ACP4190
Ceratopogonidae|GMPJA3523-21|BIOUG65692-A08|BOLD:ACP4190
Ceratopogonidae|GMPJA8507-21|BIOUG66643-E04|BOLD:ACP4190
Ceratopogonidae|GMPJA1462-21|BIOUG65670-G11|BOLD:ACP4190
Ceratopogonidae|GMPJA2481-21|BIOUG65681-E09|BOLD:ACP4190
Ceratopogonidae|GMPJA3398-21|BIOUG65690-G01|BOLD:ABY0384
Ceratopogonidae|GMPJA6264-21|BIOUG66619-H05|BOLD:AEJ1754
Ceratopogonidae|GMPJA1446-21|BIOUG65670-F07|BOLD:ABY0384
Ceratopogonidae|GMPJA3021-21|BIOUG65686-G04|BOLD:ABY0384
Ceratopogonidae|GMPJA1539-21|BIOUG65671-F05|BOLD:ABY0384
Ceratopogonidae|GMPJA2430-21|BIOUG65681-A06|BOLD:ABY0384
Ceratopogonidae|GMPJA1573-21|BIOUG65672-A04|BOLD:ABY0384
Ceratopogonidae|GMPJA4059-21|BIOUG65697-F09|BOLD:ABY0384
Ceratopogonidae|GMPJA702-21|BIOUG65662-G11|BOLD:ABY0384
Ceratopogonidae|GMPJA3042-21|BIOUG65687-A02|BOLD:ABY0384
Ceratopogonidae|GMPJA1780-21|BIOUG65674-B09|BOLD:ABY0384
Ceratopogonidae|GMPJA2334-21|BIOUG65680-A05|BOLD:ABY0384
Ceratopogonidae|GMPJA3364-21|BIOUG65690-D03|BOLD:ABY0384
Ceratopogonidae|GMPJA3388-21|BIOUG65690-F03|BOLD:ABY0384
Ceratopogonidae|GMPJA3549-21|BIOUG65692-C10|BOLD:ABY0384
Ceratopogonidae|GMPJA3206-21|BIOUG65688-F11|BOLD:ABY0384
Ceratopogonidae|GMPJA5434-21|BIOUG66611-B07|BOLD:ABY0384
Ceratopogonidae|GMPJA3193-21|BIOUG65688-E10|BOLD:ABY0384
Ceratopogonidae|GMPJA1657-21|BIOUG65672-H04|BOLD:ABY0384
Ceratopogonidae|GMPJA3203-21|BIOUG65688-F08|BOLD:ABY0384
Ceratopogonidae|GMPJA1710-21|BIOUG65673-D10|BOLD:ABY0384
Ceratopogonidae|GMPJA3591-21|BIOUG65692-G04|BOLD:ABY0384
Ceratopogonidae|GMPJA4002-21|BIOUG65697-A12|BOLD:ABY0384
Ceratopogonidae|GMPJA7598-21|BIOUG66633-H09|BOLD:ABY0384
Ceratopogonidae|GMPJA1775-21|BIOUG65674-B04|BOLD:ABY0384
Ceratopogonidae|GMPJA3621-21|BIOUG65693-A11|BOLD:ABY0384
Ceratopogonidae|GMPJA1785-21|BIOUG65674-C02|BOLD:ABY0384
Ceratopogonidae|GMPJA1703-21|BIOUG65673-D03|BOLD:ABY0384
Ceratopogonidae|GMPJA2179-21|BIOUG65678-D04|BOLD:ABY0384
Ceratopogonidae|GMPJA4201-21|BIOUG65699-B09|BOLD:ABY0384
Ceratopogonidae|GMPJA8197-21|BIOUG66640-C03|BOLD:ABY0384
Ceratopogonidae|GMPJA8011-21|BIOUG66638-C07|BOLD:ABY0384
Ceratopogonidae|GMPJA2395-21|BIOUG65680-F06|BOLD:ABY0384
Ceratopogonidae|GMPJA1772-21|BIOUG65674-B01|BOLD:ABY0384
Ceratopogonidae|GMPJA5256-21|BIOUG66609-C07|BOLD:ABY0384
Ceratopogonidae|GMPJA3425-21|BIOUG65691-A05|BOLD:ABY0384
Ceratopogonidae|GMPJA3485-21|BIOUG65691-F05|BOLD:ABY0384
Ceratopogonidae|GMPJA2025-21|BIOUG65676-G04|BOLD:ABY0384
Ceratopogonidae|GMPJA1635-21|BIOUG65672-F06|BOLD:ABY0384
Ceratopogonidae|GMPJA5524-21|BIOUG66612-B02|BOLD:ABY0384
Ceratopogonidae|GMPJA3561-21|BIOUG65692-D10|BOLD:ABY0384
Ceratopogonidae|GMPJA3636-21|BIOUG65693-C02|BOLD:ABY0384
Ceratopogonidae|GMPJA2463-21|BIOUG65681-D03|BOLD:ABY0384
Ceratopogonidae|GMPJA2415-21|BIOUG65680-H02|BOLD:ABY0384
Ceratopogonidae|GMPJA2253-21|BIOUG65679-B07|BOLD:ABY0384
Ceratopogonidae|GMPJA3644-21|BIOUG65693-C10|BOLD:ABY0384
Ceratopogonidae|GMPJA3228-21|BIOUG65688-H09|BOLD:ABY0384
Ceratopogonidae|GMPJA8767-21|BIOUG66646-C03|BOLD:ABY0384
Ceratopogonidae|GMPJA8580-21|BIOUG66644-C06|BOLD:ABY0384
Ceratopogonidae|GMPJA1335-21|BIOUG65669-E03|BOLD:ABY0384
Ceratopogonidae|GMPJA7819-21|BIOUG66636-C05|BOLD:ABY0384
Ceratopogonidae|GMPJA2023-21|BIOUG65676-G02|BOLD:ABY0384
Ceratopogonidae|GMPJA7807-21|BIOUG66636-B05|BOLD:ABY0384
Ceratopogonidae|GMPJA1693-21|BIOUG65673-C05|BOLD:ABY0384
[illegible]

Ceratopogonidae|GMPJA2023-21|BIOUG65676-G02|BOLD:ABY0384
Ceratopogonidae|GMPJA7807-21|BIOUG66636-B05|BOLD:ABY0384
Ceratopogonidae|GMPJA1693-21|BIOUG65673-C05|BOLD:ABY0384
Ceratopogonidae|GMPJA2362-21|BIOUG65680-C09|BOLD:ABY0384
Ceratopogonidae|GMPJA1626-21|BIOUG65672-E09|BOLD:ABY0384
Ceratopogonidae|GMPJA2267-21|BIOUG65679-C09|BOLD:ABY0384
Ceratopogonidae|GMPJA3307-21|BIOUG65689-G05|BOLD:ABY0384
Ceratopogonidae|GMPJA1815-21|BIOUG65674-E08|BOLD:ABY0384
Ceratopogonidae|GMPJA1512-21|BIOUG65671-D02|BOLD:ABY0384
Ceratopogonidae|GMPJA3391-21|BIOUG65690-F06|BOLD:ABY0384
Ceratopogonidae|GMPJA3387-21|BIOUG65690-F02|BOLD:ABY0384
Ceratopogonidae|GMPJA2221-21|BIOUG65678-G10|BOLD:ABY0384
Ceratopogonidae|GMPJA1847-21|BIOUG65674-H04|BOLD:ABY0384
Ceratopogonidae|GMPJA1740-21|BIOUG65673-G04|BOLD:ABY0384
Ceratopogonidae|GMPJA3185-21|BIOUG65688-E02|BOLD:ABY0384
Ceratopogonidae|GMPJA3086-21|BIOUG65687-D10|BOLD:ABY0384
Ceratopogonidae|GMPJA2319-21|BIOUG65679-H01|BOLD:ABY0384
Ceratopogonidae|GMPJA1651-21|BIOUG65672-G10|BOLD:ABY0384
Ceratopogonidae|GMPJA4319-21|BIOUG65700-D08|BOLD:ABY0384
Ceratopogonidae|GMPJA3176-21|BIOUG65688-D05|BOLD:ABY0384
Ceratopogonidae|GMPJA3156-21|BIOUG65688-B09|BOLD:ABY0384
Ceratopogonidae|GMPJA1884-21|BIOUG65675-C06|BOLD:ABY0384
Ceratopogonidae|GMPJA1138-21|BIOUG65667-D08|BOLD:ABY0384
Ceratopogonidae|GMPJA2138-21|BIOUG65677-H10|BOLD:ABY0384
Ceratopogonidae|GMPJA8815-21|BIOUG66646-G03|BOLD:ABY0384
Ceratopogonidae|GMPJA3593-21|BIOUG65692-G06|BOLD:ABY0384
Ceratopogonidae|GMPJA8057-21|BIOUG66638-G05|BOLD:ABY0384
Ceratopogonidae|GMPJA1306-21|BIOUG65669-B10|BOLD:ABY0384
Ceratopogonidae|GMPJA3257-21|BIOUG65689-C03|BOLD:ABY0384
Ceratopogonidae|GMPJA1690-21|BIOUG65673-C02|BOLD:ABY0384
Ceratopogonidae|GMPJA1523-21|BIOUG65671-E01|BOLD:ABY0384
Ceratopogonidae|GMPJA3541-21|BIOUG65692-C02|BOLD:ABY0384
Ceratopogonidae|GMPJA1958-21|BIOUG65676-A09|BOLD:ABY0384
Ceratopogonidae|GMPJA1793-21|BIOUG65674-C10|BOLD:ABY0384
Ceratopogonidae|GMPJA1501-21|BIOUG65671-C03|BOLD:ABY0384
Ceratopogonidae|GMPJA3329-21|BIOUG65690-A04|BOLD:ABY0384
Ceratopogonidae|GMPJA2268-21|BIOUG65679-C10|BOLD:ABY0384
Ceratopogonidae|GMPJA7369-21|BIOUG66631-E06|BOLD:ABY0384
Ceratopogonidae|GMPJA3301-21|BIOUG65689-F11|BOLD:ABY0384
Ceratopogonidae|GMPJA3212-21|BIOUG65688-G05|BOLD:ABY0384
Ceratopogonidae|GMPJA2059-21|BIOUG65677-B03|BOLD:ABY0384
Ceratopogonidae|GMPJA1378-21|BIOUG65669-H10|BOLD:ABY0384
Ceratopogonidae|GMPJA7781-21|BIOUG66635-H02|BOLD:ABY0384
Ceratopogonidae|GMPJA2379-21|BIOUG65680-E02|BOLD:ABY0384
Ceratopogonidae|GMPJA2344-21|BIOUG65680-B03|BOLD:ABY0384
Ceratopogonidae|GMPJA3259-21|BIOUG65689-C05|BOLD:ABY0384
Ceratopogonidae|GMPJA1542-21|BIOUG65671-F08|BOLD:ABY0384
Ceratopogonidae|GMPJA2286-21|BIOUG65679-E04|BOLD:ABY0384
Ceratopogonidae|GMPJA2370-21|BIOUG65680-D05|BOLD:ABY0384
Ceratopogonidae|GMPJA2323-21|BIOUG65679-H05|BOLD:ABY0384
Ceratopogonidae|GMPJA2143-21|BIOUG65678-A04|BOLD:ABY0384
Ceratopogonidae|GMPJA3344-21|BIOUG65690-B07|BOLD:ABY0384
Ceratopogonidae|GMPJA1506-21|BIOUG65671-C08|BOLD:ABY0384
Ceratopogonidae|GMPJA3192-21|BIOUG65688-E09|BOLD:ABY0384
Ceratopogonidae|GMPJA3620-21|BIOUG65693-A10|BOLD:ABY0384
Ceratopogonidae|GMPJA2465-21|BIOUG65681-D05|BOLD:ABY0384
Ceratopogonidae|GMPJA1770-21|BIOUG65674-A11|BOLD:ABY0384
Ceratopogonidae|GMPJA1625-21|BIOUG65672-E08|BOLD:ABY0384
Ceratopogonidae|GMPJA1924-21|BIOUG65675-F10|BOLD:ABY0384
Ceratopogonidae|GMPJA1769-21|BIOUG65674-A10|BOLD:ABY0384
Ceratopogonidae|GMPJA3324-21|BIOUG65689-H10|BOLD:ABY0384
Ceratopogonidae|GMPJA2303-21|BIOUG65679-F09|BOLD:ABY0384
Ceratopogonidae|GMPJA2226-21|BIOUG65678-H03|BOLD:ABY0384
Ceratopogonidae|GMPJA1457-21|BIOUG65670-G06|BOLD:ABY0384
Ceratopogonidae|GMPJA3544-21|BIOUG65692-C05|BOLD:ABY0384
Ceratopogonidae|GMPJA2403-21|BIOUG65680-G02|BOLD:ABY0384
Ceratopogonidae|GMPJA2393-21|BIOUG65680-F04|BOLD:ABY0384
Ceratopogonidae|GMPJA3144-21|BIOUG65688-A09|BOLD:ABY0384
Ceratopogonidae|GMPJA2197-21|BIOUG65678-E10|BOLD:ABY0384
Ceratopogonidae|GMPJA3150-21|BIOUG65688-B03|BOLD:ABY0384
Ceratopogonidae|GMPJA2291-21|BIOUG65679-E09|BOLD:ABY0384
Ceratopogonidae|GMPJA2219-21|BIOUG65678-G08|BOLD:ABY0384
Ceratopogonidae|GMPJA3598-21|BIOUG65692-G11|BOLD:ABY0384
Ceratopogonidae|GMPJA3210-21|BIOUG65688-G03|BOLD:ABY0384
Ceratopogonidae|GMPJA3530-21|BIOUG65692-B03|BOLD:ABY0384
Ceratopogonidae|GMPJA2377-21|BIOUG65680-D12|BOLD:ABY0384
Ceratopogonidae|GMPJA1313-21|BIOUG65669-C05|BOLD:ABY0384
Ceratopogonidae|GMPJA3921-21|BIOUG65696-C02|BOLD:ABY0384
Ceratopogonidae|GMPJA10330-21|BIOUG70233-E01|BOLD:ABY0384
Ceratopogonidae|GMPJA7312-21|BIOUG66630-H08|BOLD:ABY0384
Ceratopogonidae|GMPJA1442-21|BIOUG65670-F03|BOLD:ABY0384
Ceratopogonidae|GMPJA4246-21|BIOUG65699-F06|BOLD:ABY0384
Ceratopogonidae|GMPJA2422-21|BIOUG65680-H09|BOLD:ABY0384
Ceratopogonidae|GMPJA7896-21|BIOUG66637-A11|BOLD:ABY0384
Ceratopogonidae|GMPJA2320-21|BIOUG65679-H02|BOLD:ABY0384
Ceratopogonidae|GMPJA1423-21|BIOUG65670-D08|BOLD:ABY0384
Ceratopogonidae|GMPJA2264-21|BIOUG65679-C06|BOLD:ABY0384
Ceratopogonidae|GMPJA1952-21|BIOUG65676-A03|BOLD:ABY0384
Ceratopogonidae|GMPJA3507-21|BIOUG65691-H03|BOLD:ABY0384
Ceratopogonidae|GMPJA4487-21|BIOUG66602-B10|BOLD:ABY0384
Ceratopogonidae|GMPJA5170-21|BIOUG66608-D04|BOLD:ABY0384
Ceratopogonidae|GMPJA3292-21|BIOUG65689-F02|BOLD:ABY0384
Ceratopogonidae|GMPJA3460-21|BIOUG65691-D04|BOLD:ABY0384
Ceratopogonidae|GMPJA2506-21|BIOUG65681-G10|BOLD:ABY0384
Ceratopogonidae|GMPJA2278-21|BIOUG65679-D08|BOLD:ABY0384
Ceratopogonidae|GMPJA2157-21|BIOUG65678-B06|BOLD:ABY0384
Ceratopogonidae|GMPJA1816-21|BIOUG65674-E09|BOLD:ABY0384
Ceratopogonidae|GMPJA1372-21|BIOUG65669-H04|BOLD:ABY0384
Ceratopogonidae|GMPJA3211-21|BIOUG65688-G04|BOLD:ABY0384
Ceratopogonidae|GMPJA1827-21|BIOUG65674-F08|BOLD:ABY0384
Ceratopogonidae|GMPJA3348-21|BIOUG65690-B11|BOLD:ABY0384
Ceratopogonidae|GMPJA1515-21|BIOUG65671-D05|BOLD:ABY0384
Ceratopogonidae|GMPJA1927-21|BIOUG65675-G01|BOLD:ABY0384

Ceratopogonidae|GMPJA3348-21|BIOUG65690-B11|BOLD:ABY0384
Ceratopogonidae|GMPJA1515-21|BIOUG65671-D05|BOLD:ABY0384
Ceratopogonidae|GMPJA1927-21|BIOUG65675-G01|BOLD:ABY0384
Ceratopogonidae|GMPJA1371-21|BIOUG65669-H03|BOLD:ABY0384
Ceratopogonidae|GMPJA3408-21|BIOUG65690-G11|BOLD:ABY0384
Ceratopogonidae|GMPJA3145-21|BIOUG65688-A10|BOLD:ABY0384
Ceratopogonidae|GMPJA2031-21|BIOUG65676-G10|BOLD:ABY0384
Ceratopogonidae|GMPJA2309-21|BIOUG65679-G03|BOLD:ABY0384
Ceratopogonidae|GMPJA1624-21|BIOUG65672-E07|BOLD:ABY0384
Ceratopogonidae|GMPJA1897-21|BIOUG65675-D07|BOLD:ABY0384
Ceratopogonidae|GMPJA2101-21|BIOUG65677-E09|BOLD:ABY0384
Ceratopogonidae|GMPJA3464-21|BIOUG65691-D08|BOLD:ABY0384
Ceratopogonidae|GMPJA3571-21|BIOUG65692-E08|BOLD:ABY0384
Ceratopogonidae|GMPJA3381-21|BIOUG65690-E08|BOLD:ABY0384
Ceratopogonidae|GMPJA1707-21|BIOUG65673-D07|BOLD:ABY0384
Ceratopogonidae|GMPJA2312-21|BIOUG65679-G06|BOLD:ABY0384
Ceratopogonidae|GMPJA3570-21|BIOUG65692-E07|BOLD:ABY0384
Ceratopogonidae|GMPJA2109-21|BIOUG65677-F05|BOLD:ABY0384
Ceratopogonidae|GMPJA2213-21|BIOUG65678-G02|BOLD:ABY0384
Ceratopogonidae|GMPJA2076-21|BIOUG65677-C08|BOLD:ABY0384
Ceratopogonidae|GMPJA2475-21|BIOUG65681-E03|BOLD:ABY0384
Ceratopogonidae|GMPJA2468-21|BIOUG65681-D08|BOLD:ABY0384
Ceratopogonidae|GMPJA2439-21|BIOUG65681-B03|BOLD:ABY0384
Ceratopogonidae|GMPJA4118-21|BIOUG65698-C09|BOLD:ABY0384
Ceratopogonidae|GMPJA3109-21|BIOUG65687-F09|BOLD:ABY0384
Ceratopogonidae|GMPJA3680-21|BIOUG65693-F10|BOLD:ABY0384
Ceratopogonidae|GMPJA3232-21|BIOUG65689-A02|BOLD:ABY0384
Ceratopogonidae|GMPJA4222-21|BIOUG65699-D06|BOLD:ABY0384
Ceratopogonidae|GMPJA2151-21|BIOUG65678-A12|BOLD:ABY0384
Ceratopogonidae|GMPJA1721-21|BIOUG65673-E09|BOLD:ABY0384
Ceratopogonidae|GMPJA3552-21|BIOUG65692-D01|BOLD:ABY0384
Ceratopogonidae|GMPJA3306-21|BIOUG65689-G04|BOLD:ABY0384
Ceratopogonidae|GMPJA1424-21|BIOUG65670-D09|BOLD:ABY0384
Ceratopogonidae|GMPJA1527-21|BIOUG65671-E05|BOLD:ABY0384
Ceratopogonidae|GMPJA1291-21|BIOUG65669-A07|BOLD:ABY0384
Ceratopogonidae|GMPJA1967-21|BIOUG65676-B06|BOLD:ABY0384
Ceratopogonidae|GMPJA1925-21|BIOUG65675-F11|BOLD:ABY0384
Ceratopogonidae|GMPJA3237-21|BIOUG65689-A07|BOLD:ABY0384
Ceratopogonidae|GMPJA1543-21|BIOUG65671-F09|BOLD:ABY0384
Ceratopogonidae|GMPJA3124-21|BIOUG65687-G12|BOLD:ABY0384
Ceratopogonidae|GMPJA3376-21|BIOUG65690-E03|BOLD:ABY0384
Ceratopogonidae|GMPJA1495-21|BIOUG65671-B09|BOLD:ABY0384
Ceratopogonidae|GMPJA3152-21|BIOUG65688-B05|BOLD:ABY0384
Ceratopogonidae|GMPJA3198-21|BIOUG65688-F03|BOLD:ABY0384
Ceratopogonidae|GMPJA2269-21|BIOUG65679-C11|BOLD:ABY0384
Ceratopogonidae|GMPJA1885-21|BIOUG65675-C07|BOLD:ABY0384
Ceratopogonidae|GMPJA1965-21|BIOUG65676-B04|BOLD:ABY0384
Ceratopogonidae|GMPJA8068-21|BIOUG66638-H04|BOLD:ABY0384
Ceratopogonidae|GMPJA1893-21|BIOUG65675-D03|BOLD:ABY0384
Ceratopogonidae|GMPJA1557-21|BIOUG65671-G11|BOLD:ABY0384
Ceratopogonidae|GMPJA1386-21|BIOUG65670-A07|BOLD:ABY0384
Ceratopogonidae|GMPJA4194-21|BIOUG65699-B02|BOLD:ABY0384
Ceratopogonidae|GMPJA1530-21|BIOUG65671-E08|BOLD:ABY0384
Ceratopogonidae|GMPJA1781-21|BIOUG65674-B10|BOLD:ABY0384
Ceratopogonidae|GMPJA3643-21|BIOUG65693-C09|BOLD:ABY0384
Ceratopogonidae|GMPJA3602-21|BIOUG65692-H03|BOLD:ABY0384
Ceratopogonidae|GMPJA3594-21|BIOUG65692-G07|BOLD:ABY0384
Ceratopogonidae|GMPJA2428-21|BIOUG65681-A04|BOLD:ABY0384
Ceratopogonidae|GMPJA10291-21|BIOUG70233-A10|BOLD:ADT6101
Ceratopogonidae|GMPJA10237-21|BIOUG70232-E03|BOLD:ADT6101
Ceratopogonidae|GMPJA10209-21|BIOUG70232-B11|BOLD:ADT6101
Culicoides similis|GMPJA2237-21|BIOUG65679-A03|BOLD:ACM9587
Culicoides similis|GMPJA1505-21|BIOUG65671-C07|BOLD:ACM9587
Ceratopogonidae|GMPJA8000-21|BIOUG66638-B08|BOLD:ADT0350
Ceratopogonidae|GMPJA5180-21|BIOUG66608-E02|BOLD:ADT0350
Ceratopogonidae|GMPJA3037-21|BIOUG65686-H08|BOLD:ACR6752
Ceratopogonidae|GMPJA7788-21|BIOUG66635-H09|BOLD:ACR6752
Ceratopogonidae|GMPJA8611-21|BIOUG66644-F01|BOLD:ACR6752
Ceratopogonidae|GMPJA1645-21|BIOUG65672-G04|BOLD:ACR6752
Ceratopogonidae|GMPJA7765-21|BIOUG66635-F10|BOLD:ACR6752
Ceratopogonidae|GMPJA5394-21|BIOUG66610-G02|BOLD:ACI9501
Ceratopogonidae|GMPJA6874-21|BIOUG66626-C10|BOLD:ACI9501
Ceratopogonidae|GMPJA5496-21|BIOUG66611-G09|BOLD:ACI9501
Ceratopogonidae|GMPJA7742-21|BIOUG66635-D11|BOLD:ACI9501
Ceratopogonidae|GMPJA5264-21|BIOUG66609-D03|BOLD:ACI9501
Ceratopogonidae|GMPJA5254-21|BIOUG66609-C05|BOLD:ACI9501
Ceratopogonidae|GMPJA3107-21|BIOUG65687-F07|BOLD:ACI9501
Ceratopogonidae|GMPJA5375-21|BIOUG66610-E07|BOLD:ACI9501
Ceratopogonidae|GMPJA2427-21|BIOUG65681-A03|BOLD:ACI9501
Sergentomyia|GMPJA4048-21|BIOUG65697-E10|BOLD:ACD8588
Sergentomyia bailyi|GMPJA7568-21|BIOUG66633-F03|BOLD:ACD6049
Sergentomyia|GMPJA7595-21|BIOUG66633-H06|BOLD:ACD7168
Sergentomyia babu|GMPJA6434-21|BIOUG66621-F09|BOLD:ADS4902
Sergentomyia babu|GMPJA5307-21|BIOUG66609-G10|BOLD:ADS4902
Empididae|GMPJA738-21|BIOUG65663-B12|BOLD:ABW5536
Empididae|GMPJA763-21|BIOUG65663-E01|BOLD:ABW5536
Empididae|GMPJA645-21|BIOUG65662-C02|BOLD:ABW5536
Empididae|GMPJA762-21|BIOUG65663-D12|BOLD:ABW5536
Empididae|GMPJA4038-21|BIOUG65697-D12|BOLD:ABW5536
Scatopsidae|GMPJA10248-21|BIOUG70232-F02|BOLD:ACU7765
Calloptera asteria|GMPJA8454-21|BIOUG66642-H10|BOLD:AEC1863
Pachylophus|GMPJA065-21|BIOUG65656-B04|BOLD:AEJ8020
Pachylophus|GMPJA226-21|BIOUG65657-G10|BOLD:ACG0823
Pachylophus|GMPJA161-21|BIOUG65657-B05|BOLD:ACG0823
Pachylophus|GMPJA5473-21|BIOUG66611-E10|BOLD:ACF9854
Pachylophus|GMPJA486-21|BIOUG65660-E09|BOLD:ACF9854
Pachylophus|GMPJA492-21|BIOUG65660-F03|BOLD:ACF9854
Pachylophus|GMPJA4014-21|BIOUG65697-B12|BOLD:ACF9854
Pachylophus|GMPJA8040-21|BIOUG66638-E12|BOLD:ACF9854
Pachylophus|GMPJA8575-21|BIOUG66644-C01|BOLD:ACF9854
Pachylophus|GMPJA1883-21|BIOUG65675-C05|BOLD:ACF9854
Pachylophus|GMPJA5174-21|BIOUG66608-D08|BOLD:ACF9854
Psectrosciara|GMPJA8552-21|BIOUG66644-A02|BOLD:ABY0151

Pachylophus|GMPJA1883-21|BIOUG65675-C05|BOLD:ACF9854
Pachylophus|GMPJA5174-21|BIOUG66608-D08|BOLD:ACF9854
Psectrosciara|GMPJA8552-21|BIOUG66644-A02|BOLD:ABY0151
Psectrosciara|GMPJA5551-21|BIOUG66612-D05|BOLD:ABY0151
Amygdalops thomasseti|GMPJA1959-21|BIOUG65676-A10|BOLD:ACV8470
Chyromyidae|GMPJA5363-21|BIOUG66610-D07|BOLD:ACO3359
Carabidae|GMPJA7211-21|BIOUG66629-H02|BOLD:AEJ9737
Carabidae|GMPJA7203-21|BIOUG66629-G06|BOLD:AEJ9737
Carabidae|GMPJA7210-21|BIOUG66629-H01|BOLD:AEJ3165
Carabidae|GMPJA7184-21|BIOUG66629-E11|BOLD:AEJ3165
Carabidae|GMPJA7172-21|BIOUG66629-D11|BOLD:AEJ3165
Carabidae|GMPJA7205-21|BIOUG66629-G08|BOLD:AAP7997
Carabidae|GMPJA7204-21|BIOUG66629-G07|BOLD:AAP7997
Carabidae|GMPJA7199-21|BIOUG66629-G02|BOLD:AAP7997
Carabidae|GMPJA6325-21|BIOUG66620-E07|BOLD:ADZ8445
Carabidae|GMPJA7072-21|BIOUG66628-D06|BOLD:AEK2176
Carabidae|GMPJA6309-21|BIOUG66620-D03|BOLD:AAP7987
Carabidae|GMPJA6316-21|BIOUG66620-D10|BOLD:AAP7987
Carabidae|GMPJA7077-21|BIOUG66628-D11|BOLD:AAP7987
Carabidae|GMPJA7082-21|BIOUG66628-E04|BOLD:AAP7987
Carabidae|GMPJA7083-21|BIOUG66628-E05|BOLD:AAP7987
Carabidae|GMPJA6311-21|BIOUG66620-D05|BOLD:AAP7987
Carabidae|GMPJA6200-21|BIOUG66619-C01|BOLD:AAP7987
Psilopa|GMPJA4099-21|BIOUG65698-B02|BOLD:ABY7486
Psilopa|GMPJA4474-21|BIOUG66602-A09|BOLD:ABY7486
Psilopa|GMPJA3584-21|BIOUG65692-F09|BOLD:ABY7486
Psilopa|GMPJA4326-21|BIOUG65700-E03|BOLD:ABY7486
Psilopa|GMPJA3224-21|BIOUG65688-H05|BOLD:ABY7486
Psilopa|GMPJA4391-21|BIOUG66601-B09|BOLD:ABY7486
Psilopa|GMPJA4274-21|BIOUG65699-H10|BOLD:ABY7486
Psilopa|GMPJA3651-21|BIOUG65693-D05|BOLD:ABY7486
Psilopa|GMPJA3283-21|BIOUG65689-E05|BOLD:ABY7486
Psilopa|GMPJA3442-21|BIOUG65691-B10|BOLD:ABY7486
Psilopa|GMPJA4239-21|BIOUG65699-E11|BOLD:ABY7486
Psilopa|GMPJA4266-21|BIOUG65699-H02|BOLD:ABY7486
Psilopa|GMPJA4267-21|BIOUG65699-H03|BOLD:ABY7486
Psilopa|GMPJA4295-21|BIOUG65700-B08|BOLD:ABY7486
Psilopa|GMPJA4297-21|BIOUG65700-B10|BOLD:ABY7486
Psilopa|GMPJA4318-21|BIOUG65700-D07|BOLD:ABY7486
Psilopa|GMPJA3599-21|BIOUG65692-G12|BOLD:ABY7486
Psilopa|GMPJA4019-21|BIOUG65697-C05|BOLD:ABY7486
Psilopa|GMPJA4100-21|BIOUG65698-B03|BOLD:ABY7486
Psilopa|GMPJA368-21|BIOUG65659-C10|BOLD:ABY7486
Psilopa|GMPJA3455-21|BIOUG65691-C11|BOLD:ABY7486
Psilopa|GMPJA4544-21|BIOUG66602-G07|BOLD:ABY7486
Psilopa|GMPJA4523-21|BIOUG66602-E10|BOLD:ABY7486
Psilopa|GMPJA4520-21|BIOUG66602-E07|BOLD:ABY7486
Psilopa|GMPJA4512-21|BIOUG66602-D11|BOLD:ABY7486
Psilopa|GMPJA3679-21|BIOUG65693-F09|BOLD:ABY7486
Psilopa|GMPJA3191-21|BIOUG65688-E08|BOLD:ABY7486
Psilopa|GMPJA10342-21|BIOUG70233-F01|BOLD:ABY7486
Psilopa|GMPJA3052-21|BIOUG65687-A12|BOLD:ABY7486
Psilopa|GMPJA3266-21|BIOUG65689-C12|BOLD:ABY7486
Psilopa|GMPJA3103-21|BIOUG65687-F03|BOLD:ABY7486
Psilopa|GMPJA8718-21|BIOUG66645-G01|BOLD:ABY7486
Psilopa|GMPJA8426-21|BIOUG66642-F06|BOLD:ABY7486
Psilopa|GMPJA8128-21|BIOUG66639-E05|BOLD:ABY7486
Psilopa|GMPJA7759-21|BIOUG66635-F04|BOLD:ABY7486
Psilopa|GMPJA7287-21|BIOUG66630-F07|BOLD:ABY7486
Muscidae|GMPJA4550-21|BIOUG66602-H01|BOLD:ACP3429
Muscidae|GMPJA4434-21|BIOUG66601-F04|BOLD:ACP3429
Muscidae|GMPJA4275-21|BIOUG65699-H11|BOLD:ACP3429
Muscidae|GMPJA4525-21|BIOUG66602-E12|BOLD:ACP3429
Psilopa|GMPJA10233-21|BIOUG70232-D11|BOLD:ACV0965
Psilopa|GMPJA8347-21|BIOUG66641-G10|BOLD:ACV0965
Ephydridae|GMPJA10370-21|BIOUG70233-H05|BOLD:ACP4903
Ephydridae|GMPJA7292-21|BIOUG66630-F12|BOLD:ABW5510
Ephydridae|GMPJA8271-21|BIOUG66641-A06|BOLD:ABW5510
Ephydridae|GMPJA4321-21|BIOUG65700-D10|BOLD:ABW5510
Ephydridae|GMPJA10190-21|BIOUG70232-A04|BOLD:ABW5510
Ephydridae|GMPJA10238-21|BIOUG70232-E04|BOLD:ABW5510
Ephydridae|GMPJA10341-21|BIOUG70233-E12|BOLD:ABW5510
Ephydridae|GMPJA687-21|BIOUG65662-F08|BOLD:ABW5510
Ephydridae|GMPJA8194-21|BIOUG66640-B12|BOLD:ABW5510
Ephydridae|GMPJA8121-21|BIOUG66639-D10|BOLD:ABW5510
Ephydridae|GMPJA8103-21|BIOUG66639-C04|BOLD:ABW5510
Ephydridae|GMPJA7841-21|BIOUG66636-E03|BOLD:ABW5510
Ephydridae|GMPJA7577-21|BIOUG66633-F12|BOLD:ABW5510
Ephydridae|GMPJA7540-21|BIOUG66633-C11|BOLD:ABW5510
Ephydridae|GMPJA7250-21|BIOUG66630-C06|BOLD:ABW5510
Ephydridae|GMPJA7413-21|BIOUG66632-A03|BOLD:ABW5510
Ephydridae|GMPJA8482-21|BIOUG66643-C03|BOLD:AEK5168
Ephydridae|GMPJA8638-21|BIOUG66644-H04|BOLD:AEK5168
Ephydridae|GMPJA8634-21|BIOUG66644-G12|BOLD:AEK5168
Ephydridae|GMPJA7857-21|BIOUG66636-F07|BOLD:AEK5168
Ephydridae|GMPJA7839-21|BIOUG66636-E01|BOLD:AEK5168
Ephydridae|GMPJA7518-21|BIOUG66633-B01|BOLD:AEK5168
Ephydridae|GMPJA7497-21|BIOUG66632-H03|BOLD:AEK5168
Ephydridae|GMPJA7335-21|BIOUG66631-B08|BOLD:AEK5168
Ephydridae|GMPJA7275-21|BIOUG66630-E07|BOLD:AEK5168
Ephydridae|GMPJA10256-21|BIOUG70232-F10|BOLD:AEK5168
Ephydridae|GMPJA10334-21|BIOUG70233-E05|BOLD:AEK5168
Ephydridae|GMPJA7232-21|BIOUG66630-A12|BOLD:AEK5168
Ephydridae|GMPJA8411-21|BIOUG66642-E03|BOLD:AEK5168
Ephydridae|GMPJA10321-21|BIOUG70233-D04|BOLD:AEK5168
Ephydridae|GMPJA8631-21|BIOUG66644-G09|BOLD:AEK5168
Ephydridae|GMPJA8038-21|BIOUG66638-E10|BOLD:AEK5168
Ephydridae|GMPJA7370-21|BIOUG66631-E07|BOLD:AEK5168
Ephydridae|GMPJA7554-21|BIOUG66633-E01|BOLD:AEK5168
Ephydridae|GMPJA7757-21|BIOUG66635-F02|BOLD:AEK5168
Ephydridae|GMPJA7668-21|BIOUG66634-F08|BOLD:AEK5168
Ephydridae|GMPJA7843-21|BIOUG66636-E05|BOLD:AEK5168
Ephydridae|GMPJA7810-21|BIOUG66636-B08|BOLD:AEK5168

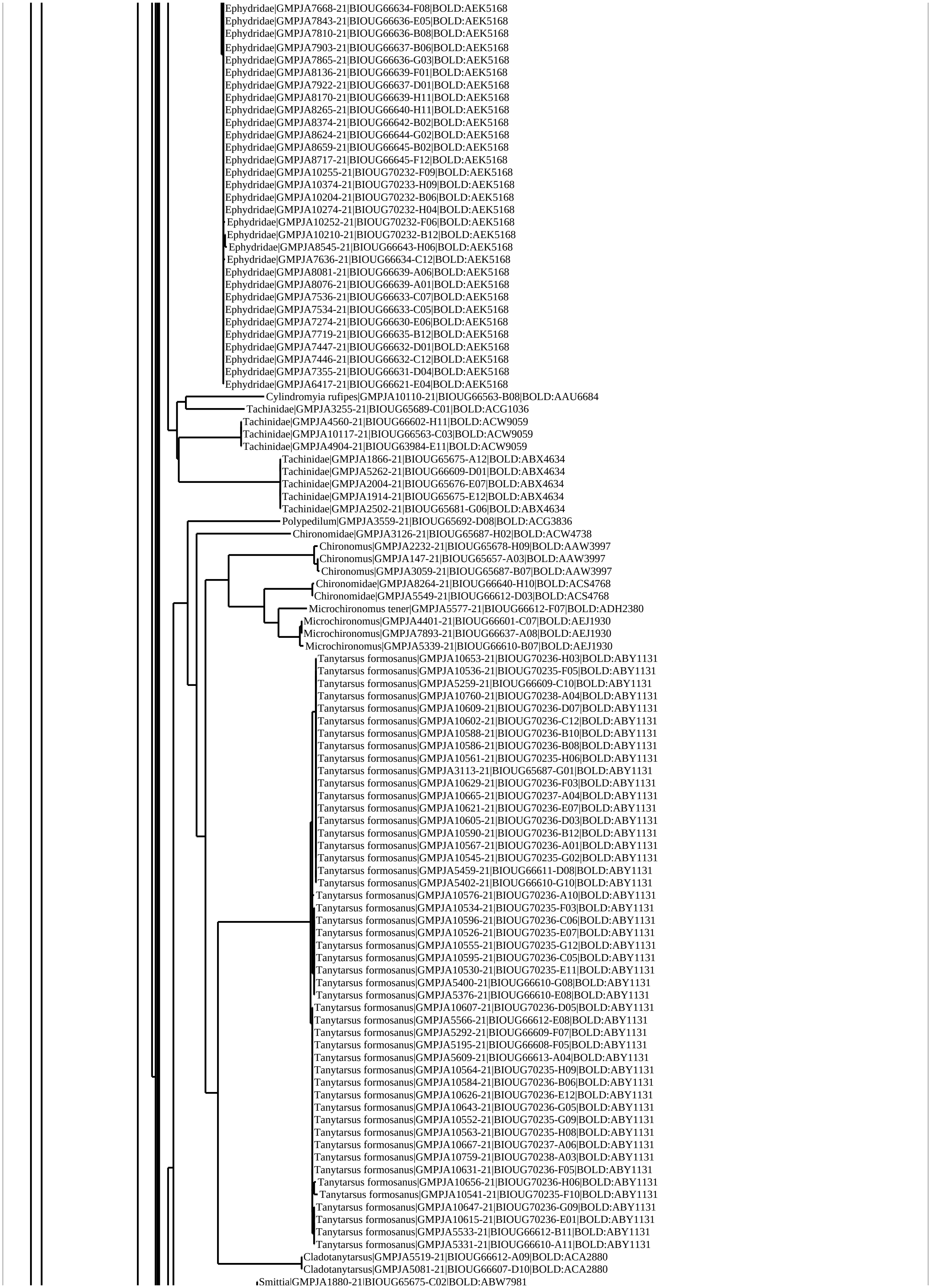
Ephydridae|GMPJA7668-21|BIOUG66634-F08|BOLD:AEK5168
Ephydridae|GMPJA7843-21|BIOUG66636-E05|BOLD:AEK5168
Ephydridae|GMPJA7810-21|BIOUG66636-B08|BOLD:AEK5168
Ephydridae|GMPJA7903-21|BIOUG66637-B06|BOLD:AEK5168
Ephydridae|GMPJA7865-21|BIOUG66636-G03|BOLD:AEK5168
Ephydridae|GMPJA8136-21|BIOUG66639-F01|BOLD:AEK5168
Ephydridae|GMPJA7922-21|BIOUG66637-D01|BOLD:AEK5168
Ephydridae|GMPJA8170-21|BIOUG66639-H11|BOLD:AEK5168
Ephydridae|GMPJA8265-21|BIOUG66640-H11|BOLD:AEK5168
Ephydridae|GMPJA8374-21|BIOUG66642-B02|BOLD:AEK5168
Ephydridae|GMPJA8624-21|BIOUG66644-G02|BOLD:AEK5168
Ephydridae|GMPJA8659-21|BIOUG66645-B02|BOLD:AEK5168
Ephydridae|GMPJA8717-21|BIOUG66645-F12|BOLD:AEK5168
Ephydridae|GMPJA10255-21|BIOUG70232-F09|BOLD:AEK5168
Ephydridae|GMPJA10374-21|BIOUG70233-H09|BOLD:AEK5168
Ephydridae|GMPJA10204-21|BIOUG70232-B06|BOLD:AEK5168
Ephydridae|GMPJA10274-21|BIOUG70232-H04|BOLD:AEK5168
Ephydridae|GMPJA10252-21|BIOUG70232-F06|BOLD:AEK5168
Ephydridae|GMPJA10210-21|BIOUG70232-B12|BOLD:AEK5168
Ephydridae|GMPJA8545-21|BIOUG66643-H06|BOLD:AEK5168
Ephydridae|GMPJA7636-21|BIOUG66634-C12|BOLD:AEK5168
Ephydridae|GMPJA8081-21|BIOUG66639-A06|BOLD:AEK5168
Ephydridae|GMPJA8076-21|BIOUG66639-A01|BOLD:AEK5168
Ephydridae|GMPJA7536-21|BIOUG66633-C07|BOLD:AEK5168
Ephydridae|GMPJA7534-21|BIOUG66633-C05|BOLD:AEK5168
Ephydridae|GMPJA7274-21|BIOUG66630-E06|BOLD:AEK5168
Ephydridae|GMPJA7719-21|BIOUG66635-B12|BOLD:AEK5168
Ephydridae|GMPJA7447-21|BIOUG66632-D01|BOLD:AEK5168
Ephydridae|GMPJA7446-21|BIOUG66632-C12|BOLD:AEK5168
Ephydridae|GMPJA7355-21|BIOUG66631-D04|BOLD:AEK5168
Ephydridae|GMPJA6417-21|BIOUG66621-E04|BOLD:AEK5168
Cylindromyia rufipes|GMPJA10110-21|BIOUG66563-B08|BOLD:AAU6684
Tachinidae|GMPJA3255-21|BIOUG65689-C01|BOLD:ACG1036
Tachinidae|GMPJA4560-21|BIOUG66602-H11|BOLD:ACW9059
Tachinidae|GMPJA10117-21|BIOUG66563-C03|BOLD:ACW9059
Tachinidae|GMPJA4904-21|BIOUG63984-E11|BOLD:ACW9059
Tachinidae|GMPJA1866-21|BIOUG65675-A12|BOLD:ABX4634
Tachinidae|GMPJA5262-21|BIOUG66609-D01|BOLD:ABX4634
Tachinidae|GMPJA2004-21|BIOUG65676-E07|BOLD:ABX4634
Tachinidae|GMPJA1914-21|BIOUG65675-E12|BOLD:ABX4634
Tachinidae|GMPJA2502-21|BIOUG65681-G06|BOLD:ABX4634
Polypedilum|GMPJA3559-21|BIOUG65692-D08|BOLD:ACG3836
Chironomidae|GMPJA3126-21|BIOUG65687-H02|BOLD:ACW4738
Chironomus|GMPJA2232-21|BIOUG65678-H09|BOLD:AAW3997
Chironomus|GMPJA147-21|BIOUG65657-A03|BOLD:AAW3997
Chironomus|GMPJA3059-21|BIOUG65687-B07|BOLD:AAW3997
Chironomidae|GMPJA8264-21|BIOUG66640-H10|BOLD:ACS4768
Chironomidae|GMPJA5549-21|BIOUG66612-D03|BOLD:ACS4768
Microchironomus tener|GMPJA5577-21|BIOUG66612-F07|BOLD:ADH2380
Microchironomus|GMPJA4401-21|BIOUG66601-C07|BOLD:AEJ1930
Microchironomus|GMPJA7893-21|BIOUG66637-A08|BOLD:AEJ1930
Microchironomus|GMPJA5339-21|BIOUG66610-B07|BOLD:AEJ1930
Tanytarsus formosanus|GMPJA10653-21|BIOUG70236-H03|BOLD:ABY1131
Tanytarsus formosanus|GMPJA10536-21|BIOUG70235-F05|BOLD:ABY1131
Tanytarsus formosanus|GMPJA5259-21|BIOUG66609-C10|BOLD:ABY1131
Tanytarsus formosanus|GMPJA10760-21|BIOUG70238-A04|BOLD:ABY1131
Tanytarsus formosanus|GMPJA10609-21|BIOUG70236-D07|BOLD:ABY1131
Tanytarsus formosanus|GMPJA10602-21|BIOUG70236-C12|BOLD:ABY1131
Tanytarsus formosanus|GMPJA10588-21|BIOUG70236-B10|BOLD:ABY1131
Tanytarsus formosanus|GMPJA10586-21|BIOUG70236-B08|BOLD:ABY1131
Tanytarsus formosanus|GMPJA10561-21|BIOUG70235-H06|BOLD:ABY1131
Tanytarsus formosanus|GMPJA3113-21|BIOUG65687-G01|BOLD:ABY1131
Tanytarsus formosanus|GMPJA10629-21|BIOUG70236-F03|BOLD:ABY1131
Tanytarsus formosanus|GMPJA10665-21|BIOUG70237-A04|BOLD:ABY1131
Tanytarsus formosanus|GMPJA10621-21|BIOUG70236-E07|BOLD:ABY1131
Tanytarsus formosanus|GMPJA10605-21|BIOUG70236-D03|BOLD:ABY1131
Tanytarsus formosanus|GMPJA10590-21|BIOUG70236-B12|BOLD:ABY1131
Tanytarsus formosanus|GMPJA10567-21|BIOUG70236-A01|BOLD:ABY1131
Tanytarsus formosanus|GMPJA10545-21|BIOUG70235-G02|BOLD:ABY1131
Tanytarsus formosanus|GMPJA5459-21|BIOUG66611-D08|BOLD:ABY1131
Tanytarsus formosanus|GMPJA5402-21|BIOUG66610-G10|BOLD:ABY1131
Tanytarsus formosanus|GMPJA10576-21|BIOUG70236-A10|BOLD:ABY1131
Tanytarsus formosanus|GMPJA10534-21|BIOUG70235-F03|BOLD:ABY1131
Tanytarsus formosanus|GMPJA10596-21|BIOUG70236-C06|BOLD:ABY1131
Tanytarsus formosanus|GMPJA10526-21|BIOUG70235-E07|BOLD:ABY1131
Tanytarsus formosanus|GMPJA10555-21|BIOUG70235-G12|BOLD:ABY1131
Tanytarsus formosanus|GMPJA10595-21|BIOUG70236-C05|BOLD:ABY1131
Tanytarsus formosanus|GMPJA10530-21|BIOUG70235-E11|BOLD:ABY1131
Tanytarsus formosanus|GMPJA5400-21|BIOUG66610-G08|BOLD:ABY1131
Tanytarsus formosanus|GMPJA5376-21|BIOUG66610-E08|BOLD:ABY1131
Tanytarsus formosanus|GMPJA10607-21|BIOUG70236-D05|BOLD:ABY1131
Tanytarsus formosanus|GMPJA5566-21|BIOUG66612-E08|BOLD:ABY1131
Tanytarsus formosanus|GMPJA5292-21|BIOUG66609-F07|BOLD:ABY1131
Tanytarsus formosanus|GMPJA5195-21|BIOUG66608-F05|BOLD:ABY1131
Tanytarsus formosanus|GMPJA5609-21|BIOUG66613-A04|BOLD:ABY1131
Tanytarsus formosanus|GMPJA10564-21|BIOUG70235-H09|BOLD:ABY1131
Tanytarsus formosanus|GMPJA10584-21|BIOUG70236-B06|BOLD:ABY1131
Tanytarsus formosanus|GMPJA10626-21|BIOUG70236-E12|BOLD:ABY1131
Tanytarsus formosanus|GMPJA10643-21|BIOUG70236-G05|BOLD:ABY1131
Tanytarsus formosanus|GMPJA10552-21|BIOUG70235-G09|BOLD:ABY1131
Tanytarsus formosanus|GMPJA10563-21|BIOUG70235-H08|BOLD:ABY1131
Tanytarsus formosanus|GMPJA10667-21|BIOUG70237-A06|BOLD:ABY1131
Tanytarsus formosanus|GMPJA10759-21|BIOUG70238-A03|BOLD:ABY1131
Tanytarsus formosanus|GMPJA10631-21|BIOUG70236-F05|BOLD:ABY1131
Tanytarsus formosanus|GMPJA10656-21|BIOUG70236-H06|BOLD:ABY1131
Tanytarsus formosanus|GMPJA10541-21|BIOUG70235-F10|BOLD:ABY1131
Tanytarsus formosanus|GMPJA10647-21|BIOUG70236-G09|BOLD:ABY1131
Tanytarsus formosanus|GMPJA10615-21|BIOUG70236-E01|BOLD:ABY1131
Tanytarsus formosanus|GMPJA5533-21|BIOUG66612-B11|BOLD:ABY1131
Tanytarsus formosanus|GMPJA5331-21|BIOUG66610-A11|BOLD:ABY1131
Cladotanytarsus|GMPJA5519-21|BIOUG66612-A09|BOLD:ACA2880
Cladotanytarsus|GMPJA5081-21|BIOUG66607-D10|BOLD:ACA2880
Smittia|GMPJA1880-21|BIOUG65675-C02|BOLD:ABW7981

Cladotanytarsus|GMPJA5519-21|BIOUG66612-A09|BOLD:ACA2880
Cladotanytarsus|GMPJA5081-21|BIOUG66607-D10|BOLD:ACA2880
Smittia|GMPJA1880-21|BIOUG65675-C02|BOLD:ABW7981
Smittia|GMPJA1043-21|BIOUG65666-D08|BOLD:ABW7981
Smittia|GMPJA697-21|BIOUG65662-G06|BOLD:ABW7981
Smittia|GMPJA640-21|BIOUG65662-B09|BOLD:ABW7981
Smittia|GMPJA179-21|BIOUG65657-C11|BOLD:ABW7981
Smittia|GMPJA114-21|BIOUG65656-F05|BOLD:ABW7981
Smittia|GMPJA10601-21|BIOUG70236-C11|BOLD:ABW7981
Tanypus|GMPJA3038-21|BIOUG65686-H09|BOLD:ADO6236
Chironomidae|GMPJA4307-21|BIOUG65700-C08|BOLD:AEK0720
Chironomidae|GMPJA627-21|BIOUG65662-A08|BOLD:ABW5533
Chironomidae|GMPJA672-21|BIOUG65662-E05|BOLD:ABW5533
Chironomidae|GMPJA667-21|BIOUG65662-D12|BOLD:ABW5533
Chironomidae|GMPJA425-21|BIOUG65659-H07|BOLD:ABW5533
Chironomidae|GMPJA354-21|BIOUG65659-B08|BOLD:ABW5533
Chironomidae|GMPJA382-21|BIOUG65659-D12|BOLD:ABW5533
Chironomidae|GMPJA369-21|BIOUG65659-C11|BOLD:ABW5533
Chironomidae|GMPJA919-21|BIOUG65665-B03|BOLD:ABW5533
Chironomidae|GMPJA433-21|BIOUG65660-A04|BOLD:ABW5533
Chironomidae|GMPJA408-21|BIOUG65659-G02|BOLD:ABW5533
Chironomidae|GMPJA689-21|BIOUG65662-F10|BOLD:ACV3309
Chironomidae|GMPJA3513-21|BIOUG65691-H09|BOLD:ACV3309
Chironomidae|GMPJA360-21|BIOUG65659-C02|BOLD:ACV3309
Chironomidae|GMPJA505-21|BIOUG65660-G04|BOLD:ACV3309
Chironomidae|GMPJA437-21|BIOUG65660-A08|BOLD:ACV3309
Chironomidae|GMPJA636-21|BIOUG65662-B05|BOLD:ACV3309
Chironomidae|GMPJA622-21|BIOUG65662-A03|BOLD:ACV3309
Chironomidae|GMPJA381-21|BIOUG65659-D11|BOLD:ACV3309
Chironomidae|GMPJA456-21|BIOUG65660-C03|BOLD:ACV3309
Chironomidae|GMPJA730-21|BIOUG65663-B04|BOLD:ACV3309
Chironomidae|GMPJA401-21|BIOUG65659-F07|BOLD:ACV3309
Chironomidae|GMPJA478-21|BIOUG65660-E01|BOLD:ABW5508
Chironomidae|GMPJA749-21|BIOUG65663-C11|BOLD:ABW5508
Chironomidae|GMPJA979-21|BIOUG65665-G03|BOLD:ABW5508
Chironomidae|GMPJA484-21|BIOUG65660-E07|BOLD:ABW5508
Chironomidae|GMPJA737-21|BIOUG65663-B11|BOLD:ABW5508
Chironomidae|GMPJA1153-21|BIOUG65667-E11|BOLD:ABW5508
Chironomidae|GMPJA760-21|BIOUG65663-D10|BOLD:ABW5508
Chironomidae|GMPJA491-21|BIOUG65660-F02|BOLD:ABW5508
Chironomidae|GMPJA464-21|BIOUG65660-C11|BOLD:ABW5508
Chironomidae|GMPJA918-21|BIOUG65665-B02|BOLD:ABW5508
Chironomidae|GMPJA1120-21|BIOUG65667-C02|BOLD:ABW5508
Chironomidae|GMPJA1177-21|BIOUG65667-G11|BOLD:ABW5508
Chironomidae|GMPJA913-21|BIOUG65665-A09|BOLD:ABW5508
Chironomidae|GMPJA447-21|BIOUG65660-B06|BOLD:ABW5508
Chironomidae|GMPJA964-21|BIOUG65665-E12|BOLD:ABW5508
Chironomidae|GMPJA353-21|BIOUG65659-B07|BOLD:ABW5508
Chironomidae|GMPJA647-21|BIOUG65662-C04|BOLD:ABW5508
Chironomidae|GMPJA756-21|BIOUG65663-D06|BOLD:ABW5508
Chironomidae|GMPJA4645-21|BIOUG66603-H01|BOLD:ABW5508
Chironomidae|GMPJA716-21|BIOUG65663-A02|BOLD:ABW5508
Chironomidae|GMPJA415-21|BIOUG65659-G09|BOLD:ABW5508
Chironomidae|GMPJA423-21|BIOUG65659-H05|BOLD:ABW5508
Chironomidae|GMPJA519-21|BIOUG65660-H06|BOLD:ABW5508
Chironomidae|GMPJA660-21|BIOUG65662-D05|BOLD:ABW5508
Chironomidae|GMPJA535-21|BIOUG65661-A11|BOLD:ABW5508
Chironomidae|GMPJA755-21|BIOUG65663-D05|BOLD:ABW5508
Chironomidae|GMPJA542-21|BIOUG65661-B06|BOLD:ABW5508
Chironomidae|GMPJA420-21|BIOUG65659-H02|BOLD:ABW5508
Chironomidae|GMPJA434-21|BIOUG65660-A05|BOLD:ABW5508
Chironomidae|GMPJA460-21|BIOUG65660-C07|BOLD:ABW5508
Chironomidae|GMPJA485-21|BIOUG65660-E08|BOLD:ABW5508
Chironomidae|GMPJA378-21|BIOUG65659-D08|BOLD:ABW5508
Chironomidae|GMPJA623-21|BIOUG65662-A04|BOLD:ABW5508
Chironomidae|GMPJA680-21|BIOUG65662-F01|BOLD:ABW5508
Chironomidae|GMPJA743-21|BIOUG65663-C05|BOLD:ABW5508
Chironomidae|GMPJA540-21|BIOUG65661-B04|BOLD:ABW5508
Chironomidae|GMPJA678-21|BIOUG65662-E11|BOLD:ABW5508
Chironomidae|GMPJA692-21|BIOUG65662-G01|BOLD:ABW5508
Chironomidae|GMPJA696-21|BIOUG65662-G05|BOLD:ABW5508
Chironomidae|GMPJA708-21|BIOUG65662-H05|BOLD:ABW5508
Chironomidae|GMPJA725-21|BIOUG65663-A11|BOLD:ABW5508
Chironomidae|GMPJA732-21|BIOUG65663-B06|BOLD:ABW5508
Chironomidae|GMPJA758-21|BIOUG65663-D08|BOLD:ABW5508
Chironomidae|GMPJA1038-21|BIOUG65666-D03|BOLD:ABW5508
Chironomidae|GMPJA1157-21|BIOUG65667-F03|BOLD:ABW5508
Chironomidae|GMPJA1171-21|BIOUG65667-G05|BOLD:ABW5508
Chironomidae|GMPJA1198-21|BIOUG65668-A09|BOLD:ABW5508
Chironomidae|GMPJA516-21|BIOUG65660-H03|BOLD:ABW5508
Chironomidae|GMPJA677-21|BIOUG65662-E10|BOLD:ABW5508
Chironomidae|GMPJA345-21|BIOUG65659-A11|BOLD:ABW5508
Chironomidae|GMPJA717-21|BIOUG65663-A03|BOLD:ABW5508
Chironomidae|GMPJA1103-21|BIOUG65667-A09|BOLD:ABW5508
Chironomidae|GMPJA501-21|BIOUG65660-F12|BOLD:ABW5508
Chironomidae|GMPJA658-21|BIOUG65662-D03|BOLD:ABW5508
Chironomidae|GMPJA909-21|BIOUG65665-A05|BOLD:ABW5508
Chironomidae|GMPJA764-21|BIOUG65663-E02|BOLD:ABW5508
Chironomidae|GMPJA705-21|BIOUG65662-H02|BOLD:ABW5508
Chironomidae|GMPJA373-21|BIOUG65659-D03|BOLD:ABW5508
Chironomidae|GMPJA759-21|BIOUG65663-D09|BOLD:ABW5508
Chironomidae|GMPJA4145-21|BIOUG65698-E12|BOLD:ABW5508
Chironomidae|GMPJA673-21|BIOUG65662-E06|BOLD:ABW5508
Chironomidae|GMPJA662-21|BIOUG65662-D07|BOLD:ABW5508
Chironomidae|GMPJA657-21|BIOUG65662-D02|BOLD:ABW5508
Chironomidae|GMPJA638-21|BIOUG65662-B07|BOLD:ABW5508
Chironomidae|GMPJA386-21|BIOUG65659-E04|BOLD:ABW5508
Chironomidae|GMPJA366-21|BIOUG65659-C08|BOLD:ABW5508
Chironomidae|GMPJA355-21|BIOUG65659-B09|BOLD:ABW5508
Chironomidae|GMPJA349-21|BIOUG65659-B03|BOLD:ABW5508
Chironomidae|GMPJA541-21|BIOUG65661-B05|BOLD:ABW5508
Chironomidae|GMPJA534-21|BIOUG65661-A10|BOLD:ABW5508
Chironomidae|GMPJA518-21|BIOUG65660-H05|BOLD:ABW5508

[illegible]
Chironomidae|GMPJA541-21|BIOUG65661-B05|BOLD:ABW5508
Chironomidae|GMPJA534-21|BIOUG65661-A10|BOLD:ABW5508
Chironomidae|GMPJA518-21|BIOUG65660-H05|BOLD:ABW5508
Chironomidae|GMPJA506-21|BIOUG65660-G05|BOLD:ABW5508
Chironomidae|GMPJA488-21|BIOUG65660-E11|BOLD:ABW5508
Chironomidae|GMPJA482-21|BIOUG65660-E05|BOLD:ABW5508
Chironomidae|GMPJA480-21|BIOUG65660-E03|BOLD:ABW5508
Chironomidae|GMPJA471-21|BIOUG65660-D06|BOLD:ABW5508
Chironomidae|GMPJA459-21|BIOUG65660-C06|BOLD:ABW5508
Chironomidae|GMPJA458-21|BIOUG65660-C05|BOLD:ABW5508
Chironomidae|GMPJA422-21|BIOUG65659-H04|BOLD:ABW5508
Chironomidae|GMPJA410-21|BIOUG65659-G04|BOLD:ABW5508
Chironomidae|GMPJA403-21|BIOUG65659-F09|BOLD:ABW5508
Chironomidae|GMPJA402-21|BIOUG65659-F08|BOLD:ABW5508
Chironomidae|GMPJA398-21|BIOUG65659-F04|BOLD:ABW5508
Chironomidae|GMPJA392-21|BIOUG65659-E10|BOLD:ABW5508
Chironomidae|GMPJA3175-21|BIOUG65688-D04|BOLD:ABW5508
Chironomidae|GMPJA10634-21|BIOUG70236-F08|BOLD:ABW5508
Chironomidae|GMPJA4093-21|BIOUG65698-A08|BOLD:ACP5165
Orthocladiinae|GMPJA1943-21|BIOUG65675-H05|BOLD:AAP5926
Orthocladiinae|GMPJA2280-21|BIOUG65679-D10|BOLD:AAP5926
Chironomidae|GMPJA1374-21|BIOUG65669-H06|BOLD:ABW5549
Chironomidae|GMPJA1792-21|BIOUG65674-C09|BOLD:ABW5549
Chironomidae|GMPJA2299-21|BIOUG65679-F05|BOLD:ABW5549
Chironomidae|GMPJA489-21|BIOUG65660-E12|BOLD:ABW5549
Chironomidae|GMPJA476-21|BIOUG65660-D11|BOLD:ABW5549
Chironomidae|GMPJA520-21|BIOUG65660-H07|BOLD:ABW5549
Chironomidae|GMPJA1572-21|BIOUG65672-A03|BOLD:ABW5549
Chironomidae|GMPJA7749-21|BIOUG66635-E06|BOLD:ABW5549
Chironomidae|GMPJA1842-21|BIOUG65674-G11|BOLD:ABW5549
Chironomidae|GMPJA987-21|BIOUG65665-G11|BOLD:ABW5549
Chironomidae|GMPJA472-21|BIOUG65660-D07|BOLD:ABW5549
Chironomidae|GMPJA693-21|BIOUG65662-G02|BOLD:ABW5549
Chironomidae|GMPJA1115-21|BIOUG65667-B09|BOLD:ABW5549
Chironomidae|GMPJA1003-21|BIOUG65666-A04|BOLD:ABW5549
Chironomidae|GMPJA761-21|BIOUG65663-D11|BOLD:ABW5549
Chironomidae|GMPJA736-21|BIOUG65663-B10|BOLD:ABW5549
Chironomidae|GMPJA724-21|BIOUG65663-A10|BOLD:ABW5549
Chironomidae|GMPJA1301-21|BIOUG65669-B05|BOLD:ABW5549
Chironomidae|GMPJA694-21|BIOUG65662-G03|BOLD:ABW5549
Chironomidae|GMPJA651-21|BIOUG65662-C08|BOLD:ABW5549
Chironomidae|GMPJA976-21|BIOUG65665-F12|BOLD:ABW5549
Chironomidae|GMPJA954-21|BIOUG65665-E02|BOLD:ABW5549
Chironomidae|GMPJA1136-21|BIOUG65667-D06|BOLD:ABW5549
Chironomidae|GMPJA1129-21|BIOUG65667-C11|BOLD:ABW5549
Chironomidae|GMPJA932-21|BIOUG65665-C04|BOLD:ABW5549
Chironomidae|GMPJA223-21|BIOUG65657-G07|BOLD:ABW5549
Chironomidae|GMPJA1811-21|BIOUG65674-E04|BOLD:ABW5549
Chironomidae|GMPJA1140-21|BIOUG65667-D10|BOLD:ABW5549
Chironomidae|GMPJA1856-21|BIOUG65675-A02|BOLD:ABW5549
Chironomidae|GMPJA1911-21|BIOUG65675-E09|BOLD:ABW5549
Chironomidae|GMPJA1933-21|BIOUG65675-G07|BOLD:ABW5549
Chironomidae|GMPJA1458-21|BIOUG65670-G07|BOLD:ABW5549
Chironomidae|GMPJA1471-21|BIOUG65670-H08|BOLD:ABW5549
Chironomidae|GMPJA1546-21|BIOUG65671-F12|BOLD:ABW5549
Chironomidae|GMPJA2201-21|BIOUG65678-F02|BOLD:ABW5549
Chironomidae|GMPJA1597-21|BIOUG65672-C04|BOLD:ABW5549
Chironomidae|GMPJA1156-21|BIOUG65667-F02|BOLD:ABW5549
Chironomidae|GMPJA1088-21|BIOUG65666-H05|BOLD:ABW5549
Chironomidae|GMPJA1524-21|BIOUG65671-E02|BOLD:ABW5549
Chironomidae|GMPJA1577-21|BIOUG65672-A08|BOLD:ABW5549
Chironomidae|GMPJA2239-21|BIOUG65679-A05|BOLD:ABW5549
Chironomidae|GMPJA1675-21|BIOUG65673-A11|BOLD:ABW5549
Chironomidae|GMPJA2121-21|BIOUG65677-G05|BOLD:ABW5549
Chironomidae|GMPJA679-21|BIOUG65662-E12|BOLD:ABW5549
Chironomidae|GMPJA545-21|BIOUG65661-B09|BOLD:ABW5549
Chironomidae|GMPJA8024-21|BIOUG66638-D08|BOLD:ABW5549
Chironomidae|GMPJA10659-21|BIOUG70236-H09|BOLD:ADS4063
Chironomidae|GMPJA5091-21|BIOUG66607-E08|BOLD:ACF8142
Chironomidae|GMPJA6854-21|BIOUG66626-B02|BOLD:ACF8142
Chironomidae|GMPJA7463-21|BIOUG66632-E05|BOLD:ACF8142
Chironomidae|GMPJA6465-21|BIOUG66622-A05|BOLD:ACF8142
Chironomidae|GMPJA5204-21|BIOUG66608-G02|BOLD:ACF8142
Chironomidae|GMPJA5370-21|BIOUG66610-E02|BOLD:ACF8142
Chironomidae|GMPJA5062-21|BIOUG66607-C03|BOLD:ACF8142
Chironomidae|GMPJA5045-21|BIOUG66607-A10|BOLD:ACF8142
Nephrotoma australasiae|GMPJA8870-21|BIOUG63985-E05|BOLD:AAI3208
Colobostema|GMPJA8536-21|BIOUG66643-G09|BOLD:AAG8389
Colobostema|GMPJA8346-21|BIOUG66641-G09|BOLD:AAG8389
Colobostema|GMPJA9168-21|BIOUG66650-A03|BOLD:AAG8389
Colobostema|GMPJA8254-21|BIOUG66640-G12|BOLD:AAG8389
Colobostema|GMPJA7874-21|BIOUG66636-G12|BOLD:AAG8389
Colobostema|GMPJA7496-21|BIOUG66632-H02|BOLD:AAG8389
Colobostema|GMPJA7409-21|BIOUG66631-H10|BOLD:AAG8389
Scatopsidae|GMPJA7809-21|BIOUG66636-B07|BOLD:ACX6638
Scatopsidae|GMPJA7266-21|BIOUG66630-D10|BOLD:ACX6638
Scatopsidae|GMPJA7260-21|BIOUG66630-D04|BOLD:ACX6638
Scatopsidae|GMPJA7429-21|BIOUG66632-B07|BOLD:ACX6638
Scatopsidae|GMPJA2244-21|BIOUG65679-A10|BOLD:ABX8773
Scatopsidae|GMPJA2410-21|BIOUG65680-G09|BOLD:ABX8773
Scatopsidae|GMPJA2392-21|BIOUG65680-F03|BOLD:ABX8773
Scatopsidae|GMPJA2040-21|BIOUG65676-H07|BOLD:ABX8773
Scatopsidae|GMPJA2016-21|BIOUG65676-F07|BOLD:ABX8773
Scatopsidae|GMPJA1520-21|BIOUG65671-D10|BOLD:ABX8773
Scatopsidae|GMPJA2055-21|BIOUG65677-A11|BOLD:ABX8773
Scatopsidae|GMPJA1972-21|BIOUG65676-B11|BOLD:ABX8773
Scatopsidae|GMPJA1320-21|BIOUG65669-C12|BOLD:ABX8773
Scatopsidae|GMPJA1790-21|BIOUG65674-C07|BOLD:ADT9699
Scatopsidae|GMPJA1300-21|BIOUG65669-B04|BOLD:ADT9699
Scatopsidae|GMPJA8124-21|BIOUG66639-E01|BOLD:ADT9699
Scatopsidae|GMPJA7586-21|BIOUG66633-G09|BOLD:ADT9699
Empididae|GMPJA1510-21|BIOUG65671-C12|BOLD:AEK0868
Empididae|GMPJA1951-21|BIOUG65676-A02|BOLD:AEK0868

[illegible]
Scatopsidae|GMPJA7586-21|BIOUG66633-G09|BOLD:ADT9699
Empididae|GMPJA1510-21|BIOUG65671-C12|BOLD:AEK0868
Empididae|GMPJA1951-21|BIOUG65676-A02|BOLD:AEK0868
Empididae|GMPJA1890-21|BIOUG65675-C12|BOLD:AEK0868
Coboldia fuscipes|GMPJA1537-21|BIOUG65671-F03|BOLD:AAN8523
Campiglossa sororcula|GMPJA10236-21|BIOUG70232-E02|BOLD:ACF4574
Scatopsidae|GMPJA7509-21|BIOUG66633-A04|BOLD:ACW4582
Scatopsidae|GMPJA8778-21|BIOUG66646-D02|BOLD:ACW4582
Scatopsidae|GMPJA8030-21|BIOUG66638-E02|BOLD:ACW4582
Scatopsidae|GMPJA7265-21|BIOUG66630-D09|BOLD:ACW4582
Scatopsidae|GMPJA10606-21|BIOUG70236-D04|BOLD:ACG0669
Scatopsidae|GMPJA8793-21|BIOUG66646-E05|BOLD:ACG0669
Scatopsidae|GMPJA5560-21|BIOUG66612-E02|BOLD:ACG0669
Scatopsidae|GMPJA7508-21|BIOUG66633-A03|BOLD:ACG0669
Scatopsidae|GMPJA2058-21|BIOUG65677-B02|BOLD:ACG0669
Scatopsidae|GMPJA10531-21|BIOUG70235-E12|BOLD:ACG0669
Scatopsidae|GMPJA10611-21|BIOUG70236-D09|BOLD:ACG0669
Scatopsidae|GMPJA8355-21|BIOUG66641-H06|BOLD:ACG0669
Scatopsidae|GMPJA5544-21|BIOUG66612-C10|BOLD:ACG0669
Scatopsidae|GMPJA2142-21|BIOUG65678-A03|BOLD:ACG0669
Scatopsidae|GMPJA8021-21|BIOUG66638-D05|BOLD:ACG0669
Scatopsidae|GMPJA4170-21|BIOUG65698-H01|BOLD:ACG0669
Scatopsidae|GMPJA5498-21|BIOUG66611-G11|BOLD:ACG0669
Scatopsidae|GMPJA5466-21|BIOUG66611-E03|BOLD:ACG0669
Scatopsidae|GMPJA8819-21|BIOUG66646-G07|BOLD:ACG0669
Scatopsidae|GMPJA8790-21|BIOUG66646-E02|BOLD:ACG0669
Scatopsidae|GMPJA8548-21|BIOUG66643-H09|BOLD:ACG0669
Scatopsidae|GMPJA8144-21|BIOUG66639-F09|BOLD:ACG0669
Scatopsidae|GMPJA8550-21|BIOUG66643-H11|BOLD:ACG0669
Scatopsidae|GMPJA8532-21|BIOUG66643-G05|BOLD:ACG0669
Scatopsidae|GMPJA10625-21|BIOUG70236-E11|BOLD:ACG0669
Scatopsidae|GMPJA7986-21|BIOUG66638-A06|BOLD:ACG0669
Scatopsidae|GMPJA8399-21|BIOUG66642-D03|BOLD:ACG0669
Scatopsidae|GMPJA7574-21|BIOUG66633-F09|BOLD:ACG0669
Scatopsidae|GMPJA1516-21|BIOUG65671-D06|BOLD:ACG0669
Scatopsidae|GMPJA7934-21|BIOUG66637-E01|BOLD:ACG0669
Scatopsidae|GMPJA8319-21|BIOUG66641-E06|BOLD:ACG0669
Scatopsidae|GMPJA7426-21|BIOUG66632-B04|BOLD:ACG0669
Scatopsidae|GMPJA2517-21|BIOUG65681-H09|BOLD:ACG0669
Scatopsidae|GMPJA10619-21|BIOUG70236-E05|BOLD:ACG0669
Scatopsidae|GMPJA8269-21|BIOUG66641-A04|BOLD:ACG0669
Scatopsidae|GMPJA10622-21|BIOUG70236-E08|BOLD:ACG0669
Scatopsidae|GMPJA10598-21|BIOUG70236-C08|BOLD:ACG0669
Scatopsidae|GMPJA6286-21|BIOUG66620-B04|BOLD:ACG0669
Scatopsidae|GMPJA1655-21|BIOUG65672-H02|BOLD:ACG0669
Scatopsidae|GMPJA2260-21|BIOUG65679-C02|BOLD:ACG0669
Scatopsidae|GMPJA8292-21|BIOUG66641-C03|BOLD:ACG0669
Scatopsidae|GMPJA8198-21|BIOUG66640-C04|BOLD:ACG0669
Scatopsidae|GMPJA7878-21|BIOUG66636-H04|BOLD:ACG0669
Scatopsidae|GMPJA7519-21|BIOUG66633-B02|BOLD:ACG0669
Scatopsidae|GMPJA5503-21|BIOUG66611-H04|BOLD:ACG0669
Scatopsidae|GMPJA5317-21|BIOUG66609-H08|BOLD:ACG0669
Scatopsidae|GMPJA4470-21|BIOUG66602-A05|BOLD:ACG0669
Scatopsidae|GMPJA8760-21|BIOUG66646-B08|BOLD:ACG0669
Scatopsidae|GMPJA8498-21|BIOUG66643-D07|BOLD:ACG0669
Scatopsidae|GMPJA8669-21|BIOUG66645-B12|BOLD:ACG0669
Scatopsidae|GMPJA2130-21|BIOUG65677-H02|BOLD:ACG0669
Scatopsidae|GMPJA8323-21|BIOUG66641-E10|BOLD:ACG0669
Scatopsidae|GMPJA6825-21|BIOUG66625-G08|BOLD:ACG0669
Scatopsidae|GMPJA6776-21|BIOUG66625-C07|BOLD:ACG0669
Scatopsidae|GMPJA6332-21|BIOUG66620-F02|BOLD:ACG0669
Scatopsidae|GMPJA7371-21|BIOUG66631-E08|BOLD:ACG0669
Scatopsidae|GMPJA7498-21|BIOUG66632-H04|BOLD:ACG0669
Scatopsidae|GMPJA6859-21|BIOUG66626-B07|BOLD:ACG0669
Scatopsidae|GMPJA8473-21|BIOUG66643-B06|BOLD:ACG0669
Scatopsidae|GMPJA8677-21|BIOUG66645-C08|BOLD:ACG0669
Scatopsidae|GMPJA7746-21|BIOUG66635-E03|BOLD:ACG0669
Scatopsidae|GMPJA7870-21|BIOUG66636-G08|BOLD:ACG0669
Scatopsidae|GMPJA7960-21|BIOUG66637-G03|BOLD:ACG0669
Scatopsidae|GMPJA8579-21|BIOUG66644-C05|BOLD:ACG0669
Scatopsidae|GMPJA10550-21|BIOUG70235-G07|BOLD:ACG0669
Scatopsidae|GMPJA10568-21|BIOUG70236-A02|BOLD:ACG0669
Scatopsidae|GMPJA10655-21|BIOUG70236-H05|BOLD:ACG0669
Scatopsidae|GMPJA10557-21|BIOUG70235-H02|BOLD:ACG0669
Scatopsidae|GMPJA5457-21|BIOUG66611-D06|BOLD:ACG0669
Scatopsidae|GMPJA5335-21|BIOUG66610-B03|BOLD:ACG0669
Scatopsidae|GMPJA4041-21|BIOUG65697-E03|BOLD:ACG0669
Scatopsidae|GMPJA10537-21|BIOUG70235-F06|BOLD:ACG0669
Scatopsidae|GMPJA8672-21|BIOUG66645-C03|BOLD:ACG0669
Scatopsidae|GMPJA7711-21|BIOUG66635-B04|BOLD:ACG0669
Scatopsidae|GMPJA5469-21|BIOUG66611-E06|BOLD:ACG0669
Scatopsidae|GMPJA7968-21|BIOUG66637-G11|BOLD:ACG0669
Scatopsidae|GMPJA7634-21|BIOUG66634-C10|BOLD:ACG0669
Scatopsidae|GMPJA1314-21|BIOUG65669-C06|BOLD:ACG0669
Scatopsidae|GMPJA3645-21|BIOUG65693-C11|BOLD:ACG0669
Scatopsidae|GMPJA8358-21|BIOUG66641-H09|BOLD:ACG0669
Scatopsidae|GMPJA7457-21|BIOUG66632-D11|BOLD:ACG0669
Scatopsidae|GMPJA8491-21|BIOUG66643-C12|BOLD:ACG0669
Scatopsidae|GMPJA6608-21|BIOUG66623-E05|BOLD:ACG0669
Scatopsidae|GMPJA7234-21|BIOUG66630-B02|BOLD:ACG0669
Scatopsidae|GMPJA7483-21|BIOUG66632-G01|BOLD:ACG0669
Scatopsidae|GMPJA7530-21|BIOUG66633-C01|BOLD:ACG0669
Scatopsidae|GMPJA8062-21|BIOUG66638-G10|BOLD:ACG0669
Scatopsidae|GMPJA7786-21|BIOUG66635-H07|BOLD:ACG0669
Scatopsidae|GMPJA8119-21|BIOUG66639-D08|BOLD:ACG0669
Scatopsidae|GMPJA8596-21|BIOUG66644-D10|BOLD:ACG0669
Scatopsidae|GMPJA8458-21|BIOUG66643-A03|BOLD:ACG0669
Scatopsidae|GMPJA8809-21|BIOUG66646-F09|BOLD:ACG0669
Scatopsidae|GMPJA10652-21|BIOUG70236-H02|BOLD:ACG0669
Scatopsidae|GMPJA10664-21|BIOUG70237-A03|BOLD:ACG0669
Scatopsidae|GMPJA4256-21|BIOUG65699-G04|BOLD:ACG0669
Scatopsidae|GMPJA1304-21|BIOUG65669-B08|BOLD:ACG0669
[illegible]

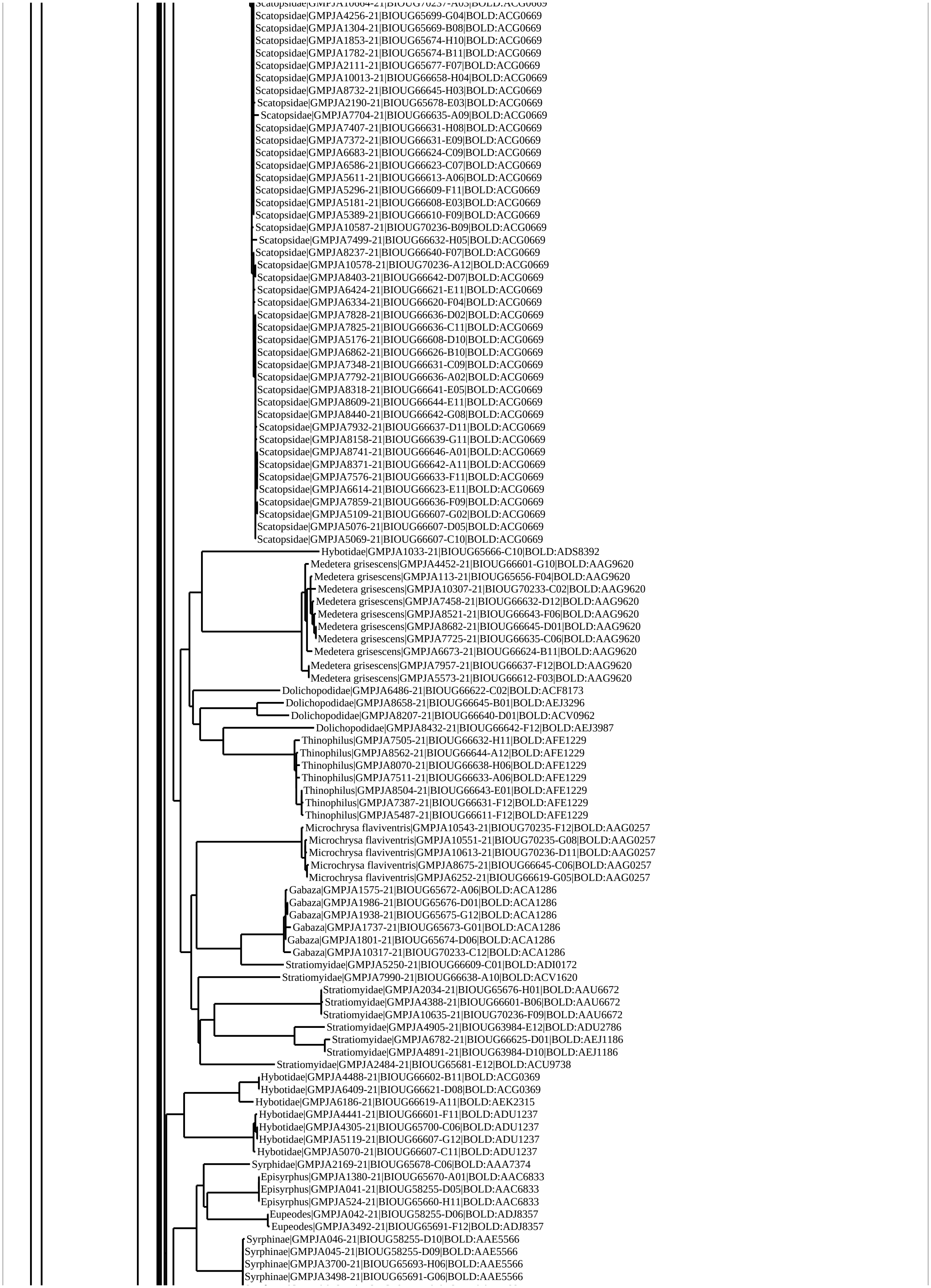
Scatopsidae|GMPJA10664-21|BIOUG70237-A03|BOLD:ACG0669
Scatopsidae|GMPJA4256-21|BIOUG65699-G04|BOLD:ACG0669
Scatopsidae|GMPJA1304-21|BIOUG65669-B08|BOLD:ACG0669
Scatopsidae|GMPJA1853-21|BIOUG65674-H10|BOLD:ACG0669
Scatopsidae|GMPJA1782-21|BIOUG65674-B11|BOLD:ACG0669
Scatopsidae|GMPJA2111-21|BIOUG65677-F07|BOLD:ACG0669
Scatopsidae|GMPJA10013-21|BIOUG66658-H04|BOLD:ACG0669
Scatopsidae|GMPJA8732-21|BIOUG66645-H03|BOLD:ACG0669
Scatopsidae|GMPJA2190-21|BIOUG65678-E03|BOLD:ACG0669
Scatopsidae|GMPJA7704-21|BIOUG66635-A09|BOLD:ACG0669
Scatopsidae|GMPJA7407-21|BIOUG66631-H08|BOLD:ACG0669
Scatopsidae|GMPJA7372-21|BIOUG66631-E09|BOLD:ACG0669
Scatopsidae|GMPJA6683-21|BIOUG66624-C09|BOLD:ACG0669
Scatopsidae|GMPJA6586-21|BIOUG66623-C07|BOLD:ACG0669
Scatopsidae|GMPJA5611-21|BIOUG66613-A06|BOLD:ACG0669
Scatopsidae|GMPJA5296-21|BIOUG66609-F11|BOLD:ACG0669
Scatopsidae|GMPJA5181-21|BIOUG66608-E03|BOLD:ACG0669
Scatopsidae|GMPJA5389-21|BIOUG66610-F09|BOLD:ACG0669
Scatopsidae|GMPJA10587-21|BIOUG70236-B09|BOLD:ACG0669
Scatopsidae|GMPJA7499-21|BIOUG66632-H05|BOLD:ACG0669
Scatopsidae|GMPJA8237-21|BIOUG66640-F07|BOLD:ACG0669
Scatopsidae|GMPJA10578-21|BIOUG70236-A12|BOLD:ACG0669
Scatopsidae|GMPJA8403-21|BIOUG66642-D07|BOLD:ACG0669
Scatopsidae|GMPJA6424-21|BIOUG66621-E11|BOLD:ACG0669
Scatopsidae|GMPJA6334-21|BIOUG66620-F04|BOLD:ACG0669
Scatopsidae|GMPJA7828-21|BIOUG66636-D02|BOLD:ACG0669
Scatopsidae|GMPJA7825-21|BIOUG66636-C11|BOLD:ACG0669
Scatopsidae|GMPJA5176-21|BIOUG66608-D10|BOLD:ACG0669
Scatopsidae|GMPJA6862-21|BIOUG66626-B10|BOLD:ACG0669
Scatopsidae|GMPJA7348-21|BIOUG66631-C09|BOLD:ACG0669
Scatopsidae|GMPJA7792-21|BIOUG66636-A02|BOLD:ACG0669
Scatopsidae|GMPJA8318-21|BIOUG66641-E05|BOLD:ACG0669
Scatopsidae|GMPJA8609-21|BIOUG66644-E11|BOLD:ACG0669
Scatopsidae|GMPJA8440-21|BIOUG66642-G08|BOLD:ACG0669
Scatopsidae|GMPJA7932-21|BIOUG66637-D11|BOLD:ACG0669
Scatopsidae|GMPJA8158-21|BIOUG66639-G11|BOLD:ACG0669
Scatopsidae|GMPJA8741-21|BIOUG66646-A01|BOLD:ACG0669
Scatopsidae|GMPJA8371-21|BIOUG66642-A11|BOLD:ACG0669
Scatopsidae|GMPJA7576-21|BIOUG66633-F11|BOLD:ACG0669
Scatopsidae|GMPJA6614-21|BIOUG66623-E11|BOLD:ACG0669
Scatopsidae|GMPJA7859-21|BIOUG66636-F09|BOLD:ACG0669
Scatopsidae|GMPJA5109-21|BIOUG66607-G02|BOLD:ACG0669
Scatopsidae|GMPJA5076-21|BIOUG66607-D05|BOLD:ACG0669
Scatopsidae|GMPJA5069-21|BIOUG66607-C10|BOLD:ACG0669
Hybotidae|GMPJA1033-21|BIOUG65666-C10|BOLD:ADS8392
Medetera grisescens|GMPJA4452-21|BIOUG66601-G10|BOLD:AAG9620
Medetera grisescens|GMPJA113-21|BIOUG65656-F04|BOLD:AAG9620
Medetera grisescens|GMPJA10307-21|BIOUG70233-C02|BOLD:AAG9620
Medetera grisescens|GMPJA7458-21|BIOUG66632-D12|BOLD:AAG9620
Medetera grisescens|GMPJA8521-21|BIOUG66643-F06|BOLD:AAG9620
Medetera grisescens|GMPJA8682-21|BIOUG66645-D01|BOLD:AAG9620
Medetera grisescens|GMPJA7725-21|BIOUG66635-C06|BOLD:AAG9620
Medetera grisescens|GMPJA6673-21|BIOUG66624-B11|BOLD:AAG9620
Medetera grisescens|GMPJA7957-21|BIOUG66637-F12|BOLD:AAG9620
Medetera grisescens|GMPJA5573-21|BIOUG66612-F03|BOLD:AAG9620
Dolichopodidae|GMPJA6486-21|BIOUG66622-C02|BOLD:ACF8173
Dolichopodidae|GMPJA8658-21|BIOUG66645-B01|BOLD:AEJ3296
Dolichopodidae|GMPJA8207-21|BIOUG66640-D01|BOLD:ACV0962
Dolichopodidae|GMPJA8432-21|BIOUG66642-F12|BOLD:AEJ3987
Thinophilus|GMPJA7505-21|BIOUG66632-H11|BOLD:AFE1229
Thinophilus|GMPJA8562-21|BIOUG66644-A12|BOLD:AFE1229
Thinophilus|GMPJA8070-21|BIOUG66638-H06|BOLD:AFE1229
Thinophilus|GMPJA7511-21|BIOUG66633-A06|BOLD:AFE1229
Thinophilus|GMPJA8504-21|BIOUG66643-E01|BOLD:AFE1229
Thinophilus|GMPJA7387-21|BIOUG66631-F12|BOLD:AFE1229
Thinophilus|GMPJA5487-21|BIOUG66611-F12|BOLD:AFE1229
Microchrysa flaviventris|GMPJA10543-21|BIOUG70235-F12|BOLD:AAG0257
Microchrysa flaviventris|GMPJA10551-21|BIOUG70235-G08|BOLD:AAG0257
Microchrysa flaviventris|GMPJA10613-21|BIOUG70236-D11|BOLD:AAG0257
Microchrysa flaviventris|GMPJA8675-21|BIOUG66645-C06|BOLD:AAG0257
Microchrysa flaviventris|GMPJA6252-21|BIOUG66619-G05|BOLD:AAG0257
Gabaza|GMPJA1575-21|BIOUG65672-A06|BOLD:ACA1286
Gabaza|GMPJA1986-21|BIOUG65676-D01|BOLD:ACA1286
Gabaza|GMPJA1938-21|BIOUG65675-G12|BOLD:ACA1286
Gabaza|GMPJA1737-21|BIOUG65673-G01|BOLD:ACA1286
Gabaza|GMPJA1801-21|BIOUG65674-D06|BOLD:ACA1286
Gabaza|GMPJA10317-21|BIOUG70233-C12|BOLD:ACA1286
Stratiomyidae|GMPJA5250-21|BIOUG66609-C01|BOLD:ADI0172
Stratiomyidae|GMPJA7990-21|BIOUG66638-A10|BOLD:ACV1620
Stratiomyidae|GMPJA2034-21|BIOUG65676-H01|BOLD:AAU6672
Stratiomyidae|GMPJA4388-21|BIOUG66601-B06|BOLD:AAU6672
Stratiomyidae|GMPJA10635-21|BIOUG70236-F09|BOLD:AAU6672
Stratiomyidae|GMPJA4905-21|BIOUG63984-E12|BOLD:ADU2786
Stratiomyidae|GMPJA6782-21|BIOUG66625-D01|BOLD:AEJ1186
Stratiomyidae|GMPJA4891-21|BIOUG63984-D10|BOLD:AEJ1186
Stratiomyidae|GMPJA2484-21|BIOUG65681-E12|BOLD:ACU9738
Hybotidae|GMPJA4488-21|BIOUG66602-B11|BOLD:ACG0369
Hybotidae|GMPJA6409-21|BIOUG66621-D08|BOLD:ACG0369
Hybotidae|GMPJA6186-21|BIOUG66619-A11|BOLD:AEK2315
Hybotidae|GMPJA4441-21|BIOUG66601-F11|BOLD:ADU1237
Hybotidae|GMPJA4305-21|BIOUG65700-C06|BOLD:ADU1237
Hybotidae|GMPJA5119-21|BIOUG66607-G12|BOLD:ADU1237
Hybotidae|GMPJA5070-21|BIOUG66607-C11|BOLD:ADU1237
Syrphidae|GMPJA2169-21|BIOUG65678-C06|BOLD:AAA7374
Episyrphus|GMPJA1380-21|BIOUG65670-A01|BOLD:AAC6833
Episyrphus|GMPJA041-21|BIOUG58255-D05|BOLD:AAC6833
Episyrphus|GMPJA524-21|BIOUG65660-H11|BOLD:AAC6833
Eupeodes|GMPJA042-21|BIOUG58255-D06|BOLD:ADJ8357
Eupeodes|GMPJA3492-21|BIOUG65691-F12|BOLD:ADJ8357
Syrphinae|GMPJA046-21|BIOUG58255-D10|BOLD:AAE5566
Syrphinae|GMPJA045-21|BIOUG58255-D09|BOLD:AAE5566
Syrphinae|GMPJA3700-21|BIOUG65693-H06|BOLD:AAE5566
Syrphinae|GMPJA3498-21|BIOUG65691-G06|BOLD:AAE5566

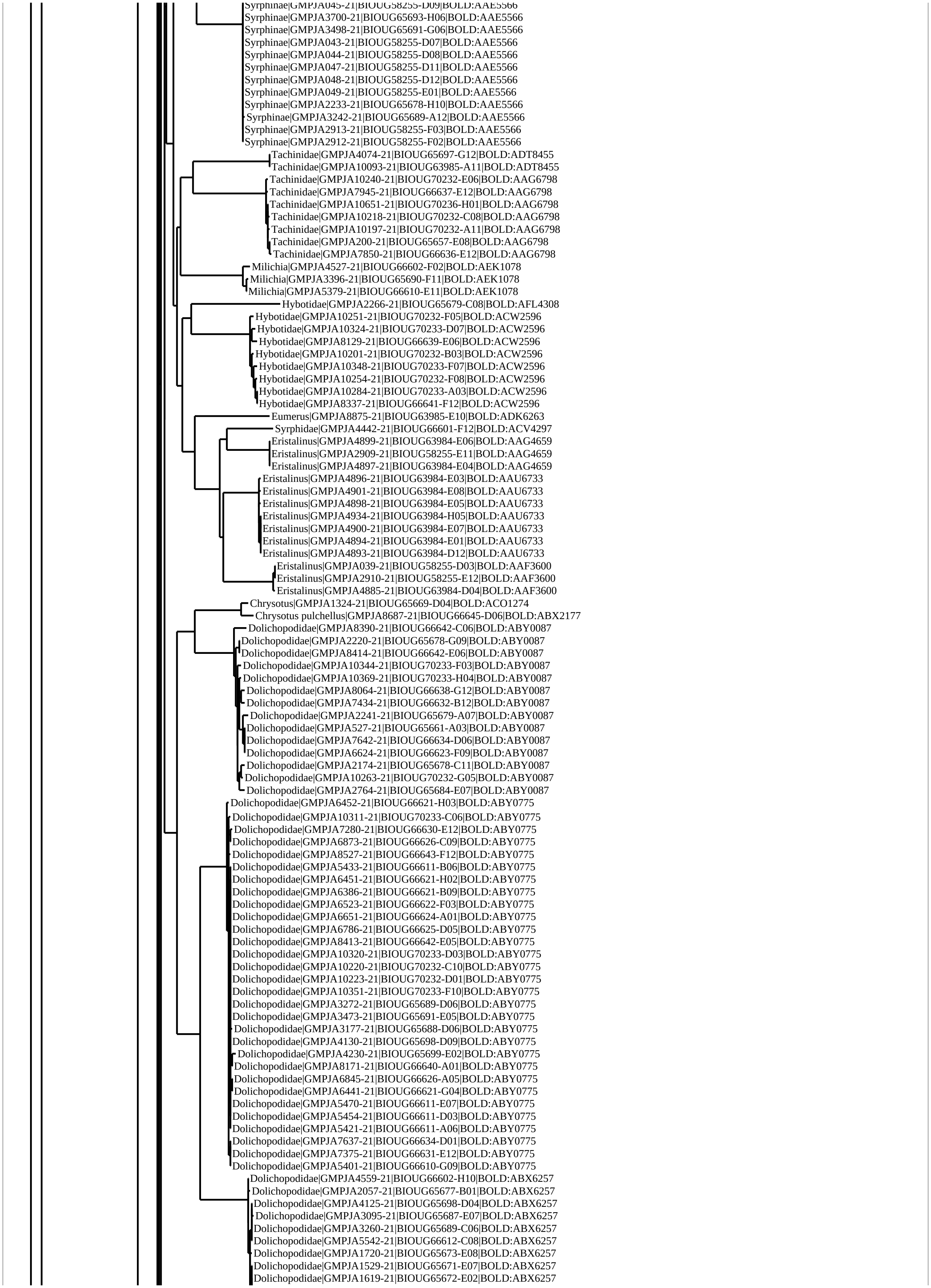
Syrphinae|GMPJA045-21|BIOUG58255-D09|BOLD:AAE5566
Syrphinae|GMPJA3700-21|BIOUG65693-H06|BOLD:AAE5566
Syrphinae|GMPJA3498-21|BIOUG65691-G06|BOLD:AAE5566
Syrphinae|GMPJA043-21|BIOUG58255-D07|BOLD:AAE5566
Syrphinae|GMPJA044-21|BIOUG58255-D08|BOLD:AAE5566
Syrphinae|GMPJA047-21|BIOUG58255-D11|BOLD:AAE5566
Syrphinae|GMPJA048-21|BIOUG58255-D12|BOLD:AAE5566
Syrphinae|GMPJA049-21|BIOUG58255-E01|BOLD:AAE5566
Syrphinae|GMPJA2233-21|BIOUG65678-H10|BOLD:AAE5566
Syrphinae|GMPJA3242-21|BIOUG65689-A12|BOLD:AAE5566
Syrphinae|GMPJA2913-21|BIOUG58255-F03|BOLD:AAE5566
Syrphinae|GMPJA2912-21|BIOUG58255-F02|BOLD:AAE5566
Tachinidae|GMPJA4074-21|BIOUG65697-G12|BOLD:ADT8455
Tachinidae|GMPJA10093-21|BIOUG63985-A11|BOLD:ADT8455
Tachinidae|GMPJA10240-21|BIOUG70232-E06|BOLD:AAG6798
Tachinidae|GMPJA7945-21|BIOUG66637-E12|BOLD:AAG6798
Tachinidae|GMPJA10651-21|BIOUG70236-H01|BOLD:AAG6798
Tachinidae|GMPJA10218-21|BIOUG70232-C08|BOLD:AAG6798
Tachinidae|GMPJA10197-21|BIOUG70232-A11|BOLD:AAG6798
Tachinidae|GMPJA200-21|BIOUG65657-E08|BOLD:AAG6798
Tachinidae|GMPJA7850-21|BIOUG66636-E12|BOLD:AAG6798
Milichia|GMPJA4527-21|BIOUG66602-F02|BOLD:AEK1078
Milichia|GMPJA3396-21|BIOUG65690-F11|BOLD:AEK1078
Milichia|GMPJA5379-21|BIOUG66610-E11|BOLD:AEK1078
Hybotidae|GMPJA2266-21|BIOUG65679-C08|BOLD:AFL4308
Hybotidae|GMPJA10251-21|BIOUG70232-F05|BOLD:ACW2596
Hybotidae|GMPJA10324-21|BIOUG70233-D07|BOLD:ACW2596
Hybotidae|GMPJA8129-21|BIOUG66639-E06|BOLD:ACW2596
Hybotidae|GMPJA10201-21|BIOUG70232-B03|BOLD:ACW2596
Hybotidae|GMPJA10348-21|BIOUG70233-F07|BOLD:ACW2596
Hybotidae|GMPJA10254-21|BIOUG70232-F08|BOLD:ACW2596
Hybotidae|GMPJA10284-21|BIOUG70233-A03|BOLD:ACW2596
Hybotidae|GMPJA8337-21|BIOUG66641-F12|BOLD:ACW2596
Eumerus|GMPJA8875-21|BIOUG63985-E10|BOLD:ADK6263
Syrphidae|GMPJA4442-21|BIOUG66601-F12|BOLD:ACV4297
Eristalinus|GMPJA4899-21|BIOUG63984-E06|BOLD:AAG4659
Eristalinus|GMPJA2909-21|BIOUG58255-E11|BOLD:AAG4659
Eristalinus|GMPJA4897-21|BIOUG63984-E04|BOLD:AAG4659
Eristalinus|GMPJA4896-21|BIOUG63984-E03|BOLD:AAU6733
Eristalinus|GMPJA4901-21|BIOUG63984-E08|BOLD:AAU6733
Eristalinus|GMPJA4898-21|BIOUG63984-E05|BOLD:AAU6733
Eristalinus|GMPJA4934-21|BIOUG63984-H05|BOLD:AAU6733
Eristalinus|GMPJA4900-21|BIOUG63984-E07|BOLD:AAU6733
Eristalinus|GMPJA4894-21|BIOUG63984-E01|BOLD:AAU6733
Eristalinus|GMPJA4893-21|BIOUG63984-D12|BOLD:AAU6733
Eristalinus|GMPJA039-21|BIOUG58255-D03|BOLD:AAF3600
Eristalinus|GMPJA2910-21|BIOUG58255-E12|BOLD:AAF3600
Eristalinus|GMPJA4885-21|BIOUG63984-D04|BOLD:AAF3600
Chrysotus|GMPJA1324-21|BIOUG65669-D04|BOLD:ACO1274
Chrysotus pulchellus|GMPJA8687-21|BIOUG66645-D06|BOLD:ABX2177
Dolichopodidae|GMPJA8390-21|BIOUG66642-C06|BOLD:ABY0087
Dolichopodidae|GMPJA2220-21|BIOUG65678-G09|BOLD:ABY0087
Dolichopodidae|GMPJA8414-21|BIOUG66642-E06|BOLD:ABY0087
Dolichopodidae|GMPJA10344-21|BIOUG70233-F03|BOLD:ABY0087
Dolichopodidae|GMPJA10369-21|BIOUG70233-H04|BOLD:ABY0087
Dolichopodidae|GMPJA8064-21|BIOUG66638-G12|BOLD:ABY0087
Dolichopodidae|GMPJA7434-21|BIOUG66632-B12|BOLD:ABY0087
Dolichopodidae|GMPJA2241-21|BIOUG65679-A07|BOLD:ABY0087
Dolichopodidae|GMPJA527-21|BIOUG65661-A03|BOLD:ABY0087
Dolichopodidae|GMPJA7642-21|BIOUG66634-D06|BOLD:ABY0087
Dolichopodidae|GMPJA6624-21|BIOUG66623-F09|BOLD:ABY0087
Dolichopodidae|GMPJA2174-21|BIOUG65678-C11|BOLD:ABY0087
Dolichopodidae|GMPJA10263-21|BIOUG70232-G05|BOLD:ABY0087
Dolichopodidae|GMPJA2764-21|BIOUG65684-E07|BOLD:ABY0087
Dolichopodidae|GMPJA6452-21|BIOUG66621-H03|BOLD:ABY0775
Dolichopodidae|GMPJA10311-21|BIOUG70233-C06|BOLD:ABY0775
Dolichopodidae|GMPJA7280-21|BIOUG66630-E12|BOLD:ABY0775
Dolichopodidae|GMPJA6873-21|BIOUG66626-C09|BOLD:ABY0775
Dolichopodidae|GMPJA8527-21|BIOUG66643-F12|BOLD:ABY0775
Dolichopodidae|GMPJA5433-21|BIOUG66611-B06|BOLD:ABY0775
Dolichopodidae|GMPJA6451-21|BIOUG66621-H02|BOLD:ABY0775
Dolichopodidae|GMPJA6386-21|BIOUG66621-B09|BOLD:ABY0775
Dolichopodidae|GMPJA6523-21|BIOUG66622-F03|BOLD:ABY0775
Dolichopodidae|GMPJA6651-21|BIOUG66624-A01|BOLD:ABY0775
Dolichopodidae|GMPJA6786-21|BIOUG66625-D05|BOLD:ABY0775
Dolichopodidae|GMPJA8413-21|BIOUG66642-E05|BOLD:ABY0775
Dolichopodidae|GMPJA10320-21|BIOUG70233-D03|BOLD:ABY0775
Dolichopodidae|GMPJA10220-21|BIOUG70232-C10|BOLD:ABY0775
Dolichopodidae|GMPJA10223-21|BIOUG70232-D01|BOLD:ABY0775
Dolichopodidae|GMPJA10351-21|BIOUG70233-F10|BOLD:ABY0775
Dolichopodidae|GMPJA3272-21|BIOUG65689-D06|BOLD:ABY0775
Dolichopodidae|GMPJA3473-21|BIOUG65691-E05|BOLD:ABY0775
Dolichopodidae|GMPJA3177-21|BIOUG65688-D06|BOLD:ABY0775
Dolichopodidae|GMPJA4130-21|BIOUG65698-D09|BOLD:ABY0775
Dolichopodidae|GMPJA4230-21|BIOUG65699-E02|BOLD:ABY0775
Dolichopodidae|GMPJA8171-21|BIOUG66640-A01|BOLD:ABY0775
Dolichopodidae|GMPJA6845-21|BIOUG66626-A05|BOLD:ABY0775
Dolichopodidae|GMPJA6441-21|BIOUG66621-G04|BOLD:ABY0775
Dolichopodidae|GMPJA5470-21|BIOUG66611-E07|BOLD:ABY0775
Dolichopodidae|GMPJA5454-21|BIOUG66611-D03|BOLD:ABY0775
Dolichopodidae|GMPJA5421-21|BIOUG66611-A06|BOLD:ABY0775
Dolichopodidae|GMPJA7637-21|BIOUG66634-D01|BOLD:ABY0775
Dolichopodidae|GMPJA7375-21|BIOUG66631-E12|BOLD:ABY0775
Dolichopodidae|GMPJA5401-21|BIOUG66610-G09|BOLD:ABY0775
Dolichopodidae|GMPJA4559-21|BIOUG66602-H10|BOLD:ABX6257
Dolichopodidae|GMPJA2057-21|BIOUG65677-B01|BOLD:ABX6257
Dolichopodidae|GMPJA4125-21|BIOUG65698-D04|BOLD:ABX6257
Dolichopodidae|GMPJA3095-21|BIOUG65687-E07|BOLD:ABX6257
Dolichopodidae|GMPJA3260-21|BIOUG65689-C06|BOLD:ABX6257
Dolichopodidae|GMPJA5542-21|BIOUG66612-C08|BOLD:ABX6257
Dolichopodidae|GMPJA1720-21|BIOUG65673-E08|BOLD:ABX6257
Dolichopodidae|GMPJA1529-21|BIOUG65671-E07|BOLD:ABX6257
Dolichopodidae|GMPJA1619-21|BIOUG65672-E02|BOLD:ABX6257

Dolichopodidae|GMPJA1720-21|BIOUG65673-E08|BOLD:ABX6257
Dolichopodidae|GMPJA1529-21|BIOUG65671-E07|BOLD:ABX6257
Dolichopodidae|GMPJA1619-21|BIOUG65672-E02|BOLD:ABX6257
Dolichopodidae|GMPJA2346-21|BIOUG65680-B05|BOLD:ABX6257
Dolichopodidae|GMPJA4553-21|BIOUG66602-H04|BOLD:ABX6257
Dolichopodidae|GMPJA4216-21|BIOUG65699-C12|BOLD:ABX6257
Dolichopodidae|GMPJA4451-21|BIOUG66601-G09|BOLD:ABX6257
Dolichopodidae|GMPJA5297-21|BIOUG66609-F12|BOLD:ABX6257
Dolichopodidae|GMPJA10224-21|BIOUG70232-D02|BOLD:ABX6257
Dolichopodidae|GMPJA4364-21|BIOUG65700-H05|BOLD:ABX6257
Dolichopodidae|GMPJA5553-21|BIOUG66612-D07|BOLD:ABX6257
Dolichopodidae|GMPJA5535-21|BIOUG66612-C01|BOLD:ABX6257
Dolichopodidae|GMPJA5224-21|BIOUG66608-H10|BOLD:ABX6257
Dolichopodidae|GMPJA5054-21|BIOUG66607-B07|BOLD:ABX6257
Dolichopodidae|GMPJA6682-21|BIOUG66624-C08|BOLD:ABX6257
Dolichopodidae|GMPJA6851-21|BIOUG66626-A11|BOLD:ABX6257
Dolichopodidae|GMPJA10582-21|BIOUG70236-B04|BOLD:ABX6257
Dolichopodidae|GMPJA3066-21|BIOUG65687-C02|BOLD:ABX6257
Dolichopodidae|GMPJA3267-21|BIOUG65689-D01|BOLD:ABX6257
Dolichopodidae|GMPJA4313-21|BIOUG65700-D02|BOLD:ABX6257
Dolichopodidae|GMPJA4409-21|BIOUG66601-D03|BOLD:ABX6257
Dolichopodidae|GMPJA3526-21|BIOUG65692-A11|BOLD:ABX6257
Dolichopodidae|GMPJA3575-21|BIOUG65692-E12|BOLD:ABX6257
Dolichopodidae|GMPJA3605-21|BIOUG65692-H06|BOLD:ABX6257
Dolichopodidae|GMPJA1474-21|BIOUG65670-H11|BOLD:ABX6257
Dolichopodidae|GMPJA1966-21|BIOUG65676-B05|BOLD:ABX6257
Dolichopodidae|GMPJA1985-21|BIOUG65676-C12|BOLD:ABX6257
Dolichopodidae|GMPJA2011-21|BIOUG65676-F02|BOLD:ABX6257
Dolichopodidae|GMPJA2032-21|BIOUG65676-G11|BOLD:ABX6257
Dolichopodidae|GMPJA1753-21|BIOUG65673-H05|BOLD:ABX6257
Dolichopodidae|GMPJA6534-21|BIOUG66622-G02|BOLD:ABX6257
Dolichopodidae|GMPJA5591-21|BIOUG66612-G09|BOLD:ABX6257
Dolichopodidae|GMPJA2478-21|BIOUG65681-E06|BOLD:ABX6257
Phoridae|GMPJA3369-21|BIOUG65690-D08|BOLD:ACV8100
Phoridae|GMPJA6467-21|BIOUG66622-A07|BOLD:ACP4519
Phoridae|GMPJA3155-21|BIOUG65688-B08|BOLD:ACP3822
Phoridae|GMPJA6706-21|BIOUG66624-E08|BOLD:ACP3822
Phoridae|GMPJA3073-21|BIOUG65687-C09|BOLD:AEJ8817
Megaselia albicans|GMPJA5456-21|BIOUG66611-D05|BOLD:ACF7689
Megaselia albicans|GMPJA5610-21|BIOUG66613-A05|BOLD:ACF7689
Megaselia albicans|GMPJA5550-21|BIOUG66612-D04|BOLD:ACF7689
Megaselia albicans|GMPJA3265-21|BIOUG65689-C11|BOLD:ACF7689
Megaselia albicans|GMPJA7958-21|BIOUG66637-G01|BOLD:ACF7689
Megaselia albicans|GMPJA4308-21|BIOUG65700-C09|BOLD:ACF7689
Megaselia albicans|GMPJA5232-21|BIOUG66609-A07|BOLD:ACF7689
Megaselia albicans|GMPJA4168-21|BIOUG65698-G11|BOLD:ACF7689
Megaselia albicans|GMPJA4551-21|BIOUG66602-H02|BOLD:ACF7689
Megaselia albicans|GMPJA5306-21|BIOUG66609-G09|BOLD:ACF7689
Megaselia albicans|GMPJA5100-21|BIOUG66607-F05|BOLD:ACF7689
Megaselia albicans|GMPJA4094-21|BIOUG65698-A09|BOLD:ACF7689
Megaselia albicans|GMPJA948-21|BIOUG65665-D08|BOLD:ACF7689
Megaselia albicans|GMPJA641-21|BIOUG65662-B10|BOLD:ACF7689
Megaselia albicans|GMPJA5593-21|BIOUG66612-G11|BOLD:ACF7689
Megaselia albicans|GMPJA5305-21|BIOUG66609-G08|BOLD:ACF7689
Megaselia albicans|GMPJA5431-21|BIOUG66611-B04|BOLD:ACF7689
Megaselia albicans|GMPJA5324-21|BIOUG66610-A04|BOLD:ACF7689
Megaselia albicans|GMPJA4547-21|BIOUG66602-G10|BOLD:ACF7689
Megaselia albicans|GMPJA3392-21|BIOUG65690-F07|BOLD:ACF7689
Megaselia albicans|GMPJA5397-21|BIOUG66610-G05|BOLD:ACF7689
Megaselia albicans|GMPJA7469-21|BIOUG66632-E11|BOLD:ACF7689
Megaselia albicans|GMPJA5260-21|BIOUG66609-C11|BOLD:ACF7689
Megaselia albicans|GMPJA5437-21|BIOUG66611-B10|BOLD:ACF7689
Megaselia albicans|GMPJA5504-21|BIOUG66611-H05|BOLD:ACF7689
Megaselia albicans|GMPJA5442-21|BIOUG66611-C03|BOLD:ACF7689
Megaselia albicans|GMPJA5585-21|BIOUG66612-G03|BOLD:ACF7689
Megaselia albicans|GMPJA4225-21|BIOUG65699-D09|BOLD:ACF7689
Megaselia albicans|GMPJA4411-21|BIOUG66601-D05|BOLD:ACF7689
Megaselia albicans|GMPJA3170-21|BIOUG65688-C11|BOLD:ACF7689
Megaselia albicans|GMPJA5426-21|BIOUG66611-A11|BOLD:ACF7689
Megaselia albicans|GMPJA5364-21|BIOUG66610-D08|BOLD:ACF7689
Megaselia albicans|GMPJA5355-21|BIOUG66610-C11|BOLD:ACF7689
Megaselia albicans|GMPJA5575-21|BIOUG66612-F05|BOLD:ACF7689
Megaselia albicans|GMPJA5491-21|BIOUG66611-G04|BOLD:ACF7689
Megaselia albicans|GMPJA5446-21|BIOUG66611-C07|BOLD:ACF7689
Megaselia albicans|GMPJA4207-21|BIOUG65699-C03|BOLD:ACF7689
Megaselia albicans|GMPJA5425-21|BIOUG66611-A10|BOLD:ACF7689
Megaselia albicans|GMPJA2354-21|BIOUG65680-C01|BOLD:ACF7689
Megaselia albicans|GMPJA10542-21|BIOUG70235-F11|BOLD:ACF7689
Megaselia albicans|GMPJA5438-21|BIOUG66611-B11|BOLD:ACF7689
Megaselia albicans|GMPJA5468-21|BIOUG66611-E05|BOLD:ACF7689
Megaselia albicans|GMPJA3229-21|BIOUG65688-H10|BOLD:ACF7689
Megaselia albicans|GMPJA5383-21|BIOUG66610-F03|BOLD:ACF7689
Megaselia albicans|GMPJA5384-21|BIOUG66610-F04|BOLD:ACF7689
Megaselia albicans|GMPJA5411-21|BIOUG66610-H07|BOLD:ACF7689
Megaselia albicans|GMPJA5448-21|BIOUG66611-C09|BOLD:ACF7689
Megaselia albicans|GMPJA5097-21|BIOUG66607-F02|BOLD:ACF7689
Megaselia albicans|GMPJA5110-21|BIOUG66607-G03|BOLD:ACF7689
Megaselia albicans|GMPJA5138-21|BIOUG66608-A08|BOLD:ACF7689
Megaselia albicans|GMPJA5139-21|BIOUG66608-A09|BOLD:ACF7689
Megaselia albicans|GMPJA5187-21|BIOUG66608-E09|BOLD:ACF7689
Megaselia albicans|GMPJA5193-21|BIOUG66608-F03|BOLD:ACF7689
Megaselia albicans|GMPJA5217-21|BIOUG66608-H03|BOLD:ACF7689
Megaselia albicans|GMPJA5221-21|BIOUG66608-H07|BOLD:ACF7689
Megaselia albicans|GMPJA5239-21|BIOUG66609-B02|BOLD:ACF7689
Megaselia albicans|GMPJA5281-21|BIOUG66609-E08|BOLD:ACF7689
Megaselia albicans|GMPJA5290-21|BIOUG66609-F05|BOLD:ACF7689
Megaselia albicans|GMPJA5555-21|BIOUG66612-D09|BOLD:ACF7689
Megaselia albicans|GMPJA5597-21|BIOUG66612-H03|BOLD:ACF7689
Megaselia albicans|GMPJA4497-21|BIOUG66602-C08|BOLD:ACF7689
Megaselia albicans|GMPJA10610-21|BIOUG70236-D08|BOLD:ACF7689
Megaselia albicans|GMPJA4202-21|BIOUG65699-B10|BOLD:ACF7689
Megaselia albicans|GMPJA4244-21|BIOUG65699-F04|BOLD:ACF7689
Megaselia albicans|GMPJA4375-21|BIOUG66601-A05|BOLD:ACF7689

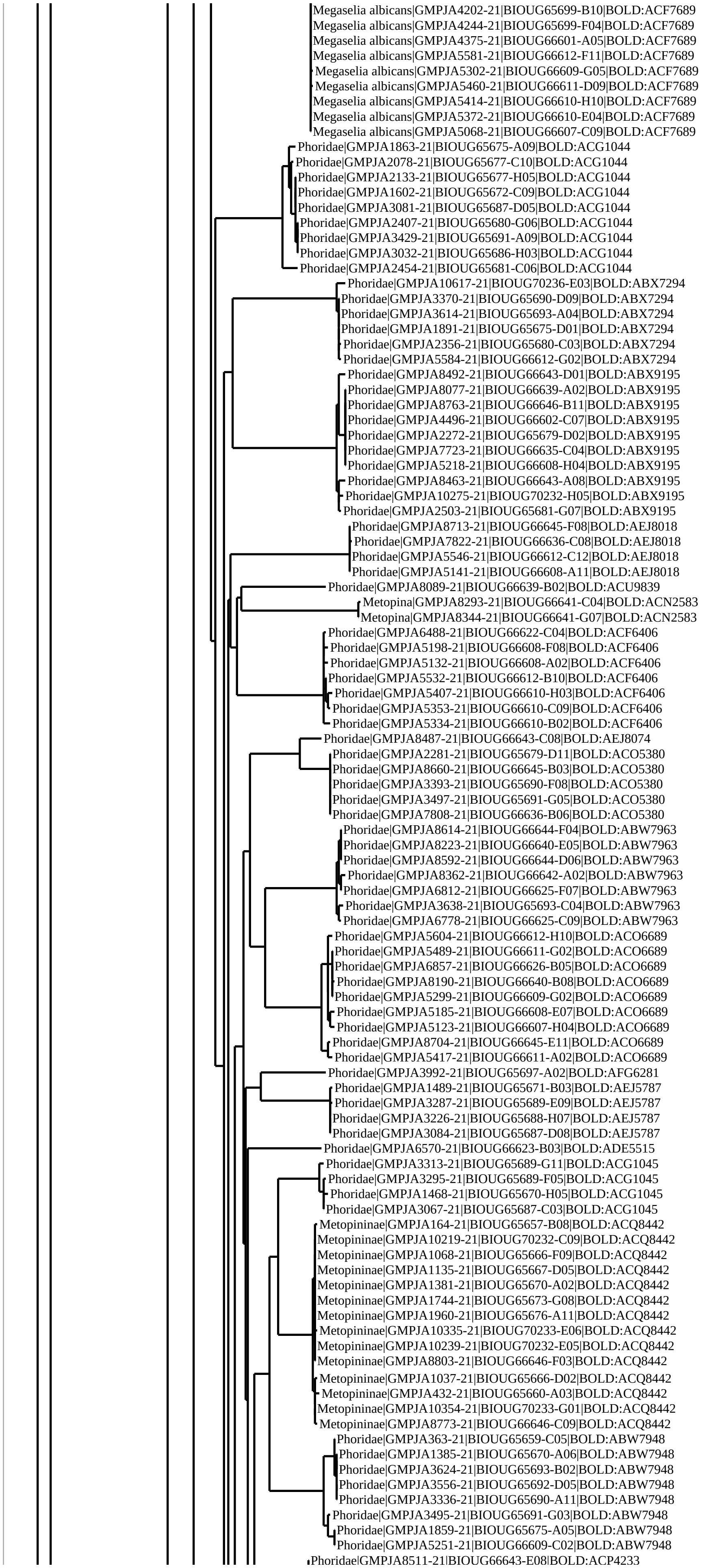

Megaselia albicans|GMPJA4202-21|BIOUG65699-B10|BOLD:ACF7689
Megaselia albicans|GMPJA4244-21|BIOUG65699-F04|BOLD:ACF7689
Megaselia albicans|GMPJA4375-21|BIOUG66601-A05|BOLD:ACF7689
Megaselia albicans|GMPJA5581-21|BIOUG66612-F11|BOLD:ACF7689
Megaselia albicans|GMPJA5302-21|BIOUG66609-G05|BOLD:ACF7689
Megaselia albicans|GMPJA5460-21|BIOUG66611-D09|BOLD:ACF7689
Megaselia albicans|GMPJA5414-21|BIOUG66610-H10|BOLD:ACF7689
Megaselia albicans|GMPJA5372-21|BIOUG66610-E04|BOLD:ACF7689
Megaselia albicans|GMPJA5068-21|BIOUG66607-C09|BOLD:ACF7689
Phoridae|GMPJA1863-21|BIOUG65675-A09|BOLD:ACG1044
Phoridae|GMPJA2078-21|BIOUG65677-C10|BOLD:ACG1044
Phoridae|GMPJA2133-21|BIOUG65677-H05|BOLD:ACG1044
Phoridae|GMPJA1602-21|BIOUG65672-C09|BOLD:ACG1044
Phoridae|GMPJA3081-21|BIOUG65687-D05|BOLD:ACG1044
Phoridae|GMPJA2407-21|BIOUG65680-G06|BOLD:ACG1044
Phoridae|GMPJA3429-21|BIOUG65691-A09|BOLD:ACG1044
Phoridae|GMPJA3032-21|BIOUG65686-H03|BOLD:ACG1044
Phoridae|GMPJA2454-21|BIOUG65681-C06|BOLD:ACG1044
Phoridae|GMPJA10617-21|BIOUG70236-E03|BOLD:ABX7294
Phoridae|GMPJA3370-21|BIOUG65690-D09|BOLD:ABX7294
Phoridae|GMPJA3614-21|BIOUG65693-A04|BOLD:ABX7294
Phoridae|GMPJA1891-21|BIOUG65675-D01|BOLD:ABX7294
Phoridae|GMPJA2356-21|BIOUG65680-C03|BOLD:ABX7294
Phoridae|GMPJA5584-21|BIOUG66612-G02|BOLD:ABX7294
Phoridae|GMPJA8492-21|BIOUG66643-D01|BOLD:ABX9195
Phoridae|GMPJA8077-21|BIOUG66639-A02|BOLD:ABX9195
Phoridae|GMPJA8763-21|BIOUG66646-B11|BOLD:ABX9195
Phoridae|GMPJA4496-21|BIOUG66602-C07|BOLD:ABX9195
Phoridae|GMPJA2272-21|BIOUG65679-D02|BOLD:ABX9195
Phoridae|GMPJA7723-21|BIOUG66635-C04|BOLD:ABX9195
Phoridae|GMPJA5218-21|BIOUG66608-H04|BOLD:ABX9195
Phoridae|GMPJA8463-21|BIOUG66643-A08|BOLD:ABX9195
Phoridae|GMPJA10275-21|BIOUG70232-H05|BOLD:ABX9195
Phoridae|GMPJA2503-21|BIOUG65681-G07|BOLD:ABX9195
Phoridae|GMPJA8713-21|BIOUG66645-F08|BOLD:AEJ8018
Phoridae|GMPJA7822-21|BIOUG66636-C08|BOLD:AEJ8018
Phoridae|GMPJA5546-21|BIOUG66612-C12|BOLD:AEJ8018
Phoridae|GMPJA5141-21|BIOUG66608-A11|BOLD:AEJ8018
Phoridae|GMPJA8089-21|BIOUG66639-B02|BOLD:ACU9839
Metopina|GMPJA8293-21|BIOUG66641-C04|BOLD:ACN2583
Metopina|GMPJA8344-21|BIOUG66641-G07|BOLD:ACN2583
Phoridae|GMPJA6488-21|BIOUG66622-C04|BOLD:ACF6406
Phoridae|GMPJA5198-21|BIOUG66608-F08|BOLD:ACF6406
Phoridae|GMPJA5132-21|BIOUG66608-A02|BOLD:ACF6406
Phoridae|GMPJA5532-21|BIOUG66612-B10|BOLD:ACF6406
Phoridae|GMPJA5407-21|BIOUG66610-H03|BOLD:ACF6406
Phoridae|GMPJA5353-21|BIOUG66610-C09|BOLD:ACF6406
Phoridae|GMPJA5334-21|BIOUG66610-B02|BOLD:ACF6406
Phoridae|GMPJA8487-21|BIOUG66643-C08|BOLD:AEJ8074
Phoridae|GMPJA2281-21|BIOUG65679-D11|BOLD:ACO5380
Phoridae|GMPJA8660-21|BIOUG66645-B03|BOLD:ACO5380
Phoridae|GMPJA3393-21|BIOUG65690-F08|BOLD:ACO5380
Phoridae|GMPJA3497-21|BIOUG65691-G05|BOLD:ACO5380
Phoridae|GMPJA7808-21|BIOUG66636-B06|BOLD:ACO5380
Phoridae|GMPJA8614-21|BIOUG66644-F04|BOLD:ABW7963
Phoridae|GMPJA8223-21|BIOUG66640-E05|BOLD:ABW7963
Phoridae|GMPJA8592-21|BIOUG66644-D06|BOLD:ABW7963
Phoridae|GMPJA8362-21|BIOUG66642-A02|BOLD:ABW7963
Phoridae|GMPJA6812-21|BIOUG66625-F07|BOLD:ABW7963
Phoridae|GMPJA3638-21|BIOUG65693-C04|BOLD:ABW7963
Phoridae|GMPJA6778-21|BIOUG66625-C09|BOLD:ABW7963
Phoridae|GMPJA5604-21|BIOUG66612-H10|BOLD:ACO6689
Phoridae|GMPJA5489-21|BIOUG66611-G02|BOLD:ACO6689
Phoridae|GMPJA6857-21|BIOUG66626-B05|BOLD:ACO6689
Phoridae|GMPJA8190-21|BIOUG66640-B08|BOLD:ACO6689
Phoridae|GMPJA5299-21|BIOUG66609-G02|BOLD:ACO6689
Phoridae|GMPJA5185-21|BIOUG66608-E07|BOLD:ACO6689
Phoridae|GMPJA5123-21|BIOUG66607-H04|BOLD:ACO6689
Phoridae|GMPJA8704-21|BIOUG66645-E11|BOLD:ACO6689
Phoridae|GMPJA5417-21|BIOUG66611-A02|BOLD:ACO6689
Phoridae|GMPJA3992-21|BIOUG65697-A02|BOLD:AFG6281
Phoridae|GMPJA1489-21|BIOUG65671-B03|BOLD:AEJ5787
Phoridae|GMPJA3287-21|BIOUG65689-E09|BOLD:AEJ5787
Phoridae|GMPJA3226-21|BIOUG65688-H07|BOLD:AEJ5787
Phoridae|GMPJA3084-21|BIOUG65687-D08|BOLD:AEJ5787
Phoridae|GMPJA6570-21|BIOUG66623-B03|BOLD:ADE5515
Phoridae|GMPJA3313-21|BIOUG65689-G11|BOLD:ACG1045
Phoridae|GMPJA3295-21|BIOUG65689-F05|BOLD:ACG1045
Phoridae|GMPJA1468-21|BIOUG65670-H05|BOLD:ACG1045
Phoridae|GMPJA3067-21|BIOUG65687-C03|BOLD:ACG1045
Metopininae|GMPJA164-21|BIOUG65657-B08|BOLD:ACQ8442
Metopininae|GMPJA10219-21|BIOUG70232-C09|BOLD:ACQ8442
Metopininae|GMPJA1068-21|BIOUG65666-F09|BOLD:ACQ8442
Metopininae|GMPJA1135-21|BIOUG65667-D05|BOLD:ACQ8442
Metopininae|GMPJA1381-21|BIOUG65670-A02|BOLD:ACQ8442
Metopininae|GMPJA1744-21|BIOUG65673-G08|BOLD:ACQ8442
Metopininae|GMPJA1960-21|BIOUG65676-A11|BOLD:ACQ8442
Metopininae|GMPJA10335-21|BIOUG70233-E06|BOLD:ACQ8442
Metopininae|GMPJA10239-21|BIOUG70232-E05|BOLD:ACQ8442
Metopininae|GMPJA8803-21|BIOUG66646-F03|BOLD:ACQ8442
Metopininae|GMPJA1037-21|BIOUG65666-D02|BOLD:ACQ8442
Metopininae|GMPJA432-21|BIOUG65660-A03|BOLD:ACQ8442
Metopininae|GMPJA10354-21|BIOUG70233-G01|BOLD:ACQ8442
Metopininae|GMPJA8773-21|BIOUG66646-C09|BOLD:ACQ8442
Phoridae|GMPJA363-21|BIOUG65659-C05|BOLD:ABW7948
Phoridae|GMPJA1385-21|BIOUG65670-A06|BOLD:ABW7948
Phoridae|GMPJA3624-21|BIOUG65693-B02|BOLD:ABW7948
Phoridae|GMPJA3556-21|BIOUG65692-D05|BOLD:ABW7948
Phoridae|GMPJA3336-21|BIOUG65690-A11|BOLD:ABW7948
Phoridae|GMPJA3495-21|BIOUG65691-G03|BOLD:ABW7948
Phoridae|GMPJA1859-21|BIOUG65675-A05|BOLD:ABW7948
Phoridae|GMPJA5251-21|BIOUG66609-C02|BOLD:ABW7948
Phoridae|GMPJA8511-21|BIOUG66643-E08|BOLD:ACP4233

Phoridae|GMPJA1859-21|BIOUG65675-A05|BOLD:ABW7948
Phoridae|GMPJA5251-21|BIOUG66609-C02|BOLD:ABW7948
Phoridae|GMPJA8511-21|BIOUG66643-E08|BOLD:ACP4233
Phoridae|GMPJA10371-21|BIOUG70233-H06|BOLD:ACP4233
Phoridae|GMPJA8429-21|BIOUG66642-F09|BOLD:ACP4233
Megaselia|GMPJA4564-21|BIOUG66603-A04|BOLD:AEJ9548
Megaselia|GMPJA4227-21|BIOUG65699-D11|BOLD:ACA3150
Megaselia|GMPJA706-21|BIOUG65662-H03|BOLD:ACA3150
Megaselia|GMPJA3162-21|BIOUG65688-C03|BOLD:ACA3150
Megaselia|GMPJA8752-21|BIOUG66646-A12|BOLD:ACA3150
Megaselia|GMPJA7679-21|BIOUG66634-G07|BOLD:ACA3150
Megaselia|GMPJA7744-21|BIOUG66635-E01|BOLD:ACA3150
Megaselia|GMPJA2120-21|BIOUG65677-G04|BOLD:ACA3150
Megaselia|GMPJA8691-21|BIOUG66645-D10|BOLD:ACA3150
Megaselia|GMPJA7789-21|BIOUG66635-H10|BOLD:ACA3150
Megaselia|GMPJA8256-21|BIOUG66640-H02|BOLD:ACA3150
Megaselia|GMPJA8137-21|BIOUG66639-F02|BOLD:ACA3150
Megaselia|GMPJA7395-21|BIOUG66631-G08|BOLD:ACA3150
Megaselia|GMPJA10253-21|BIOUG70232-F07|BOLD:ACA3150
Megaselia|GMPJA4385-21|BIOUG66601-B03|BOLD:ACA3150
Megaselia|GMPJA10618-21|BIOUG70236-E04|BOLD:ACA3150
Megaselia|GMPJA8641-21|BIOUG66644-H07|BOLD:ACA3150
Megaselia|GMPJA8270-21|BIOUG66641-A05|BOLD:ACA3150
Megaselia|GMPJA5485-21|BIOUG66611-F10|BOLD:ACA3150
Megaselia|GMPJA8576-21|BIOUG66644-C02|BOLD:ACA3150
Megaselia|GMPJA5538-21|BIOUG66612-C04|BOLD:ACA3150
Megaselia|GMPJA4166-21|BIOUG65698-G09|BOLD:ACA3150
Megaselia|GMPJA7515-21|BIOUG66633-A10|BOLD:ACA3150
Megaselia|GMPJA4142-21|BIOUG65698-E09|BOLD:ACA3150
Megaselia|GMPJA8582-21|BIOUG66644-C08|BOLD:ACA3150
Megaselia|GMPJA3615-21|BIOUG65693-A05|BOLD:ACA3150
Megaselia|GMPJA8496-21|BIOUG66643-D05|BOLD:ACA3150
Megaselia|GMPJA2167-21|BIOUG65678-C04|BOLD:ACA3150
Megaselia|GMPJA8730-21|BIOUG66645-H01|BOLD:ACA3150
Megaselia|GMPJA8637-21|BIOUG66644-H03|BOLD:ACA3150
Megaselia|GMPJA7556-21|BIOUG66633-E03|BOLD:ACA3150
Megaselia|GMPJA5236-21|BIOUG66609-A11|BOLD:ACA3150
Megaselia|GMPJA6590-21|BIOUG66623-C11|BOLD:ACA3150
Megaselia|GMPJA10638-21|BIOUG70236-F12|BOLD:ACA3150
Megaselia|GMPJA6844-21|BIOUG66626-A04|BOLD:ACA3150
Megaselia|GMPJA6671-21|BIOUG66624-B09|BOLD:ACA3150
Megaselia|GMPJA8689-21|BIOUG66645-D08|BOLD:ACA3150
Megaselia|GMPJA8120-21|BIOUG66639-D09|BOLD:ACA3150
Megaselia|GMPJA084-21|BIOUG65656-C11|BOLD:ACA3150
Megaselia|GMPJA5483-21|BIOUG66611-F08|BOLD:ACA3150
Megaselia|GMPJA1467-21|BIOUG65670-H04|BOLD:ACA3150
Megaselia|GMPJA4325-21|BIOUG65700-E02|BOLD:ACA3150
Megaselia|GMPJA5478-21|BIOUG66611-F03|BOLD:ACA3150
Megaselia|GMPJA2122-21|BIOUG65677-G06|BOLD:ACA3150
Megaselia|GMPJA057-21|BIOUG65656-A08|BOLD:ACA3150
Megaselia|GMPJA4402-21|BIOUG66601-C08|BOLD:ACA3150
Megaselia|GMPJA4219-21|BIOUG65699-D03|BOLD:ACA3150
Megaselia|GMPJA8712-21|BIOUG66645-F07|BOLD:ACA3150
Megaselia|GMPJA8118-21|BIOUG66639-D07|BOLD:ACA3150
Megaselia|GMPJA7646-21|BIOUG66634-D10|BOLD:ACA3150
Megaselia|GMPJA7997-21|BIOUG66638-B05|BOLD:ACA3150
Megaselia|GMPJA7258-21|BIOUG66630-D02|BOLD:ACA3150
Megaselia|GMPJA7729-21|BIOUG66635-C10|BOLD:ACA3150
Megaselia|GMPJA7449-21|BIOUG66632-D03|BOLD:ACA3150
Megaselia|GMPJA6747-21|BIOUG66625-A02|BOLD:ACA3150
Megaselia|GMPJA6635-21|BIOUG66623-G08|BOLD:ACA3150
Megaselia|GMPJA6583-21|BIOUG66623-C04|BOLD:ACA3150
Megaselia|GMPJA5602-21|BIOUG66612-H08|BOLD:ACA3150
Megaselia|GMPJA5213-21|BIOUG66608-G11|BOLD:ACA3150
Megaselia|GMPJA4005-21|BIOUG65697-B03|BOLD:ACA3150
Megaselia|GMPJA5429-21|BIOUG66611-B02|BOLD:ACA3150
Megaselia|GMPJA8706-21|BIOUG66645-F01|BOLD:ACA3150
Megaselia|GMPJA3998-21|BIOUG65697-A08|BOLD:ACA3150
Megaselia|GMPJA5328-21|BIOUG66610-A08|BOLD:ACA3150
Phoridae|GMPJA1080-21|BIOUG65666-G09|BOLD:ABW5513
Phoridae|GMPJA1076-21|BIOUG65666-G05|BOLD:ABW5513
Phoridae|GMPJA2042-21|BIOUG65676-H09|BOLD:ABW5513
Phoridae|GMPJA1045-21|BIOUG65666-D10|BOLD:ABW5513
Phoridae|GMPJA379-21|BIOUG65659-D09|BOLD:ABW5513
Phoridae|GMPJA507-21|BIOUG65660-G06|BOLD:ABW5513
Phoridae|GMPJA3120-21|BIOUG65687-G08|BOLD:ABW5562
Phoridae|GMPJA5318-21|BIOUG66609-H09|BOLD:ABW5562
Phoridae|GMPJA6881-21|BIOUG66626-D05|BOLD:ABW5562
Phoridae|GMPJA5229-21|BIOUG66609-A04|BOLD:ABW5562
Phoridae|GMPJA5596-21|BIOUG66612-H02|BOLD:ABW5562
Phoridae|GMPJA5263-21|BIOUG66609-D02|BOLD:ABW5562
Phoridae|GMPJA2337-21|BIOUG65680-A08|BOLD:ABW5562
Phoridae|GMPJA4092-21|BIOUG65698-A07|BOLD:ABW5562
Phoridae|GMPJA4076-21|BIOUG65697-H02|BOLD:ABW5562
Phoridae|GMPJA4261-21|BIOUG65699-G09|BOLD:ABW5562
Phoridae|GMPJA3677-21|BIOUG65693-F07|BOLD:ABW5562
Phoridae|GMPJA7678-21|BIOUG66634-G06|BOLD:ABW5562
Phoridae|GMPJA6612-21|BIOUG66623-E09|BOLD:ABW5562
Phoridae|GMPJA5497-21|BIOUG66611-G10|BOLD:ABW5562
Phoridae|GMPJA5272-21|BIOUG66609-D11|BOLD:ABW5562
Phoridae|GMPJA5157-21|BIOUG66608-C03|BOLD:ABW5562
Phoridae|GMPJA5388-21|BIOUG66610-F08|BOLD:ABW5562
Phoridae|GMPJA7450-21|BIOUG66632-D04|BOLD:ACS3082
Phoridae|GMPJA6659-21|BIOUG66624-A09|BOLD:ACS3082
Phoridae|GMPJA6016-21|BIOUG66617-C07|BOLD:ACS3082
Phoridae|GMPJA5165-21|BIOUG66608-C11|BOLD:ACS3082
Phoridae|GMPJA8045-21|BIOUG66638-F05|BOLD:ACS3082
Phoridae|GMPJA5182-21|BIOUG66608-E04|BOLD:ACS3082
Phoridae|GMPJA8208-21|BIOUG66640-D02|BOLD:ACS3082
Phoridae|GMPJA10271-21|BIOUG70232-H01|BOLD:ACS3082
Phoridae|GMPJA8213-21|BIOUG66640-D07|BOLD:ACS3082
Phoridae|GMPJA8047-21|BIOUG66638-F07|BOLD:ACS3082
Phoridae|GMPJA1614-21|BIOUG65672-D09|BOLD:ACS3082

Phoridae|GMPJA8213-21|BIOUG66640-D07|BOLD:ACS3082
Phoridae|GMPJA8047-21|BIOUG66638-F07|BOLD:ACS3082
Phoridae|GMPJA1614-21|BIOUG65672-D09|BOLD:ACS3082
Phoridae|GMPJA083-21|BIOUG65656-C10|BOLD:ACS3082
Phoridae|GMPJA5055-21|BIOUG66607-B08|BOLD:ACS3082
Megaselia|GMPJA1450-21|BIOUG65670-F11|BOLD:ABW7950
Megaselia|GMPJA2261-21|BIOUG65679-C03|BOLD:ABW7950
Megaselia|GMPJA4463-21|BIOUG66601-H09|BOLD:ABW7950
Megaselia|GMPJA3169-21|BIOUG65688-C10|BOLD:ABW7950
Phoridae|GMPJA3654-21|BIOUG65693-D08|BOLD:ABX2832
Phoridae|GMPJA5539-21|BIOUG66612-C05|BOLD:ABX2832
Phoridae|GMPJA5219-21|BIOUG66608-H05|BOLD:ABX2832
Phoridae|GMPJA5116-21|BIOUG66607-G09|BOLD:ABX2832
Phoridae|GMPJA5099-21|BIOUG66607-F04|BOLD:ABX2832
Phoridae|GMPJA5536-21|BIOUG66612-C02|BOLD:ABX2832
Phoridae|GMPJA8428-21|BIOUG66642-F08|BOLD:ABX2832
Phoridae|GMPJA6250-21|BIOUG66619-G03|BOLD:ABX2832
Phoridae|GMPJA5049-21|BIOUG66607-B02|BOLD:ABX2832
Phoridae|GMPJA8274-21|BIOUG66641-A09|BOLD:ABX6652
Phoridae|GMPJA1030-21|BIOUG65666-C07|BOLD:ABW7976
Phoridae|GMPJA1950-21|BIOUG65676-A01|BOLD:ABW7976
Phoridae|GMPJA1627-21|BIOUG65672-E10|BOLD:ABW7976
Phoridae|GMPJA344-21|BIOUG65659-A10|BOLD:ABW7976
Phoridae|GMPJA3304-21|BIOUG65689-G02|BOLD:ABW7976
Phoridae|GMPJA1337-21|BIOUG65669-E05|BOLD:ABW7976
Phoridae|GMPJA2255-21|BIOUG65679-B09|BOLD:ABW7976
Phoridae|GMPJA1555-21|BIOUG65671-G09|BOLD:ABW7976
Phoridae|GMPJA931-21|BIOUG65665-C03|BOLD:ABW7976
Phoridae|GMPJA10277-21|BIOUG70232-H07|BOLD:ABW7976
Phoridae|GMPJA7248-21|BIOUG66630-C04|BOLD:ABW7976
Phoridae|GMPJA3454-21|BIOUG65691-C10|BOLD:ABW7976
Phoridae|GMPJA2495-21|BIOUG65681-F11|BOLD:ABW7976
Phoridae|GMPJA2022-21|BIOUG65676-G01|BOLD:ABW5515
Phoridae|GMPJA2129-21|BIOUG65677-H01|BOLD:ABW5515
Phoridae|GMPJA1355-21|BIOUG65669-F11|BOLD:ABW5515
Phoridae|GMPJA2298-21|BIOUG65679-F04|BOLD:ABW5515
Phoridae|GMPJA2290-21|BIOUG65679-E08|BOLD:ABW5515
Phoridae|GMPJA1441-21|BIOUG65670-F02|BOLD:ABW5515
Phoridae|GMPJA1353-21|BIOUG65669-F09|BOLD:ABW5515
Phoridae|GMPJA184-21|BIOUG65657-D04|BOLD:ABW5515
Phoridae|GMPJA3239-21|BIOUG65689-A09|BOLD:ABW5515
Megaselia halterata|GMPJA3199-21|BIOUG65688-F04|BOLD:ABV8939
Megaselia halterata|GMPJA1074-21|BIOUG65666-G03|BOLD:ABV8939
Megaselia halterata|GMPJA1027-21|BIOUG65666-C04|BOLD:ABV8939
Megaselia halterata|GMPJA1375-21|BIOUG65669-H07|BOLD:ABV8939
Megaselia halterata|GMPJA2316-21|BIOUG65679-G10|BOLD:ABV8939
Megaselia halterata|GMPJA947-21|BIOUG65665-D07|BOLD:ABV8939
Megaselia halterata|GMPJA648-21|BIOUG65662-C05|BOLD:ABV8939
Megaselia halterata|GMPJA2512-21|BIOUG65681-H04|BOLD:ABV8939
Megaselia halterata|GMPJA1921-21|BIOUG65675-F07|BOLD:ABV8939
Megaselia halterata|GMPJA1364-21|BIOUG65669-G08|BOLD:ABV8939
Megaselia halterata|GMPJA1563-21|BIOUG65671-H05|BOLD:ABV8939
Megaselia halterata|GMPJA1377-21|BIOUG65669-H09|BOLD:ABV8939
Megaselia halterata|GMPJA1152-21|BIOUG65667-E10|BOLD:ABV8939
Megaselia halterata|GMPJA910-21|BIOUG65665-A06|BOLD:ABV8939
Megaselia halterata|GMPJA1197-21|BIOUG65668-A08|BOLD:ABV8939
Megaselia halterata|GMPJA2194-21|BIOUG65678-E07|BOLD:ABV8939
Megaselia halterata|GMPJA1044-21|BIOUG65666-D09|BOLD:ABV8939
Megaselia halterata|GMPJA2175-21|BIOUG65678-C12|BOLD:ABV8939
Megaselia halterata|GMPJA4067-21|BIOUG65697-G05|BOLD:ABV8939
Megaselia halterata|GMPJA2355-21|BIOUG65680-C02|BOLD:ABV8939
Megaselia halterata|GMPJA3368-21|BIOUG65690-D07|BOLD:ABV8939
Megaselia halterata|GMPJA440-21|BIOUG65660-A11|BOLD:ABV8939
Megaselia halterata|GMPJA1727-21|BIOUG65673-F03|BOLD:ABV8939
Megaselia halterata|GMPJA1776-21|BIOUG65674-B05|BOLD:ABV8939
Megaselia halterata|GMPJA1942-21|BIOUG65675-H04|BOLD:ABV8939
Megaselia halterata|GMPJA1786-21|BIOUG65674-C03|BOLD:ABV8939
Megaselia halterata|GMPJA2079-21|BIOUG65677-C11|BOLD:ABV8939
Megaselia halterata|GMPJA2063-21|BIOUG65677-B07|BOLD:ABV8939
Megaselia halterata|GMPJA1709-21|BIOUG65673-D09|BOLD:ABV8939
Megaselia halterata|GMPJA2350-21|BIOUG65680-B09|BOLD:ABV8939
Megaselia halterata|GMPJA2333-21|BIOUG65680-A04|BOLD:ABV8939
Megaselia halterata|GMPJA2325-21|BIOUG65679-H07|BOLD:ABV8939
Megaselia halterata|GMPJA2251-21|BIOUG65679-B05|BOLD:ABV8939
Megaselia halterata|GMPJA2184-21|BIOUG65678-D09|BOLD:ABV8939
Megaselia halterata|GMPJA2171-21|BIOUG65678-C08|BOLD:ABV8939
Megaselia halterata|GMPJA1621-21|BIOUG65672-E04|BOLD:ABV8939
Megaselia halterata|GMPJA1604-21|BIOUG65672-C11|BOLD:ABV8939
Megaselia halterata|GMPJA1594-21|BIOUG65672-C01|BOLD:ABV8939
Megaselia halterata|GMPJA1561-21|BIOUG65671-H03|BOLD:ABV8939
Megaselia halterata|GMPJA1475-21|BIOUG65671-A01|BOLD:ABV8939
Megaselia halterata|GMPJA1851-21|BIOUG65674-H08|BOLD:ABV8939
Megaselia halterata|GMPJA1800-21|BIOUG65674-D05|BOLD:ABV8939
Megaselia halterata|GMPJA1384-21|BIOUG65670-A05|BOLD:ABV8939
Megaselia halterata|GMPJA1347-21|BIOUG65669-F03|BOLD:ABV8939
Megaselia halterata|GMPJA1050-21|BIOUG65666-E03|BOLD:ABV8939
Megaselia halterata|GMPJA733-21|BIOUG65663-B07|BOLD:ABV8939
Megaselia halterata|GMPJA721-21|BIOUG65663-A07|BOLD:ABV8939
Megaselia halterata|GMPJA1310-21|BIOUG65669-C02|BOLD:ABV8939
Megaselia halterata|GMPJA1766-21|BIOUG65674-A07|BOLD:ABV8939
Megaselia halterata|GMPJA1761-21|BIOUG65674-A02|BOLD:ABV8939
Megaselia halterata|GMPJA695-21|BIOUG65662-G04|BOLD:ABV8939
Megaselia halterata|GMPJA681-21|BIOUG65662-F02|BOLD:ABV8939
Megaselia halterata|GMPJA664-21|BIOUG65662-D09|BOLD:ABV8939
Megaselia halterata|GMPJA633-21|BIOUG65662-B02|BOLD:ABV8939
Megaselia halterata|GMPJA626-21|BIOUG65662-A07|BOLD:ABV8939
Megaselia halterata|GMPJA625-21|BIOUG65662-A06|BOLD:ABV8939
Megaselia halterata|GMPJA624-21|BIOUG65662-A05|BOLD:ABV8939
Megaselia halterata|GMPJA384-21|BIOUG65659-E02|BOLD:ABV8939
Megaselia halterata|GMPJA365-21|BIOUG65659-C07|BOLD:ABV8939
Megaselia halterata|GMPJA907-21|BIOUG65665-A03|BOLD:ABV8939
Megaselia halterata|GMPJA493-21|BIOUG65660-F04|BOLD:ABV8939
Megaselia halterata|GMPJA3655-21|BIOUG65693-D09|BOLD:ABV8939

Megaselia halterata|GMPJA907-21|BIOUG65665-A03|BOLD:ABV8939
Megaselia halterata|GMPJA493-21|BIOUG65660-F04|BOLD:ABV8939
Megaselia halterata|GMPJA3655-21|BIOUG65693-D09|BOLD:ABV8939
Megaselia halterata|GMPJA3639-21|BIOUG65693-C05|BOLD:ABV8939
Megaselia halterata|GMPJA3625-21|BIOUG65693-B03|BOLD:ABV8939
Megaselia halterata|GMPJA3582-21|BIOUG65692-F07|BOLD:ABV8939
Megaselia halterata|GMPJA4408-21|BIOUG66601-D02|BOLD:ABV8939
Megaselia halterata|GMPJA4309-21|BIOUG65700-C10|BOLD:ABV8939
Megaselia halterata|GMPJA4269-21|BIOUG65699-H05|BOLD:ABV8939
Megaselia halterata|GMPJA4231-21|BIOUG65699-E03|BOLD:ABV8939
Megaselia halterata|GMPJA4212-21|BIOUG65699-C08|BOLD:ABV8939
Megaselia halterata|GMPJA3467-21|BIOUG65691-D11|BOLD:ABV8939
Megaselia halterata|GMPJA3465-21|BIOUG65691-D09|BOLD:ABV8939
Megaselia halterata|GMPJA3298-21|BIOUG65689-F08|BOLD:ABV8939
Megaselia halterata|GMPJA3270-21|BIOUG65689-D04|BOLD:ABV8939
Megaselia halterata|GMPJA3401-21|BIOUG65690-G04|BOLD:ABV8939
Megaselia halterata|GMPJA3256-21|BIOUG65689-C02|BOLD:ABV8939
Megaselia halterata|GMPJA3238-21|BIOUG65689-A08|BOLD:ABV8939
Megaselia halterata|GMPJA3099-21|BIOUG65687-E11|BOLD:ABV8939
Megaselia halterata|GMPJA3097-21|BIOUG65687-E09|BOLD:ABV8939
Megaselia halterata|GMPJA3181-21|BIOUG65688-D10|BOLD:ABV8939
Megaselia halterata|GMPJA5206-21|BIOUG66608-G04|BOLD:ABV8939
Megaselia halterata|GMPJA2766-21|BIOUG65684-E09|BOLD:ABV8939
Megaselia halterata|GMPJA2518-21|BIOUG65681-H10|BOLD:ABV8939
Megaselia halterata|GMPJA2476-21|BIOUG65681-E04|BOLD:ABV8939
Megaselia halterata|GMPJA2453-21|BIOUG65681-C05|BOLD:ABV8939
Megaselia halterata|GMPJA2445-21|BIOUG65681-B09|BOLD:ABV8939
Megaselia halterata|GMPJA1470-21|BIOUG65670-H07|BOLD:ABV8939
Megaselia halterata|GMPJA1887-21|BIOUG65675-C09|BOLD:ABV8939
Megaselia halterata|GMPJA1592-21|BIOUG65672-B11|BOLD:ABV8939
Megaselia halterata|GMPJA1565-21|BIOUG65671-H07|BOLD:ABV8939
Megaselia halterata|GMPJA2030-21|BIOUG65676-G09|BOLD:ABV8939
Megaselia halterata|GMPJA2006-21|BIOUG65676-E09|BOLD:ABV8939
Megaselia halterata|GMPJA2086-21|BIOUG65677-D06|BOLD:ABV8939
Megaselia halterata|GMPJA2090-21|BIOUG65677-D10|BOLD:ABV8939
Megaselia halterata|GMPJA2093-21|BIOUG65677-E01|BOLD:ABV8939
Megaselia halterata|GMPJA2106-21|BIOUG65677-F02|BOLD:ABV8939
Megaselia halterata|GMPJA2132-21|BIOUG65677-H04|BOLD:ABV8939
Megaselia halterata|GMPJA2144-21|BIOUG65678-A05|BOLD:ABV8939
Megaselia halterata|GMPJA2373-21|BIOUG65680-D08|BOLD:ABV8939
Megaselia halterata|GMPJA2387-21|BIOUG65680-E10|BOLD:ABV8939
Megaselia halterata|GMPJA1691-21|BIOUG65673-C03|BOLD:ABV8939
Megaselia halterata|GMPJA1436-21|BIOUG65670-E09|BOLD:ABV8939
Megaselia halterata|GMPJA4247-21|BIOUG65699-F07|BOLD:ABV8939
Megaselia halterata|GMPJA4232-21|BIOUG65699-E04|BOLD:ABV8939
Megaselia halterata|GMPJA1482-21|BIOUG65671-A08|BOLD:ABV8939
Megaselia halterata|GMPJA1898-21|BIOUG65675-D08|BOLD:ABV8939
Megaselia halterata|GMPJA4404-21|BIOUG66601-C10|BOLD:ABV8939
Megaselia halterata|GMPJA3061-21|BIOUG65687-B09|BOLD:ABV8939
Megaselia halterata|GMPJA2431-21|BIOUG65681-A07|BOLD:ABV8939
Sphaeroceridae|GMPJA7659-21|BIOUG66634-E11|BOLD:ABW7892
Sphaeroceridae|GMPJA7607-21|BIOUG66634-A07|BOLD:ABW7892
Sphaeroceridae|GMPJA8356-21|BIOUG66641-H07|BOLD:ABW7892
Sphaeroceridae|GMPJA8233-21|BIOUG66640-F03|BOLD:ABW7892
Sphaeroceridae|GMPJA7904-21|BIOUG66637-B07|BOLD:ABW7892
Sphaeroceridae|GMPJA7667-21|BIOUG66634-F07|BOLD:ABW7892
Sphaeroceridae|GMPJA8516-21|BIOUG66643-F01|BOLD:ABW7892
Sphaeroceridae|GMPJA8431-21|BIOUG66642-F11|BOLD:ABW7892
Sphaeroceridae|GMPJA7570-21|BIOUG66633-F05|BOLD:ABW7892
Sphaeroceridae|GMPJA7336-21|BIOUG66631-B09|BOLD:ABW7892
Sphaeroceridae|GMPJA7298-21|BIOUG66630-G06|BOLD:ABW7892
Sphaeroceridae|GMPJA7295-21|BIOUG66630-G03|BOLD:ABW7892
Sphaeroceridae|GMPJA8761-21|BIOUG66646-B09|BOLD:ABW7892
Sphaeroceridae|GMPJA8650-21|BIOUG66645-A05|BOLD:ABW7892
Sphaeroceridae|GMPJA7284-21|BIOUG66630-F04|BOLD:ABW7892
Coproica|GMPJA7911-21|BIOUG66637-C02|BOLD:ABW7888
Coproica|GMPJA8591-21|BIOUG66644-D05|BOLD:ABW7888
Coproica|GMPJA7565-21|BIOUG66633-E12|BOLD:ABW7888
Coproica|GMPJA7431-21|BIOUG66632-B09|BOLD:ABW7888
Coproica|GMPJA7738-21|BIOUG66635-D07|BOLD:ABW7888
Coproica|GMPJA7539-21|BIOUG66633-C10|BOLD:ABW7888
Coproica|GMPJA8398-21|BIOUG66642-D02|BOLD:ABW7888
Coproica|GMPJA8780-21|BIOUG66646-D04|BOLD:ABW7888
Coproica|GMPJA8002-21|BIOUG66638-B10|BOLD:ABW7888
Coproica|GMPJA3999-21|BIOUG65697-A09|BOLD:ABW7888
Coproica|GMPJA3499-21|BIOUG65691-G07|BOLD:ABW7888
Coproica|GMPJA3340-21|BIOUG65690-B03|BOLD:ABW7888
Coproica|GMPJA7940-21|BIOUG66637-E07|BOLD:ABW7888
Coproica|GMPJA7585-21|BIOUG66633-G08|BOLD:ABW7888
Coproica|GMPJA7981-21|BIOUG66638-A01|BOLD:ABW7888
Coproica|GMPJA7415-21|BIOUG66632-A05|BOLD:ABW7888
Coproica|GMPJA6820-21|BIOUG66625-G03|BOLD:ABW7888
Coproica|GMPJA6515-21|BIOUG66622-E07|BOLD:ABW7888
Drosophila busckii|GMPJA1145-21|BIOUG65667-E03|BOLD:AAM6803
Ephydridae|GMPJA752-21|BIOUG65663-D02|BOLD:ACG2025
Ephydridae|GMPJA465-21|BIOUG65660-C12|BOLD:ACG2025
Melanagromyza|GMPJA1641-21|BIOUG65672-F12|BOLD:ABX2154
Phytomyza|GMPJA2279-21|BIOUG65679-D09|BOLD:ABX2164
Agromyzidae|GMPJA8500-21|BIOUG66643-D09|BOLD:ACO8055
Agromyzidae|GMPJA8127-21|BIOUG66639-E04|BOLD:ACO8055
Agromyzidae|GMPJA7587-21|BIOUG66633-G10|BOLD:ACO8055
Agromyzidae|GMPJA8049-21|BIOUG66638-F09|BOLD:ACO8055
Agromyzidae|GMPJA547-21|BIOUG65661-B11|BOLD:ACV1277
Agromyzidae|GMPJA056-21|BIOUG65656-A07|BOLD:ABX2135
Phytomyza thalictrella|GMPJA4196-21|BIOUG65699-B04|BOLD:ABX2176
Phytomyza thalictrella|GMPJA4477-21|BIOUG66602-A12|BOLD:ABX2176
Phytomyza thalictrella|GMPJA8776-21|BIOUG66646-C12|BOLD:ABX2176
Ephydridae|GMPJA10189-21|BIOUG70232-A03|BOLD:AEK2503
Ephydridae|GMPJA7235-21|BIOUG66630-B03|BOLD:AEK2503
Phytoliriomyza|GMPJA2156-21|BIOUG65678-B05|BOLD:ABX2113
Phytoliriomyza|GMPJA2037-21|BIOUG65676-H04|BOLD:ABX2113
Liriomyza brassicae|GMPJA3674-21|BIOUG65693-F04|BOLD:AAF6806
Liriomyza brassicae|GMPJA10349-21|BIOUG70233-F08|BOLD:AAF6806

[illegible]
Phytoliriomyza|GMPJA2037-21|BIOUG65676-H04|BOLD:ABX2113
Liriomyza brassicae|GMPJA3674-21|BIOUG65693-F04|BOLD:AAF6806
Liriomyza brassicae|GMPJA10349-21|BIOUG70233-F08|BOLD:AAF6806
Agromyzidae|GMPJA438-21|BIOUG65660-A09|BOLD:AEK2073
Phytomyzinae|GMPJA8087-21|BIOUG66639-A12|BOLD:AAF6797
Phytomyzinae|GMPJA544-21|BIOUG65661-B08|BOLD:AAF6797
Phytomyzinae|GMPJA8218-21|BIOUG66640-D12|BOLD:AAF6797
Phytomyzinae|GMPJA5565-21|BIOUG66612-E07|BOLD:AAF6797
Cacoxenus|GMPJA1540-21|BIOUG65671-F06|BOLD:ACQ2399
Cacoxenus|GMPJA1001-21|BIOUG65666-A02|BOLD:ACQ2399
Ophiomyia|GMPJA119-21|BIOUG65656-F10|BOLD:AAW9165
Agromyzidae|GMPJA2103-21|BIOUG65677-E11|BOLD:ACG0943
Agromyzidae|GMPJA1723-21|BIOUG65673-E11|BOLD:ACG0943
Agromyzidae|GMPJA1630-21|BIOUG65672-F01|BOLD:ACG0943
Ophiomyia lantanae|GMPJA1620-21|BIOUG65672-E03|BOLD:AAW9154
Agromyzidae|GMPJA10332-21|BIOUG70233-E03|BOLD:ABZ8478
Agromyzidae|GMPJA10208-21|BIOUG70232-B10|BOLD:ABZ8478
Agromyzidae|GMPJA7268-21|BIOUG66630-D12|BOLD:ABZ8478
Agromyzidae|GMPJA7696-21|BIOUG66635-A01|BOLD:ABZ8478
Agromyzidae|GMPJA6850-21|BIOUG66626-A10|BOLD:ABZ8478
Agromyzidae|GMPJA5274-21|BIOUG66609-E01|BOLD:ABZ8478
Melanagromyza obtusa|GMPJA1765-21|BIOUG65674-A06|BOLD:AAT3633
Melanagromyza obtusa|GMPJA2056-21|BIOUG65677-A12|BOLD:AAT3633
Melanagromyza obtusa|GMPJA3681-21|BIOUG65693-F11|BOLD:AAT3633
Agromyzidae|GMPJA7933-21|BIOUG66637-D12|BOLD:ADU0050
Agromyzidae|GMPJA8010-21|BIOUG66638-C06|BOLD:ACP3491
Agromyzidae|GMPJA7989-21|BIOUG66638-A09|BOLD:ACP3491
Agromyzidae|GMPJA6880-21|BIOUG66626-D04|BOLD:ADT3531
Agromyzidae|GMPJA3569-21|BIOUG65692-E06|BOLD:ADU6291
Agromyzidae|GMPJA8802-21|BIOUG66646-F02|BOLD:ADU6291
Agromyzidae|GMPJA4480-21|BIOUG66602-B03|BOLD:ACM4922
Agromyzidae|GMPJA7561-21|BIOUG66633-E08|BOLD:ACM4922
Agromyzidae|GMPJA7784-21|BIOUG66635-H05|BOLD:ABY1623
Agromyzidae|GMPJA7790-21|BIOUG66635-H11|BOLD:ABY1623
Agromyzidae|GMPJA10623-21|BIOUG70236-E09|BOLD:ABY1623
Agromyzidae|GMPJA8711-21|BIOUG66645-F06|BOLD:ABY1623
Agromyzidae|GMPJA8480-21|BIOUG66643-C01|BOLD:ABY1623
Agromyzidae|GMPJA8284-21|BIOUG66641-B07|BOLD:ABY1623
Agromyzidae|GMPJA8080-21|BIOUG66639-A05|BOLD:ABY1623
Agromyzidae|GMPJA7349-21|BIOUG66631-C10|BOLD:ABY1623
Agromyzidae|GMPJA6855-21|BIOUG66626-B03|BOLD:ABY1623
Agromyzidae|GMPJA6725-21|BIOUG66624-G03|BOLD:ABY1623
Agromyzidae|GMPJA7520-21|BIOUG66633-B03|BOLD:ABY1623
Agromyzidae|GMPJA4328-21|BIOUG65700-E05|BOLD:ABY1623
Agromyzidae|GMPJA7686-21|BIOUG66634-H02|BOLD:ABY1623
Agromyzidae|GMPJA7914-21|BIOUG66637-C05|BOLD:ABY1623
Agromyzidae|GMPJA7459-21|BIOUG66632-E01|BOLD:ABY1623
Agromyzidae|GMPJA7722-21|BIOUG66635-C03|BOLD:ABY1623
Agromyzidae|GMPJA4159-21|BIOUG65698-G02|BOLD:ABY1623
Agromyzidae|GMPJA4362-21|BIOUG65700-H03|BOLD:ABY1623
Agromyzidae|GMPJA7581-21|BIOUG66633-G04|BOLD:ABY1623
Agromyzidae|GMPJA4255-21|BIOUG65699-G03|BOLD:ABY1623
Agromyzidae|GMPJA4147-21|BIOUG65698-F02|BOLD:ABY1623
Agromyzidae|GMPJA8664-21|BIOUG66645-B07|BOLD:ABY1623
Agromyzidae|GMPJA8610-21|BIOUG66644-E12|BOLD:ABY1623
Agromyzidae|GMPJA4367-21|BIOUG65700-H08|BOLD:ABY1623
Agromyzidae|GMPJA6547-21|BIOUG66622-H03|BOLD:ABY1623
Agromyzidae|GMPJA7779-21|BIOUG66635-G12|BOLD:ABY1623
Agromyzidae|GMPJA8248-21|BIOUG66640-G06|BOLD:ABY1623
Agromyzidae|GMPJA4427-21|BIOUG66601-E09|BOLD:ABY1623
Agromyzidae|GMPJA5534-21|BIOUG66612-B12|BOLD:ABY1623
Agromyzidae|GMPJA6454-21|BIOUG66621-H05|BOLD:ABY1623
Agromyzidae|GMPJA10242-21|BIOUG70232-E08|BOLD:ABY1623
Agromyzidae|GMPJA8455-21|BIOUG66642-H11|BOLD:ABY1623
Agromyzidae|GMPJA7964-21|BIOUG66637-G07|BOLD:ABY1623
Agromyzidae|GMPJA8460-21|BIOUG66643-A05
Agromyzidae|GMPJA8153-21|BIOUG66639-G06|BOLD:AEJ2112
Agromyzidae|GMPJA10660-21|BIOUG70236-H10|BOLD:ABY1623
Agromyzidae|GMPJA6567-21|BIOUG66623-A12|BOLD:ABY1623
Agromyzidae|GMPJA5235-21|BIOUG66609-A10|BOLD:ABY1623
Agromyzidae|GMPJA5156-21|BIOUG66608-C02|BOLD:ABY1623
Drosophilidae|GMPJA6709-21|BIOUG66624-E11|BOLD:ACG0240
Drosophilidae|GMPJA10288-21|BIOUG70233-A07|BOLD:ACG0240
Drosophilidae|GMPJA6863-21|BIOUG66626-B11|BOLD:ACG0240
Drosophilidae|GMPJA6796-21|BIOUG66625-E03|BOLD:ACG0240
Drosophilidae|GMPJA6404-21|BIOUG66621-D03|BOLD:ACG0240
Drosophilidae|GMPJA6367-21|BIOUG66621-A02|BOLD:ACG0240
Meoneura palaestinensis|GMPJA5359-21|BIOUG66610-D03|BOLD:ACO5552
Meoneura palaestinensis|GMPJA5304-21|BIOUG66609-G07|BOLD:ACO5552
Meoneura palaestinensis|GMPJA5461-21|BIOUG66611-D10|BOLD:ACO5552
Meoneura palaestinensis|GMPJA5420-21|BIOUG66611-A05|BOLD:ACO5552
Meoneura palaestinensis|GMPJA5365-21|BIOUG66610-D09|BOLD:ACO5552
Meoneura palaestinensis|GMPJA4482-21|BIOUG66602-B05|BOLD:ACO5552
Meoneura palaestinensis|GMPJA5056-21|BIOUG66607-B09|BOLD:ACO5552
Ephydridae|GMPJA729-21|BIOUG65663-B03|BOLD:ABX0206
Ephydridae|GMPJA2417-21|BIOUG65680-H04|BOLD:ABX0206
Ephydridae|GMPJA143-21|BIOUG65656-H10|BOLD:ABX0206
Ephydridae|GMPJA985-21|BIOUG65665-G09|BOLD:ABX0206
Ephydridae|GMPJA740-21|BIOUG65663-C02|BOLD:ABX0206
Ephydridae|GMPJA102-21|BIOUG65656-E05|BOLD:ABX0206
Ephydridae|GMPJA412-21|BIOUG65659-G06|BOLD:ABX0206
Ephydridae|GMPJA2263-21|BIOUG65679-C05|BOLD:ABX0302
Ephydridae|GMPJA1648-21|BIOUG65672-G07|BOLD:ABX0302
Ephydridae|GMPJA1141-21|BIOUG65667-D11|BOLD:ABX0302
Ephydridae|GMPJA951-21|BIOUG65665-D11|BOLD:ABX0302
Ephydridae|GMPJA055-21|BIOUG65656-A06|BOLD:ABX6211
Ephydridae|GMPJA6759-21|BIOUG66625-B02|BOLD:ABX6211
Nostima|GMPJA748-21|BIOUG65663-C10|BOLD:ACA2879
Nostima|GMPJA4475-21|BIOUG66602-A10|BOLD:ACA2879
Nostima|GMPJA1803-21|BIOUG65674-D08|BOLD:ACA2879
Nostima|GMPJA10230-21|BIOUG70232-D08|BOLD:ACA2879
Nostima|GMPJA666-21|BIOUG65662-D11|BOLD:ACA2879
[illegible]

Nostima|GMPJA18[illegible]-21|BIOUG656[illegible]-D0[illegible]|BOLD:ACA2879
Nostima|GMPJA10230-21|BIOUG70232-D08|BOLD:ACA2879
Nostima|GMPJA666-21|BIOUG65662-D11|BOLD:ACA2879
Nostima|GMPJA350-21|BIOUG65659-B04|BOLD:ACA2879
Nostima|GMPJA5366-21|BIOUG66610-D10|BOLD:ACA2879
Muscidae|GMPJA5476-21|BIOUG66611-F01|BOLD:ADK0626
Coenosia attenuata|GMPJA3617-21|BIOUG65693-A07|BOLD:AAD7633
Coenosia attenuata|GMPJA10633-21|BIOUG70236-F07|BOLD:AAD7633
Coenosia attenuata|GMPJA490-21|BIOUG65660-F01|BOLD:AAD7633
Coenosia attenuata|GMPJA3468-21|BIOUG65691-D12|BOLD:AAD7633
Coenosia attenuata|GMPJA4558-21|BIOUG66602-H09|BOLD:AAD7633
Coenosia attenuata|GMPJA4453-21|BIOUG66601-G11|BOLD:AAD7633
Coenosia attenuata|GMPJA3161-21|BIOUG65688-C02|BOLD:AAD7633
Coenosia attenuata|GMPJA8539-21|BIOUG66643-G12|BOLD:AAD7633
Coenosia attenuata|GMPJA1472-21|BIOUG65670-H09|BOLD:AAD7633
Coenosia attenuata|GMPJA4008-21|BIOUG65697-B06|BOLD:AAD7633
Coenosia attenuata|GMPJA3627-21|BIOUG65693-B05|BOLD:AAD7633
Coenosia attenuata|GMPJA4552-21|BIOUG66602-H03|BOLD:AAD7633
Coenosia attenuata|GMPJA6803-21|BIOUG66625-E10|BOLD:AAD7633
Coenosia attenuata|GMPJA3443-21|BIOUG65691-B11|BOLD:AAD7633
Coenosia attenuata|GMPJA4500-21|BIOUG66602-C11|BOLD:AAD7633
Coenosia attenuata|GMPJA4510-21|BIOUG66602-D09|BOLD:AAD7633
Coenosia attenuata|GMPJA2276-21|BIOUG65679-D06|BOLD:AAD7633
Coenosia attenuata|GMPJA1725-21|BIOUG65673-F01|BOLD:AAD7633
Coenosia attenuata|GMPJA1738-21|BIOUG65673-G02|BOLD:AAD7633
Coenosia attenuata|GMPJA1997-21|BIOUG65676-D12|BOLD:AAD7633
Coenosia attenuata|GMPJA4342-21|BIOUG65700-F07|BOLD:AAD7633
Coenosia attenuata|GMPJA10591-21|BIOUG70236-C01|BOLD:AAD7633
Coenosia attenuata|GMPJA4579-21|BIOUG66603-B07|BOLD:AAD7633
Coenosia attenuata|GMPJA2327-21|BIOUG65679-H09|BOLD:AAD7633
Coenosia attenuata|GMPJA4151-21|BIOUG65698-F06|BOLD:AAD7633
Coenosia attenuata|GMPJA8236-21|BIOUG66640-F06|BOLD:AAD7633
Coenosia attenuata|GMPJA3148-21|BIOUG65688-B01|BOLD:AAD7633
Coenosia attenuata|GMPJA4327-21|BIOUG65700-E04|BOLD:AAD7633
Coenosia attenuata|GMPJA2145-21|BIOUG65678-A06|BOLD:AAD7633
Coenosia attenuata|GMPJA6559-21|BIOUG66623-A04|BOLD:AAD7633
Coenosia attenuata|GMPJA6444-21|BIOUG66621-G07|BOLD:AAD7633
Coenosia acuticornis|GMPJA1582-21|BIOUG65672-B01|BOLD:AAG6793
Coenosia acuticornis|GMPJA157-21|BIOUG65657-B01|BOLD:AAG6793
Coenosia acuticornis|GMPJA10529-21|BIOUG70235-E10|BOLD:AAG6793
Coenosia acuticornis|GMPJA443-21|BIOUG65660-B02|BOLD:AAG6793
Coenosia acuticornis|GMPJA496-21|BIOUG65660-F07|BOLD:AAG6793
Coenosia acuticornis|GMPJA229-21|BIOUG65657-H01|BOLD:AAG6793
Coenosia acuticornis|GMPJA3588-21|BIOUG65692-G01|BOLD:AAG6793
Coenosia acuticornis|GMPJA3474-21|BIOUG65691-E06|BOLD:AAG6793
Coenosia acuticornis|GMPJA5320-21|BIOUG66609-H11|BOLD:AAG6793
Coenosia acuticornis|GMPJA2497-21|BIOUG65681-G01|BOLD:AAG6793
Ephydridae|GMPJA191-21|BIOUG65657-D11|BOLD:ACV0496
Ephydridae|GMPJA8450-21|BIOUG66642-H06|BOLD:AEJ1222
Drosophilinae|GMPJA8028-21|BIOUG66638-D12|BOLD:AAB6200
Musca ventrosa|GMPJA477-21|BIOUG65660-D12|BOLD:AAX3130
Musca|GMPJA4412-21|BIOUG66601-D06|BOLD:ADV1109
Musca crassirostris|GMPJA6686-21|BIOUG66624-C12|BOLD:AAF6545
Dolichopodidae|GMPJA8297-21|BIOUG66641-C08|BOLD:AEJ3624
Drosophila|GMPJA1831-21|BIOUG65674-F12|BOLD:AAV6734
Drosophila|GMPJA6821-21|BIOUG66625-G04|BOLD:AAV6734
Leucophenga|GMPJA6442-21|BIOUG66621-G05|BOLD:ACP4278
Culicidae|GMPJA383-21|BIOUG65659-E01|BOLD:AAA4751
Ephydridae|GMPJA10250-21|BIOUG70232-F04|BOLD:AEL0517
Chyromyidae|GMPJA1918-21|BIOUG65675-F04|BOLD:ACV5073
Ephydridae|GMPJA7831-21|BIOUG66636-D05|BOLD:ACA3099
Ephydridae|GMPJA10640-21|BIOUG70236-G02|BOLD:ACA3099
Ephydridae|GMPJA10637-21|BIOUG70236-F11|BOLD:ACA3099
Ephydridae|GMPJA453-21|BIOUG65660-B12|BOLD:ACA3099
Ephydridae|GMPJA5289-21|BIOUG66609-F04|BOLD:ACA3099
Ephydridae|GMPJA5154-21|BIOUG66608-B12|BOLD:ACA3099
Ephydridae|GMPJA5136-21|BIOUG66608-A06|BOLD:ACA3099
Ephydridae|GMPJA8813-21|BIOUG66646-G01|BOLD:AEK1134
Ephydridae|GMPJA8016-21|BIOUG66638-C12|BOLD:AEK1134
Ephydridae|GMPJA7383-21|BIOUG66631-F08|BOLD:AEK1134
Ephydridae|GMPJA5186-21|BIOUG66608-E08|BOLD:AEK1134
Ephydridae|GMPJA5117-21|BIOUG66607-G10|BOLD:AEK1134
Ephydridae|GMPJA10108-21|BIOUG66563-B06|BOLD:AEJ6745
Ephydridae|GMPJA5166-21|BIOUG66608-C12|BOLD:AEJ6745
Ephydridae|GMPJA5606-21|BIOUG66613-A01|BOLD:AEJ6745
Ephydridae|GMPJA5613-21|BIOUG66613-A08|BOLD:AEJ6745
Ephydridae|GMPJA6473-21|BIOUG66622-B01|BOLD:AEJ6745
Ephydridae|GMPJA6745-21|BIOUG66624-H11|BOLD:AEJ6745
Ephydridae|GMPJA6533-21|BIOUG66622-G01|BOLD:AEJ6745
Ephydridae|GMPJA6545-21|BIOUG66622-H01|BOLD:AEJ6745
Ephydridae|GMPJA6568-21|BIOUG66623-B01|BOLD:AEJ6745
Ephydridae|GMPJA6758-21|BIOUG66625-B01|BOLD:AEJ6745
Ephydridae|GMPJA7364-21|BIOUG66631-E01|BOLD:AEJ6745
Ephydridae|GMPJA7316-21|BIOUG66631-A01|BOLD:AEJ6745
Ephydridae|GMPJA10113-21|BIOUG66563-B11|BOLD:AEJ6745
Ephydridae|GMPJA10166-21|BIOUG66563-G04|BOLD:AEJ6745
Ephydridae|GMPJA5398-21|BIOUG66610-G06|BOLD:AEJ6745
Ephydridae|GMPJA5369-21|BIOUG66610-E01|BOLD:AEJ6745
Philygria|GMPJA064-21|BIOUG65656-B03|BOLD:AEJ7310
Philygria|GMPJA1168-21|BIOUG65667-G02|BOLD:ABW7973
Philygria|GMPJA8012-21|BIOUG66638-C08|BOLD:ABW7973
Polytrichophora|GMPJA7325-21|BIOUG66631-A10|BOLD:AEJ5171
Ephydridae|GMPJA7578-21|BIOUG66633-G01|BOLD:ACU9704
Ephydridae|GMPJA8379-21|BIOUG66642-B07|BOLD:ACU9704
Ephydridae|GMPJA6373-21|BIOUG66621-A08|BOLD:ACU9704
Ephydridae|GMPJA7441-21|BIOUG66632-C07|BOLD:ACU9704
Ephydridae|GMPJA6632-21|BIOUG66623-G05|BOLD:ACU9704
Ephydridae|GMPJA5518-21|BIOUG66612-A08|BOLD:ACU9704
Ephydridae|GMPJA4501-21|BIOUG66602-C12|BOLD:ACS3391
Ephydridae|GMPJA6425-21|BIOUG66621-E12|BOLD:ACY2855
Ephydridae|GMPJA7286-21|BIOUG66630-F06|BOLD:ACO4648
Ephydridae|GMPJA5505-21|BIOUG66611-H06|BOLD:ACO4648
Ephydridae|GMPJA5481-21|BIOUG66611-F06|BOLD:ACO4648
[illegible]

Ephydridae|GMPJA7286-21|BIOUG66630-F06|BOLD:ACO4648
Ephydridae|GMPJA5505-21|BIOUG66611-H06|BOLD:ACO4648
Ephydridae|GMPJA5481-21|BIOUG66611-F06|BOLD:ACO4648
Ephydridae|GMPJA7767-21|BIOUG66635-F12|BOLD:ACO4648
Ephydridae|GMPJA430-21|BIOUG65660-A01|BOLD:ACO4648
Ephydridae|GMPJA7315-21|BIOUG66630-H11|BOLD:ACO4648
Ephydridae|GMPJA5222-21|BIOUG66608-H08|BOLD:ACO4648
Ephydridae|GMPJA5540-21|BIOUG66612-C06|BOLD:ACO4648
Ephydridae|GMPJA6664-21|BIOUG66624-B02|BOLD:ACO4648
Ephydridae|GMPJA6828-21|BIOUG66625-G11|BOLD:ACO4648
Ephydridae|GMPJA7363-21|BIOUG66631-D12|BOLD:ACO4648
Ephydridae|GMPJA6882-21|BIOUG66626-D06|BOLD:ACO4648
Ephydridae|GMPJA10646-21|BIOUG70236-G08|BOLD:ACO4648
Ephydridae|GMPJA5114-21|BIOUG66607-G07|BOLD:ACO4648
Ephydridae|GMPJA5255-21|BIOUG66609-C06|BOLD:ACO4648
Ephydridae|GMPJA7601-21|BIOUG66634-A01|BOLD:ACO4648
Ephydridae|GMPJA5452-21|BIOUG66611-D01|BOLD:ACO4648
Ephydridae|GMPJA5415-21|BIOUG66610-H11|BOLD:ACO4648
Ephydridae|GMPJA5440-21|BIOUG66611-C01|BOLD:ACO4648
Ephydridae|GMPJA5205-21|BIOUG66608-G03|BOLD:ACO4648
Ephydridae|GMPJA5220-21|BIOUG66608-H06|BOLD:ACO4648
Ephydridae|GMPJA5233-21|BIOUG66609-A08|BOLD:ACO4648
Ephydridae|GMPJA5249-21|BIOUG66609-B12|BOLD:ACO4648
Ephydridae|GMPJA5291-21|BIOUG66609-F06|BOLD:ACO4648
Ephydridae|GMPJA5516-21|BIOUG66612-A06|BOLD:ACO4648
Ephydridae|GMPJA5552-21|BIOUG66612-D06|BOLD:ACO4648
Ephydridae|GMPJA6728-21|BIOUG66624-G06|BOLD:ACO4648
Ephydridae|GMPJA6557-21|BIOUG66623-A02|BOLD:ACO4648
Ephydridae|GMPJA6633-21|BIOUG66623-G06|BOLD:ACO4648
Ephydridae|GMPJA6697-21|BIOUG66624-D11|BOLD:ACO4648
Ephydridae|GMPJA6838-21|BIOUG66625-H09|BOLD:ACO4648
Ephydridae|GMPJA6888-21|BIOUG66626-D12|BOLD:ACO4648
Ephydridae|GMPJA7755-21|BIOUG66635-E12|BOLD:ACO4648
Ephydridae|GMPJA8111-21|BIOUG66639-C12|BOLD:ACO4648
Ephydridae|GMPJA8242-21|BIOUG66640-F12|BOLD:ACO4648
Ephydridae|GMPJA8372-21|BIOUG66642-A12|BOLD:ACO4648
Ephydridae|GMPJA10367-21|BIOUG70233-H02|BOLD:ACO4648
Ephydridae|GMPJA10216-21|BIOUG70232-C06|BOLD:ACO4648
Ephydridae|GMPJA4563-21|BIOUG66603-A03|BOLD:ACO4648
Ephydridae|GMPJA10294-21|BIOUG70233-B01|BOLD:ACO4648
Ephydridae|GMPJA4378-21|BIOUG66601-A08|BOLD:ACO4648
Ephydridae|GMPJA400-21|BIOUG65659-F06|BOLD:ACO4648
Ephydridae|GMPJA498-21|BIOUG65660-F09|BOLD:ACO4648
Ephydridae|GMPJA511-21|BIOUG65660-G10|BOLD:ACO4648
Ephydridae|GMPJA335-21|BIOUG65659-A01|BOLD:ACO4648
Ephydridae|GMPJA347-21|BIOUG65659-B01|BOLD:ACO4648
Ephydridae|GMPJA10241-21|BIOUG70232-E07|BOLD:ACO4648
Ephydridae|GMPJA930-21|BIOUG65665-C02|BOLD:ACO4648
Ephydridae|GMPJA5356-21|BIOUG66610-C12|BOLD:ACO4648
Ephydridae|GMPJA1644-21|BIOUG65672-G03|BOLD:ABX2830
Ephydridae|GMPJA4518-21|BIOUG66602-E05|BOLD:AEJ3334
Ephydridae|GMPJA4030-21|BIOUG65697-D04|BOLD:AEJ3334
Ephydridae|GMPJA10562-21|BIOUG70235-H07|BOLD:AEJ3334
Ephydridae|GMPJA8794-21|BIOUG66646-E06|BOLD:AEJ3334
Ephydridae|GMPJA8587-21|BIOUG66644-D01|BOLD:AEJ3334
Ephydridae|GMPJA4199-21|BIOUG65699-B07|BOLD:AEJ3334
Ephydridae|GMPJA7817-21|BIOUG66636-C03|BOLD:AEJ3334
Ephydridae|GMPJA5548-21|BIOUG66612-D02|BOLD:ABY1193
Ephydridae|GMPJA2307-21|BIOUG65679-G01|BOLD:ABY1193
Ephydridae|GMPJA10662-21|BIOUG70237-A01|BOLD:ABY1193
Ephydridae|GMPJA8117-21|BIOUG66639-D06|BOLD:ABY1193
Ephydridae|GMPJA7261-21|BIOUG66630-D05|BOLD:ABY1193
Ephydridae|GMPJA6528-21|BIOUG66622-F08|BOLD:ABY1193
Ephydridae|GMPJA5589-21|BIOUG66612-G07|BOLD:ABY1193
Ephydridae|GMPJA4140-21|BIOUG65698-E07|BOLD:ABY1193
Ephydridae|GMPJA8472-21|BIOUG66643-B05|BOLD:ABY1193
Ephydridae|GMPJA8332-21|BIOUG66641-F07|BOLD:ABY1193
Ephydridae|GMPJA5245-21|BIOUG66609-B08|BOLD:ABY1193
Ephydridae|GMPJA8377-21|BIOUG66642-B05|BOLD:ABY1193
Ephydridae|GMPJA5168-21|BIOUG66608-D02|BOLD:ABY1193
Ephydridae|GMPJA4384-21|BIOUG66601-B02|BOLD:ABY1193
Ephydridae|GMPJA4114-21|BIOUG65698-C05|BOLD:ABY1193
Ephydridae|GMPJA4524-21|BIOUG66602-E11|BOLD:ABY1193
Ephydridae|GMPJA6489-21|BIOUG66622-C05|BOLD:ABY1193
Ephydridae|GMPJA8501-21|BIOUG66643-D10|BOLD:ABY1193
Ephydridae|GMPJA5451-21|BIOUG66611-C12|BOLD:ABY1193
Ephydridae|GMPJA6701-21|BIOUG66624-E03|BOLD:ABY1193
Ephydridae|GMPJA7654-21|BIOUG66634-E06|BOLD:ABY1193
Ephydridae|GMPJA5240-21|BIOUG66609-B03|BOLD:ABY1193
Ephydridae|GMPJA5169-21|BIOUG66608-D03|BOLD:ABY1193
Ephydridae|GMPJA8523-21|BIOUG66643-F08|BOLD:ABY1193
Ephydridae|GMPJA7354-21|BIOUG66631-D03|BOLD:ABY1193
Ephydridae|GMPJA10539-21|BIOUG70235-F08|BOLD:ABY1193
Ephydridae|GMPJA8735-21|BIOUG66645-H06|BOLD:ABY1193
Ephydridae|GMPJA8630-21|BIOUG66644-G08|BOLD:ABY1193
Ephydridae|GMPJA6741-21|BIOUG66624-H07|BOLD:ABY1193
Ephydridae|GMPJA5148-21|BIOUG66608-B06|BOLD:ABY1193
Ephydridae|GMPJA5449-21|BIOUG66611-C10|BOLD:ABY1193
Ephydridae|GMPJA4373-21|BIOUG66601-A03|BOLD:ABY1193
Ephydridae|GMPJA5058-21|BIOUG66607-B11|BOLD:ABY1193
Ephydridae|GMPJA5042-21|BIOUG66607-A07|BOLD:ABY1193
Mycodrosophila aqua|GMPJA8636-21|BIOUG66644-H02|BOLD:AFC0769
Drosophilidae|GMPJA7376-21|BIOUG66631-F01|BOLD:ACG8597
Drosophila simulans|GMPJA1481-21|BIOUG65671-A07|BOLD:AAE8098
Drosophila simulans|GMPJA1969-21|BIOUG65676-B08|BOLD:AAE8098
Drosophila simulans|GMPJA1526-21|BIOUG65671-E04|BOLD:AAE8098
Drosophila simulans|GMPJA1757-21|BIOUG65673-H09|BOLD:AAE8098
Drosophila simulans|GMPJA1762-21|BIOUG65674-A03|BOLD:AAE8098
Drosophila simulans|GMPJA1808-21|BIOUG65674-E01|BOLD:AAE8098
Drosophila simulans|GMPJA1410-21|BIOUG65670-C07|BOLD:AAE8098
Drosophila simulans|GMPJA1349-21|BIOUG65669-F05|BOLD:AAE8098
Drosophila takahashii|GMPJA3319-21|BIOUG65689-H05|BOLD:AAU0289
Drosophila|GMPJA1285-21|BIOUG65669-A01|BOLD:ACE9640

Drosophila simulans|GMPJA1349-21|BIOUG65669-F05|BOLD:AAE8098
Drosophila takahashii|GMPJA3319-21|BIOUG65689-H05|BOLD:AAU0289
Drosophila|GMPJA1285-21|BIOUG65669-A01|BOLD:ACE9640
Drosophila|GMPJA2448-21|BIOUG65681-B12|BOLD:ACE9640
Drosophila|GMPJA2444-21|BIOUG65681-B08|BOLD:ACE9640
Sarcophagidae|GMPJA4360-21|BIOUG65700-H01|BOLD:AEJ8819
Sarcophagidae|GMPJA5362-21|BIOUG66610-D06|BOLD:ACA1194
Sarcophagidae|GMPJA4123-21|BIOUG65698-D02|BOLD:ACA1194
Sarcophagidae|GMPJA5672-21|BIOUG66613-F07|BOLD:ACA1194
Sarcophagidae|GMPJA6769-21|BIOUG66625-B12|BOLD:ACA1194
Sarcophagidae|GMPJA5223-21|BIOUG66608-H09|BOLD:ACA1194
Sarcophagidae|GMPJA5386-21|BIOUG66610-F06|BOLD:ACA1194
Sarcophagidae|GMPJA5315-21|BIOUG66609-H06|BOLD:ACA1194
Sarcophagidae|GMPJA7221-21|BIOUG66630-A01|BOLD:ACA1194
Sarcophagidae|GMPJA6366-21|BIOUG66621-A01|BOLD:ACA1194
Sarcophagidae|GMPJA6371-21|BIOUG66621-A06|BOLD:ACA1194
Sarcophagidae|GMPJA6502-21|BIOUG66622-D06|BOLD:ACA1194
Sarcophagidae|GMPJA6628-21|BIOUG66623-G01|BOLD:ACA1194
Sarcophagidae|GMPJA6640-21|BIOUG66623-H01|BOLD:ACA1194
Sarcophagidae|GMPJA6711-21|BIOUG66624-F01|BOLD:ACA1194
Sarcophagidae|GMPJA6817-21|BIOUG66625-F12|BOLD:ACA1194
Sarcophagidae|GMPJA7685-21|BIOUG66634-H01|BOLD:ACA1194
Sarcophagidae|GMPJA7851-21|BIOUG66636-F01|BOLD:ACA1194
Sarcophagidae|GMPJA3135-21|BIOUG65687-H11|BOLD:ACA1194
Sarcophagidae|GMPJA10103-21|BIOUG66563-B01|BOLD:ACA1194
Sarcophagidae|GMPJA10162-21|BIOUG66563-F12|BOLD:ACA1194
Sarcophagidae|GMPJA4502-21|BIOUG66602-D01|BOLD:ACA1194
Sarcophagidae|GMPJA4276-21|BIOUG65700-A01|BOLD:ACA1194
Sarcophagidae|GMPJA4394-21|BIOUG66601-B12|BOLD:ACA1194
Sarcophagidae|GMPJA3616-21|BIOUG65693-A06|BOLD:ACA1194
Sarcophagidae|GMPJA4063-21|BIOUG65697-G01|BOLD:ACA1194
Sarcophagidae|GMPJA4085-21|BIOUG65697-H11|BOLD:ACA1194
Sarcophagidae|GMPJA4371-21|BIOUG66601-A01|BOLD:ACA1194
Sarcophagidae|GMPJA6794-21|BIOUG66625-E01|BOLD:ACA1194
Sarcophagidae|GMPJA6282-21|BIOUG66620-A12|BOLD:ACA1194
Sarcophagidae|GMPJA6266-21|BIOUG66619-H07|BOLD:ACA1194
Sarcophagidae|GMPJA6743-21|BIOUG66624-H09|BOLD:ACA1194
Sarcophagidae|GMPJA6197-21|BIOUG66619-B10|BOLD:ACA1194
Sarcophagidae|GMPJA5893-21|BIOUG66616-A03|BOLD:ACA1194
Sarcophagidae|GMPJA5892-21|BIOUG66616-A02|BOLD:ACA1194
Sarcophagidae|GMPJA5677-21|BIOUG66613-F12|BOLD:ACA1194
Sarcophagidae|GMPJA5675-21|BIOUG66613-F10|BOLD:ACA1194
Sarcophagidae|GMPJA5673-21|BIOUG66613-F08|BOLD:ACA1194
Sarcophagidae|GMPJA5671-21|BIOUG66613-F06|BOLD:ACA1194
Sarcophagidae|GMPJA5670-21|BIOUG66613-F05|BOLD:ACA1194
Sarcophagidae|GMPJA5155-21|BIOUG66608-C01|BOLD:ACA1194
Sarcophagidae|GMPJA5120-21|BIOUG66607-H01|BOLD:ACA1194
Sarcophagidae|GMPJA5345-21|BIOUG66610-C01|BOLD:ACA1194
Sarcophagidae|GMPJA6650-21|BIOUG66623-H11|BOLD:ACA1194
Sarcophagidae|GMPJA6791-21|BIOUG66625-D10|BOLD:ACA1194
Sarcophagidae|GMPJA6846-21|BIOUG66626-A06|BOLD:ACA1194
Sarcophagidae|GMPJA8243-21|BIOUG66640-G01|BOLD:ACA1194
Sarcophagidae|GMPJA8657-21|BIOUG66645-A12|BOLD:ACA1194
Sarcophagidae|GMPJA10164-21|BIOUG66563-G02|BOLD:ACA1194
Sarcophagidae|GMPJA4572-21|BIOUG66603-A12|BOLD:ACA1194
Sarcophagidae|GMPJA1333-21|BIOUG65669-E01|BOLD:ACA1194
Sarcophagidae|GMPJA6505-21|BIOUG66622-D09|BOLD:ACA1194
Sarcophagidae|GMPJA6365-21|BIOUG66620-H11|BOLD:ACA1194
Sarcophagidae|GMPJA5129-21|BIOUG66607-H10|BOLD:ACA1194
Sarcophagidae|GMPJA5083-21|BIOUG66607-D12|BOLD:ACA1194
Sarcophagidae|GMPJA5381-21|BIOUG66610-F01|BOLD:ACA1194
Sarcophagidae|GMPJA5065-21|BIOUG66607-C06|BOLD:ACA1194
Sarcophagidae|GMPJA5676-21|BIOUG66613-F11|BOLD:ACA1194
Sarcophagidae|GMPJA5113-21|BIOUG66607-G06|BOLD:ACA1194
Sarcophagidae|GMPJA6259-21|BIOUG66619-G12|BOLD:ACA1194
Sarcophagidae|GMPJA6362-21|BIOUG66620-H08|BOLD:ACA1194
Sarcophagidae|GMPJA6279-21|BIOUG66620-A09|BOLD:ACA1194
Sarcophagidae|GMPJA6740-21|BIOUG66624-H06|BOLD:ACA1194
Sarcophagidae|GMPJA6195-21|BIOUG66619-B08|BOLD:ACA1194
Sarcophagidae|GMPJA6364-21|BIOUG66620-H10|BOLD:ACA1194
Sarcophagidae|GMPJA6363-21|BIOUG66620-H09|BOLD:ACA1194
Sarcophagidae|GMPJA7393-21|BIOUG66631-G06|BOLD:ACA1194
Sarcophagidae|GMPJA6597-21|BIOUG66623-D06|BOLD:ACA1194
Sarcophagidae|GMPJA7352-21|BIOUG66631-D01|BOLD:ACA1194
Sarcophagidae|GMPJA6908-21|BIOUG66626-F08|BOLD:ACA1194
Sarcophagidae|GMPJA8088-21|BIOUG66639-B01|BOLD:ACA1194
Sarcophagidae|GMPJA7998-21|BIOUG66638-B06|BOLD:ACA1194
Sarcophagidae|GMPJA10106-21|BIOUG66563-B04|BOLD:ACA1194
Sarcophagidae|GMPJA7553-21|BIOUG66633-D12|BOLD:ACA1194
Sarcophagidae|GMPJA10163-21|BIOUG66563-G01|BOLD:ACA1194
Sarcophagidae|GMPJA10109-21|BIOUG66563-B07|BOLD:ACA1194
Sarcophagidae|GMPJA6852-21|BIOUG66626-A12|BOLD:ACA1194
Sarcophagidae|GMPJA6835-21|BIOUG66625-H06|BOLD:ACA1194
Sarcophagidae|GMPJA10167-21|BIOUG66563-G05|BOLD:ACA1194
Sarcophagidae|GMPJA10165-21|BIOUG66563-G03|BOLD:ACA1194
Sarcophagidae|GMPJA3504-21|BIOUG65691-G12|BOLD:ACA1194
Sarcophagidae|GMPJA4181-21|BIOUG65699-A01|BOLD:ACA1194
Sarcophagidae|GMPJA4515-21|BIOUG66602-E02|BOLD:ACA1194
Sarcophagidae|GMPJA3029-21|BIOUG65686-G12|BOLD:ACA1194
Sarcophagidae|GMPXX030-21|BIOUG60809-D01|BOLD:ACA1194
Sarcophagidae|GMPXX137-21|BIOUG60810-D09|BOLD:ACA1194
Sarcophagidae|GMPXX138-21|BIOUG60810-D10|BOLD:ACA1194
Sarcophagidae|GMPXX139-21|BIOUG60810-D11|BOLD:ACA1194
Sarcophagidae|GMPXX026-21|BIOUG60809-C09|BOLD:ACA1194
Sarcophagidae|GMPXX022-21|BIOUG60809-C05|BOLD:ACA1194
Sarcophagidae|GMPJA4344-21|BIOUG65700-F09|BOLD:ACA1194
Sarcophagidae|GMPJA4347-21|BIOUG65700-F12|BOLD:ACA1194
Sarcophagidae|GMPJA3385-21|BIOUG65690-E12|BOLD:ACA1194
Sarcophagidae|GMPJA8740-21|BIOUG66645-H11|BOLD:ACA1194
Sarcophagidae|GMPJA6871-21|BIOUG66626-C07|BOLD:ACA1194
Sarcophagidae|GMPJA6526-21|BIOUG66622-F06|BOLD:ACA1194
Sarcophagidae|GMPJA6431-21|BIOUG66621-F06|BOLD:ACA1194
Sarcophagidae|GMPJA6443-21|BIOUG66621-G06|BOLD:ACA1194

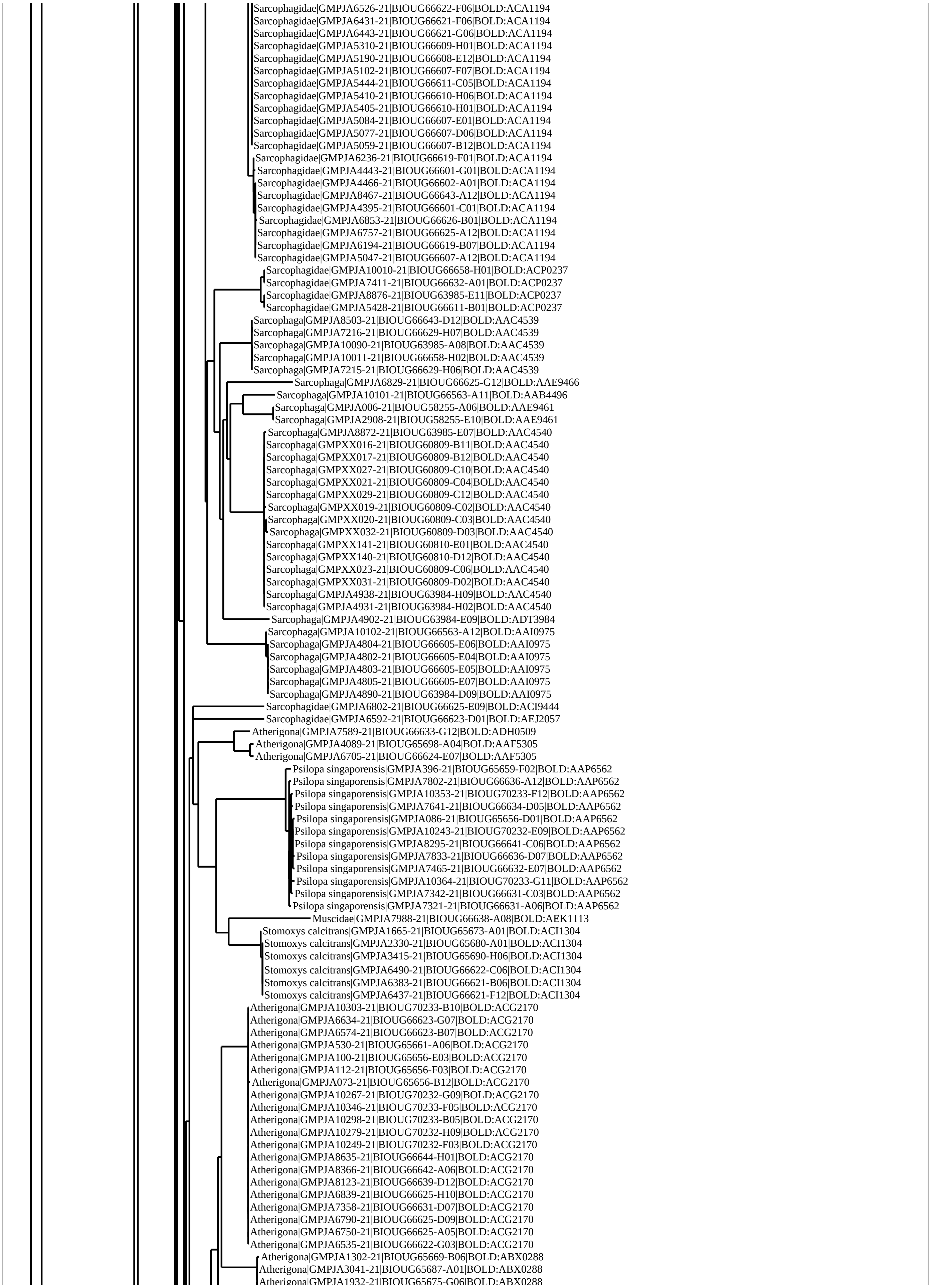
Sarcophagidae|GMPJA6526-21|BIOUG66622-F06|BOLD:ACA1194
Sarcophagidae|GMPJA6431-21|BIOUG66621-F06|BOLD:ACA1194
Sarcophagidae|GMPJA6443-21|BIOUG66621-G06|BOLD:ACA1194
Sarcophagidae|GMPJA5310-21|BIOUG66609-H01|BOLD:ACA1194
Sarcophagidae|GMPJA5190-21|BIOUG66608-E12|BOLD:ACA1194
Sarcophagidae|GMPJA5102-21|BIOUG66607-F07|BOLD:ACA1194
Sarcophagidae|GMPJA5444-21|BIOUG66611-C05|BOLD:ACA1194
Sarcophagidae|GMPJA5410-21|BIOUG66610-H06|BOLD:ACA1194
Sarcophagidae|GMPJA5405-21|BIOUG66610-H01|BOLD:ACA1194
Sarcophagidae|GMPJA5084-21|BIOUG66607-E01|BOLD:ACA1194
Sarcophagidae|GMPJA5077-21|BIOUG66607-D06|BOLD:ACA1194
Sarcophagidae|GMPJA5059-21|BIOUG66607-B12|BOLD:ACA1194
Sarcophagidae|GMPJA6236-21|BIOUG66619-F01|BOLD:ACA1194
Sarcophagidae|GMPJA4443-21|BIOUG66601-G01|BOLD:ACA1194
Sarcophagidae|GMPJA4466-21|BIOUG66602-A01|BOLD:ACA1194
Sarcophagidae|GMPJA8467-21|BIOUG66643-A12|BOLD:ACA1194
Sarcophagidae|GMPJA4395-21|BIOUG66601-C01|BOLD:ACA1194
Sarcophagidae|GMPJA6853-21|BIOUG66626-B01|BOLD:ACA1194
Sarcophagidae|GMPJA6757-21|BIOUG66625-A12|BOLD:ACA1194
Sarcophagidae|GMPJA6194-21|BIOUG66619-B07|BOLD:ACA1194
Sarcophagidae|GMPJA5047-21|BIOUG66607-A12|BOLD:ACA1194
Sarcophagidae|GMPJA10010-21|BIOUG66658-H01|BOLD:ACP0237
Sarcophagidae|GMPJA7411-21|BIOUG66632-A01|BOLD:ACP0237
Sarcophagidae|GMPJA8876-21|BIOUG63985-E11|BOLD:ACP0237
Sarcophagidae|GMPJA5428-21|BIOUG66611-B01|BOLD:ACP0237
Sarcophaga|GMPJA8503-21|BIOUG66643-D12|BOLD:AAC4539
Sarcophaga|GMPJA7216-21|BIOUG66629-H07|BOLD:AAC4539
Sarcophaga|GMPJA10090-21|BIOUG63985-A08|BOLD:AAC4539
Sarcophaga|GMPJA10011-21|BIOUG66658-H02|BOLD:AAC4539
Sarcophaga|GMPJA7215-21|BIOUG66629-H06|BOLD:AAC4539
Sarcophaga|GMPJA6829-21|BIOUG66625-G12|BOLD:AAE9466
Sarcophaga|GMPJA10101-21|BIOUG66563-A11|BOLD:AAB4496
Sarcophaga|GMPJA006-21|BIOUG58255-A06|BOLD:AAE9461
Sarcophaga|GMPJA2908-21|BIOUG58255-E10|BOLD:AAE9461
Sarcophaga|GMPJA8872-21|BIOUG63985-E07|BOLD:AAC4540
Sarcophaga|GMPXX016-21|BIOUG60809-B11|BOLD:AAC4540
Sarcophaga|GMPXX017-21|BIOUG60809-B12|BOLD:AAC4540
Sarcophaga|GMPXX027-21|BIOUG60809-C10|BOLD:AAC4540
Sarcophaga|GMPXX021-21|BIOUG60809-C04|BOLD:AAC4540
Sarcophaga|GMPXX029-21|BIOUG60809-C12|BOLD:AAC4540
Sarcophaga|GMPXX019-21|BIOUG60809-C02|BOLD:AAC4540
Sarcophaga|GMPXX020-21|BIOUG60809-C03|BOLD:AAC4540
Sarcophaga|GMPXX032-21|BIOUG60809-D03|BOLD:AAC4540
Sarcophaga|GMPXX141-21|BIOUG60810-E01|BOLD:AAC4540
Sarcophaga|GMPXX140-21|BIOUG60810-D12|BOLD:AAC4540
Sarcophaga|GMPXX023-21|BIOUG60809-C06|BOLD:AAC4540
Sarcophaga|GMPXX031-21|BIOUG60809-D02|BOLD:AAC4540
Sarcophaga|GMPJA4938-21|BIOUG63984-H09|BOLD:AAC4540
Sarcophaga|GMPJA4931-21|BIOUG63984-H02|BOLD:AAC4540
Sarcophaga|GMPJA4902-21|BIOUG63984-E09|BOLD:ADT3984
Sarcophaga|GMPJA10102-21|BIOUG66563-A12|BOLD:AAI0975
Sarcophaga|GMPJA4804-21|BIOUG66605-E06|BOLD:AAI0975
Sarcophaga|GMPJA4802-21|BIOUG66605-E04|BOLD:AAI0975
Sarcophaga|GMPJA4803-21|BIOUG66605-E05|BOLD:AAI0975
Sarcophaga|GMPJA4805-21|BIOUG66605-E07|BOLD:AAI0975
Sarcophaga|GMPJA4890-21|BIOUG63984-D09|BOLD:AAI0975
Sarcophagidae|GMPJA6802-21|BIOUG66625-E09|BOLD:ACI9444
Sarcophagidae|GMPJA6592-21|BIOUG66623-D01|BOLD:AEJ2057
Atherigona|GMPJA7589-21|BIOUG66633-G12|BOLD:ADH0509
Atherigona|GMPJA4089-21|BIOUG65698-A04|BOLD:AAF5305
Atherigona|GMPJA6705-21|BIOUG66624-E07|BOLD:AAF5305
Psilopa singaporensis|GMPJA396-21|BIOUG65659-F02|BOLD:AAP6562
Psilopa singaporensis|GMPJA7802-21|BIOUG66636-A12|BOLD:AAP6562
Psilopa singaporensis|GMPJA10353-21|BIOUG70233-F12|BOLD:AAP6562
Psilopa singaporensis|GMPJA7641-21|BIOUG66634-D05|BOLD:AAP6562
Psilopa singaporensis|GMPJA086-21|BIOUG65656-D01|BOLD:AAP6562
Psilopa singaporensis|GMPJA10243-21|BIOUG70232-E09|BOLD:AAP6562
Psilopa singaporensis|GMPJA8295-21|BIOUG66641-C06|BOLD:AAP6562
Psilopa singaporensis|GMPJA7833-21|BIOUG66636-D07|BOLD:AAP6562
Psilopa singaporensis|GMPJA7465-21|BIOUG66632-E07|BOLD:AAP6562
Psilopa singaporensis|GMPJA10364-21|BIOUG70233-G11|BOLD:AAP6562
Psilopa singaporensis|GMPJA7342-21|BIOUG66631-C03|BOLD:AAP6562
Psilopa singaporensis|GMPJA7321-21|BIOUG66631-A06|BOLD:AAP6562
Muscidae|GMPJA7988-21|BIOUG66638-A08|BOLD:AEK1113
Stomoxys calcitrans|GMPJA1665-21|BIOUG65673-A01|BOLD:ACI1304
Stomoxys calcitrans|GMPJA2330-21|BIOUG65680-A01|BOLD:ACI1304
Stomoxys calcitrans|GMPJA3415-21|BIOUG65690-H06|BOLD:ACI1304
Stomoxys calcitrans|GMPJA6490-21|BIOUG66622-C06|BOLD:ACI1304
Stomoxys calcitrans|GMPJA6383-21|BIOUG66621-B06|BOLD:ACI1304
Stomoxys calcitrans|GMPJA6437-21|BIOUG66621-F12|BOLD:ACI1304
Atherigona|GMPJA10303-21|BIOUG70233-B10|BOLD:ACG2170
Atherigona|GMPJA6634-21|BIOUG66623-G07|BOLD:ACG2170
Atherigona|GMPJA6574-21|BIOUG66623-B07|BOLD:ACG2170
Atherigona|GMPJA530-21|BIOUG65661-A06|BOLD:ACG2170
Atherigona|GMPJA100-21|BIOUG65656-E03|BOLD:ACG2170
Atherigona|GMPJA112-21|BIOUG65656-F03|BOLD:ACG2170
Atherigona|GMPJA073-21|BIOUG65656-B12|BOLD:ACG2170
Atherigona|GMPJA10267-21|BIOUG70232-G09|BOLD:ACG2170
Atherigona|GMPJA10346-21|BIOUG70233-F05|BOLD:ACG2170
Atherigona|GMPJA10298-21|BIOUG70233-B05|BOLD:ACG2170
Atherigona|GMPJA10279-21|BIOUG70232-H09|BOLD:ACG2170
Atherigona|GMPJA10249-21|BIOUG70232-F03|BOLD:ACG2170
Atherigona|GMPJA8635-21|BIOUG66644-H01|BOLD:ACG2170
Atherigona|GMPJA8366-21|BIOUG66642-A06|BOLD:ACG2170
Atherigona|GMPJA8123-21|BIOUG66639-D12|BOLD:ACG2170
Atherigona|GMPJA6839-21|BIOUG66625-H10|BOLD:ACG2170
Atherigona|GMPJA7358-21|BIOUG66631-D07|BOLD:ACG2170
Atherigona|GMPJA6790-21|BIOUG66625-D09|BOLD:ACG2170
Atherigona|GMPJA6750-21|BIOUG66625-A05|BOLD:ACG2170
Atherigona|GMPJA6535-21|BIOUG66622-G03|BOLD:ACG2170
Atherigona|GMPJA1302-21|BIOUG65669-B06|BOLD:ABX0288
Atherigona|GMPJA3041-21|BIOUG65687-A01|BOLD:ABX0288
Atherigona|GMPJA1932-21|BIOUG65675-G06|BOLD:ABX0288

Atherigona|GMPJA1302-21|BIOUG65669-B06|BOLD:ABX0288
Atherigona|GMPJA3041-21|BIOUG65687-A01|BOLD:ABX0288
Atherigona|GMPJA1932-21|BIOUG65675-G06|BOLD:ABX0288
Atherigona|GMPJA1688-21|BIOUG65673-B12|BOLD:ABX0288
Atherigona|GMPJA5499-21|BIOUG66611-G12|BOLD:ABX0288
Muscidae|GMPJA6521-21|BIOUG66622-F01|BOLD:ACV1193
Atherigona reversura|GMPJA6797-21|BIOUG66625-E04|BOLD:AAN8579
Atherigona reversura|GMPJA6719-21|BIOUG66624-F09|BOLD:AAN8579
Atherigona reversura|GMPJA2357-21|BIOUG65680-C04|BOLD:AAN8579
Atherigona reversura|GMPJA6663-21|BIOUG66624-B01|BOLD:AAN8579
Atherigona reversura|GMPJA6753-21|BIOUG66625-A08|BOLD:AAN8579
Atherigona reversura|GMPJA8147-21|BIOUG66639-F12|BOLD:AAN8579
Atherigona reversura|GMPJA336-21|BIOUG65659-A02|BOLD:AAN8579
Atherigona reversura|GMPJA6484-21|BIOUG66622-B12|BOLD:AAN8579
Atherigona reversura|GMPJA6414-21|BIOUG66621-E01|BOLD:AAN8579
Atherigona reversura|GMPJA3278-21|BIOUG65689-D12|BOLD:AAN8579
Atherigona reversura|GMPJA3359-21|BIOUG65690-C10|BOLD:AAN8579
Atherigona reversura|GMPJA922-21|BIOUG65665-B06|BOLD:AAN8579
Atherigona reversura|GMPJA2389-21|BIOUG65680-E12|BOLD:AAN8579
Atherigona reversura|GMPJA691-21|BIOUG65662-F12|BOLD:AAN8579
Atherigona reversura|GMPJA6576-21|BIOUG66623-B09|BOLD:AAN8579
Atherigona reversura|GMPJA10269-21|BIOUG70232-G11|BOLD:AAN8579
Atherigona reversura|GMPJA6804-21|BIOUG66625-E11|BOLD:AAN8579
Atherigona reversura|GMPJA6436-21|BIOUG66621-F11|BOLD:AAN8579
Atherigona reversura|GMPJA6463-21|BIOUG66622-A03|BOLD:AAN8579
Atherigona reversura|GMPJA6542-21|BIOUG66622-G10|BOLD:AAN8579
Atherigona reversura|GMPJA6690-21|BIOUG66624-D04|BOLD:AAN8579
Atherigona reversura|GMPJA6700-21|BIOUG66624-E02|BOLD:AAN8579
Atherigona reversura|GMPJA7856-21|BIOUG66636-F06|BOLD:AAN8579
Atherigona reversura|GMPJA8479-21|BIOUG66643-B12|BOLD:AAN8579
Atherigona reversura|GMPJA3360-21|BIOUG65690-C11|BOLD:AAN8579
Atherigona reversura|GMPJA418-21|BIOUG65659-G12|BOLD:AAN8579
Atherigona reversura|GMPJA8679-21|BIOUG66645-C10|BOLD:AAN8579
Atherigona reversura|GMPJA6763-21|BIOUG66625-B06|BOLD:AAN8579
Atherigona reversura|GMPJA5343-21|BIOUG66610-B11|BOLD:AAN8579
Atherigona|GMPJA2008-21|BIOUG65676-E11|BOLD:ABX4632
Atherigona|GMPJA6462-21|BIOUG66622-A02|BOLD:AAG1745
Atherigona|GMPJA6445-21|BIOUG66621-G08|BOLD:AAG1745
Muscidae|GMPJA10325-21|BIOUG70233-D08|BOLD:ACV8099
Atherigona|GMPJA6498-21|BIOUG66622-D02|BOLD:ACJ5809
Atherigona|GMPJA5463-21|BIOUG66611-D12|BOLD:ACJ5809
Atherigona|GMPJA5427-21|BIOUG66611-A12|BOLD:ACJ5809
Atherigona|GMPJA7600-21|BIOUG66633-H11|BOLD:ACJ5809
Atherigona|GMPJA5075-21|BIOUG66607-D04|BOLD:ACJ5809
Orchisia costata|GMPJA1469-21|BIOUG65670-H06|BOLD:ABX0213
Orchisia costata|GMPJA10527-21|BIOUG70235-E08|BOLD:ABX0213
Diptera|GMPJA2187-21|BIOUG65678-D12
Limnophora|GMPJA6901-21|BIOUG66626-F01|BOLD:ACN3496
Limnophora|GMPJA6695-21|BIOUG66624-D09|BOLD:ACN3496
Limnophora|GMPJA6623-21|BIOUG66623-F08|BOLD:ACN3496
Limnophora|GMPJA6416-21|BIOUG66621-E03|BOLD:ACN3496
Melanostoma fasciatum|GMPJA3634-21|BIOUG65693-B12|BOLD:ABY6279
Melanostoma fasciatum|GMPJA1926-21|BIOUG65675-F12|BOLD:ABY6279
Melanostoma fasciatum|GMPJA168-21|BIOUG65657-B12|BOLD:ABY6279
Melanostoma fasciatum|GMPJA3308-21|BIOUG65689-G06|BOLD:ABY6279
Sarcophagidae|GMPJA10278-21|BIOUG70232-H08|BOLD:AEL5012
Tachinidae|GMPJA6885-21|BIOUG66626-D09|BOLD:ACU7511
Hemipyrellia|GMPJA005-21|BIOUG58255-A05|BOLD:AAE4423
Hemipyrellia|GMPJA8871-21|BIOUG63985-E06|BOLD:AAE4423
Sarcophagidae|GMPJA4056-21|BIOUG65697-F06|BOLD:ACB4981
Sarcophagidae|GMPJA4806-21|BIOUG66605-E08|BOLD:ACB4981
Sarcophagidae|GMPJA7506-21|BIOUG66633-A01|BOLD:ACB4981
Sarcophagidae|GMPJA6735-21|BIOUG66624-H01|BOLD:ACB4981
Sarcophagidae|GMPJA4075-21|BIOUG65697-H01|BOLD:ACB4981
Sarcophagidae|GMPJA6561-21|BIOUG66623-A06|BOLD:ACB4981
Sarcophagidae|GMPJA5674-21|BIOUG66613-F09|BOLD:ACB4981
Sarcophagidae|GMPJA5332-21|BIOUG66610-A12|BOLD:ACB4981
Sarcophagidae|GMPJA5071-21|BIOUG66607-C12|BOLD:ACB4981
Anthomyia illocata|GMPJA1392-21|BIOUG65670-B01|BOLD:ACD8455
Anthomyia illocata|GMPJA068-21|BIOUG65656-B07|BOLD:ACD8455
Anthomyia illocata|GMPJA3090-21|BIOUG65687-E02|BOLD:ACD8455
Calliphoridae|GMPJA4465-21|BIOUG66601-H11|BOLD:ACA4119
Calliphoridae|GMPJA10115-21|BIOUG66563-C01|BOLD:ACA4119
Calliphoridae|GMPJA10104-21|BIOUG66563-B02|BOLD:ACA4119
Calliphoridae|GMPJA6793-21|BIOUG66625-D12|BOLD:ACA4119
Calliphoridae|GMPJA6770-21|BIOUG66625-C01|BOLD:ACA4119
Bengalia|GMPJA6734-21|BIOUG66624-G12|BOLD:ACX7944
Anthomyiidae|GMPJA536-21|BIOUG65661-A12|BOLD:AAZ4294
Anthomyiidae|GMPJA1059-21|BIOUG65666-E12|BOLD:AAZ4294
Anthomyiidae|GMPJA1060-21|BIOUG65666-F01|BOLD:AAZ4294
Anthomyiidae|GMPJA1089-21|BIOUG65666-H06|BOLD:AAZ4294
Anthomyiidae|GMPJA1130-21|BIOUG65667-C12|BOLD:AAZ4294
Anthomyiidae|GMPJA1356-21|BIOUG65669-F12|BOLD:AAZ4294
Anthomyiidae|GMPJA2224-21|BIOUG65678-H01|BOLD:AAZ4294
Anthomyiidae|GMPJA2306-21|BIOUG65679-F12|BOLD:AAZ4294
Anthomyiidae|GMPJA2335-21|BIOUG65680-A06|BOLD:AAZ4294
Anthomyiidae|GMPJA2140-21|BIOUG65678-A01|BOLD:AAZ4294
Anthomyiidae|GMPJA1040-21|BIOUG65666-D05|BOLD:AAZ4294
Anthomyiidae|GMPJA3444-21|BIOUG65691-B12|BOLD:AAZ4294
Anthomyiidae|GMPJA156-21|BIOUG65657-A12|BOLD:AAZ4294
Anthomyiidae|GMPJA079-21|BIOUG65656-C06|BOLD:AAZ4294
Anthomyiidae|GMPJA069-21|BIOUG65656-B08|BOLD:AAZ4294
Anthomyiidae|GMPJA514-21|BIOUG65660-H01|BOLD:AAZ4294
Anthomyiidae|GMPJA441-21|BIOUG65660-A12|BOLD:AAZ4294
Anthomyiidae|GMPJA3609-21|BIOUG65692-H10|BOLD:AAZ4294
Anthomyiidae|GMPJA3539-21|BIOUG65692-B12|BOLD:AAZ4294
Anthomyiidae|GMPJA3433-21|BIOUG65691-B01|BOLD:AAZ4294
Anthomyiidae|GMPJA3367-21|BIOUG65690-D06|BOLD:AAZ4294
Anthomyiidae|GMPJA3207-21|BIOUG65688-F12|BOLD:AAZ4294
Anthomyiidae|GMPJA715-21|BIOUG65663-A01|BOLD:AAZ4294
Anthomyiidae|GMPJA1297-21|BIOUG65669-B01|BOLD:AAZ4294
Anthomyiidae|GMPJA3343-21|BIOUG65690-B06|BOLD:AAZ4294
Anthomyiidae|GMPJA2398-21|BIOUG65680-F09|BOLD:AAZ4294

[illegible]
Anthomyiidae|GMPJA1297-21|BIOUG65669-B01|BOLD:AAZ4294
Anthomyiidae|GMPJA3343-21|BIOUG65690-B06|BOLD:AAZ4294
Anthomyiidae|GMPJA2398-21|BIOUG65680-F09|BOLD:AAZ4294
Anthomyiidae|GMPJA989-21|BIOUG65665-H01|BOLD:AAZ4294
Anthomyiidae|GMPJA3337-21|BIOUG65690-A12|BOLD:AAZ4294
Anthomyiidae|GMPJA3325-21|BIOUG65689-H11|BOLD:AAZ4294
Anthomyiidae|GMPJA494-21|BIOUG65660-F05|BOLD:AAZ4294
Anthomyiidae|GMPJA117-21|BIOUG65656-F08|BOLD:AAZ4294
Anthomyiidae|GMPJA3510-21|BIOUG65691-H06|BOLD:AAZ4294
Anthomyiidae|GMPJA3320-21|BIOUG65689-H06|BOLD:AAZ4294
Anthomyiidae|GMPJA3315-21|BIOUG65689-H01|BOLD:AAZ4294
Anthomyiidae|GMPJA3254-21|BIOUG65689-B12|BOLD:AAZ4294
Anthomyiidae|GMPJA1308-21|BIOUG65669-B12|BOLD:AAZ4294
Anthomyiidae|GMPJA162-21|BIOUG65657-B06|BOLD:AAZ4294
Anthomyiidae|GMPJA1345-21|BIOUG65669-F01|BOLD:AAZ4294
Anthomyiidae|GMPJA1072-21|BIOUG65666-G01|BOLD:AAZ4294
Anthomyiidae|GMPJA1065-21|BIOUG65666-F06|BOLD:AAZ4294
Anthomyiidae|GMPJA1036-21|BIOUG65666-D01|BOLD:AAZ4294
Anthomyiidae|GMPJA1409-21|BIOUG65670-C06|BOLD:AAZ4294
Anthomyiidae|GMPJA1570-21|BIOUG65672-A01|BOLD:AAZ4294
Anthomyiidae|GMPJA1581-21|BIOUG65672-A12|BOLD:AAZ4294
Anthomyiidae|GMPJA2294-21|BIOUG65679-E12|BOLD:AAZ4294
Anthomyiidae|GMPJA2092-21|BIOUG65677-D12|BOLD:AAZ4294
Anthomyiidae|GMPJA2098-21|BIOUG65677-E06|BOLD:AAZ4294
Anthomyiidae|GMPJA2401-21|BIOUG65680-F12|BOLD:AAZ4294
Anthomyiidae|GMPJA2044-21|BIOUG65676-H11|BOLD:AAZ4294
Anthomyiidae|GMPJA3246-21|BIOUG65689-B04|BOLD:AAZ4294
Anthomyiidae|GMPJA3158-21|BIOUG65688-B11|BOLD:AAZ4294
Adia cinerella|GMPJA2247-21|BIOUG65679-B01|BOLD:AAG2452
Adia cinerella|GMPJA1771-21|BIOUG65674-A12|BOLD:AAG2452
Adia cinerella|GMPJA1428-21|BIOUG65670-E01|BOLD:AAG2452
Adia cinerella|GMPJA1915-21|BIOUG65675-F01|BOLD:AAG2452
Adia cinerella|GMPJA1053-21|BIOUG65666-E06|BOLD:AAG2452
Adia cinerella|GMPJA1071-21|BIOUG65666-F12|BOLD:AAG2452
Adia cinerella|GMPJA1357-21|BIOUG65669-G01|BOLD:AAG2452
Adia cinerella|GMPJA958-21|BIOUG65665-E06|BOLD:AAG2452
Adia cinerella|GMPJA1403-21|BIOUG65670-B12|BOLD:AAG2452
Adia cinerella|GMPJA1029-21|BIOUG65666-C06|BOLD:AAG2452
Adia cinerella|GMPJA1416-21|BIOUG65670-D01|BOLD:AAG2452
Adia cinerella|GMPJA1713-21|BIOUG65673-E01|BOLD:AAG2452
Adia cinerella|GMPJA934-21|BIOUG65665-C06|BOLD:AAG2452
Chrysomya|GMPJA4888-21|BIOUG63984-D07|BOLD:AAA5667
Chrysomya|GMPJA004-21|BIOUG58255-A04|BOLD:AAA5667
Chrysomya|GMPJA4887-21|BIOUG63984-D06|BOLD:AAA5667
Chrysomya|GMPJA4889-21|BIOUG63984-D08|BOLD:AAA5667
Chrysomya|GMPJA4886-21|BIOUG63984-D05|BOLD:AAA5667
Delia|GMPJA1789-21|BIOUG65674-C06|BOLD:AAG2511
Delia|GMPJA940-21|BIOUG65665-C12|BOLD:AAG2511
Delia|GMPJA953-21|BIOUG65665-E01|BOLD:AAG2511
Delia|GMPJA1718-21|BIOUG65673-E06|BOLD:AAG2511
Delia|GMPJA727-21|BIOUG65663-B01|BOLD:AAG2511
Delia|GMPJA1023-21|BIOUG65666-B12|BOLD:AAG2511
Delia|GMPJA744-21|BIOUG65663-C06|BOLD:AAG2511
Delia|GMPJA917-21|BIOUG65665-B01|BOLD:AAG2511
Delia|GMPJA407-21|BIOUG65659-G01|BOLD:AAG2511
Delia|GMPJA525-21|BIOUG65661-A01|BOLD:AAG2511
Delia|GMPJA2128-21|BIOUG65677-G12|BOLD:AAG2511
Delia|GMPJA1094-21|BIOUG65666-H11|BOLD:AAG2511
Delia|GMPJA1167-21|BIOUG65667-G01|BOLD:AAG2511
Delia|GMPJA946-21|BIOUG65665-D06|BOLD:AAG2511
Delia|GMPJA1202-21|BIOUG65668-B01|BOLD:AAG2511
Delia|GMPJA1011-21|BIOUG65666-A12|BOLD:AAG2511
Delia|GMPJA988-21|BIOUG65665-G12|BOLD:AAG2511
Delia|GMPJA739-21|BIOUG65663-C01|BOLD:AAG2511
Delia|GMPJA1296-21|BIOUG65669-A12|BOLD:AAG2511
Delia|GMPJA1048-21|BIOUG65666-E01|BOLD:AAG2511
Delia|GMPJA1047-21|BIOUG65666-D12|BOLD:AAG2511
Delia|GMPJA1017-21|BIOUG65666-B06|BOLD:AAG2511
Delia|GMPJA1012-21|BIOUG65666-B01|BOLD:AAG2511
Delia|GMPJA1035-21|BIOUG65666-C12|BOLD:AAG2511
Delia|GMPJA1024-21|BIOUG65666-C01|BOLD:AAG2511
Delia|GMPJA1107-21|BIOUG65667-B01|BOLD:AAG2511
Delia|GMPJA1112-21|BIOUG65667-B06|BOLD:AAG2511
Delia|GMPJA1124-21|BIOUG65667-C06|BOLD:AAG2511
Delia|GMPJA1154-21|BIOUG65667-E12|BOLD:AAG2511
Delia|GMPJA1160-21|BIOUG65667-F06|BOLD:AAG2511
Delia|GMPJA1166-21|BIOUG65667-F12|BOLD:AAG2511
Delia|GMPJA1368-21|BIOUG65669-G12|BOLD:AAG2511
Delia|GMPJA1404-21|BIOUG65670-C01|BOLD:AAG2511
Delia|GMPJA1439-21|BIOUG65670-E12|BOLD:AAG2511
Delia|GMPJA1452-21|BIOUG65670-G01|BOLD:AAG2511
Delia|GMPJA952-21|BIOUG65665-D12|BOLD:AAG2511
Delia|GMPJA371-21|BIOUG65659-D01|BOLD:AAG2511
Delia|GMPJA941-21|BIOUG65665-D01|BOLD:AAG2511
Delia|GMPJA916-21|BIOUG65665-A12|BOLD:AAG2511
Delia|GMPJA2473-21|BIOUG65681-E01|BOLD:AAG2511
Delia|GMPJA1369-21|BIOUG65669-H01|BOLD:AAG2511
Delia|GMPJA1041-21|BIOUG65666-D06|BOLD:AAG2511
Delia|GMPJA2769-21|BIOUG65684-E12|BOLD:AAG2511
Delia|GMPJA1587-21|BIOUG65672-B06|BOLD:AAG2511
Delia|GMPJA2295-21|BIOUG65679-F01|BOLD:AAG2511
Delia|GMPJA1623-21|BIOUG65672-E06|BOLD:AAG2511
Delia|GMPJA1618-21|BIOUG65672-E01|BOLD:AAG2511
Delia|GMPJA1119-21|BIOUG65667-C01|BOLD:AAG2511
Delia|GMPJA1095-21|BIOUG65667-A01|BOLD:AAG2511
Delia|GMPJA1077-21|BIOUG65666-G06|BOLD:AAG2511
Delia|GMPJA513-21|BIOUG65660-G12|BOLD:AAG2511
Delia|GMPJA127-21|BIOUG65656-G06|BOLD:AAG2511
Delia|GMPJA2436-21|BIOUG65681-A12|BOLD:AAG2511
Asteia amoena|GMPJA3188-21|BIOUG65688-E05|BOLD:ACG0368
Asteia amoena|GMPJA4160-21|BIOUG65698-G03|BOLD:ACG0368
Asteia amoena|GMPJA3576-21|BIOUG65692-F01|BOLD:ACG0368
Asteia amoena|GMPJA3282-21|BIOUG65689-E04|BOLD:ACG0368

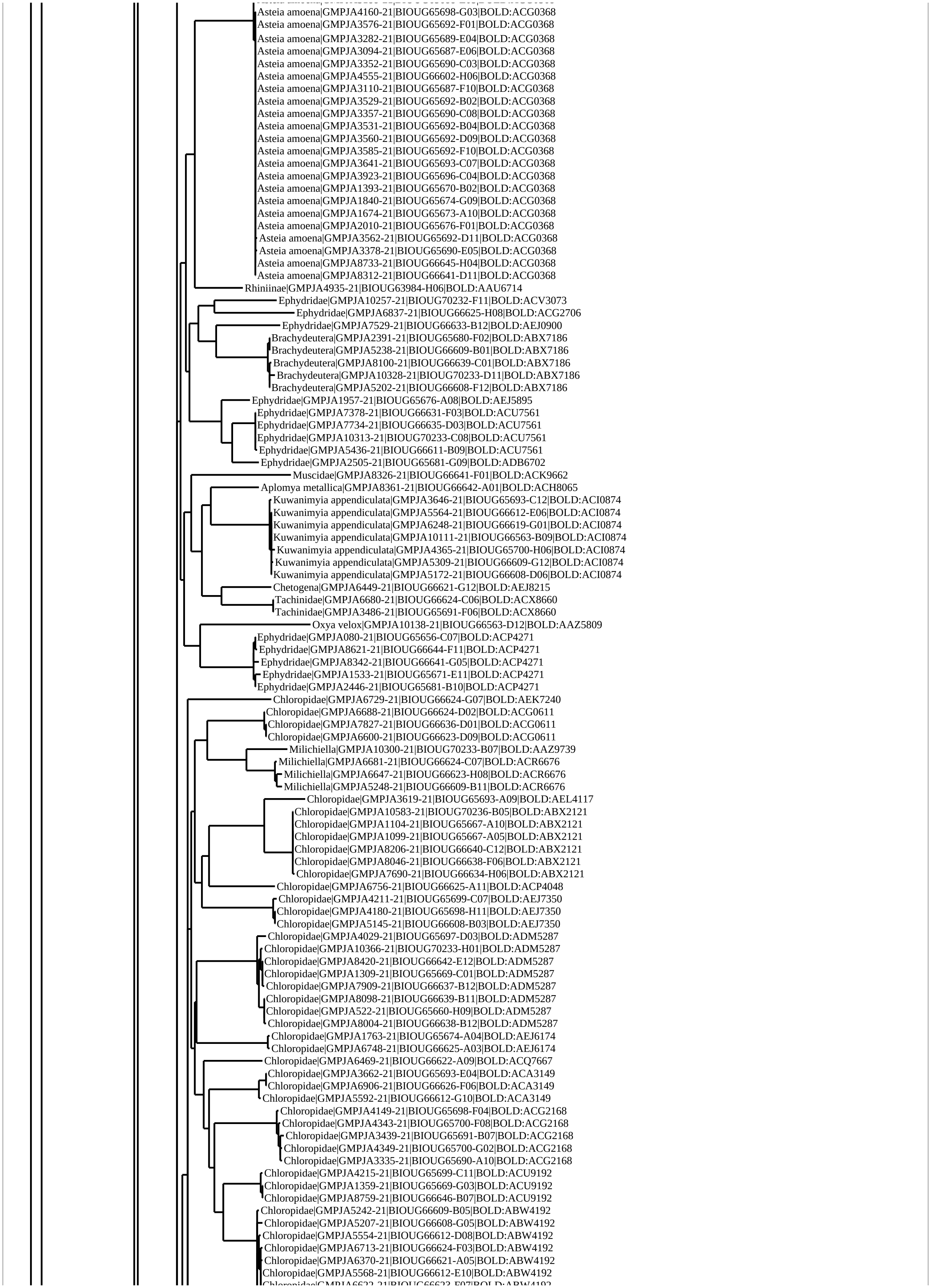
Asteia amoena|GMPJA4160-21|BIOUG65698-G03|BOLD:ACG0368
Asteia amoena|GMPJA3576-21|BIOUG65692-F01|BOLD:ACG0368
Asteia amoena|GMPJA3282-21|BIOUG65689-E04|BOLD:ACG0368
Asteia amoena|GMPJA3094-21|BIOUG65687-E06|BOLD:ACG0368
Asteia amoena|GMPJA3352-21|BIOUG65690-C03|BOLD:ACG0368
Asteia amoena|GMPJA4555-21|BIOUG66602-H06|BOLD:ACG0368
Asteia amoena|GMPJA3110-21|BIOUG65687-F10|BOLD:ACG0368
Asteia amoena|GMPJA3529-21|BIOUG65692-B02|BOLD:ACG0368
Asteia amoena|GMPJA3357-21|BIOUG65690-C08|BOLD:ACG0368
Asteia amoena|GMPJA3531-21|BIOUG65692-B04|BOLD:ACG0368
Asteia amoena|GMPJA3560-21|BIOUG65692-D09|BOLD:ACG0368
Asteia amoena|GMPJA3585-21|BIOUG65692-F10|BOLD:ACG0368
Asteia amoena|GMPJA3641-21|BIOUG65693-C07|BOLD:ACG0368
Asteia amoena|GMPJA3923-21|BIOUG65696-C04|BOLD:ACG0368
Asteia amoena|GMPJA1393-21|BIOUG65670-B02|BOLD:ACG0368
Asteia amoena|GMPJA1840-21|BIOUG65674-G09|BOLD:ACG0368
Asteia amoena|GMPJA1674-21|BIOUG65673-A10|BOLD:ACG0368
Asteia amoena|GMPJA2010-21|BIOUG65676-F01|BOLD:ACG0368
Asteia amoena|GMPJA3562-21|BIOUG65692-D11|BOLD:ACG0368
Asteia amoena|GMPJA3378-21|BIOUG65690-E05|BOLD:ACG0368
Asteia amoena|GMPJA8733-21|BIOUG66645-H04|BOLD:ACG0368
Asteia amoena|GMPJA8312-21|BIOUG66641-D11|BOLD:ACG0368
Rhiniinae|GMPJA4935-21|BIOUG63984-H06|BOLD:AAU6714
Ephydridae|GMPJA10257-21|BIOUG70232-F11|BOLD:ACV3073
Ephydridae|GMPJA6837-21|BIOUG66625-H08|BOLD:ACG2706
Ephydridae|GMPJA7529-21|BIOUG66633-B12|BOLD:AEJ0900
Brachydeutera|GMPJA2391-21|BIOUG65680-F02|BOLD:ABX7186
Brachydeutera|GMPJA5238-21|BIOUG66609-B01|BOLD:ABX7186
Brachydeutera|GMPJA8100-21|BIOUG66639-C01|BOLD:ABX7186
Brachydeutera|GMPJA10328-21|BIOUG70233-D11|BOLD:ABX7186
Brachydeutera|GMPJA5202-21|BIOUG66608-F12|BOLD:ABX7186
Ephydridae|GMPJA1957-21|BIOUG65676-A08|BOLD:AEJ5895
Ephydridae|GMPJA7378-21|BIOUG66631-F03|BOLD:ACU7561
Ephydridae|GMPJA7734-21|BIOUG66635-D03|BOLD:ACU7561
Ephydridae|GMPJA10313-21|BIOUG70233-C08|BOLD:ACU7561
Ephydridae|GMPJA5436-21|BIOUG66611-B09|BOLD:ACU7561
Ephydridae|GMPJA2505-21|BIOUG65681-G09|BOLD:ADB6702
Muscidae|GMPJA8326-21|BIOUG66641-F01|BOLD:ACK9662
Aplomya metallica|GMPJA8361-21|BIOUG66642-A01|BOLD:ACH8065
Kuwanimyia appendiculata|GMPJA3646-21|BIOUG65693-C12|BOLD:ACI0874
Kuwanimyia appendiculata|GMPJA5564-21|BIOUG66612-E06|BOLD:ACI0874
Kuwanimyia appendiculata|GMPJA6248-21|BIOUG66619-G01|BOLD:ACI0874
Kuwanimyia appendiculata|GMPJA10111-21|BIOUG66563-B09|BOLD:ACI0874
Kuwanimyia appendiculata|GMPJA4365-21|BIOUG65700-H06|BOLD:ACI0874
Kuwanimyia appendiculata|GMPJA5309-21|BIOUG66609-G12|BOLD:ACI0874
Kuwanimyia appendiculata|GMPJA5172-21|BIOUG66608-D06|BOLD:ACI0874
Chetogena|GMPJA6449-21|BIOUG66621-G12|BOLD:AEJ8215
Tachinidae|GMPJA6680-21|BIOUG66624-C06|BOLD:ACX8660
Tachinidae|GMPJA3486-21|BIOUG65691-F06|BOLD:ACX8660
Oxya velox|GMPJA10138-21|BIOUG66563-D12|BOLD:AAZ5809
Ephydridae|GMPJA080-21|BIOUG65656-C07|BOLD:ACP4271
Ephydridae|GMPJA8621-21|BIOUG66644-F11|BOLD:ACP4271
Ephydridae|GMPJA8342-21|BIOUG66641-G05|BOLD:ACP4271
Ephydridae|GMPJA1533-21|BIOUG65671-E11|BOLD:ACP4271
Ephydridae|GMPJA2446-21|BIOUG65681-B10|BOLD:ACP4271
Chloropidae|GMPJA6729-21|BIOUG66624-G07|BOLD:AEK7240
Chloropidae|GMPJA6688-21|BIOUG66624-D02|BOLD:ACG0611
Chloropidae|GMPJA7827-21|BIOUG66636-D01|BOLD:ACG0611
Chloropidae|GMPJA6600-21|BIOUG66623-D09|BOLD:ACG0611
Milichiella|GMPJA10300-21|BIOUG70233-B07|BOLD:AAZ9739
Milichiella|GMPJA6681-21|BIOUG66624-C07|BOLD:ACR6676
Milichiella|GMPJA6647-21|BIOUG66623-H08|BOLD:ACR6676
Milichiella|GMPJA5248-21|BIOUG66609-B11|BOLD:ACR6676
Chloropidae|GMPJA3619-21|BIOUG65693-A09|BOLD:AEL4117
Chloropidae|GMPJA10583-21|BIOUG70236-B05|BOLD:ABX2121
Chloropidae|GMPJA1104-21|BIOUG65667-A10|BOLD:ABX2121
Chloropidae|GMPJA1099-21|BIOUG65667-A05|BOLD:ABX2121
Chloropidae|GMPJA8206-21|BIOUG66640-C12|BOLD:ABX2121
Chloropidae|GMPJA8046-21|BIOUG66638-F06|BOLD:ABX2121
Chloropidae|GMPJA7690-21|BIOUG66634-H06|BOLD:ABX2121
Chloropidae|GMPJA6756-21|BIOUG66625-A11|BOLD:ACP4048
Chloropidae|GMPJA4211-21|BIOUG65699-C07|BOLD:AEJ7350
Chloropidae|GMPJA4180-21|BIOUG65698-H11|BOLD:AEJ7350
Chloropidae|GMPJA5145-21|BIOUG66608-B03|BOLD:AEJ7350
Chloropidae|GMPJA4029-21|BIOUG65697-D03|BOLD:ADM5287
Chloropidae|GMPJA10366-21|BIOUG70233-H01|BOLD:ADM5287
Chloropidae|GMPJA8420-21|BIOUG66642-E12|BOLD:ADM5287
Chloropidae|GMPJA1309-21|BIOUG65669-C01|BOLD:ADM5287
Chloropidae|GMPJA7909-21|BIOUG66637-B12|BOLD:ADM5287
Chloropidae|GMPJA8098-21|BIOUG66639-B11|BOLD:ADM5287
Chloropidae|GMPJA522-21|BIOUG65660-H09|BOLD:ADM5287
Chloropidae|GMPJA8004-21|BIOUG66638-B12|BOLD:ADM5287
Chloropidae|GMPJA1763-21|BIOUG65674-A04|BOLD:AEJ6174
Chloropidae|GMPJA6748-21|BIOUG66625-A03|BOLD:AEJ6174
Chloropidae|GMPJA6469-21|BIOUG66622-A09|BOLD:ACQ7667
Chloropidae|GMPJA3662-21|BIOUG65693-E04|BOLD:ACA3149
Chloropidae|GMPJA6906-21|BIOUG66626-F06|BOLD:ACA3149
Chloropidae|GMPJA5592-21|BIOUG66612-G10|BOLD:ACA3149
Chloropidae|GMPJA4149-21|BIOUG65698-F04|BOLD:ACG2168
Chloropidae|GMPJA4343-21|BIOUG65700-F08|BOLD:ACG2168
Chloropidae|GMPJA3439-21|BIOUG65691-B07|BOLD:ACG2168
Chloropidae|GMPJA4349-21|BIOUG65700-G02|BOLD:ACG2168
Chloropidae|GMPJA3335-21|BIOUG65690-A10|BOLD:ACG2168
Chloropidae|GMPJA4215-21|BIOUG65699-C11|BOLD:ACU9192
Chloropidae|GMPJA1359-21|BIOUG65669-G03|BOLD:ACU9192
Chloropidae|GMPJA8759-21|BIOUG66646-B07|BOLD:ACU9192
Chloropidae|GMPJA5242-21|BIOUG66609-B05|BOLD:ABW4192
Chloropidae|GMPJA5207-21|BIOUG66608-G05|BOLD:ABW4192
Chloropidae|GMPJA5554-21|BIOUG66612-D08|BOLD:ABW4192
Chloropidae|GMPJA6713-21|BIOUG66624-F03|BOLD:ABW4192
Chloropidae|GMPJA6370-21|BIOUG66621-A05|BOLD:ABW4192
Chloropidae|GMPJA5568-21|BIOUG66612-E10|BOLD:ABW4192

[illegible]
Chloropidae|GMPJA6370-21|BIOUG66621-A05|BOLD:ABW4192
Chloropidae|GMPJA5568-21|BIOUG66612-E10|BOLD:ABW4192
Chloropidae|GMPJA6622-21|BIOUG66623-F07|BOLD:ABW4192
Chloropidae|GMPJA4120-21|BIOUG65698-C11|BOLD:ABW4192
Chloropidae|GMPJA5529-21|BIOUG66612-B07|BOLD:ABW4192
Chloropidae|GMPJA5267-21|BIOUG66609-D06|BOLD:ABW4192
Chloropidae|GMPJA5052-21|BIOUG66607-B05|BOLD:ABW4192
Chloropidae|GMPJA2018-21|BIOUG65676-F09|BOLD:ABW4192
Chloropidae|GMPJA6652-21|BIOUG66624-A02|BOLD:ABW4192
Chloropidae|GMPJA5330-21|BIOUG66610-A10|BOLD:ABW4192
Chloropidae|GMPJA7860-21|BIOUG66636-F10|BOLD:ABW4192
Chloropidae|GMPJA6611-21|BIOUG66623-E08|BOLD:ABW4192
Chloropidae|GMPJA4190-21|BIOUG65699-A10|BOLD:ABW4192
Chloropidae|GMPJA4054-21|BIOUG65697-F04|BOLD:ABW4192
Chloropidae|GMPJA1977-21|BIOUG65676-C04|BOLD:ABW4192
Chloropidae|GMPJA5583-21|BIOUG66612-G01|BOLD:ABW4192
Chloropidae|GMPJA5241-21|BIOUG66609-B04|BOLD:ABW4192
Chloropidae|GMPJA5435-21|BIOUG66611-B08|BOLD:ABW4192
Chloropidae|GMPJA5073-21|BIOUG66607-D02|BOLD:ABW4192
Chloropidae|GMPJA4119-21|BIOUG65698-C10|BOLD:ABW4192
Chloropidae|GMPJA5300-21|BIOUG66609-G03|BOLD:ABW4192
Chloropidae|GMPJA5390-21|BIOUG66610-F10|BOLD:ABW4192
Chloropidae|GMPJA3075-21|BIOUG65687-C11|BOLD:ABW4192
Chloropidae|GMPJA5313-21|BIOUG66609-H04|BOLD:ABW4192
Chloropidae|GMPJA4503-21|BIOUG66602-D02|BOLD:ABW4192
Chloropidae|GMPJA10654-21|BIOUG70236-H04|BOLD:ABW4192
Chloropidae|GMPJA3400-21|BIOUG65690-G03|BOLD:ABW4192
Chloropidae|GMPJA6672-21|BIOUG66624-B10|BOLD:ABW4192
Chloropidae|GMPJA6575-21|BIOUG66623-B08|BOLD:ABW4192
Chloropidae|GMPJA6411-21|BIOUG66621-D10|BOLD:ABW4192
Chloropidae|GMPJA4245-21|BIOUG65699-F05|BOLD:ABW4192
Chloropidae|GMPJA3327-21|BIOUG65690-A02|BOLD:ABW4192
Chloropidae|GMPJA5477-21|BIOUG66611-F02|BOLD:ABW4192
Chloropidae|GMPJA6283-21|BIOUG66620-B01|BOLD:ABW4192
Chloropidae|GMPJA5423-21|BIOUG66611-A08|BOLD:ABW4192
Chloropidae|GMPJA5209-21|BIOUG66608-G07|BOLD:ABW4192
Chloropidae|GMPJA6410-21|BIOUG66621-D09|BOLD:ABW4192
Chloropidae|GMPJA5561-21|BIOUG66612-E03|BOLD:ABW4192
Chloropidae|GMPJA5247-21|BIOUG66609-B10|BOLD:ABW4192
Chloropidae|GMPJA5517-21|BIOUG66612-A07|BOLD:ABW4192
Chloropidae|GMPJA6904-21|BIOUG66626-F04|BOLD:ABW4192
Chloropidae|GMPJA8502-21|BIOUG66643-D11|BOLD:ABW4192
Chloropidae|GMPJA3334-21|BIOUG65690-A09|BOLD:ABW4192
Chloropidae|GMPJA3380-21|BIOUG65690-E07|BOLD:ABW4192
Chloropidae|GMPJA10340-21|BIOUG70233-E11|BOLD:ABW4192
Chloropidae|GMPJA4461-21|BIOUG66601-H07|BOLD:ABW4192
Chloropidae|GMPJA10556-21|BIOUG70235-H01|BOLD:ABW4192
Chloropidae|GMPJA4003-21|BIOUG65697-B01|BOLD:ABW4192
Chloropidae|GMPJA2297-21|BIOUG65679-F03|BOLD:ABW4192
Chloropidae|GMPJA1807-21|BIOUG65674-D12|BOLD:ABW4192
Chloropidae|GMPJA3993-21|BIOUG65697-A03|BOLD:ABW4192
Chloropidae|GMPJA10639-21|BIOUG70236-G01|BOLD:ABW4192
Chloropidae|GMPJA6192-21|BIOUG66619-B05|BOLD:ABW4192
Chloropidae|GMPJA5507-21|BIOUG66611-H08|BOLD:ABW4192
Chloropidae|GMPJA5270-21|BIOUG66609-D09|BOLD:ABW4192
Chloropidae|GMPJA5226-21|BIOUG66609-A01|BOLD:ABW4192
Chloropidae|GMPJA5450-21|BIOUG66611-C11|BOLD:ABW4192
Chloropidae|GMPJA5418-21|BIOUG66611-A03|BOLD:ABW4192
Chloropidae|GMPJA5040-21|BIOUG66607-A05|BOLD:ABW4192
Chloropidae|GMPJA4090-21|BIOUG65698-A05|BOLD:ADZ5395
Chloropidae|GMPJA4273-21|BIOUG65699-H09|BOLD:ADZ5395
Chloropidae|GMPJA7245-21|BIOUG66630-C01|BOLD:ADZ5395
Chloropidae|GMPJA1498-21|BIOUG65671-B12|BOLD:AEJ0753
Chloropidae|GMPJA4098-21|BIOUG65698-B01|BOLD:AEJ0753
Chloropidae|GMPJA4410-21|BIOUG66601-D04|BOLD:AEJ0753
Chloropidae|GMPJA6749-21|BIOUG66625-A04|BOLD:AEJ0753
Chloropidae|GMPJA6408-21|BIOUG66621-D07|BOLD:AEJ0753
Oscinellinae|GMPJA8438-21|BIOUG66642-G06|BOLD:AAG1512
Oscinellinae|GMPJA7528-21|BIOUG66633-B11|BOLD:AAG1512
Oscinellinae|GMPJA7548-21|BIOUG66633-D07|BOLD:AAG1512
Oscinellinae|GMPJA8350-21|BIOUG66641-H01|BOLD:AAG1512
Oscinellinae|GMPJA4464-21|BIOUG66601-H10|BOLD:AAG1512
Oscinellinae|GMPJA8442-21|BIOUG66642-G10|BOLD:AAG1512
Oscinellinae|GMPJA8542-21|BIOUG66643-H03|BOLD:AAG1512
Oscinellinae|GMPJA1881-21|BIOUG65675-C03|BOLD:AAG1512
Oscinellinae|GMPJA8736-21|BIOUG66645-H07|BOLD:AAG1512
Oscinellinae|GMPJA6836-21|BIOUG66625-H07|BOLD:AAG1512
Oscinellinae|GMPJA6543-21|BIOUG66622-G11|BOLD:AAG1512
Anatrichus|GMPJA1724-21|BIOUG65673-E12|BOLD:AEJ2403
Anatrichus|GMPJA8485-21|BIOUG66643-C06|BOLD:ACV2691
Anatrichus|GMPJA10221-21|BIOUG70232-C11|BOLD:ACV2691
Anatrichus|GMPJA063-21|BIOUG65656-B02|BOLD:ACV2691
Anatrichus|GMPJA7980-21|BIOUG66637-H11|BOLD:ACV2691
Anatrichus|GMPJA10352-21|BIOUG70233-F11|BOLD:ACV2691
Anatrichus|GMPJA10290-21|BIOUG70233-A09|BOLD:ACV2691
Anatrichus|GMPJA10225-21|BIOUG70232-D03|BOLD:ACV2691
Anatrichus|GMPJA10205-21|BIOUG70232-B07|BOLD:ACV2691
Anatrichus|GMPJA8693-21|BIOUG66645-D12|BOLD:ACV2691
Anatrichus|GMPJA7244-21|BIOUG66630-B12|BOLD:ACV2691
Anatrichus|GMPJA10373-21|BIOUG70233-H08|BOLD:ACV2691
Anatrichus|GMPJA7281-21|BIOUG66630-F01|BOLD:ACV2691
Anatrichus|GMPJA10314-21|BIOUG70233-C09|BOLD:ACV2691
Anatrichus|GMPJA177-21|BIOUG65657-C09|BOLD:ACV2691
Anatrichus|GMPJA5391-21|BIOUG66610-F11|BOLD:ACV2691
Chloropidae|GMPJA405-21|BIOUG65659-F11|BOLD:ACU7682
Chloropidae|GMPJA3078-21|BIOUG65687-D02|BOLD:ACV1221
Melanochaeta|GMPJA1825-21|BIOUG65674-F06|BOLD:ACR3351
Melanochaeta|GMPJA10308-21|BIOUG70233-C03|BOLD:ACR3351
Melanochaeta|GMPJA10296-21|BIOUG70233-B03|BOLD:ACR3351
Melanochaeta|GMPJA8182-21|BIOUG66640-A12|BOLD:ACR3351
Chloropidae|GMPJA10304-21|BIOUG70233-B11|BOLD:ABX5968
Chloropidae|GMPJA1681-21|BIOUG65673-B05|BOLD:ABX5968
[illegible]

Melanochaeta|GMPJA8182-21|BIOUG66640-A12|BOLD:ACR3351
Chloropidae|GMPJA10304-21|BIOUG70233-B11|BOLD:ABX5968
Chloropidae|GMPJA1681-21|BIOUG65673-B05|BOLD:ABX5968
Chloropidae|GMPJA8528-21|BIOUG66643-G01|BOLD:ABX5968
Chloropidae|GMPJA377-21|BIOUG65659-D07|BOLD:ABX5968
Chloropidae|GMPJA10345-21|BIOUG70233-F04|BOLD:ABX5968
Chloropidae|GMPJA1795-21|BIOUG65674-C12|BOLD:ABX5968
Chloropidae|GMPJA372-21|BIOUG65659-D02|BOLD:ABX5968
Chloropidae|GMPJA2388-21|BIOUG65680-E11|BOLD:ABX5968
Chloropidae|GMPJA10281-21|BIOUG70232-H11|BOLD:ABX5968
Chloropidae|GMPJA7979-21|BIOUG66637-H10|BOLD:ABX5968
Chloropidae|GMPJA7332-21|BIOUG66631-B05|BOLD:ABX5968
Cadrema|GMPJA8083-21|BIOUG66639-A08|BOLD:AAF5121
Cadrema|GMPJA6696-21|BIOUG66624-D10|BOLD:AAF5121
Cadrema|GMPJA6615-21|BIOUG66623-E12|BOLD:AAF5121
Cadrema|GMPJA7308-21|BIOUG66630-H04|BOLD:AAF5121
Cadrema|GMPJA6764-21|BIOUG66625-B07|BOLD:AAF5121
Cadrema|GMPJA6512-21|BIOUG66622-E04|BOLD:AAF5121
Cadrema|GMPJA7333-21|BIOUG66631-B06|BOLD:AAF5121
Cadrema|GMPJA7669-21|BIOUG66634-F09|BOLD:AAF5121
Cadrema|GMPJA8348-21|BIOUG66641-G11|BOLD:AAF5121
Cadrema|GMPJA8290-21|BIOUG66641-C01|BOLD:AAF5121
Cadrema|GMPJA8771-21|BIOUG66646-C07|BOLD:AAF5121
Cadrema|GMPJA10193-21|BIOUG70232-A07|BOLD:AAF5121
Cadrema|GMPJA10547-21|BIOUG70235-G04|BOLD:AAF5121
Cadrema|GMPJA10597-21|BIOUG70236-C07|BOLD:AAF5121
Cadrema|GMPJA1532-21|BIOUG65671-E10|BOLD:AAF5121
Cadrema|GMPJA7706-21|BIOUG66635-A11|BOLD:AAF5121
Cadrema|GMPJA6191-21|BIOUG66619-B04|BOLD:AAF5121
Chloropidae|GMPJA1701-21|BIOUG65673-D01|BOLD:ACA3146
Chloropidae|GMPJA5164-21|BIOUG66608-C10|BOLD:ACA3146
Chloropidae|GMPJA6548-21|BIOUG66622-H04|BOLD:ACG2169
Chloropidae|GMPJA5128-21|BIOUG66607-H09|BOLD:ACP2616
Chloropidae|GMPJA5358-21|BIOUG66610-D02|BOLD:ACF9016
Siphunculina striolata|GMPJA509-21|BIOUG65660-G08|BOLD:ADZ3871
Chloropidae|GMPJA8690-21|BIOUG66645-D09|BOLD:AEL6471
Chloropidae|GMPJA5288-21|BIOUG66609-F03|BOLD:ACG0241
Chloropidae|GMPJA7863-21|BIOUG66636-G01|BOLD:ACG0241
Chloropidae|GMPJA5244-21|BIOUG66609-B07|BOLD:ACG0241
Chloropidae|GMPJA8649-21|BIOUG66645-A04|BOLD:ACG0241
Chloropidae|GMPJA7283-21|BIOUG66630-F03|BOLD:ACG0241
Chloropidae|GMPJA5382-21|BIOUG66610-F02|BOLD:ACG0241
Chloropidae|GMPJA7618-21|BIOUG66634-B06|BOLD:ACG0241
Chloropidae|GMPJA8200-21|BIOUG66640-C06|BOLD:ACG0241
Chloropidae|GMPJA7873-21|BIOUG66636-G11|BOLD:ACG0241
Chloropidae|GMPJA7917-21|BIOUG66637-C08|BOLD:ACG0241
Chloropidae|GMPJA4197-21|BIOUG65699-B05|BOLD:ACG0241
Chloropidae|GMPJA7754-21|BIOUG66635-E11|BOLD:ACG0241
Chloropidae|GMPJA7984-21|BIOUG66638-A04|BOLD:ACG0241
Chloropidae|GMPJA5067-21|BIOUG66607-C08|BOLD:ACG0241
Chloropidae|GMPJA10669-21|BIOUG70237-A08|BOLD:ACG0241
Chloropidae|GMPJA4491-21|BIOUG66602-C02|BOLD:ACG0241
Chloropidae|GMPJA8671-21|BIOUG66645-C02|BOLD:ACG0241
Chloropidae|GMPJA8465-21|BIOUG66643-A10|BOLD:ACG0241
Chloropidae|GMPJA8464-21|BIOUG66643-A09|BOLD:ACG0241
Chloropidae|GMPJA8209-21|BIOUG66640-D03|BOLD:ACG0241
Chloropidae|GMPJA7249-21|BIOUG66630-C05|BOLD:ACG0241
Chloropidae|GMPJA6824-21|BIOUG66625-G07|BOLD:ACG0241
Chloropidae|GMPJA6593-21|BIOUG66623-D02|BOLD:ACG0241
Chloropidae|GMPJA5057-21|BIOUG66607-B10|BOLD:ACG0241
Chloropidae|GMPJA3060-21|BIOUG65687-B08|BOLD:ACG0241
Chloropidae|GMPJA4581-21|BIOUG66603-B09|BOLD:ACG0241
Chloropidae|GMPJA8466-21|BIOUG66643-A11|BOLD:ACG0241
Chloropidae|GMPJA6288-21|BIOUG66620-B06|BOLD:ACG0241
Chloropidae|GMPJA5144-21|BIOUG66608-B02|BOLD:ACG0241
Chloropidae|GMPJA4223-21|BIOUG65699-D07|BOLD:ACG0241
Chloropidae|GMPJA8526-21|BIOUG66643-F11|BOLD:ACG0241
Chloropidae|GMPJA8169-21|BIOUG66639-H10|BOLD:ACG0241
Chloropidae|GMPJA10574-21|BIOUG70236-A08|BOLD:ACG0241
Chloropidae|GMPJA5557-21|BIOUG66612-D11|BOLD:ACG0241
Chloropidae|GMPJA3271-21|BIOUG65689-D05|BOLD:ACG0241
Chloropidae|GMPJA4129-21|BIOUG65698-D08|BOLD:ACG0241
Chloropidae|GMPJA8506-21|BIOUG66643-E03|BOLD:ACG0241
Chloropidae|GMPJA8172-21|BIOUG66640-A02|BOLD:ACG0241
Chloropidae|GMPJA5492-21|BIOUG66611-G05|BOLD:ACG0241
Chloropidae|GMPJA8757-21|BIOUG66646-B05|BOLD:ACG0241
Chloropidae|GMPJA5191-21|BIOUG66608-F01|BOLD:ACG0241
Chloropidae|GMPJA7858-21|BIOUG66636-F08|BOLD:ACG0241
Chloropidae|GMPJA6730-21|BIOUG66624-G08|BOLD:ACG0241
Chloropidae|GMPJA5043-21|BIOUG66607-A08|BOLD:ACG0241
Chloropidae|GMPJA4105-21|BIOUG65698-B08|BOLD:ACP4699
Chloropidae|GMPJA8699-21|BIOUG66645-E06|BOLD:ACP4699
Chloropidae|GMPJA10322-21|BIOUG70233-D05|BOLD:ACP4699
Chloropidae|GMPJA158-21|BIOUG65657-B02|BOLD:ACP4699
Chloropidae|GMPJA5199-21|BIOUG66608-F09|BOLD:ACP4699
Chloropidae|GMPJA7545-21|BIOUG66633-D04|BOLD:ACP4699
Chloropidae|GMPJA7240-21|BIOUG66630-B08|BOLD:ACP4699
Chloropidae|GMPJA4271-21|BIOUG65699-H07|BOLD:ACP4699
Chloropidae|GMPJA3111-21|BIOUG65687-F11|BOLD:ACP4699
Chloropidae|GMPJA474-21|BIOUG65660-D09|BOLD:ACP4699
Chloropidae|GMPJA7758-21|BIOUG66635-F03|BOLD:ACP4699
Chloropidae|GMPJA052-21|BIOUG65656-A03|BOLD:ACP4699
Chloropidae|GMPJA8514-21|BIOUG66643-E11|BOLD:ACP4699
Chloropidae|GMPJA8301-21|BIOUG66641-C12|BOLD:ACP4699
Chloropidae|GMPJA7747-21|BIOUG66635-E04|BOLD:ACP4699
Chloropidae|GMPJA8415-21|BIOUG66642-E07|BOLD:ACP4699
Chloropidae|GMPJA436-21|BIOUG65660-A07|BOLD:ACP4699
Chloropidae|GMPJA135-21|BIOUG65656-H02|BOLD:ACP4699
Chloropidae|GMPJA8227-21|BIOUG66640-E09|BOLD:ACP4699
Chloropidae|GMPJA3671-21|BIOUG65693-F01|BOLD:ACP4699
Chloropidae|GMPJA8709-21|BIOUG66645-F04|BOLD:ACP4699
Chloropidae|GMPJA8338-21|BIOUG66641-G01|BOLD:ACP4699
Chloropidae|GMPJA7388-21|BIOUG66631-G01|BOLD:ACP4699
[illegible]

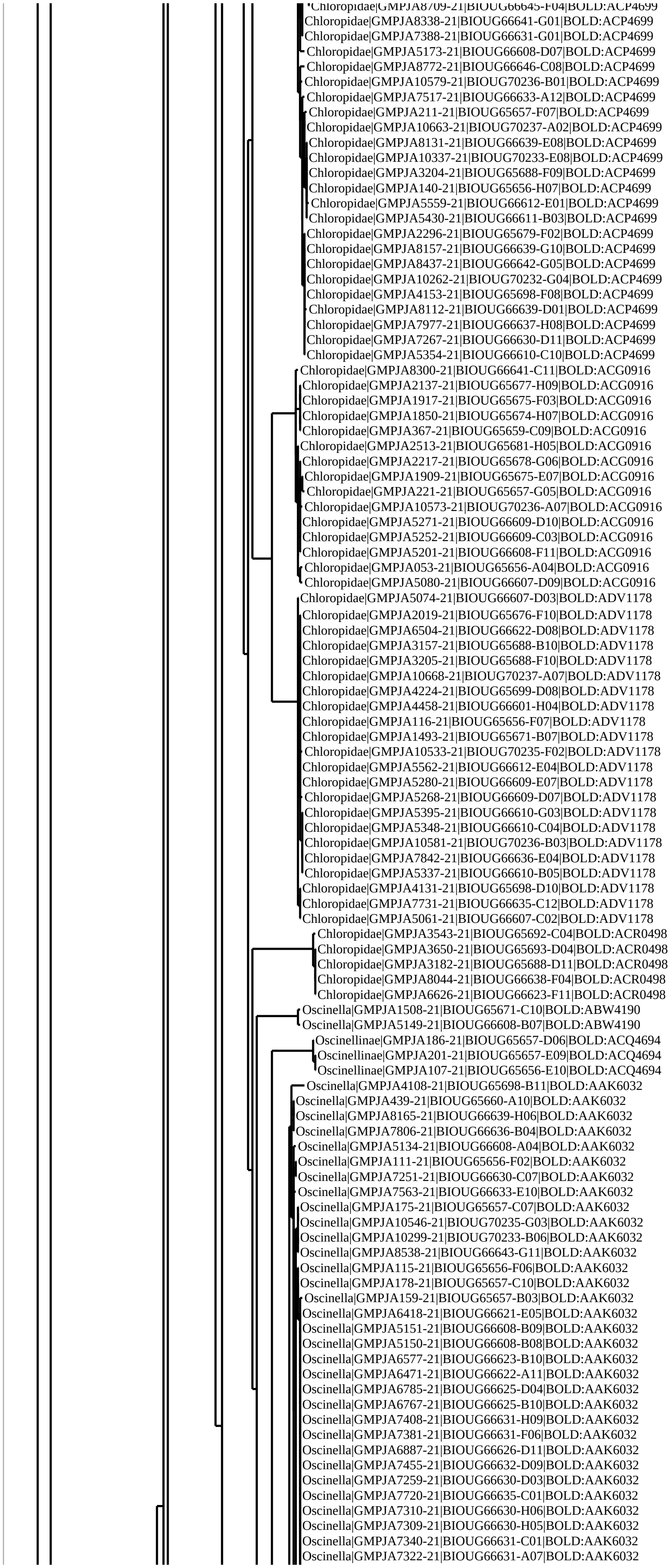
Chloropidae|GMPJA8709-21|BIOUG66645-F04|BOLD:ACP4699
Chloropidae|GMPJA8338-21|BIOUG66641-G01|BOLD:ACP4699
Chloropidae|GMPJA7388-21|BIOUG66631-G01|BOLD:ACP4699
Chloropidae|GMPJA5173-21|BIOUG66608-D07|BOLD:ACP4699
Chloropidae|GMPJA8772-21|BIOUG66646-C08|BOLD:ACP4699
Chloropidae|GMPJA10579-21|BIOUG70236-B01|BOLD:ACP4699
Chloropidae|GMPJA7517-21|BIOUG66633-A12|BOLD:ACP4699
Chloropidae|GMPJA211-21|BIOUG65657-F07|BOLD:ACP4699
Chloropidae|GMPJA10663-21|BIOUG70237-A02|BOLD:ACP4699
Chloropidae|GMPJA8131-21|BIOUG66639-E08|BOLD:ACP4699
Chloropidae|GMPJA10337-21|BIOUG70233-E08|BOLD:ACP4699
Chloropidae|GMPJA3204-21|BIOUG65688-F09|BOLD:ACP4699
Chloropidae|GMPJA140-21|BIOUG65656-H07|BOLD:ACP4699
Chloropidae|GMPJA5559-21|BIOUG66612-E01|BOLD:ACP4699
Chloropidae|GMPJA5430-21|BIOUG66611-B03|BOLD:ACP4699
Chloropidae|GMPJA2296-21|BIOUG65679-F02|BOLD:ACP4699
Chloropidae|GMPJA8157-21|BIOUG66639-G10|BOLD:ACP4699
Chloropidae|GMPJA8437-21|BIOUG66642-G05|BOLD:ACP4699
Chloropidae|GMPJA10262-21|BIOUG70232-G04|BOLD:ACP4699
Chloropidae|GMPJA4153-21|BIOUG65698-F08|BOLD:ACP4699
Chloropidae|GMPJA8112-21|BIOUG66639-D01|BOLD:ACP4699
Chloropidae|GMPJA7977-21|BIOUG66637-H08|BOLD:ACP4699
Chloropidae|GMPJA7267-21|BIOUG66630-D11|BOLD:ACP4699
Chloropidae|GMPJA5354-21|BIOUG66610-C10|BOLD:ACP4699
Chloropidae|GMPJA8300-21|BIOUG66641-C11|BOLD:ACG0916
Chloropidae|GMPJA2137-21|BIOUG65677-H09|BOLD:ACG0916
Chloropidae|GMPJA1917-21|BIOUG65675-F03|BOLD:ACG0916
Chloropidae|GMPJA1850-21|BIOUG65674-H07|BOLD:ACG0916
Chloropidae|GMPJA367-21|BIOUG65659-C09|BOLD:ACG0916
Chloropidae|GMPJA2513-21|BIOUG65681-H05|BOLD:ACG0916
Chloropidae|GMPJA2217-21|BIOUG65678-G06|BOLD:ACG0916
Chloropidae|GMPJA1909-21|BIOUG65675-E07|BOLD:ACG0916
Chloropidae|GMPJA221-21|BIOUG65657-G05|BOLD:ACG0916
Chloropidae|GMPJA10573-21|BIOUG70236-A07|BOLD:ACG0916
Chloropidae|GMPJA5271-21|BIOUG66609-D10|BOLD:ACG0916
Chloropidae|GMPJA5252-21|BIOUG66609-C03|BOLD:ACG0916
Chloropidae|GMPJA5201-21|BIOUG66608-F11|BOLD:ACG0916
Chloropidae|GMPJA053-21|BIOUG65656-A04|BOLD:ACG0916
Chloropidae|GMPJA5080-21|BIOUG66607-D09|BOLD:ACG0916
Chloropidae|GMPJA5074-21|BIOUG66607-D03|BOLD:ADV1178
Chloropidae|GMPJA2019-21|BIOUG65676-F10|BOLD:ADV1178
Chloropidae|GMPJA6504-21|BIOUG66622-D08|BOLD:ADV1178
Chloropidae|GMPJA3157-21|BIOUG65688-B10|BOLD:ADV1178
Chloropidae|GMPJA3205-21|BIOUG65688-F10|BOLD:ADV1178
Chloropidae|GMPJA10668-21|BIOUG70237-A07|BOLD:ADV1178
Chloropidae|GMPJA4224-21|BIOUG65699-D08|BOLD:ADV1178
Chloropidae|GMPJA4458-21|BIOUG66601-H04|BOLD:ADV1178
Chloropidae|GMPJA116-21|BIOUG65656-F07|BOLD:ADV1178
Chloropidae|GMPJA1493-21|BIOUG65671-B07|BOLD:ADV1178
Chloropidae|GMPJA10533-21|BIOUG70235-F02|BOLD:ADV1178
Chloropidae|GMPJA5562-21|BIOUG66612-E04|BOLD:ADV1178
Chloropidae|GMPJA5280-21|BIOUG66609-E07|BOLD:ADV1178
Chloropidae|GMPJA5268-21|BIOUG66609-D07|BOLD:ADV1178
Chloropidae|GMPJA5395-21|BIOUG66610-G03|BOLD:ADV1178
Chloropidae|GMPJA5348-21|BIOUG66610-C04|BOLD:ADV1178
Chloropidae|GMPJA10581-21|BIOUG70236-B03|BOLD:ADV1178
Chloropidae|GMPJA7842-21|BIOUG66636-E04|BOLD:ADV1178
Chloropidae|GMPJA5337-21|BIOUG66610-B05|BOLD:ADV1178
Chloropidae|GMPJA4131-21|BIOUG65698-D10|BOLD:ADV1178
Chloropidae|GMPJA7731-21|BIOUG66635-C12|BOLD:ADV1178
Chloropidae|GMPJA5061-21|BIOUG66607-C02|BOLD:ADV1178
Chloropidae|GMPJA3543-21|BIOUG65692-C04|BOLD:ACR0498
Chloropidae|GMPJA3650-21|BIOUG65693-D04|BOLD:ACR0498
Chloropidae|GMPJA3182-21|BIOUG65688-D11|BOLD:ACR0498
Chloropidae|GMPJA8044-21|BIOUG66638-F04|BOLD:ACR0498
Chloropidae|GMPJA6626-21|BIOUG66623-F11|BOLD:ACR0498
Oscinella|GMPJA1508-21|BIOUG65671-C10|BOLD:ABW4190
Oscinella|GMPJA5149-21|BIOUG66608-B07|BOLD:ABW4190
Oscinellinae|GMPJA186-21|BIOUG65657-D06|BOLD:ACQ4694
Oscinellinae|GMPJA201-21|BIOUG65657-E09|BOLD:ACQ4694
Oscinellinae|GMPJA107-21|BIOUG65656-E10|BOLD:ACQ4694
Oscinella|GMPJA4108-21|BIOUG65698-B11|BOLD:AAK6032
Oscinella|GMPJA439-21|BIOUG65660-A10|BOLD:AAK6032
Oscinella|GMPJA8165-21|BIOUG66639-H06|BOLD:AAK6032
Oscinella|GMPJA7806-21|BIOUG66636-B04|BOLD:AAK6032
Oscinella|GMPJA5134-21|BIOUG66608-A04|BOLD:AAK6032
Oscinella|GMPJA111-21|BIOUG65656-F02|BOLD:AAK6032
Oscinella|GMPJA7251-21|BIOUG66630-C07|BOLD:AAK6032
Oscinella|GMPJA7563-21|BIOUG66633-E10|BOLD:AAK6032
Oscinella|GMPJA175-21|BIOUG65657-C07|BOLD:AAK6032
Oscinella|GMPJA10546-21|BIOUG70235-G03|BOLD:AAK6032
Oscinella|GMPJA10299-21|BIOUG70233-B06|BOLD:AAK6032
Oscinella|GMPJA8538-21|BIOUG66643-G11|BOLD:AAK6032
Oscinella|GMPJA115-21|BIOUG65656-F06|BOLD:AAK6032
Oscinella|GMPJA178-21|BIOUG65657-C10|BOLD:AAK6032
Oscinella|GMPJA159-21|BIOUG65657-B03|BOLD:AAK6032
Oscinella|GMPJA6418-21|BIOUG66621-E05|BOLD:AAK6032
Oscinella|GMPJA5151-21|BIOUG66608-B09|BOLD:AAK6032
Oscinella|GMPJA5150-21|BIOUG66608-B08|BOLD:AAK6032
Oscinella|GMPJA6577-21|BIOUG66623-B10|BOLD:AAK6032
Oscinella|GMPJA6471-21|BIOUG66622-A11|BOLD:AAK6032
Oscinella|GMPJA6785-21|BIOUG66625-D04|BOLD:AAK6032
Oscinella|GMPJA6767-21|BIOUG66625-B10|BOLD:AAK6032
Oscinella|GMPJA7408-21|BIOUG66631-H09|BOLD:AAK6032
Oscinella|GMPJA7381-21|BIOUG66631-F06|BOLD:AAK6032
Oscinella|GMPJA6887-21|BIOUG66626-D11|BOLD:AAK6032
Oscinella|GMPJA7455-21|BIOUG66632-D09|BOLD:AAK6032
Oscinella|GMPJA7259-21|BIOUG66630-D03|BOLD:AAK6032
Oscinella|GMPJA7720-21|BIOUG66635-C01|BOLD:AAK6032
Oscinella|GMPJA7310-21|BIOUG66630-H06|BOLD:AAK6032
Oscinella|GMPJA7309-21|BIOUG66630-H05|BOLD:AAK6032
Oscinella|GMPJA7340-21|BIOUG66631-C01|BOLD:AAK6032
Oscinella|GMPJA7322-21|BIOUG66631-A07|BOLD:AAK6032

Oscinella|GMPJA7309-21|BIOUG66630-H05|BOLD:AAK6032
Oscinella|GMPJA7340-21|BIOUG66631-C01|BOLD:AAK6032
Oscinella|GMPJA7322-21|BIOUG66631-A07|BOLD:AAK6032
Oscinella|GMPJA7992-21|BIOUG66638-A12|BOLD:AAK6032
Oscinella|GMPJA7475-21|BIOUG66632-F05|BOLD:AAK6032
Oscinella|GMPJA8043-21|BIOUG66638-F03|BOLD:AAK6032
Oscinella|GMPJA8005-21|BIOUG66638-C01|BOLD:AAK6032
Oscinella|GMPJA8234-21|BIOUG66640-F04|BOLD:AAK6032
Oscinella|GMPJA8110-21|BIOUG66639-C11|BOLD:AAK6032
Oscinella|GMPJA7853-21|BIOUG66636-F03|BOLD:AAK6032
Oscinella|GMPJA7840-21|BIOUG66636-E02|BOLD:AAK6032
Oscinella|GMPJA7778-21|BIOUG66635-G11|BOLD:AAK6032
Oscinella|GMPJA7630-21|BIOUG66634-C06|BOLD:AAK6032
Oscinella|GMPJA7584-21|BIOUG66633-G07|BOLD:AAK6032
Oscinella|GMPJA8063-21|BIOUG66638-G11|BOLD:AAK6032
Oscinella|GMPJA7891-21|BIOUG66637-A06|BOLD:AAK6032
Oscinella|GMPJA7867-21|BIOUG66636-G05|BOLD:AAK6032
Oscinella|GMPJA8275-21|BIOUG66641-A10|BOLD:AAK6032
Oscinella|GMPJA8268-21|BIOUG66641-A03|BOLD:AAK6032
Oscinella|GMPJA8401-21|BIOUG66642-D05|BOLD:AAK6032
Oscinella|GMPJA8396-21|BIOUG66642-C12|BOLD:AAK6032
Oscinella|GMPJA8384-21|BIOUG66642-B12|BOLD:AAK6032
Oscinella|GMPJA8367-21|BIOUG66642-A07|BOLD:AAK6032
Oscinella|GMPJA8444-21|BIOUG66642-G12|BOLD:AAK6032
Oscinella|GMPJA8408-21|BIOUG66642-D12|BOLD:AAK6032
Oscinella|GMPJA8705-21|BIOUG66645-E12|BOLD:AAK6032
Oscinella|GMPJA8684-21|BIOUG66645-D03|BOLD:AAK6032
Oscinella|GMPJA8626-21|BIOUG66644-G04|BOLD:AAK6032
Oscinella|GMPJA8461-21|BIOUG66643-A06|BOLD:AAK6032
Oscinella|GMPJA8788-21|BIOUG66646-D12|BOLD:AAK6032
Oscinella|GMPJA8725-21|BIOUG66645-G08|BOLD:AAK6032
Oscinella|GMPJA10217-21|BIOUG70232-C07|BOLD:AAK6032
Oscinella|GMPJA10199-21|BIOUG70232-B01|BOLD:AAK6032
Oscinella|GMPJA10577-21|BIOUG70236-A11|BOLD:AAK6032
Oscinella|GMPJA10372-21|BIOUG70233-H07|BOLD:AAK6032
Oscinella|GMPJA10231-21|BIOUG70232-D09|BOLD:AAK6032
Oscinella|GMPJA8800-21|BIOUG66646-E12|BOLD:AAK6032
Oscinella|GMPJA10326-21|BIOUG70233-D09|BOLD:AAK6032
Oscinella|GMPJA10261-21|BIOUG70232-G03|BOLD:AAK6032
Oscinella|GMPJA10232-21|BIOUG70232-D10|BOLD:AAK6032
Oscinella|GMPJA10222-21|BIOUG70232-C12|BOLD:AAK6032
Oscinella|GMPJA054-21|BIOUG65656-A05|BOLD:AAK6032
Oscinella|GMPJA515-21|BIOUG65660-H02|BOLD:AAK6032
Oscinella|GMPJA416-21|BIOUG65659-G10|BOLD:AAK6032
Oscinella|GMPJA4165-21|BIOUG65698-G08|BOLD:AAK6032
Oscinella|GMPJA5093-21|BIOUG66607-E10|BOLD:AAK6032
Oscinella|GMPJA5464-21|BIOUG66611-E01|BOLD:AAK6032
Oscinella|GMPJA071-21|BIOUG65656-B10|BOLD:AAK6032
Oscinella|GMPJA059-21|BIOUG65656-A10|BOLD:AAK6032
Oscinella|GMPJA5111-21|BIOUG66607-G04|BOLD:AAK6032
Oscinella|GMPJA5094-21|BIOUG66607-E11|BOLD:AAK6032
Oscinella|GMPJA081-21|BIOUG65656-C08|BOLD:AAK6032
Oscinella|GMPJA074-21|BIOUG65656-C01|BOLD:AAK6032
Oscinella|GMPJA5293-21|BIOUG66609-F08|BOLD:AAK6032
Oscinella|GMPJA5197-21|BIOUG66608-F07|BOLD:AAK6032
Oscinella|GMPJA5590-21|BIOUG66612-G08|BOLD:AAK6032
Oscinella|GMPJA5472-21|BIOUG66611-E09|BOLD:AAK6032
Oscinella|GMPJA6703-21|BIOUG66624-E05|BOLD:AAK6032
Oscinella|GMPJA6581-21|BIOUG66623-C02|BOLD:AAK6032
Oscinella|GMPJA7836-21|BIOUG66636-D10|BOLD:AAK6032
Oscinella|GMPJA7830-21|BIOUG66636-D04|BOLD:AAK6032
Oscinella|GMPJA146-21|BIOUG65657-A02|BOLD:AAK6032
Oscinella|GMPJA101-21|BIOUG65656-E04|BOLD:AAK6032
Oscinella|GMPJA148-21|BIOUG65657-A04|BOLD:AAK6032
Oscinella|GMPJA172-21|BIOUG65657-C04|BOLD:AAK6032
Oscinella|GMPJA209-21|BIOUG65657-F05|BOLD:AAK6032
Oscinella|GMPJA218-21|BIOUG65657-G02|BOLD:AAK6032
Oscinella|GMPJA487-21|BIOUG65660-E10|BOLD:AAK6032
Oscinella|GMPJA7648-21|BIOUG66634-D12|BOLD:AAK6032
Oscinella|GMPJA10343-21|BIOUG70233-F02|BOLD:AAK6032
Oscinella|GMPJA376-21|BIOUG65659-D06|BOLD:AAK6032
Oscinella|GMPJA106-21|BIOUG65656-E09|BOLD:AAK6032
Oscinella|GMPJA104-21|BIOUG65656-E07|BOLD:AAK6032
Diptera|GMPJA090-21|BIOUG65656-D05
Oscinella|GMPJA087-21|BIOUG65656-D02|BOLD:AAK6032
Oscinella|GMPJA4070-21|BIOUG65697-G08|BOLD:AAK6032
Oscinella|GMPJA202-21|BIOUG65657-E10|BOLD:AAK6032
Oscinella|GMPJA3163-21|BIOUG65688-C04|BOLD:AAK6032
Oscinella|GMPJA8058-21|BIOUG66638-G06|BOLD:AAK6032
Oscinella|GMPJA7339-21|BIOUG66631-B12|BOLD:AAK6032
Oscinella|GMPJA5361-21|BIOUG66610-D05|BOLD:AAK6032
Oscinella|GMPJA10356-21|BIOUG70233-G03|BOLD:AAK6032
Oscinella|GMPJA5530-21|BIOUG66612-B08|BOLD:AAK6032
Oscinella|GMPJA5336-21|BIOUG66610-B04|BOLD:AAK6032
Oscinella|GMPJA7470-21|BIOUG66632-E12|BOLD:AAK6032
Oscinella|GMPJA5183-21|BIOUG66608-E05|BOLD:AAK6032
Oscinella|GMPJA8041-21|BIOUG66638-F01|BOLD:AAK6032
Oscinella|GMPJA6429-21|BIOUG66621-F04|BOLD:AAK6032
Oscinella|GMPJA082-21|BIOUG65656-C09|BOLD:AAK6032
Oscinella|GMPJA5051-21|BIOUG66607-B04|BOLD:AAK6032
Oscinella|GMPJA3201-21|BIOUG65688-F06|BOLD:AEJ4416
Oscinella|GMPJA2198-21|BIOUG65678-E11|BOLD:ABZ3963
Oscinella|GMPJA4294-21|BIOUG65700-B07|BOLD:ABZ3963
Oscinella|GMPJA214-21|BIOUG65657-F10|BOLD:ABZ3963
Oscinella|GMPJA167-21|BIOUG65657-B11|BOLD:ABZ3963
Oscinella|GMPJA10570-21|BIOUG70236-A04|BOLD:ABZ3963
Oscinella|GMPJA5480-21|BIOUG66611-F05|BOLD:ABZ3963
Oscinella|GMPJA5105-21|BIOUG66607-F10|BOLD:ABZ3963
Oscinella|GMPJA196-21|BIOUG65657-E04|BOLD:ABZ3963
Oscinella|GMPJA1834-21|BIOUG65674-G03|BOLD:ABZ3963
Oscinella|GMPJA3244-21|BIOUG65689-B02|BOLD:ABZ3963
Oscinella|GMPJA4277-21|BIOUG65700-A02|BOLD:ABZ3963
Oscinella|GMPJA1192-21|BIOUG65668-A03|BOLD:ABZ3963

Oscinella|GMPJA3244-21|BIOUG65689-B02|BOLD:ABZ3963
Oscinella|GMPJA4277-21|BIOUG65700-A02|BOLD:ABZ3963
Oscinella|GMPJA1192-21|BIOUG65668-A03|BOLD:ABZ3963
Oscinella|GMPJA1968-21|BIOUG65676-B07|BOLD:ABZ3963
Oscinella|GMPJA149-21|BIOUG65657-A05|BOLD:ABZ3963
Oscinella|GMPJA3633-21|BIOUG65693-B11|BOLD:ABZ3963
Oscinella|GMPJA5311-21|BIOUG66609-H02|BOLD:ABZ3963
Oscinella|GMPJA5360-21|BIOUG66610-D04|BOLD:ABZ3963
Oscinella|GMPJA3142-21|BIOUG65688-A07|BOLD:ABZ3963
Oscinella|GMPJA6849-21|BIOUG66626-A09|BOLD:ABZ3963
Oscinella|GMPJA1818-21|BIOUG65674-E11|BOLD:ABZ3963
Oscinella|GMPJA1401-21|BIOUG65670-B10|BOLD:ABZ3963
Oscinella|GMPJA10293-21|BIOUG70233-A12|BOLD:ABZ3963
Oscinella|GMPJA1394-21|BIOUG65670-B03|BOLD:ABZ3963
Oscinella|GMPJA4060-21|BIOUG65697-F10|BOLD:ABZ3963
Oscinella|GMPJA5385-21|BIOUG66610-F05|BOLD:ABZ3963
Oscinella|GMPJA170-21|BIOUG65657-C02|BOLD:ABZ3963
Oscinella|GMPJA070-21|BIOUG65656-B09|BOLD:ABZ3963
Oscinella|GMPJA098-21|BIOUG65656-E01|BOLD:ABZ3963
Oscinella|GMPJA3463-21|BIOUG65691-D07|BOLD:ABZ3963
Oscinella|GMPJA4278-21|BIOUG65700-A03|BOLD:ABZ3963
Oscinella|GMPJA1659-21|BIOUG65672-H06|BOLD:ABZ3963
Oscinella|GMPJA3407-21|BIOUG65690-G10|BOLD:ABZ3963
Oscinella|GMPJA10245-21|BIOUG70232-E11|BOLD:ABZ3963
Oscinella|GMPJA3025-21|BIOUG65686-G08|BOLD:ABZ3963
Oscinella|GMPJA5531-21|BIOUG66612-B09|BOLD:ABZ3963
Oscinella|GMPJA10666-21|BIOUG70237-A05|BOLD:ABZ3963
Oscinella|GMPJA10329-21|BIOUG70233-D12|BOLD:ABZ3963
Oscinella|GMPJA10612-21|BIOUG70236-D10|BOLD:ABZ3963
Oscinella|GMPJA4237-21|BIOUG65699-E09|BOLD:ABZ3963
Oscinella|GMPJA4311-21|BIOUG65700-C12|BOLD:ABZ3963
Oscinella|GMPJA4352-21|BIOUG65700-G05|BOLD:ABZ3963
Oscinella|GMPJA4104-21|BIOUG65698-B07|BOLD:ABZ3963
Oscinella|GMPJA4148-21|BIOUG65698-F03|BOLD:ABZ3963
Oscinella|GMPJA404-21|BIOUG65659-F10|BOLD:ABZ3963
Oscinella|GMPJA429-21|BIOUG65659-H11|BOLD:ABZ3963
Oscinella|GMPJA469-21|BIOUG65660-D04|BOLD:ABZ3963
Oscinella|GMPJA1502-21|BIOUG65671-C04|BOLD:ABZ3963
Oscinella|GMPJA1646-21|BIOUG65672-G05|BOLD:ABZ3963
Oscinella|GMPJA3093-21|BIOUG65687-E05|BOLD:ABZ3963
Oscinella|GMPJA8201-21|BIOUG66640-C07|BOLD:ABZ3963
Oscinella|GMPJA4254-21|BIOUG65699-G02|BOLD:ABZ3963
Oscinella|GMPJA5316-21|BIOUG66609-H07|BOLD:ABZ3963
Oscinella|GMPJA5175-21|BIOUG66608-D09|BOLD:ABZ3963
Oscinella|GMPJA5044-21|BIOUG66607-A09|BOLD:ABZ3963
Oscinella|GMPJA2150-21|BIOUG65678-A11|BOLD:ABZ3963
Oscinella|GMPJA1895-21|BIOUG65675-D05|BOLD:ABZ3963
Oscinella|GMPJA091-21|BIOUG65656-D06|BOLD:ABZ3963
Oscinella|GMPJA3522-21|BIOUG65692-A07|BOLD:ABZ3963
Oscinella|GMPJA1031-21|BIOUG65666-C08|BOLD:ABZ3963
Oscinella|GMPJA747-21|BIOUG65663-C09|BOLD:ABZ3963
Oscinella|GMPJA5547-21|BIOUG66612-D01|BOLD:ABZ3963
Oscinella|GMPJA1595-21|BIOUG65672-C02|BOLD:ABZ3963
Oscinella|GMPJA3665-21|BIOUG65693-E07|BOLD:ABZ3963
Oscinella|GMPJA4221-21|BIOUG65699-D05|BOLD:ABZ3963
Oscinella|GMPJA4472-21|BIOUG66602-A07|BOLD:ABZ3963
Oscinella|GMPJA10202-21|BIOUG70232-B04|BOLD:ABZ3963
Oscinella|GMPJA7391-21|BIOUG66631-G04|BOLD:ABZ3963
Oscinella|GMPJA5508-21|BIOUG66611-H09|BOLD:ABZ3963
Oscinella|GMPJA3209-21|BIOUG65688-G02|BOLD:ABZ3963
Oscinella|GMPJA3173-21|BIOUG65688-D02|BOLD:ABZ3963
Oscinella|GMPJA1961-21|BIOUG65676-A12|BOLD:ABZ3963
Oscinella|GMPJA986-21|BIOUG65665-G10|BOLD:ABZ3963
Oscinella|GMPJA495-21|BIOUG65660-F06|BOLD:ABZ3963
Oscinella|GMPJA3317-21|BIOUG65689-H03|BOLD:ABZ3963
Oscinella|GMPJA142-21|BIOUG65656-H09|BOLD:ABZ3963
Oscinella|GMPJA5495-21|BIOUG66611-G08|BOLD:ABZ3963
Oscinella|GMPJA3520-21|BIOUG65692-A05|BOLD:ABZ3963
Oscinella|GMPJA10339-21|BIOUG70233-E10|BOLD:ABZ3963
Oscinella|GMPJA10319-21|BIOUG70233-D02|BOLD:ABZ3963
Oscinella|GMPJA10559-21|BIOUG70235-H04|BOLD:ABZ3963
Oscinella|GMPJA3154-21|BIOUG65688-B07|BOLD:ABZ3963
Oscinella|GMPJA10312-21|BIOUG70233-C07|BOLD:ABZ3963
Oscinella|GMPJA1315-21|BIOUG65669-C07|BOLD:ABZ3963
Oscinella|GMPJA5603-21|BIOUG66612-H09|BOLD:ABZ3963
Oscinella|GMPJA5527-21|BIOUG66612-B05|BOLD:ABZ3963
Oscinella|GMPJA5455-21|BIOUG66611-D04|BOLD:ABZ3963
Oscinella|GMPJA5085-21|BIOUG66607-E02|BOLD:ABZ3963
Oscinella|GMPJA5146-21|BIOUG66608-B04|BOLD:ABZ3963
Oscinella|GMPJA5462-21|BIOUG66611-D11|BOLD:ABZ3963
Oscinella|GMPJA8674-21|BIOUG66645-C05|BOLD:ABZ3963
Oscinella|GMPJA7936-21|BIOUG66637-E03|BOLD:ABZ3963
Oscinella|GMPJA3353-21|BIOUG65690-C04|BOLD:ABZ3963
Oscinella|GMPJA10565-21|BIOUG70235-H10|BOLD:ABZ3963
Oscinella|GMPJA5284-21|BIOUG66609-E11|BOLD:ABZ3963
Oscinella|GMPJA5269-21|BIOUG66609-D08|BOLD:ABZ3963
Oscinella|GMPJA4262-21|BIOUG65699-G10|BOLD:ABZ3963
Oscinella|GMPJA3586-21|BIOUG65692-F11|BOLD:ABZ3963
Oscinella|GMPJA126-21|BIOUG65656-G05|BOLD:ABZ3963
Oscinella|GMPJA4111-21|BIOUG65698-C02|BOLD:ABZ3963
Oscinella|GMPJA351-21|BIOUG65659-B05|BOLD:ABZ3963
Oscinella|GMPJA1195-21|BIOUG65668-A06|BOLD:ABZ3963
Oscinella|GMPJA1908-21|BIOUG65675-E06|BOLD:ABZ3963
Oscinella|GMPJA2203-21|BIOUG65678-F04|BOLD:ABZ3963
Oscinella|GMPJA2206-21|BIOUG65678-F07|BOLD:ABZ3963
Oscinella|GMPJA1686-21|BIOUG65673-B10|BOLD:ABZ3963
Oscinella|GMPJA1735-21|BIOUG65673-F11|BOLD:ABZ3963
Oscinella|GMPJA967-21|BIOUG65665-F03|BOLD:ABZ3963
Oscinella|GMPJA3068-21|BIOUG65687-C04|BOLD:ABZ3963
Oscinella|GMPJA5082-21|BIOUG66607-D11|BOLD:ABZ3963
Oscinella|GMPJA2763-21|BIOUG65684-E06|BOLD:ABZ3963
Oscinella|GMPJA2435-21|BIOUG65681-A11|BOLD:ABZ3963
Eudorylas|GMPJA7541-21|BIOUG66633-C12|BOLD:ACO3871

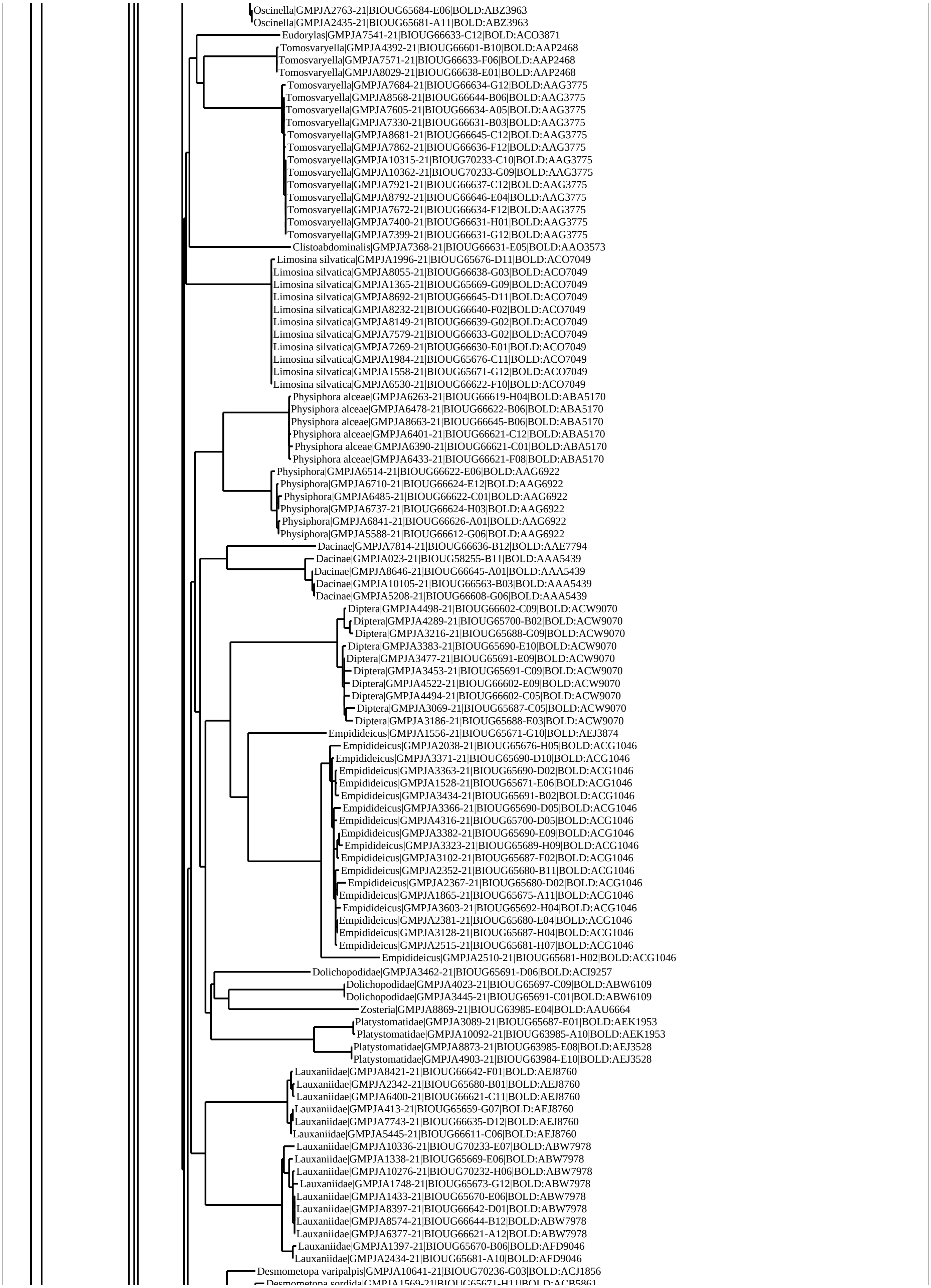
Oscinella|GMPJA2763-21|BIOUG65684-E06|BOLD:ABZ3963
Oscinella|GMPJA2435-21|BIOUG65681-A11|BOLD:ABZ3963
Eudorylas|GMPJA7541-21|BIOUG66633-C12|BOLD:ACO3871
Tomosvaryella|GMPJA4392-21|BIOUG66601-B10|BOLD:AAP2468
Tomosvaryella|GMPJA7571-21|BIOUG66633-F06|BOLD:AAP2468
Tomosvaryella|GMPJA8029-21|BIOUG66638-E01|BOLD:AAP2468
Tomosvaryella|GMPJA7684-21|BIOUG66634-G12|BOLD:AAG3775
Tomosvaryella|GMPJA8568-21|BIOUG66644-B06|BOLD:AAG3775
Tomosvaryella|GMPJA7605-21|BIOUG66634-A05|BOLD:AAG3775
Tomosvaryella|GMPJA7330-21|BIOUG66631-B03|BOLD:AAG3775
Tomosvaryella|GMPJA8681-21|BIOUG66645-C12|BOLD:AAG3775
Tomosvaryella|GMPJA7862-21|BIOUG66636-F12|BOLD:AAG3775
Tomosvaryella|GMPJA10315-21|BIOUG70233-C10|BOLD:AAG3775
Tomosvaryella|GMPJA10362-21|BIOUG70233-G09|BOLD:AAG3775
Tomosvaryella|GMPJA7921-21|BIOUG66637-C12|BOLD:AAG3775
Tomosvaryella|GMPJA8792-21|BIOUG66646-E04|BOLD:AAG3775
Tomosvaryella|GMPJA7672-21|BIOUG66634-F12|BOLD:AAG3775
Tomosvaryella|GMPJA7400-21|BIOUG66631-H01|BOLD:AAG3775
Tomosvaryella|GMPJA7399-21|BIOUG66631-G12|BOLD:AAG3775
Clistoabdominalis|GMPJA7368-21|BIOUG66631-E05|BOLD:AAO3573
Limosina silvatica|GMPJA1996-21|BIOUG65676-D11|BOLD:ACO7049
Limosina silvatica|GMPJA8055-21|BIOUG66638-G03|BOLD:ACO7049
Limosina silvatica|GMPJA1365-21|BIOUG65669-G09|BOLD:ACO7049
Limosina silvatica|GMPJA8692-21|BIOUG66645-D11|BOLD:ACO7049
Limosina silvatica|GMPJA8232-21|BIOUG66640-F02|BOLD:ACO7049
Limosina silvatica|GMPJA8149-21|BIOUG66639-G02|BOLD:ACO7049
Limosina silvatica|GMPJA7579-21|BIOUG66633-G02|BOLD:ACO7049
Limosina silvatica|GMPJA7269-21|BIOUG66630-E01|BOLD:ACO7049
Limosina silvatica|GMPJA1984-21|BIOUG65676-C11|BOLD:ACO7049
Limosina silvatica|GMPJA1558-21|BIOUG65671-G12|BOLD:ACO7049
Limosina silvatica|GMPJA6530-21|BIOUG66622-F10|BOLD:ACO7049
Physiphora alceae|GMPJA6263-21|BIOUG66619-H04|BOLD:ABA5170
Physiphora alceae|GMPJA6478-21|BIOUG66622-B06|BOLD:ABA5170
Physiphora alceae|GMPJA8663-21|BIOUG66645-B06|BOLD:ABA5170
Physiphora alceae|GMPJA6401-21|BIOUG66621-C12|BOLD:ABA5170
Physiphora alceae|GMPJA6390-21|BIOUG66621-C01|BOLD:ABA5170
Physiphora alceae|GMPJA6433-21|BIOUG66621-F08|BOLD:ABA5170
Physiphora|GMPJA6514-21|BIOUG66622-E06|BOLD:AAG6922
Physiphora|GMPJA6710-21|BIOUG66624-E12|BOLD:AAG6922
Physiphora|GMPJA6485-21|BIOUG66622-C01|BOLD:AAG6922
Physiphora|GMPJA6737-21|BIOUG66624-H03|BOLD:AAG6922
Physiphora|GMPJA6841-21|BIOUG66626-A01|BOLD:AAG6922
Physiphora|GMPJA5588-21|BIOUG66612-G06|BOLD:AAG6922
Dacinae|GMPJA7814-21|BIOUG66636-B12|BOLD:AAE7794
Dacinae|GMPJA023-21|BIOUG58255-B11|BOLD:AAA5439
Dacinae|GMPJA8646-21|BIOUG66645-A01|BOLD:AAA5439
Dacinae|GMPJA10105-21|BIOUG66563-B03|BOLD:AAA5439
Dacinae|GMPJA5208-21|BIOUG66608-G06|BOLD:AAA5439
Diptera|GMPJA4498-21|BIOUG66602-C09|BOLD:ACW9070
Diptera|GMPJA4289-21|BIOUG65700-B02|BOLD:ACW9070
Diptera|GMPJA3216-21|BIOUG65688-G09|BOLD:ACW9070
Diptera|GMPJA3383-21|BIOUG65690-E10|BOLD:ACW9070
Diptera|GMPJA3477-21|BIOUG65691-E09|BOLD:ACW9070
Diptera|GMPJA3453-21|BIOUG65691-C09|BOLD:ACW9070
Diptera|GMPJA4522-21|BIOUG66602-E09|BOLD:ACW9070
Diptera|GMPJA4494-21|BIOUG66602-C05|BOLD:ACW9070
Diptera|GMPJA3069-21|BIOUG65687-C05|BOLD:ACW9070
Diptera|GMPJA3186-21|BIOUG65688-E03|BOLD:ACW9070
Empidideicus|GMPJA1556-21|BIOUG65671-G10|BOLD:AEJ3874
Empidideicus|GMPJA2038-21|BIOUG65676-H05|BOLD:ACG1046
Empidideicus|GMPJA3371-21|BIOUG65690-D10|BOLD:ACG1046
Empidideicus|GMPJA3363-21|BIOUG65690-D02|BOLD:ACG1046
Empidideicus|GMPJA1528-21|BIOUG65671-E06|BOLD:ACG1046
Empidideicus|GMPJA3434-21|BIOUG65691-B02|BOLD:ACG1046
Empidideicus|GMPJA3366-21|BIOUG65690-D05|BOLD:ACG1046
Empidideicus|GMPJA4316-21|BIOUG65700-D05|BOLD:ACG1046
Empidideicus|GMPJA3382-21|BIOUG65690-E09|BOLD:ACG1046
Empidideicus|GMPJA3323-21|BIOUG65689-H09|BOLD:ACG1046
Empidideicus|GMPJA3102-21|BIOUG65687-F02|BOLD:ACG1046
Empidideicus|GMPJA2352-21|BIOUG65680-B11|BOLD:ACG1046
Empidideicus|GMPJA2367-21|BIOUG65680-D02|BOLD:ACG1046
Empidideicus|GMPJA1865-21|BIOUG65675-A11|BOLD:ACG1046
Empidideicus|GMPJA3603-21|BIOUG65692-H04|BOLD:ACG1046
Empidideicus|GMPJA2381-21|BIOUG65680-E04|BOLD:ACG1046
Empidideicus|GMPJA3128-21|BIOUG65687-H04|BOLD:ACG1046
Empidideicus|GMPJA2515-21|BIOUG65681-H07|BOLD:ACG1046
Empidideicus|GMPJA2510-21|BIOUG65681-H02|BOLD:ACG1046
Dolichopodidae|GMPJA3462-21|BIOUG65691-D06|BOLD:ACI9257
Dolichopodidae|GMPJA4023-21|BIOUG65697-C09|BOLD:ABW6109
Dolichopodidae|GMPJA3445-21|BIOUG65691-C01|BOLD:ABW6109
Zosteria|GMPJA8869-21|BIOUG63985-E04|BOLD:AAU6664
Platystomatidae|GMPJA3089-21|BIOUG65687-E01|BOLD:AEK1953
Platystomatidae|GMPJA10092-21|BIOUG63985-A10|BOLD:AEK1953
Platystomatidae|GMPJA8873-21|BIOUG63985-E08|BOLD:AEJ3528
Platystomatidae|GMPJA4903-21|BIOUG63984-E10|BOLD:AEJ3528
Lauxaniidae|GMPJA8421-21|BIOUG66642-F01|BOLD:AEJ8760
Lauxaniidae|GMPJA2342-21|BIOUG65680-B01|BOLD:AEJ8760
Lauxaniidae|GMPJA6400-21|BIOUG66621-C11|BOLD:AEJ8760
Lauxaniidae|GMPJA413-21|BIOUG65659-G07|BOLD:AEJ8760
Lauxaniidae|GMPJA7743-21|BIOUG66635-D12|BOLD:AEJ8760
Lauxaniidae|GMPJA5445-21|BIOUG66611-C06|BOLD:AEJ8760
Lauxaniidae|GMPJA10336-21|BIOUG70233-E07|BOLD:ABW7978
Lauxaniidae|GMPJA1338-21|BIOUG65669-E06|BOLD:ABW7978
Lauxaniidae|GMPJA10276-21|BIOUG70232-H06|BOLD:ABW7978
Lauxaniidae|GMPJA1748-21|BIOUG65673-G12|BOLD:ABW7978
Lauxaniidae|GMPJA1433-21|BIOUG65670-E06|BOLD:ABW7978
Lauxaniidae|GMPJA8397-21|BIOUG66642-D01|BOLD:ABW7978
Lauxaniidae|GMPJA8574-21|BIOUG66644-B12|BOLD:ABW7978
Lauxaniidae|GMPJA6377-21|BIOUG66621-A12|BOLD:ABW7978
Lauxaniidae|GMPJA1397-21|BIOUG65670-B06|BOLD:AFD9046
Lauxaniidae|GMPJA2434-21|BIOUG65681-A10|BOLD:AFD9046
Desmometopa varipalpis|GMPJA10641-21|BIOUG70236-G03|BOLD:ACJ1856
Desmometopa sordida|GMPJA1569-21|BIOUG65671-H11|BOLD:ACB5861

[illegible]
Lauxaniidae|GMPJA2434-21|BIOUG65681-A10|BOLD:AFD9046
Desmometopa varipalpis|GMPJA10641-21|BIOUG70236-G03|BOLD:ACJ1856
Desmometopa sordida|GMPJA1569-21|BIOUG65671-H11|BOLD:ACB5861
Desmometopa sordida|GMPJA3476-21|BIOUG65691-E08|BOLD:ACB5861
Desmometopa sordida|GMPJA3108-21|BIOUG65687-F08|BOLD:ACB5861
Desmometopa sordida|GMPJA3358-21|BIOUG65690-C09|BOLD:ACB5861
Desmometopa sordida|GMPJA2382-21|BIOUG65680-E05|BOLD:ACB5861
Desmometopa sordida|GMPJA3494-21|BIOUG65691-G02|BOLD:ACB5861
Desmometopa sordida|GMPJA1973-21|BIOUG65676-B12|BOLD:ACB5861
Desmometopa sordida|GMPJA3043-21|BIOUG65687-A03|BOLD:ACB5861
Desmometopa sordida|GMPJA3279-21|BIOUG65689-E01|BOLD:ACB5861
Oscinellinae|GMPJA6503-21|BIOUG66622-D07|BOLD:AAG1505
Oscinellinae|GMPJA10272-21|BIOUG70232-H02|BOLD:AAG1505
Oscinellinae|GMPJA222-21|BIOUG65657-G06|BOLD:AAG1505
Oscinellinae|GMPJA6249-21|BIOUG66619-G02|BOLD:AAG1505
Oscinellinae|GMPJA10608-21|BIOUG70236-D06|BOLD:AAG1505
Oscinellinae|GMPJA10280-21|BIOUG70232-H10|BOLD:AAG1505
Oscinellinae|GMPJA7824-21|BIOUG66636-C10|BOLD:AAG1505
Oscinellinae|GMPJA6702-21|BIOUG66624-E04|BOLD:AAG1505
Oscinellinae|GMPJA6546-21|BIOUG66622-H02|BOLD:AAG1505
Oscinellinae|GMPJA6540-21|BIOUG66622-G08|BOLD:AAG1505
Oscinellinae|GMPJA5088-21|BIOUG66607-E05|BOLD:AAG1505
Oscinellinae|GMPJA5322-21|BIOUG66610-A02|BOLD:AAG1505
Oscinellinae|GMPJA3626-21|BIOUG65693-B04|BOLD:AAG1505
Oscinellinae|GMPJA8289-21|BIOUG66641-B12|BOLD:AAG1505
Oscinellinae|GMPJA6826-21|BIOUG66625-G09|BOLD:AAG1505
Oscinellinae|GMPJA6599-21|BIOUG66623-D08|BOLD:AAG1505
Oscinellinae|GMPJA6470-21|BIOUG66622-A10|BOLD:AAG1505
Oscinellinae|GMPJA8643-21|BIOUG66644-H09|BOLD:AAG1505
Oscinellinae|GMPJA6905-21|BIOUG66626-F05|BOLD:AAG1505
Oscinellinae|GMPJA6892-21|BIOUG66626-E04|BOLD:AAG1505
Oscinellinae|GMPJA6494-21|BIOUG66622-C10|BOLD:AAG1505
Oscinellinae|GMPJA1987-21|BIOUG65676-D02|BOLD:AAG1505
Oscinellinae|GMPJA684-21|BIOUG65662-F05|BOLD:AAG1505
Oscinellinae|GMPJA10306-21|BIOUG70233-C01|BOLD:AAG1505
Oscinellinae|GMPJA7532-21|BIOUG66633-C03|BOLD:AAG1505
Oscinellinae|GMPJA6833-21|BIOUG66625-H04|BOLD:AAG1505
Oscinellinae|GMPJA6832-21|BIOUG66625-H03|BOLD:AAG1505
Oscinellinae|GMPJA6665-21|BIOUG66624-B03|BOLD:AAG1505
Oscinellinae|GMPJA6641-21|BIOUG66623-H02|BOLD:AAG1505
Oscinellinae|GMPJA6606-21|BIOUG66623-E03|BOLD:AAG1505
Oscinellinae|GMPJA5327-21|BIOUG66610-A07|BOLD:AAG1505
Oscinellinae|GMPJA7293-21|BIOUG66630-G01|BOLD:AAG1505
Oscinellinae|GMPJA4476-21|BIOUG66602-A11|BOLD:AAG1505
Oscinellinae|GMPJA6616-21|BIOUG66623-F01|BOLD:AAG1505
Oscinellinae|GMPJA6513-21|BIOUG66622-E05|BOLD:AAG1505
Oscinellinae|GMPJA8598-21|BIOUG66644-D12|BOLD:AAG1505
Oscinellinae|GMPJA8204-21|BIOUG66640-C10|BOLD:AAG1505
Oscinellinae|GMPJA4590-21|BIOUG66603-C06|BOLD:AAG1505
Oscinellinae|GMPJA6649-21|BIOUG66623-H10|BOLD:AAG1505
Oscinellinae|GMPJA6457-21|BIOUG66621-H08|BOLD:AAG1505
Oscinellinae|GMPJA3924-21|BIOUG65696-C05|BOLD:AAG1505
Oscinellinae|GMPJA4368-21|BIOUG65700-H09|BOLD:AAG1505
Oscinellinae|GMPJA6869-21|BIOUG66626-C05|BOLD:AAG1505
Oscinellinae|GMPJA6827-21|BIOUG66625-G10|BOLD:AAG1505
Oscinellinae|GMPJA6491-21|BIOUG66622-C07|BOLD:AAG1505
Oscinellinae|GMPJA6476-21|BIOUG66622-B04|BOLD:AAG1505
Oscinellinae|GMPJA7861-21|BIOUG66636-F11|BOLD:AAG1505
Oscinellinae|GMPJA8714-21|BIOUG66645-F09|BOLD:AAG1505
Oscinellinae|GMPJA6630-21|BIOUG66623-G03|BOLD:AAG1505
Oscinellinae|GMPJA6394-21|BIOUG66621-C05|BOLD:AAG1505
Oscinellinae|GMPJA6388-21|BIOUG66621-B11|BOLD:AAG1505
Oscinellinae|GMPJA6572-21|BIOUG66623-B05|BOLD:AAG1505
Oscinellinae|GMPJA8138-21|BIOUG66639-F03|BOLD:AAG1505
Oscinellinae|GMPJA5349-21|BIOUG66610-C05|BOLD:AAG1505
Oscinellinae|GMPJA5265-21|BIOUG66609-D04|BOLD:AAG1505
Oscinellinae|GMPJA5484-21|BIOUG66611-F09|BOLD:AAG1505
Oscinellinae|GMPJA5506-21|BIOUG66611-H07|BOLD:AAG1505
Oscinellinae|GMPJA6427-21|BIOUG66621-F02|BOLD:AAG1505
Oscinellinae|GMPJA6453-21|BIOUG66621-H04|BOLD:AAG1505
Oscinellinae|GMPJA6369-21|BIOUG66621-A04|BOLD:AAG1505
Oscinellinae|GMPJA6382-21|BIOUG66621-B05|BOLD:AAG1505
Oscinellinae|GMPJA6385-21|BIOUG66621-B08|BOLD:AAG1505
Oscinellinae|GMPJA6391-21|BIOUG66621-C02|BOLD:AAG1505
Oscinellinae|GMPJA6396-21|BIOUG66621-C07|BOLD:AAG1505
Oscinellinae|GMPJA6397-21|BIOUG66621-C08|BOLD:AAG1505
Oscinellinae|GMPJA6403-21|BIOUG66621-D02|BOLD:AAG1505
Oscinellinae|GMPJA6420-21|BIOUG66621-E07|BOLD:AAG1505
Oscinellinae|GMPJA6423-21|BIOUG66621-E10|BOLD:AAG1505
Oscinellinae|GMPJA6518-21|BIOUG66622-E10|BOLD:AAG1505
Oscinellinae|GMPJA6571-21|BIOUG66623-B04|BOLD:AAG1505
Oscinellinae|GMPJA6582-21|BIOUG66623-C03|BOLD:AAG1505
Oscinellinae|GMPJA6585-21|BIOUG66623-C06|BOLD:AAG1505
Oscinellinae|GMPJA6602-21|BIOUG66623-D11|BOLD:AAG1505
Oscinellinae|GMPJA6610-21|BIOUG66623-E07|BOLD:AAG1505
Oscinellinae|GMPJA6617-21|BIOUG66623-F02|BOLD:AAG1505
Oscinellinae|GMPJA6625-21|BIOUG66623-F10|BOLD:AAG1505
Oscinellinae|GMPJA6642-21|BIOUG66623-H03|BOLD:AAG1505
Oscinellinae|GMPJA6658-21|BIOUG66624-A08|BOLD:AAG1505
Oscinellinae|GMPJA6660-21|BIOUG66624-A10|BOLD:AAG1505
Oscinellinae|GMPJA6677-21|BIOUG66624-C03|BOLD:AAG1505
Oscinellinae|GMPJA6679-21|BIOUG66624-C05|BOLD:AAG1505
Oscinellinae|GMPJA6691-21|BIOUG66624-D05|BOLD:AAG1505
Oscinellinae|GMPJA6752-21|BIOUG66625-A07|BOLD:AAG1505
Oscinellinae|GMPJA6755-21|BIOUG66625-A10|BOLD:AAG1505
Oscinellinae|GMPJA6760-21|BIOUG66625-B03|BOLD:AAG1505
Oscinellinae|GMPJA6788-21|BIOUG66625-D07|BOLD:AAG1505
Oscinellinae|GMPJA6795-21|BIOUG66625-E02|BOLD:AAG1505
Oscinellinae|GMPJA7362-21|BIOUG66631-D11|BOLD:AAG1505
Oscinellinae|GMPJA7414-21|BIOUG66632-A04|BOLD:AAG1505
Oscinellinae|GMPJA6842-21|BIOUG66626-A02|BOLD:AAG1505
Oscinellinae|GMPJA6867-21|BIOUG66626-C03|BOLD:AAG1505
Oscinellinae|GMPJA6872-21|BIOUG66626-C08|BOLD:AAG1505

Oscinellinae|GMPJA7414-21|BIOUG66632-A04|BOLD:AAG1505
Oscinellinae|GMPJA6842-21|BIOUG66626-A02|BOLD:AAG1505
Oscinellinae|GMPJA6867-21|BIOUG66626-C03|BOLD:AAG1505
Oscinellinae|GMPJA6872-21|BIOUG66626-C08|BOLD:AAG1505
Oscinellinae|GMPJA6894-21|BIOUG66626-E06|BOLD:AAG1505
Oscinellinae|GMPJA7680-21|BIOUG66634-G08|BOLD:AAG1505
Oscinellinae|GMPJA7306-21|BIOUG66630-H02|BOLD:AAG1505
Oscinellinae|GMPJA8003-21|BIOUG66638-B11|BOLD:AAG1505
Oscinellinae|GMPJA7931-21|BIOUG66637-D10|BOLD:AAG1505
Oscinellinae|GMPJA8163-21|BIOUG66639-H04|BOLD:AAG1505
Oscinellinae|GMPJA8255-21|BIOUG66640-H01|BOLD:AAG1505
Oscinellinae|GMPJA8724-21|BIOUG66645-G07|BOLD:AAG1505
Oscinellinae|GMPJA10214-21|BIOUG70232-C04|BOLD:AAG1505
Oscinellinae|GMPJA10227-21|BIOUG70232-D05|BOLD:AAG1505
Oscinellinae|GMPJA10289-21|BIOUG70233-A08|BOLD:AAG1505
Oscinellinae|GMPJA10357-21|BIOUG70233-G04|BOLD:AAG1505
Oscinellinae|GMPJA10206-21|BIOUG70232-B08|BOLD:AAG1505
Oscinellinae|GMPJA4580-21|BIOUG66603-B08|BOLD:AAG1505
Oscinellinae|GMPJA4587-21|BIOUG66603-C03|BOLD:AAG1505
Oscinellinae|GMPJA10266-21|BIOUG70232-G08|BOLD:AAG1505
Oscinellinae|GMPJA3047-21|BIOUG65687-A07|BOLD:AAG1505
Oscinellinae|GMPJA10301-21|BIOUG70233-B08|BOLD:AAG1505
Oscinellinae|GMPJA10302-21|BIOUG70233-B09|BOLD:AAG1505
Oscinellinae|GMPJA10310-21|BIOUG70233-C05|BOLD:AAG1505
Oscinellinae|GMPJA10318-21|BIOUG70233-D01|BOLD:AAG1505
Oscinellinae|GMPJA10569-21|BIOUG70236-A03|BOLD:AAG1505
Oscinellinae|GMPJA10600-21|BIOUG70236-C10|BOLD:AAG1505
Oscinellinae|GMPJA4351-21|BIOUG65700-G04|BOLD:AAG1505
Oscinellinae|GMPJA4369-21|BIOUG65700-H10|BOLD:AAG1505
Oscinellinae|GMPJA4372-21|BIOUG66601-A02|BOLD:AAG1505
Oscinellinae|GMPJA10670-21|BIOUG70237-A09|BOLD:AAG1505
Oscinellinae|GMPJA4185-21|BIOUG65699-A05|BOLD:AAG1505
Oscinellinae|GMPJA414-21|BIOUG65659-G08|BOLD:AAG1505
Oscinellinae|GMPJA6566-21|BIOUG66623-A11|BOLD:AAG1505
Oscinellinae|GMPJA10292-21|BIOUG70233-A11|BOLD:AAG1505
Oscinellinae|GMPJA8178-21|BIOUG66640-A08|BOLD:AAG1505
Oscinellinae|GMPJA5158-21|BIOUG66608-C04|BOLD:AAG1505
Oscinellinae|GMPJA5153-21|BIOUG66608-B11|BOLD:AAG1505
Oscinellinae|GMPJA5122-21|BIOUG66607-H03|BOLD:AAG1505
Oscinellinae|GMPJA5086-21|BIOUG66607-E03|BOLD:AAG1505
Madizinae|GMPJA8544-21|BIOUG66643-H05|BOLD:AEK0835
Desmometopa|GMPJA4022-21|BIOUG65697-C08|BOLD:ACR6117
Desmometopa|GMPJA5188-21|BIOUG66608-E10|BOLD:ACR6117
Drosophila|GMPJA169-21|BIOUG65657-C01|BOLD:AAE8038
Scaptomyza pallida|GMPJA2009-21|BIOUG65676-E12|BOLD:AAG8493
Scaptomyza pallida|GMPJA1841-21|BIOUG65674-G10|BOLD:AAG8493
Scaptomyza pallida|GMPJA1514-21|BIOUG65671-D04|BOLD:AAG8493
Scaptomyza pallida|GMPJA757-21|BIOUG65663-D07|BOLD:AAG8493
Scaptomyza pallida|GMPJA750-21|BIOUG65663-C12|BOLD:AAG8493
Scaptomyza pallida|GMPJA1426-21|BIOUG65670-D11|BOLD:AAG8493
Scaptomyza pallida|GMPJA2223-21|BIOUG65678-G12|BOLD:AAG8493
Scaptomyza pallida|GMPJA2420-21|BIOUG65680-H07|BOLD:AAG8493
Scaptomyza pallida|GMPJA1491-21|BIOUG65671-B05|BOLD:AAG8493
Scaptomyza pallida|GMPJA1407-21|BIOUG65670-C04|BOLD:AAG8493
Scaptomyza pallida|GMPJA130-21|BIOUG65656-G09|BOLD:AAG8493
Leptocera|GMPJA2146-21|BIOUG65678-A07|BOLD:ACO3855
Leptocera|GMPJA2222-21|BIOUG65678-G11|BOLD:ACO3855
Sphaeroceridae|GMPJA2252-21|BIOUG65679-B06|BOLD:ACS6009
Sphaeroceridae|GMPJA215-21|BIOUG65657-F11|BOLD:ACS6009
Sphaeroceridae|GMPJA198-21|BIOUG65657-E06|BOLD:ACS6009
Sphaeroceridae|GMPJA132-21|BIOUG65656-G11|BOLD:ACS6009
Lamproscatella|GMPJA1169-21|BIOUG65667-G03|BOLD:ABX2137
Ephydridae|GMPJA2126-21|BIOUG65677-G10|BOLD:ACW1379
Ephydridae|GMPJA8723-21|BIOUG66645-G06|BOLD:ACW1379
Ephydridae|GMPJA6527-21|BIOUG66622-F07|BOLD:AEJ6528
Sepsis thoracica|GMPJA1759-21|BIOUG65673-H11|BOLD:AAJ7614
Sepsis thoracica|GMPJA2235-21|BIOUG65679-A01|BOLD:AAJ7614
Sepsis thoracica|GMPJA1706-21|BIOUG65673-D06|BOLD:AAJ7614
Sepsis thoracica|GMPJA1642-21|BIOUG65672-G01|BOLD:AAJ7614
Diptera|GMPJA4383-21|BIOUG66601-B01
Scatella|GMPJA213-21|BIOUG65657-F09|BOLD:ACZ6583
Scatella|GMPJA997-21|BIOUG65665-H09|BOLD:ACZ6583
Scatella|GMPJA2027-21|BIOUG65676-G06|BOLD:ACZ6583
Scatella|GMPJA1055-21|BIOUG65666-E08|BOLD:ACZ6583
Scatella|GMPJA1945-21|BIOUG65675-H07|BOLD:ACZ6583
Scatella|GMPJA1339-21|BIOUG65669-E07|BOLD:ACZ6583
Scatella|GMPJA973-21|BIOUG65665-F09|BOLD:ACZ6583
Scatella|GMPJA1444-21|BIOUG65670-F05|BOLD:ACZ6583
Scatella|GMPJA2155-21|BIOUG65678-B04|BOLD:ACZ6583
Scatella|GMPJA1421-21|BIOUG65670-D06|BOLD:ACZ6583
Scatella|GMPJA1551-21|BIOUG65671-G05|BOLD:ACZ6583
Scatella|GMPJA1190-21|BIOUG65668-A01|BOLD:ACZ6583
Scatella|GMPJA2385-21|BIOUG65680-E08|BOLD:ACZ6583
Scatella|GMPJA2020-21|BIOUG65676-F11|BOLD:ACZ6583
Scatella|GMPJA1032-21|BIOUG65666-C09|BOLD:ACZ6583
Scatella|GMPJA426-21|BIOUG65659-H08|BOLD:ACZ6583
Scatella|GMPJA1069-21|BIOUG65666-F10|BOLD:ACZ6583
Scatella|GMPJA1907-21|BIOUG65675-E05|BOLD:ACZ6583
Scatella|GMPJA1184-21|BIOUG65667-H06|BOLD:ACZ6583
Scatella|GMPJA1525-21|BIOUG65671-E03|BOLD:ACZ6583
Scatella|GMPJA676-21|BIOUG65662-E09|BOLD:ACZ6583
Scatella|GMPJA663-21|BIOUG65662-D08|BOLD:ACZ6583
Scatella|GMPJA649-21|BIOUG65662-C06|BOLD:ACZ6583
Scatella|GMPJA1064-21|BIOUG65666-F05|BOLD:ACZ6583
Scatella|GMPJA1187-21|BIOUG65667-H09|BOLD:ACZ6583
Scatella|GMPJA4178-21|BIOUG65698-H09|BOLD:ACZ6583
Scatella|GMPJA1418-21|BIOUG65670-D03|BOLD:ACZ6583
Scatella|GMPJA2305-21|BIOUG65679-F11|BOLD:ACZ6583
Scatella|GMPJA2423-21|BIOUG65680-H10|BOLD:ACZ6583
Scatella|GMPJA2413-21|BIOUG65680-G12|BOLD:ACZ6583
Scatella|GMPJA2409-21|BIOUG65680-G08|BOLD:ACZ6583
Scatella|GMPJA2081-21|BIOUG65677-D01|BOLD:ACZ6583
Scatella|GMPJA2028-21|BIOUG65676-G07|BOLD:ACZ6583
Scatella|GMPJA[illegible]-21|BIOUG[illegible]|BOLD:ACZ6583

[illegible]
Scatella|GMPJA2081-21|BIOUG65677-D01|BOLD:ACZ6583
Scatella|GMPJA2028-21|BIOUG65676-G07|BOLD:ACZ6583
Scatella|GMPJA2283-21|BIOUG65679-E01|BOLD:ACZ6583
Scatella|GMPJA1179-21|BIOUG65667-H01|BOLD:ACZ6583
Scatella|GMPJA700-21|BIOUG65662-G09|BOLD:ACZ6583
Scatella|GMPJA1201-21|BIOUG65668-A12|BOLD:ACZ6583
Scatella|GMPJA683-21|BIOUG65662-F04|BOLD:ACZ6583
Scatella|GMPJA062-21|BIOUG65656-B01|BOLD:ACZ6583
Scatella|GMPJA963-21|BIOUG65665-E11|BOLD:ACZ6583
Scatella|GMPJA457-21|BIOUG65660-C04|BOLD:ACZ6583
Scatella|GMPJA1189-21|BIOUG65667-H11|BOLD:ACZ6583
Scatella|GMPJA713-21|BIOUG65662-H10|BOLD:ACZ6583
Scatella|GMPJA4361-21|BIOUG65700-H02|BOLD:ACZ6583
Scatella|GMPJA6375-21|BIOUG66621-A10|BOLD:ACZ6583
Scatella|GMPJA1962-21|BIOUG65676-B01|BOLD:ACZ6583
Scatella|GMPJA961-21|BIOUG65665-E09|BOLD:ACZ6583
Scatella|GMPJA659-21|BIOUG65662-D04|BOLD:ACZ6583
Scatella|GMPJA1122-21|BIOUG65667-C04|BOLD:ACZ6583
Scatella|GMPJA1185-21|BIOUG65667-H07|BOLD:ACZ6583
Scatella|GMPJA1008-21|BIOUG65666-A09|BOLD:ACZ6583
Scatella|GMPJA1028-21|BIOUG65666-C05|BOLD:ACZ6583
Scatella|GMPJA992-21|BIOUG65665-H04|BOLD:ACZ6583
Scatella|GMPJA1132-21|BIOUG65667-D02|BOLD:ACZ6583
Scatella|GMPJA926-21|BIOUG65665-B10|BOLD:ACZ6583
Scatella|GMPJA2757-21|BIOUG65684-D12|BOLD:ACZ6583
Scatella|GMPJA945-21|BIOUG65665-D05|BOLD:ACZ6583
Scatella|GMPJA965-21|BIOUG65665-F01|BOLD:ACZ6583
Scatella|GMPJA628-21|BIOUG65662-A09|BOLD:ACZ6583
Scatella|GMPJA668-21|BIOUG65662-E01|BOLD:ACZ6583
Scatella|GMPJA670-21|BIOUG65662-E03|BOLD:ACZ6583
Scatella|GMPJA685-21|BIOUG65662-F06|BOLD:ACZ6583
Scatella|GMPJA1006-21|BIOUG65666-A07|BOLD:ACZ6583
Scatella|GMPJA1092-21|BIOUG65666-H09|BOLD:ACZ6583
Scatella|GMPJA1101-21|BIOUG65667-A07|BOLD:ACZ6583
Scatella|GMPJA1109-21|BIOUG65667-B03|BOLD:ACZ6583
Scatella|GMPJA1114-21|BIOUG65667-B08|BOLD:ACZ6583
Scatella|GMPJA1134-21|BIOUG65667-D04|BOLD:ACZ6583
Scatella|GMPJA1159-21|BIOUG65667-F05|BOLD:ACZ6583
Scatella|GMPJA1358-21|BIOUG65669-G02|BOLD:ACZ6583
Scatella|GMPJA1971-21|BIOUG65676-B10|BOLD:ACZ6583
Scatella|GMPJA2074-21|BIOUG65677-C06|BOLD:ACZ6583
Scatella|GMPJA1082-21|BIOUG65666-G11|BOLD:ACZ6583
Scatella|GMPJA949-21|BIOUG65665-D09|BOLD:ACZ6583
Scatella|GMPJA994-21|BIOUG65665-H06|BOLD:ACZ6583
Scatella|GMPJA1015-21|BIOUG65666-B04|BOLD:ACZ6583
Scatella|GMPJA1013-21|BIOUG65666-B02|BOLD:ACZ6583
Scatella|GMPJA1007-21|BIOUG65666-A08|BOLD:ACZ6583
Scatella|GMPJA1005-21|BIOUG65666-A06|BOLD:ACZ6583
Scatella|GMPJA1004-21|BIOUG65666-A05|BOLD:ACZ6583
Scatella|GMPJA998-21|BIOUG65665-H10|BOLD:ACZ6583
Scatella|GMPJA719-21|BIOUG65663-A05|BOLD:ACZ6583
Scatella|GMPJA712-21|BIOUG65662-H09|BOLD:ACZ6583
Scatella|GMPJA1093-21|BIOUG65666-H10|BOLD:ACZ6583
Scatella|GMPJA1091-21|BIOUG65666-H08|BOLD:ACZ6583
Scatella|GMPJA1086-21|BIOUG65666-H03|BOLD:ACZ6583
Scatella|GMPJA1081-21|BIOUG65666-G10|BOLD:ACZ6583
Scatella|GMPJA707-21|BIOUG65662-H04|BOLD:ACZ6583
Scatella|GMPJA699-21|BIOUG65662-G08|BOLD:ACZ6583
Scatella|GMPJA1325-21|BIOUG65669-D05|BOLD:ACZ6583
Scatella|GMPJA1318-21|BIOUG65669-C10|BOLD:ACZ6583
Scatella|GMPJA654-21|BIOUG65662-C11|BOLD:ACZ6583
Scatella|GMPJA650-21|BIOUG65662-C07|BOLD:ACZ6583
Scatella|GMPJA632-21|BIOUG65662-B01|BOLD:ACZ6583
Scatella|GMPJA620-21|BIOUG65662-A01|BOLD:ACZ6583
Scatella|GMPJA362-21|BIOUG65659-C04|BOLD:ACZ6583
Scatella|GMPJA975-21|BIOUG65665-F11|BOLD:ACZ6583
Scatella|GMPJA1070-21|BIOUG65666-F11|BOLD:ACZ6583
Scatella|GMPJA1067-21|BIOUG65666-F08|BOLD:ACZ6583
Scatella|GMPJA1066-21|BIOUG65666-F07|BOLD:ACZ6583
Scatella|GMPJA1061-21|BIOUG65666-F02|BOLD:ACZ6583
Scatella|GMPJA1476-21|BIOUG65671-A02|BOLD:ACZ6583
Scatella|GMPJA1440-21|BIOUG65670-F01|BOLD:ACZ6583
Scatella|GMPJA974-21|BIOUG65665-F10|BOLD:ACZ6583
Scatella|GMPJA970-21|BIOUG65665-F06|BOLD:ACZ6583
Scatella|GMPJA1058-21|BIOUG65666-E11|BOLD:ACZ6583
Scatella|GMPJA1054-21|BIOUG65666-E07|BOLD:ACZ6583
Scatella|GMPJA1052-21|BIOUG65666-E05|BOLD:ACZ6583
Scatella|GMPJA1046-21|BIOUG65666-D11|BOLD:ACZ6583
Scatella|GMPJA969-21|BIOUG65665-F05|BOLD:ACZ6583
Scatella|GMPJA959-21|BIOUG65665-E07|BOLD:ACZ6583
Scatella|GMPJA955-21|BIOUG65665-E03|BOLD:ACZ6583
Scatella|GMPJA944-21|BIOUG65665-D04|BOLD:ACZ6583
Scatella|GMPJA943-21|BIOUG65665-D03|BOLD:ACZ6583
Scatella|GMPJA935-21|BIOUG65665-C07|BOLD:ACZ6583
Scatella|GMPJA933-21|BIOUG65665-C05|BOLD:ACZ6583
Scatella|GMPJA927-21|BIOUG65665-B11|BOLD:ACZ6583
Scatella|GMPJA925-21|BIOUG65665-B09|BOLD:ACZ6583
Scatella|GMPJA914-21|BIOUG65665-A10|BOLD:ACZ6583
Scatella|GMPJA1144-21|BIOUG65667-E02|BOLD:ACZ6583
Scatella|GMPJA1142-21|BIOUG65667-D12|BOLD:ACZ6583
Scatella|GMPJA1039-21|BIOUG65666-D04|BOLD:ACZ6583
Scatella|GMPJA1034-21|BIOUG65666-C11|BOLD:ACZ6583
Scatella|GMPJA060-21|BIOUG65656-A11|BOLD:ACZ6583
Scatella|GMPJA499-21|BIOUG65660-F10|BOLD:ACZ6583
Scatella|GMPJA1133-21|BIOUG65667-D03|BOLD:ACZ6583
Scatella|GMPJA1128-21|BIOUG65667-C10|BOLD:ACZ6583
Scatella|GMPJA1025-21|BIOUG65666-C02|BOLD:ACZ6583
Scatella|GMPJA1018-21|BIOUG65666-B07|BOLD:ACZ6583
Scatella|GMPJA137-21|BIOUG65656-H04|BOLD:ACZ6583
Scatella|GMPJA4243-21|BIOUG65699-F03|BOLD:ACZ6583
Scatella|GMPJA3451-21|BIOUG65691-C07|BOLD:ACZ6583
Scatella|GMPJA10195-21|BIOUG70232-A09|BOLD:ACZ6583
[illegible]

Scatella|GMPJA4243-21|BIOUG65699-F03|BOLD:ACZ6583
Scatella|GMPJA3451-21|BIOUG65691-C07|BOLD:ACZ6583
Scatella|GMPJA10195-21|BIOUG70232-A09|BOLD:ACZ6583
Scatella|GMPJA8639-21|BIOUG66644-H05|BOLD:ACZ6583
Scatella|GMPJA8313-21|BIOUG66641-D12|BOLD:ACZ6583
Scatella|GMPJA1147-21|BIOUG65667-E05|BOLD:ACZ6583
Scatella|GMPJA1146-21|BIOUG65667-E04|BOLD:ACZ6583
Scatella|GMPJA1438-21|BIOUG65670-E11|BOLD:ACZ6583
Scatella|GMPJA1873-21|BIOUG65675-B07|BOLD:ACZ6583
Scatella|GMPJA1585-21|BIOUG65672-B04|BOLD:ACZ6583
Scatella|GMPJA1583-21|BIOUG65672-B02|BOLD:ACZ6583
Scatella|GMPJA1544-21|BIOUG65671-F10|BOLD:ACZ6583
Scatella|GMPJA1541-21|BIOUG65671-F07|BOLD:ACZ6583
Scatella|GMPJA1538-21|BIOUG65671-F04|BOLD:ACZ6583
Scatella|GMPJA1535-21|BIOUG65671-F01|BOLD:ACZ6583
Scatella|GMPJA1872-21|BIOUG65675-B06|BOLD:ACZ6583
Scatella|GMPJA1870-21|BIOUG65675-B04|BOLD:ACZ6583
Scatella|GMPJA1828-21|BIOUG65674-F09|BOLD:ACZ6583
Scatella|GMPJA1813-21|BIOUG65674-E06|BOLD:ACZ6583
Scatella|GMPJA1802-21|BIOUG65674-D07|BOLD:ACZ6583
Scatella|GMPJA1373-21|BIOUG65669-H05|BOLD:ACZ6583
Scatella|GMPJA1193-21|BIOUG65668-A04|BOLD:ACZ6583
Scatella|GMPJA1191-21|BIOUG65668-A02|BOLD:ACZ6583
Scatella|GMPJA1186-21|BIOUG65667-H08|BOLD:ACZ6583
Scatella|GMPJA1182-21|BIOUG65667-H04|BOLD:ACZ6583
Scatella|GMPJA1180-21|BIOUG65667-H02|BOLD:ACZ6583
Scatella|GMPJA1178-21|BIOUG65667-G12|BOLD:ACZ6583
Scatella|GMPJA1175-21|BIOUG65667-G09|BOLD:ACZ6583
Scatella|GMPJA1164-21|BIOUG65667-F10|BOLD:ACZ6583
Scatella|GMPJA1161-21|BIOUG65667-F07|BOLD:ACZ6583
Scatella|GMPJA1151-21|BIOUG65667-E09|BOLD:ACZ6583
Scatella|GMPJA1127-21|BIOUG65667-C09|BOLD:ACZ6583
Scatella|GMPJA1123-21|BIOUG65667-C05|BOLD:ACZ6583
Scatella|GMPJA1110-21|BIOUG65667-B04|BOLD:ACZ6583
Scatella|GMPJA1108-21|BIOUG65667-B02|BOLD:ACZ6583
Scatella|GMPJA1097-21|BIOUG65667-A03|BOLD:ACZ6583
Scatella|GMPJA1096-21|BIOUG65667-A02|BOLD:ACZ6583
Scatella|GMPJA710-21|BIOUG65662-H07|BOLD:ACZ6583
Scatella|GMPJA709-21|BIOUG65662-H06|BOLD:ACZ6583
Scatella|GMPJA2186-21|BIOUG65678-D11|BOLD:ACZ6583
Scatella|GMPJA1611-21|BIOUG65672-D06|BOLD:ACZ6583
Scatella|GMPJA1295-21|BIOUG65669-A11|BOLD:ACZ6583
Scatella|GMPJA669-21|BIOUG65662-E02|BOLD:ACZ6583
Scatella|GMPJA734-21|BIOUG65663-B08|BOLD:ACZ6583
Scatella|GMPJA731-21|BIOUG65663-B05|BOLD:ACZ6583
Scatella|GMPJA984-21|BIOUG65665-G08|BOLD:ACZ6583
Scatella|GMPJA746-21|BIOUG65663-C08|BOLD:ACZ6583
Scatella|GMPJA996-21|BIOUG65665-H08|BOLD:ACZ6583
Scatella|GMPJA995-21|BIOUG65665-H07|BOLD:ACZ6583
Scatella|GMPJA2248-21|BIOUG65679-B02|BOLD:ACZ6583
Scatella|GMPJA2193-21|BIOUG65678-E06|BOLD:ACZ6583
Scatella|GMPJA2277-21|BIOUG65679-D07|BOLD:ACZ6583
Scatella|GMPJA2259-21|BIOUG65679-C01|BOLD:ACZ6583
Scatella|GMPJA1113-21|BIOUG65667-B07|BOLD:ACZ6583
Scatella|GMPJA1722-21|BIOUG65673-E10|BOLD:ACZ6583
Scatella|GMPJA1734-21|BIOUG65673-F10|BOLD:ACZ6583
Scatella|GMPJA1749-21|BIOUG65673-H01|BOLD:ACZ6583
Scatella|GMPJA1778-21|BIOUG65674-B07|BOLD:ACZ6583
Scatella|GMPJA1953-21|BIOUG65676-A04|BOLD:ACZ6583
Scatella|GMPJA1990-21|BIOUG65676-D05|BOLD:ACZ6583
Scatella|GMPJA2069-21|BIOUG65677-C01|BOLD:ACZ6583
Scatella|GMPJA1105-21|BIOUG65667-A11|BOLD:ACZ6583
Scatella|GMPJA1976-21|BIOUG65676-C03|BOLD:ACZ6583
Scatella|GMPJA2017-21|BIOUG65676-F08|BOLD:ACZ6583
Scatella|GMPJA1507-21|BIOUG65671-C09|BOLD:ACZ6583
Scatella|GMPJA2365-21|BIOUG65680-C12|BOLD:ACZ6583
Scatella|GMPJA1026-21|BIOUG65666-C03|BOLD:ACZ6583
Scatella|GMPJA1062-21|BIOUG65666-F03|BOLD:ACZ6583
Scatella|GMPJA1553-21|BIOUG65671-G07|BOLD:ACZ6583
Scatella|GMPJA1173-21|BIOUG65667-G07|BOLD:ACZ6583
Ephydrinae|GMPJA4187-21|BIOUG65699-A07|BOLD:ACZ6583
Scatella|GMPJA2472-21|BIOUG65681-D12|BOLD:ACZ6583
Scatella|GMPJA1634-21|BIOUG65672-F05|BOLD:ACZ6583
Scatella|GMPJA912-21|BIOUG65665-A08|BOLD:ACZ6583
Scatella|GMPJA735-21|BIOUG65663-B09|BOLD:ACZ6583
Scatella|GMPJA526-21|BIOUG65661-A02|BOLD:ACZ6583
Scatella|GMPJA938-21|BIOUG65665-C10|BOLD:ACZ6583
Scatella|GMPJA1079-21|BIOUG65666-G08|BOLD:ACZ6583
Scatella|GMPJA1116-21|BIOUG65667-B10|BOLD:ACZ6583
Scatella|GMPJA1149-21|BIOUG65667-E07|BOLD:ACZ6583
Scatella|GMPJA1509-21|BIOUG65671-C11|BOLD:ACZ6583
Scatella|GMPJA1682-21|BIOUG65673-B06|BOLD:ACZ6583
Scatella|GMPJA665-21|BIOUG65662-D10|BOLD:ACZ6583
Scatella|GMPJA726-21|BIOUG65663-A12|BOLD:ACZ6583
Scatella|GMPJA765-21|BIOUG65663-E03|BOLD:ACZ6583
Scatella|GMPJA1020-21|BIOUG65666-B09|BOLD:ACZ6583
Scatella|GMPJA1022-21|BIOUG65666-B11|BOLD:ACZ6583
Scatella|GMPJA1170-21|BIOUG65667-G04|BOLD:ACZ6583
Scatella|GMPJA1934-21|BIOUG65675-G08|BOLD:ACZ6583
Scatella|GMPJA2166-21|BIOUG65678-C03|BOLD:ACZ6583
Scatella|GMPJA911-21|BIOUG65665-A07|BOLD:ACZ6583
Scatella|GMPJA968-21|BIOUG65665-F04|BOLD:ACZ6583
Scatella|GMPJA1021-21|BIOUG65666-B10|BOLD:ACZ6583
Scatella|GMPJA971-21|BIOUG65665-F07|BOLD:ACZ6583
Scatella|GMPJA2767-21|BIOUG65684-E10|BOLD:ACZ6583
Scatella|GMPJA653-21|BIOUG65662-C10|BOLD:ACZ6583
Scatella|GMPJA1087-21|BIOUG65666-H04|BOLD:ACZ6583
Scatella|GMPJA1158-21|BIOUG65667-F04|BOLD:ACZ6583
Scatella|GMPJA1654-21|BIOUG65672-H01|BOLD:ACZ6583
Scatella|GMPJA1714-21|BIOUG65673-E02|BOLD:ACZ6583
Scatella|GMPJA1975-21|BIOUG65676-C02|BOLD:ACZ6583
Scatella|GMPJA1991-21|BIOUG65676-D06|BOLD:ACZ6583
Scatella|GMPJA2051-21|BIOUG65677-A07|BOLD:ACZ6583
[illegible]

Scatella|GMPJA1975-21|BIOUG65676-C02|BOLD:ACZ6583
Scatella|GMPJA1991-21|BIOUG65676-D06|BOLD:ACZ6583
Scatella|GMPJA2051-21|BIOUG65677-A07|BOLD:ACZ6583
Scatella|GMPJA2115-21|BIOUG65677-F11|BOLD:ACZ6583
Scatella|GMPJA2118-21|BIOUG65677-G02|BOLD:ACZ6583
Scatella|GMPJA2123-21|BIOUG65677-G07|BOLD:ACZ6583
Scatella|GMPJA4489-21|BIOUG66602-B12|BOLD:ACZ6583
Scatella|GMPJA2158-21|BIOUG65678-B07|BOLD:ACZ6583
Scatella|GMPJA2372-21|BIOUG65680-D07|BOLD:ACZ6583
Scatella|GMPJA2397-21|BIOUG65680-F08|BOLD:ACZ6583
Scatella|GMPJA1183-21|BIOUG65667-H05|BOLD:ACZ6583
Scatella|GMPJA643-21|BIOUG65662-B12|BOLD:ACZ6583
Scatella|GMPJA937-21|BIOUG65665-C09|BOLD:ACZ6583
Scatella|GMPJA2185-21|BIOUG65678-D10|BOLD:ACZ6583
Scatella|GMPJA2164-21|BIOUG65678-C01|BOLD:ACZ6583
Scatella|GMPJA3106-21|BIOUG65687-F06|BOLD:ACZ6583
Scatella|GMPJA8697-21|BIOUG66645-E04|BOLD:ACZ6583
Scatella|GMPJA2486-21|BIOUG65681-F02|BOLD:ACZ6583
Scatella|GMPJA442-21|BIOUG65660-B01|BOLD:ACZ6583
Scatella|GMPJA134-21|BIOUG65656-H01|BOLD:ACZ6583
Scatella|GMPJA165-21|BIOUG65657-B09|BOLD:ACZ6583
Scatella|GMPJA078-21|BIOUG65656-C05|BOLD:ACZ6583
Scatella|GMPJA906-21|BIOUG65665-A02|BOLD:ACZ6583
Scatella|GMPJA189-21|BIOUG65657-D09|BOLD:ACZ6583
Scatella|GMPJA956-21|BIOUG65665-E04|BOLD:ACZ6583
Scatella|GMPJA950-21|BIOUG65665-D10|BOLD:ACZ6583
Scatella|GMPJA939-21|BIOUG65665-C11|BOLD:ACZ6583
Scatella|GMPJA936-21|BIOUG65665-C08|BOLD:ACZ6583
Scatella|GMPJA929-21|BIOUG65665-C01|BOLD:ACZ6583
Scatella|GMPJA924-21|BIOUG65665-B08|BOLD:ACZ6583
Scatella|GMPJA923-21|BIOUG65665-B07|BOLD:ACZ6583
Scatella|GMPJA908-21|BIOUG65665-A04|BOLD:ACZ6583
Scatella|GMPJA982-21|BIOUG65665-G06|BOLD:ACZ6583
Scatella|GMPJA980-21|BIOUG65665-G04|BOLD:ACZ6583
Scatella|GMPJA630-21|BIOUG65662-A11|BOLD:ACZ6583
Scatella|GMPJA358-21|BIOUG65659-B12|BOLD:ACZ6583
Scatella|GMPJA637-21|BIOUG65662-B06|BOLD:ACZ6583
Scatella|GMPJA635-21|BIOUG65662-B04|BOLD:ACZ6583
Scatella|GMPJA671-21|BIOUG65662-E04|BOLD:ACZ6583
Scatella|GMPJA661-21|BIOUG65662-D06|BOLD:ACZ6583
Scatella|GMPJA703-21|BIOUG65662-G12|BOLD:ACZ6583
Scatella|GMPJA674-21|BIOUG65662-E07|BOLD:ACZ6583
Scatella|GMPJA1137-21|BIOUG65667-D07|BOLD:ACZ6583
Scatella|GMPJA1126-21|BIOUG65667-C08|BOLD:ACZ6583
Scatella|GMPJA720-21|BIOUG65663-A06|BOLD:ACZ6583
Scatella|GMPJA704-21|BIOUG65662-H01|BOLD:ACZ6583
Scatella|GMPJA1000-21|BIOUG65666-A01|BOLD:ACZ6583
Scatella|GMPJA991-21|BIOUG65665-H03|BOLD:ACZ6583
Scatella|GMPJA1042-21|BIOUG65666-D07|BOLD:ACZ6583
Scatella|GMPJA1016-21|BIOUG65666-B05|BOLD:ACZ6583
Scatella|GMPJA1057-21|BIOUG65666-E10|BOLD:ACZ6583
Scatella|GMPJA1049-21|BIOUG65666-E02|BOLD:ACZ6583
Scatella|GMPJA1085-21|BIOUG65666-H02|BOLD:ACZ6583
Scatella|GMPJA1075-21|BIOUG65666-G04|BOLD:ACZ6583
Scatella|GMPJA1411-21|BIOUG65670-C08|BOLD:ACZ6583
Scatella|GMPJA1196-21|BIOUG65668-A07|BOLD:ACZ6583
Scatella|GMPJA1100-21|BIOUG65667-A06|BOLD:ACZ6583
Scatella|GMPJA1098-21|BIOUG65667-A04|BOLD:ACZ6583
Scatella|GMPJA1165-21|BIOUG65667-F11|BOLD:ACZ6583
Scatella|GMPJA1143-21|BIOUG65667-E01|BOLD:ACZ6583
Scatella|GMPJA1181-21|BIOUG65667-H03|BOLD:ACZ6583
Scatella|GMPJA1176-21|BIOUG65667-G10|BOLD:ACZ6583
Scatella|GMPJA1574-21|BIOUG65672-A05|BOLD:ACZ6583
Scatella|GMPJA1536-21|BIOUG65671-F02|BOLD:ACZ6583
Scatella|GMPJA1920-21|BIOUG65675-F06|BOLD:ACZ6583
Scatella|GMPJA1820-21|BIOUG65674-F01|BOLD:ACZ6583
Scatella|GMPJA2180-21|BIOUG65678-D05|BOLD:ACZ6583
Scatella|GMPJA1591-21|BIOUG65672-B10|BOLD:ACZ6583
Scatella|GMPJA2469-21|BIOUG65681-D09|BOLD:ACZ6583
Scatella|GMPJA529-21|BIOUG65661-A05|BOLD:ABW7890
Scatella|GMPJA10549-21|BIOUG70235-G06|BOLD:ABW7890
Scatella|GMPJA497-21|BIOUG65660-F08|BOLD:ABW7890
Scatella|GMPJA352-21|BIOUG65659-B06|BOLD:ABW7890
Scatella|GMPJA5537-21|BIOUG66612-C03|BOLD:ABW7890
Scatella|GMPJA1194-21|BIOUG65668-A05|BOLD:ABW7890
Scatella|GMPJA1948-21|BIOUG65675-H10|BOLD:ABW7890
Scatella|GMPJA1773-21|BIOUG65674-B02|BOLD:ABW7890
Scatella|GMPJA2275-21|BIOUG65679-D05|BOLD:ABW7890
Scatella|GMPJA391-21|BIOUG65659-E09|BOLD:ABW7890
Scatella|GMPJA364-21|BIOUG65659-C06|BOLD:ABW7890
Scatella|GMPJA960-21|BIOUG65665-E08|BOLD:ABW7890
Scatella|GMPJA152-21|BIOUG65657-A08|BOLD:ABW7890
Scatella|GMPJA10234-21|BIOUG70232-D12|BOLD:ABW7890
Scatella|GMPJA2442-21|BIOUG65681-B06|BOLD:ABW7890
Sphaeroceridae|GMPJA7337-21|BIOUG66631-B10|BOLD:ADH0761
Sphaeroceridae|GMPJA8252-21|BIOUG66640-G10|BOLD:ADH0761
Sphaeroceridae|GMPJA7282-21|BIOUG66630-F02|BOLD:ADH0761
Milichiella lacteipennis|GMPJA10196-21|BIOUG70232-A10|BOLD:AAG7056
Milichiella lacteipennis|GMPJA6601-21|BIOUG66623-D10|BOLD:AAG7056
Trachyopella leucoptera|GMPJA7263-21|BIOUG66630-D07|BOLD:ACO8232
Sphaeroceridae|GMPJA1336-21|BIOUG65669-E04|BOLD:ACV3269
Sphaeroceridae|GMPJA2214-21|BIOUG65678-G03|BOLD:ACV3269
Sphaeroceridae|GMPJA2310-21|BIOUG65679-G04|BOLD:ACV3269
Sphaeroceridae|GMPJA8179-21|BIOUG66640-A09|BOLD:ACV3269
Sphaeroceridae|GMPJA1578-21|BIOUG65672-A09|BOLD:ACV3269
Sphaeroceridae|GMPJA2374-21|BIOUG65680-D09|BOLD:ACV3269
Sphaeroceridae|GMPJA1702-21|BIOUG65673-D02|BOLD:ACV3269
Sphaeroceridae|GMPJA1879-21|BIOUG65675-C01|BOLD:ACV3269
Sphaeroceridae|GMPJA8430-21|BIOUG66642-F10|BOLD:ACV3269
Sphaeroceridae|GMPJA1459-21|BIOUG65670-G08|BOLD:ACV3269
Sphaeroceridae|GMPJA2192-21|BIOUG65678-E05|BOLD:ACV3269
Sphaeroceridae|GMPJA1519-21|BIOUG65671-D09|BOLD:ACV3269
Sphaeroceridae|GMPJA1799-21|BIOUG65674-D04|BOLD:ACV3269

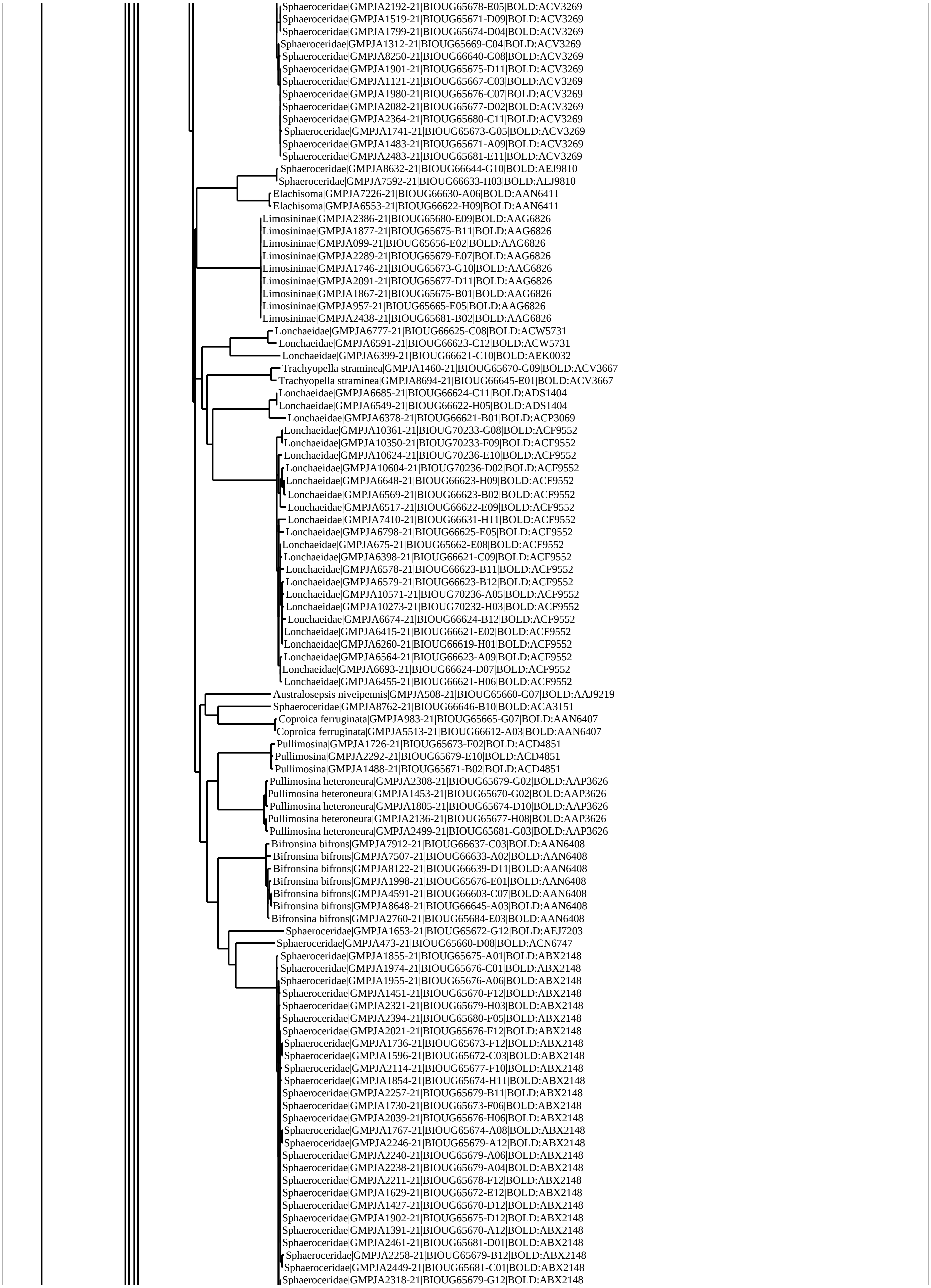
Sphaeroceridae|GMPJA2192-21|BIOUG65678-E05|BOLD:ACV3269
Sphaeroceridae|GMPJA1519-21|BIOUG65671-D09|BOLD:ACV3269
Sphaeroceridae|GMPJA1799-21|BIOUG65674-D04|BOLD:ACV3269
Sphaeroceridae|GMPJA1312-21|BIOUG65669-C04|BOLD:ACV3269
Sphaeroceridae|GMPJA8250-21|BIOUG66640-G08|BOLD:ACV3269
Sphaeroceridae|GMPJA1901-21|BIOUG65675-D11|BOLD:ACV3269
Sphaeroceridae|GMPJA1121-21|BIOUG65667-C03|BOLD:ACV3269
Sphaeroceridae|GMPJA1980-21|BIOUG65676-C07|BOLD:ACV3269
Sphaeroceridae|GMPJA2082-21|BIOUG65677-D02|BOLD:ACV3269
Sphaeroceridae|GMPJA2364-21|BIOUG65680-C11|BOLD:ACV3269
Sphaeroceridae|GMPJA1741-21|BIOUG65673-G05|BOLD:ACV3269
Sphaeroceridae|GMPJA1483-21|BIOUG65671-A09|BOLD:ACV3269
Sphaeroceridae|GMPJA2483-21|BIOUG65681-E11|BOLD:ACV3269
Sphaeroceridae|GMPJA8632-21|BIOUG66644-G10|BOLD:AEJ9810
Sphaeroceridae|GMPJA7592-21|BIOUG66633-H03|BOLD:AEJ9810
Elachisoma|GMPJA7226-21|BIOUG66630-A06|BOLD:AAN6411
Elachisoma|GMPJA6553-21|BIOUG66622-H09|BOLD:AAN6411
Limosininae|GMPJA2386-21|BIOUG65680-E09|BOLD:AAG6826
Limosininae|GMPJA1877-21|BIOUG65675-B11|BOLD:AAG6826
Limosininae|GMPJA099-21|BIOUG65656-E02|BOLD:AAG6826
Limosininae|GMPJA2289-21|BIOUG65679-E07|BOLD:AAG6826
Limosininae|GMPJA1746-21|BIOUG65673-G10|BOLD:AAG6826
Limosininae|GMPJA2091-21|BIOUG65677-D11|BOLD:AAG6826
Limosininae|GMPJA1867-21|BIOUG65675-B01|BOLD:AAG6826
Limosininae|GMPJA957-21|BIOUG65665-E05|BOLD:AAG6826
Limosininae|GMPJA2438-21|BIOUG65681-B02|BOLD:AAG6826
Lonchaeidae|GMPJA6777-21|BIOUG66625-C08|BOLD:ACW5731
Lonchaeidae|GMPJA6591-21|BIOUG66623-C12|BOLD:ACW5731
Lonchaeidae|GMPJA6399-21|BIOUG66621-C10|BOLD:AEK0032
Trachyopella straminea|GMPJA1460-21|BIOUG65670-G09|BOLD:ACV3667
Trachyopella straminea|GMPJA8694-21|BIOUG66645-E01|BOLD:ACV3667
Lonchaeidae|GMPJA6685-21|BIOUG66624-C11|BOLD:ADS1404
Lonchaeidae|GMPJA6549-21|BIOUG66622-H05|BOLD:ADS1404
Lonchaeidae|GMPJA6378-21|BIOUG66621-B01|BOLD:ACP3069
Lonchaeidae|GMPJA10361-21|BIOUG70233-G08|BOLD:ACF9552
Lonchaeidae|GMPJA10350-21|BIOUG70233-F09|BOLD:ACF9552
Lonchaeidae|GMPJA10624-21|BIOUG70236-E10|BOLD:ACF9552
Lonchaeidae|GMPJA10604-21|BIOUG70236-D02|BOLD:ACF9552
Lonchaeidae|GMPJA6648-21|BIOUG66623-H09|BOLD:ACF9552
Lonchaeidae|GMPJA6569-21|BIOUG66623-B02|BOLD:ACF9552
Lonchaeidae|GMPJA6517-21|BIOUG66622-E09|BOLD:ACF9552
Lonchaeidae|GMPJA7410-21|BIOUG66631-H11|BOLD:ACF9552
Lonchaeidae|GMPJA6798-21|BIOUG66625-E05|BOLD:ACF9552
Lonchaeidae|GMPJA675-21|BIOUG65662-E08|BOLD:ACF9552
Lonchaeidae|GMPJA6398-21|BIOUG66621-C09|BOLD:ACF9552
Lonchaeidae|GMPJA6578-21|BIOUG66623-B11|BOLD:ACF9552
Lonchaeidae|GMPJA6579-21|BIOUG66623-B12|BOLD:ACF9552
Lonchaeidae|GMPJA10571-21|BIOUG70236-A05|BOLD:ACF9552
Lonchaeidae|GMPJA10273-21|BIOUG70232-H03|BOLD:ACF9552
Lonchaeidae|GMPJA6674-21|BIOUG66624-B12|BOLD:ACF9552
Lonchaeidae|GMPJA6415-21|BIOUG66621-E02|BOLD:ACF9552
Lonchaeidae|GMPJA6260-21|BIOUG66619-H01|BOLD:ACF9552
Lonchaeidae|GMPJA6564-21|BIOUG66623-A09|BOLD:ACF9552
Lonchaeidae|GMPJA6693-21|BIOUG66624-D07|BOLD:ACF9552
Lonchaeidae|GMPJA6455-21|BIOUG66621-H06|BOLD:ACF9552
Australosepsis niveipennis|GMPJA508-21|BIOUG65660-G07|BOLD:AAJ9219
Sphaeroceridae|GMPJA8762-21|BIOUG66646-B10|BOLD:ACA3151
Coproica ferruginata|GMPJA983-21|BIOUG65665-G07|BOLD:AAN6407
Coproica ferruginata|GMPJA5513-21|BIOUG66612-A03|BOLD:AAN6407
Pullimosina|GMPJA1726-21|BIOUG65673-F02|BOLD:ACD4851
Pullimosina|GMPJA2292-21|BIOUG65679-E10|BOLD:ACD4851
Pullimosina|GMPJA1488-21|BIOUG65671-B02|BOLD:ACD4851
Pullimosina heteroneura|GMPJA2308-21|BIOUG65679-G02|BOLD:AAP3626
Pullimosina heteroneura|GMPJA1453-21|BIOUG65670-G02|BOLD:AAP3626
Pullimosina heteroneura|GMPJA1805-21|BIOUG65674-D10|BOLD:AAP3626
Pullimosina heteroneura|GMPJA2136-21|BIOUG65677-H08|BOLD:AAP3626
Pullimosina heteroneura|GMPJA2499-21|BIOUG65681-G03|BOLD:AAP3626
Bifronsina bifrons|GMPJA7912-21|BIOUG66637-C03|BOLD:AAN6408
Bifronsina bifrons|GMPJA7507-21|BIOUG66633-A02|BOLD:AAN6408
Bifronsina bifrons|GMPJA8122-21|BIOUG66639-D11|BOLD:AAN6408
Bifronsina bifrons|GMPJA1998-21|BIOUG65676-E01|BOLD:AAN6408
Bifronsina bifrons|GMPJA4591-21|BIOUG66603-C07|BOLD:AAN6408
Bifronsina bifrons|GMPJA8648-21|BIOUG66645-A03|BOLD:AAN6408
Bifronsina bifrons|GMPJA2760-21|BIOUG65684-E03|BOLD:AAN6408
Sphaeroceridae|GMPJA1653-21|BIOUG65672-G12|BOLD:AEJ7203
Sphaeroceridae|GMPJA473-21|BIOUG65660-D08|BOLD:ACN6747
Sphaeroceridae|GMPJA1855-21|BIOUG65675-A01|BOLD:ABX2148
Sphaeroceridae|GMPJA1974-21|BIOUG65676-C01|BOLD:ABX2148
Sphaeroceridae|GMPJA1955-21|BIOUG65676-A06|BOLD:ABX2148
Sphaeroceridae|GMPJA1451-21|BIOUG65670-F12|BOLD:ABX2148
Sphaeroceridae|GMPJA2321-21|BIOUG65679-H03|BOLD:ABX2148
Sphaeroceridae|GMPJA2394-21|BIOUG65680-F05|BOLD:ABX2148
Sphaeroceridae|GMPJA2021-21|BIOUG65676-F12|BOLD:ABX2148
Sphaeroceridae|GMPJA1736-21|BIOUG65673-F12|BOLD:ABX2148
Sphaeroceridae|GMPJA1596-21|BIOUG65672-C03|BOLD:ABX2148
Sphaeroceridae|GMPJA2114-21|BIOUG65677-F10|BOLD:ABX2148
Sphaeroceridae|GMPJA1854-21|BIOUG65674-H11|BOLD:ABX2148
Sphaeroceridae|GMPJA2257-21|BIOUG65679-B11|BOLD:ABX2148
Sphaeroceridae|GMPJA1730-21|BIOUG65673-F06|BOLD:ABX2148
Sphaeroceridae|GMPJA2039-21|BIOUG65676-H06|BOLD:ABX2148
Sphaeroceridae|GMPJA1767-21|BIOUG65674-A08|BOLD:ABX2148
Sphaeroceridae|GMPJA2246-21|BIOUG65679-A12|BOLD:ABX2148
Sphaeroceridae|GMPJA2240-21|BIOUG65679-A06|BOLD:ABX2148
Sphaeroceridae|GMPJA2238-21|BIOUG65679-A04|BOLD:ABX2148
Sphaeroceridae|GMPJA2211-21|BIOUG65678-F12|BOLD:ABX2148
Sphaeroceridae|GMPJA1629-21|BIOUG65672-E12|BOLD:ABX2148
Sphaeroceridae|GMPJA1427-21|BIOUG65670-D12|BOLD:ABX2148
Sphaeroceridae|GMPJA1902-21|BIOUG65675-D12|BOLD:ABX2148
Sphaeroceridae|GMPJA1391-21|BIOUG65670-A12|BOLD:ABX2148
Sphaeroceridae|GMPJA2461-21|BIOUG65681-D01|BOLD:ABX2148
Sphaeroceridae|GMPJA2258-21|BIOUG65679-B12|BOLD:ABX2148
Sphaeroceridae|GMPJA2449-21|BIOUG65681-C01|BOLD:ABX2148
Sphaeroceridae|GMPJA2318-21|BIOUG65679-G12|BOLD:ABX2148

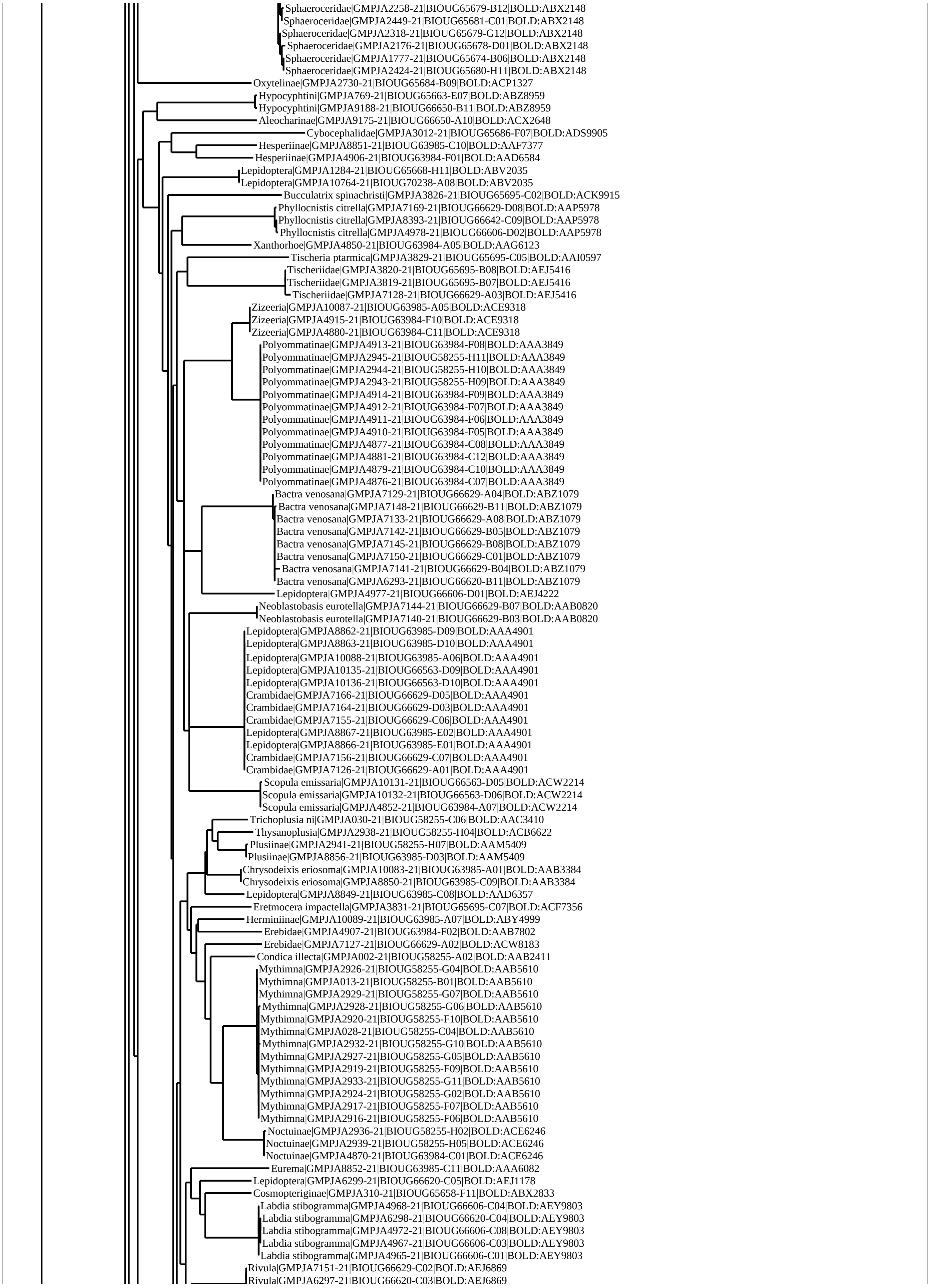
Sphaeroceridae|GMPJA2258-21|BIOUG65679-B12|BOLD:ABX2148
Sphaeroceridae|GMPJA2449-21|BIOUG65681-C01|BOLD:ABX2148
Sphaeroceridae|GMPJA2318-21|BIOUG65679-G12|BOLD:ABX2148
Sphaeroceridae|GMPJA2176-21|BIOUG65678-D01|BOLD:ABX2148
Sphaeroceridae|GMPJA1777-21|BIOUG65674-B06|BOLD:ABX2148
Sphaeroceridae|GMPJA2424-21|BIOUG65680-H11|BOLD:ABX2148
Oxytelinae|GMPJA2730-21|BIOUG65684-B09|BOLD:ACP1327
Hypocyphtini|GMPJA769-21|BIOUG65663-E07|BOLD:ABZ8959
Hypocyphtini|GMPJA9188-21|BIOUG66650-B11|BOLD:ABZ8959
Aleocharinae|GMPJA9175-21|BIOUG66650-A10|BOLD:ACX2648
Cybocephalidae|GMPJA3012-21|BIOUG65686-F07|BOLD:ADS9905
Hesperiinae|GMPJA8851-21|BIOUG63985-C10|BOLD:AAF7377
Hesperiinae|GMPJA4906-21|BIOUG63984-F01|BOLD:AAD6584
Lepidoptera|GMPJA1284-21|BIOUG65668-H11|BOLD:ABV2035
Lepidoptera|GMPJA10764-21|BIOUG70238-A08|BOLD:ABV2035
Bucculatrix spinachristi|GMPJA3826-21|BIOUG65695-C02|BOLD:ACK9915
Phyllocnistis citrella|GMPJA7169-21|BIOUG66629-D08|BOLD:AAP5978
Phyllocnistis citrella|GMPJA8393-21|BIOUG66642-C09|BOLD:AAP5978
Phyllocnistis citrella|GMPJA4978-21|BIOUG66606-D02|BOLD:AAP5978
Xanthorhoe|GMPJA4850-21|BIOUG63984-A05|BOLD:AAG6123
Tischeria ptarmica|GMPJA3829-21|BIOUG65695-C05|BOLD:AAI0597
Tischeriidae|GMPJA3820-21|BIOUG65695-B08|BOLD:AEJ5416
Tischeriidae|GMPJA3819-21|BIOUG65695-B07|BOLD:AEJ5416
Tischeriidae|GMPJA7128-21|BIOUG66629-A03|BOLD:AEJ5416
Zizeeria|GMPJA10087-21|BIOUG63985-A05|BOLD:ACE9318
Zizeeria|GMPJA4915-21|BIOUG63984-F10|BOLD:ACE9318
Zizeeria|GMPJA4880-21|BIOUG63984-C11|BOLD:ACE9318
Polyommatinae|GMPJA4913-21|BIOUG63984-F08|BOLD:AAA3849
Polyommatinae|GMPJA2945-21|BIOUG58255-H11|BOLD:AAA3849
Polyommatinae|GMPJA2944-21|BIOUG58255-H10|BOLD:AAA3849
Polyommatinae|GMPJA2943-21|BIOUG58255-H09|BOLD:AAA3849
Polyommatinae|GMPJA4914-21|BIOUG63984-F09|BOLD:AAA3849
Polyommatinae|GMPJA4912-21|BIOUG63984-F07|BOLD:AAA3849
Polyommatinae|GMPJA4911-21|BIOUG63984-F06|BOLD:AAA3849
Polyommatinae|GMPJA4910-21|BIOUG63984-F05|BOLD:AAA3849
Polyommatinae|GMPJA4877-21|BIOUG63984-C08|BOLD:AAA3849
Polyommatinae|GMPJA4881-21|BIOUG63984-C12|BOLD:AAA3849
Polyommatinae|GMPJA4879-21|BIOUG63984-C10|BOLD:AAA3849
Polyommatinae|GMPJA4876-21|BIOUG63984-C07|BOLD:AAA3849
Bactra venosana|GMPJA7129-21|BIOUG66629-A04|BOLD:ABZ1079
Bactra venosana|GMPJA7148-21|BIOUG66629-B11|BOLD:ABZ1079
Bactra venosana|GMPJA7133-21|BIOUG66629-A08|BOLD:ABZ1079
Bactra venosana|GMPJA7142-21|BIOUG66629-B05|BOLD:ABZ1079
Bactra venosana|GMPJA7145-21|BIOUG66629-B08|BOLD:ABZ1079
Bactra venosana|GMPJA7150-21|BIOUG66629-C01|BOLD:ABZ1079
Bactra venosana|GMPJA7141-21|BIOUG66629-B04|BOLD:ABZ1079
Bactra venosana|GMPJA6293-21|BIOUG66620-B11|BOLD:ABZ1079
Lepidoptera|GMPJA4977-21|BIOUG66606-D01|BOLD:AEJ4222
Neoblastobasis eurotella|GMPJA7144-21|BIOUG66629-B07|BOLD:AAB0820
Neoblastobasis eurotella|GMPJA7140-21|BIOUG66629-B03|BOLD:AAB0820
Lepidoptera|GMPJA8862-21|BIOUG63985-D09|BOLD:AAA4901
Lepidoptera|GMPJA8863-21|BIOUG63985-D10|BOLD:AAA4901
Lepidoptera|GMPJA10088-21|BIOUG63985-A06|BOLD:AAA4901
Lepidoptera|GMPJA10135-21|BIOUG66563-D09|BOLD:AAA4901
Lepidoptera|GMPJA10136-21|BIOUG66563-D10|BOLD:AAA4901
Crambidae|GMPJA7166-21|BIOUG66629-D05|BOLD:AAA4901
Crambidae|GMPJA7164-21|BIOUG66629-D03|BOLD:AAA4901
Crambidae|GMPJA7155-21|BIOUG66629-C06|BOLD:AAA4901
Lepidoptera|GMPJA8867-21|BIOUG63985-E02|BOLD:AAA4901
Lepidoptera|GMPJA8866-21|BIOUG63985-E01|BOLD:AAA4901
Crambidae|GMPJA7156-21|BIOUG66629-C07|BOLD:AAA4901
Crambidae|GMPJA7126-21|BIOUG66629-A01|BOLD:AAA4901
Scopula emissaria|GMPJA10131-21|BIOUG66563-D05|BOLD:ACW2214
Scopula emissaria|GMPJA10132-21|BIOUG66563-D06|BOLD:ACW2214
Scopula emissaria|GMPJA4852-21|BIOUG63984-A07|BOLD:ACW2214
Trichoplusia ni|GMPJA030-21|BIOUG58255-C06|BOLD:AAC3410
Thysanoplusia|GMPJA2938-21|BIOUG58255-H04|BOLD:ACB6622
Plusiinae|GMPJA2941-21|BIOUG58255-H07|BOLD:AAM5409
Plusiinae|GMPJA8856-21|BIOUG63985-D03|BOLD:AAM5409
Chrysodeixis eriosoma|GMPJA10083-21|BIOUG63985-A01|BOLD:AAB3384
Chrysodeixis eriosoma|GMPJA8850-21|BIOUG63985-C09|BOLD:AAB3384
Lepidoptera|GMPJA8849-21|BIOUG63985-C08|BOLD:AAD6357
Eretmocera impactella|GMPJA3831-21|BIOUG65695-C07|BOLD:ACF7356
Herminiinae|GMPJA10089-21|BIOUG63985-A07|BOLD:ABY4999
Erebidae|GMPJA4907-21|BIOUG63984-F02|BOLD:AAB7802
Erebidae|GMPJA7127-21|BIOUG66629-A02|BOLD:ACW8183
Condica illecta|GMPJA002-21|BIOUG58255-A02|BOLD:AAB2411
Mythimna|GMPJA2926-21|BIOUG58255-G04|BOLD:AAB5610
Mythimna|GMPJA013-21|BIOUG58255-B01|BOLD:AAB5610
Mythimna|GMPJA2929-21|BIOUG58255-G07|BOLD:AAB5610
Mythimna|GMPJA2928-21|BIOUG58255-G06|BOLD:AAB5610
Mythimna|GMPJA2920-21|BIOUG58255-F10|BOLD:AAB5610
Mythimna|GMPJA028-21|BIOUG58255-C04|BOLD:AAB5610
Mythimna|GMPJA2932-21|BIOUG58255-G10|BOLD:AAB5610
Mythimna|GMPJA2927-21|BIOUG58255-G05|BOLD:AAB5610
Mythimna|GMPJA2919-21|BIOUG58255-F09|BOLD:AAB5610
Mythimna|GMPJA2933-21|BIOUG58255-G11|BOLD:AAB5610
Mythimna|GMPJA2924-21|BIOUG58255-G02|BOLD:AAB5610
Mythimna|GMPJA2917-21|BIOUG58255-F07|BOLD:AAB5610
Mythimna|GMPJA2916-21|BIOUG58255-F06|BOLD:AAB5610
Noctuinae|GMPJA2936-21|BIOUG58255-H02|BOLD:ACE6246
Noctuinae|GMPJA2939-21|BIOUG58255-H05|BOLD:ACE6246
Noctuinae|GMPJA4870-21|BIOUG63984-C01|BOLD:ACE6246
Eurema|GMPJA8852-21|BIOUG63985-C11|BOLD:AAA6082
Lepidoptera|GMPJA6299-21|BIOUG66620-C05|BOLD:AEJ1178
Cosmopteriginae|GMPJA310-21|BIOUG65658-F11|BOLD:ABX2833
Labdia stibogramma|GMPJA4968-21|BIOUG66606-C04|BOLD:AEY9803
Labdia stibogramma|GMPJA6298-21|BIOUG66620-C04|BOLD:AEY9803
Labdia stibogramma|GMPJA4972-21|BIOUG66606-C08|BOLD:AEY9803
Labdia stibogramma|GMPJA4967-21|BIOUG66606-C03|BOLD:AEY9803
Labdia stibogramma|GMPJA4965-21|BIOUG66606-C01|BOLD:AEY9803
Rivula|GMPJA7151-21|BIOUG66629-C02|BOLD:AEJ6869
Rivula|GMPJA6297-21|BIOUG66620-C03|BOLD:AEJ6869

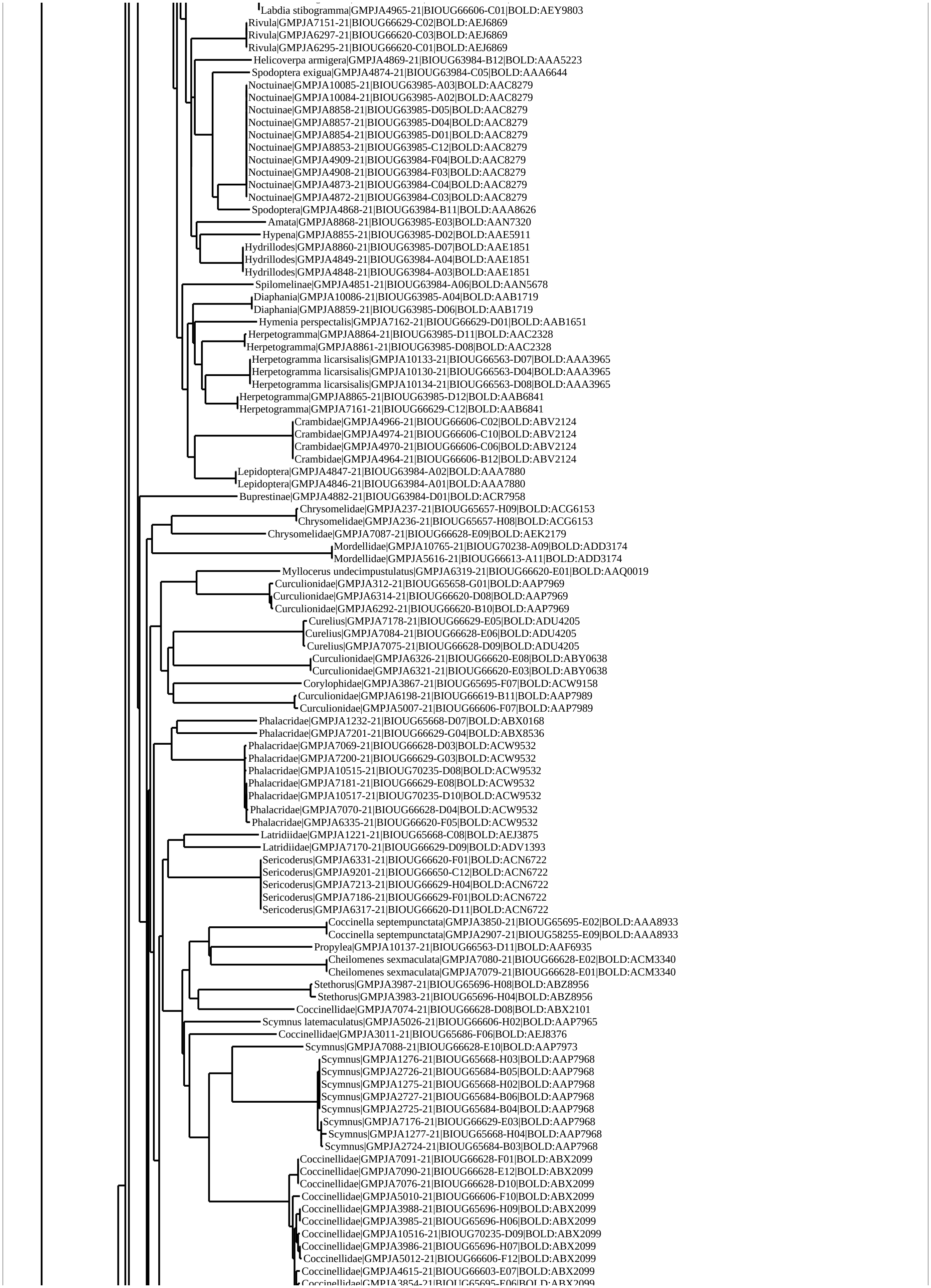
Labdia stibogramma|GMPJA4965-21|BIOUG66606-C01|BOLD:AEY9803
Rivula|GMPJA7151-21|BIOUG66629-C02|BOLD:AEJ6869
Rivula|GMPJA6297-21|BIOUG66620-C03|BOLD:AEJ6869
Rivula|GMPJA6295-21|BIOUG66620-C01|BOLD:AEJ6869
Helicoverpa armigera|GMPJA4869-21|BIOUG63984-B12|BOLD:AAA5223
Spodoptera exigua|GMPJA4874-21|BIOUG63984-C05|BOLD:AAA6644
Noctuinae|GMPJA10085-21|BIOUG63985-A03|BOLD:AAC8279
Noctuinae|GMPJA10084-21|BIOUG63985-A02|BOLD:AAC8279
Noctuinae|GMPJA8858-21|BIOUG63985-D05|BOLD:AAC8279
Noctuinae|GMPJA8857-21|BIOUG63985-D04|BOLD:AAC8279
Noctuinae|GMPJA8854-21|BIOUG63985-D01|BOLD:AAC8279
Noctuinae|GMPJA8853-21|BIOUG63985-C12|BOLD:AAC8279
Noctuinae|GMPJA4909-21|BIOUG63984-F04|BOLD:AAC8279
Noctuinae|GMPJA4908-21|BIOUG63984-F03|BOLD:AAC8279
Noctuinae|GMPJA4873-21|BIOUG63984-C04|BOLD:AAC8279
Noctuinae|GMPJA4872-21|BIOUG63984-C03|BOLD:AAC8279
Spodoptera|GMPJA4868-21|BIOUG63984-B11|BOLD:AAA8626
Amata|GMPJA8868-21|BIOUG63985-E03|BOLD:AAN7320
Hypena|GMPJA8855-21|BIOUG63985-D02|BOLD:AAE5911
Hydrillodes|GMPJA8860-21|BIOUG63985-D07|BOLD:AAE1851
Hydrillodes|GMPJA4849-21|BIOUG63984-A04|BOLD:AAE1851
Hydrillodes|GMPJA4848-21|BIOUG63984-A03|BOLD:AAE1851
Spilomelinae|GMPJA4851-21|BIOUG63984-A06|BOLD:AAN5678
Diaphania|GMPJA10086-21|BIOUG63985-A04|BOLD:AAB1719
Diaphania|GMPJA8859-21|BIOUG63985-D06|BOLD:AAB1719
Hymenia perspectalis|GMPJA7162-21|BIOUG66629-D01|BOLD:AAB1651
Herpetogramma|GMPJA8864-21|BIOUG63985-D11|BOLD:AAC2328
Herpetogramma|GMPJA8861-21|BIOUG63985-D08|BOLD:AAC2328
Herpetogramma licarsisalis|GMPJA10133-21|BIOUG66563-D07|BOLD:AAA3965
Herpetogramma licarsisalis|GMPJA10130-21|BIOUG66563-D04|BOLD:AAA3965
Herpetogramma licarsisalis|GMPJA10134-21|BIOUG66563-D08|BOLD:AAA3965
Herpetogramma|GMPJA8865-21|BIOUG63985-D12|BOLD:AAB6841
Herpetogramma|GMPJA7161-21|BIOUG66629-C12|BOLD:AAB6841
Crambidae|GMPJA4966-21|BIOUG66606-C02|BOLD:ABV2124
Crambidae|GMPJA4974-21|BIOUG66606-C10|BOLD:ABV2124
Crambidae|GMPJA4970-21|BIOUG66606-C06|BOLD:ABV2124
Crambidae|GMPJA4964-21|BIOUG66606-B12|BOLD:ABV2124
Lepidoptera|GMPJA4847-21|BIOUG63984-A02|BOLD:AAA7880
Lepidoptera|GMPJA4846-21|BIOUG63984-A01|BOLD:AAA7880
Buprestinae|GMPJA4882-21|BIOUG63984-D01|BOLD:ACR7958
Chrysomelidae|GMPJA237-21|BIOUG65657-H09|BOLD:ACG6153
Chrysomelidae|GMPJA236-21|BIOUG65657-H08|BOLD:ACG6153
Chrysomelidae|GMPJA7087-21|BIOUG66628-E09|BOLD:AEK2179
Mordellidae|GMPJA10765-21|BIOUG70238-A09|BOLD:ADD3174
Mordellidae|GMPJA5616-21|BIOUG66613-A11|BOLD:ADD3174
Myllocerus undecimpustulatus|GMPJA6319-21|BIOUG66620-E01|BOLD:AAQ0019
Curculionidae|GMPJA312-21|BIOUG65658-G01|BOLD:AAP7969
Curculionidae|GMPJA6314-21|BIOUG66620-D08|BOLD:AAP7969
Curculionidae|GMPJA6292-21|BIOUG66620-B10|BOLD:AAP7969
Curelius|GMPJA7178-21|BIOUG66629-E05|BOLD:ADU4205
Curelius|GMPJA7084-21|BIOUG66628-E06|BOLD:ADU4205
Curelius|GMPJA7075-21|BIOUG66628-D09|BOLD:ADU4205
Curculionidae|GMPJA6326-21|BIOUG66620-E08|BOLD:ABY0638
Curculionidae|GMPJA6321-21|BIOUG66620-E03|BOLD:ABY0638
Corylophidae|GMPJA3867-21|BIOUG65695-F07|BOLD:ACW9158
Curculionidae|GMPJA6198-21|BIOUG66619-B11|BOLD:AAP7989
Curculionidae|GMPJA5007-21|BIOUG66606-F07|BOLD:AAP7989
Phalacridae|GMPJA1232-21|BIOUG65668-D07|BOLD:ABX0168
Phalacridae|GMPJA7201-21|BIOUG66629-G04|BOLD:ABX8536
Phalacridae|GMPJA7069-21|BIOUG66628-D03|BOLD:ACW9532
Phalacridae|GMPJA7200-21|BIOUG66629-G03|BOLD:ACW9532
Phalacridae|GMPJA10515-21|BIOUG70235-D08|BOLD:ACW9532
Phalacridae|GMPJA7181-21|BIOUG66629-E08|BOLD:ACW9532
Phalacridae|GMPJA10517-21|BIOUG70235-D10|BOLD:ACW9532
Phalacridae|GMPJA7070-21|BIOUG66628-D04|BOLD:ACW9532
Phalacridae|GMPJA6335-21|BIOUG66620-F05|BOLD:ACW9532
Latridiidae|GMPJA1221-21|BIOUG65668-C08|BOLD:AEJ3875
Latridiidae|GMPJA7170-21|BIOUG66629-D09|BOLD:ADV1393
Sericoderus|GMPJA6331-21|BIOUG66620-F01|BOLD:ACN6722
Sericoderus|GMPJA9201-21|BIOUG66650-C12|BOLD:ACN6722
Sericoderus|GMPJA7213-21|BIOUG66629-H04|BOLD:ACN6722
Sericoderus|GMPJA7186-21|BIOUG66629-F01|BOLD:ACN6722
Sericoderus|GMPJA6317-21|BIOUG66620-D11|BOLD:ACN6722
Coccinella septempunctata|GMPJA3850-21|BIOUG65695-E02|BOLD:AAA8933
Coccinella septempunctata|GMPJA2907-21|BIOUG58255-E09|BOLD:AAA8933
Propylea|GMPJA10137-21|BIOUG66563-D11|BOLD:AAF6935
Cheilomenes sexmaculata|GMPJA7080-21|BIOUG66628-E02|BOLD:ACM3340
Cheilomenes sexmaculata|GMPJA7079-21|BIOUG66628-E01|BOLD:ACM3340
Stethorus|GMPJA3987-21|BIOUG65696-H08|BOLD:ABZ8956
Stethorus|GMPJA3983-21|BIOUG65696-H04|BOLD:ABZ8956
Coccinellidae|GMPJA7074-21|BIOUG66628-D08|BOLD:ABX2101
Scymnus latemaculatus|GMPJA5026-21|BIOUG66606-H02|BOLD:AAP7965
Coccinellidae|GMPJA3011-21|BIOUG65686-F06|BOLD:AEJ8376
Scymnus|GMPJA7088-21|BIOUG66628-E10|BOLD:AAP7973
Scymnus|GMPJA1276-21|BIOUG65668-H03|BOLD:AAP7968
Scymnus|GMPJA2726-21|BIOUG65684-B05|BOLD:AAP7968
Scymnus|GMPJA1275-21|BIOUG65668-H02|BOLD:AAP7968
Scymnus|GMPJA2727-21|BIOUG65684-B06|BOLD:AAP7968
Scymnus|GMPJA2725-21|BIOUG65684-B04|BOLD:AAP7968
Scymnus|GMPJA7176-21|BIOUG66629-E03|BOLD:AAP7968
Scymnus|GMPJA1277-21|BIOUG65668-H04|BOLD:AAP7968
Scymnus|GMPJA2724-21|BIOUG65684-B03|BOLD:AAP7968
Coccinellidae|GMPJA7091-21|BIOUG66628-F01|BOLD:ABX2099
Coccinellidae|GMPJA7090-21|BIOUG66628-E12|BOLD:ABX2099
Coccinellidae|GMPJA7076-21|BIOUG66628-D10|BOLD:ABX2099
Coccinellidae|GMPJA5010-21|BIOUG66606-F10|BOLD:ABX2099
Coccinellidae|GMPJA3988-21|BIOUG65696-H09|BOLD:ABX2099
Coccinellidae|GMPJA3985-21|BIOUG65696-H06|BOLD:ABX2099
Coccinellidae|GMPJA10516-21|BIOUG70235-D09|BOLD:ABX2099
Coccinellidae|GMPJA3986-21|BIOUG65696-H07|BOLD:ABX2099
Coccinellidae|GMPJA5012-21|BIOUG66606-F12|BOLD:ABX2099
Coccinellidae|GMPJA4615-21|BIOUG66603-E07|BOLD:ABX2099
Coccinellidae|GMPJA3854-21|BIOUG65695-F06|BOLD:ABX2099

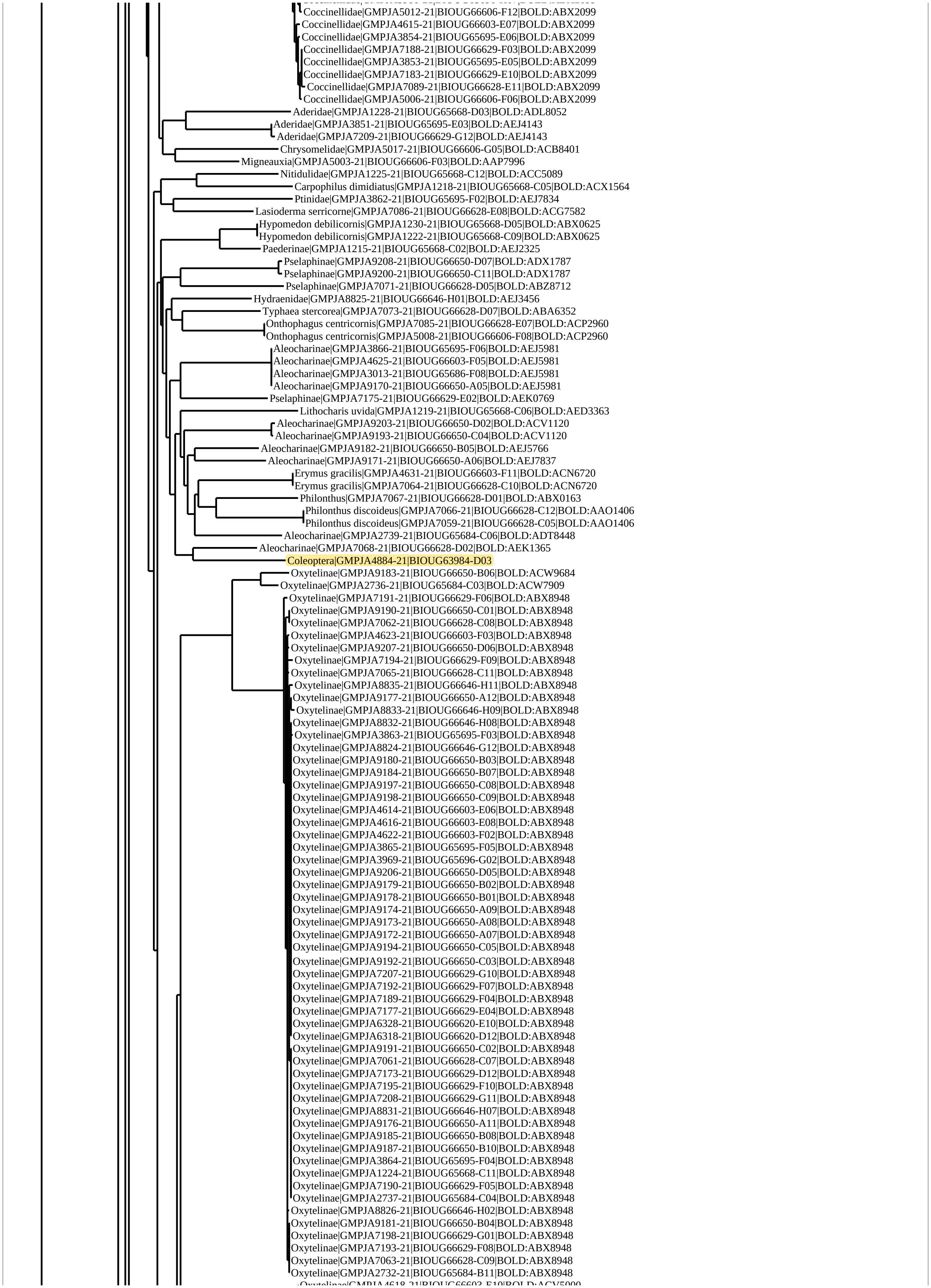
Coccinellidae|GMPJA5012-21|BIOUG66606-F12|BOLD:ABX2099
Coccinellidae|GMPJA4615-21|BIOUG66603-E07|BOLD:ABX2099
Coccinellidae|GMPJA3854-21|BIOUG65695-E06|BOLD:ABX2099
Coccinellidae|GMPJA7188-21|BIOUG66629-F03|BOLD:ABX2099
Coccinellidae|GMPJA3853-21|BIOUG65695-E05|BOLD:ABX2099
Coccinellidae|GMPJA7183-21|BIOUG66629-E10|BOLD:ABX2099
Coccinellidae|GMPJA7089-21|BIOUG66628-E11|BOLD:ABX2099
Coccinellidae|GMPJA5006-21|BIOUG66606-F06|BOLD:ABX2099
Aderidae|GMPJA1228-21|BIOUG65668-D03|BOLD:ADL8052
Aderidae|GMPJA3851-21|BIOUG65695-E03|BOLD:AEJ4143
Aderidae|GMPJA7209-21|BIOUG66629-G12|BOLD:AEJ4143
Chrysomelidae|GMPJA5017-21|BIOUG66606-G05|BOLD:ACB8401
Migneauxia|GMPJA5003-21|BIOUG66606-F03|BOLD:AAP7996
Nitidulidae|GMPJA1225-21|BIOUG65668-C12|BOLD:ACC5089
Carpophilus dimidiatus|GMPJA1218-21|BIOUG65668-C05|BOLD:ACX1564
Ptinidae|GMPJA3862-21|BIOUG65695-F02|BOLD:AEJ7834
Lasioderma serricorne|GMPJA7086-21|BIOUG66628-E08|BOLD:ACG7582
Hypomedon debilicornis|GMPJA1230-21|BIOUG65668-D05|BOLD:ABX0625
Hypomedon debilicornis|GMPJA1222-21|BIOUG65668-C09|BOLD:ABX0625
Paederinae|GMPJA1215-21|BIOUG65668-C02|BOLD:AEJ2325
Pselaphinae|GMPJA9208-21|BIOUG66650-D07|BOLD:ADX1787
Pselaphinae|GMPJA9200-21|BIOUG66650-C11|BOLD:ADX1787
Pselaphinae|GMPJA7071-21|BIOUG66628-D05|BOLD:ABZ8712
Hydraenidae|GMPJA8825-21|BIOUG66646-H01|BOLD:AEJ3456
Typhaea stercorea|GMPJA7073-21|BIOUG66628-D07|BOLD:ABA6352
Onthophagus centricornis|GMPJA7085-21|BIOUG66628-E07|BOLD:ACP2960
Onthophagus centricornis|GMPJA5008-21|BIOUG66606-F08|BOLD:ACP2960
Aleocharinae|GMPJA3866-21|BIOUG65695-F06|BOLD:AEJ5981
Aleocharinae|GMPJA4625-21|BIOUG66603-F05|BOLD:AEJ5981
Aleocharinae|GMPJA3013-21|BIOUG65686-F08|BOLD:AEJ5981
Aleocharinae|GMPJA9170-21|BIOUG66650-A05|BOLD:AEJ5981
Pselaphinae|GMPJA7175-21|BIOUG66629-E02|BOLD:AEK0769
Lithocharis uvida|GMPJA1219-21|BIOUG65668-C06|BOLD:AED3363
Aleocharinae|GMPJA9203-21|BIOUG66650-D02|BOLD:ACV1120
Aleocharinae|GMPJA9193-21|BIOUG66650-C04|BOLD:ACV1120
Aleocharinae|GMPJA9182-21|BIOUG66650-B05|BOLD:AEJ5766
Aleocharinae|GMPJA9171-21|BIOUG66650-A06|BOLD:AEJ7837
Erymus gracilis|GMPJA4631-21|BIOUG66603-F11|BOLD:ACN6720
Erymus gracilis|GMPJA7064-21|BIOUG66628-C10|BOLD:ACN6720
Philonthus|GMPJA7067-21|BIOUG66628-D01|BOLD:ABX0163
Philonthus discoideus|GMPJA7066-21|BIOUG66628-C12|BOLD:AAO1406
Philonthus discoideus|GMPJA7059-21|BIOUG66628-C05|BOLD:AAO1406
Aleocharinae|GMPJA2739-21|BIOUG65684-C06|BOLD:ADT8448
Aleocharinae|GMPJA7068-21|BIOUG66628-D02|BOLD:AEK1365
Coleoptera|GMPJA4884-21|BIOUG63984-D03
Oxytelinae|GMPJA9183-21|BIOUG66650-B06|BOLD:ACW9684
Oxytelinae|GMPJA2736-21|BIOUG65684-C03|BOLD:ACW7909
Oxytelinae|GMPJA7191-21|BIOUG66629-F06|BOLD:ABX8948
Oxytelinae|GMPJA9190-21|BIOUG66650-C01|BOLD:ABX8948
Oxytelinae|GMPJA7062-21|BIOUG66628-C08|BOLD:ABX8948
Oxytelinae|GMPJA4623-21|BIOUG66603-F03|BOLD:ABX8948
Oxytelinae|GMPJA9207-21|BIOUG66650-D06|BOLD:ABX8948
Oxytelinae|GMPJA7194-21|BIOUG66629-F09|BOLD:ABX8948
Oxytelinae|GMPJA7065-21|BIOUG66628-C11|BOLD:ABX8948
Oxytelinae|GMPJA8835-21|BIOUG66646-H11|BOLD:ABX8948
Oxytelinae|GMPJA9177-21|BIOUG66650-A12|BOLD:ABX8948
Oxytelinae|GMPJA8833-21|BIOUG66646-H09|BOLD:ABX8948
Oxytelinae|GMPJA8832-21|BIOUG66646-H08|BOLD:ABX8948
Oxytelinae|GMPJA3863-21|BIOUG65695-F03|BOLD:ABX8948
Oxytelinae|GMPJA8824-21|BIOUG66646-G12|BOLD:ABX8948
Oxytelinae|GMPJA9180-21|BIOUG66650-B03|BOLD:ABX8948
Oxytelinae|GMPJA9184-21|BIOUG66650-B07|BOLD:ABX8948
Oxytelinae|GMPJA9197-21|BIOUG66650-C08|BOLD:ABX8948
Oxytelinae|GMPJA9198-21|BIOUG66650-C09|BOLD:ABX8948
Oxytelinae|GMPJA4614-21|BIOUG66603-E06|BOLD:ABX8948
Oxytelinae|GMPJA4616-21|BIOUG66603-E08|BOLD:ABX8948
Oxytelinae|GMPJA4622-21|BIOUG66603-F02|BOLD:ABX8948
Oxytelinae|GMPJA3865-21|BIOUG65695-F05|BOLD:ABX8948
Oxytelinae|GMPJA3969-21|BIOUG65696-G02|BOLD:ABX8948
Oxytelinae|GMPJA9206-21|BIOUG66650-D05|BOLD:ABX8948
Oxytelinae|GMPJA9179-21|BIOUG66650-B02|BOLD:ABX8948
Oxytelinae|GMPJA9178-21|BIOUG66650-B01|BOLD:ABX8948
Oxytelinae|GMPJA9174-21|BIOUG66650-A09|BOLD:ABX8948
Oxytelinae|GMPJA9173-21|BIOUG66650-A08|BOLD:ABX8948
Oxytelinae|GMPJA9172-21|BIOUG66650-A07|BOLD:ABX8948
Oxytelinae|GMPJA9194-21|BIOUG66650-C05|BOLD:ABX8948
Oxytelinae|GMPJA9192-21|BIOUG66650-C03|BOLD:ABX8948
Oxytelinae|GMPJA7207-21|BIOUG66629-G10|BOLD:ABX8948
Oxytelinae|GMPJA7192-21|BIOUG66629-F07|BOLD:ABX8948
Oxytelinae|GMPJA7189-21|BIOUG66629-F04|BOLD:ABX8948
Oxytelinae|GMPJA7177-21|BIOUG66629-E04|BOLD:ABX8948
Oxytelinae|GMPJA6328-21|BIOUG66620-E10|BOLD:ABX8948
Oxytelinae|GMPJA6318-21|BIOUG66620-D12|BOLD:ABX8948
Oxytelinae|GMPJA9191-21|BIOUG66650-C02|BOLD:ABX8948
Oxytelinae|GMPJA7061-21|BIOUG66628-C07|BOLD:ABX8948
Oxytelinae|GMPJA7173-21|BIOUG66629-D12|BOLD:ABX8948
Oxytelinae|GMPJA7195-21|BIOUG66629-F10|BOLD:ABX8948
Oxytelinae|GMPJA7208-21|BIOUG66629-G11|BOLD:ABX8948
Oxytelinae|GMPJA8831-21|BIOUG66646-H07|BOLD:ABX8948
Oxytelinae|GMPJA9176-21|BIOUG66650-A11|BOLD:ABX8948
Oxytelinae|GMPJA9185-21|BIOUG66650-B08|BOLD:ABX8948
Oxytelinae|GMPJA9187-21|BIOUG66650-B10|BOLD:ABX8948
Oxytelinae|GMPJA3864-21|BIOUG65695-F04|BOLD:ABX8948
Oxytelinae|GMPJA1224-21|BIOUG65668-C11|BOLD:ABX8948
Oxytelinae|GMPJA7190-21|BIOUG66629-F05|BOLD:ABX8948
Oxytelinae|GMPJA2737-21|BIOUG65684-C04|BOLD:ABX8948
Oxytelinae|GMPJA8826-21|BIOUG66646-H02|BOLD:ABX8948
Oxytelinae|GMPJA9181-21|BIOUG66650-B04|BOLD:ABX8948
Oxytelinae|GMPJA7198-21|BIOUG66629-G01|BOLD:ABX8948
Oxytelinae|GMPJA7193-21|BIOUG66629-F08|BOLD:ABX8948
Oxytelinae|GMPJA7063-21|BIOUG66628-C09|BOLD:ABX8948
Oxytelinae|GMPJA2732-21|BIOUG65684-B11|BOLD:ABX8948

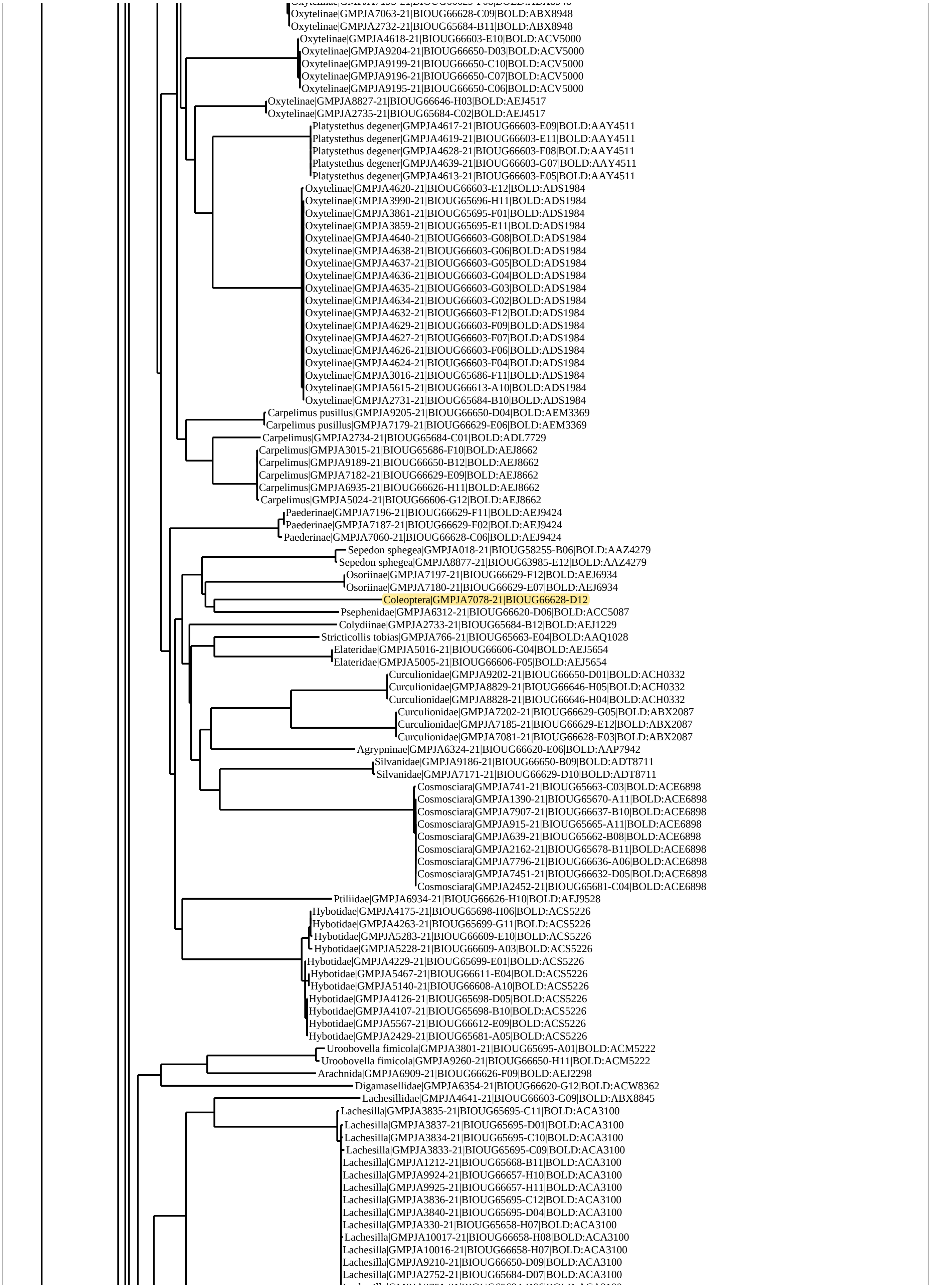
Oxytelinae|GMPJA7063-21|BIOUG66628-C09|BOLD:ABX8948
Oxytelinae|GMPJA2732-21|BIOUG65684-B11|BOLD:ABX8948
Oxytelinae|GMPJA4618-21|BIOUG66603-E10|BOLD:ACV5000
Oxytelinae|GMPJA9204-21|BIOUG66650-D03|BOLD:ACV5000
Oxytelinae|GMPJA9199-21|BIOUG66650-C10|BOLD:ACV5000
Oxytelinae|GMPJA9196-21|BIOUG66650-C07|BOLD:ACV5000
Oxytelinae|GMPJA9195-21|BIOUG66650-C06|BOLD:ACV5000
Oxytelinae|GMPJA8827-21|BIOUG66646-H03|BOLD:AEJ4517
Oxytelinae|GMPJA2735-21|BIOUG65684-C02|BOLD:AEJ4517
Platystethus degener|GMPJA4617-21|BIOUG66603-E09|BOLD:AAY4511
Platystethus degener|GMPJA4619-21|BIOUG66603-E11|BOLD:AAY4511
Platystethus degener|GMPJA4628-21|BIOUG66603-F08|BOLD:AAY4511
Platystethus degener|GMPJA4639-21|BIOUG66603-G07|BOLD:AAY4511
Platystethus degener|GMPJA4613-21|BIOUG66603-E05|BOLD:AAY4511
Oxytelinae|GMPJA4620-21|BIOUG66603-E12|BOLD:ADS1984
Oxytelinae|GMPJA3990-21|BIOUG65696-H11|BOLD:ADS1984
Oxytelinae|GMPJA3861-21|BIOUG65695-F01|BOLD:ADS1984
Oxytelinae|GMPJA3859-21|BIOUG65695-E11|BOLD:ADS1984
Oxytelinae|GMPJA4640-21|BIOUG66603-G08|BOLD:ADS1984
Oxytelinae|GMPJA4638-21|BIOUG66603-G06|BOLD:ADS1984
Oxytelinae|GMPJA4637-21|BIOUG66603-G05|BOLD:ADS1984
Oxytelinae|GMPJA4636-21|BIOUG66603-G04|BOLD:ADS1984
Oxytelinae|GMPJA4635-21|BIOUG66603-G03|BOLD:ADS1984
Oxytelinae|GMPJA4634-21|BIOUG66603-G02|BOLD:ADS1984
Oxytelinae|GMPJA4632-21|BIOUG66603-F12|BOLD:ADS1984
Oxytelinae|GMPJA4629-21|BIOUG66603-F09|BOLD:ADS1984
Oxytelinae|GMPJA4627-21|BIOUG66603-F07|BOLD:ADS1984
Oxytelinae|GMPJA4626-21|BIOUG66603-F06|BOLD:ADS1984
Oxytelinae|GMPJA4624-21|BIOUG66603-F04|BOLD:ADS1984
Oxytelinae|GMPJA3016-21|BIOUG65686-F11|BOLD:ADS1984
Oxytelinae|GMPJA5615-21|BIOUG66613-A10|BOLD:ADS1984
Oxytelinae|GMPJA2731-21|BIOUG65684-B10|BOLD:ADS1984
Carpelimus pusillus|GMPJA9205-21|BIOUG66650-D04|BOLD:AEM3369
Carpelimus pusillus|GMPJA7179-21|BIOUG66629-E06|BOLD:AEM3369
Carpelimus|GMPJA2734-21|BIOUG65684-C01|BOLD:ADL7729
Carpelimus|GMPJA3015-21|BIOUG65686-F10|BOLD:AEJ8662
Carpelimus|GMPJA9189-21|BIOUG66650-B12|BOLD:AEJ8662
Carpelimus|GMPJA7182-21|BIOUG66629-E09|BOLD:AEJ8662
Carpelimus|GMPJA6935-21|BIOUG66626-H11|BOLD:AEJ8662
Carpelimus|GMPJA5024-21|BIOUG66606-G12|BOLD:AEJ8662
Paederinae|GMPJA7196-21|BIOUG66629-F11|BOLD:AEJ9424
Paederinae|GMPJA7187-21|BIOUG66629-F02|BOLD:AEJ9424
Paederinae|GMPJA7060-21|BIOUG66628-C06|BOLD:AEJ9424
Sepedon sphegea|GMPJA018-21|BIOUG58255-B06|BOLD:AAZ4279
Sepedon sphegea|GMPJA8877-21|BIOUG63985-E12|BOLD:AAZ4279
Osoriinae|GMPJA7197-21|BIOUG66629-F12|BOLD:AEJ6934
Osoriinae|GMPJA7180-21|BIOUG66629-E07|BOLD:AEJ6934
Coleoptera|GMPJA7078-21|BIOUG66628-D12
Psephenidae|GMPJA6312-21|BIOUG66620-D06|BOLD:ACC5087
Colydiinae|GMPJA2733-21|BIOUG65684-B12|BOLD:AEJ1229
Stricticollis tobias|GMPJA766-21|BIOUG65663-E04|BOLD:AAQ1028
Elateridae|GMPJA5016-21|BIOUG66606-G04|BOLD:AEJ5654
Elateridae|GMPJA5005-21|BIOUG66606-F05|BOLD:AEJ5654
Curculionidae|GMPJA9202-21|BIOUG66650-D01|BOLD:ACH0332
Curculionidae|GMPJA8829-21|BIOUG66646-H05|BOLD:ACH0332
Curculionidae|GMPJA8828-21|BIOUG66646-H04|BOLD:ACH0332
Curculionidae|GMPJA7202-21|BIOUG66629-G05|BOLD:ABX2087
Curculionidae|GMPJA7185-21|BIOUG66629-E12|BOLD:ABX2087
Curculionidae|GMPJA7081-21|BIOUG66628-E03|BOLD:ABX2087
Agrypninae|GMPJA6324-21|BIOUG66620-E06|BOLD:AAP7942
Silvanidae|GMPJA9186-21|BIOUG66650-B09|BOLD:ADT8711
Silvanidae|GMPJA7171-21|BIOUG66629-D10|BOLD:ADT8711
Cosmosciara|GMPJA741-21|BIOUG65663-C03|BOLD:ACE6898
Cosmosciara|GMPJA1390-21|BIOUG65670-A11|BOLD:ACE6898
Cosmosciara|GMPJA7907-21|BIOUG66637-B10|BOLD:ACE6898
Cosmosciara|GMPJA915-21|BIOUG65665-A11|BOLD:ACE6898
Cosmosciara|GMPJA639-21|BIOUG65662-B08|BOLD:ACE6898
Cosmosciara|GMPJA2162-21|BIOUG65678-B11|BOLD:ACE6898
Cosmosciara|GMPJA7796-21|BIOUG66636-A06|BOLD:ACE6898
Cosmosciara|GMPJA7451-21|BIOUG66632-D05|BOLD:ACE6898
Cosmosciara|GMPJA2452-21|BIOUG65681-C04|BOLD:ACE6898
Ptiliidae|GMPJA6934-21|BIOUG66626-H10|BOLD:AEJ9528
Hybotidae|GMPJA4175-21|BIOUG65698-H06|BOLD:ACS5226
Hybotidae|GMPJA4263-21|BIOUG65699-G11|BOLD:ACS5226
Hybotidae|GMPJA5283-21|BIOUG66609-E10|BOLD:ACS5226
Hybotidae|GMPJA5228-21|BIOUG66609-A03|BOLD:ACS5226
Hybotidae|GMPJA4229-21|BIOUG65699-E01|BOLD:ACS5226
Hybotidae|GMPJA5467-21|BIOUG66611-E04|BOLD:ACS5226
Hybotidae|GMPJA5140-21|BIOUG66608-A10|BOLD:ACS5226
Hybotidae|GMPJA4126-21|BIOUG65698-D05|BOLD:ACS5226
Hybotidae|GMPJA4107-21|BIOUG65698-B10|BOLD:ACS5226
Hybotidae|GMPJA5567-21|BIOUG66612-E09|BOLD:ACS5226
Hybotidae|GMPJA2429-21|BIOUG65681-A05|BOLD:ACS5226
Uroobovella fimicola|GMPJA3801-21|BIOUG65695-A01|BOLD:ACM5222
Uroobovella fimicola|GMPJA9260-21|BIOUG66650-H11|BOLD:ACM5222
Arachnida|GMPJA6909-21|BIOUG66626-F09|BOLD:AEJ2298
Digamasellidae|GMPJA6354-21|BIOUG66620-G12|BOLD:ACW8362
Lachesillidae|GMPJA4641-21|BIOUG66603-G09|BOLD:ABX8845
Lachesilla|GMPJA3835-21|BIOUG65695-C11|BOLD:ACA3100
Lachesilla|GMPJA3837-21|BIOUG65695-D01|BOLD:ACA3100
Lachesilla|GMPJA3834-21|BIOUG65695-C10|BOLD:ACA3100
Lachesilla|GMPJA3833-21|BIOUG65695-C09|BOLD:ACA3100
Lachesilla|GMPJA1212-21|BIOUG65668-B11|BOLD:ACA3100
Lachesilla|GMPJA9924-21|BIOUG66657-H10|BOLD:ACA3100
Lachesilla|GMPJA9925-21|BIOUG66657-H11|BOLD:ACA3100
Lachesilla|GMPJA3836-21|BIOUG65695-C12|BOLD:ACA3100
Lachesilla|GMPJA3840-21|BIOUG65695-D04|BOLD:ACA3100
Lachesilla|GMPJA330-21|BIOUG65658-H07|BOLD:ACA3100
Lachesilla|GMPJA10017-21|BIOUG66658-H08|BOLD:ACA3100
Lachesilla|GMPJA10016-21|BIOUG66658-H07|BOLD:ACA3100
Lachesilla|GMPJA9210-21|BIOUG66650-D09|BOLD:ACA3100
Lachesilla|GMPJA2752-21|BIOUG65684-D07|BOLD:ACA3100

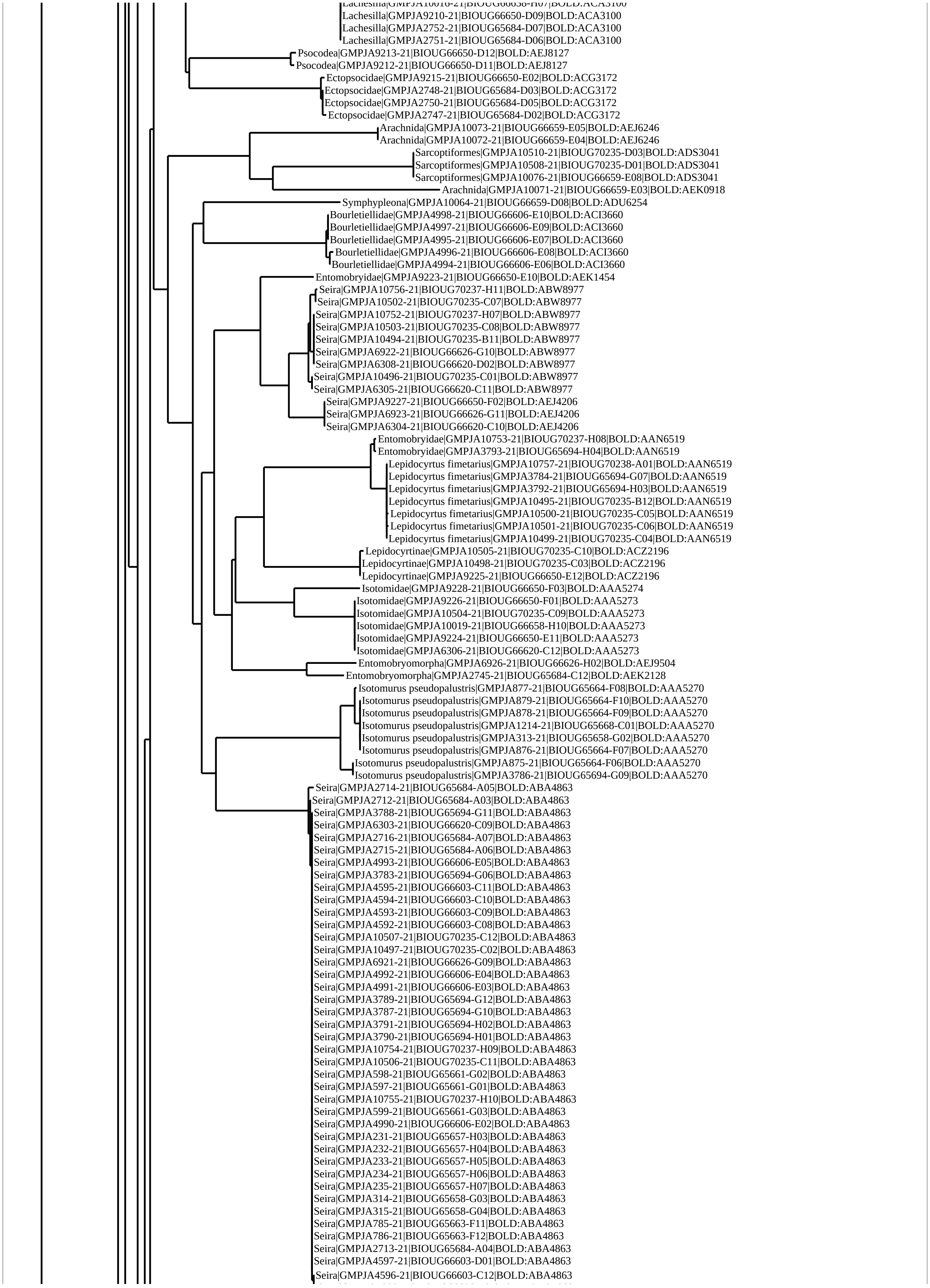
Lachesilla|GMPJA10018-21|BIOUG66658-H07|BOLD:ACA3100
Lachesilla|GMPJA9210-21|BIOUG66650-D09|BOLD:ACA3100
Lachesilla|GMPJA2752-21|BIOUG65684-D07|BOLD:ACA3100
Lachesilla|GMPJA2751-21|BIOUG65684-D06|BOLD:ACA3100
Psocodea|GMPJA9213-21|BIOUG66650-D12|BOLD:AEJ8127
Psocodea|GMPJA9212-21|BIOUG66650-D11|BOLD:AEJ8127
Ectopsocidae|GMPJA9215-21|BIOUG66650-E02|BOLD:ACG3172
Ectopsocidae|GMPJA2748-21|BIOUG65684-D03|BOLD:ACG3172
Ectopsocidae|GMPJA2750-21|BIOUG65684-D05|BOLD:ACG3172
Ectopsocidae|GMPJA2747-21|BIOUG65684-D02|BOLD:ACG3172
Arachnida|GMPJA10073-21|BIOUG66659-E05|BOLD:AEJ6246
Arachnida|GMPJA10072-21|BIOUG66659-E04|BOLD:AEJ6246
Sarcoptiformes|GMPJA10510-21|BIOUG70235-D03|BOLD:ADS3041
Sarcoptiformes|GMPJA10508-21|BIOUG70235-D01|BOLD:ADS3041
Sarcoptiformes|GMPJA10076-21|BIOUG66659-E08|BOLD:ADS3041
Arachnida|GMPJA10071-21|BIOUG66659-E03|BOLD:AEK0918
Symphypleona|GMPJA10064-21|BIOUG66659-D08|BOLD:ADU6254
Bourletiellidae|GMPJA4998-21|BIOUG66606-E10|BOLD:ACI3660
Bourletiellidae|GMPJA4997-21|BIOUG66606-E09|BOLD:ACI3660
Bourletiellidae|GMPJA4995-21|BIOUG66606-E07|BOLD:ACI3660
Bourletiellidae|GMPJA4996-21|BIOUG66606-E08|BOLD:ACI3660
Bourletiellidae|GMPJA4994-21|BIOUG66606-E06|BOLD:ACI3660
Entomobryidae|GMPJA9223-21|BIOUG66650-E10|BOLD:AEK1454
Seira|GMPJA10756-21|BIOUG70237-H11|BOLD:ABW8977
Seira|GMPJA10502-21|BIOUG70235-C07|BOLD:ABW8977
Seira|GMPJA10752-21|BIOUG70237-H07|BOLD:ABW8977
Seira|GMPJA10503-21|BIOUG70235-C08|BOLD:ABW8977
Seira|GMPJA10494-21|BIOUG70235-B11|BOLD:ABW8977
Seira|GMPJA6922-21|BIOUG66626-G10|BOLD:ABW8977
Seira|GMPJA6308-21|BIOUG66620-D02|BOLD:ABW8977
Seira|GMPJA10496-21|BIOUG70235-C01|BOLD:ABW8977
Seira|GMPJA6305-21|BIOUG66620-C11|BOLD:ABW8977
Seira|GMPJA9227-21|BIOUG66650-F02|BOLD:AEJ4206
Seira|GMPJA6923-21|BIOUG66626-G11|BOLD:AEJ4206
Seira|GMPJA6304-21|BIOUG66620-C10|BOLD:AEJ4206
Entomobryidae|GMPJA10753-21|BIOUG70237-H08|BOLD:AAN6519
Entomobryidae|GMPJA3793-21|BIOUG65694-H04|BOLD:AAN6519
Lepidocyrtus fimetarius|GMPJA10757-21|BIOUG70238-A01|BOLD:AAN6519
Lepidocyrtus fimetarius|GMPJA3784-21|BIOUG65694-G07|BOLD:AAN6519
Lepidocyrtus fimetarius|GMPJA3792-21|BIOUG65694-H03|BOLD:AAN6519
Lepidocyrtus fimetarius|GMPJA10495-21|BIOUG70235-B12|BOLD:AAN6519
Lepidocyrtus fimetarius|GMPJA10500-21|BIOUG70235-C05|BOLD:AAN6519
Lepidocyrtus fimetarius|GMPJA10501-21|BIOUG70235-C06|BOLD:AAN6519
Lepidocyrtus fimetarius|GMPJA10499-21|BIOUG70235-C04|BOLD:AAN6519
Lepidocyrtinae|GMPJA10505-21|BIOUG70235-C10|BOLD:ACZ2196
Lepidocyrtinae|GMPJA10498-21|BIOUG70235-C03|BOLD:ACZ2196
Lepidocyrtinae|GMPJA9225-21|BIOUG66650-E12|BOLD:ACZ2196
Isotomidae|GMPJA9228-21|BIOUG66650-F03|BOLD:AAA5274
Isotomidae|GMPJA9226-21|BIOUG66650-F01|BOLD:AAA5273
Isotomidae|GMPJA10504-21|BIOUG70235-C09|BOLD:AAA5273
Isotomidae|GMPJA10019-21|BIOUG66658-H10|BOLD:AAA5273
Isotomidae|GMPJA9224-21|BIOUG66650-E11|BOLD:AAA5273
Isotomidae|GMPJA6306-21|BIOUG66620-C12|BOLD:AAA5273
Entomobryomorpha|GMPJA6926-21|BIOUG66626-H02|BOLD:AEJ9504
Entomobryomorpha|GMPJA2745-21|BIOUG65684-C12|BOLD:AEK2128
Isotomurus pseudopalustris|GMPJA877-21|BIOUG65664-F08|BOLD:AAA5270
Isotomurus pseudopalustris|GMPJA879-21|BIOUG65664-F10|BOLD:AAA5270
Isotomurus pseudopalustris|GMPJA878-21|BIOUG65664-F09|BOLD:AAA5270
Isotomurus pseudopalustris|GMPJA1214-21|BIOUG65668-C01|BOLD:AAA5270
Isotomurus pseudopalustris|GMPJA313-21|BIOUG65658-G02|BOLD:AAA5270
Isotomurus pseudopalustris|GMPJA876-21|BIOUG65664-F07|BOLD:AAA5270
Isotomurus pseudopalustris|GMPJA875-21|BIOUG65664-F06|BOLD:AAA5270
Isotomurus pseudopalustris|GMPJA3786-21|BIOUG65694-G09|BOLD:AAA5270
Seira|GMPJA2714-21|BIOUG65684-A05|BOLD:ABA4863
Seira|GMPJA2712-21|BIOUG65684-A03|BOLD:ABA4863
Seira|GMPJA3788-21|BIOUG65694-G11|BOLD:ABA4863
Seira|GMPJA6303-21|BIOUG66620-C09|BOLD:ABA4863
Seira|GMPJA2716-21|BIOUG65684-A07|BOLD:ABA4863
Seira|GMPJA2715-21|BIOUG65684-A06|BOLD:ABA4863
Seira|GMPJA4993-21|BIOUG66606-E05|BOLD:ABA4863
Seira|GMPJA3783-21|BIOUG65694-G06|BOLD:ABA4863
Seira|GMPJA4595-21|BIOUG66603-C11|BOLD:ABA4863
Seira|GMPJA4594-21|BIOUG66603-C10|BOLD:ABA4863
Seira|GMPJA4593-21|BIOUG66603-C09|BOLD:ABA4863
Seira|GMPJA4592-21|BIOUG66603-C08|BOLD:ABA4863
Seira|GMPJA10507-21|BIOUG70235-C12|BOLD:ABA4863
Seira|GMPJA10497-21|BIOUG70235-C02|BOLD:ABA4863
Seira|GMPJA6921-21|BIOUG66626-G09|BOLD:ABA4863
Seira|GMPJA4992-21|BIOUG66606-E04|BOLD:ABA4863
Seira|GMPJA4991-21|BIOUG66606-E03|BOLD:ABA4863
Seira|GMPJA3789-21|BIOUG65694-G12|BOLD:ABA4863
Seira|GMPJA3787-21|BIOUG65694-G10|BOLD:ABA4863
Seira|GMPJA3791-21|BIOUG65694-H02|BOLD:ABA4863
Seira|GMPJA3790-21|BIOUG65694-H01|BOLD:ABA4863
Seira|GMPJA10754-21|BIOUG70237-H09|BOLD:ABA4863
Seira|GMPJA10506-21|BIOUG70235-C11|BOLD:ABA4863
Seira|GMPJA598-21|BIOUG65661-G02|BOLD:ABA4863
Seira|GMPJA597-21|BIOUG65661-G01|BOLD:ABA4863
Seira|GMPJA10755-21|BIOUG70237-H10|BOLD:ABA4863
Seira|GMPJA599-21|BIOUG65661-G03|BOLD:ABA4863
Seira|GMPJA4990-21|BIOUG66606-E02|BOLD:ABA4863
Seira|GMPJA231-21|BIOUG65657-H03|BOLD:ABA4863
Seira|GMPJA232-21|BIOUG65657-H04|BOLD:ABA4863
Seira|GMPJA233-21|BIOUG65657-H05|BOLD:ABA4863
Seira|GMPJA234-21|BIOUG65657-H06|BOLD:ABA4863
Seira|GMPJA235-21|BIOUG65657-H07|BOLD:ABA4863
Seira|GMPJA314-21|BIOUG65658-G03|BOLD:ABA4863
Seira|GMPJA315-21|BIOUG65658-G04|BOLD:ABA4863
Seira|GMPJA785-21|BIOUG65663-F11|BOLD:ABA4863
Seira|GMPJA786-21|BIOUG65663-F12|BOLD:ABA4863
Seira|GMPJA2713-21|BIOUG65684-A04|BOLD:ABA4863
Seira|GMPJA4597-21|BIOUG66603-D01|BOLD:ABA4863
Seira|GMPJA4596-21|BIOUG66603-C12|BOLD:ABA4863

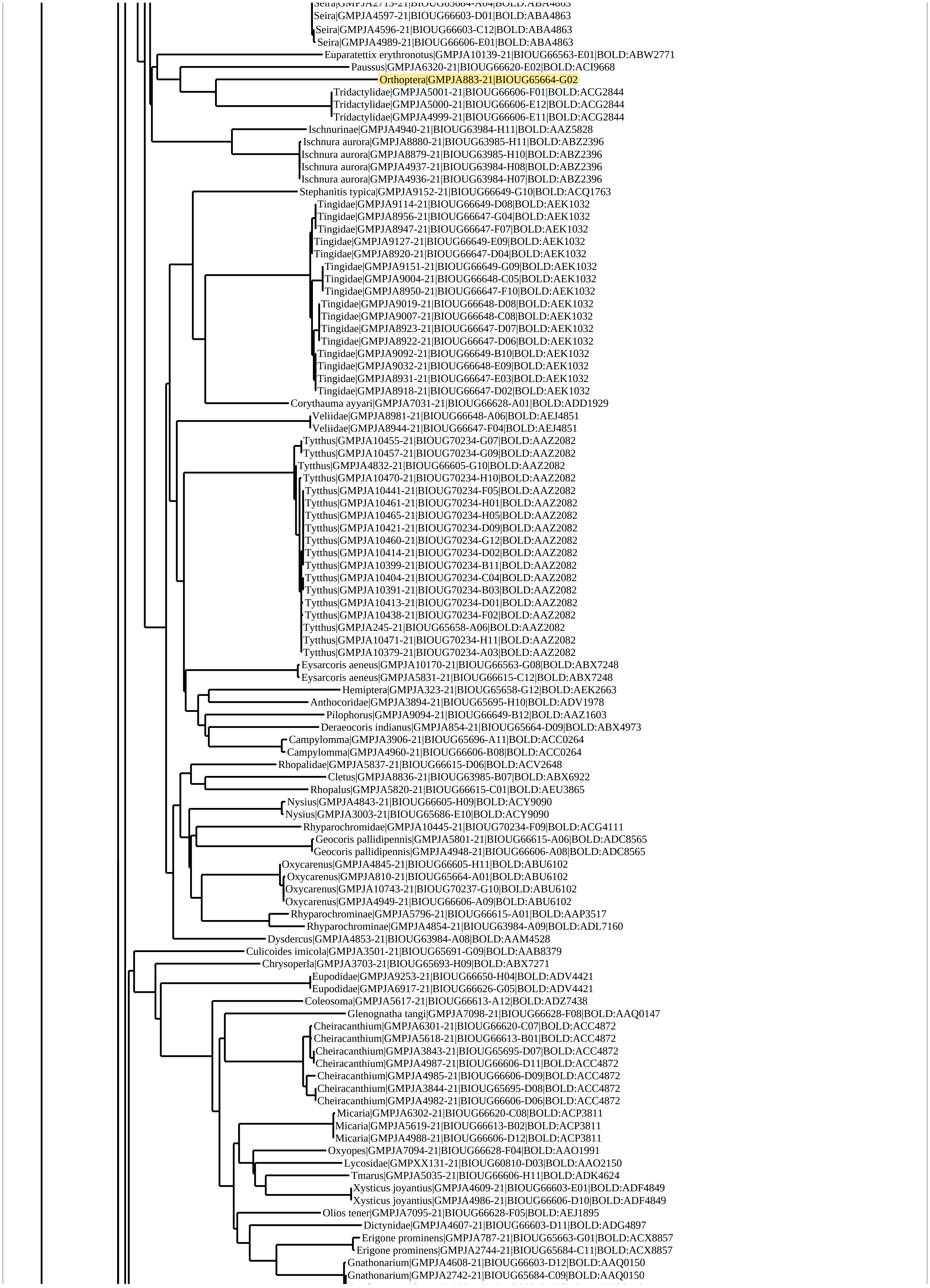
Seira|GMPJA4597-21|BIOUG66603-D01|BOLD:ABA4863
Seira|GMPJA4596-21|BIOUG66603-C12|BOLD:ABA4863
Seira|GMPJA4989-21|BIOUG66606-E01|BOLD:ABA4863
Euparatettix erythronotus|GMPJA10139-21|BIOUG66563-E01|BOLD:ABW2771
Paussus|GMPJA6320-21|BIOUG66620-E02|BOLD:ACI9668
Orthoptera|GMPJA883-21|BIOUG65664-G02
Tridactylidae|GMPJA5001-21|BIOUG66606-F01|BOLD:ACG2844
Tridactylidae|GMPJA5000-21|BIOUG66606-E12|BOLD:ACG2844
Tridactylidae|GMPJA4999-21|BIOUG66606-E11|BOLD:ACG2844
Ischnurinae|GMPJA4940-21|BIOUG63984-H11|BOLD:AAZ5828
Ischnura aurora|GMPJA8880-21|BIOUG63985-H11|BOLD:ABZ2396
Ischnura aurora|GMPJA8879-21|BIOUG63985-H10|BOLD:ABZ2396
Ischnura aurora|GMPJA4937-21|BIOUG63984-H08|BOLD:ABZ2396
Ischnura aurora|GMPJA4936-21|BIOUG63984-H07|BOLD:ABZ2396
Stephanitis typica|GMPJA9152-21|BIOUG66649-G10|BOLD:ACQ1763
Tingidae|GMPJA9114-21|BIOUG66649-D08|BOLD:AEK1032
Tingidae|GMPJA8956-21|BIOUG66647-G04|BOLD:AEK1032
Tingidae|GMPJA8947-21|BIOUG66647-F07|BOLD:AEK1032
Tingidae|GMPJA9127-21|BIOUG66649-E09|BOLD:AEK1032
Tingidae|GMPJA8920-21|BIOUG66647-D04|BOLD:AEK1032
Tingidae|GMPJA9151-21|BIOUG66649-G09|BOLD:AEK1032
Tingidae|GMPJA9004-21|BIOUG66648-C05|BOLD:AEK1032
Tingidae|GMPJA8950-21|BIOUG66647-F10|BOLD:AEK1032
Tingidae|GMPJA9019-21|BIOUG66648-D08|BOLD:AEK1032
Tingidae|GMPJA9007-21|BIOUG66648-C08|BOLD:AEK1032
Tingidae|GMPJA8923-21|BIOUG66647-D07|BOLD:AEK1032
Tingidae|GMPJA8922-21|BIOUG66647-D06|BOLD:AEK1032
Tingidae|GMPJA9092-21|BIOUG66649-B10|BOLD:AEK1032
Tingidae|GMPJA9032-21|BIOUG66648-E09|BOLD:AEK1032
Tingidae|GMPJA8931-21|BIOUG66647-E03|BOLD:AEK1032
Tingidae|GMPJA8918-21|BIOUG66647-D02|BOLD:AEK1032
Corythauma ayyari|GMPJA7031-21|BIOUG66628-A01|BOLD:ADD1929
Veliidae|GMPJA8981-21|BIOUG66648-A06|BOLD:AEJ4851
Veliidae|GMPJA8944-21|BIOUG66647-F04|BOLD:AEJ4851
Tytthus|GMPJA10455-21|BIOUG70234-G07|BOLD:AAZ2082
Tytthus|GMPJA10457-21|BIOUG70234-G09|BOLD:AAZ2082
Tytthus|GMPJA4832-21|BIOUG66605-G10|BOLD:AAZ2082
Tytthus|GMPJA10470-21|BIOUG70234-H10|BOLD:AAZ2082
Tytthus|GMPJA10441-21|BIOUG70234-F05|BOLD:AAZ2082
Tytthus|GMPJA10461-21|BIOUG70234-H01|BOLD:AAZ2082
Tytthus|GMPJA10465-21|BIOUG70234-H05|BOLD:AAZ2082
Tytthus|GMPJA10421-21|BIOUG70234-D09|BOLD:AAZ2082
Tytthus|GMPJA10460-21|BIOUG70234-G12|BOLD:AAZ2082
Tytthus|GMPJA10414-21|BIOUG70234-D02|BOLD:AAZ2082
Tytthus|GMPJA10399-21|BIOUG70234-B11|BOLD:AAZ2082
Tytthus|GMPJA10404-21|BIOUG70234-C04|BOLD:AAZ2082
Tytthus|GMPJA10391-21|BIOUG70234-B03|BOLD:AAZ2082
Tytthus|GMPJA10413-21|BIOUG70234-D01|BOLD:AAZ2082
Tytthus|GMPJA10438-21|BIOUG70234-F02|BOLD:AAZ2082
Tytthus|GMPJA245-21|BIOUG65658-A06|BOLD:AAZ2082
Tytthus|GMPJA10471-21|BIOUG70234-H11|BOLD:AAZ2082
Tytthus|GMPJA10379-21|BIOUG70234-A03|BOLD:AAZ2082
Eysarcoris aeneus|GMPJA10170-21|BIOUG66563-G08|BOLD:ABX7248
Eysarcoris aeneus|GMPJA5831-21|BIOUG66615-C12|BOLD:ABX7248
Hemiptera|GMPJA323-21|BIOUG65658-G12|BOLD:AEK2663
Anthocoridae|GMPJA3894-21|BIOUG65695-H10|BOLD:ADV1978
Pilophorus|GMPJA9094-21|BIOUG66649-B12|BOLD:AAZ1603
Deraeocoris indianus|GMPJA854-21|BIOUG65664-D09|BOLD:ABX4973
Campylomma|GMPJA3906-21|BIOUG65696-A11|BOLD:ACC0264
Campylomma|GMPJA4960-21|BIOUG66606-B08|BOLD:ACC0264
Rhopalidae|GMPJA5837-21|BIOUG66615-D06|BOLD:ACV2648
Cletus|GMPJA8836-21|BIOUG63985-B07|BOLD:ABX6922
Rhopalus|GMPJA5820-21|BIOUG66615-C01|BOLD:AEU3865
Nysius|GMPJA4843-21|BIOUG66605-H09|BOLD:ACY9090
Nysius|GMPJA3003-21|BIOUG65686-E10|BOLD:ACY9090
Rhyparochromidae|GMPJA10445-21|BIOUG70234-F09|BOLD:ACG4111
Geocoris pallidipennis|GMPJA5801-21|BIOUG66615-A06|BOLD:ADC8565
Geocoris pallidipennis|GMPJA4948-21|BIOUG66606-A08|BOLD:ADC8565
Oxycarenus|GMPJA4845-21|BIOUG66605-H11|BOLD:ABU6102
Oxycarenus|GMPJA810-21|BIOUG65664-A01|BOLD:ABU6102
Oxycarenus|GMPJA10743-21|BIOUG70237-G10|BOLD:ABU6102
Oxycarenus|GMPJA4949-21|BIOUG66606-A09|BOLD:ABU6102
Rhyparochrominae|GMPJA5796-21|BIOUG66615-A01|BOLD:AAP3517
Rhyparochrominae|GMPJA4854-21|BIOUG63984-A09|BOLD:ADL7160
Dysdercus|GMPJA4853-21|BIOUG63984-A08|BOLD:AAM4528
Culicoides imicola|GMPJA3501-21|BIOUG65691-G09|BOLD:AAB8379
Chrysoperla|GMPJA3703-21|BIOUG65693-H09|BOLD:ABX7271
Eupodidae|GMPJA9253-21|BIOUG66650-H04|BOLD:ADV4421
Eupodidae|GMPJA6917-21|BIOUG66626-G05|BOLD:ADV4421
Coleosoma|GMPJA5617-21|BIOUG66613-A12|BOLD:ADZ7438
Glenognatha tangi|GMPJA7098-21|BIOUG66628-F08|BOLD:AAQ0147
Cheiracanthium|GMPJA6301-21|BIOUG66620-C07|BOLD:ACC4872
Cheiracanthium|GMPJA5618-21|BIOUG66613-B01|BOLD:ACC4872
Cheiracanthium|GMPJA3843-21|BIOUG65695-D07|BOLD:ACC4872
Cheiracanthium|GMPJA4987-21|BIOUG66606-D11|BOLD:ACC4872
Cheiracanthium|GMPJA4985-21|BIOUG66606-D09|BOLD:ACC4872
Cheiracanthium|GMPJA3844-21|BIOUG65695-D08|BOLD:ACC4872
Cheiracanthium|GMPJA4982-21|BIOUG66606-D06|BOLD:ACC4872
Micaria|GMPJA6302-21|BIOUG66620-C08|BOLD:ACP3811
Micaria|GMPJA5619-21|BIOUG66613-B02|BOLD:ACP3811
Micaria|GMPJA4988-21|BIOUG66606-D12|BOLD:ACP3811
Oxyopes|GMPJA7094-21|BIOUG66628-F04|BOLD:AAO1991
Lycosidae|GMPXX131-21|BIOUG60810-D03|BOLD:AAO2150
Tmarus|GMPJA5035-21|BIOUG66606-H11|BOLD:ADK4624
Xysticus joyantius|GMPJA4609-21|BIOUG66603-E01|BOLD:ADF4849
Xysticus joyantius|GMPJA4986-21|BIOUG66606-D10|BOLD:ADF4849
Olios tener|GMPJA7095-21|BIOUG66628-F05|BOLD:AEJ1895
Dictynidae|GMPJA4607-21|BIOUG66603-D11|BOLD:ADG4897
Erigone prominens|GMPJA787-21|BIOUG65663-G01|BOLD:ACX8857
Erigone prominens|GMPJA2744-21|BIOUG65684-C11|BOLD:ACX8857
Gnathonarium|GMPJA4608-21|BIOUG66603-D12|BOLD:AAQ0150
Gnathonarium|GMPJA2742-21|BIOUG65684-C09|BOLD:AAQ0150

Erigone prominens|GMPJA2744-21|BIOUG65684-C11|BOLD:ACX8857
Gnathonarium|GMPJA4608-21|BIOUG66603-D12|BOLD:AAQ0150
Gnathonarium|GMPJA2742-21|BIOUG65684-C09|BOLD:AAQ0150
Gnathonarium|GMPJA7096-21|BIOUG66628-F06|BOLD:AAQ0150
Gnathonarium|GMPJA7097-21|BIOUG66628-F07|BOLD:AAQ0150
Gnathonarium|GMPJA7099-21|BIOUG66628-F09|BOLD:AAQ0150
Gnathonarium|GMPJA7218-21|BIOUG66629-H09|BOLD:AAQ0150
Gnathonarium|GMPJA2743-21|BIOUG65684-C10|BOLD:AAQ0150
Gnathonarium|GMPJA4980-21|BIOUG66606-D04|BOLD:AAQ0150
Coproporus tachyporoides|GMPJA1226-21|BIOUG65668-D01|BOLD:ACY5689
Phoridae|GMPJA3354-21|BIOUG65690-C05|BOLD:AEJ2959
Phoridae|GMPJA502-21|BIOUG65660-G01|BOLD:ACW2563
Phoridae|GMPJA8217-21|BIOUG66640-D11|BOLD:ACV5591
Phoridae|GMPJA7356-21|BIOUG66631-D05|BOLD:ACV5591
Hybotidae|GMPJA8564-21|BIOUG66644-B02|BOLD:ACO4582
Hybotidae|GMPJA450-21|BIOUG65660-B09|BOLD:ACG0243
Hybotidae|GMPJA10658-21|BIOUG70236-H08|BOLD:ACG0243
Hybotidae|GMPJA8160-21|BIOUG66639-H01|BOLD:ACG0243
Hybotidae|GMPJA3233-21|BIOUG65689-A03|BOLD:ACG0243
Hybotidae|GMPJA5502-21|BIOUG66611-H03|BOLD:ACG0243
Hybotidae|GMPJA5312-21|BIOUG66609-H03|BOLD:ACG0243
Porcellionides pruinosus|GMPXX142-21|BIOUG60810-E02|BOLD:AAL0707
Porcellionides pruinosus|GMPJA872-21|BIOUG65664-F03|BOLD:AAL0707
Porcellionides pruinosus|GMPJA870-21|BIOUG65664-F01|BOLD:AAL0707
Porcellionides pruinosus|GMPJA2729-21|BIOUG65684-B08|BOLD:AAL0707
Brunettia|GMPJA2047-21|BIOUG65677-A03|BOLD:ADT3105
Brunettia|GMPJA990-21|BIOUG65665-H02|BOLD:ADT3105
Brunettia|GMPJA1448-21|BIOUG65670-F09|BOLD:AAU4651
Brunettia|GMPJA3565-21|BIOUG65692-E02|BOLD:AAU4651
Brunettia|GMPJA1449-21|BIOUG65670-F10|BOLD:AAU4651
Brunettia|GMPJA1478-21|BIOUG65671-A04|BOLD:AAU4651
Brunettia|GMPJA1292-21|BIOUG65669-A08|BOLD:AAU4651
Brunettia|GMPJA1287-21|BIOUG65669-A03|BOLD:AAU4651
Brunettia|GMPJA8495-21|BIOUG66643-D04|BOLD:AAU4651
Brunettia|GMPJA728-21|BIOUG65663-B02|BOLD:AAU4651
Brunettia|GMPJA2500-21|BIOUG65681-G04|BOLD:AAU4651
Brunettia|GMPJA2457-21|BIOUG65681-C09|BOLD:AAU4651
Psychodidae|GMPJA1676-21|BIOUG65673-A12|BOLD:AEJ4703
Psychodidae|GMPJA2340-21|BIOUG65680-A11|BOLD:AEJ4703
Psychodidae|GMPJA1420-21|BIOUG65670-D05|BOLD:AEJ4703
Psychodidae|GMPJA138-21|BIOUG65656-H05|BOLD:AEJ4703
Psychodidae|GMPJA1954-21|BIOUG65676-A05|BOLD:ACG1040
Psychoda alternata|GMPJA2113-21|BIOUG65677-F09|BOLD:AAF9306
Psychoda alternata|GMPJA1809-21|BIOUG65674-E02|BOLD:AAF9306
Psychoda alternata|GMPJA1882-21|BIOUG65675-C04|BOLD:AAF9306
Psychoda alternata|GMPJA393-21|BIOUG65659-E11|BOLD:AAF9306
Psychodidae|GMPJA1899-21|BIOUG65675-D09|BOLD:ABX2127
Psychodidae|GMPJA1567-21|BIOUG65671-H09|BOLD:ABX2127
Psychodidae|GMPJA2349-21|BIOUG65680-B08|BOLD:ABX2127
Psychodidae|GMPJA972-21|BIOUG65665-F08|BOLD:ABX2127
Psychodidae|GMPJA1829-21|BIOUG65674-F10|BOLD:ABX2127
Psychodidae|GMPJA1612-21|BIOUG65672-D07|BOLD:ABX2127
Psychodidae|GMPJA1678-21|BIOUG65673-B02|BOLD:ABX2127
Psychodidae|GMPJA1352-21|BIOUG65669-F08|BOLD:ABX2127
Psychodidae|GMPJA10645-21|BIOUG70236-G07|BOLD:ABX2127
Psychoda|GMPJA8811-21|BIOUG66646-F11|BOLD:AEG6732
Psychoda|GMPJA4226-21|BIOUG65699-D10|BOLD:AEG6732
Psychoda|GMPJA8820-21|BIOUG66646-G08|BOLD:AEG6732
Psychoda|GMPJA8185-21|BIOUG66640-B03|BOLD:AEG6732
Psychoda|GMPJA8363-21|BIOUG66642-A03|BOLD:ACM4821
Psychoda|GMPJA6405-21|BIOUG66621-D04|BOLD:ACM4821
Psychodidae|GMPJA2228-21|BIOUG65678-H05|BOLD:ABW7944
Psychodidae|GMPJA2405-21|BIOUG65680-G04|BOLD:ABW7944
Psychodidae|GMPJA1500-21|BIOUG65671-C02|BOLD:ABW7944
Psychodidae|GMPJA1492-21|BIOUG65671-B06|BOLD:ABW7944
Psychodidae|GMPJA1456-21|BIOUG65670-G05|BOLD:ABW7944
Psychodidae|GMPJA2369-21|BIOUG65680-D04|BOLD:ABW7944
Psychodidae|GMPJA1742-21|BIOUG65673-G06|BOLD:ABW7944
Psychodidae|GMPJA2458-21|BIOUG65681-C10|BOLD:ABW7944
Psychodidae|GMPJA999-21|BIOUG65665-H11|BOLD:ABW7944
Psychodidae|GMPJA2451-21|BIOUG65681-C03|BOLD:ABW7944
Phoridae|GMPJA8419-21|BIOUG66642-E11|BOLD:AEJ3885
Phoridae|GMPJA7307-21|BIOUG66630-H03|BOLD:ABX8771
Phoridae|GMPJA5211-21|BIOUG66608-G09|BOLD:ABX8771
Lispe|GMPJA6858-21|BIOUG66626-B06|BOLD:ACA1246
Lispe|GMPJA3505-21|BIOUG65691-H01|BOLD:ACA1246
Lispe|GMPJA6781-21|BIOUG66625-C12|BOLD:ACA1246
Lispe|GMPJA4144-21|BIOUG65698-E11|BOLD:ACA1246
Lispe|GMPJA3314-21|BIOUG65689-G12|BOLD:ACA1246
Lispe|GMPJA3172-21|BIOUG65688-D01|BOLD:ACA1246
Lispe|GMPJA1464-21|BIOUG65670-H01|BOLD:ACA1246
Lispe|GMPJA3456-21|BIOUG65691-C12|BOLD:ACA1246
Lispe|GMPJA3349-21|BIOUG65690-B12|BOLD:ACA1246
Lispe|GMPJA3072-21|BIOUG65687-C08|BOLD:ACA1246
Lispe|GMPJA10107-21|BIOUG66563-B05|BOLD:ACA1246
Lispe|GMPJA6262-21|BIOUG66619-H03|BOLD:ACA1246
Lispe assimilis|GMPJA10114-21|BIOUG66563-B12|BOLD:AAZ1114
Lispe assimilis|GMPJA5368-21|BIOUG66610-D12|BOLD:AAZ1114
Lispe assimilis|GMPJA6806-21|BIOUG66625-F01|BOLD:AAZ1114
Lispe assimilis|GMPJA751-21|BIOUG65663-D01|BOLD:AAZ1114
Lispe assimilis|GMPJA4573-21|BIOUG66603-B01|BOLD:AAZ1114
Lispe assimilis|GMPJA10112-21|BIOUG66563-B10|BOLD:AAZ1114
Lispe assimilis|GMPJA3136-21|BIOUG65688-A01|BOLD:AAZ1114
Lispe assimilis|GMPJA3088-21|BIOUG65687-D12|BOLD:AAZ1114
Lispe assimilis|GMPJA2914-21|BIOUG58255-F04|BOLD:AAZ1114
Lispe assimilis|GMPJA10091-21|BIOUG63985-A09|BOLD:AAZ1114
Lispe assimilis|GMPJA6731-21|BIOUG66624-G09|BOLD:AAZ1114
Lispe assimilis|GMPJA6716-21|BIOUG66624-F06|BOLD:AAZ1114
Lispe assimilis|GMPJA6508-21|BIOUG66622-D12|BOLD:AAZ1114
Lispe assimilis|GMPJA6746-21|BIOUG66625-A01|BOLD:AAZ1114
Lispe assimilis|GMPJA3053-21|BIOUG65687-B01|BOLD:AAZ1114
Lispe assimilis|GMPJA3231-21|BIOUG65689-A01|BOLD:AAZ1114
Lispe assimilis|GMPJA4366-21|BIOUG65700-H07|BOLD:AAZ1114

Lispe assimilis|GMPJA3053-21|BIOUG65687-B01|BOLD:AAZ1114
Lispe assimilis|GMPJA3231-21|BIOUG65689-A01|BOLD:AAZ1114
Lispe assimilis|GMPJA4366-21|BIOUG65700-H07|BOLD:AAZ1114
Lispe assimilis|GMPJA3611-21|BIOUG65693-A01|BOLD:AAZ1114
Lispe assimilis|GMPJA3622-21|BIOUG65693-A12|BOLD:AAZ1114
Lispe assimilis|GMPJA4097-21|BIOUG65698-A12|BOLD:AAZ1114
Lispe assimilis|GMPJA040-21|BIOUG58255-D04|BOLD:AAZ1114
Lispe assimilis|GMPJA230-21|BIOUG65657-H02|BOLD:AAZ1114
Lispe assimilis|GMPJA928-21|BIOUG65665-B12|BOLD:AAZ1114
Lispe assimilis|GMPJA2045-21|BIOUG65677-A01|BOLD:AAZ1114
Lispe assimilis|GMPJA2163-21|BIOUG65678-B12|BOLD:AAZ1114
Lispe assimilis|GMPJA3516-21|BIOUG65692-A01|BOLD:AAZ1114
Lispe assimilis|GMPJA3493-21|BIOUG65691-G01|BOLD:AAZ1114
Lispe assimilis|GMPJA4513-21|BIOUG66602-D12|BOLD:AAZ1114
Lispe assimilis|GMPJA6661-21|BIOUG66624-A11|BOLD:AAZ1114
Lispe assimilis|GMPJA4436-21|BIOUG66601-F06|BOLD:AAZ1114
Lispe assimilis|GMPJA182-21|BIOUG65657-D02|BOLD:AAZ1114
Lispe assimilis|GMPJA3450-21|BIOUG65691-C06|BOLD:AAZ1114
Lispe assimilis|GMPJA6840-21|BIOUG66625-H11|BOLD:AAZ1114
Lispe assimilis|GMPJA6189-21|BIOUG66619-B02|BOLD:AAZ1114
Lispe assimilis|GMPJA5582-21|BIOUG66612-F12|BOLD:AAZ1114
Lispe assimilis|GMPJA5576-21|BIOUG66612-F06|BOLD:AAZ1114
Lispe assimilis|GMPJA2756-21|BIOUG65684-D11|BOLD:AAZ1114
Lispe assimilis|GMPJA2496-21|BIOUG65681-F12|BOLD:AAZ1114
Lispe|GMPJA1155-21|BIOUG65667-F01|BOLD:ABW5571
Lispe|GMPJA1084-21|BIOUG65666-H01|BOLD:ABW5571
Lispe|GMPJA5594-21|BIOUG66612-G12|BOLD:ABW5492
Lispe|GMPJA4086-21|BIOUG65698-A01|BOLD:ABW5492
Lispe|GMPJA6687-21|BIOUG66624-D01|BOLD:ABW5492
Lispe|GMPJA6814-21|BIOUG66625-F09|BOLD:ABW5492
Lispe|GMPJA6744-21|BIOUG66624-H10|BOLD:ABW5492
Lispe|GMPJA6810-21|BIOUG66625-F05|BOLD:ABW5492
Lispe|GMPJA5344-21|BIOUG66610-B12|BOLD:ABW5492
Lispe|GMPJA4413-21|BIOUG66601-D07|BOLD:ABW5492
Lispe|GMPJA6563-21|BIOUG66623-A08|BOLD:ABW5492
Lispe|GMPJA3230-21|BIOUG65688-H11|BOLD:ABW5492
Lispe|GMPJA688-21|BIOUG65662-F09|BOLD:ABW5492
Lispe|GMPJA4355-21|BIOUG65700-G08|BOLD:ABW5492
Lispe|GMPJA6497-21|BIOUG66622-D01|BOLD:ABW5492
Lispe|GMPJA6638-21|BIOUG66623-G11|BOLD:ABW5492
Lispe|GMPJA6538-21|BIOUG66622-G06|BOLD:ABW5492
Lispe|GMPJA6389-21|BIOUG66621-B12|BOLD:ABW5492
Lispe|GMPJA5273-21|BIOUG66609-D12|BOLD:ABW5492
Lispe|GMPJA6822-21|BIOUG66625-G05|BOLD:ABW5492
Lispe|GMPJA6723-21|BIOUG66624-G01|BOLD:ABW5492
Lispe|GMPJA6468-21|BIOUG66622-A08|BOLD:ABW5492
Lispe|GMPJA6244-21|BIOUG66619-F09|BOLD:ABW5492
Lispe|GMPJA6627-21|BIOUG66623-F12|BOLD:ABW5492
Lispe|GMPJA5556-21|BIOUG66612-D10|BOLD:ABW5492
Lispe|GMPJA4281-21|BIOUG65700-A06|BOLD:ABW5492
Lispe|GMPJA6284-21|BIOUG66620-B02|BOLD:ABW5492
Lispe|GMPJA5510-21|BIOUG66611-H11|BOLD:ABW5492
Lispe|GMPJA5179-21|BIOUG66608-E01|BOLD:ABW5492
Lispe|GMPJA6562-21|BIOUG66623-A07|BOLD:ABW5492
Lispe|GMPJA10558-21|BIOUG70235-H03|BOLD:ABW5492
Lispe|GMPJA6877-21|BIOUG66626-D01|BOLD:ABW5492
Lispe|GMPJA5231-21|BIOUG66609-A06|BOLD:ABW5492
Lispe|GMPJA7969-21|BIOUG66637-G12|BOLD:ABW5492
Lispe|GMPJA1593-21|BIOUG65672-B12|BOLD:ABW5492
Lispe|GMPJA6460-21|BIOUG66621-H11|BOLD:ABW5492
Lispe|GMPJA656-21|BIOUG65662-D01|BOLD:ABW5492
Lispe|GMPJA10264-21|BIOUG70232-G06|BOLD:ABW5492
Lispe|GMPJA5225-21|BIOUG66608-H11|BOLD:ABW5492
Lispe|GMPJA5203-21|BIOUG66608-G01|BOLD:ABW5492
Lispe|GMPJA3426-21|BIOUG65691-A06|BOLD:ABW5492
Lispe|GMPJA4299-21|BIOUG65700-B12|BOLD:ABW5492
Lispe|GMPJA8277-21|BIOUG66641-A12|BOLD:ABW5492
Lispe|GMPJA6532-21|BIOUG66622-F12|BOLD:ABW5492
Lispe|GMPJA1118-21|BIOUG65667-B12|BOLD:ABW5492
Lispe|GMPJA5243-21|BIOUG66609-B06|BOLD:ABW5492
Lispe|GMPJA2234-21|BIOUG65678-H11|BOLD:ABW5492
Lispe|GMPJA2181-21|BIOUG65678-D06|BOLD:ABW5492
Lispe|GMPJA6834-21|BIOUG66625-H05|BOLD:ABW5492
Lispe|GMPJA3338-21|BIOUG65690-B01|BOLD:ABW5492
Lispe|GMPJA8093-21|BIOUG66639-B06|BOLD:ABW5492
Lispe|GMPJA5522-21|BIOUG66612-A12|BOLD:ABW5492
Lispe|GMPJA10192-21|BIOUG70232-A06|BOLD:ABW5492
Lispe|GMPJA5523-21|BIOUG66612-B01|BOLD:ABW5492
Lispe|GMPJA5196-21|BIOUG66608-F06|BOLD:ABW5492
Lispe|GMPJA5107-21|BIOUG66607-F12|BOLD:ABW5492
Lispe|GMPJA5380-21|BIOUG66610-E12|BOLD:ABW5492
Lispe|GMPJA6407-21|BIOUG66621-D06|BOLD:ABW5492
Lispe|GMPJA6707-21|BIOUG66624-E09|BOLD:ABW5492
Lispe|GMPJA6823-21|BIOUG66625-G06|BOLD:ABW5492
Lispe|GMPJA8640-21|BIOUG66644-H06|BOLD:ABW5492
Lispe|GMPJA10368-21|BIOUG70233-H03|BOLD:ABW5492
Lispe|GMPJA10116-21|BIOUG66563-C02|BOLD:ABW5492
Lispe|GMPJA10200-21|BIOUG70232-B02|BOLD:ABW5492
Lispe|GMPJA4460-21|BIOUG66601-H06|BOLD:ABW5492
Lispe|GMPJA1106-21|BIOUG65667-A12|BOLD:ABW5492
Lispe|GMPJA1949-21|BIOUG65675-H11|BOLD:ABW5492
Lispe|GMPJA205-21|BIOUG65657-F01|BOLD:ABW5492
Lispe|GMPJA4540-21|BIOUG66602-G03|BOLD:ABW5492
Lispe|GMPJA5095-21|BIOUG66607-E12|BOLD:ABW5492
Lispe|GMPJA5321-21|BIOUG66610-A01|BOLD:ABW5492
Lispe|GMPJA1758-21|BIOUG65673-H10|BOLD:ABW5492
Lispe|GMPJA6496-21|BIOUG66622-C12|BOLD:ABW5492
Lispe|GMPJA6509-21|BIOUG66622-E01|BOLD:ABW5492
Lispe|GMPJA6450-21|BIOUG66621-H01|BOLD:ABW5492
Lispe|GMPJA631-21|BIOUG65662-A12|BOLD:ABW5492
Lispe|GMPJA085-21|BIOUG65656-C12|BOLD:ABW5492
Lispe|GMPJA7826-21|BIOUG66636-C12|BOLD:ABW5492
Lispe|GMPJA6604-21|BIOUG66623-E01|BOLD:ABW5492

Lispe|GMPJA085-21|BIOUG65656-C12|BOLD:ABW5492
Lispe|GMPJA7826-21|BIOUG66636-C12|BOLD:ABW5492
Lispe|GMPJA6604-21|BIOUG66623-E01|BOLD:ABW5492
Lispe|GMPJA6598-21|BIOUG66623-D07|BOLD:ABW5492
Lispe|GMPJA6555-21|BIOUG66622-H11|BOLD:ABW5492
Lispe|GMPJA6495-21|BIOUG66622-C11|BOLD:ABW5492
Lispe|GMPJA3515-21|BIOUG65691-H11|BOLD:ABW5492
Lispe|GMPJA5178-21|BIOUG66608-D12|BOLD:ABW5492
Lispe|GMPJA10599-21|BIOUG70236-C09|BOLD:ABW5492
Lispe|GMPJA4015-21|BIOUG65697-C01|BOLD:ABW5492
Lispe|GMPJA905-21|BIOUG65665-A01|BOLD:ABW5492
Lispe|GMPJA3399-21|BIOUG65690-G02|BOLD:ABW5492
Lispe|GMPJA10532-21|BIOUG70235-F01|BOLD:ABW5492
Lispe|GMPJA10229-21|BIOUG70232-D07|BOLD:ABW5492
Lispe|GMPJA6784-21|BIOUG66625-D03|BOLD:ABW5492
Lispe|GMPJA4062-21|BIOUG65697-F12|BOLD:ABW5492
Lispe|GMPJA10169-21|BIOUG66563-G07|BOLD:ABW5492
Lispe|GMPJA6639-21|BIOUG66623-G12|BOLD:ABW5492
Lispe|GMPJA5374-21|BIOUG66610-E06|BOLD:ABW5492
Lispe|GMPJA2419-21|BIOUG65680-H06|BOLD:ABW5492
Lispe|GMPJA4312-21|BIOUG65700-D01|BOLD:ABW5492
Lispe|GMPJA6799-21|BIOUG66625-E06|BOLD:ABW5492
Lispe|GMPJA5558-21|BIOUG66612-D12|BOLD:ABW5492
Lispe|GMPJA6787-21|BIOUG66625-D06|BOLD:ABW5492
Lispe|GMPJA4264-21|BIOUG65699-G12|BOLD:ABW5492
Lispe|GMPJA3074-21|BIOUG65687-C10|BOLD:ABW5492
Lispe|GMPJA5106-21|BIOUG66607-F11|BOLD:ABW5492
Lispe|GMPJA4430-21|BIOUG66601-E12|BOLD:ABW5492
Lispe|GMPJA3048-21|BIOUG65687-A08|BOLD:ABW5492
Lispe|GMPJA5101-21|BIOUG66607-F06|BOLD:ABW5492
Lispe|GMPJA10566-21|BIOUG70235-H11|BOLD:ABW5492
Lispe|GMPJA10553-21|BIOUG70235-G10|BOLD:ABW5492
Lispe|GMPJA537-21|BIOUG65661-B01|BOLD:ABW5492
Lispe|GMPJA10603-21|BIOUG70236-D01|BOLD:ABW5492
Lispe|GMPJA5403-21|BIOUG66610-G11|BOLD:ABW5492
Lispe|GMPJA10644-21|BIOUG70236-G06|BOLD:ABW5492
Lispe|GMPJA6865-21|BIOUG66626-C01|BOLD:ABW5492
Lispe|GMPJA4341-21|BIOUG65700-F06|BOLD:ABW5492
Lispe|GMPJA6886-21|BIOUG66626-D10|BOLD:ABW5492
Lispe|GMPJA6870-21|BIOUG66626-C06|BOLD:ABW5492
Lispe|GMPJA6830-21|BIOUG66625-H01|BOLD:ABW5492
Lispe|GMPJA6800-21|BIOUG66625-E07|BOLD:ABW5492
Lispe|GMPJA4437-21|BIOUG66601-F07|BOLD:ABW5492
Lispe|GMPJA4359-21|BIOUG65700-G12|BOLD:ABW5492
Lispe|GMPJA6675-21|BIOUG66624-C01|BOLD:ABW5492
Lispe|GMPJA6558-21|BIOUG66623-A03|BOLD:ABW5492
Lispe|GMPJA10268-21|BIOUG70232-G10|BOLD:ABW5492
Lispe|GMPJA4571-21|BIOUG66603-A11|BOLD:ABW5492
Lispe|GMPJA6544-21|BIOUG66622-G12|BOLD:ABW5492
Lispe|GMPJA6500-21|BIOUG66622-D04|BOLD:ABW5492
Lispe|GMPJA6285-21|BIOUG66620-B03|BOLD:ABW5492
Lispe|GMPJA6721-21|BIOUG66624-F11|BOLD:ABW5492
Lispe|GMPJA6184-21|BIOUG66619-A09|BOLD:ABW5492
Lispe|GMPJA6461-21|BIOUG66622-A01|BOLD:ABW5492
Lispe|GMPJA3373-21|BIOUG65690-D12|BOLD:ABW5492
Lispe|GMPJA3361-21|BIOUG65690-C12|BOLD:ABW5492
Lispe|GMPJA3160-21|BIOUG65688-C01|BOLD:ABW5492
Lispe|GMPJA8099-21|BIOUG66639-B12|BOLD:ABW5492
Lispe|GMPJA6426-21|BIOUG66621-F01|BOLD:ABW5492
Lispe|GMPJA5601-21|BIOUG66612-H07|BOLD:ABW5492
Lispe|GMPJA5587-21|BIOUG66612-G05|BOLD:ABW5492
Lispe|GMPJA5143-21|BIOUG66608-B01|BOLD:ABW5492
Lispe|GMPJA5130-21|BIOUG66607-H11|BOLD:ABW5492
Lispe|GMPJA5108-21|BIOUG66607-G01|BOLD:ABW5492
Lispe|GMPJA6493-21|BIOUG66622-C09|BOLD:ABW5492
Lispe|GMPJA6413-21|BIOUG66621-D12|BOLD:ABW5492
Lispe|GMPJA4102-21|BIOUG65698-B05|BOLD:ABW5492
Lispe|GMPJA4444-21|BIOUG66601-G02|BOLD:ABW5492
Lispe|GMPJA6751-21|BIOUG66625-A06|BOLD:ABW5492
Lispe|GMPJA6694-21|BIOUG66624-D08|BOLD:ABW5492
Lispe|GMPJA4122-21|BIOUG65698-D01|BOLD:ABW5492
Lispe|GMPJA4192-21|BIOUG65699-A12|BOLD:ABW5492
Lispe|GMPJA150-21|BIOUG65657-A06|BOLD:ABW5492
Lispe|GMPJA370-21|BIOUG65659-C12|BOLD:ABW5492
Lispe|GMPJA1083-21|BIOUG65666-G12|BOLD:ABW5492
Lispe|GMPJA1131-21|BIOUG65667-D01|BOLD:ABW5492
Lispe|GMPJA1172-21|BIOUG65667-G06|BOLD:ABW5492
Lispe|GMPJA1344-21|BIOUG65669-E12|BOLD:ABW5492
Lispe|GMPJA4516-21|BIOUG66602-E03|BOLD:ABW5492
Lispe|GMPJA10616-21|BIOUG70236-E02|BOLD:ABW5492
Lispe|GMPJA3421-21|BIOUG65691-A01|BOLD:ABW5492
Lispe|GMPJA7327-21|BIOUG66631-A12|BOLD:ABW5492
Lispe|GMPJA6818-21|BIOUG66625-G01|BOLD:ABW5492
Lispe|GMPJA6237-21|BIOUG66619-F02|BOLD:ABW5492
Lispe|GMPJA6699-21|BIOUG66624-E01|BOLD:ABW5492
Lispe|GMPJA6704-21|BIOUG66624-E06|BOLD:ABW5492
Lispe|GMPJA3049-21|BIOUG65687-A09|BOLD:ABW5492
Lispe|GMPJA4358-21|BIOUG65700-G11|BOLD:ABW5492
Lispe|GMPJA5600-21|BIOUG66612-H06|BOLD:ABW5492
Lispe|GMPJA5416-21|BIOUG66611-A01|BOLD:ABW5492
Lispe|GMPJA3469-21|BIOUG65691-E01|BOLD:ABW5492
Lispe|GMPJA5216-21|BIOUG66608-H02|BOLD:ABW5492
Lispe|GMPJA5041-21|BIOUG66607-A06|BOLD:ABW5492
Lispe|GMPJA6645-21|BIOUG66623-H06|BOLD:ABW5492
Lispe|GMPJA5413-21|BIOUG66610-H09|BOLD:ABW5492
Lispe|GMPJA5338-21|BIOUG66610-B06|BOLD:ABW5492
Lispe|GMPJA2433-21|BIOUG65681-A09|BOLD:ABW5492
Lispe|GMPJA6816-21|BIOUG66625-F11|BOLD:ABW5492
Lispe|GMPJA6889-21|BIOUG66626-E01|BOLD:ABW5492
Lispe|GMPJA6603-21|BIOUG66623-D12|BOLD:ABW5492
Lispe|GMPJA6595-21|BIOUG66623-D04|BOLD:ABW5492
Lispe|GMPJA394-21|BIOUG65659-E12|BOLD:ABW5492
Lispe|GMPJA10187-21|BIOUG70232-A01|BOLD:ABW5492

Lispe|GMPJA6595-21|BIOUG66623-D04|BOLD:ABW5492
Lispe|GMPJA394-21|BIOUG65659-E12|BOLD:ABW5492
Lispe|GMPJA10187-21|BIOUG70232-A01|BOLD:ABW5492
Lispe|GMPJA2003-21|BIOUG65676-E06|BOLD:ABW5492
Lispe|GMPJA3236-21|BIOUG65689-A06|BOLD:ABW5492
Lispe|GMPJA6811-21|BIOUG66625-F06|BOLD:ABW5492
Lispe|GMPJA6775-21|BIOUG66625-C06|BOLD:ABW5492
Lispe|GMPJA10168-21|BIOUG66563-G06|BOLD:ABW5492
Lispe|GMPJA6831-21|BIOUG66625-H02|BOLD:ABW5492
Lispe|GMPJA6556-21|BIOUG66623-A01|BOLD:ABW5492
Lispe|GMPJA5528-21|BIOUG66612-B06|BOLD:ABW5492
Lispe|GMPJA5285-21|BIOUG66609-E12|BOLD:ABW5492
Lispe|GMPJA139-21|BIOUG65656-H06|BOLD:ABW5492
Lispe|GMPJA5439-21|BIOUG66611-B12|BOLD:ABW5492
Lispe|GMPJA3438-21|BIOUG65691-B06|BOLD:ABW5492
Lispe|GMPJA8349-21|BIOUG66641-G12|BOLD:ABW5492
Lispe|GMPJA4455-21|BIOUG66601-H01|BOLD:ABW5492
Lispe|GMPJA10636-21|BIOUG70236-F10|BOLD:ABW5492
Lispe|GMPJA181-21|BIOUG65657-D01|BOLD:ABW5492
Lispe|GMPJA4363-21|BIOUG65700-H04|BOLD:ABW5492
Lispe|GMPJA6560-21|BIOUG66623-A05|BOLD:ABW5492
Lispe|GMPJA3331-21|BIOUG65690-A06|BOLD:ABW5492
Lispe|GMPJA2432-21|BIOUG65681-A08|BOLD:ABW5492
Sciaridae|GMPJA2007-21|BIOUG65676-E10|BOLD:ACY2633
Sciaridae|GMPJA3346-21|BIOUG65690-B09|BOLD:ACY2633
Sciaridae|GMPJA8402-21|BIOUG66642-D06|BOLD:ACY2633
Sciaridae|GMPJA3264-21|BIOUG65689-C10|BOLD:ACY2633
Sciaridae|GMPJA4004-21|BIOUG65697-B02|BOLD:ACY2633
Sciaridae|GMPJA4073-21|BIOUG65697-G11|BOLD:ACY2633
Sciaridae|GMPJA7591-21|BIOUG66633-H02|BOLD:ACY2633
Sciaridae|GMPJA5545-21|BIOUG66612-C11|BOLD:ACY2633
Sciaridae|GMPJA7996-21|BIOUG66638-B04|BOLD:AEJ9417
Bradysia impatiens|GMPJA5509-21|BIOUG66611-H10|BOLD:AAV1295
Sciaridae|GMPJA2097-21|BIOUG65677-E05|BOLD:AEL8855
Sciaridae|GMPJA6655-21|BIOUG66624-A05|BOLD:AEL8855
Sciaridae|GMPJA5078-21|BIOUG66607-D07|BOLD:AEL8855
Sciaridae|GMPJA2375-21|BIOUG65680-D10|BOLD:AEK2502
Sciaridae|GMPJA3375-21|BIOUG65690-E02|BOLD:ADU0241
Sciaridae|GMPJA1667-21|BIOUG65673-A03|BOLD:ABW5519
Sciaridae|GMPJA2249-21|BIOUG65679-B03|BOLD:ABW5519
Sciaridae|GMPJA1290-21|BIOUG65669-A06|BOLD:ABW5519
Sciaridae|GMPJA4013-21|BIOUG65697-B11|BOLD:ABW5519
Sciaridae|GMPJA4528-21|BIOUG66602-F03|BOLD:ABW5519
Sciaridae|GMPJA1649-21|BIOUG65672-G08|BOLD:ABW5519
Sciaridae|GMPJA1461-21|BIOUG65670-G10|BOLD:ABW5519
Sciaridae|GMPJA2400-21|BIOUG65680-F11|BOLD:ABW5519
Sciaridae|GMPJA2117-21|BIOUG65677-G01|BOLD:ABW5519
Sciaridae|GMPJA3395-21|BIOUG65690-F10|BOLD:ABW5519
Sciaridae|GMPJA2212-21|BIOUG65678-G01|BOLD:ABW5519
Sciaridae|GMPJA2311-21|BIOUG65679-G05|BOLD:ABW5519
Sciaridae|GMPJA2061-21|BIOUG65677-B05|BOLD:ABW5519
Sciaridae|GMPJA2075-21|BIOUG65677-C07|BOLD:ABW5519
Sciaridae|GMPJA2085-21|BIOUG65677-D05|BOLD:ABW5519
Sciaridae|GMPJA2161-21|BIOUG65678-B10|BOLD:ABW5519
Sciaridae|GMPJA2066-21|BIOUG65677-B10|BOLD:ABW5519
Sciaridae|GMPJA1480-21|BIOUG65671-A06|BOLD:ABW5519
Sciaridae|GMPJA1864-21|BIOUG65675-A10|BOLD:ABW5519
Sciaridae|GMPJA1303-21|BIOUG65669-B07|BOLD:ABW5519
Sciaridae|GMPJA208-21|BIOUG65657-F04|BOLD:ABW5519
Sciaridae|GMPJA2501-21|BIOUG65681-G05|BOLD:ABW5519
Sciaridae|GMPJA2313-21|BIOUG65679-G07|BOLD:ABW5519
Sciaridae|GMPJA2460-21|BIOUG65681-C12|BOLD:ABW5519
Sciaridae|GMPJA1857-21|BIOUG65675-A03|BOLD:ABW5519
Sciaridae|GMPJA1463-21|BIOUG65670-G12|BOLD:ABW5519
Sciaridae|GMPJA1496-21|BIOUG65671-B10|BOLD:ABW5519
Sciaridae|GMPJA1656-21|BIOUG65672-H03|BOLD:ABW5519
Sciaridae|GMPJA754-21|BIOUG65663-D04|BOLD:ABW5519
Sciaridae|GMPJA1935-21|BIOUG65675-G09|BOLD:ABW5519
Sciaridae|GMPJA2489-21|BIOUG65681-F05|BOLD:ABW5519
Sciaridae|GMPJA2482-21|BIOUG65681-E10|BOLD:ABW5519
Sciaridae|GMPJA2479-21|BIOUG65681-E07|BOLD:ABW5519
Sciaridae|GMPJA1419-21|BIOUG65670-D04|BOLD:ABW5519
Sciaridae|GMPJA2450-21|BIOUG65681-C02|BOLD:ABW5519
Sciaridae|GMPJA424-21|BIOUG65659-H06|BOLD:ABX2119
Sciaridae|GMPJA2170-21|BIOUG65678-C07|BOLD:ABX1230
Sciaridae|GMPJA1636-21|BIOUG65672-F07|BOLD:ABX1230
Sciaridae|GMPJA3262-21|BIOUG65689-C08|BOLD:ABX1230
Sciaridae|GMPJA1609-21|BIOUG65672-D04|BOLD:ABX1230
Sciaridae|GMPJA145-21|BIOUG65657-A01|BOLD:ABX1230
Sciaridae|GMPJA050-21|BIOUG65656-A01|BOLD:ABX1230
Sciaridae|GMPJA543-21|BIOUG65661-B07|BOLD:ABX1230
Sciaridae|GMPJA3470-21|BIOUG65691-E02|BOLD:ABX1230
Sciaridae|GMPJA10360-21|BIOUG70233-G07|BOLD:ABX1230
Bradysia nomica|GMPJA2380-21|BIOUG65680-E03|BOLD:ABW7972
Bradysia nomica|GMPJA1610-21|BIOUG65672-D05|BOLD:ABW7972
Bradysia nomica|GMPJA1937-21|BIOUG65675-G11|BOLD:ABW7972
Bradysia nomica|GMPJA2317-21|BIOUG65679-G11|BOLD:ABW7972
Bradysia nomica|GMPJA2124-21|BIOUG65677-G08|BOLD:ABW7972
Bradysia nomica|GMPJA3414-21|BIOUG65690-H05|BOLD:ABW7972
Bradysia nomica|GMPJA2204-21|BIOUG65678-F05|BOLD:ABW7972
Bradysia nomica|GMPJA1383-21|BIOUG65670-A04|BOLD:ABW7972
Bradysia nomica|GMPJA1163-21|BIOUG65667-F09|BOLD:ABW7972
Bradysia nomica|GMPJA3437-21|BIOUG65691-B05|BOLD:ABW7972
Bradysia nomica|GMPJA3436-21|BIOUG65691-B04|BOLD:ABW7972
Bradysia nomica|GMPJA1685-21|BIOUG65673-B09|BOLD:ABW7972
Bradysia nomica|GMPJA1823-21|BIOUG65674-F04|BOLD:ABW7972
Bradysia nomica|GMPJA3153-21|BIOUG65688-B06|BOLD:ABW7972
Bradysia nomica|GMPJA3166-21|BIOUG65688-C07|BOLD:ABW7972
Bradysia nomica|GMPJA2504-21|BIOUG65681-G08|BOLD:ABW7972
Sciaridae|GMPJA1455-21|BIOUG65670-G04|BOLD:AFH1570
Sciaridae|GMPJA10258-21|BIOUG70232-F12|BOLD:AFH1570
Sciaridae|GMPJA3114-21|BIOUG65687-G02|BOLD:AEK3817
Sciaridae|GMPJA722-21|BIOUG65663-A08|BOLD:ABX2132

[illegible]
Sciaridae|GMPJA10258-21|BIOUG70232-F12|BOLD:AFH1570
Sciaridae|GMPJA3114-21|BIOUG65687-G02|BOLD:AEK3817
Sciaridae|GMPJA722-21|BIOUG65663-A08|BOLD:ABX2132
Sciaridae|GMPJA3550-21|BIOUG65692-C11|BOLD:ABX2132
Sciaridae|GMPJA3431-21|BIOUG65691-A11|BOLD:ABX2132
Sciaridae|GMPJA698-21|BIOUG65662-G07|BOLD:ABX2132
Sciaridae|GMPJA3519-21|BIOUG65692-A04|BOLD:ABX2132
Sciaridae|GMPJA3446-21|BIOUG65691-C02|BOLD:ABX2132
Sciaridae|GMPJA3435-21|BIOUG65691-B03|BOLD:ABX2132
Sciaridae|GMPJA3447-21|BIOUG65691-C03|BOLD:ABX2132
Bradysia|GMPJA2262-21|BIOUG65679-C04|BOLD:ADT4118
Bradysia|GMPJA124-21|BIOUG65656-G03|BOLD:ADU9444
Bradysia ocellaris|GMPJA7791-21|BIOUG66636-A01|BOLD:ABW7969
Bradysia ocellaris|GMPJA4154-21|BIOUG65698-F09|BOLD:AAO8565
Bradysia ocellaris|GMPJA1783-21|BIOUG65674-B12|BOLD:AAO8565
Bradysia ocellaris|GMPJA1848-21|BIOUG65674-H05|BOLD:AAO8565
Bradysia ocellaris|GMPJA1568-21|BIOUG65671-H10|BOLD:AAO8565
Bradysia ocellaris|GMPJA466-21|BIOUG65660-D01|BOLD:AAO8565
Bradysia ocellaris|GMPJA2366-21|BIOUG65680-D01|BOLD:AAO8565
Bradysia ocellaris|GMPJA621-21|BIOUG65662-A02|BOLD:AAO8565
Bradysia ocellaris|GMPJA753-21|BIOUG65663-D03|BOLD:AAO8565
Bradysia ocellaris|GMPJA6789-21|BIOUG66625-D08|BOLD:AAO8565
Bradysia ocellaris|GMPJA7683-21|BIOUG66634-G11|BOLD:AAO8565
Bradysia ocellaris|GMPJA7334-21|BIOUG66631-B07|BOLD:AAO8565
Bradysia ocellaris|GMPJA7995-21|BIOUG66638-B03|BOLD:AAO8565
Bradysia ocellaris|GMPJA8042-21|BIOUG66638-F02|BOLD:AAO8565
Bradysia ocellaris|GMPJA7562-21|BIOUG66633-E09|BOLD:AAO8565
Bradysia ocellaris|GMPJA7770-21|BIOUG66635-G03|BOLD:AAO8565
Bradysia ocellaris|GMPJA7965-21|BIOUG66637-G08|BOLD:AAO8565
Bradysia ocellaris|GMPJA8557-21|BIOUG66644-A07|BOLD:AAO8565
Bradysia ocellaris|GMPJA10244-21|BIOUG70232-E10|BOLD:AAO8565
Bradysia ocellaris|GMPJA3268-21|BIOUG65689-D02|BOLD:AAO8565
Bradysia ocellaris|GMPJA10363-21|BIOUG70233-G10|BOLD:AAO8565
Bradysia ocellaris|GMPJA10544-21|BIOUG70235-G01|BOLD:AAO8565
Bradysia ocellaris|GMPJA10589-21|BIOUG70236-B11|BOLD:AAO8565
Bradysia ocellaris|GMPJA10628-21|BIOUG70236-F02|BOLD:AAO8565
Bradysia ocellaris|GMPJA3389-21|BIOUG65690-F04|BOLD:AAO8565
Bradysia ocellaris|GMPJA3079-21|BIOUG65687-D03|BOLD:AAO8565
Bradysia ocellaris|GMPJA123-21|BIOUG65656-G02|BOLD:AAO8565
Bradysia ocellaris|GMPJA136-21|BIOUG65656-H03|BOLD:AAO8565
Bradysia ocellaris|GMPJA141-21|BIOUG65656-H08|BOLD:AAO8565
Bradysia ocellaris|GMPJA411-21|BIOUG65659-G05|BOLD:AAO8565
Bradysia ocellaris|GMPJA427-21|BIOUG65659-H09|BOLD:AAO8565
Bradysia ocellaris|GMPJA431-21|BIOUG65660-A02|BOLD:AAO8565
Bradysia ocellaris|GMPJA435-21|BIOUG65660-A06|BOLD:AAO8565
Bradysia ocellaris|GMPJA455-21|BIOUG65660-C02|BOLD:AAO8565
Bradysia ocellaris|GMPJA461-21|BIOUG65660-C08|BOLD:AAO8565
Bradysia ocellaris|GMPJA462-21|BIOUG65660-C09|BOLD:AAO8565
Bradysia ocellaris|GMPJA467-21|BIOUG65660-D02|BOLD:AAO8565
Bradysia ocellaris|GMPJA470-21|BIOUG65660-D05|BOLD:AAO8565
Bradysia ocellaris|GMPJA475-21|BIOUG65660-D10|BOLD:AAO8565
Bradysia ocellaris|GMPJA479-21|BIOUG65660-E02|BOLD:AAO8565
Bradysia ocellaris|GMPJA483-21|BIOUG65660-E06|BOLD:AAO8565
Bradysia ocellaris|GMPJA500-21|BIOUG65660-F11|BOLD:AAO8565
Bradysia ocellaris|GMPJA510-21|BIOUG65660-G09|BOLD:AAO8565
Bradysia ocellaris|GMPJA517-21|BIOUG65660-H04|BOLD:AAO8565
Bradysia ocellaris|GMPJA521-21|BIOUG65660-H08|BOLD:AAO8565
Bradysia ocellaris|GMPJA528-21|BIOUG65661-A04|BOLD:AAO8565
Bradysia ocellaris|GMPJA532-21|BIOUG65661-A08|BOLD:AAO8565
Bradysia ocellaris|GMPJA546-21|BIOUG65661-B10|BOLD:AAO8565
Bradysia ocellaris|GMPJA061-21|BIOUG65656-A12|BOLD:AAO8565
Bradysia ocellaris|GMPJA072-21|BIOUG65656-B11|BOLD:AAO8565
Bradysia ocellaris|GMPJA075-21|BIOUG65656-C02|BOLD:AAO8565
Bradysia ocellaris|GMPJA092-21|BIOUG65656-D07|BOLD:AAO8565
Bradysia ocellaris|GMPJA093-21|BIOUG65656-D08|BOLD:AAO8565
Bradysia ocellaris|GMPJA094-21|BIOUG65656-D09|BOLD:AAO8565
Bradysia ocellaris|GMPJA103-21|BIOUG65656-E06|BOLD:AAO8565
Bradysia ocellaris|GMPJA105-21|BIOUG65656-E08|BOLD:AAO8565
Bradysia ocellaris|GMPJA108-21|BIOUG65656-E11|BOLD:AAO8565
Bradysia ocellaris|GMPJA109-21|BIOUG65656-E12|BOLD:AAO8565
Bradysia ocellaris|GMPJA110-21|BIOUG65656-F01|BOLD:AAO8565
Bradysia ocellaris|GMPJA155-21|BIOUG65657-A11|BOLD:AAO8565
Bradysia ocellaris|GMPJA160-21|BIOUG65657-B04|BOLD:AAO8565
Bradysia ocellaris|GMPJA163-21|BIOUG65657-B07|BOLD:AAO8565
Bradysia ocellaris|GMPJA185-21|BIOUG65657-D05|BOLD:AAO8565
Bradysia ocellaris|GMPJA190-21|BIOUG65657-D10|BOLD:AAO8565
Bradysia ocellaris|GMPJA197-21|BIOUG65657-E05|BOLD:AAO8565
Bradysia ocellaris|GMPJA199-21|BIOUG65657-E07|BOLD:AAO8565
Bradysia ocellaris|GMPJA219-21|BIOUG65657-G03|BOLD:AAO8565
Bradysia ocellaris|GMPJA220-21|BIOUG65657-G04|BOLD:AAO8565
Bradysia ocellaris|GMPJA227-21|BIOUG65657-G11|BOLD:AAO8565
Bradysia ocellaris|GMPJA337-21|BIOUG65659-A03|BOLD:AAO8565
Bradysia ocellaris|GMPJA340-21|BIOUG65659-A06|BOLD:AAO8565
Bradysia ocellaris|GMPJA342-21|BIOUG65659-A08|BOLD:AAO8565
Bradysia ocellaris|GMPJA343-21|BIOUG65659-A09|BOLD:AAO8565
Bradysia ocellaris|GMPJA348-21|BIOUG65659-B02|BOLD:AAO8565
Bradysia ocellaris|GMPJA356-21|BIOUG65659-B10|BOLD:AAO8565
Bradysia ocellaris|GMPJA361-21|BIOUG65659-C03|BOLD:AAO8565
Bradysia ocellaris|GMPJA374-21|BIOUG65659-D04|BOLD:AAO8565
Bradysia ocellaris|GMPJA375-21|BIOUG65659-D05|BOLD:AAO8565
Bradysia ocellaris|GMPJA380-21|BIOUG65659-D10|BOLD:AAO8565
Bradysia ocellaris|GMPJA385-21|BIOUG65659-E03|BOLD:AAO8565
Bradysia ocellaris|GMPJA387-21|BIOUG65659-E05|BOLD:AAO8565
Bradysia ocellaris|GMPJA388-21|BIOUG65659-E06|BOLD:AAO8565
Bradysia ocellaris|GMPJA389-21|BIOUG65659-E07|BOLD:AAO8565
Bradysia ocellaris|GMPJA642-21|BIOUG65662-B11|BOLD:AAO8565
Bradysia ocellaris|GMPJA690-21|BIOUG65662-F11|BOLD:AAO8565
Bradysia ocellaris|GMPJA1330-21|BIOUG65669-D10|BOLD:AAO8565
Bradysia ocellaris|GMPJA701-21|BIOUG65662-G10|BOLD:AAO8565
Bradysia ocellaris|GMPJA742-21|BIOUG65663-C04|BOLD:AAO8565
Bradysia ocellaris|GMPJA745-21|BIOUG65663-C07|BOLD:AAO8565
Bradysia ocellaris|GMPJA1010-21|BIOUG65666-A11|BOLD:AAO8565
Bradysia ocellaris|GMPJA1125-21|BIOUG65667-C07|BOLD:AAO8565

Bradysia ocellaris|GMPJA742-21|BIOUG65663-C04|BOLD:AAO8565
Bradysia ocellaris|GMPJA745-21|BIOUG65663-C07|BOLD:AAO8565
Bradysia ocellaris|GMPJA1010-21|BIOUG65666-A11|BOLD:AAO8565
Bradysia ocellaris|GMPJA1125-21|BIOUG65667-C07|BOLD:AAO8565
Bradysia ocellaris|GMPJA1396-21|BIOUG65670-B05|BOLD:AAO8565
Bradysia ocellaris|GMPJA1399-21|BIOUG65670-B08|BOLD:AAO8565
Bradysia ocellaris|GMPJA1405-21|BIOUG65670-C02|BOLD:AAO8565
Bradysia ocellaris|GMPJA1414-21|BIOUG65670-C11|BOLD:AAO8565
Bradysia ocellaris|GMPJA1838-21|BIOUG65674-G07|BOLD:AAO8565
Bradysia ocellaris|GMPJA1849-21|BIOUG65674-H06|BOLD:AAO8565
Bradysia ocellaris|GMPJA1858-21|BIOUG65675-A04|BOLD:AAO8565
Bradysia ocellaris|GMPJA1861-21|BIOUG65675-A07|BOLD:AAO8565
Bradysia ocellaris|GMPJA1886-21|BIOUG65675-C08|BOLD:AAO8565
Bradysia ocellaris|GMPJA1892-21|BIOUG65675-D02|BOLD:AAO8565
Bradysia ocellaris|GMPJA1919-21|BIOUG65675-F05|BOLD:AAO8565
Bradysia ocellaris|GMPJA1429-21|BIOUG65670-E02|BOLD:AAO8565
Bradysia ocellaris|GMPJA1504-21|BIOUG65671-C06|BOLD:AAO8565
Bradysia ocellaris|GMPJA1547-21|BIOUG65671-G01|BOLD:AAO8565
Bradysia ocellaris|GMPJA1589-21|BIOUG65672-B08|BOLD:AAO8565
Bradysia ocellaris|GMPJA2227-21|BIOUG65678-H04|BOLD:AAO8565
Bradysia ocellaris|GMPJA2230-21|BIOUG65678-H07|BOLD:AAO8565
Bradysia ocellaris|GMPJA2231-21|BIOUG65678-H08|BOLD:AAO8565
Bradysia ocellaris|GMPJA2353-21|BIOUG65680-B12|BOLD:AAO8565
Bradysia ocellaris|GMPJA2358-21|BIOUG65680-C05|BOLD:AAO8565
Bradysia ocellaris|GMPJA1660-21|BIOUG65672-H07|BOLD:AAO8565
Bradysia ocellaris|GMPJA1692-21|BIOUG65673-C04|BOLD:AAO8565
Bradysia ocellaris|GMPJA1704-21|BIOUG65673-D04|BOLD:AAO8565
Bradysia ocellaris|GMPJA1751-21|BIOUG65673-H03|BOLD:AAO8565
Bradysia ocellaris|GMPJA1794-21|BIOUG65674-C11|BOLD:AAO8565
Bradysia ocellaris|GMPJA2002-21|BIOUG65676-E05|BOLD:AAO8565
Bradysia ocellaris|GMPJA2005-21|BIOUG65676-E08|BOLD:AAO8565
Bradysia ocellaris|GMPJA2013-21|BIOUG65676-F04|BOLD:AAO8565
Bradysia ocellaris|GMPJA2014-21|BIOUG65676-F05|BOLD:AAO8565
Bradysia ocellaris|GMPJA2041-21|BIOUG65676-H08|BOLD:AAO8565
Bradysia ocellaris|GMPJA2048-21|BIOUG65677-A04|BOLD:AAO8565
Bradysia ocellaris|GMPJA2052-21|BIOUG65677-A08|BOLD:AAO8565
Bradysia ocellaris|GMPJA2053-21|BIOUG65677-A09|BOLD:AAO8565
Bradysia ocellaris|GMPJA2083-21|BIOUG65677-D03|BOLD:AAO8565
Bradysia ocellaris|GMPJA2125-21|BIOUG65677-G09|BOLD:AAO8565
Bradysia ocellaris|GMPJA2135-21|BIOUG65677-H07|BOLD:AAO8565
Bradysia ocellaris|GMPJA449-21|BIOUG65660-B08|BOLD:AAO8565
Bradysia ocellaris|GMPJA1111-21|BIOUG65667-B05|BOLD:AAO8565
Bradysia ocellaris|GMPJA2408-21|BIOUG65680-G07|BOLD:AAO8565
Bradysia ocellaris|GMPJA2218-21|BIOUG65678-G07|BOLD:AAO8565
Bradysia ocellaris|GMPJA10535-21|BIOUG70235-F04|BOLD:AAO8565
Bradysia ocellaris|GMPJA523-21|BIOUG65660-H10|BOLD:AAO8565
Bradysia ocellaris|GMPJA357-21|BIOUG65659-B11|BOLD:AAO8565
Bradysia ocellaris|GMPJA634-21|BIOUG65662-B03|BOLD:AAO8565
Bradysia ocellaris|GMPJA1327-21|BIOUG65669-D07|BOLD:AAO8565
Bradysia ocellaris|GMPJA1947-21|BIOUG65675-H09|BOLD:AAO8565
Bradysia ocellaris|GMPJA2094-21|BIOUG65677-E02|BOLD:AAO8565
Bradysia ocellaris|GMPJA096-21|BIOUG65656-D11|BOLD:AAO8565
Bradysia ocellaris|GMPJA6594-21|BIOUG66623-D03|BOLD:AAO8565
Bradysia ocellaris|GMPJA2758-21|BIOUG65684-E01|BOLD:AAO8565
Bradysia ocellaris|GMPJA2509-21|BIOUG65681-H01|BOLD:AAO8565
Bradysia ocellaris|GMPJA2494-21|BIOUG65681-F10|BOLD:AAO8565
Bradysia ocellaris|GMPJA2491-21|BIOUG65681-F07|BOLD:AAO8565
Sciaridae|GMPJA2216-21|BIOUG65678-G05|BOLD:ABW5532
Sciaridae|GMPJA4356-21|BIOUG65700-G09|BOLD:ABW5532
Sciaridae|GMPJA711-21|BIOUG65662-H08|BOLD:AEK0603
Lycoriella|GMPJA3922-21|BIOUG65696-C03|BOLD:AAH4015
Sciaridae|GMPJA1078-21|BIOUG65666-G07|BOLD:ABW5516
Sciaridae|GMPJA1672-21|BIOUG65673-A08|BOLD:ABW5516
Sciaridae|GMPJA188-21|BIOUG65657-D08|BOLD:ABW5516
Sciaridae|GMPJA655-21|BIOUG65662-C12|BOLD:ABW5516
Sciaridae|GMPJA1332-21|BIOUG65669-D12|BOLD:ABW5516
Sciaridae|GMPJA3601-21|BIOUG65692-H02|BOLD:ABW5516
Sciaridae|GMPJA5258-21|BIOUG66609-C09|BOLD:ABX2162
Sciaridae|GMPJA8746-21|BIOUG66646-A06|BOLD:ABX2162
Sciaridae|GMPJA5066-21|BIOUG66607-C07|BOLD:ABX2162
Scatopsciara alawae|GMPJA4113-21|BIOUG65698-C04|BOLD:AAH3962
Scatopsciara alawae|GMPJA4251-21|BIOUG65699-F11|BOLD:AAH3962
Sciaridae|GMPJA2511-21|BIOUG65681-H03|BOLD:ACA3148
Sciaridae|GMPJA4053-21|BIOUG65697-F03|BOLD:ACA3148
Sciaridae|GMPJA2153-21|BIOUG65678-B02|BOLD:ACA3148
Sciaridae|GMPJA1477-21|BIOUG65671-A03|BOLD:ACA3148
Sciaridae|GMPJA3402-21|BIOUG65690-G05|BOLD:ACA3148
Sciaridae|GMPJA1499-21|BIOUG65671-C01|BOLD:ACA3148
Sciaridae|GMPJA2765-21|BIOUG65684-E08|BOLD:ACA3148
Sciaridae|GMPJA1669-21|BIOUG65673-A05|BOLD:ACA3148
Sciaridae|GMPJA2131-21|BIOUG65677-H03|BOLD:ACA3148
Sciaridae|GMPJA962-21|BIOUG65665-E10|BOLD:ACA3148
Sciaridae|GMPJA2274-21|BIOUG65679-D04|BOLD:ACA3148
Sciaridae|GMPJA3452-21|BIOUG65691-C08|BOLD:ACA3148
Sciaridae|GMPJA3131-21|BIOUG65687-H07|BOLD:ACA3148
Sciaridae|GMPJA2202-21|BIOUG65678-F03|BOLD:ACA3148
Sciaridae|GMPJA1513-21|BIOUG65671-D03|BOLD:ACA3148
Sciaridae|GMPJA3122-21|BIOUG65687-G10|BOLD:ACA3148
Sciaridae|GMPJA3223-21|BIOUG65688-H04|BOLD:ACA3148
Sciaridae|GMPJA3249-21|BIOUG65689-B07|BOLD:ACA3148
Sciaridae|GMPJA3269-21|BIOUG65689-D03|BOLD:ACA3148
Sciaridae|GMPJA3274-21|BIOUG65689-D08|BOLD:ACA3148
Sciaridae|GMPJA3316-21|BIOUG65689-H02|BOLD:ACA3148
Sciaridae|GMPJA3384-21|BIOUG65690-E11|BOLD:ACA3148
Sciaridae|GMPJA3418-21|BIOUG65690-H09|BOLD:ACA3148
Sciaridae|GMPJA3441-21|BIOUG65691-B09|BOLD:ACA3148
Sciaridae|GMPJA3416-21|BIOUG65690-H07|BOLD:ACA3148
Sciaridae|GMPJA3422-21|BIOUG65691-A02|BOLD:ACA3148
Sciaridae|GMPJA3118-21|BIOUG65687-G06|BOLD:ACA3148
Sciaridae|GMPJA3221-21|BIOUG65688-H02|BOLD:ACA3148
Sciaridae|GMPJA3247-21|BIOUG65689-B05|BOLD:ACA3148
Sciaridae|GMPJA3675-21|BIOUG65693-F05|BOLD:ACA3148
Sciaridae|GMPJA3318-21|BIOUG65689-H04|BOLD:ACA3148
Sciaridae|GMPJA[illegible]|BIOUG656[illegible]|BOLD:ACA3148

Sciaridae|GMPJA3247-21|BIOUG65689-B05|BOLD:ACA3148
Sciaridae|GMPJA3675-21|BIOUG65693-F05|BOLD:ACA3148
Sciaridae|GMPJA3318-21|BIOUG65689-H04|BOLD:ACA3148
Sciaridae|GMPJA3365-21|BIOUG65690-D04|BOLD:ACA3148
Sciaridae|GMPJA3471-21|BIOUG65691-E03|BOLD:ACA3148
Sciaridae|GMPJA3482-21|BIOUG65691-F02|BOLD:ACA3148
Sciaridae|GMPJA3500-21|BIOUG65691-G08|BOLD:ACA3148
Sciaridae|GMPJA3509-21|BIOUG65691-H05|BOLD:ACA3148
Sciaridae|GMPJA3512-21|BIOUG65691-H08|BOLD:ACA3148
Sciaridae|GMPJA4233-21|BIOUG65699-E05|BOLD:ACA3148
Sciaridae|GMPJA3532-21|BIOUG65692-B05|BOLD:ACA3148
Sciaridae|GMPJA3558-21|BIOUG65692-D07|BOLD:ACA3148
Sciaridae|GMPJA3577-21|BIOUG65692-F02|BOLD:ACA3148
Sciaridae|GMPJA3587-21|BIOUG65692-F12|BOLD:ACA3148
Sciaridae|GMPJA3595-21|BIOUG65692-G08|BOLD:ACA3148
Sciaridae|GMPJA3596-21|BIOUG65692-G09|BOLD:ACA3148
Sciaridae|GMPJA3597-21|BIOUG65692-G10|BOLD:ACA3148
Sciaridae|GMPJA3613-21|BIOUG65693-A03|BOLD:ACA3148
Sciaridae|GMPJA3629-21|BIOUG65693-B07|BOLD:ACA3148
Sciaridae|GMPJA3642-21|BIOUG65693-C08|BOLD:ACA3148
Sciaridae|GMPJA3649-21|BIOUG65693-D03|BOLD:ACA3148
Sciaridae|GMPJA3656-21|BIOUG65693-D10|BOLD:ACA3148
Sciaridae|GMPJA3660-21|BIOUG65693-E02|BOLD:ACA3148
Sciaridae|GMPJA3928-21|BIOUG65696-C09|BOLD:ACA3148
Sciaridae|GMPJA4174-21|BIOUG65698-H05|BOLD:ACA3148
Sciaridae|GMPJA1293-21|BIOUG65669-A09|BOLD:ACA3148
Sciaridae|GMPJA1319-21|BIOUG65669-C11|BOLD:ACA3148
Sciaridae|GMPJA1348-21|BIOUG65669-F04|BOLD:ACA3148
Sciaridae|GMPJA1382-21|BIOUG65670-A03|BOLD:ACA3148
Sciaridae|GMPJA1415-21|BIOUG65670-C12|BOLD:ACA3148
Sciaridae|GMPJA1804-21|BIOUG65674-D09|BOLD:ACA3148
Sciaridae|GMPJA1812-21|BIOUG65674-E05|BOLD:ACA3148
Sciaridae|GMPJA1824-21|BIOUG65674-F05|BOLD:ACA3148
Sciaridae|GMPJA1833-21|BIOUG65674-G02|BOLD:ACA3148
Sciaridae|GMPJA1874-21|BIOUG65675-B08|BOLD:ACA3148
Sciaridae|GMPJA1903-21|BIOUG65675-E01|BOLD:ACA3148
Sciaridae|GMPJA1928-21|BIOUG65675-G02|BOLD:ACA3148
Sciaridae|GMPJA1929-21|BIOUG65675-G03|BOLD:ACA3148
Sciaridae|GMPJA1936-21|BIOUG65675-G10|BOLD:ACA3148
Sciaridae|GMPJA1422-21|BIOUG65670-D07|BOLD:ACA3148
Sciaridae|GMPJA1490-21|BIOUG65671-B04|BOLD:ACA3148
Sciaridae|GMPJA1517-21|BIOUG65671-D07|BOLD:ACA3148
Sciaridae|GMPJA1522-21|BIOUG65671-D12|BOLD:ACA3148
Sciaridae|GMPJA1584-21|BIOUG65672-B03|BOLD:ACA3148
Sciaridae|GMPJA1631-21|BIOUG65672-F02|BOLD:ACA3148
Sciaridae|GMPJA1632-21|BIOUG65672-F03|BOLD:ACA3148
Sciaridae|GMPJA1633-21|BIOUG65672-F04|BOLD:ACA3148
Sciaridae|GMPJA2182-21|BIOUG65678-D07|BOLD:ACA3148
Sciaridae|GMPJA2195-21|BIOUG65678-E08|BOLD:ACA3148
Sciaridae|GMPJA2199-21|BIOUG65678-E12|BOLD:ACA3148
Sciaridae|GMPJA2209-21|BIOUG65678-F10|BOLD:ACA3148
Sciaridae|GMPJA2265-21|BIOUG65679-C07|BOLD:ACA3148
Sciaridae|GMPJA2324-21|BIOUG65679-H06|BOLD:ACA3148
Sciaridae|GMPJA2339-21|BIOUG65680-A10|BOLD:ACA3148
Sciaridae|GMPJA2345-21|BIOUG65680-B04|BOLD:ACA3148
Sciaridae|GMPJA2359-21|BIOUG65680-C06|BOLD:ACA3148
Sciaridae|GMPJA1668-21|BIOUG65673-A04|BOLD:ACA3148
Sciaridae|GMPJA1680-21|BIOUG65673-B04|BOLD:ACA3148
Sciaridae|GMPJA1963-21|BIOUG65676-B02|BOLD:ACA3148
Sciaridae|GMPJA1993-21|BIOUG65676-D08|BOLD:ACA3148
Sciaridae|GMPJA2050-21|BIOUG65677-A06|BOLD:ACA3148
Sciaridae|GMPJA1756-21|BIOUG65673-H08|BOLD:ACA3148
Sciaridae|GMPJA1739-21|BIOUG65673-G03|BOLD:ACA3148
Sciaridae|GMPJA2207-21|BIOUG65678-F08|BOLD:ACA3148
Sciaridae|GMPJA1548-21|BIOUG65671-G02|BOLD:ACA3148
Sciaridae|GMPJA1970-21|BIOUG65676-B09|BOLD:ACA3148
Sciaridae|GMPJA2029-21|BIOUG65676-G08|BOLD:ACA3148
Sciaridae|GMPJA2073-21|BIOUG65677-C05|BOLD:ACA3148
Sciaridae|GMPJA2087-21|BIOUG65677-D07|BOLD:ACA3148
Sciaridae|GMPJA2100-21|BIOUG65677-E08|BOLD:ACA3148
Sciaridae|GMPJA2119-21|BIOUG65677-G03|BOLD:ACA3148
Sciaridae|GMPJA2154-21|BIOUG65678-B03|BOLD:ACA3148
Sciaridae|GMPJA1862-21|BIOUG65675-A08|BOLD:ACA3148
Sciaridae|GMPJA1601-21|BIOUG65672-C08|BOLD:ACA3148
Sciaridae|GMPJA3057-21|BIOUG65687-B05|BOLD:ACA3148
Sciaridae|GMPJA2088-21|BIOUG65677-D08|BOLD:ACA3148
Sciaridae|GMPJA1341-21|BIOUG65669-E09|BOLD:ACA3148
Sciaridae|GMPJA3116-21|BIOUG65687-G04|BOLD:ACA3148
Sciaridae|GMPJA3538-21|BIOUG65692-B11|BOLD:ACA3148
Sciaridae|GMPJA3187-21|BIOUG65688-E04|BOLD:ACA3148
Sciaridae|GMPJA2519-21|BIOUG65681-H11|BOLD:ACA3148
Sciaridae|GMPJA2514-21|BIOUG65681-H06|BOLD:ACA3148
Sciaridae|GMPJA2467-21|BIOUG65681-D07|BOLD:ACA3148
Sciaridae|GMPJA2466-21|BIOUG65681-D06|BOLD:ACA3148
Sciaridae|GMPJA2464-21|BIOUG65681-D04|BOLD:ACA3148
Sciaridae|GMPJA2441-21|BIOUG65681-B05|BOLD:ACA3148
Sciaridae|GMPJA3104-21|BIOUG65687-F04|BOLD:ACA3148
Sciaridae|GMPJA2165-21|BIOUG65678-C02|BOLD:ACA3148
Sciaridae|GMPJA3489-21|BIOUG65691-F09|BOLD:ACA3148
Sciaridae|GMPJA1379-21|BIOUG65669-H11|BOLD:ACA3148
Sciaridae|GMPJA2498-21|BIOUG65681-G02|BOLD:ACA3148
Sciaridae|GMPJA2492-21|BIOUG65681-F08|BOLD:ACA3148
Sciaridae|GMPJA1343-21|BIOUG65669-E11|BOLD:ACA3148
Sciaridae|GMPJA3240-21|BIOUG65689-A10|BOLD:ACA3148
Sciaridae|GMPJA1317-21|BIOUG65669-C09|BOLD:ACA3148
Sciaridae|GMPJA3448-21|BIOUG65691-C04|BOLD:ACA3148
Sciaridae|GMPJA1768-21|BIOUG65674-A09|BOLD:ACA3148
Sciaridae|GMPJA3484-21|BIOUG65691-F04|BOLD:ACA3148
Sciaridae|GMPJA2462-21|BIOUG65681-D02|BOLD:ACA3148
Sciaridae|GMPJA3478-21|BIOUG65691-E10|BOLD:ACA3148
Sciaridae|GMPJA3055-21|BIOUG65687-B03|BOLD:ACA3148
Sciaridae|GMPJA3234-21|BIOUG65689-A04|BOLD:ACA3148
Sciaridae|GMPJA3554-21|BIOUG65692-D03|BOLD:ACA3148
[illegible]

Sciaridae|GMPJA3055-21|BIOUG65687-B03|BOLD:ACA3148
Sciaridae|GMPJA3234-21|BIOUG65689-A04|BOLD:ACA3148
Sciaridae|GMPJA3554-21|BIOUG65692-D03|BOLD:ACA3148
Sciaridae|GMPJA2189-21|BIOUG65678-E02|BOLD:ACA3148
Sciaridae|GMPJA3458-21|BIOUG65691-D02|BOLD:ACA3148
Sciaridae|GMPJA3483-21|BIOUG65691-F03|BOLD:ACA3148
Sciaridae|GMPJA1923-21|BIOUG65675-F09|BOLD:ACA3148
Sciaridae|GMPJA2067-21|BIOUG65677-B11|BOLD:ACA3148
Sciaridae|GMPJA3339-21|BIOUG65690-B02|BOLD:ACA3148
Sciaridae|GMPJA3506-21|BIOUG65691-H02|BOLD:ACA3148
Sciaridae|GMPJA3578-21|BIOUG65692-F03|BOLD:ACA3148
Sciaridae|GMPJA2178-21|BIOUG65678-D03|BOLD:ACA3148
Sciaridae|GMPJA1363-21|BIOUG65669-G07|BOLD:ACA3148
Sciaridae|GMPJA1640-21|BIOUG65672-F11|BOLD:ACA3148
Sciaridae|GMPJA1978-21|BIOUG65676-C05|BOLD:ACA3148
Sciaridae|GMPJA2480-21|BIOUG65681-E08|BOLD:ACA3148
Sciaridae|GMPJA3261-21|BIOUG65689-C07|BOLD:ACA3148
Sciaridae|GMPJA3637-21|BIOUG65693-C03|BOLD:ACA3148
Sciaridae|GMPJA1321-21|BIOUG65669-D01|BOLD:ACA3148
Sciaridae|GMPJA1431-21|BIOUG65670-E04|BOLD:ACA3148
Sciaridae|GMPJA2474-21|BIOUG65681-E02|BOLD:ACA3148
Sciaridae|GMPJA2487-21|BIOUG65681-F03|BOLD:ACA3148
Sciaridae|GMPJA2490-21|BIOUG65681-F06|BOLD:ACA3148
Sciaridae|GMPJA2508-21|BIOUG65681-G12|BOLD:ACA3148
Sciaridae|GMPJA2516-21|BIOUG65681-H08|BOLD:ACA3148
Sciaridae|GMPJA2759-21|BIOUG65684-E02|BOLD:ACA3148
Sciaridae|GMPJA2761-21|BIOUG65684-E04|BOLD:ACA3148
Sciaridae|GMPJA2762-21|BIOUG65684-E05|BOLD:ACA3148
Sciaridae|GMPJA2768-21|BIOUG65684-E11|BOLD:ACA3148
Sciaridae|GMPJA5275-21|BIOUG66609-E02|BOLD:ACA3148
Sciaridae|GMPJA3197-21|BIOUG65688-F02|BOLD:ACA3148
Sciaridae|GMPJA3027-21|BIOUG65686-G10|BOLD:ACA3148
Sciaridae|GMPJA3502-21|BIOUG65691-G10|BOLD:ACA3148
Sciaridae|GMPJA3286-21|BIOUG65689-E08|BOLD:ACA3148
Sciaridae|GMPJA3312-21|BIOUG65689-G10|BOLD:ACA3148
Sciaridae|GMPJA3141-21|BIOUG65688-A06|BOLD:ACA3148
Sciaridae|GMPJA3321-21|BIOUG65689-H07|BOLD:ACA3148
Sciaridae|GMPJA3427-21|BIOUG65691-A07|BOLD:ACA3148
Sciaridae|GMPJA3394-21|BIOUG65690-F09|BOLD:ACA3148
Sciaridae|GMPJA3419-21|BIOUG65690-H10|BOLD:ACA3148
Sciaridae|GMPJA3430-21|BIOUG65691-A10|BOLD:ACA3148
Sciaridae|GMPJA3019-21|BIOUG65686-G02|BOLD:ACA3148
Sciaridae|GMPJA3024-21|BIOUG65686-G07|BOLD:ACA3148
Sciaridae|GMPJA3026-21|BIOUG65686-G09|BOLD:ACA3148
Sciaridae|GMPJA3028-21|BIOUG65686-G11|BOLD:ACA3148
Sciaridae|GMPJA3050-21|BIOUG65687-A10|BOLD:ACA3148
Sciaridae|GMPJA3056-21|BIOUG65687-B04|BOLD:ACA3148
Sciaridae|GMPJA3064-21|BIOUG65687-B12|BOLD:ACA3148
Sciaridae|GMPJA3130-21|BIOUG65687-H06|BOLD:ACA3148
Sciaridae|GMPJA3241-21|BIOUG65689-A11|BOLD:ACA3148
Sciaridae|GMPJA3245-21|BIOUG65689-B03|BOLD:ACA3148
Sciaridae|GMPJA3253-21|BIOUG65689-B11|BOLD:ACA3148
Sciaridae|GMPJA3332-21|BIOUG65690-A07|BOLD:ACA3148
Sciaridae|GMPJA3351-21|BIOUG65690-C02|BOLD:ACA3148
Sciaridae|GMPJA3475-21|BIOUG65691-E07|BOLD:ACA3148
Sciaridae|GMPJA3488-21|BIOUG65691-F08|BOLD:ACA3148
Sciaridae|GMPJA3496-21|BIOUG65691-G04|BOLD:ACA3148
Sciaridae|GMPJA3511-21|BIOUG65691-H07|BOLD:ACA3148
Sciaridae|GMPJA4302-21|BIOUG65700-C03|BOLD:ACA3148
Sciaridae|GMPJA3518-21|BIOUG65692-A03|BOLD:ACA3148
Sciaridae|GMPJA3534-21|BIOUG65692-B07|BOLD:ACA3148
Sciaridae|GMPJA3536-21|BIOUG65692-B09|BOLD:ACA3148
Sciaridae|GMPJA3537-21|BIOUG65692-B10|BOLD:ACA3148
Sciaridae|GMPJA3542-21|BIOUG65692-C03|BOLD:ACA3148
Sciaridae|GMPJA3580-21|BIOUG65692-F05|BOLD:ACA3148
Sciaridae|GMPJA3583-21|BIOUG65692-F08|BOLD:ACA3148
Sciaridae|GMPJA3592-21|BIOUG65692-G05|BOLD:ACA3148
Sciaridae|GMPJA3604-21|BIOUG65692-H05|BOLD:ACA3148
Sciaridae|GMPJA3612-21|BIOUG65693-A02|BOLD:ACA3148
Sciaridae|GMPJA3618-21|BIOUG65693-A08|BOLD:ACA3148
Sciaridae|GMPJA3630-21|BIOUG65693-B08|BOLD:ACA3148
Sciaridae|GMPJA3631-21|BIOUG65693-B09|BOLD:ACA3148
Sciaridae|GMPJA3929-21|BIOUG65696-C10|BOLD:ACA3148
Sciaridae|GMPJA4024-21|BIOUG65697-C10|BOLD:ACA3148
Sciaridae|GMPJA4033-21|BIOUG65697-D07|BOLD:ACA3148
Sciaridae|GMPJA4176-21|BIOUG65698-H07|BOLD:ACA3148
Sciaridae|GMPJA942-21|BIOUG65665-D02|BOLD:ACA3148
Sciaridae|GMPJA1288-21|BIOUG65669-A04|BOLD:ACA3148
Sciaridae|GMPJA1289-21|BIOUG65669-A05|BOLD:ACA3148
Sciaridae|GMPJA1294-21|BIOUG65669-A10|BOLD:ACA3148
Sciaridae|GMPJA1322-21|BIOUG65669-D02|BOLD:ACA3148
Sciaridae|GMPJA1326-21|BIOUG65669-D06|BOLD:ACA3148
Sciaridae|GMPJA1331-21|BIOUG65669-D11|BOLD:ACA3148
Sciaridae|GMPJA1063-21|BIOUG65666-F04|BOLD:ACA3148
Sciaridae|GMPJA1090-21|BIOUG65666-H07|BOLD:ACA3148
Sciaridae|GMPJA1102-21|BIOUG65667-A08|BOLD:ACA3148
Sciaridae|GMPJA1174-21|BIOUG65667-G08|BOLD:ACA3148
Sciaridae|GMPJA1334-21|BIOUG65669-E02|BOLD:ACA3148
Sciaridae|GMPJA1340-21|BIOUG65669-E08|BOLD:ACA3148
Sciaridae|GMPJA1342-21|BIOUG65669-E10|BOLD:ACA3148
Sciaridae|GMPJA1354-21|BIOUG65669-F10|BOLD:ACA3148
Sciaridae|GMPJA1389-21|BIOUG65670-A10|BOLD:ACA3148
Sciaridae|GMPJA1400-21|BIOUG65670-B09|BOLD:ACA3148
Sciaridae|GMPJA1412-21|BIOUG65670-C09|BOLD:ACA3148
Sciaridae|GMPJA1797-21|BIOUG65674-D02|BOLD:ACA3148
Sciaridae|GMPJA1806-21|BIOUG65674-D11|BOLD:ACA3148
Sciaridae|GMPJA1810-21|BIOUG65674-E03|BOLD:ACA3148
Sciaridae|GMPJA1830-21|BIOUG65674-F11|BOLD:ACA3148
Sciaridae|GMPJA1837-21|BIOUG65674-G06|BOLD:ACA3148
Sciaridae|GMPJA1844-21|BIOUG65674-H01|BOLD:ACA3148
Sciaridae|GMPJA1845-21|BIOUG65674-H02|BOLD:ACA3148
Sciaridae|GMPJA1852-21|BIOUG65674-H09|BOLD:ACA3148
Sciaridae|GMPJA1860-21|BIOUG65675-A06|BOLD:ACA3148

Sciaridae|GMPJA1845-21|BIOUG65674-H02|BOLD:ACA3148
Sciaridae|GMPJA1852-21|BIOUG65674-H09|BOLD:ACA3148
Sciaridae|GMPJA1860-21|BIOUG65675-A06|BOLD:ACA3148
Sciaridae|GMPJA1894-21|BIOUG65675-D04|BOLD:ACA3148
Sciaridae|GMPJA1905-21|BIOUG65675-E03|BOLD:ACA3148
Sciaridae|GMPJA1906-21|BIOUG65675-E04|BOLD:ACA3148
Sciaridae|GMPJA1930-21|BIOUG65675-G04|BOLD:ACA3148
Sciaridae|GMPJA1430-21|BIOUG65670-E03|BOLD:ACA3148
Sciaridae|GMPJA1466-21|BIOUG65670-H03|BOLD:ACA3148
Sciaridae|GMPJA1473-21|BIOUG65670-H10|BOLD:ACA3148
Sciaridae|GMPJA1479-21|BIOUG65671-A05|BOLD:ACA3148
Sciaridae|GMPJA1497-21|BIOUG65671-B11|BOLD:ACA3148
Sciaridae|GMPJA1503-21|BIOUG65671-C05|BOLD:ACA3148
Sciaridae|GMPJA1534-21|BIOUG65671-E12|BOLD:ACA3148
Sciaridae|GMPJA1545-21|BIOUG65671-F11|BOLD:ACA3148
Sciaridae|GMPJA1554-21|BIOUG65671-G08|BOLD:ACA3148
Sciaridae|GMPJA1571-21|BIOUG65672-A02|BOLD:ACA3148
Sciaridae|GMPJA1586-21|BIOUG65672-B05|BOLD:ACA3148
Sciaridae|GMPJA1588-21|BIOUG65672-B07|BOLD:ACA3148
Sciaridae|GMPJA1598-21|BIOUG65672-C05|BOLD:ACA3148
Sciaridae|GMPJA1607-21|BIOUG65672-D02|BOLD:ACA3148
Sciaridae|GMPJA1615-21|BIOUG65672-D10|BOLD:ACA3148
Sciaridae|GMPJA1637-21|BIOUG65672-F08|BOLD:ACA3148
Sciaridae|GMPJA1638-21|BIOUG65672-F09|BOLD:ACA3148
Sciaridae|GMPJA1650-21|BIOUG65672-G09|BOLD:ACA3148
Sciaridae|GMPJA2168-21|BIOUG65678-C05|BOLD:ACA3148
Sciaridae|GMPJA2177-21|BIOUG65678-D02|BOLD:ACA3148
Sciaridae|GMPJA2188-21|BIOUG65678-E01|BOLD:ACA3148
Sciaridae|GMPJA2191-21|BIOUG65678-E04|BOLD:ACA3148
Sciaridae|GMPJA2208-21|BIOUG65678-F09|BOLD:ACA3148
Sciaridae|GMPJA2215-21|BIOUG65678-G04|BOLD:ACA3148
Sciaridae|GMPJA2242-21|BIOUG65679-A08|BOLD:ACA3148
Sciaridae|GMPJA2243-21|BIOUG65679-A09|BOLD:ACA3148
Sciaridae|GMPJA2245-21|BIOUG65679-A11|BOLD:ACA3148
Sciaridae|GMPJA2254-21|BIOUG65679-B08|BOLD:ACA3148
Sciaridae|GMPJA2273-21|BIOUG65679-D03|BOLD:ACA3148
Sciaridae|GMPJA2293-21|BIOUG65679-E11|BOLD:ACA3148
Sciaridae|GMPJA2315-21|BIOUG65679-G09|BOLD:ACA3148
Sciaridae|GMPJA2322-21|BIOUG65679-H04|BOLD:ACA3148
Sciaridae|GMPJA2351-21|BIOUG65680-B10|BOLD:ACA3148
Sciaridae|GMPJA2361-21|BIOUG65680-C08|BOLD:ACA3148
Sciaridae|GMPJA1666-21|BIOUG65673-A02|BOLD:ACA3148
Sciaridae|GMPJA1683-21|BIOUG65673-B07|BOLD:ACA3148
Sciaridae|GMPJA1684-21|BIOUG65673-B08|BOLD:ACA3148
Sciaridae|GMPJA1687-21|BIOUG65673-B11|BOLD:ACA3148
Sciaridae|GMPJA1705-21|BIOUG65673-D05|BOLD:ACA3148
Sciaridae|GMPJA1715-21|BIOUG65673-E03|BOLD:ACA3148
Sciaridae|GMPJA1716-21|BIOUG65673-E04|BOLD:ACA3148
Sciaridae|GMPJA1717-21|BIOUG65673-E05|BOLD:ACA3148
Sciaridae|GMPJA1729-21|BIOUG65673-F05|BOLD:ACA3148
Sciaridae|GMPJA1743-21|BIOUG65673-G07|BOLD:ACA3148
Sciaridae|GMPJA1745-21|BIOUG65673-G09|BOLD:ACA3148
Sciaridae|GMPJA1750-21|BIOUG65673-H02|BOLD:ACA3148
Sciaridae|GMPJA1752-21|BIOUG65673-H04|BOLD:ACA3148
Sciaridae|GMPJA1764-21|BIOUG65674-A05|BOLD:ACA3148
Sciaridae|GMPJA1941-21|BIOUG65675-H03|BOLD:ACA3148
Sciaridae|GMPJA1964-21|BIOUG65676-B03|BOLD:ACA3148
Sciaridae|GMPJA1981-21|BIOUG65676-C08|BOLD:ACA3148
Sciaridae|GMPJA1982-21|BIOUG65676-C09|BOLD:ACA3148
Sciaridae|GMPJA1988-21|BIOUG65676-D03|BOLD:ACA3148
Sciaridae|GMPJA1989-21|BIOUG65676-D04|BOLD:ACA3148
Sciaridae|GMPJA1992-21|BIOUG65676-D07|BOLD:ACA3148
Sciaridae|GMPJA1994-21|BIOUG65676-D09|BOLD:ACA3148
Sciaridae|GMPJA2000-21|BIOUG65676-E03|BOLD:ACA3148
Sciaridae|GMPJA2033-21|BIOUG65676-G12|BOLD:ACA3148
Sciaridae|GMPJA2035-21|BIOUG65676-H02|BOLD:ACA3148
Sciaridae|GMPJA2036-21|BIOUG65676-H03|BOLD:ACA3148
Sciaridae|GMPJA2046-21|BIOUG65677-A02|BOLD:ACA3148
Sciaridae|GMPJA2070-21|BIOUG65677-C02|BOLD:ACA3148
Sciaridae|GMPJA2071-21|BIOUG65677-C03|BOLD:ACA3148
Sciaridae|GMPJA2099-21|BIOUG65677-E07|BOLD:ACA3148
Sciaridae|GMPJA2107-21|BIOUG65677-F03|BOLD:ACA3148
Sciaridae|GMPJA2112-21|BIOUG65677-F08|BOLD:ACA3148
Sciaridae|GMPJA2134-21|BIOUG65677-H06|BOLD:ACA3148
Sciaridae|GMPJA2141-21|BIOUG65678-A02|BOLD:ACA3148
Sciaridae|GMPJA2147-21|BIOUG65678-A08|BOLD:ACA3148
Sciaridae|GMPJA2148-21|BIOUG65678-A09|BOLD:ACA3148
Sciaridae|GMPJA2152-21|BIOUG65678-B01|BOLD:ACA3148
Sciaridae|GMPJA2160-21|BIOUG65678-B09|BOLD:ACA3148
Sciaridae|GMPJA2411-21|BIOUG65680-G10|BOLD:ACA3148
Sciaridae|GMPJA2418-21|BIOUG65680-H05|BOLD:ACA3148
Sciaridae|GMPJA3275-21|BIOUG65689-D09|BOLD:ACA3148
Sciaridae|GMPJA1361-21|BIOUG65669-G05|BOLD:ACA3148
Sciaridae|GMPJA1900-21|BIOUG65675-D10|BOLD:ACA3148
Sciaridae|GMPJA2332-21|BIOUG65680-A03|BOLD:ACA3148
Sciaridae|GMPJA1940-21|BIOUG65675-H02|BOLD:ACA3148
Sciaridae|GMPJA2084-21|BIOUG65677-D04|BOLD:ACA3148
Sciaridae|GMPJA3555-21|BIOUG65692-D04|BOLD:ACA3148
Sciaridae|GMPJA2384-21|BIOUG65680-E07|BOLD:ACA3148
Sciaridae|GMPJA4191-21|BIOUG65699-A11|BOLD:ACA3148
Sciaridae|GMPJA4414-21|BIOUG66601-D08|BOLD:ACA3148
Sciaridae|GMPJA3087-21|BIOUG65687-D11|BOLD:ACA3148
Sciaridae|GMPJA2447-21|BIOUG65681-B11|BOLD:ACA3148
Sciaridae|GMPJA2443-21|BIOUG65681-B07|BOLD:ACA3148
Sciaridae|GMPJA2426-21|BIOUG65681-A02|BOLD:ACA3148
Cecidomyiidae|GMPJA723-21|BIOUG65663-A09|BOLD:ABW5482
Cecidomyiidae|GMPJA682-21|BIOUG65662-F03|BOLD:ABW5482
Cecidomyiidae|GMPJA503-21|BIOUG65660-G02|BOLD:AEJ1491
Oligota parva|GMPJA8834-21|BIOUG66646-H10|BOLD:AAP9955
Cecidomyiidae|GMPJA10082-21|BIOUG66659-F02|BOLD:AEJ8146
Cecidomyiidae|GMPJA8436-21|BIOUG66642-G04|BOLD:AEJ8146
Cecidomyiidae|GMPJA7899-21|BIOUG66637-B02|BOLD:AEJ8146
Cecidomyiidae|GMPJA7616-21|BIOUG66634-B04|BOLD:AEJ8146

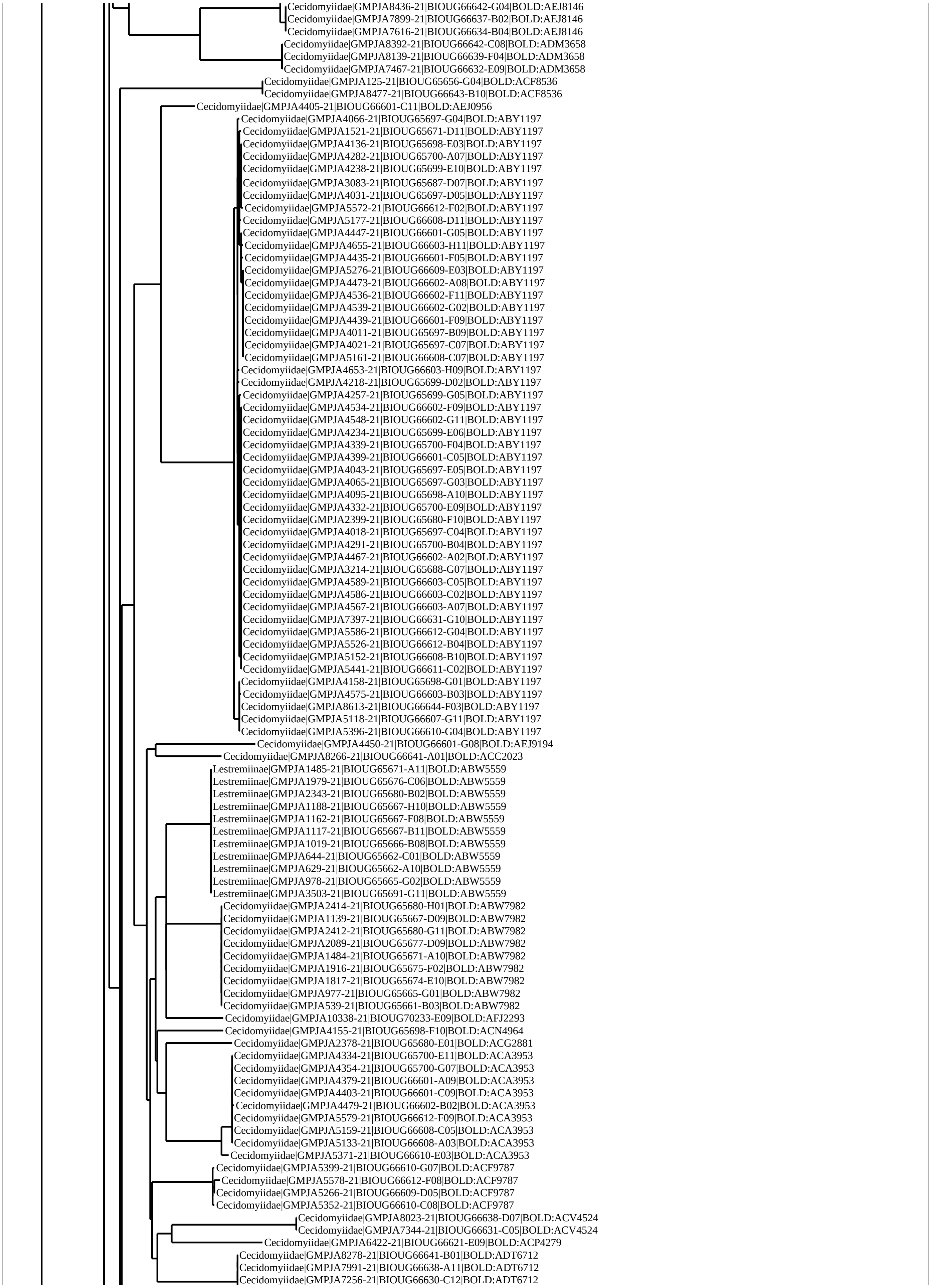
Cecidomyiidae|GMPJA8436-21|BIOUG66642-G04|BOLD:AEJ8146
Cecidomyiidae|GMPJA7899-21|BIOUG66637-B02|BOLD:AEJ8146
Cecidomyiidae|GMPJA7616-21|BIOUG66634-B04|BOLD:AEJ8146
Cecidomyiidae|GMPJA8392-21|BIOUG66642-C08|BOLD:ADM3658
Cecidomyiidae|GMPJA8139-21|BIOUG66639-F04|BOLD:ADM3658
Cecidomyiidae|GMPJA7467-21|BIOUG66632-E09|BOLD:ADM3658
Cecidomyiidae|GMPJA125-21|BIOUG65656-G04|BOLD:ACF8536
Cecidomyiidae|GMPJA8477-21|BIOUG66643-B10|BOLD:ACF8536
Cecidomyiidae|GMPJA4405-21|BIOUG66601-C11|BOLD:AEJ0956
Cecidomyiidae|GMPJA4066-21|BIOUG65697-G04|BOLD:ABY1197
Cecidomyiidae|GMPJA1521-21|BIOUG65671-D11|BOLD:ABY1197
Cecidomyiidae|GMPJA4136-21|BIOUG65698-E03|BOLD:ABY1197
Cecidomyiidae|GMPJA4282-21|BIOUG65700-A07|BOLD:ABY1197
Cecidomyiidae|GMPJA4238-21|BIOUG65699-E10|BOLD:ABY1197
Cecidomyiidae|GMPJA3083-21|BIOUG65687-D07|BOLD:ABY1197
Cecidomyiidae|GMPJA4031-21|BIOUG65697-D05|BOLD:ABY1197
Cecidomyiidae|GMPJA5572-21|BIOUG66612-F02|BOLD:ABY1197
Cecidomyiidae|GMPJA5177-21|BIOUG66608-D11|BOLD:ABY1197
Cecidomyiidae|GMPJA4447-21|BIOUG66601-G05|BOLD:ABY1197
Cecidomyiidae|GMPJA4655-21|BIOUG66603-H11|BOLD:ABY1197
Cecidomyiidae|GMPJA4435-21|BIOUG66601-F05|BOLD:ABY1197
Cecidomyiidae|GMPJA5276-21|BIOUG66609-E03|BOLD:ABY1197
Cecidomyiidae|GMPJA4473-21|BIOUG66602-A08|BOLD:ABY1197
Cecidomyiidae|GMPJA4536-21|BIOUG66602-F11|BOLD:ABY1197
Cecidomyiidae|GMPJA4539-21|BIOUG66602-G02|BOLD:ABY1197
Cecidomyiidae|GMPJA4439-21|BIOUG66601-F09|BOLD:ABY1197
Cecidomyiidae|GMPJA4011-21|BIOUG65697-B09|BOLD:ABY1197
Cecidomyiidae|GMPJA4021-21|BIOUG65697-C07|BOLD:ABY1197
Cecidomyiidae|GMPJA5161-21|BIOUG66608-C07|BOLD:ABY1197
Cecidomyiidae|GMPJA4653-21|BIOUG66603-H09|BOLD:ABY1197
Cecidomyiidae|GMPJA4218-21|BIOUG65699-D02|BOLD:ABY1197
Cecidomyiidae|GMPJA4257-21|BIOUG65699-G05|BOLD:ABY1197
Cecidomyiidae|GMPJA4534-21|BIOUG66602-F09|BOLD:ABY1197
Cecidomyiidae|GMPJA4548-21|BIOUG66602-G11|BOLD:ABY1197
Cecidomyiidae|GMPJA4234-21|BIOUG65699-E06|BOLD:ABY1197
Cecidomyiidae|GMPJA4339-21|BIOUG65700-F04|BOLD:ABY1197
Cecidomyiidae|GMPJA4399-21|BIOUG66601-C05|BOLD:ABY1197
Cecidomyiidae|GMPJA4043-21|BIOUG65697-E05|BOLD:ABY1197
Cecidomyiidae|GMPJA4065-21|BIOUG65697-G03|BOLD:ABY1197
Cecidomyiidae|GMPJA4095-21|BIOUG65698-A10|BOLD:ABY1197
Cecidomyiidae|GMPJA4332-21|BIOUG65700-E09|BOLD:ABY1197
Cecidomyiidae|GMPJA2399-21|BIOUG65680-F10|BOLD:ABY1197
Cecidomyiidae|GMPJA4018-21|BIOUG65697-C04|BOLD:ABY1197
Cecidomyiidae|GMPJA4291-21|BIOUG65700-B04|BOLD:ABY1197
Cecidomyiidae|GMPJA4467-21|BIOUG66602-A02|BOLD:ABY1197
Cecidomyiidae|GMPJA3214-21|BIOUG65688-G07|BOLD:ABY1197
Cecidomyiidae|GMPJA4589-21|BIOUG66603-C05|BOLD:ABY1197
Cecidomyiidae|GMPJA4586-21|BIOUG66603-C02|BOLD:ABY1197
Cecidomyiidae|GMPJA4567-21|BIOUG66603-A07|BOLD:ABY1197
Cecidomyiidae|GMPJA7397-21|BIOUG66631-G10|BOLD:ABY1197
Cecidomyiidae|GMPJA5586-21|BIOUG66612-G04|BOLD:ABY1197
Cecidomyiidae|GMPJA5526-21|BIOUG66612-B04|BOLD:ABY1197
Cecidomyiidae|GMPJA5152-21|BIOUG66608-B10|BOLD:ABY1197
Cecidomyiidae|GMPJA5441-21|BIOUG66611-C02|BOLD:ABY1197
Cecidomyiidae|GMPJA4158-21|BIOUG65698-G01|BOLD:ABY1197
Cecidomyiidae|GMPJA4575-21|BIOUG66603-B03|BOLD:ABY1197
Cecidomyiidae|GMPJA8613-21|BIOUG66644-F03|BOLD:ABY1197
Cecidomyiidae|GMPJA5118-21|BIOUG66607-G11|BOLD:ABY1197
Cecidomyiidae|GMPJA5396-21|BIOUG66610-G04|BOLD:ABY1197
Cecidomyiidae|GMPJA4450-21|BIOUG66601-G08|BOLD:AEJ9194
Cecidomyiidae|GMPJA8266-21|BIOUG66641-A01|BOLD:ACC2023
Lestremiinae|GMPJA1485-21|BIOUG65671-A11|BOLD:ABW5559
Lestremiinae|GMPJA1979-21|BIOUG65676-C06|BOLD:ABW5559
Lestremiinae|GMPJA2343-21|BIOUG65680-B02|BOLD:ABW5559
Lestremiinae|GMPJA1188-21|BIOUG65667-H10|BOLD:ABW5559
Lestremiinae|GMPJA1162-21|BIOUG65667-F08|BOLD:ABW5559
Lestremiinae|GMPJA1117-21|BIOUG65667-B11|BOLD:ABW5559
Lestremiinae|GMPJA1019-21|BIOUG65666-B08|BOLD:ABW5559
Lestremiinae|GMPJA644-21|BIOUG65662-C01|BOLD:ABW5559
Lestremiinae|GMPJA629-21|BIOUG65662-A10|BOLD:ABW5559
Lestremiinae|GMPJA978-21|BIOUG65665-G02|BOLD:ABW5559
Lestremiinae|GMPJA3503-21|BIOUG65691-G11|BOLD:ABW5559
Cecidomyiidae|GMPJA2414-21|BIOUG65680-H01|BOLD:ABW7982
Cecidomyiidae|GMPJA1139-21|BIOUG65667-D09|BOLD:ABW7982
Cecidomyiidae|GMPJA2412-21|BIOUG65680-G11|BOLD:ABW7982
Cecidomyiidae|GMPJA2089-21|BIOUG65677-D09|BOLD:ABW7982
Cecidomyiidae|GMPJA1484-21|BIOUG65671-A10|BOLD:ABW7982
Cecidomyiidae|GMPJA1916-21|BIOUG65675-F02|BOLD:ABW7982
Cecidomyiidae|GMPJA1817-21|BIOUG65674-E10|BOLD:ABW7982
Cecidomyiidae|GMPJA977-21|BIOUG65665-G01|BOLD:ABW7982
Cecidomyiidae|GMPJA539-21|BIOUG65661-B03|BOLD:ABW7982
Cecidomyiidae|GMPJA10338-21|BIOUG70233-E09|BOLD:AFJ2293
Cecidomyiidae|GMPJA4155-21|BIOUG65698-F10|BOLD:ACN4964
Cecidomyiidae|GMPJA2378-21|BIOUG65680-E01|BOLD:ACG2881
Cecidomyiidae|GMPJA4334-21|BIOUG65700-E11|BOLD:ACA3953
Cecidomyiidae|GMPJA4354-21|BIOUG65700-G07|BOLD:ACA3953
Cecidomyiidae|GMPJA4379-21|BIOUG66601-A09|BOLD:ACA3953
Cecidomyiidae|GMPJA4403-21|BIOUG66601-C09|BOLD:ACA3953
Cecidomyiidae|GMPJA4479-21|BIOUG66602-B02|BOLD:ACA3953
Cecidomyiidae|GMPJA5579-21|BIOUG66612-F09|BOLD:ACA3953
Cecidomyiidae|GMPJA5159-21|BIOUG66608-C05|BOLD:ACA3953
Cecidomyiidae|GMPJA5133-21|BIOUG66608-A03|BOLD:ACA3953
Cecidomyiidae|GMPJA5371-21|BIOUG66610-E03|BOLD:ACA3953
Cecidomyiidae|GMPJA5399-21|BIOUG66610-G07|BOLD:ACF9787
Cecidomyiidae|GMPJA5578-21|BIOUG66612-F08|BOLD:ACF9787
Cecidomyiidae|GMPJA5266-21|BIOUG66609-D05|BOLD:ACF9787
Cecidomyiidae|GMPJA5352-21|BIOUG66610-C08|BOLD:ACF9787
Cecidomyiidae|GMPJA8023-21|BIOUG66638-D07|BOLD:ACV4524
Cecidomyiidae|GMPJA7344-21|BIOUG66631-C05|BOLD:ACV4524
Cecidomyiidae|GMPJA6422-21|BIOUG66621-E09|BOLD:ACP4279
Cecidomyiidae|GMPJA8278-21|BIOUG66641-B01|BOLD:ADT6712
Cecidomyiidae|GMPJA7991-21|BIOUG66638-A11|BOLD:ADT6712
Cecidomyiidae|GMPJA7256-21|BIOUG66630-C12|BOLD:ADT6712

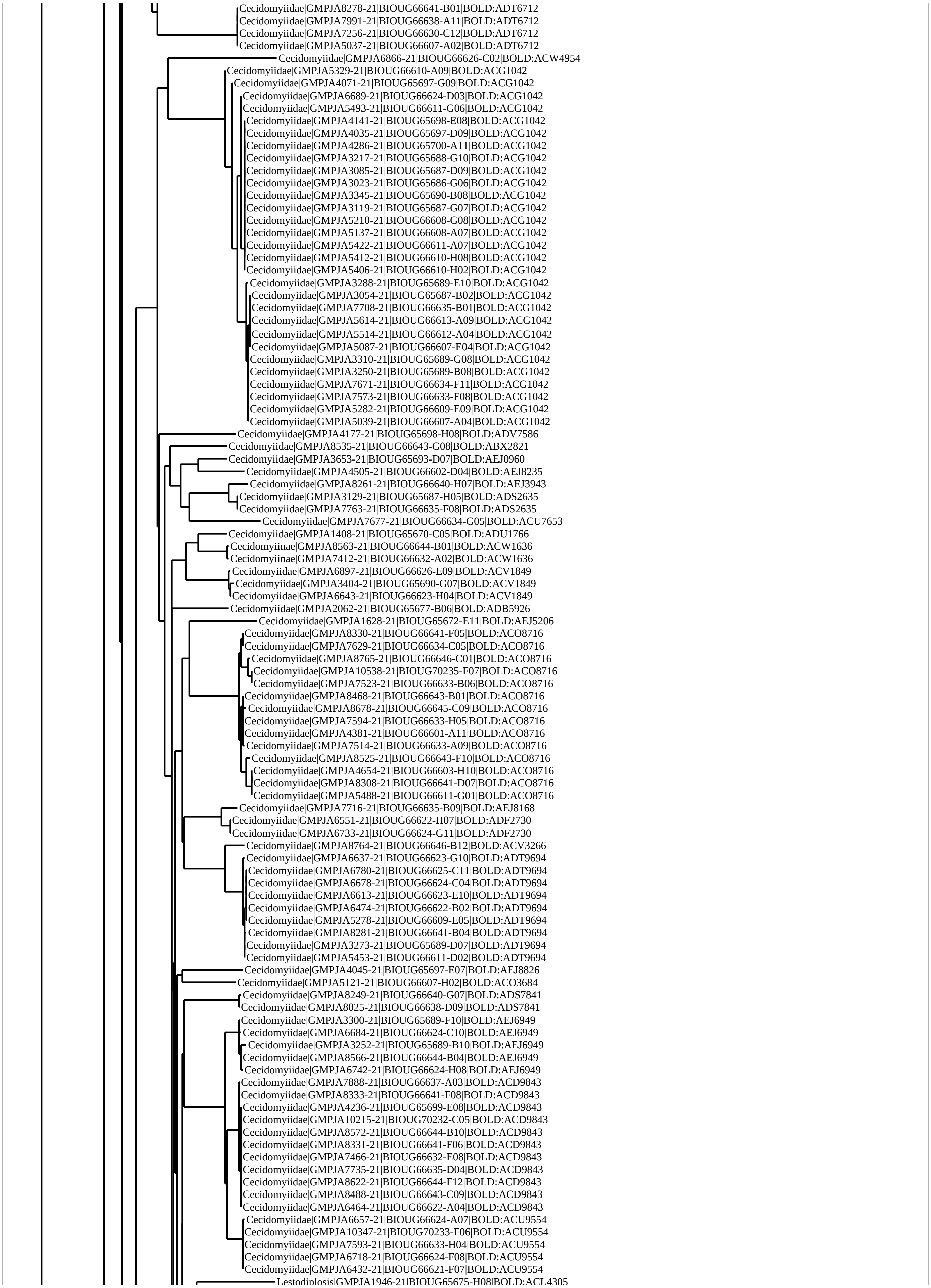

Cecidomyiidae|GMPJA8278-21|BIOUG66641-B01|BOLD:ADT6712
Cecidomyiidae|GMPJA7991-21|BIOUG66638-A11|BOLD:ADT6712
Cecidomyiidae|GMPJA7256-21|BIOUG66630-C12|BOLD:ADT6712
Cecidomyiidae|GMPJA5037-21|BIOUG66607-A02|BOLD:ADT6712
Cecidomyiidae|GMPJA6866-21|BIOUG66626-C02|BOLD:ACW4954
Cecidomyiidae|GMPJA5329-21|BIOUG66610-A09|BOLD:ACG1042
Cecidomyiidae|GMPJA4071-21|BIOUG65697-G09|BOLD:ACG1042
Cecidomyiidae|GMPJA6689-21|BIOUG66624-D03|BOLD:ACG1042
Cecidomyiidae|GMPJA5493-21|BIOUG66611-G06|BOLD:ACG1042
Cecidomyiidae|GMPJA4141-21|BIOUG65698-E08|BOLD:ACG1042
Cecidomyiidae|GMPJA4035-21|BIOUG65697-D09|BOLD:ACG1042
Cecidomyiidae|GMPJA4286-21|BIOUG65700-A11|BOLD:ACG1042
Cecidomyiidae|GMPJA3217-21|BIOUG65688-G10|BOLD:ACG1042
Cecidomyiidae|GMPJA3085-21|BIOUG65687-D09|BOLD:ACG1042
Cecidomyiidae|GMPJA3023-21|BIOUG65686-G06|BOLD:ACG1042
Cecidomyiidae|GMPJA3345-21|BIOUG65690-B08|BOLD:ACG1042
Cecidomyiidae|GMPJA3119-21|BIOUG65687-G07|BOLD:ACG1042
Cecidomyiidae|GMPJA5210-21|BIOUG66608-G08|BOLD:ACG1042
Cecidomyiidae|GMPJA5137-21|BIOUG66608-A07|BOLD:ACG1042
Cecidomyiidae|GMPJA5422-21|BIOUG66611-A07|BOLD:ACG1042
Cecidomyiidae|GMPJA5412-21|BIOUG66610-H08|BOLD:ACG1042
Cecidomyiidae|GMPJA5406-21|BIOUG66610-H02|BOLD:ACG1042
Cecidomyiidae|GMPJA3288-21|BIOUG65689-E10|BOLD:ACG1042
Cecidomyiidae|GMPJA3054-21|BIOUG65687-B02|BOLD:ACG1042
Cecidomyiidae|GMPJA7708-21|BIOUG66635-B01|BOLD:ACG1042
Cecidomyiidae|GMPJA5614-21|BIOUG66613-A09|BOLD:ACG1042
Cecidomyiidae|GMPJA5514-21|BIOUG66612-A04|BOLD:ACG1042
Cecidomyiidae|GMPJA5087-21|BIOUG66607-E04|BOLD:ACG1042
Cecidomyiidae|GMPJA3310-21|BIOUG65689-G08|BOLD:ACG1042
Cecidomyiidae|GMPJA3250-21|BIOUG65689-B08|BOLD:ACG1042
Cecidomyiidae|GMPJA7671-21|BIOUG66634-F11|BOLD:ACG1042
Cecidomyiidae|GMPJA7573-21|BIOUG66633-F08|BOLD:ACG1042
Cecidomyiidae|GMPJA5282-21|BIOUG66609-E09|BOLD:ACG1042
Cecidomyiidae|GMPJA5039-21|BIOUG66607-A04|BOLD:ACG1042
Cecidomyiidae|GMPJA4177-21|BIOUG65698-H08|BOLD:ADV7586
Cecidomyiidae|GMPJA8535-21|BIOUG66643-G08|BOLD:ABX2821
Cecidomyiidae|GMPJA3653-21|BIOUG65693-D07|BOLD:AEJ0960
Cecidomyiidae|GMPJA4505-21|BIOUG66602-D04|BOLD:AEJ8235
Cecidomyiidae|GMPJA8261-21|BIOUG66640-H07|BOLD:AEJ3943
Cecidomyiidae|GMPJA3129-21|BIOUG65687-H05|BOLD:ADS2635
Cecidomyiidae|GMPJA7763-21|BIOUG66635-F08|BOLD:ADS2635
Cecidomyiidae|GMPJA7677-21|BIOUG66634-G05|BOLD:ACU7653
Cecidomyiidae|GMPJA1408-21|BIOUG65670-C05|BOLD:ADU1766
Cecidomyiinae|GMPJA8563-21|BIOUG66644-B01|BOLD:ACW1636
Cecidomyiinae|GMPJA7412-21|BIOUG66632-A02|BOLD:ACW1636
Cecidomyiidae|GMPJA6897-21|BIOUG66626-E09|BOLD:ACV1849
Cecidomyiidae|GMPJA3404-21|BIOUG65690-G07|BOLD:ACV1849
Cecidomyiidae|GMPJA6643-21|BIOUG66623-H04|BOLD:ACV1849
Cecidomyiidae|GMPJA2062-21|BIOUG65677-B06|BOLD:ADB5926
Cecidomyiidae|GMPJA1628-21|BIOUG65672-E11|BOLD:AEJ5206
Cecidomyiidae|GMPJA8330-21|BIOUG66641-F05|BOLD:ACO8716
Cecidomyiidae|GMPJA7629-21|BIOUG66634-C05|BOLD:ACO8716
Cecidomyiidae|GMPJA8765-21|BIOUG66646-C01|BOLD:ACO8716
Cecidomyiidae|GMPJA10538-21|BIOUG70235-F07|BOLD:ACO8716
Cecidomyiidae|GMPJA7523-21|BIOUG66633-B06|BOLD:ACO8716
Cecidomyiidae|GMPJA8468-21|BIOUG66643-B01|BOLD:ACO8716
Cecidomyiidae|GMPJA8678-21|BIOUG66645-C09|BOLD:ACO8716
Cecidomyiidae|GMPJA7594-21|BIOUG66633-H05|BOLD:ACO8716
Cecidomyiidae|GMPJA4381-21|BIOUG66601-A11|BOLD:ACO8716
Cecidomyiidae|GMPJA7514-21|BIOUG66633-A09|BOLD:ACO8716
Cecidomyiidae|GMPJA8525-21|BIOUG66643-F10|BOLD:ACO8716
Cecidomyiidae|GMPJA4654-21|BIOUG66603-H10|BOLD:ACO8716
Cecidomyiidae|GMPJA8308-21|BIOUG66641-D07|BOLD:ACO8716
Cecidomyiidae|GMPJA5488-21|BIOUG66611-G01|BOLD:ACO8716
Cecidomyiidae|GMPJA7716-21|BIOUG66635-B09|BOLD:AEJ8168
Cecidomyiidae|GMPJA6551-21|BIOUG66622-H07|BOLD:ADF2730
Cecidomyiidae|GMPJA6733-21|BIOUG66624-G11|BOLD:ADF2730
Cecidomyiidae|GMPJA8764-21|BIOUG66646-B12|BOLD:ACV3266
Cecidomyiidae|GMPJA6637-21|BIOUG66623-G10|BOLD:ADT9694
Cecidomyiidae|GMPJA6780-21|BIOUG66625-C11|BOLD:ADT9694
Cecidomyiidae|GMPJA6678-21|BIOUG66624-C04|BOLD:ADT9694
Cecidomyiidae|GMPJA6613-21|BIOUG66623-E10|BOLD:ADT9694
Cecidomyiidae|GMPJA6474-21|BIOUG66622-B02|BOLD:ADT9694
Cecidomyiidae|GMPJA5278-21|BIOUG66609-E05|BOLD:ADT9694
Cecidomyiidae|GMPJA8281-21|BIOUG66641-B04|BOLD:ADT9694
Cecidomyiidae|GMPJA3273-21|BIOUG65689-D07|BOLD:ADT9694
Cecidomyiidae|GMPJA5453-21|BIOUG66611-D02|BOLD:ADT9694
Cecidomyiidae|GMPJA4045-21|BIOUG65697-E07|BOLD:AEJ8826
Cecidomyiidae|GMPJA5121-21|BIOUG66607-H02|BOLD:ACO3684
Cecidomyiidae|GMPJA8249-21|BIOUG66640-G07|BOLD:ADS7841
Cecidomyiidae|GMPJA8025-21|BIOUG66638-D09|BOLD:ADS7841
Cecidomyiidae|GMPJA3300-21|BIOUG65689-F10|BOLD:AEJ6949
Cecidomyiidae|GMPJA6684-21|BIOUG66624-C10|BOLD:AEJ6949
Cecidomyiidae|GMPJA3252-21|BIOUG65689-B10|BOLD:AEJ6949
Cecidomyiidae|GMPJA8566-21|BIOUG66644-B04|BOLD:AEJ6949
Cecidomyiidae|GMPJA6742-21|BIOUG66624-H08|BOLD:AEJ6949
Cecidomyiidae|GMPJA7888-21|BIOUG66637-A03|BOLD:ACD9843
Cecidomyiidae|GMPJA8333-21|BIOUG66641-F08|BOLD:ACD9843
Cecidomyiidae|GMPJA4236-21|BIOUG65699-E08|BOLD:ACD9843
Cecidomyiidae|GMPJA10215-21|BIOUG70232-C05|BOLD:ACD9843
Cecidomyiidae|GMPJA8572-21|BIOUG66644-B10|BOLD:ACD9843
Cecidomyiidae|GMPJA8331-21|BIOUG66641-F06|BOLD:ACD9843
Cecidomyiidae|GMPJA7466-21|BIOUG66632-E08|BOLD:ACD9843
Cecidomyiidae|GMPJA7735-21|BIOUG66635-D04|BOLD:ACD9843
Cecidomyiidae|GMPJA8622-21|BIOUG66644-F12|BOLD:ACD9843
Cecidomyiidae|GMPJA8488-21|BIOUG66643-C09|BOLD:ACD9843
Cecidomyiidae|GMPJA6464-21|BIOUG66622-A04|BOLD:ACD9843
Cecidomyiidae|GMPJA6657-21|BIOUG66624-A07|BOLD:ACU9554
Cecidomyiidae|GMPJA10347-21|BIOUG70233-F06|BOLD:ACU9554
Cecidomyiidae|GMPJA7593-21|BIOUG66633-H04|BOLD:ACU9554
Cecidomyiidae|GMPJA6718-21|BIOUG66624-F08|BOLD:ACU9554
Cecidomyiidae|GMPJA6432-21|BIOUG66621-F07|BOLD:ACU9554
Lestodiplosis|GMPJA1946-21|BIOUG65675-H08|BOLD:ACL4305

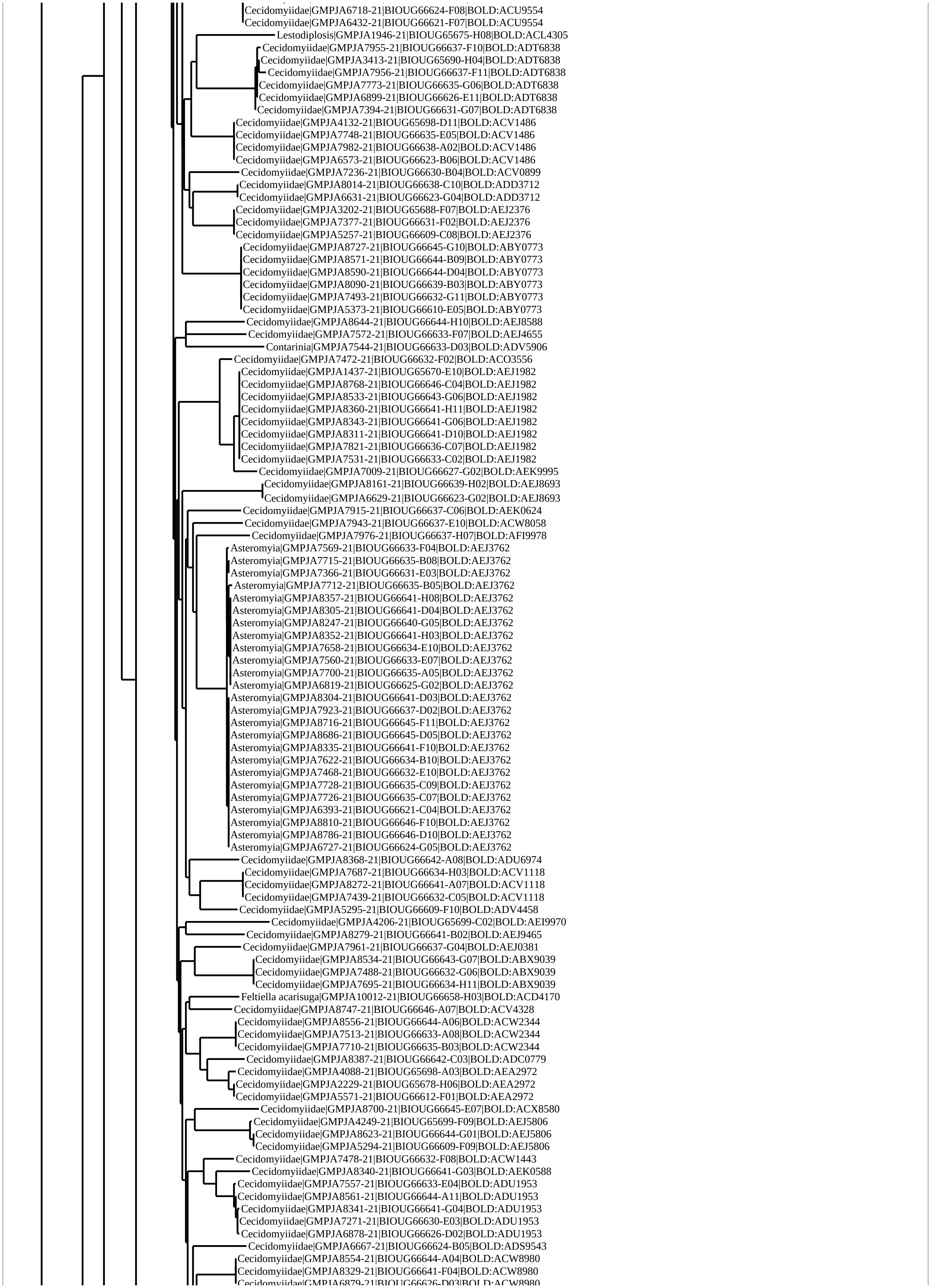
Cecidomyiidae|GMPJA6718-21|BIOUG66624-F08|BOLD:ACU9554
Cecidomyiidae|GMPJA6432-21|BIOUG66621-F07|BOLD:ACU9554
Lestodiplosis|GMPJA1946-21|BIOUG65675-H08|BOLD:ACL4305
Cecidomyiidae|GMPJA7955-21|BIOUG66637-F10|BOLD:ADT6838
Cecidomyiidae|GMPJA3413-21|BIOUG65690-H04|BOLD:ADT6838
Cecidomyiidae|GMPJA7956-21|BIOUG66637-F11|BOLD:ADT6838
Cecidomyiidae|GMPJA7773-21|BIOUG66635-G06|BOLD:ADT6838
Cecidomyiidae|GMPJA6899-21|BIOUG66626-E11|BOLD:ADT6838
Cecidomyiidae|GMPJA7394-21|BIOUG66631-G07|BOLD:ADT6838
Cecidomyiidae|GMPJA4132-21|BIOUG65698-D11|BOLD:ACV1486
Cecidomyiidae|GMPJA7748-21|BIOUG66635-E05|BOLD:ACV1486
Cecidomyiidae|GMPJA7982-21|BIOUG66638-A02|BOLD:ACV1486
Cecidomyiidae|GMPJA6573-21|BIOUG66623-B06|BOLD:ACV1486
Cecidomyiidae|GMPJA7236-21|BIOUG66630-B04|BOLD:ACV0899
Cecidomyiidae|GMPJA8014-21|BIOUG66638-C10|BOLD:ADD3712
Cecidomyiidae|GMPJA6631-21|BIOUG66623-G04|BOLD:ADD3712
Cecidomyiidae|GMPJA3202-21|BIOUG65688-F07|BOLD:AEJ2376
Cecidomyiidae|GMPJA7377-21|BIOUG66631-F02|BOLD:AEJ2376
Cecidomyiidae|GMPJA5257-21|BIOUG66609-C08|BOLD:AEJ2376
Cecidomyiidae|GMPJA8727-21|BIOUG66645-G10|BOLD:ABY0773
Cecidomyiidae|GMPJA8571-21|BIOUG66644-B09|BOLD:ABY0773
Cecidomyiidae|GMPJA8590-21|BIOUG66644-D04|BOLD:ABY0773
Cecidomyiidae|GMPJA8090-21|BIOUG66639-B03|BOLD:ABY0773
Cecidomyiidae|GMPJA7493-21|BIOUG66632-G11|BOLD:ABY0773
Cecidomyiidae|GMPJA5373-21|BIOUG66610-E05|BOLD:ABY0773
Cecidomyiidae|GMPJA8644-21|BIOUG66644-H10|BOLD:AEJ8588
Cecidomyiidae|GMPJA7572-21|BIOUG66633-F07|BOLD:AEJ4655
Contarinia|GMPJA7544-21|BIOUG66633-D03|BOLD:ADV5906
Cecidomyiidae|GMPJA7472-21|BIOUG66632-F02|BOLD:ACO3556
Cecidomyiidae|GMPJA1437-21|BIOUG65670-E10|BOLD:AEJ1982
Cecidomyiidae|GMPJA8768-21|BIOUG66646-C04|BOLD:AEJ1982
Cecidomyiidae|GMPJA8533-21|BIOUG66643-G06|BOLD:AEJ1982
Cecidomyiidae|GMPJA8360-21|BIOUG66641-H11|BOLD:AEJ1982
Cecidomyiidae|GMPJA8343-21|BIOUG66641-G06|BOLD:AEJ1982
Cecidomyiidae|GMPJA8311-21|BIOUG66641-D10|BOLD:AEJ1982
Cecidomyiidae|GMPJA7821-21|BIOUG66636-C07|BOLD:AEJ1982
Cecidomyiidae|GMPJA7531-21|BIOUG66633-C02|BOLD:AEJ1982
Cecidomyiidae|GMPJA7009-21|BIOUG66627-G02|BOLD:AEK9995
Cecidomyiidae|GMPJA8161-21|BIOUG66639-H02|BOLD:AEJ8693
Cecidomyiidae|GMPJA6629-21|BIOUG66623-G02|BOLD:AEJ8693
Cecidomyiidae|GMPJA7915-21|BIOUG66637-C06|BOLD:AEK0624
Cecidomyiidae|GMPJA7943-21|BIOUG66637-E10|BOLD:ACW8058
Cecidomyiidae|GMPJA7976-21|BIOUG66637-H07|BOLD:AFI9978
Asteromyia|GMPJA7569-21|BIOUG66633-F04|BOLD:AEJ3762
Asteromyia|GMPJA7715-21|BIOUG66635-B08|BOLD:AEJ3762
Asteromyia|GMPJA7366-21|BIOUG66631-E03|BOLD:AEJ3762
Asteromyia|GMPJA7712-21|BIOUG66635-B05|BOLD:AEJ3762
Asteromyia|GMPJA8357-21|BIOUG66641-H08|BOLD:AEJ3762
Asteromyia|GMPJA8305-21|BIOUG66641-D04|BOLD:AEJ3762
Asteromyia|GMPJA8247-21|BIOUG66640-G05|BOLD:AEJ3762
Asteromyia|GMPJA8352-21|BIOUG66641-H03|BOLD:AEJ3762
Asteromyia|GMPJA7658-21|BIOUG66634-E10|BOLD:AEJ3762
Asteromyia|GMPJA7560-21|BIOUG66633-E07|BOLD:AEJ3762
Asteromyia|GMPJA7700-21|BIOUG66635-A05|BOLD:AEJ3762
Asteromyia|GMPJA6819-21|BIOUG66625-G02|BOLD:AEJ3762
Asteromyia|GMPJA8304-21|BIOUG66641-D03|BOLD:AEJ3762
Asteromyia|GMPJA7923-21|BIOUG66637-D02|BOLD:AEJ3762
Asteromyia|GMPJA8716-21|BIOUG66645-F11|BOLD:AEJ3762
Asteromyia|GMPJA8686-21|BIOUG66645-D05|BOLD:AEJ3762
Asteromyia|GMPJA8335-21|BIOUG66641-F10|BOLD:AEJ3762
Asteromyia|GMPJA7622-21|BIOUG66634-B10|BOLD:AEJ3762
Asteromyia|GMPJA7468-21|BIOUG66632-E10|BOLD:AEJ3762
Asteromyia|GMPJA7728-21|BIOUG66635-C09|BOLD:AEJ3762
Asteromyia|GMPJA7726-21|BIOUG66635-C07|BOLD:AEJ3762
Asteromyia|GMPJA6393-21|BIOUG66621-C04|BOLD:AEJ3762
Asteromyia|GMPJA8810-21|BIOUG66646-F10|BOLD:AEJ3762
Asteromyia|GMPJA8786-21|BIOUG66646-D10|BOLD:AEJ3762
Asteromyia|GMPJA6727-21|BIOUG66624-G05|BOLD:AEJ3762
Cecidomyiidae|GMPJA8368-21|BIOUG66642-A08|BOLD:ADU6974
Cecidomyiidae|GMPJA7687-21|BIOUG66634-H03|BOLD:ACV1118
Cecidomyiidae|GMPJA8272-21|BIOUG66641-A07|BOLD:ACV1118
Cecidomyiidae|GMPJA7439-21|BIOUG66632-C05|BOLD:ACV1118
Cecidomyiidae|GMPJA5295-21|BIOUG66609-F10|BOLD:ADV4458
Cecidomyiidae|GMPJA4206-21|BIOUG65699-C02|BOLD:AEI9970
Cecidomyiidae|GMPJA8279-21|BIOUG66641-B02|BOLD:AEJ9465
Cecidomyiidae|GMPJA7961-21|BIOUG66637-G04|BOLD:AEJ0381
Cecidomyiidae|GMPJA8534-21|BIOUG66643-G07|BOLD:ABX9039
Cecidomyiidae|GMPJA7488-21|BIOUG66632-G06|BOLD:ABX9039
Cecidomyiidae|GMPJA7695-21|BIOUG66634-H11|BOLD:ABX9039
Feltiella acarisuga|GMPJA10012-21|BIOUG66658-H03|BOLD:ACD4170
Cecidomyiidae|GMPJA8747-21|BIOUG66646-A07|BOLD:ACV4328
Cecidomyiidae|GMPJA8556-21|BIOUG66644-A06|BOLD:ACW2344
Cecidomyiidae|GMPJA7513-21|BIOUG66633-A08|BOLD:ACW2344
Cecidomyiidae|GMPJA7710-21|BIOUG66635-B03|BOLD:ACW2344
Cecidomyiidae|GMPJA8387-21|BIOUG66642-C03|BOLD:ADC0779
Cecidomyiidae|GMPJA4088-21|BIOUG65698-A03|BOLD:AEA2972
Cecidomyiidae|GMPJA2229-21|BIOUG65678-H06|BOLD:AEA2972
Cecidomyiidae|GMPJA5571-21|BIOUG66612-F01|BOLD:AEA2972
Cecidomyiidae|GMPJA8700-21|BIOUG66645-E07|BOLD:ACX8580
Cecidomyiidae|GMPJA4249-21|BIOUG65699-F09|BOLD:AEJ5806
Cecidomyiidae|GMPJA8623-21|BIOUG66644-G01|BOLD:AEJ5806
Cecidomyiidae|GMPJA5294-21|BIOUG66609-F09|BOLD:AEJ5806
Cecidomyiidae|GMPJA7478-21|BIOUG66632-F08|BOLD:ACW1443
Cecidomyiidae|GMPJA8340-21|BIOUG66641-G03|BOLD:AEK0588
Cecidomyiidae|GMPJA7557-21|BIOUG66633-E04|BOLD:ADU1953
Cecidomyiidae|GMPJA8561-21|BIOUG66644-A11|BOLD:ADU1953
Cecidomyiidae|GMPJA8341-21|BIOUG66641-G04|BOLD:ADU1953
Cecidomyiidae|GMPJA7271-21|BIOUG66630-E03|BOLD:ADU1953
Cecidomyiidae|GMPJA6878-21|BIOUG66626-D02|BOLD:ADU1953
Cecidomyiidae|GMPJA6667-21|BIOUG66624-B05|BOLD:ADS9543
Cecidomyiidae|GMPJA8554-21|BIOUG66644-A04|BOLD:ACW8980
Cecidomyiidae|GMPJA8329-21|BIOUG66641-F04|BOLD:ACW8980
Cecidomyiidae|GMPJA6879-21|BIOUG66626-D03|BOLD:ACW8980

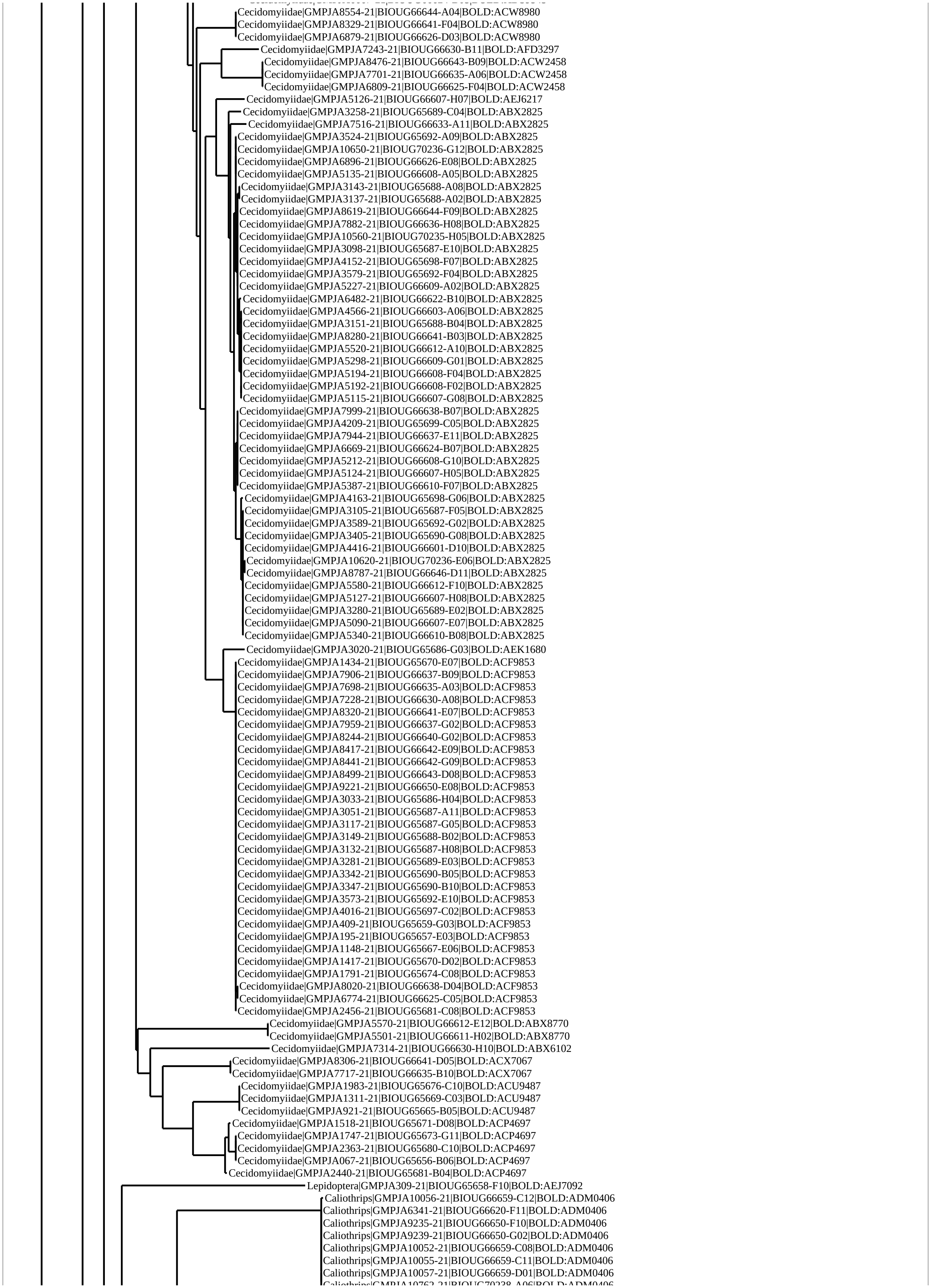

[illegible]
Cecidomyiidae|GMPJA8554-21|BIOUG66644-A04|BOLD:ACW8980
Cecidomyiidae|GMPJA8329-21|BIOUG66641-F04|BOLD:ACW8980
Cecidomyiidae|GMPJA6879-21|BIOUG66626-D03|BOLD:ACW8980
Cecidomyiidae|GMPJA7243-21|BIOUG66630-B11|BOLD:AFD3297
Cecidomyiidae|GMPJA8476-21|BIOUG66643-B09|BOLD:ACW2458
Cecidomyiidae|GMPJA7701-21|BIOUG66635-A06|BOLD:ACW2458
Cecidomyiidae|GMPJA6809-21|BIOUG66625-F04|BOLD:ACW2458
Cecidomyiidae|GMPJA5126-21|BIOUG66607-H07|BOLD:AEJ6217
Cecidomyiidae|GMPJA3258-21|BIOUG65689-C04|BOLD:ABX2825
Cecidomyiidae|GMPJA7516-21|BIOUG66633-A11|BOLD:ABX2825
Cecidomyiidae|GMPJA3524-21|BIOUG65692-A09|BOLD:ABX2825
Cecidomyiidae|GMPJA10650-21|BIOUG70236-G12|BOLD:ABX2825
Cecidomyiidae|GMPJA6896-21|BIOUG66626-E08|BOLD:ABX2825
Cecidomyiidae|GMPJA5135-21|BIOUG66608-A05|BOLD:ABX2825
Cecidomyiidae|GMPJA3143-21|BIOUG65688-A08|BOLD:ABX2825
Cecidomyiidae|GMPJA3137-21|BIOUG65688-A02|BOLD:ABX2825
Cecidomyiidae|GMPJA8619-21|BIOUG66644-F09|BOLD:ABX2825
Cecidomyiidae|GMPJA7882-21|BIOUG66636-H08|BOLD:ABX2825
Cecidomyiidae|GMPJA10560-21|BIOUG70235-H05|BOLD:ABX2825
Cecidomyiidae|GMPJA3098-21|BIOUG65687-E10|BOLD:ABX2825
Cecidomyiidae|GMPJA4152-21|BIOUG65698-F07|BOLD:ABX2825
Cecidomyiidae|GMPJA3579-21|BIOUG65692-F04|BOLD:ABX2825
Cecidomyiidae|GMPJA5227-21|BIOUG66609-A02|BOLD:ABX2825
Cecidomyiidae|GMPJA6482-21|BIOUG66622-B10|BOLD:ABX2825
Cecidomyiidae|GMPJA4566-21|BIOUG66603-A06|BOLD:ABX2825
Cecidomyiidae|GMPJA3151-21|BIOUG65688-B04|BOLD:ABX2825
Cecidomyiidae|GMPJA8280-21|BIOUG66641-B03|BOLD:ABX2825
Cecidomyiidae|GMPJA5520-21|BIOUG66612-A10|BOLD:ABX2825
Cecidomyiidae|GMPJA5298-21|BIOUG66609-G01|BOLD:ABX2825
Cecidomyiidae|GMPJA5194-21|BIOUG66608-F04|BOLD:ABX2825
Cecidomyiidae|GMPJA5192-21|BIOUG66608-F02|BOLD:ABX2825
Cecidomyiidae|GMPJA5115-21|BIOUG66607-G08|BOLD:ABX2825
Cecidomyiidae|GMPJA7999-21|BIOUG66638-B07|BOLD:ABX2825
Cecidomyiidae|GMPJA4209-21|BIOUG65699-C05|BOLD:ABX2825
Cecidomyiidae|GMPJA7944-21|BIOUG66637-E11|BOLD:ABX2825
Cecidomyiidae|GMPJA6669-21|BIOUG66624-B07|BOLD:ABX2825
Cecidomyiidae|GMPJA5212-21|BIOUG66608-G10|BOLD:ABX2825
Cecidomyiidae|GMPJA5124-21|BIOUG66607-H05|BOLD:ABX2825
Cecidomyiidae|GMPJA5387-21|BIOUG66610-F07|BOLD:ABX2825
Cecidomyiidae|GMPJA4163-21|BIOUG65698-G06|BOLD:ABX2825
Cecidomyiidae|GMPJA3105-21|BIOUG65687-F05|BOLD:ABX2825
Cecidomyiidae|GMPJA3589-21|BIOUG65692-G02|BOLD:ABX2825
Cecidomyiidae|GMPJA3405-21|BIOUG65690-G08|BOLD:ABX2825
Cecidomyiidae|GMPJA4416-21|BIOUG66601-D10|BOLD:ABX2825
Cecidomyiidae|GMPJA10620-21|BIOUG70236-E06|BOLD:ABX2825
Cecidomyiidae|GMPJA8787-21|BIOUG66646-D11|BOLD:ABX2825
Cecidomyiidae|GMPJA5580-21|BIOUG66612-F10|BOLD:ABX2825
Cecidomyiidae|GMPJA5127-21|BIOUG66607-H08|BOLD:ABX2825
Cecidomyiidae|GMPJA3280-21|BIOUG65689-E02|BOLD:ABX2825
Cecidomyiidae|GMPJA5090-21|BIOUG66607-E07|BOLD:ABX2825
Cecidomyiidae|GMPJA5340-21|BIOUG66610-B08|BOLD:ABX2825
Cecidomyiidae|GMPJA3020-21|BIOUG65686-G03|BOLD:AEK1680
Cecidomyiidae|GMPJA1434-21|BIOUG65670-E07|BOLD:ACF9853
Cecidomyiidae|GMPJA7906-21|BIOUG66637-B09|BOLD:ACF9853
Cecidomyiidae|GMPJA7698-21|BIOUG66635-A03|BOLD:ACF9853
Cecidomyiidae|GMPJA7228-21|BIOUG66630-A08|BOLD:ACF9853
Cecidomyiidae|GMPJA8320-21|BIOUG66641-E07|BOLD:ACF9853
Cecidomyiidae|GMPJA7959-21|BIOUG66637-G02|BOLD:ACF9853
Cecidomyiidae|GMPJA8244-21|BIOUG66640-G02|BOLD:ACF9853
Cecidomyiidae|GMPJA8417-21|BIOUG66642-E09|BOLD:ACF9853
Cecidomyiidae|GMPJA8441-21|BIOUG66642-G09|BOLD:ACF9853
Cecidomyiidae|GMPJA8499-21|BIOUG66643-D08|BOLD:ACF9853
Cecidomyiidae|GMPJA9221-21|BIOUG66650-E08|BOLD:ACF9853
Cecidomyiidae|GMPJA3033-21|BIOUG65686-H04|BOLD:ACF9853
Cecidomyiidae|GMPJA3051-21|BIOUG65687-A11|BOLD:ACF9853
Cecidomyiidae|GMPJA3117-21|BIOUG65687-G05|BOLD:ACF9853
Cecidomyiidae|GMPJA3149-21|BIOUG65688-B02|BOLD:ACF9853
Cecidomyiidae|GMPJA3132-21|BIOUG65687-H08|BOLD:ACF9853
Cecidomyiidae|GMPJA3281-21|BIOUG65689-E03|BOLD:ACF9853
Cecidomyiidae|GMPJA3342-21|BIOUG65690-B05|BOLD:ACF9853
Cecidomyiidae|GMPJA3347-21|BIOUG65690-B10|BOLD:ACF9853
Cecidomyiidae|GMPJA3573-21|BIOUG65692-E10|BOLD:ACF9853
Cecidomyiidae|GMPJA4016-21|BIOUG65697-C02|BOLD:ACF9853
Cecidomyiidae|GMPJA409-21|BIOUG65659-G03|BOLD:ACF9853
Cecidomyiidae|GMPJA195-21|BIOUG65657-E03|BOLD:ACF9853
Cecidomyiidae|GMPJA1148-21|BIOUG65667-E06|BOLD:ACF9853
Cecidomyiidae|GMPJA1417-21|BIOUG65670-D02|BOLD:ACF9853
Cecidomyiidae|GMPJA1791-21|BIOUG65674-C08|BOLD:ACF9853
Cecidomyiidae|GMPJA8020-21|BIOUG66638-D04|BOLD:ACF9853
Cecidomyiidae|GMPJA6774-21|BIOUG66625-C05|BOLD:ACF9853
Cecidomyiidae|GMPJA2456-21|BIOUG65681-C08|BOLD:ACF9853
Cecidomyiidae|GMPJA5570-21|BIOUG66612-E12|BOLD:ABX8770
Cecidomyiidae|GMPJA5501-21|BIOUG66611-H02|BOLD:ABX8770
Cecidomyiidae|GMPJA7314-21|BIOUG66630-H10|BOLD:ABX6102
Cecidomyiidae|GMPJA8306-21|BIOUG66641-D05|BOLD:ACX7067
Cecidomyiidae|GMPJA7717-21|BIOUG66635-B10|BOLD:ACX7067
Cecidomyiidae|GMPJA1983-21|BIOUG65676-C10|BOLD:ACU9487
Cecidomyiidae|GMPJA1311-21|BIOUG65669-C03|BOLD:ACU9487
Cecidomyiidae|GMPJA921-21|BIOUG65665-B05|BOLD:ACU9487
Cecidomyiidae|GMPJA1518-21|BIOUG65671-D08|BOLD:ACP4697
Cecidomyiidae|GMPJA1747-21|BIOUG65673-G11|BOLD:ACP4697
Cecidomyiidae|GMPJA2363-21|BIOUG65680-C10|BOLD:ACP4697
Cecidomyiidae|GMPJA067-21|BIOUG65656-B06|BOLD:ACP4697
Cecidomyiidae|GMPJA2440-21|BIOUG65681-B04|BOLD:ACP4697
Lepidoptera|GMPJA309-21|BIOUG65658-F10|BOLD:AEJ7092
Caliothrips|GMPJA10056-21|BIOUG66659-C12|BOLD:ADM0406
Caliothrips|GMPJA6341-21|BIOUG66620-F11|BOLD:ADM0406
Caliothrips|GMPJA9235-21|BIOUG66650-F10|BOLD:ADM0406
Caliothrips|GMPJA9239-21|BIOUG66650-G02|BOLD:ADM0406
Caliothrips|GMPJA10052-21|BIOUG66659-C08|BOLD:ADM0406
Caliothrips|GMPJA10055-21|BIOUG66659-C11|BOLD:ADM0406
Caliothrips|GMPJA10057-21|BIOUG66659-D01|BOLD:ADM0406
Caliothrips|GMPJA10762-21|BIOUG70238-A06|BOLD:ADM0406

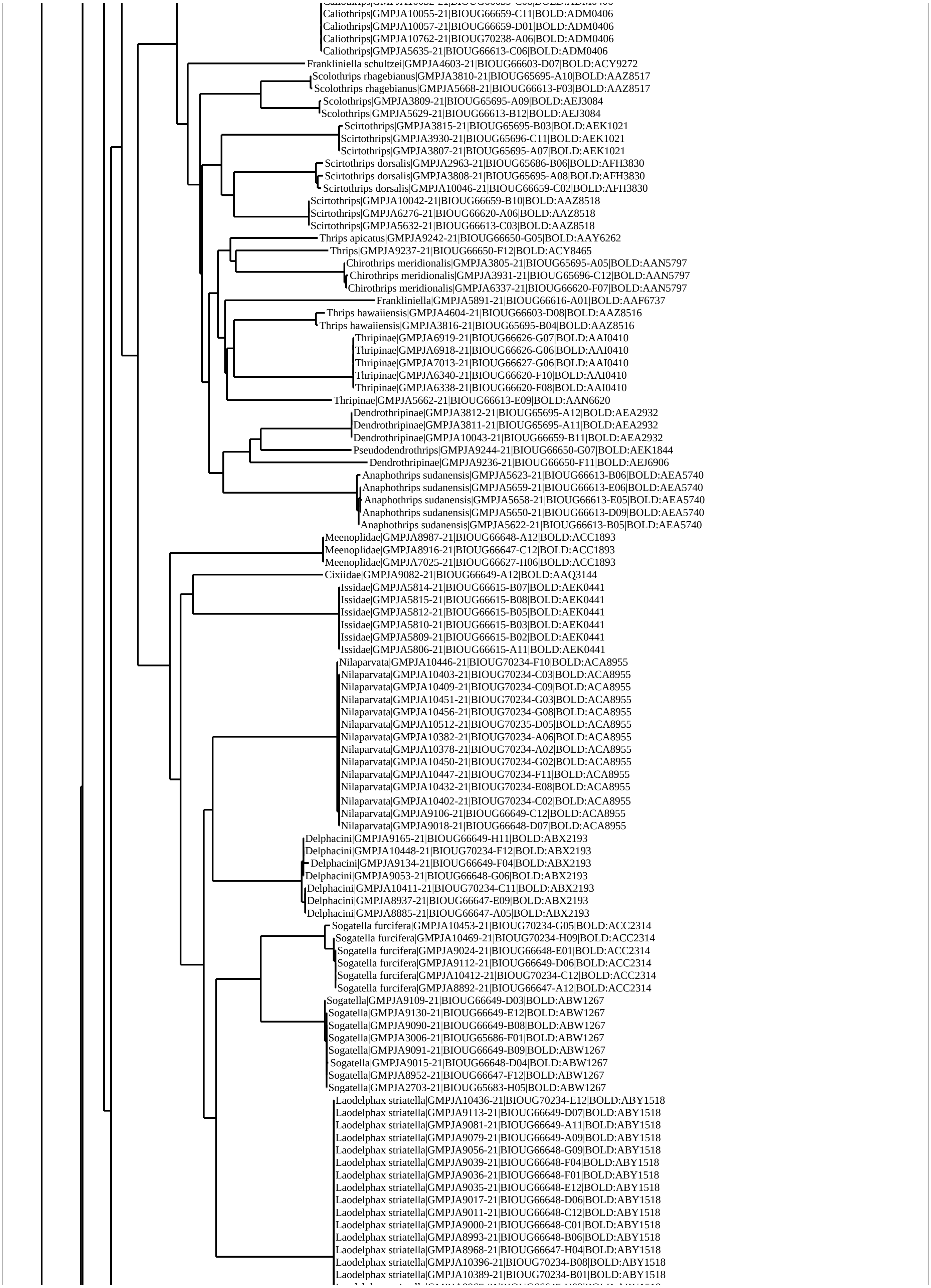
Caliothrips|GMPJA10055-21|BIOUG66659-C11|BOLD:ADM0406
Caliothrips|GMPJA10057-21|BIOUG66659-D01|BOLD:ADM0406
Caliothrips|GMPJA10762-21|BIOUG70238-A06|BOLD:ADM0406
Caliothrips|GMPJA5635-21|BIOUG66613-C06|BOLD:ADM0406
Frankliniella schultzei|GMPJA4603-21|BIOUG66603-D07|BOLD:ACY9272
Scolothrips rhagebianus|GMPJA3810-21|BIOUG65695-A10|BOLD:AAZ8517
Scolothrips rhagebianus|GMPJA5668-21|BIOUG66613-F03|BOLD:AAZ8517
Scolothrips|GMPJA3809-21|BIOUG65695-A09|BOLD:AEJ3084
Scolothrips|GMPJA5629-21|BIOUG66613-B12|BOLD:AEJ3084
Scirtothrips|GMPJA3815-21|BIOUG65695-B03|BOLD:AEK1021
Scirtothrips|GMPJA3930-21|BIOUG65696-C11|BOLD:AEK1021
Scirtothrips|GMPJA3807-21|BIOUG65695-A07|BOLD:AEK1021
Scirtothrips dorsalis|GMPJA2963-21|BIOUG65686-B06|BOLD:AFH3830
Scirtothrips dorsalis|GMPJA3808-21|BIOUG65695-A08|BOLD:AFH3830
Scirtothrips dorsalis|GMPJA10046-21|BIOUG66659-C02|BOLD:AFH3830
Scirtothrips|GMPJA10042-21|BIOUG66659-B10|BOLD:AAZ8518
Scirtothrips|GMPJA6276-21|BIOUG66620-A06|BOLD:AAZ8518
Scirtothrips|GMPJA5632-21|BIOUG66613-C03|BOLD:AAZ8518
Thrips apicatus|GMPJA9242-21|BIOUG66650-G05|BOLD:AAY6262
Thrips|GMPJA9237-21|BIOUG66650-F12|BOLD:ACY8465
Chirothrips meridionalis|GMPJA3805-21|BIOUG65695-A05|BOLD:AAN5797
Chirothrips meridionalis|GMPJA3931-21|BIOUG65696-C12|BOLD:AAN5797
Chirothrips meridionalis|GMPJA6337-21|BIOUG66620-F07|BOLD:AAN5797
Frankliniella|GMPJA5891-21|BIOUG66616-A01|BOLD:AAF6737
Thrips hawaiiensis|GMPJA4604-21|BIOUG66603-D08|BOLD:AAZ8516
Thrips hawaiiensis|GMPJA3816-21|BIOUG65695-B04|BOLD:AAZ8516
Thripinae|GMPJA6919-21|BIOUG66626-G07|BOLD:AAI0410
Thripinae|GMPJA6918-21|BIOUG66626-G06|BOLD:AAI0410
Thripinae|GMPJA7013-21|BIOUG66627-G06|BOLD:AAI0410
Thripinae|GMPJA6340-21|BIOUG66620-F10|BOLD:AAI0410
Thripinae|GMPJA6338-21|BIOUG66620-F08|BOLD:AAI0410
Thripinae|GMPJA5662-21|BIOUG66613-E09|BOLD:AAN6620
Dendrothripinae|GMPJA3812-21|BIOUG65695-A12|BOLD:AEA2932
Dendrothripinae|GMPJA3811-21|BIOUG65695-A11|BOLD:AEA2932
Dendrothripinae|GMPJA10043-21|BIOUG66659-B11|BOLD:AEA2932
Pseudodendrothrips|GMPJA9244-21|BIOUG66650-G07|BOLD:AEK1844
Dendrothripinae|GMPJA9236-21|BIOUG66650-F11|BOLD:AEJ6906
Anaphothrips sudanensis|GMPJA5623-21|BIOUG66613-B06|BOLD:AEA5740
Anaphothrips sudanensis|GMPJA5659-21|BIOUG66613-E06|BOLD:AEA5740
Anaphothrips sudanensis|GMPJA5658-21|BIOUG66613-E05|BOLD:AEA5740
Anaphothrips sudanensis|GMPJA5650-21|BIOUG66613-D09|BOLD:AEA5740
Anaphothrips sudanensis|GMPJA5622-21|BIOUG66613-B05|BOLD:AEA5740
Meenoplidae|GMPJA8987-21|BIOUG66648-A12|BOLD:ACC1893
Meenoplidae|GMPJA8916-21|BIOUG66647-C12|BOLD:ACC1893
Meenoplidae|GMPJA7025-21|BIOUG66627-H06|BOLD:ACC1893
Cixiidae|GMPJA9082-21|BIOUG66649-A12|BOLD:AAQ3144
Issidae|GMPJA5814-21|BIOUG66615-B07|BOLD:AEK0441
Issidae|GMPJA5815-21|BIOUG66615-B08|BOLD:AEK0441
Issidae|GMPJA5812-21|BIOUG66615-B05|BOLD:AEK0441
Issidae|GMPJA5810-21|BIOUG66615-B03|BOLD:AEK0441
Issidae|GMPJA5809-21|BIOUG66615-B02|BOLD:AEK0441
Issidae|GMPJA5806-21|BIOUG66615-A11|BOLD:AEK0441
Nilaparvata|GMPJA10446-21|BIOUG70234-F10|BOLD:ACA8955
Nilaparvata|GMPJA10403-21|BIOUG70234-C03|BOLD:ACA8955
Nilaparvata|GMPJA10409-21|BIOUG70234-C09|BOLD:ACA8955
Nilaparvata|GMPJA10451-21|BIOUG70234-G03|BOLD:ACA8955
Nilaparvata|GMPJA10456-21|BIOUG70234-G08|BOLD:ACA8955
Nilaparvata|GMPJA10512-21|BIOUG70235-D05|BOLD:ACA8955
Nilaparvata|GMPJA10382-21|BIOUG70234-A06|BOLD:ACA8955
Nilaparvata|GMPJA10378-21|BIOUG70234-A02|BOLD:ACA8955
Nilaparvata|GMPJA10450-21|BIOUG70234-G02|BOLD:ACA8955
Nilaparvata|GMPJA10447-21|BIOUG70234-F11|BOLD:ACA8955
Nilaparvata|GMPJA10432-21|BIOUG70234-E08|BOLD:ACA8955
Nilaparvata|GMPJA10402-21|BIOUG70234-C02|BOLD:ACA8955
Nilaparvata|GMPJA9106-21|BIOUG66649-C12|BOLD:ACA8955
Nilaparvata|GMPJA9018-21|BIOUG66648-D07|BOLD:ACA8955
Delphacini|GMPJA9165-21|BIOUG66649-H11|BOLD:ABX2193
Delphacini|GMPJA10448-21|BIOUG70234-F12|BOLD:ABX2193
Delphacini|GMPJA9134-21|BIOUG66649-F04|BOLD:ABX2193
Delphacini|GMPJA9053-21|BIOUG66648-G06|BOLD:ABX2193
Delphacini|GMPJA10411-21|BIOUG70234-C11|BOLD:ABX2193
Delphacini|GMPJA8937-21|BIOUG66647-E09|BOLD:ABX2193
Delphacini|GMPJA8885-21|BIOUG66647-A05|BOLD:ABX2193
Sogatella furcifera|GMPJA10453-21|BIOUG70234-G05|BOLD:ACC2314
Sogatella furcifera|GMPJA10469-21|BIOUG70234-H09|BOLD:ACC2314
Sogatella furcifera|GMPJA9024-21|BIOUG66648-E01|BOLD:ACC2314
Sogatella furcifera|GMPJA9112-21|BIOUG66649-D06|BOLD:ACC2314
Sogatella furcifera|GMPJA10412-21|BIOUG70234-C12|BOLD:ACC2314
Sogatella furcifera|GMPJA8892-21|BIOUG66647-A12|BOLD:ACC2314
Sogatella|GMPJA9109-21|BIOUG66649-D03|BOLD:ABW1267
Sogatella|GMPJA9130-21|BIOUG66649-E12|BOLD:ABW1267
Sogatella|GMPJA9090-21|BIOUG66649-B08|BOLD:ABW1267
Sogatella|GMPJA3006-21|BIOUG65686-F01|BOLD:ABW1267
Sogatella|GMPJA9091-21|BIOUG66649-B09|BOLD:ABW1267
Sogatella|GMPJA9015-21|BIOUG66648-D04|BOLD:ABW1267
Sogatella|GMPJA8952-21|BIOUG66647-F12|BOLD:ABW1267
Sogatella|GMPJA2703-21|BIOUG65683-H05|BOLD:ABW1267
Laodelphax striatella|GMPJA10436-21|BIOUG70234-E12|BOLD:ABY1518
Laodelphax striatella|GMPJA9113-21|BIOUG66649-D07|BOLD:ABY1518
Laodelphax striatella|GMPJA9081-21|BIOUG66649-A11|BOLD:ABY1518
Laodelphax striatella|GMPJA9079-21|BIOUG66649-A09|BOLD:ABY1518
Laodelphax striatella|GMPJA9056-21|BIOUG66648-G09|BOLD:ABY1518
Laodelphax striatella|GMPJA9039-21|BIOUG66648-F04|BOLD:ABY1518
Laodelphax striatella|GMPJA9036-21|BIOUG66648-F01|BOLD:ABY1518
Laodelphax striatella|GMPJA9035-21|BIOUG66648-E12|BOLD:ABY1518
Laodelphax striatella|GMPJA9017-21|BIOUG66648-D06|BOLD:ABY1518
Laodelphax striatella|GMPJA9011-21|BIOUG66648-C12|BOLD:ABY1518
Laodelphax striatella|GMPJA9000-21|BIOUG66648-C01|BOLD:ABY1518
Laodelphax striatella|GMPJA8993-21|BIOUG66648-B06|BOLD:ABY1518
Laodelphax striatella|GMPJA8968-21|BIOUG66647-H04|BOLD:ABY1518
Laodelphax striatella|GMPJA10396-21|BIOUG70234-B08|BOLD:ABY1518
Laodelphax striatella|GMPJA10389-21|BIOUG70234-B01|BOLD:ABY1518

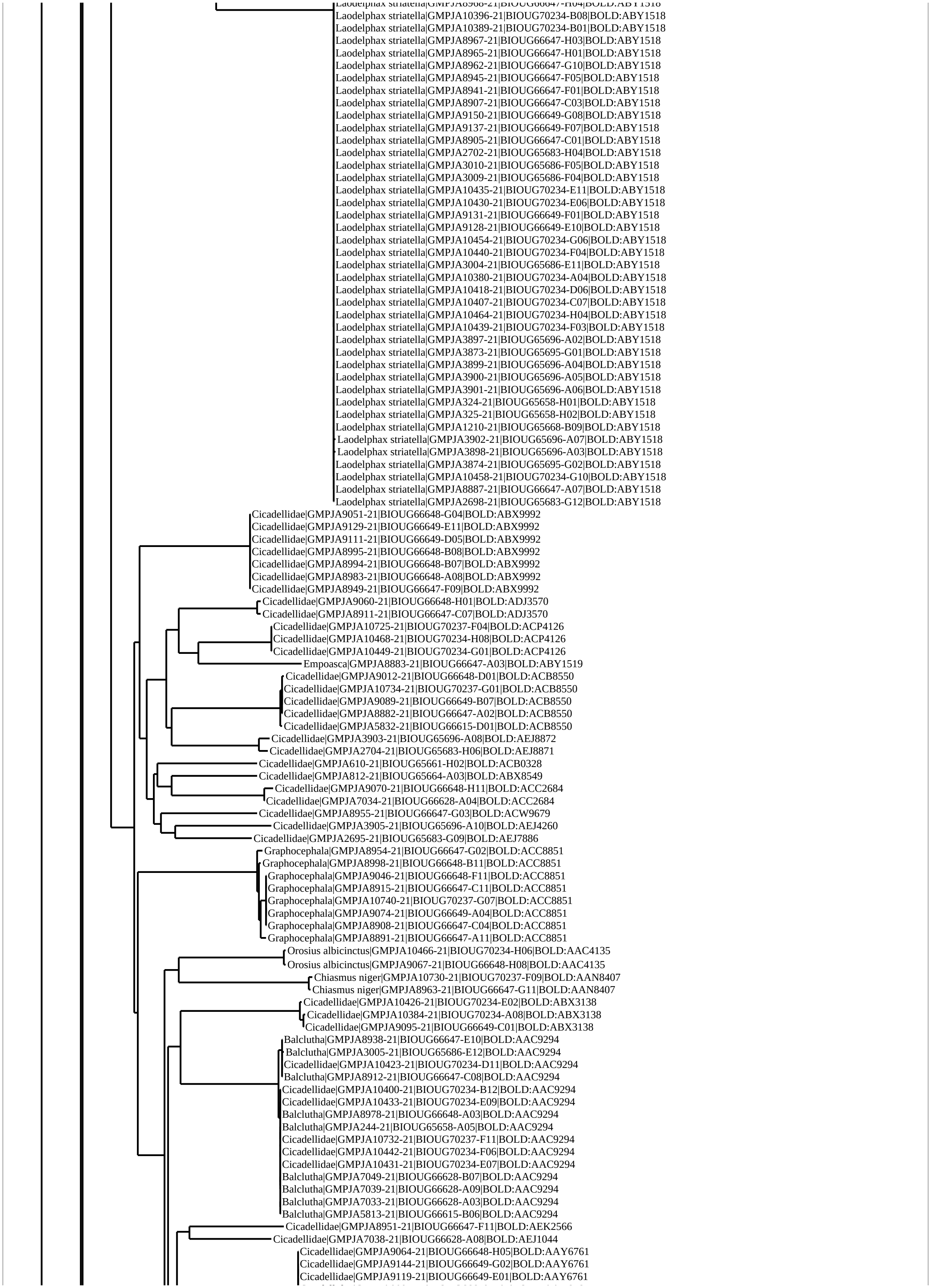

Laodelphax striatella|GMPJA8968-21|BIOUG66647-H04|BOLD:ABY1518
Laodelphax striatella|GMPJA10396-21|BIOUG70234-B08|BOLD:ABY1518
Laodelphax striatella|GMPJA10389-21|BIOUG70234-B01|BOLD:ABY1518
Laodelphax striatella|GMPJA8967-21|BIOUG66647-H03|BOLD:ABY1518
Laodelphax striatella|GMPJA8965-21|BIOUG66647-H01|BOLD:ABY1518
Laodelphax striatella|GMPJA8962-21|BIOUG66647-G10|BOLD:ABY1518
Laodelphax striatella|GMPJA8945-21|BIOUG66647-F05|BOLD:ABY1518
Laodelphax striatella|GMPJA8941-21|BIOUG66647-F01|BOLD:ABY1518
Laodelphax striatella|GMPJA8907-21|BIOUG66647-C03|BOLD:ABY1518
Laodelphax striatella|GMPJA9150-21|BIOUG66649-G08|BOLD:ABY1518
Laodelphax striatella|GMPJA9137-21|BIOUG66649-F07|BOLD:ABY1518
Laodelphax striatella|GMPJA8905-21|BIOUG66647-C01|BOLD:ABY1518
Laodelphax striatella|GMPJA2702-21|BIOUG65683-H04|BOLD:ABY1518
Laodelphax striatella|GMPJA3010-21|BIOUG65686-F05|BOLD:ABY1518
Laodelphax striatella|GMPJA3009-21|BIOUG65686-F04|BOLD:ABY1518
Laodelphax striatella|GMPJA10435-21|BIOUG70234-E11|BOLD:ABY1518
Laodelphax striatella|GMPJA10430-21|BIOUG70234-E06|BOLD:ABY1518
Laodelphax striatella|GMPJA9131-21|BIOUG66649-F01|BOLD:ABY1518
Laodelphax striatella|GMPJA9128-21|BIOUG66649-E10|BOLD:ABY1518
Laodelphax striatella|GMPJA10454-21|BIOUG70234-G06|BOLD:ABY1518
Laodelphax striatella|GMPJA10440-21|BIOUG70234-F04|BOLD:ABY1518
Laodelphax striatella|GMPJA3004-21|BIOUG65686-E11|BOLD:ABY1518
Laodelphax striatella|GMPJA10380-21|BIOUG70234-A04|BOLD:ABY1518
Laodelphax striatella|GMPJA10418-21|BIOUG70234-D06|BOLD:ABY1518
Laodelphax striatella|GMPJA10407-21|BIOUG70234-C07|BOLD:ABY1518
Laodelphax striatella|GMPJA10464-21|BIOUG70234-H04|BOLD:ABY1518
Laodelphax striatella|GMPJA10439-21|BIOUG70234-F03|BOLD:ABY1518
Laodelphax striatella|GMPJA3897-21|BIOUG65696-A02|BOLD:ABY1518
Laodelphax striatella|GMPJA3873-21|BIOUG65695-G01|BOLD:ABY1518
Laodelphax striatella|GMPJA3899-21|BIOUG65696-A04|BOLD:ABY1518
Laodelphax striatella|GMPJA3900-21|BIOUG65696-A05|BOLD:ABY1518
Laodelphax striatella|GMPJA3901-21|BIOUG65696-A06|BOLD:ABY1518
Laodelphax striatella|GMPJA324-21|BIOUG65658-H01|BOLD:ABY1518
Laodelphax striatella|GMPJA325-21|BIOUG65658-H02|BOLD:ABY1518
Laodelphax striatella|GMPJA1210-21|BIOUG65668-B09|BOLD:ABY1518
Laodelphax striatella|GMPJA3902-21|BIOUG65696-A07|BOLD:ABY1518
Laodelphax striatella|GMPJA3898-21|BIOUG65696-A03|BOLD:ABY1518
Laodelphax striatella|GMPJA3874-21|BIOUG65695-G02|BOLD:ABY1518
Laodelphax striatella|GMPJA10458-21|BIOUG70234-G10|BOLD:ABY1518
Laodelphax striatella|GMPJA8887-21|BIOUG66647-A07|BOLD:ABY1518
Laodelphax striatella|GMPJA2698-21|BIOUG65683-G12|BOLD:ABY1518
Cicadellidae|GMPJA9051-21|BIOUG66648-G04|BOLD:ABX9992
Cicadellidae|GMPJA9129-21|BIOUG66649-E11|BOLD:ABX9992
Cicadellidae|GMPJA9111-21|BIOUG66649-D05|BOLD:ABX9992
Cicadellidae|GMPJA8995-21|BIOUG66648-B08|BOLD:ABX9992
Cicadellidae|GMPJA8994-21|BIOUG66648-B07|BOLD:ABX9992
Cicadellidae|GMPJA8983-21|BIOUG66648-A08|BOLD:ABX9992
Cicadellidae|GMPJA8949-21|BIOUG66647-F09|BOLD:ABX9992
Cicadellidae|GMPJA9060-21|BIOUG66648-H01|BOLD:ADJ3570
Cicadellidae|GMPJA8911-21|BIOUG66647-C07|BOLD:ADJ3570
Cicadellidae|GMPJA10725-21|BIOUG70237-F04|BOLD:ACP4126
Cicadellidae|GMPJA10468-21|BIOUG70234-H08|BOLD:ACP4126
Cicadellidae|GMPJA10449-21|BIOUG70234-G01|BOLD:ACP4126
Empoasca|GMPJA8883-21|BIOUG66647-A03|BOLD:ABY1519
Cicadellidae|GMPJA9012-21|BIOUG66648-D01|BOLD:ACB8550
Cicadellidae|GMPJA10734-21|BIOUG70237-G01|BOLD:ACB8550
Cicadellidae|GMPJA9089-21|BIOUG66649-B07|BOLD:ACB8550
Cicadellidae|GMPJA8882-21|BIOUG66647-A02|BOLD:ACB8550
Cicadellidae|GMPJA5832-21|BIOUG66615-D01|BOLD:ACB8550
Cicadellidae|GMPJA3903-21|BIOUG65696-A08|BOLD:AEJ8872
Cicadellidae|GMPJA2704-21|BIOUG65683-H06|BOLD:AEJ8871
Cicadellidae|GMPJA610-21|BIOUG65661-H02|BOLD:ACB0328
Cicadellidae|GMPJA812-21|BIOUG65664-A03|BOLD:ABX8549
Cicadellidae|GMPJA9070-21|BIOUG66648-H11|BOLD:ACC2684
Cicadellidae|GMPJA7034-21|BIOUG66628-A04|BOLD:ACC2684
Cicadellidae|GMPJA8955-21|BIOUG66647-G03|BOLD:ACW9679
Cicadellidae|GMPJA3905-21|BIOUG65696-A10|BOLD:AEJ4260
Cicadellidae|GMPJA2695-21|BIOUG65683-G09|BOLD:AEJ7886
Graphocephala|GMPJA8954-21|BIOUG66647-G02|BOLD:ACC8851
Graphocephala|GMPJA8998-21|BIOUG66648-B11|BOLD:ACC8851
Graphocephala|GMPJA9046-21|BIOUG66648-F11|BOLD:ACC8851
Graphocephala|GMPJA8915-21|BIOUG66647-C11|BOLD:ACC8851
Graphocephala|GMPJA10740-21|BIOUG70237-G07|BOLD:ACC8851
Graphocephala|GMPJA9074-21|BIOUG66649-A04|BOLD:ACC8851
Graphocephala|GMPJA8908-21|BIOUG66647-C04|BOLD:ACC8851
Graphocephala|GMPJA8891-21|BIOUG66647-A11|BOLD:ACC8851
Orosius albicinctus|GMPJA10466-21|BIOUG70234-H06|BOLD:AAC4135
Orosius albicinctus|GMPJA9067-21|BIOUG66648-H08|BOLD:AAC4135
Chiasmus niger|GMPJA10730-21|BIOUG70237-F09|BOLD:AAN8407
Chiasmus niger|GMPJA8963-21|BIOUG66647-G11|BOLD:AAN8407
Cicadellidae|GMPJA10426-21|BIOUG70234-E02|BOLD:ABX3138
Cicadellidae|GMPJA10384-21|BIOUG70234-A08|BOLD:ABX3138
Cicadellidae|GMPJA9095-21|BIOUG66649-C01|BOLD:ABX3138
Balclutha|GMPJA8938-21|BIOUG66647-E10|BOLD:AAC9294
Balclutha|GMPJA3005-21|BIOUG65686-E12|BOLD:AAC9294
Cicadellidae|GMPJA10423-21|BIOUG70234-D11|BOLD:AAC9294
Balclutha|GMPJA8912-21|BIOUG66647-C08|BOLD:AAC9294
Cicadellidae|GMPJA10400-21|BIOUG70234-B12|BOLD:AAC9294
Cicadellidae|GMPJA10433-21|BIOUG70234-E09|BOLD:AAC9294
Balclutha|GMPJA8978-21|BIOUG66648-A03|BOLD:AAC9294
Balclutha|GMPJA244-21|BIOUG65658-A05|BOLD:AAC9294
Cicadellidae|GMPJA10732-21|BIOUG70237-F11|BOLD:AAC9294
Cicadellidae|GMPJA10442-21|BIOUG70234-F06|BOLD:AAC9294
Cicadellidae|GMPJA10431-21|BIOUG70234-E07|BOLD:AAC9294
Balclutha|GMPJA7049-21|BIOUG66628-B07|BOLD:AAC9294
Balclutha|GMPJA7039-21|BIOUG66628-A09|BOLD:AAC9294
Balclutha|GMPJA7033-21|BIOUG66628-A03|BOLD:AAC9294
Balclutha|GMPJA5813-21|BIOUG66615-B06|BOLD:AAC9294
Cicadellidae|GMPJA8951-21|BIOUG66647-F11|BOLD:AEK2566
Cicadellidae|GMPJA7038-21|BIOUG66628-A08|BOLD:AEJ1044
Cicadellidae|GMPJA9064-21|BIOUG66648-H05|BOLD:AAY6761
Cicadellidae|GMPJA9144-21|BIOUG66649-G02|BOLD:AAY6761
Cicadellidae|GMPJA9119-21|BIOUG66649-E01|BOLD:AAY6761

Cicadellidae|GMPJA9064-21|BIOUG66648-H05|BOLD:AAY6761
Cicadellidae|GMPJA9144-21|BIOUG66649-G02|BOLD:AAY6761
Cicadellidae|GMPJA9119-21|BIOUG66649-E01|BOLD:AAY6761
Cicadellidae|GMPJA9084-21|BIOUG66649-B02|BOLD:AAY6761
Cicadellidae|GMPJA9063-21|BIOUG66648-H04|BOLD:AAY6761
Cicadellidae|GMPJA9008-21|BIOUG66648-C09|BOLD:AAY6761
Cicadellidae|GMPJA7045-21|BIOUG66628-B03|BOLD:AAY6761
Cicadellidae|GMPJA7029-21|BIOUG66627-H10|BOLD:AAY6761
Psammotettix|GMPJA9083-21|BIOUG66649-B01|BOLD:AAN8400
Exitianus|GMPJA326-21|BIOUG65658-H03|BOLD:AAZ5690
Exitianus|GMPJA5819-21|BIOUG66615-B12|BOLD:AAZ5690
Cicadellidae|GMPJA10388-21|BIOUG70234-A12|BOLD:ABY1520
Cicadellidae|GMPJA10385-21|BIOUG70234-A09|BOLD:ABY1520
Cicadellidae|GMPJA10419-21|BIOUG70234-D07|BOLD:ABY1520
Cicadellidae|GMPJA7051-21|BIOUG66628-B09|BOLD:ABY1520
Cicadellidae|GMPJA7048-21|BIOUG66628-B06|BOLD:ABY1520
Cicadellidae|GMPJA7046-21|BIOUG66628-B04|BOLD:ABY1520
Cicadellidae|GMPJA7032-21|BIOUG66628-A02|BOLD:ABY1520
Cicadellidae|GMPJA7028-21|BIOUG66627-H09|BOLD:ABY1520
Maiestas|GMPJA7030-21|BIOUG66627-H11|BOLD:AAZ2068
Maiestas hopponis|GMPJA10427-21|BIOUG70234-E03|BOLD:ACF9803
Maiestas hopponis|GMPJA9100-21|BIOUG66649-C06|BOLD:ACF9803
Maiestas hopponis|GMPJA9068-21|BIOUG66648-H09|BOLD:ACF9803
Maiestas hopponis|GMPJA9102-21|BIOUG66649-C08|BOLD:ACF9803
Maiestas hopponis|GMPJA9123-21|BIOUG66649-E05|BOLD:ACF9803
Maiestas hopponis|GMPJA10406-21|BIOUG70234-C06|BOLD:ACF9803
Maiestas hopponis|GMPJA10437-21|BIOUG70234-F01|BOLD:ACF9803
Maiestas hopponis|GMPJA9022-21|BIOUG66648-D11|BOLD:ACF9803
Maiestas hopponis|GMPJA9034-21|BIOUG66648-E11|BOLD:ACF9803
Maiestas hopponis|GMPJA8979-21|BIOUG66648-A04|BOLD:ACF9803
Maiestas hopponis|GMPJA8975-21|BIOUG66647-H11|BOLD:ACF9803
Maiestas hopponis|GMPJA8958-21|BIOUG66647-G06|BOLD:ACF9803
Maiestas hopponis|GMPJA8888-21|BIOUG66647-A08|BOLD:ACF9803
Maiestas hopponis|GMPJA7036-21|BIOUG66628-A06|BOLD:ACF9803
Maiestas hopponis|GMPJA10377-21|BIOUG70234-A01|BOLD:ACF9803
Maiestas hopponis|GMPJA8964-21|BIOUG66647-G12|BOLD:ACF9803
Maiestas hopponis|GMPJA10394-21|BIOUG70234-B06|BOLD:ACF9803
Maiestas hopponis|GMPJA10422-21|BIOUG70234-D10|BOLD:ACF9803
Maiestas hopponis|GMPJA10514-21|BIOUG70235-D07|BOLD:ACF9803
Maiestas hopponis|GMPJA10428-21|BIOUG70234-E04|BOLD:ACF9803
Maiestas hopponis|GMPJA10381-21|BIOUG70234-A05|BOLD:ACF9803
Maiestas hopponis|GMPJA9099-21|BIOUG66649-C05|BOLD:ACF9803
Maiestas hopponis|GMPJA9080-21|BIOUG66649-A10|BOLD:ACF9803
Maiestas hopponis|GMPJA9038-21|BIOUG66648-F03|BOLD:ACF9803
Maiestas hopponis|GMPJA8991-21|BIOUG66648-B04|BOLD:ACF9803
Maiestas hopponis|GMPJA8977-21|BIOUG66648-A02|BOLD:ACF9803
Maiestas hopponis|GMPJA8936-21|BIOUG66647-E08|BOLD:ACF9803
Maiestas hopponis|GMPJA8913-21|BIOUG66647-C09|BOLD:ACF9803
Maiestas hopponis|GMPJA7040-21|BIOUG66628-A10|BOLD:ACF9803
Maiestas hopponis|GMPJA8910-21|BIOUG66647-C06|BOLD:ACF9803
Maiestas hopponis|GMPJA8980-21|BIOUG66648-A05|BOLD:ACF9803
Maiestas hopponis|GMPJA10452-21|BIOUG70234-G04|BOLD:ACF9803
Maiestas hopponis|GMPJA10443-21|BIOUG70234-F07|BOLD:ACF9803
Maiestas hopponis|GMPJA10387-21|BIOUG70234-A11|BOLD:ACF9803
Maiestas hopponis|GMPJA10386-21|BIOUG70234-A10|BOLD:ACF9803
Maiestas hopponis|GMPJA9097-21|BIOUG66649-C03|BOLD:ACF9803
Maiestas hopponis|GMPJA9096-21|BIOUG66649-C02|BOLD:ACF9803
Maiestas hopponis|GMPJA9075-21|BIOUG66649-A05|BOLD:ACF9803
Maiestas hopponis|GMPJA9058-21|BIOUG66648-G11|BOLD:ACF9803
Maiestas hopponis|GMPJA9085-21|BIOUG66649-B03|BOLD:ACF9803
Maiestas hopponis|GMPJA9076-21|BIOUG66649-A06|BOLD:ACF9803
Maiestas hopponis|GMPJA10401-21|BIOUG70234-C01|BOLD:ACF9803
Maiestas hopponis|GMPJA10398-21|BIOUG70234-B10|BOLD:ACF9803
Maiestas hopponis|GMPJA10463-21|BIOUG70234-H03|BOLD:ACF9803
Maiestas hopponis|GMPJA10462-21|BIOUG70234-H02|BOLD:ACF9803
Maiestas hopponis|GMPJA10513-21|BIOUG70235-D06|BOLD:ACF9803
Maiestas hopponis|GMPJA10467-21|BIOUG70234-H07|BOLD:ACF9803
Maiestas hopponis|GMPJA10390-21|BIOUG70234-B02|BOLD:ACF9803
Maiestas hopponis|GMPJA10383-21|BIOUG70234-A07|BOLD:ACF9803
Maiestas hopponis|GMPJA10397-21|BIOUG70234-B09|BOLD:ACF9803
Maiestas hopponis|GMPJA10410-21|BIOUG70234-C10|BOLD:ACF9803
Maiestas hopponis|GMPJA10416-21|BIOUG70234-D04|BOLD:ACF9803
Maiestas hopponis|GMPJA10434-21|BIOUG70234-E10|BOLD:ACF9803
Maiestas hopponis|GMPJA10459-21|BIOUG70234-G11|BOLD:ACF9803
Maiestas hopponis|GMPJA9062-21|BIOUG66648-H03|BOLD:ACF9803
Maiestas hopponis|GMPJA9057-21|BIOUG66648-G10|BOLD:ACF9803
Maiestas hopponis|GMPJA9052-21|BIOUG66648-G05|BOLD:ACF9803
Maiestas hopponis|GMPJA9043-21|BIOUG66648-F08|BOLD:ACF9803
Maiestas hopponis|GMPJA9016-21|BIOUG66648-D05|BOLD:ACF9803
Maiestas hopponis|GMPJA9014-21|BIOUG66648-D03|BOLD:ACF9803
Maiestas hopponis|GMPJA9010-21|BIOUG66648-C11|BOLD:ACF9803
Maiestas hopponis|GMPJA9009-21|BIOUG66648-C10|BOLD:ACF9803
Maiestas hopponis|GMPJA8992-21|BIOUG66648-B05|BOLD:ACF9803
Maiestas hopponis|GMPJA8957-21|BIOUG66647-G05|BOLD:ACF9803
Maiestas hopponis|GMPJA8914-21|BIOUG66647-C10|BOLD:ACF9803
Maiestas hopponis|GMPJA8909-21|BIOUG66647-C05|BOLD:ACF9803
Maiestas hopponis|GMPJA8906-21|BIOUG66647-C02|BOLD:ACF9803
Maiestas hopponis|GMPJA8890-21|BIOUG66647-A10|BOLD:ACF9803
Maiestas hopponis|GMPJA8889-21|BIOUG66647-A09|BOLD:ACF9803
Maiestas hopponis|GMPJA8886-21|BIOUG66647-A06|BOLD:ACF9803
Maiestas hopponis|GMPJA8884-21|BIOUG66647-A04|BOLD:ACF9803
Maiestas hopponis|GMPJA7044-21|BIOUG66628-B02|BOLD:ACF9803
Maiestas hopponis|GMPJA7042-21|BIOUG66628-A12|BOLD:ACF9803
Maiestas hopponis|GMPJA7041-21|BIOUG66628-A11|BOLD:ACF9803
Maiestas hopponis|GMPJA7037-21|BIOUG66628-A07|BOLD:ACF9803
Maiestas hopponis|GMPJA7035-21|BIOUG66628-A05|BOLD:ACF9803
Maiestas hopponis|GMPJA7027-21|BIOUG66627-H08|BOLD:ACF9803
Maiestas hopponis|GMPJA7026-21|BIOUG66627-H07|BOLD:ACF9803
Mukaria splendida|GMPJA609-21|BIOUG65661-H01|BOLD:ADV6524
Mukaria splendida|GMPJA9077-21|BIOUG66649-A07|BOLD:ADV6524
Mukaria splendida|GMPJA5825-21|BIOUG66615-C06|BOLD:ADV6524
Mukaria splendida|GMPJA4951-21|BIOUG66606-A11|BOLD:ADV6524
Cicadellidae|GMPJA811-21|BIOUG65664-A02|BOLD:AAY6763

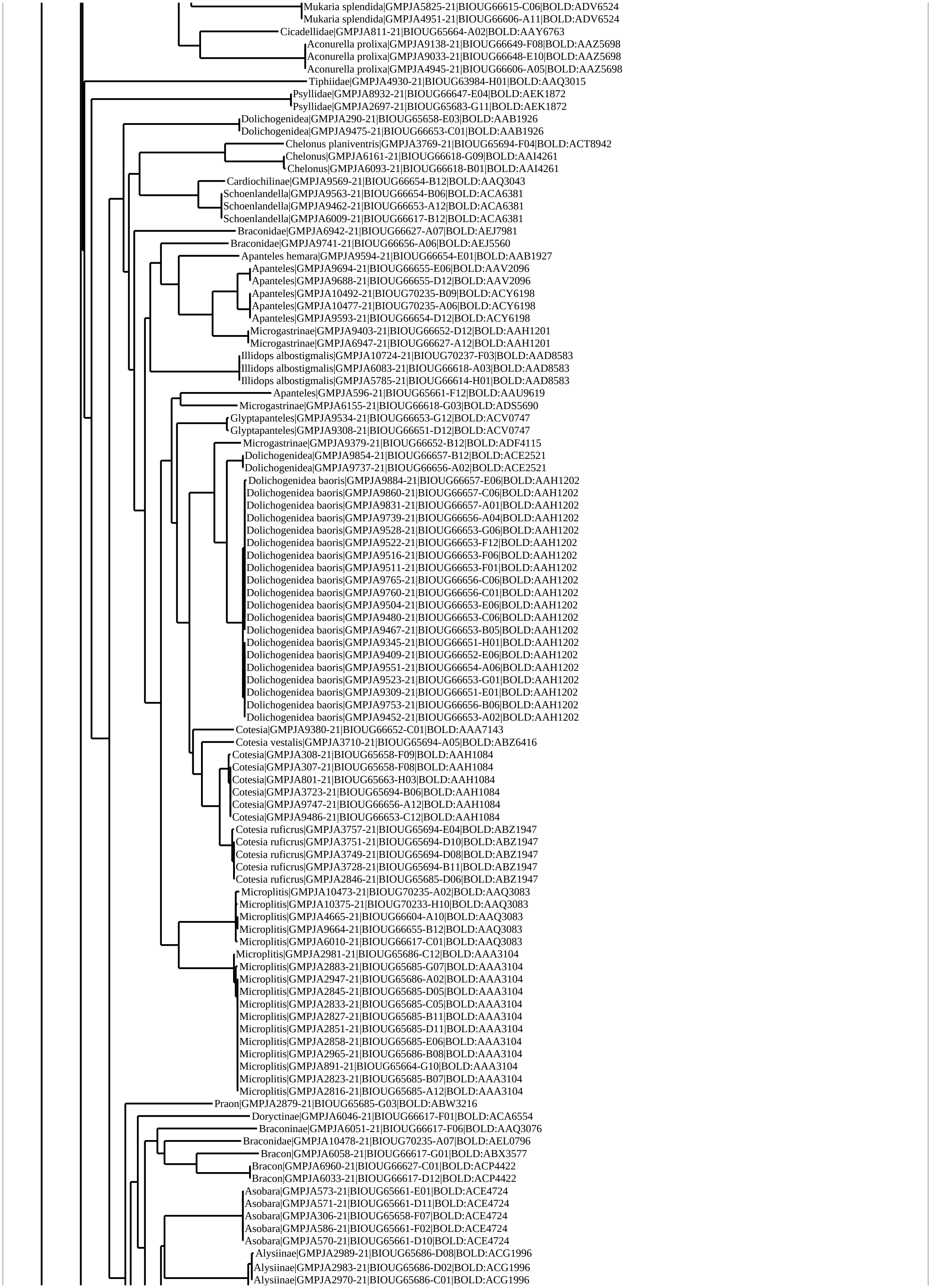
Mukaria splendida|GMPJA5825-21|BIOUG66615-C06|BOLD:ADV6524
Mukaria splendida|GMPJA4951-21|BIOUG66606-A11|BOLD:ADV6524
Cicadellidae|GMPJA811-21|BIOUG65664-A02|BOLD:AAY6763
Aconurella prolixa|GMPJA9138-21|BIOUG66649-F08|BOLD:AAZ5698
Aconurella prolixa|GMPJA9033-21|BIOUG66648-E10|BOLD:AAZ5698
Aconurella prolixa|GMPJA4945-21|BIOUG66606-A05|BOLD:AAZ5698
Tiphiidae|GMPJA4930-21|BIOUG63984-H01|BOLD:AAQ3015
Psyllidae|GMPJA8932-21|BIOUG66647-E04|BOLD:AEK1872
Psyllidae|GMPJA2697-21|BIOUG65683-G11|BOLD:AEK1872
Dolichogenidea|GMPJA290-21|BIOUG65658-E03|BOLD:AAB1926
Dolichogenidea|GMPJA9475-21|BIOUG66653-C01|BOLD:AAB1926
Chelonus planiventris|GMPJA3769-21|BIOUG65694-F04|BOLD:ACT8942
Chelonus|GMPJA6161-21|BIOUG66618-G09|BOLD:AAI4261
Chelonus|GMPJA6093-21|BIOUG66618-B01|BOLD:AAI4261
Cardiochilinae|GMPJA9569-21|BIOUG66654-B12|BOLD:AAQ3043
Schoenlandella|GMPJA9563-21|BIOUG66654-B06|BOLD:ACA6381
Schoenlandella|GMPJA9462-21|BIOUG66653-A12|BOLD:ACA6381
Schoenlandella|GMPJA6009-21|BIOUG66617-B12|BOLD:ACA6381
Braconidae|GMPJA6942-21|BIOUG66627-A07|BOLD:AEJ7981
Braconidae|GMPJA9741-21|BIOUG66656-A06|BOLD:AEJ5560
Apanteles hemara|GMPJA9594-21|BIOUG66654-E01|BOLD:AAB1927
Apanteles|GMPJA9694-21|BIOUG66655-E06|BOLD:AAV2096
Apanteles|GMPJA9688-21|BIOUG66655-D12|BOLD:AAV2096
Apanteles|GMPJA10492-21|BIOUG70235-B09|BOLD:ACY6198
Apanteles|GMPJA10477-21|BIOUG70235-A06|BOLD:ACY6198
Apanteles|GMPJA9593-21|BIOUG66654-D12|BOLD:ACY6198
Microgastrinae|GMPJA9403-21|BIOUG66652-D12|BOLD:AAH1201
Microgastrinae|GMPJA6947-21|BIOUG66627-A12|BOLD:AAH1201
Illidops albostigmalis|GMPJA10724-21|BIOUG70237-F03|BOLD:AAD8583
Illidops albostigmalis|GMPJA6083-21|BIOUG66618-A03|BOLD:AAD8583
Illidops albostigmalis|GMPJA5785-21|BIOUG66614-H01|BOLD:AAD8583
Apanteles|GMPJA596-21|BIOUG65661-F12|BOLD:AAU9619
Microgastrinae|GMPJA6155-21|BIOUG66618-G03|BOLD:ADS5690
Glyptapanteles|GMPJA9534-21|BIOUG66653-G12|BOLD:ACV0747
Glyptapanteles|GMPJA9308-21|BIOUG66651-D12|BOLD:ACV0747
Microgastrinae|GMPJA9379-21|BIOUG66652-B12|BOLD:ADF4115
Dolichogenidea|GMPJA9854-21|BIOUG66657-B12|BOLD:ACE2521
Dolichogenidea|GMPJA9737-21|BIOUG66656-A02|BOLD:ACE2521
Dolichogenidea baoris|GMPJA9884-21|BIOUG66657-E06|BOLD:AAH1202
Dolichogenidea baoris|GMPJA9860-21|BIOUG66657-C06|BOLD:AAH1202
Dolichogenidea baoris|GMPJA9831-21|BIOUG66657-A01|BOLD:AAH1202
Dolichogenidea baoris|GMPJA9739-21|BIOUG66656-A04|BOLD:AAH1202
Dolichogenidea baoris|GMPJA9528-21|BIOUG66653-G06|BOLD:AAH1202
Dolichogenidea baoris|GMPJA9522-21|BIOUG66653-F12|BOLD:AAH1202
Dolichogenidea baoris|GMPJA9516-21|BIOUG66653-F06|BOLD:AAH1202
Dolichogenidea baoris|GMPJA9511-21|BIOUG66653-F01|BOLD:AAH1202
Dolichogenidea baoris|GMPJA9765-21|BIOUG66656-C06|BOLD:AAH1202
Dolichogenidea baoris|GMPJA9760-21|BIOUG66656-C01|BOLD:AAH1202
Dolichogenidea baoris|GMPJA9504-21|BIOUG66653-E06|BOLD:AAH1202
Dolichogenidea baoris|GMPJA9480-21|BIOUG66653-C06|BOLD:AAH1202
Dolichogenidea baoris|GMPJA9467-21|BIOUG66653-B05|BOLD:AAH1202
Dolichogenidea baoris|GMPJA9345-21|BIOUG66651-H01|BOLD:AAH1202
Dolichogenidea baoris|GMPJA9409-21|BIOUG66652-E06|BOLD:AAH1202
Dolichogenidea baoris|GMPJA9551-21|BIOUG66654-A06|BOLD:AAH1202
Dolichogenidea baoris|GMPJA9523-21|BIOUG66653-G01|BOLD:AAH1202
Dolichogenidea baoris|GMPJA9309-21|BIOUG66651-E01|BOLD:AAH1202
Dolichogenidea baoris|GMPJA9753-21|BIOUG66656-B06|BOLD:AAH1202
Dolichogenidea baoris|GMPJA9452-21|BIOUG66653-A02|BOLD:AAH1202
Cotesia|GMPJA9380-21|BIOUG66652-C01|BOLD:AAA7143
Cotesia vestalis|GMPJA3710-21|BIOUG65694-A05|BOLD:ABZ6416
Cotesia|GMPJA308-21|BIOUG65658-F09|BOLD:AAH1084
Cotesia|GMPJA307-21|BIOUG65658-F08|BOLD:AAH1084
Cotesia|GMPJA801-21|BIOUG65663-H03|BOLD:AAH1084
Cotesia|GMPJA3723-21|BIOUG65694-B06|BOLD:AAH1084
Cotesia|GMPJA9747-21|BIOUG66656-A12|BOLD:AAH1084
Cotesia|GMPJA9486-21|BIOUG66653-C12|BOLD:AAH1084
Cotesia ruficrus|GMPJA3757-21|BIOUG65694-E04|BOLD:ABZ1947
Cotesia ruficrus|GMPJA3751-21|BIOUG65694-D10|BOLD:ABZ1947
Cotesia ruficrus|GMPJA3749-21|BIOUG65694-D08|BOLD:ABZ1947
Cotesia ruficrus|GMPJA3728-21|BIOUG65694-B11|BOLD:ABZ1947
Cotesia ruficrus|GMPJA2846-21|BIOUG65685-D06|BOLD:ABZ1947
Microplitis|GMPJA10473-21|BIOUG70235-A02|BOLD:AAQ3083
Microplitis|GMPJA10375-21|BIOUG70233-H10|BOLD:AAQ3083
Microplitis|GMPJA4665-21|BIOUG66604-A10|BOLD:AAQ3083
Microplitis|GMPJA9664-21|BIOUG66655-B12|BOLD:AAQ3083
Microplitis|GMPJA6010-21|BIOUG66617-C01|BOLD:AAQ3083
Microplitis|GMPJA2981-21|BIOUG65686-C12|BOLD:AAA3104
Microplitis|GMPJA2883-21|BIOUG65685-G07|BOLD:AAA3104
Microplitis|GMPJA2947-21|BIOUG65686-A02|BOLD:AAA3104
Microplitis|GMPJA2845-21|BIOUG65685-D05|BOLD:AAA3104
Microplitis|GMPJA2833-21|BIOUG65685-C05|BOLD:AAA3104
Microplitis|GMPJA2827-21|BIOUG65685-B11|BOLD:AAA3104
Microplitis|GMPJA2851-21|BIOUG65685-D11|BOLD:AAA3104
Microplitis|GMPJA2858-21|BIOUG65685-E06|BOLD:AAA3104
Microplitis|GMPJA2965-21|BIOUG65686-B08|BOLD:AAA3104
Microplitis|GMPJA891-21|BIOUG65664-G10|BOLD:AAA3104
Microplitis|GMPJA2823-21|BIOUG65685-B07|BOLD:AAA3104
Microplitis|GMPJA2816-21|BIOUG65685-A12|BOLD:AAA3104
Praon|GMPJA2879-21|BIOUG65685-G03|BOLD:ABW3216
Doryctinae|GMPJA6046-21|BIOUG66617-F01|BOLD:ACA6554
Braconinae|GMPJA6051-21|BIOUG66617-F06|BOLD:AAQ3076
Braconidae|GMPJA10478-21|BIOUG70235-A07|BOLD:AEL0796
Bracon|GMPJA6058-21|BIOUG66617-G01|BOLD:ABX3577
Bracon|GMPJA6960-21|BIOUG66627-C01|BOLD:ACP4422
Bracon|GMPJA6033-21|BIOUG66617-D12|BOLD:ACP4422
Asobara|GMPJA573-21|BIOUG65661-E01|BOLD:ACE4724
Asobara|GMPJA571-21|BIOUG65661-D11|BOLD:ACE4724
Asobara|GMPJA306-21|BIOUG65658-F07|BOLD:ACE4724
Asobara|GMPJA586-21|BIOUG65661-F02|BOLD:ACE4724
Asobara|GMPJA570-21|BIOUG65661-D10|BOLD:ACE4724
Alysiinae|GMPJA2989-21|BIOUG65686-D08|BOLD:ACG1996
Alysiinae|GMPJA2983-21|BIOUG65686-D02|BOLD:ACG1996
Alysiinae|GMPJA2970-21|BIOUG65686-C01|BOLD:ACG1996

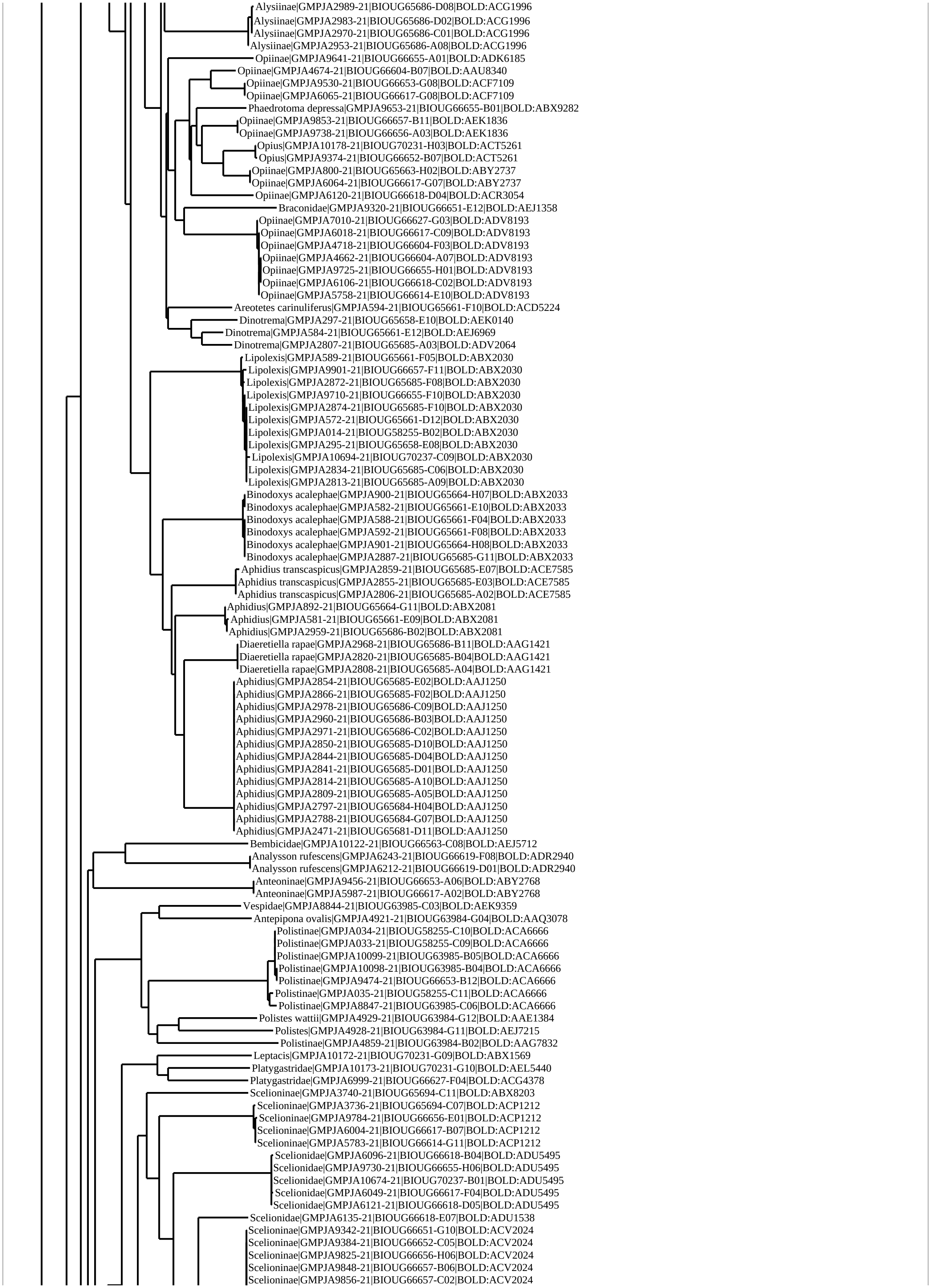
Alysiinae|GMPJA2989-21|BIOUG65686-D08|BOLD:ACG1996
Alysiinae|GMPJA2983-21|BIOUG65686-D02|BOLD:ACG1996
Alysiinae|GMPJA2970-21|BIOUG65686-C01|BOLD:ACG1996
Alysiinae|GMPJA2953-21|BIOUG65686-A08|BOLD:ACG1996
Opiinae|GMPJA9641-21|BIOUG66655-A01|BOLD:ADK6185
Opiinae|GMPJA4674-21|BIOUG66604-B07|BOLD:AAU8340
Opiinae|GMPJA9530-21|BIOUG66653-G08|BOLD:ACF7109
Opiinae|GMPJA6065-21|BIOUG66617-G08|BOLD:ACF7109
Phaedrotoma depressa|GMPJA9653-21|BIOUG66655-B01|BOLD:ABX9282
Opiinae|GMPJA9853-21|BIOUG66657-B11|BOLD:AEK1836
Opiinae|GMPJA9738-21|BIOUG66656-A03|BOLD:AEK1836
Opius|GMPJA10178-21|BIOUG70231-H03|BOLD:ACT5261
Opius|GMPJA9374-21|BIOUG66652-B07|BOLD:ACT5261
Opiinae|GMPJA800-21|BIOUG65663-H02|BOLD:ABY2737
Opiinae|GMPJA6064-21|BIOUG66617-G07|BOLD:ABY2737
Opiinae|GMPJA6120-21|BIOUG66618-D04|BOLD:ACR3054
Braconidae|GMPJA9320-21|BIOUG66651-E12|BOLD:AEJ1358
Opiinae|GMPJA7010-21|BIOUG66627-G03|BOLD:ADV8193
Opiinae|GMPJA6018-21|BIOUG66617-C09|BOLD:ADV8193
Opiinae|GMPJA4718-21|BIOUG66604-F03|BOLD:ADV8193
Opiinae|GMPJA4662-21|BIOUG66604-A07|BOLD:ADV8193
Opiinae|GMPJA9725-21|BIOUG66655-H01|BOLD:ADV8193
Opiinae|GMPJA6106-21|BIOUG66618-C02|BOLD:ADV8193
Opiinae|GMPJA5758-21|BIOUG66614-E10|BOLD:ADV8193
Areotetes carinuliferus|GMPJA594-21|BIOUG65661-F10|BOLD:ACD5224
Dinotrema|GMPJA297-21|BIOUG65658-E10|BOLD:AEK0140
Dinotrema|GMPJA584-21|BIOUG65661-E12|BOLD:AEJ6969
Dinotrema|GMPJA2807-21|BIOUG65685-A03|BOLD:ADV2064
Lipolexis|GMPJA589-21|BIOUG65661-F05|BOLD:ABX2030
Lipolexis|GMPJA9901-21|BIOUG66657-F11|BOLD:ABX2030
Lipolexis|GMPJA2872-21|BIOUG65685-F08|BOLD:ABX2030
Lipolexis|GMPJA9710-21|BIOUG66655-F10|BOLD:ABX2030
Lipolexis|GMPJA2874-21|BIOUG65685-F10|BOLD:ABX2030
Lipolexis|GMPJA572-21|BIOUG65661-D12|BOLD:ABX2030
Lipolexis|GMPJA014-21|BIOUG58255-B02|BOLD:ABX2030
Lipolexis|GMPJA295-21|BIOUG65658-E08|BOLD:ABX2030
Lipolexis|GMPJA10694-21|BIOUG70237-C09|BOLD:ABX2030
Lipolexis|GMPJA2834-21|BIOUG65685-C06|BOLD:ABX2030
Lipolexis|GMPJA2813-21|BIOUG65685-A09|BOLD:ABX2030
Binodoxys acalephae|GMPJA900-21|BIOUG65664-H07|BOLD:ABX2033
Binodoxys acalephae|GMPJA582-21|BIOUG65661-E10|BOLD:ABX2033
Binodoxys acalephae|GMPJA588-21|BIOUG65661-F04|BOLD:ABX2033
Binodoxys acalephae|GMPJA592-21|BIOUG65661-F08|BOLD:ABX2033
Binodoxys acalephae|GMPJA901-21|BIOUG65664-H08|BOLD:ABX2033
Binodoxys acalephae|GMPJA2887-21|BIOUG65685-G11|BOLD:ABX2033
Aphidius transcaspicus|GMPJA2859-21|BIOUG65685-E07|BOLD:ACE7585
Aphidius transcaspicus|GMPJA2855-21|BIOUG65685-E03|BOLD:ACE7585
Aphidius transcaspicus|GMPJA2806-21|BIOUG65685-A02|BOLD:ACE7585
Aphidius|GMPJA892-21|BIOUG65664-G11|BOLD:ABX2081
Aphidius|GMPJA581-21|BIOUG65661-E09|BOLD:ABX2081
Aphidius|GMPJA2959-21|BIOUG65686-B02|BOLD:ABX2081
Diaeretiella rapae|GMPJA2968-21|BIOUG65686-B11|BOLD:AAG1421
Diaeretiella rapae|GMPJA2820-21|BIOUG65685-B04|BOLD:AAG1421
Diaeretiella rapae|GMPJA2808-21|BIOUG65685-A04|BOLD:AAG1421
Aphidius|GMPJA2854-21|BIOUG65685-E02|BOLD:AAJ1250
Aphidius|GMPJA2866-21|BIOUG65685-F02|BOLD:AAJ1250
Aphidius|GMPJA2978-21|BIOUG65686-C09|BOLD:AAJ1250
Aphidius|GMPJA2960-21|BIOUG65686-B03|BOLD:AAJ1250
Aphidius|GMPJA2971-21|BIOUG65686-C02|BOLD:AAJ1250
Aphidius|GMPJA2850-21|BIOUG65685-D10|BOLD:AAJ1250
Aphidius|GMPJA2844-21|BIOUG65685-D04|BOLD:AAJ1250
Aphidius|GMPJA2841-21|BIOUG65685-D01|BOLD:AAJ1250
Aphidius|GMPJA2814-21|BIOUG65685-A10|BOLD:AAJ1250
Aphidius|GMPJA2809-21|BIOUG65685-A05|BOLD:AAJ1250
Aphidius|GMPJA2797-21|BIOUG65684-H04|BOLD:AAJ1250
Aphidius|GMPJA2788-21|BIOUG65684-G07|BOLD:AAJ1250
Aphidius|GMPJA2471-21|BIOUG65681-D11|BOLD:AAJ1250
Bembicidae|GMPJA10122-21|BIOUG66563-C08|BOLD:AEJ5712
Analysson rufescens|GMPJA6243-21|BIOUG66619-F08|BOLD:ADR2940
Analysson rufescens|GMPJA6212-21|BIOUG66619-D01|BOLD:ADR2940
Anteoninae|GMPJA9456-21|BIOUG66653-A06|BOLD:ABY2768
Anteoninae|GMPJA5987-21|BIOUG66617-A02|BOLD:ABY2768
Vespidae|GMPJA8844-21|BIOUG63985-C03|BOLD:AEK9359
Antepipona ovalis|GMPJA4921-21|BIOUG63984-G04|BOLD:AAQ3078
Polistinae|GMPJA034-21|BIOUG58255-C10|BOLD:ACA6666
Polistinae|GMPJA033-21|BIOUG58255-C09|BOLD:ACA6666
Polistinae|GMPJA10099-21|BIOUG63985-B05|BOLD:ACA6666
Polistinae|GMPJA10098-21|BIOUG63985-B04|BOLD:ACA6666
Polistinae|GMPJA9474-21|BIOUG66653-B12|BOLD:ACA6666
Polistinae|GMPJA035-21|BIOUG58255-C11|BOLD:ACA6666
Polistinae|GMPJA8847-21|BIOUG63985-C06|BOLD:ACA6666
Polistes wattii|GMPJA4929-21|BIOUG63984-G12|BOLD:AAE1384
Polistes|GMPJA4928-21|BIOUG63984-G11|BOLD:AEJ7215
Polistinae|GMPJA4859-21|BIOUG63984-B02|BOLD:AAG7832
Leptacis|GMPJA10172-21|BIOUG70231-G09|BOLD:ABX1569
Platygastridae|GMPJA10173-21|BIOUG70231-G10|BOLD:AEL5440
Platygastridae|GMPJA6999-21|BIOUG66627-F04|BOLD:ACG4378
Scelioninae|GMPJA3740-21|BIOUG65694-C11|BOLD:ABX8203
Scelioninae|GMPJA3736-21|BIOUG65694-C07|BOLD:ACP1212
Scelioninae|GMPJA9784-21|BIOUG66656-E01|BOLD:ACP1212
Scelioninae|GMPJA6004-21|BIOUG66617-B07|BOLD:ACP1212
Scelioninae|GMPJA5783-21|BIOUG66614-G11|BOLD:ACP1212
Scelionidae|GMPJA6096-21|BIOUG66618-B04|BOLD:ADU5495
Scelionidae|GMPJA9730-21|BIOUG66655-H06|BOLD:ADU5495
Scelionidae|GMPJA10674-21|BIOUG70237-B01|BOLD:ADU5495
Scelionidae|GMPJA6049-21|BIOUG66617-F04|BOLD:ADU5495
Scelionidae|GMPJA6121-21|BIOUG66618-D05|BOLD:ADU5495
Scelionidae|GMPJA6135-21|BIOUG66618-E07|BOLD:ADU1538
Scelioninae|GMPJA9342-21|BIOUG66651-G10|BOLD:ACV2024
Scelioninae|GMPJA9384-21|BIOUG66652-C05|BOLD:ACV2024
Scelioninae|GMPJA9825-21|BIOUG66656-H06|BOLD:ACV2024
Scelioninae|GMPJA9848-21|BIOUG66657-B06|BOLD:ACV2024
Scelioninae|GMPJA9856-21|BIOUG66657-C02|BOLD:ACV2024

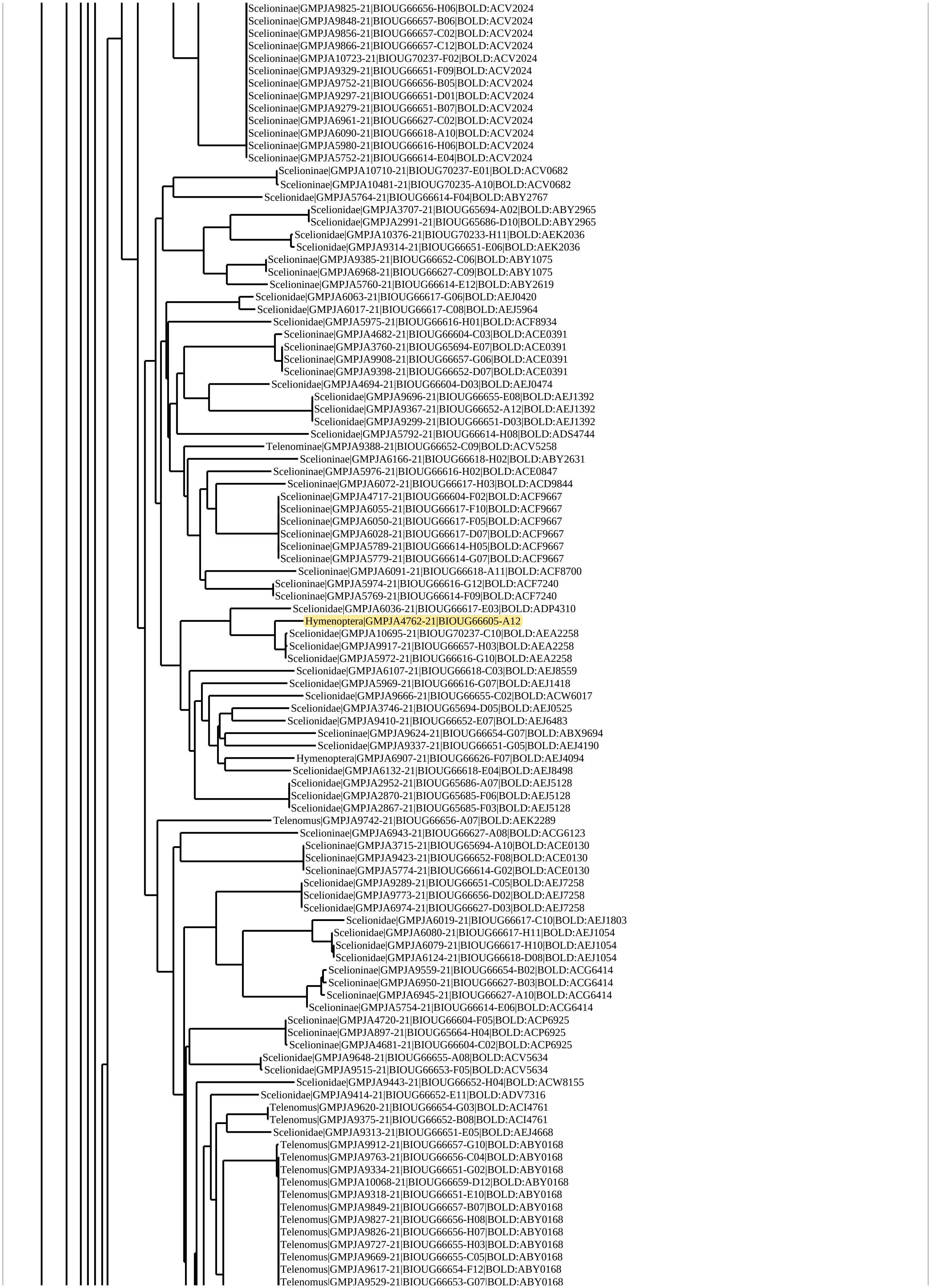
Scelioninae|GMPJA9825-21|BIOUG66656-H06|BOLD:ACV2024
Scelioninae|GMPJA9848-21|BIOUG66657-B06|BOLD:ACV2024
Scelioninae|GMPJA9856-21|BIOUG66657-C02|BOLD:ACV2024
Scelioninae|GMPJA9866-21|BIOUG66657-C12|BOLD:ACV2024
Scelioninae|GMPJA10723-21|BIOUG70237-F02|BOLD:ACV2024
Scelioninae|GMPJA9329-21|BIOUG66651-F09|BOLD:ACV2024
Scelioninae|GMPJA9752-21|BIOUG66656-B05|BOLD:ACV2024
Scelioninae|GMPJA9297-21|BIOUG66651-D01|BOLD:ACV2024
Scelioninae|GMPJA9279-21|BIOUG66651-B07|BOLD:ACV2024
Scelioninae|GMPJA6961-21|BIOUG66627-C02|BOLD:ACV2024
Scelioninae|GMPJA6090-21|BIOUG66618-A10|BOLD:ACV2024
Scelioninae|GMPJA5980-21|BIOUG66616-H06|BOLD:ACV2024
Scelioninae|GMPJA5752-21|BIOUG66614-E04|BOLD:ACV2024
Scelioninae|GMPJA10710-21|BIOUG70237-E01|BOLD:ACV0682
Scelioninae|GMPJA10481-21|BIOUG70235-A10|BOLD:ACV0682
Scelionidae|GMPJA5764-21|BIOUG66614-F04|BOLD:ABY2767
Scelionidae|GMPJA3707-21|BIOUG65694-A02|BOLD:ABY2965
Scelionidae|GMPJA2991-21|BIOUG65686-D10|BOLD:ABY2965
Scelionidae|GMPJA10376-21|BIOUG70233-H11|BOLD:AEK2036
Scelionidae|GMPJA9314-21|BIOUG66651-E06|BOLD:AEK2036
Scelioninae|GMPJA9385-21|BIOUG66652-C06|BOLD:ABY1075
Scelioninae|GMPJA6968-21|BIOUG66627-C09|BOLD:ABY1075
Scelioninae|GMPJA5760-21|BIOUG66614-E12|BOLD:ABY2619
Scelionidae|GMPJA6063-21|BIOUG66617-G06|BOLD:AEJ0420
Scelionidae|GMPJA6017-21|BIOUG66617-C08|BOLD:AEJ5964
Scelionidae|GMPJA5975-21|BIOUG66616-H01|BOLD:ACF8934
Scelioninae|GMPJA4682-21|BIOUG66604-C03|BOLD:ACE0391
Scelioninae|GMPJA3760-21|BIOUG65694-E07|BOLD:ACE0391
Scelioninae|GMPJA9908-21|BIOUG66657-G06|BOLD:ACE0391
Scelioninae|GMPJA9398-21|BIOUG66652-D07|BOLD:ACE0391
Scelionidae|GMPJA4694-21|BIOUG66604-D03|BOLD:AEJ0474
Scelionidae|GMPJA9696-21|BIOUG66655-E08|BOLD:AEJ1392
Scelionidae|GMPJA9367-21|BIOUG66652-A12|BOLD:AEJ1392
Scelionidae|GMPJA9299-21|BIOUG66651-D03|BOLD:AEJ1392
Scelionidae|GMPJA5792-21|BIOUG66614-H08|BOLD:ADS4744
Telenominae|GMPJA9388-21|BIOUG66652-C09|BOLD:ACV5258
Scelioninae|GMPJA6166-21|BIOUG66618-H02|BOLD:ABY2631
Scelionidae|GMPJA5976-21|BIOUG66616-H02|BOLD:ACE0847
Scelioninae|GMPJA6072-21|BIOUG66617-H03|BOLD:ACD9844
Scelioninae|GMPJA4717-21|BIOUG66604-F02|BOLD:ACF9667
Scelioninae|GMPJA6055-21|BIOUG66617-F10|BOLD:ACF9667
Scelioninae|GMPJA6050-21|BIOUG66617-F05|BOLD:ACF9667
Scelioninae|GMPJA6028-21|BIOUG66617-D07|BOLD:ACF9667
Scelioninae|GMPJA5789-21|BIOUG66614-H05|BOLD:ACF9667
Scelioninae|GMPJA5779-21|BIOUG66614-G07|BOLD:ACF9667
Scelioninae|GMPJA6091-21|BIOUG66618-A11|BOLD:ACF8700
Scelioninae|GMPJA5974-21|BIOUG66616-G12|BOLD:ACF7240
Scelioninae|GMPJA5769-21|BIOUG66614-F09|BOLD:ACF7240
Scelionidae|GMPJA6036-21|BIOUG66617-E03|BOLD:ADP4310
Hymenoptera|GMPJA4762-21|BIOUG66605-A12
Scelionidae|GMPJA10695-21|BIOUG70237-C10|BOLD:AEA2258
Scelionidae|GMPJA9917-21|BIOUG66657-H03|BOLD:AEA2258
Scelionidae|GMPJA5972-21|BIOUG66616-G10|BOLD:AEA2258
Scelionidae|GMPJA6107-21|BIOUG66618-C03|BOLD:AEJ8559
Scelionidae|GMPJA5969-21|BIOUG66616-G07|BOLD:AEJ1418
Scelionidae|GMPJA9666-21|BIOUG66655-C02|BOLD:ACW6017
Scelionidae|GMPJA3746-21|BIOUG65694-D05|BOLD:AEJ0525
Scelionidae|GMPJA9410-21|BIOUG66652-E07|BOLD:AEJ6483
Scelioninae|GMPJA9624-21|BIOUG66654-G07|BOLD:ABX9694
Scelionidae|GMPJA9337-21|BIOUG66651-G05|BOLD:AEJ4190
Hymenoptera|GMPJA6907-21|BIOUG66626-F07|BOLD:AEJ4094
Scelionidae|GMPJA6132-21|BIOUG66618-E04|BOLD:AEJ8498
Scelionidae|GMPJA2952-21|BIOUG65686-A07|BOLD:AEJ5128
Scelionidae|GMPJA2870-21|BIOUG65685-F06|BOLD:AEJ5128
Scelionidae|GMPJA2867-21|BIOUG65685-F03|BOLD:AEJ5128
Telenomus|GMPJA9742-21|BIOUG66656-A07|BOLD:AEK2289
Scelioninae|GMPJA6943-21|BIOUG66627-A08|BOLD:ACG6123
Scelioninae|GMPJA3715-21|BIOUG65694-A10|BOLD:ACE0130
Scelioninae|GMPJA9423-21|BIOUG66652-F08|BOLD:ACE0130
Scelioninae|GMPJA5774-21|BIOUG66614-G02|BOLD:ACE0130
Scelionidae|GMPJA9289-21|BIOUG66651-C05|BOLD:AEJ7258
Scelionidae|GMPJA9773-21|BIOUG66656-D02|BOLD:AEJ7258
Scelionidae|GMPJA6974-21|BIOUG66627-D03|BOLD:AEJ7258
Scelionidae|GMPJA6019-21|BIOUG66617-C10|BOLD:AEJ1803
Scelionidae|GMPJA6080-21|BIOUG66617-H11|BOLD:AEJ1054
Scelionidae|GMPJA6079-21|BIOUG66617-H10|BOLD:AEJ1054
Scelionidae|GMPJA6124-21|BIOUG66618-D08|BOLD:AEJ1054
Scelioninae|GMPJA9559-21|BIOUG66654-B02|BOLD:ACG6414
Scelioninae|GMPJA6950-21|BIOUG66627-B03|BOLD:ACG6414
Scelioninae|GMPJA6945-21|BIOUG66627-A10|BOLD:ACG6414
Scelioninae|GMPJA5754-21|BIOUG66614-E06|BOLD:ACG6414
Scelioninae|GMPJA4720-21|BIOUG66604-F05|BOLD:ACP6925
Scelioninae|GMPJA897-21|BIOUG65664-H04|BOLD:ACP6925
Scelioninae|GMPJA4681-21|BIOUG66604-C02|BOLD:ACP6925
Scelionidae|GMPJA9648-21|BIOUG66655-A08|BOLD:ACV5634
Scelionidae|GMPJA9515-21|BIOUG66653-F05|BOLD:ACV5634
Scelionidae|GMPJA9443-21|BIOUG66652-H04|BOLD:ACW8155
Scelionidae|GMPJA9414-21|BIOUG66652-E11|BOLD:ADV7316
Telenomus|GMPJA9620-21|BIOUG66654-G03|BOLD:ACI4761
Telenomus|GMPJA9375-21|BIOUG66652-B08|BOLD:ACI4761
Scelionidae|GMPJA9313-21|BIOUG66651-E05|BOLD:AEJ4668
Telenomus|GMPJA9912-21|BIOUG66657-G10|BOLD:ABY0168
Telenomus|GMPJA9763-21|BIOUG66656-C04|BOLD:ABY0168
Telenomus|GMPJA9334-21|BIOUG66651-G02|BOLD:ABY0168
Telenomus|GMPJA10068-21|BIOUG66659-D12|BOLD:ABY0168
Telenomus|GMPJA9318-21|BIOUG66651-E10|BOLD:ABY0168
Telenomus|GMPJA9849-21|BIOUG66657-B07|BOLD:ABY0168
Telenomus|GMPJA9827-21|BIOUG66656-H08|BOLD:ABY0168
Telenomus|GMPJA9826-21|BIOUG66656-H07|BOLD:ABY0168
Telenomus|GMPJA9727-21|BIOUG66655-H03|BOLD:ABY0168
Telenomus|GMPJA9669-21|BIOUG66655-C05|BOLD:ABY0168
Telenomus|GMPJA9617-21|BIOUG66654-F12|BOLD:ABY0168
Telenomus|GMPJA9529-21|BIOUG66653-G07|BOLD:ABY0168

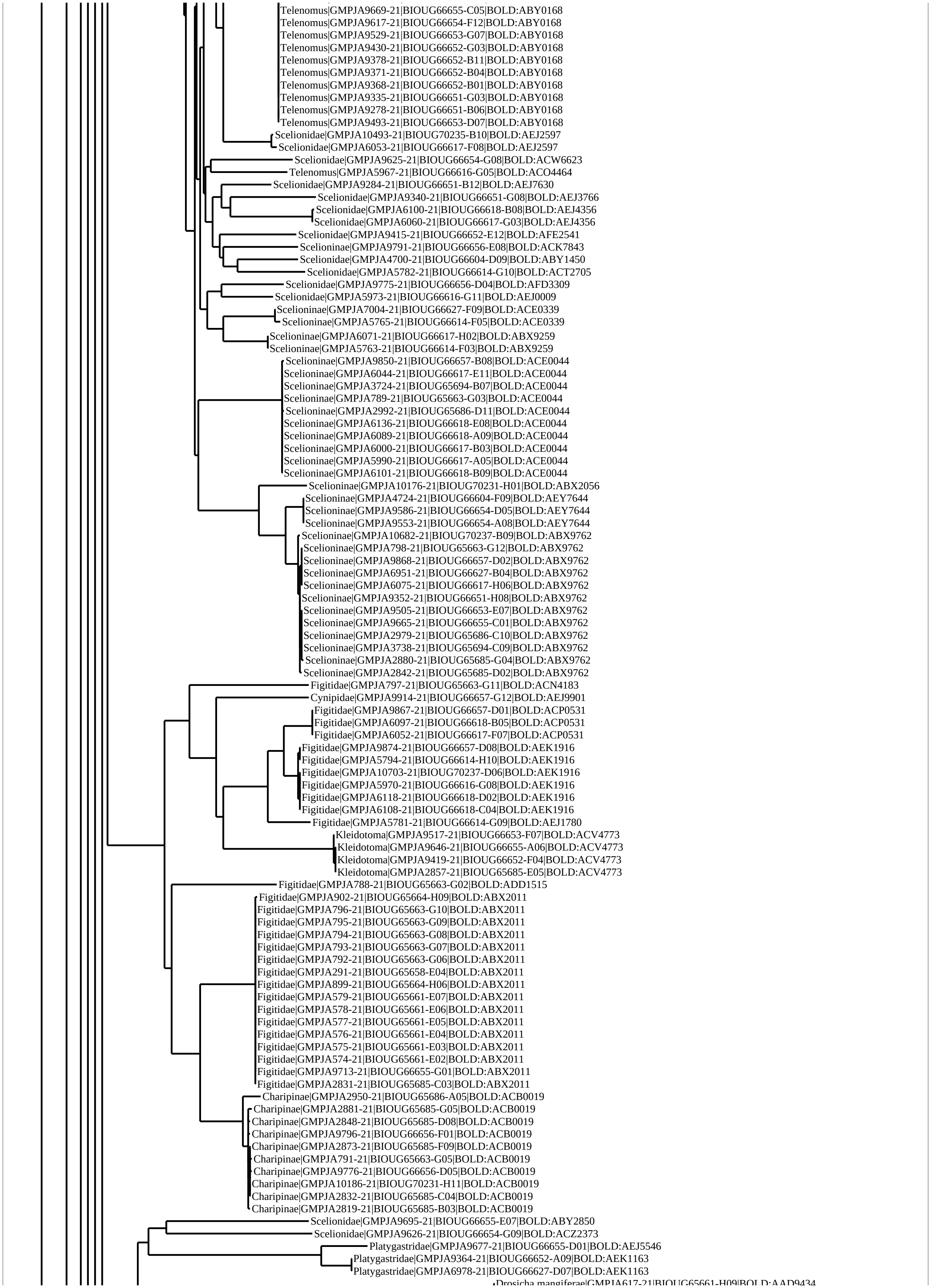
Telenomus|GMPJA9669-21|BIOUG66655-C05|BOLD:ABY0168
Telenomus|GMPJA9617-21|BIOUG66654-F12|BOLD:ABY0168
Telenomus|GMPJA9529-21|BIOUG66653-G07|BOLD:ABY0168
Telenomus|GMPJA9430-21|BIOUG66652-G03|BOLD:ABY0168
Telenomus|GMPJA9378-21|BIOUG66652-B11|BOLD:ABY0168
Telenomus|GMPJA9371-21|BIOUG66652-B04|BOLD:ABY0168
Telenomus|GMPJA9368-21|BIOUG66652-B01|BOLD:ABY0168
Telenomus|GMPJA9335-21|BIOUG66651-G03|BOLD:ABY0168
Telenomus|GMPJA9278-21|BIOUG66651-B06|BOLD:ABY0168
Telenomus|GMPJA9493-21|BIOUG66653-D07|BOLD:ABY0168
Scelionidae|GMPJA10493-21|BIOUG70235-B10|BOLD:AEJ2597
Scelionidae|GMPJA6053-21|BIOUG66617-F08|BOLD:AEJ2597
Scelionidae|GMPJA9625-21|BIOUG66654-G08|BOLD:ACW6623
Telenomus|GMPJA5967-21|BIOUG66616-G05|BOLD:ACO4464
Scelionidae|GMPJA9284-21|BIOUG66651-B12|BOLD:AEJ7630
Scelionidae|GMPJA9340-21|BIOUG66651-G08|BOLD:AEJ3766
Scelionidae|GMPJA6100-21|BIOUG66618-B08|BOLD:AEJ4356
Scelionidae|GMPJA6060-21|BIOUG66617-G03|BOLD:AEJ4356
Scelionidae|GMPJA9415-21|BIOUG66652-E12|BOLD:AFE2541
Scelioninae|GMPJA9791-21|BIOUG66656-E08|BOLD:ACK7843
Scelionidae|GMPJA4700-21|BIOUG66604-D09|BOLD:ABY1450
Scelionidae|GMPJA5782-21|BIOUG66614-G10|BOLD:ACT2705
Scelionidae|GMPJA9775-21|BIOUG66656-D04|BOLD:AFD3309
Scelionidae|GMPJA5973-21|BIOUG66616-G11|BOLD:AEJ0009
Scelioninae|GMPJA7004-21|BIOUG66627-F09|BOLD:ACE0339
Scelioninae|GMPJA5765-21|BIOUG66614-F05|BOLD:ACE0339
Scelioninae|GMPJA6071-21|BIOUG66617-H02|BOLD:ABX9259
Scelioninae|GMPJA5763-21|BIOUG66614-F03|BOLD:ABX9259
Scelioninae|GMPJA9850-21|BIOUG66657-B08|BOLD:ACE0044
Scelioninae|GMPJA6044-21|BIOUG66617-E11|BOLD:ACE0044
Scelioninae|GMPJA3724-21|BIOUG65694-B07|BOLD:ACE0044
Scelioninae|GMPJA789-21|BIOUG65663-G03|BOLD:ACE0044
Scelioninae|GMPJA2992-21|BIOUG65686-D11|BOLD:ACE0044
Scelioninae|GMPJA6136-21|BIOUG66618-E08|BOLD:ACE0044
Scelioninae|GMPJA6089-21|BIOUG66618-A09|BOLD:ACE0044
Scelioninae|GMPJA6000-21|BIOUG66617-B03|BOLD:ACE0044
Scelioninae|GMPJA5990-21|BIOUG66617-A05|BOLD:ACE0044
Scelioninae|GMPJA6101-21|BIOUG66618-B09|BOLD:ACE0044
Scelioninae|GMPJA10176-21|BIOUG70231-H01|BOLD:ABX2056
Scelioninae|GMPJA4724-21|BIOUG66604-F09|BOLD:AEY7644
Scelioninae|GMPJA9586-21|BIOUG66654-D05|BOLD:AEY7644
Scelioninae|GMPJA9553-21|BIOUG66654-A08|BOLD:AEY7644
Scelioninae|GMPJA10682-21|BIOUG70237-B09|BOLD:ABX9762
Scelioninae|GMPJA798-21|BIOUG65663-G12|BOLD:ABX9762
Scelioninae|GMPJA9868-21|BIOUG66657-D02|BOLD:ABX9762
Scelioninae|GMPJA6951-21|BIOUG66627-B04|BOLD:ABX9762
Scelioninae|GMPJA6075-21|BIOUG66617-H06|BOLD:ABX9762
Scelioninae|GMPJA9352-21|BIOUG66651-H08|BOLD:ABX9762
Scelioninae|GMPJA9505-21|BIOUG66653-E07|BOLD:ABX9762
Scelioninae|GMPJA9665-21|BIOUG66655-C01|BOLD:ABX9762
Scelioninae|GMPJA2979-21|BIOUG65686-C10|BOLD:ABX9762
Scelioninae|GMPJA3738-21|BIOUG65694-C09|BOLD:ABX9762
Scelioninae|GMPJA2880-21|BIOUG65685-G04|BOLD:ABX9762
Scelioninae|GMPJA2842-21|BIOUG65685-D02|BOLD:ABX9762
Figitidae|GMPJA797-21|BIOUG65663-G11|BOLD:ACN4183
Cynipidae|GMPJA9914-21|BIOUG66657-G12|BOLD:AEJ9901
Figitidae|GMPJA9867-21|BIOUG66657-D01|BOLD:ACP0531
Figitidae|GMPJA6097-21|BIOUG66618-B05|BOLD:ACP0531
Figitidae|GMPJA6052-21|BIOUG66617-F07|BOLD:ACP0531
Figitidae|GMPJA9874-21|BIOUG66657-D08|BOLD:AEK1916
Figitidae|GMPJA5794-21|BIOUG66614-H10|BOLD:AEK1916
Figitidae|GMPJA10703-21|BIOUG70237-D06|BOLD:AEK1916
Figitidae|GMPJA5970-21|BIOUG66616-G08|BOLD:AEK1916
Figitidae|GMPJA6118-21|BIOUG66618-D02|BOLD:AEK1916
Figitidae|GMPJA6108-21|BIOUG66618-C04|BOLD:AEK1916
Figitidae|GMPJA5781-21|BIOUG66614-G09|BOLD:AEJ1780
Kleidotoma|GMPJA9517-21|BIOUG66653-F07|BOLD:ACV4773
Kleidotoma|GMPJA9646-21|BIOUG66655-A06|BOLD:ACV4773
Kleidotoma|GMPJA9419-21|BIOUG66652-F04|BOLD:ACV4773
Kleidotoma|GMPJA2857-21|BIOUG65685-E05|BOLD:ACV4773
Figitidae|GMPJA788-21|BIOUG65663-G02|BOLD:ADD1515
Figitidae|GMPJA902-21|BIOUG65664-H09|BOLD:ABX2011
Figitidae|GMPJA796-21|BIOUG65663-G10|BOLD:ABX2011
Figitidae|GMPJA795-21|BIOUG65663-G09|BOLD:ABX2011
Figitidae|GMPJA794-21|BIOUG65663-G08|BOLD:ABX2011
Figitidae|GMPJA793-21|BIOUG65663-G07|BOLD:ABX2011
Figitidae|GMPJA792-21|BIOUG65663-G06|BOLD:ABX2011
Figitidae|GMPJA291-21|BIOUG65658-E04|BOLD:ABX2011
Figitidae|GMPJA899-21|BIOUG65664-H06|BOLD:ABX2011
Figitidae|GMPJA579-21|BIOUG65661-E07|BOLD:ABX2011
Figitidae|GMPJA578-21|BIOUG65661-E06|BOLD:ABX2011
Figitidae|GMPJA577-21|BIOUG65661-E05|BOLD:ABX2011
Figitidae|GMPJA576-21|BIOUG65661-E04|BOLD:ABX2011
Figitidae|GMPJA575-21|BIOUG65661-E03|BOLD:ABX2011
Figitidae|GMPJA574-21|BIOUG65661-E02|BOLD:ABX2011
Figitidae|GMPJA9713-21|BIOUG66655-G01|BOLD:ABX2011
Figitidae|GMPJA2831-21|BIOUG65685-C03|BOLD:ABX2011
Charipinae|GMPJA2950-21|BIOUG65686-A05|BOLD:ACB0019
Charipinae|GMPJA2881-21|BIOUG65685-G05|BOLD:ACB0019
Charipinae|GMPJA2848-21|BIOUG65685-D08|BOLD:ACB0019
Charipinae|GMPJA9796-21|BIOUG66656-F01|BOLD:ACB0019
Charipinae|GMPJA2873-21|BIOUG65685-F09|BOLD:ACB0019
Charipinae|GMPJA791-21|BIOUG65663-G05|BOLD:ACB0019
Charipinae|GMPJA9776-21|BIOUG66656-D05|BOLD:ACB0019
Charipinae|GMPJA10186-21|BIOUG70231-H11|BOLD:ACB0019
Charipinae|GMPJA2832-21|BIOUG65685-C04|BOLD:ACB0019
Charipinae|GMPJA2819-21|BIOUG65685-B03|BOLD:ACB0019
Scelionidae|GMPJA9695-21|BIOUG66655-E07|BOLD:ABY2850
Scelionidae|GMPJA9626-21|BIOUG66654-G09|BOLD:ACZ2373
Platygastridae|GMPJA9677-21|BIOUG66655-D01|BOLD:AEJ5546
Platygastridae|GMPJA9364-21|BIOUG66652-A09|BOLD:AEK1163
Platygastridae|GMPJA6978-21|BIOUG66627-D07|BOLD:AEK1163
Drosicha mangiferae|GMPJA617-21|BIOUG65661-H09|BOLD:AAD9434

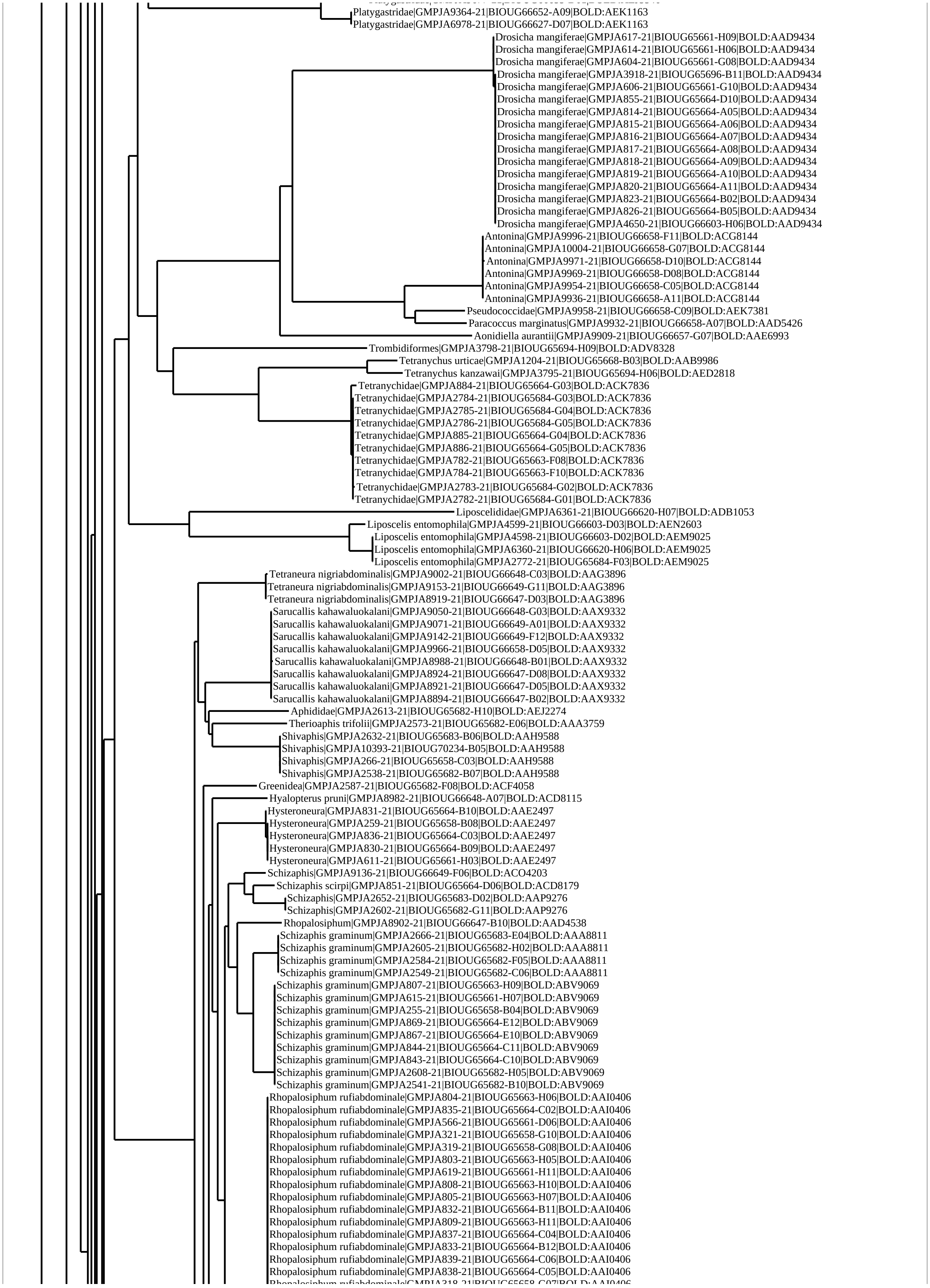
Platygastridae|GMPJA9364-21|BIOUG66652-A09|BOLD:AEK1163
Platygastridae|GMPJA6978-21|BIOUG66627-D07|BOLD:AEK1163
Drosicha mangiferae|GMPJA617-21|BIOUG65661-H09|BOLD:AAD9434
Drosicha mangiferae|GMPJA614-21|BIOUG65661-H06|BOLD:AAD9434
Drosicha mangiferae|GMPJA604-21|BIOUG65661-G08|BOLD:AAD9434
Drosicha mangiferae|GMPJA3918-21|BIOUG65696-B11|BOLD:AAD9434
Drosicha mangiferae|GMPJA606-21|BIOUG65661-G10|BOLD:AAD9434
Drosicha mangiferae|GMPJA855-21|BIOUG65664-D10|BOLD:AAD9434
Drosicha mangiferae|GMPJA814-21|BIOUG65664-A05|BOLD:AAD9434
Drosicha mangiferae|GMPJA815-21|BIOUG65664-A06|BOLD:AAD9434
Drosicha mangiferae|GMPJA816-21|BIOUG65664-A07|BOLD:AAD9434
Drosicha mangiferae|GMPJA817-21|BIOUG65664-A08|BOLD:AAD9434
Drosicha mangiferae|GMPJA818-21|BIOUG65664-A09|BOLD:AAD9434
Drosicha mangiferae|GMPJA819-21|BIOUG65664-A10|BOLD:AAD9434
Drosicha mangiferae|GMPJA820-21|BIOUG65664-A11|BOLD:AAD9434
Drosicha mangiferae|GMPJA823-21|BIOUG65664-B02|BOLD:AAD9434
Drosicha mangiferae|GMPJA826-21|BIOUG65664-B05|BOLD:AAD9434
Drosicha mangiferae|GMPJA4650-21|BIOUG66603-H06|BOLD:AAD9434
Antonina|GMPJA9996-21|BIOUG66658-F11|BOLD:ACG8144
Antonina|GMPJA10004-21|BIOUG66658-G07|BOLD:ACG8144
Antonina|GMPJA9971-21|BIOUG66658-D10|BOLD:ACG8144
Antonina|GMPJA9969-21|BIOUG66658-D08|BOLD:ACG8144
Antonina|GMPJA9954-21|BIOUG66658-C05|BOLD:ACG8144
Antonina|GMPJA9936-21|BIOUG66658-A11|BOLD:ACG8144
Pseudococcidae|GMPJA9958-21|BIOUG66658-C09|BOLD:AEK7381
Paracoccus marginatus|GMPJA9932-21|BIOUG66658-A07|BOLD:AAD5426
Aonidiella aurantii|GMPJA9909-21|BIOUG66657-G07|BOLD:AAE6993
Trombidiformes|GMPJA3798-21|BIOUG65694-H09|BOLD:ADV8328
Tetranychus urticae|GMPJA1204-21|BIOUG65668-B03|BOLD:AAB9986
Tetranychus kanzawai|GMPJA3795-21|BIOUG65694-H06|BOLD:AED2818
Tetranychidae|GMPJA884-21|BIOUG65664-G03|BOLD:ACK7836
Tetranychidae|GMPJA2784-21|BIOUG65684-G03|BOLD:ACK7836
Tetranychidae|GMPJA2785-21|BIOUG65684-G04|BOLD:ACK7836
Tetranychidae|GMPJA2786-21|BIOUG65684-G05|BOLD:ACK7836
Tetranychidae|GMPJA885-21|BIOUG65664-G04|BOLD:ACK7836
Tetranychidae|GMPJA886-21|BIOUG65664-G05|BOLD:ACK7836
Tetranychidae|GMPJA782-21|BIOUG65663-F08|BOLD:ACK7836
Tetranychidae|GMPJA784-21|BIOUG65663-F10|BOLD:ACK7836
Tetranychidae|GMPJA2783-21|BIOUG65684-G02|BOLD:ACK7836
Tetranychidae|GMPJA2782-21|BIOUG65684-G01|BOLD:ACK7836
Liposcelididae|GMPJA6361-21|BIOUG66620-H07|BOLD:ADB1053
Liposcelis entomophila|GMPJA4599-21|BIOUG66603-D03|BOLD:AEN2603
Liposcelis entomophila|GMPJA4598-21|BIOUG66603-D02|BOLD:AEM9025
Liposcelis entomophila|GMPJA6360-21|BIOUG66620-H06|BOLD:AEM9025
Liposcelis entomophila|GMPJA2772-21|BIOUG65684-F03|BOLD:AEM9025
Tetraneura nigriabdominalis|GMPJA9002-21|BIOUG66648-C03|BOLD:AAG3896
Tetraneura nigriabdominalis|GMPJA9153-21|BIOUG66649-G11|BOLD:AAG3896
Tetraneura nigriabdominalis|GMPJA8919-21|BIOUG66647-D03|BOLD:AAG3896
Sarucallis kahawaluokalani|GMPJA9050-21|BIOUG66648-G03|BOLD:AAX9332
Sarucallis kahawaluokalani|GMPJA9071-21|BIOUG66649-A01|BOLD:AAX9332
Sarucallis kahawaluokalani|GMPJA9142-21|BIOUG66649-F12|BOLD:AAX9332
Sarucallis kahawaluokalani|GMPJA9966-21|BIOUG66658-D05|BOLD:AAX9332
Sarucallis kahawaluokalani|GMPJA8988-21|BIOUG66648-B01|BOLD:AAX9332
Sarucallis kahawaluokalani|GMPJA8924-21|BIOUG66647-D08|BOLD:AAX9332
Sarucallis kahawaluokalani|GMPJA8921-21|BIOUG66647-D05|BOLD:AAX9332
Sarucallis kahawaluokalani|GMPJA8894-21|BIOUG66647-B02|BOLD:AAX9332
Aphididae|GMPJA2613-21|BIOUG65682-H10|BOLD:AEJ2274
Therioaphis trifolii|GMPJA2573-21|BIOUG65682-E06|BOLD:AAA3759
Shivaphis|GMPJA2632-21|BIOUG65683-B06|BOLD:AAH9588
Shivaphis|GMPJA10393-21|BIOUG70234-B05|BOLD:AAH9588
Shivaphis|GMPJA266-21|BIOUG65658-C03|BOLD:AAH9588
Shivaphis|GMPJA2538-21|BIOUG65682-B07|BOLD:AAH9588
Greenidea|GMPJA2587-21|BIOUG65682-F08|BOLD:ACF4058
Hyalopterus pruni|GMPJA8982-21|BIOUG66648-A07|BOLD:ACD8115
Hysteroneura|GMPJA831-21|BIOUG65664-B10|BOLD:AAE2497
Hysteroneura|GMPJA259-21|BIOUG65658-B08|BOLD:AAE2497
Hysteroneura|GMPJA836-21|BIOUG65664-C03|BOLD:AAE2497
Hysteroneura|GMPJA830-21|BIOUG65664-B09|BOLD:AAE2497
Hysteroneura|GMPJA611-21|BIOUG65661-H03|BOLD:AAE2497
Schizaphis|GMPJA9136-21|BIOUG66649-F06|BOLD:ACO4203
Schizaphis scirpi|GMPJA851-21|BIOUG65664-D06|BOLD:ACD8179
Schizaphis|GMPJA2652-21|BIOUG65683-D02|BOLD:AAP9276
Schizaphis|GMPJA2602-21|BIOUG65682-G11|BOLD:AAP9276
Rhopalosiphum|GMPJA8902-21|BIOUG66647-B10|BOLD:AAD4538
Schizaphis graminum|GMPJA2666-21|BIOUG65683-E04|BOLD:AAA8811
Schizaphis graminum|GMPJA2605-21|BIOUG65682-H02|BOLD:AAA8811
Schizaphis graminum|GMPJA2584-21|BIOUG65682-F05|BOLD:AAA8811
Schizaphis graminum|GMPJA2549-21|BIOUG65682-C06|BOLD:AAA8811
Schizaphis graminum|GMPJA807-21|BIOUG65663-H09|BOLD:ABV9069
Schizaphis graminum|GMPJA615-21|BIOUG65661-H07|BOLD:ABV9069
Schizaphis graminum|GMPJA255-21|BIOUG65658-B04|BOLD:ABV9069
Schizaphis graminum|GMPJA869-21|BIOUG65664-E12|BOLD:ABV9069
Schizaphis graminum|GMPJA867-21|BIOUG65664-E10|BOLD:ABV9069
Schizaphis graminum|GMPJA844-21|BIOUG65664-C11|BOLD:ABV9069
Schizaphis graminum|GMPJA843-21|BIOUG65664-C10|BOLD:ABV9069
Schizaphis graminum|GMPJA2608-21|BIOUG65682-H05|BOLD:ABV9069
Schizaphis graminum|GMPJA2541-21|BIOUG65682-B10|BOLD:ABV9069
Rhopalosiphum rufiabdominale|GMPJA804-21|BIOUG65663-H06|BOLD:AAI0406
Rhopalosiphum rufiabdominale|GMPJA835-21|BIOUG65664-C02|BOLD:AAI0406
Rhopalosiphum rufiabdominale|GMPJA566-21|BIOUG65661-D06|BOLD:AAI0406
Rhopalosiphum rufiabdominale|GMPJA321-21|BIOUG65658-G10|BOLD:AAI0406
Rhopalosiphum rufiabdominale|GMPJA319-21|BIOUG65658-G08|BOLD:AAI0406
Rhopalosiphum rufiabdominale|GMPJA803-21|BIOUG65663-H05|BOLD:AAI0406
Rhopalosiphum rufiabdominale|GMPJA619-21|BIOUG65661-H11|BOLD:AAI0406
Rhopalosiphum rufiabdominale|GMPJA808-21|BIOUG65663-H10|BOLD:AAI0406
Rhopalosiphum rufiabdominale|GMPJA805-21|BIOUG65663-H07|BOLD:AAI0406
Rhopalosiphum rufiabdominale|GMPJA832-21|BIOUG65664-B11|BOLD:AAI0406
Rhopalosiphum rufiabdominale|GMPJA809-21|BIOUG65663-H11|BOLD:AAI0406
Rhopalosiphum rufiabdominale|GMPJA837-21|BIOUG65664-C04|BOLD:AAI0406
Rhopalosiphum rufiabdominale|GMPJA833-21|BIOUG65664-B12|BOLD:AAI0406
Rhopalosiphum rufiabdominale|GMPJA839-21|BIOUG65664-C06|BOLD:AAI0406
Rhopalosiphum rufiabdominale|GMPJA838-21|BIOUG65664-C05|BOLD:AAI0406

[illegible]
Rhopalosiphum rufiabdominale|GMPJA839-21|BIOUG65664-C06|BOLD:AAI0406
Rhopalosiphum rufiabdominale|GMPJA838-21|BIOUG65664-C05|BOLD:AAI0406
Rhopalosiphum rufiabdominale|GMPJA318-21|BIOUG65658-G07|BOLD:AAI0406
Rhopalosiphum rufiabdominale|GMPJA316-21|BIOUG65658-G05|BOLD:AAI0406
Rhopalosiphum rufiabdominale|GMPJA265-21|BIOUG65658-C02|BOLD:AAI0406
Rhopalosiphum rufiabdominale|GMPJA842-21|BIOUG65664-C09|BOLD:AAI0406
Rhopalosiphum rufiabdominale|GMPJA569-21|BIOUG65661-D09|BOLD:AAI0406
Rhopalosiphum rufiabdominale|GMPJA608-21|BIOUG65661-G12|BOLD:AAI0406
Rhopalosiphum rufiabdominale|GMPJA605-21|BIOUG65661-G09|BOLD:AAI0406
Rhopalosiphum rufiabdominale|GMPJA602-21|BIOUG65661-G06|BOLD:AAI0406
Rhopalosiphum rufiabdominale|GMPJA9162-21|BIOUG66649-H08|BOLD:AAI0406
Rhopalosiphum rufiabdominale|GMPJA9159-21|BIOUG66649-H05|BOLD:AAI0406
Rhopalosiphum rufiabdominale|GMPJA9121-21|BIOUG66649-E03|BOLD:AAI0406
Rhopalosiphum rufiabdominale|GMPJA9020-21|BIOUG66648-D09|BOLD:AAI0406
Rhopalosiphum rufiabdominale|GMPJA616-21|BIOUG65661-H08|BOLD:AAI0406
Rhopalosiphum rufiabdominale|GMPJA329-21|BIOUG65658-H06|BOLD:AAI0406
Rhopalosiphum rufiabdominale|GMPJA8942-21|BIOUG66647-F02|BOLD:AAI0406
Rhopalosiphum rufiabdominale|GMPJA9069-21|BIOUG66648-H10|BOLD:AAI0406
Rhopalosiphum rufiabdominale|GMPJA9148-21|BIOUG66649-G06|BOLD:AAI0406
Rhopalosiphum rufiabdominale|GMPJA9045-21|BIOUG66648-F10|BOLD:AAI0406
Rhopalosiphum rufiabdominale|GMPJA9005-21|BIOUG66648-C06|BOLD:AAI0406
Rhopalosiphum rufiabdominale|GMPJA8985-21|BIOUG66648-A10|BOLD:AAI0406
Rhopalosiphum rufiabdominale|GMPJA8971-21|BIOUG66647-H07|BOLD:AAI0406
Rhopalosiphum rufiabdominale|GMPJA8904-21|BIOUG66647-B12|BOLD:AAI0406
Rhopalosiphum padi|GMPJA2631-21|BIOUG65683-B05|BOLD:ACF2924
Rhopalosiphum padi|GMPJA864-21|BIOUG65664-E07|BOLD:ACF2924
Rhopalosiphum padi|GMPJA2634-21|BIOUG65683-B08|BOLD:ACF2924
Rhopalosiphum padi|GMPJA2589-21|BIOUG65682-F10|BOLD:ACF2924
Rhopalosiphum padi|GMPJA2578-21|BIOUG65682-E11|BOLD:ACF2924
Rhopalosiphum padi|GMPJA841-21|BIOUG65664-C08|BOLD:ACF2924
Rhopalosiphum padi|GMPJA2648-21|BIOUG65683-C10|BOLD:ACF2924
Rhopalosiphum padi|GMPJA2653-21|BIOUG65683-D03|BOLD:ACF2924
Rhopalosiphum padi|GMPJA2663-21|BIOUG65683-E01|BOLD:ACF2924
Rhopalosiphum padi|GMPJA2667-21|BIOUG65683-E05|BOLD:ACF2924
Rhopalosiphum padi|GMPJA2690-21|BIOUG65683-G04|BOLD:ACF2924
Rhopalosiphum padi|GMPJA2692-21|BIOUG65683-G06|BOLD:ACF2924
Rhopalosiphum padi|GMPJA848-21|BIOUG65664-D03|BOLD:ACF2924
Rhopalosiphum padi|GMPJA834-21|BIOUG65664-C01|BOLD:ACF2924
Rhopalosiphum padi|GMPJA3001-21|BIOUG65686-E08|BOLD:ACF2924
Rhopalosiphum padi|GMPJA2639-21|BIOUG65683-C01|BOLD:ACF2924
Rhopalosiphum padi|GMPJA2626-21|BIOUG65683-A12|BOLD:ACF2924
Rhopalosiphum padi|GMPJA2623-21|BIOUG65683-A09|BOLD:ACF2924
Rhopalosiphum padi|GMPJA2622-21|BIOUG65683-A08|BOLD:ACF2924
Rhopalosiphum padi|GMPJA2619-21|BIOUG65683-A05|BOLD:ACF2924
Rhopalosiphum padi|GMPJA2618-21|BIOUG65683-A04|BOLD:ACF2924
Rhopalosiphum padi|GMPJA2617-21|BIOUG65683-A03|BOLD:ACF2924
Rhopalosiphum padi|GMPJA2610-21|BIOUG65682-H07|BOLD:ACF2924
Rhopalosiphum padi|GMPJA2609-21|BIOUG65682-H06|BOLD:ACF2924
Rhopalosiphum padi|GMPJA2607-21|BIOUG65682-H04|BOLD:ACF2924
Rhopalosiphum padi|GMPJA2579-21|BIOUG65682-E12|BOLD:ACF2924
Rhopalosiphum padi|GMPJA2572-21|BIOUG65682-E05|BOLD:ACF2924
Rhopalosiphum padi|GMPJA2565-21|BIOUG65682-D10|BOLD:ACF2924
Rhopalosiphum padi|GMPJA2554-21|BIOUG65682-C11|BOLD:ACF2924
Rhopalosiphum padi|GMPJA2606-21|BIOUG65682-H03|BOLD:AAA9899
Rhopalosiphum padi|GMPJA2551-21|BIOUG65682-C08|BOLD:AAA9899
Rhopalosiphum padi|GMPJA852-21|BIOUG65664-D07|BOLD:AAA9899
Rhopalosiphum padi|GMPJA2528-21|BIOUG65682-A09|BOLD:AAA9899
Rhopalosiphum padi|GMPJA2526-21|BIOUG65682-A07|BOLD:AAA9899
Rhopalosiphum padi|GMPJA2527-21|BIOUG65682-A08|BOLD:AAA9899
Rhopalosiphum padi|GMPJA2530-21|BIOUG65682-A11|BOLD:AAA9899
Rhopalosiphum padi|GMPJA2531-21|BIOUG65682-A12|BOLD:AAA9899
Rhopalosiphum padi|GMPJA2535-21|BIOUG65682-B04|BOLD:AAA9899
Rhopalosiphum padi|GMPJA2536-21|BIOUG65682-B05|BOLD:AAA9899
Rhopalosiphum padi|GMPJA2537-21|BIOUG65682-B06|BOLD:AAA9899
Rhopalosiphum padi|GMPJA2539-21|BIOUG65682-B08|BOLD:AAA9899
Rhopalosiphum padi|GMPJA2547-21|BIOUG65682-C04|BOLD:AAA9899
Rhopalosiphum padi|GMPJA2550-21|BIOUG65682-C07|BOLD:AAA9899
Rhopalosiphum padi|GMPJA2553-21|BIOUG65682-C10|BOLD:AAA9899
Rhopalosiphum padi|GMPJA2555-21|BIOUG65682-C12|BOLD:AAA9899
Rhopalosiphum padi|GMPJA2556-21|BIOUG65682-D01|BOLD:AAA9899
Rhopalosiphum padi|GMPJA2557-21|BIOUG65682-D02|BOLD:AAA9899
Rhopalosiphum padi|GMPJA2558-21|BIOUG65682-D03|BOLD:AAA9899
Rhopalosiphum padi|GMPJA2560-21|BIOUG65682-D05|BOLD:AAA9899
Rhopalosiphum padi|GMPJA2562-21|BIOUG65682-D07|BOLD:AAA9899
Rhopalosiphum padi|GMPJA2563-21|BIOUG65682-D08|BOLD:AAA9899
Rhopalosiphum padi|GMPJA2564-21|BIOUG65682-D09|BOLD:AAA9899
Rhopalosiphum padi|GMPJA2567-21|BIOUG65682-D12|BOLD:AAA9899
Rhopalosiphum padi|GMPJA2574-21|BIOUG65682-E07|BOLD:AAA9899
Rhopalosiphum padi|GMPJA2575-21|BIOUG65682-E08|BOLD:AAA9899
Rhopalosiphum padi|GMPJA2577-21|BIOUG65682-E10|BOLD:AAA9899
Rhopalosiphum padi|GMPJA2580-21|BIOUG65682-F01|BOLD:AAA9899
Rhopalosiphum padi|GMPJA2581-21|BIOUG65682-F02|BOLD:AAA9899
Rhopalosiphum padi|GMPJA2583-21|BIOUG65682-F04|BOLD:AAA9899
Rhopalosiphum padi|GMPJA2590-21|BIOUG65682-F11|BOLD:AAA9899
Rhopalosiphum padi|GMPJA2591-21|BIOUG65682-F12|BOLD:AAA9899
Rhopalosiphum padi|GMPJA2594-21|BIOUG65682-G03|BOLD:AAA9899
Rhopalosiphum padi|GMPJA2595-21|BIOUG65682-G04|BOLD:AAA9899
Rhopalosiphum padi|GMPJA2620-21|BIOUG65683-A06|BOLD:AAA9899
Rhopalosiphum padi|GMPJA2621-21|BIOUG65683-A07|BOLD:AAA9899
Rhopalosiphum padi|GMPJA2624-21|BIOUG65683-A10|BOLD:AAA9899
Rhopalosiphum padi|GMPJA2625-21|BIOUG65683-A11|BOLD:AAA9899
Rhopalosiphum padi|GMPJA2628-21|BIOUG65683-B02|BOLD:AAA9899
Rhopalosiphum padi|GMPJA2633-21|BIOUG65683-B07|BOLD:AAA9899
Rhopalosiphum padi|GMPJA2635-21|BIOUG65683-B09|BOLD:AAA9899
Rhopalosiphum padi|GMPJA2641-21|BIOUG65683-C03|BOLD:AAA9899
Rhopalosiphum padi|GMPJA2642-21|BIOUG65683-C04|BOLD:AAA9899
Rhopalosiphum padi|GMPJA2643-21|BIOUG65683-C05|BOLD:AAA9899
Rhopalosiphum padi|GMPJA2644-21|BIOUG65683-C06|BOLD:AAA9899
Rhopalosiphum padi|GMPJA2645-21|BIOUG65683-C07|BOLD:AAA9899
Rhopalosiphum padi|GMPJA2649-21|BIOUG65683-C11|BOLD:AAA9899
Rhopalosiphum padi|GMPJA2651-21|BIOUG65683-D01|BOLD:AAA9899
Rhopalosiphum padi|GMPJA2654-21|BIOUG65683-D04|BOLD:AAA9899
[illegible]

Rhopalosiphum padi|GMPJA2649-21|BIOUG65683-C11|BOLD:AAA9899
Rhopalosiphum padi|GMPJA2651-21|BIOUG65683-D01|BOLD:AAA9899
Rhopalosiphum padi|GMPJA2654-21|BIOUG65683-D04|BOLD:AAA9899
Rhopalosiphum padi|GMPJA2655-21|BIOUG65683-D05|BOLD:AAA9899
Rhopalosiphum padi|GMPJA2659-21|BIOUG65683-D09|BOLD:AAA9899
Rhopalosiphum padi|GMPJA2664-21|BIOUG65683-E02|BOLD:AAA9899
Rhopalosiphum padi|GMPJA2668-21|BIOUG65683-E06|BOLD:AAA9899
Rhopalosiphum padi|GMPJA2685-21|BIOUG65683-F11|BOLD:AAA9899
Rhopalosiphum padi|GMPJA3872-21|BIOUG65695-F12|BOLD:AAA9899
Rhopalosiphum padi|GMPJA856-21|BIOUG65664-D11|BOLD:AAA9899
Rhopalosiphum padi|GMPJA858-21|BIOUG65664-E01|BOLD:AAA9899
Rhopalosiphum padi|GMPJA2548-21|BIOUG65682-C05|BOLD:AAA9899
Rhopalosiphum padi|GMPJA2660-21|BIOUG65683-D10|BOLD:AAA9899
Rhopalosiphum padi|GMPJA2673-21|BIOUG65683-E11|BOLD:AAA9899
Rhopalosiphum padi|GMPJA2586-21|BIOUG65682-F07|BOLD:AAA9899
Rhopalosiphum padi|GMPJA2525-21|BIOUG65682-A06|BOLD:AAA9899
Rhopalosiphum padi|GMPJA2524-21|BIOUG65682-A05|BOLD:AAA9899
Rhopalosiphum padi|GMPJA2521-21|BIOUG65682-A02|BOLD:AAA9899
Melanaphis|GMPJA8972-21|BIOUG66647-H08|BOLD:AAK7235
Melanaphis|GMPJA8933-21|BIOUG66647-E05|BOLD:AAK7235
Melanaphis|GMPJA8970-21|BIOUG66647-H06|BOLD:AAK7235
Melanaphis|GMPJA9987-21|BIOUG66658-F02|BOLD:AAK7235
Melanaphis|GMPJA8917-21|BIOUG66647-D01|BOLD:AAK7235
Melanaphis|GMPJA9078-21|BIOUG66649-A08|BOLD:AAK7235
Melanaphis|GMPJA9139-21|BIOUG66649-F09|BOLD:AAK7235
Melanaphis|GMPJA9160-21|BIOUG66649-H06|BOLD:AAK7235
Melanaphis|GMPJA9044-21|BIOUG66648-F09|BOLD:AAK7235
Melanaphis|GMPJA7052-21|BIOUG66628-B10|BOLD:AAK7235
Melanaphis|GMPJA8903-21|BIOUG66647-B11|BOLD:AAK7235
Melanaphis|GMPJA8901-21|BIOUG66647-B09|BOLD:AAK7235
Melanaphis|GMPJA8900-21|BIOUG66647-B08|BOLD:AAK7235
Melanaphis|GMPJA8899-21|BIOUG66647-B07|BOLD:AAK7235
Melanaphis|GMPJA8896-21|BIOUG66647-B04|BOLD:AAK7235
Melanaphis|GMPJA8895-21|BIOUG66647-B03|BOLD:AAK7235
Melanaphis|GMPJA8881-21|BIOUG66647-A01|BOLD:AAK7235
Melanaphis|GMPJA7053-21|BIOUG66628-B11|BOLD:AAK7235
Melanaphis|GMPJA9103-21|BIOUG66649-C09|BOLD:AAK7235
Melanaphis|GMPJA9093-21|BIOUG66649-B11|BOLD:AAK7235
Melanaphis|GMPJA9147-21|BIOUG66649-G05|BOLD:AAK7235
Melanaphis|GMPJA9146-21|BIOUG66649-G04|BOLD:AAK7235
Melanaphis|GMPJA9145-21|BIOUG66649-G03|BOLD:AAK7235
Melanaphis|GMPJA9140-21|BIOUG66649-F10|BOLD:AAK7235
Melanaphis|GMPJA9108-21|BIOUG66649-D02|BOLD:AAK7235
Melanaphis|GMPJA9105-21|BIOUG66649-C11|BOLD:AAK7235
Melanaphis|GMPJA9088-21|BIOUG66649-B06|BOLD:AAK7235
Melanaphis|GMPJA9087-21|BIOUG66649-B05|BOLD:AAK7235
Melanaphis|GMPJA9073-21|BIOUG66649-A03|BOLD:AAK7235
Melanaphis|GMPJA9065-21|BIOUG66648-H06|BOLD:AAK7235
Melanaphis|GMPJA9061-21|BIOUG66648-H02|BOLD:AAK7235
Melanaphis|GMPJA9059-21|BIOUG66648-G12|BOLD:AAK7235
Melanaphis|GMPJA9055-21|BIOUG66648-G08|BOLD:AAK7235
Melanaphis|GMPJA9048-21|BIOUG66648-G01|BOLD:AAK7235
Melanaphis|GMPJA9042-21|BIOUG66648-F07|BOLD:AAK7235
Melanaphis|GMPJA9041-21|BIOUG66648-F06|BOLD:AAK7235
Melanaphis|GMPJA9031-21|BIOUG66648-E08|BOLD:AAK7235
Melanaphis|GMPJA9030-21|BIOUG66648-E07|BOLD:AAK7235
Melanaphis|GMPJA9027-21|BIOUG66648-E04|BOLD:AAK7235
Melanaphis|GMPJA9025-21|BIOUG66648-E02|BOLD:AAK7235
Melanaphis|GMPJA9021-21|BIOUG66648-D10|BOLD:AAK7235
Melanaphis|GMPJA9003-21|BIOUG66648-C04|BOLD:AAK7235
Melanaphis|GMPJA9001-21|BIOUG66648-C02|BOLD:AAK7235
Melanaphis|GMPJA8999-21|BIOUG66648-B12|BOLD:AAK7235
Melanaphis|GMPJA8986-21|BIOUG66648-A11|BOLD:AAK7235
Melanaphis|GMPJA8969-21|BIOUG66647-H05|BOLD:AAK7235
Melanaphis|GMPJA8966-21|BIOUG66647-H02|BOLD:AAK7235
Melanaphis|GMPJA8960-21|BIOUG66647-G08|BOLD:AAK7235
Melanaphis|GMPJA8959-21|BIOUG66647-G07|BOLD:AAK7235
Melanaphis|GMPJA8953-21|BIOUG66647-G01|BOLD:AAK7235
Melanaphis|GMPJA8948-21|BIOUG66647-F08|BOLD:AAK7235
Melanaphis|GMPJA8943-21|BIOUG66647-F03|BOLD:AAK7235
Melanaphis|GMPJA9126-21|BIOUG66649-E08|BOLD:AAK7235
Melanaphis|GMPJA9117-21|BIOUG66649-D11|BOLD:AAK7235
Melanaphis|GMPJA9156-21|BIOUG66649-H02|BOLD:AAK7235
Melanaphis|GMPJA9155-21|BIOUG66649-H01|BOLD:AAK7235
Melanaphis|GMPJA9161-21|BIOUG66649-H07|BOLD:AAK7235
Melanaphis|GMPJA9158-21|BIOUG66649-H04|BOLD:AAK7235
Melanaphis|GMPJA9164-21|BIOUG66649-H10|BOLD:AAK7235
Melanaphis|GMPJA9163-21|BIOUG66649-H09|BOLD:AAK7235
Melanaphis|GMPJA9222-21|BIOUG66650-E09|BOLD:AAK7235
Melanaphis|GMPJA9166-21|BIOUG66650-A01|BOLD:AAK7235
Melanaphis|GMPJA10015-21|BIOUG66658-H06|BOLD:AAK7235
Melanaphis|GMPJA9927-21|BIOUG66658-A02|BOLD:AAK7235
Melanaphis|GMPJA9935-21|BIOUG66658-A10|BOLD:AAK7235
Melanaphis|GMPJA9939-21|BIOUG66658-B02|BOLD:AAK7235
Melanaphis|GMPJA9953-21|BIOUG66658-C04|BOLD:AAK7235
Melanaphis|GMPJA9961-21|BIOUG66658-C12|BOLD:AAK7235
Melanaphis|GMPJA9977-21|BIOUG66658-E04|BOLD:AAK7235
Melanaphis|GMPJA10000-21|BIOUG66658-G03|BOLD:AAK7235
Melanaphis|GMPJA9135-21|BIOUG66649-F05|BOLD:AAK7235
Melanaphis|GMPJA10069-21|BIOUG66659-E01|BOLD:AAK7235
Melanaphis|GMPJA9951-21|BIOUG66658-C02|BOLD:AAK7235
Melanaphis|GMPJA7054-21|BIOUG66628-B12|BOLD:AAK7235
Melanaphis|GMPJA2637-21|BIOUG65683-B11|BOLD:AAK7235
Aphis|GMPJA2656-21|BIOUG65683-D06|BOLD:AAB7937
Aphis|GMPJA840-21|BIOUG65664-C07|BOLD:AAA4183
Aphis|GMPJA866-21|BIOUG65664-E09|BOLD:AAA4183
Aphis|GMPJA2570-21|BIOUG65682-E03|BOLD:AAA4183
Aphis|GMPJA806-21|BIOUG65663-H08|BOLD:AAA4183
Aphis|GMPJA618-21|BIOUG65661-H10|BOLD:AAA4183
Aphis|GMPJA2552-21|BIOUG65682-C09|BOLD:AAA4183
Aphis|GMPJA2603-21|BIOUG65682-G12|BOLD:AAA3070
Aphis|GMPJA2706-21|BIOUG65683-H08|BOLD:AAA3070
Aphis|GMPJA565-21|BIOUG65661-D05|BOLD:AAA3070
[illegible]

Aphis|GMPJA2603-21|BIOUG65682-G12|BOLD:AAA3070
Aphis|GMPJA2706-21|BIOUG65683-H08|BOLD:AAA3070
Aphis|GMPJA565-21|BIOUG65661-D05|BOLD:AAA3070
Aphis|GMPJA568-21|BIOUG65661-D08|BOLD:AAA3070
Aphis|GMPJA8974-21|BIOUG66647-H10|BOLD:AAA3070
Aphis|GMPJA2688-21|BIOUG65683-G02|BOLD:AAA3070
Aphis|GMPJA9141-21|BIOUG66649-F11|BOLD:AAA3070
Aphis|GMPJA8940-21|BIOUG66647-E12|BOLD:AAA3070
Aphis|GMPJA9086-21|BIOUG66649-B04|BOLD:AAA3070
Aphis|GMPJA2657-21|BIOUG65683-D07|BOLD:AAA3070
Aphis|GMPJA567-21|BIOUG65661-D07|BOLD:AAA3070
Aphis|GMPJA2545-21|BIOUG65682-C02|BOLD:AAA3070
Aphis|GMPJA2646-21|BIOUG65683-C08|BOLD:AAA3070
Aphis|GMPJA2647-21|BIOUG65683-C09|BOLD:AAA3070
Aphis|GMPJA2662-21|BIOUG65683-D12|BOLD:AAA3070
Aphis|GMPJA2708-21|BIOUG65683-H10|BOLD:AAA3070
Aphis|GMPJA5843-21|BIOUG66615-D12|BOLD:AAA3070
Aphis|GMPJA8893-21|BIOUG66647-B01|BOLD:AAA3070
Aphis|GMPJA8897-21|BIOUG66647-B05|BOLD:AAA3070
Aphis|GMPJA8925-21|BIOUG66647-D09|BOLD:AAA3070
Aphis|GMPJA8927-21|BIOUG66647-D11|BOLD:AAA3070
Aphis|GMPJA8928-21|BIOUG66647-D12|BOLD:AAA3070
Aphis|GMPJA8935-21|BIOUG66647-E07|BOLD:AAA3070
Aphis|GMPJA8939-21|BIOUG66647-E11|BOLD:AAA3070
Aphis|GMPJA8973-21|BIOUG66647-H09|BOLD:AAA3070
Aphis|GMPJA8984-21|BIOUG66648-A09|BOLD:AAA3070
Aphis|GMPJA8989-21|BIOUG66648-B02|BOLD:AAA3070
Aphis|GMPJA8990-21|BIOUG66648-B03|BOLD:AAA3070
Aphis|GMPJA8996-21|BIOUG66648-B09|BOLD:AAA3070
Aphis|GMPJA9006-21|BIOUG66648-C07|BOLD:AAA3070
Aphis|GMPJA9013-21|BIOUG66648-D02|BOLD:AAA3070
Aphis|GMPJA9026-21|BIOUG66648-E03|BOLD:AAA3070
Aphis|GMPJA9028-21|BIOUG66648-E05|BOLD:AAA3070
Aphis|GMPJA9037-21|BIOUG66648-F02|BOLD:AAA3070
Aphis|GMPJA9047-21|BIOUG66648-F12|BOLD:AAA3070
Aphis|GMPJA9049-21|BIOUG66648-G02|BOLD:AAA3070
Aphis|GMPJA9098-21|BIOUG66649-C04|BOLD:AAA3070
Aphis|GMPJA9104-21|BIOUG66649-C10|BOLD:AAA3070
Aphis|GMPJA9115-21|BIOUG66649-D09|BOLD:AAA3070
Aphis|GMPJA9116-21|BIOUG66649-D10|BOLD:AAA3070
Aphis|GMPJA9120-21|BIOUG66649-E02|BOLD:AAA3070
Aphis|GMPJA9122-21|BIOUG66649-E04|BOLD:AAA3070
Aphis|GMPJA9125-21|BIOUG66649-E07|BOLD:AAA3070
Aphis|GMPJA9133-21|BIOUG66649-F03|BOLD:AAA3070
Aphis|GMPJA9149-21|BIOUG66649-G07|BOLD:AAA3070
Aphis|GMPJA9154-21|BIOUG66649-G12|BOLD:AAA3070
Aphis|GMPJA9944-21|BIOUG66658-B07|BOLD:AAA3070
Aphis|GMPJA9997-21|BIOUG66658-F12|BOLD:AAA3070
Aphis|GMPJA10415-21|BIOUG70234-D03|BOLD:AAA3070
Aphis|GMPJA10425-21|BIOUG70234-E01|BOLD:AAA3070
Aphis|GMPJA10525-21|BIOUG70235-E06|BOLD:AAA3070
Aphis|GMPJA10733-21|BIOUG70237-F12|BOLD:AAA3070
Aphis|GMPJA249-21|BIOUG65658-A10|BOLD:AAA3070
Aphis|GMPJA256-21|BIOUG65658-B05|BOLD:AAA3070
Aphis|GMPJA257-21|BIOUG65658-B06|BOLD:AAA3070
Aphis|GMPJA258-21|BIOUG65658-B07|BOLD:AAA3070
Aphis|GMPJA260-21|BIOUG65658-B09|BOLD:AAA3070
Aphis|GMPJA261-21|BIOUG65658-B10|BOLD:AAA3070
Aphis|GMPJA317-21|BIOUG65658-G06|BOLD:AAA3070
Aphis|GMPJA263-21|BIOUG65658-B12|BOLD:AAA3070
Aphis|GMPJA322-21|BIOUG65658-G11|BOLD:AAA3070
Aphis|GMPJA2566-21|BIOUG65682-D11|BOLD:AAA3070
Aphis|GMPJA8961-21|BIOUG66647-G09|BOLD:AAA3070
Aphis|GMPJA8926-21|BIOUG66647-D10|BOLD:AAA3070
Aphis|GMPJA8898-21|BIOUG66647-B06|BOLD:AAA3070
Aphis|GMPJA2544-21|BIOUG65682-C01|BOLD:AAA3070
Aphis|GMPJA2543-21|BIOUG65682-B12|BOLD:AAA3070
Rhopalosiphum|GMPJA262-21|BIOUG65658-B11|BOLD:AAE3554
Aphis nerii|GMPJA320-21|BIOUG65658-G09|BOLD:AAC1372
Aphis nerii|GMPJA248-21|BIOUG65658-A09|BOLD:AAC1372
Aphis nerii|GMPJA2615-21|BIOUG65683-A01|BOLD:AAC1372
Aphis nerii|GMPJA2592-21|BIOUG65682-G01|BOLD:AAC1372
Aphis nerii|GMPJA2561-21|BIOUG65682-D06|BOLD:AAC1372
Aphis nerii|GMPJA2542-21|BIOUG65682-B11|BOLD:AAC1372
Myzus persicae|GMPJA827-21|BIOUG65664-B06|BOLD:AAA7683
Myzus persicae|GMPJA868-21|BIOUG65664-E11|BOLD:AAA7683
Myzus persicae|GMPJA2675-21|BIOUG65683-F01|BOLD:AAA7683
Aphididae|GMPJA9143-21|BIOUG66649-G01|BOLD:ADZ9938
Brevicoryne brassicae|GMPJA2640-21|BIOUG65683-C02|BOLD:AAD0145
Brevicoryne brassicae|GMPJA2629-21|BIOUG65683-B03|BOLD:AAD0145
Hyperomyzus carduellinus|GMPJA2585-21|BIOUG65682-F06|BOLD:ABZ3432
Hyperomyzus|GMPJA2600-21|BIOUG65682-G09|BOLD:AAB8566
Hyperomyzus|GMPJA2522-21|BIOUG65682-A03|BOLD:AAB8566
Sitobion avenae|GMPJA2533-21|BIOUG65682-B02|BOLD:AAB4894
Sitobion avenae|GMPJA2532-21|BIOUG65682-B01|BOLD:AAB4894
Sitobion avenae|GMPJA2601-21|BIOUG65682-G10|BOLD:AAB4894
Sitobion avenae|GMPJA2582-21|BIOUG65682-F03|BOLD:AAB4894
Sitobion avenae|GMPJA2665-21|BIOUG65683-E03|BOLD:AAB4894
Sitobion avenae|GMPJA2588-21|BIOUG65682-F09|BOLD:AAB4894
Sitobion avenae|GMPJA2650-21|BIOUG65683-C12|BOLD:AAB4894
Sitobion avenae|GMPJA2599-21|BIOUG65682-G08|BOLD:AAB4894
Sitobion avenae|GMPJA2576-21|BIOUG65682-E09|BOLD:AAB4894
Sitobion avenae|GMPJA2540-21|BIOUG65682-B09|BOLD:AAB4894
Sitobion avenae|GMPJA2614-21|BIOUG65682-H11|BOLD:AAB4894
Sitobion avenae|GMPJA2598-21|BIOUG65682-G07|BOLD:AAB4894
Sitobion avenae|GMPJA2597-21|BIOUG65682-G06|BOLD:AAB4894
Sitobion avenae|GMPJA2568-21|BIOUG65682-E01|BOLD:AAB4894
Sitobion avenae|GMPJA2534-21|BIOUG65682-B03|BOLD:AAB4894
Sitobion avenae|GMPJA2529-21|BIOUG65682-A10|BOLD:AAB4894
Sitobion avenae|GMPJA2616-21|BIOUG65683-A02|BOLD:AAB4894
Sitobion avenae|GMPJA2638-21|BIOUG65683-B12|BOLD:AAB4894
Sitobion avenae|GMPJA2596-21|BIOUG65682-G05|BOLD:AAB4894
Sitobion avenae|GMPJA2630-21|BIOUG65683-B04|BOLD:AAB4894

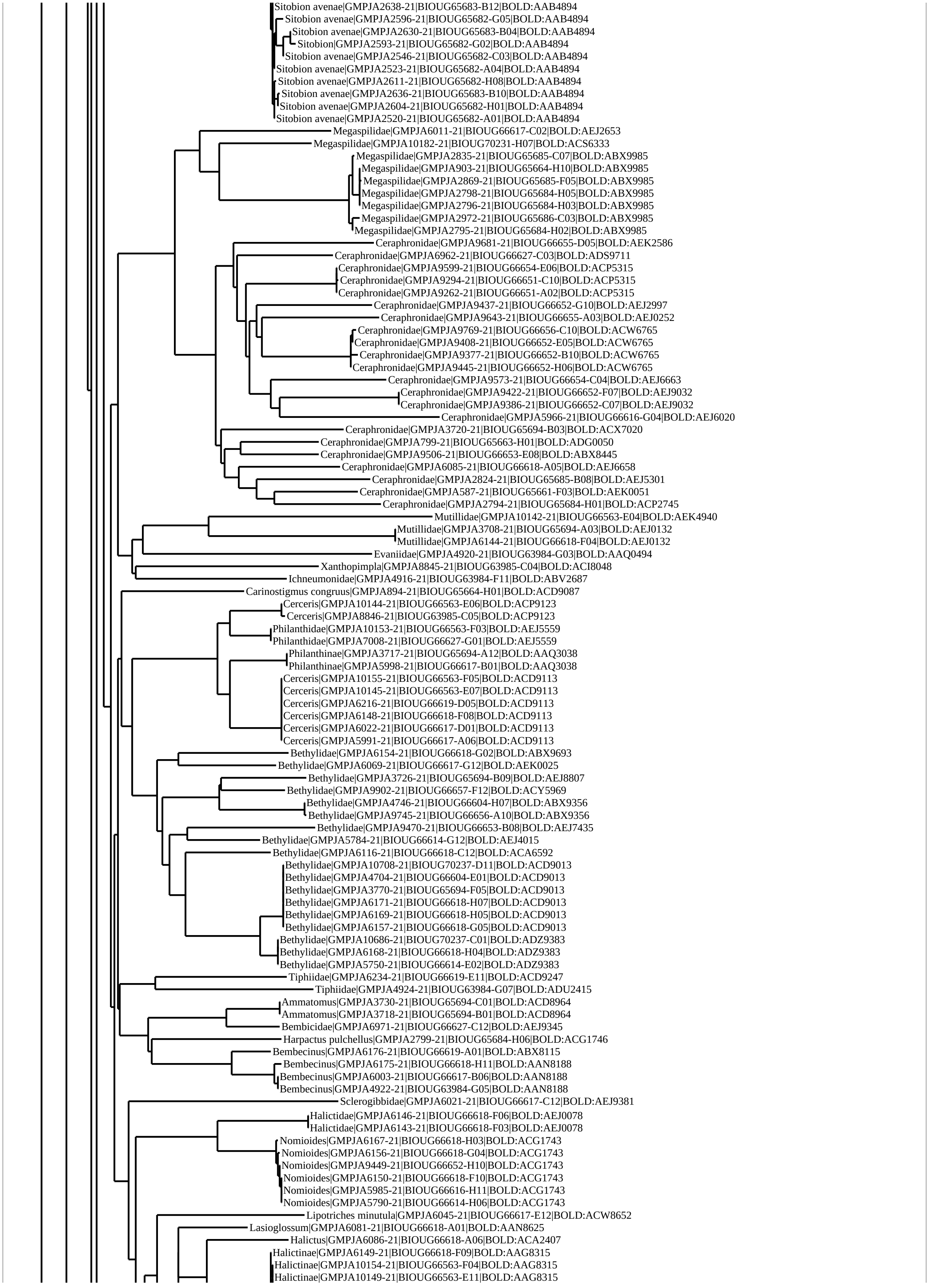
Sitobion avenae|GMPJA2638-21|BIOUG65683-B12|BOLD:AAB4894
Sitobion avenae|GMPJA2596-21|BIOUG65682-G05|BOLD:AAB4894
Sitobion avenae|GMPJA2630-21|BIOUG65683-B04|BOLD:AAB4894
Sitobion|GMPJA2593-21|BIOUG65682-G02|BOLD:AAB4894
Sitobion avenae|GMPJA2546-21|BIOUG65682-C03|BOLD:AAB4894
Sitobion avenae|GMPJA2523-21|BIOUG65682-A04|BOLD:AAB4894
Sitobion avenae|GMPJA2611-21|BIOUG65682-H08|BOLD:AAB4894
Sitobion avenae|GMPJA2636-21|BIOUG65683-B10|BOLD:AAB4894
Sitobion avenae|GMPJA2604-21|BIOUG65682-H01|BOLD:AAB4894
Sitobion avenae|GMPJA2520-21|BIOUG65682-A01|BOLD:AAB4894
Megaspilidae|GMPJA6011-21|BIOUG66617-C02|BOLD:AEJ2653
Megaspilidae|GMPJA10182-21|BIOUG70231-H07|BOLD:ACS6333
Megaspilidae|GMPJA2835-21|BIOUG65685-C07|BOLD:ABX9985
Megaspilidae|GMPJA903-21|BIOUG65664-H10|BOLD:ABX9985
Megaspilidae|GMPJA2869-21|BIOUG65685-F05|BOLD:ABX9985
Megaspilidae|GMPJA2798-21|BIOUG65684-H05|BOLD:ABX9985
Megaspilidae|GMPJA2796-21|BIOUG65684-H03|BOLD:ABX9985
Megaspilidae|GMPJA2972-21|BIOUG65686-C03|BOLD:ABX9985
Megaspilidae|GMPJA2795-21|BIOUG65684-H02|BOLD:ABX9985
Ceraphronidae|GMPJA9681-21|BIOUG66655-D05|BOLD:AEK2586
Ceraphronidae|GMPJA6962-21|BIOUG66627-C03|BOLD:ADS9711
Ceraphronidae|GMPJA9599-21|BIOUG66654-E06|BOLD:ACP5315
Ceraphronidae|GMPJA9294-21|BIOUG66651-C10|BOLD:ACP5315
Ceraphronidae|GMPJA9262-21|BIOUG66651-A02|BOLD:ACP5315
Ceraphronidae|GMPJA9437-21|BIOUG66652-G10|BOLD:AEJ2997
Ceraphronidae|GMPJA9643-21|BIOUG66655-A03|BOLD:AEJ0252
Ceraphronidae|GMPJA9769-21|BIOUG66656-C10|BOLD:ACW6765
Ceraphronidae|GMPJA9408-21|BIOUG66652-E05|BOLD:ACW6765
Ceraphronidae|GMPJA9377-21|BIOUG66652-B10|BOLD:ACW6765
Ceraphronidae|GMPJA9445-21|BIOUG66652-H06|BOLD:ACW6765
Ceraphronidae|GMPJA9573-21|BIOUG66654-C04|BOLD:AEJ6663
Ceraphronidae|GMPJA9422-21|BIOUG66652-F07|BOLD:AEJ9032
Ceraphronidae|GMPJA9386-21|BIOUG66652-C07|BOLD:AEJ9032
Ceraphronidae|GMPJA5966-21|BIOUG66616-G04|BOLD:AEJ6020
Ceraphronidae|GMPJA3720-21|BIOUG65694-B03|BOLD:ACX7020
Ceraphronidae|GMPJA799-21|BIOUG65663-H01|BOLD:ADG0050
Ceraphronidae|GMPJA9506-21|BIOUG66653-E08|BOLD:ABX8445
Ceraphronidae|GMPJA6085-21|BIOUG66618-A05|BOLD:AEJ6658
Ceraphronidae|GMPJA2824-21|BIOUG65685-B08|BOLD:AEJ5301
Ceraphronidae|GMPJA587-21|BIOUG65661-F03|BOLD:AEK0051
Ceraphronidae|GMPJA2794-21|BIOUG65684-H01|BOLD:ACP2745
Mutillidae|GMPJA10142-21|BIOUG66563-E04|BOLD:AEK4940
Mutillidae|GMPJA3708-21|BIOUG65694-A03|BOLD:AEJ0132
Mutillidae|GMPJA6144-21|BIOUG66618-F04|BOLD:AEJ0132
Evaniidae|GMPJA4920-21|BIOUG63984-G03|BOLD:AAQ0494
Xanthopimpla|GMPJA8845-21|BIOUG63985-C04|BOLD:ACI8048
Ichneumonidae|GMPJA4916-21|BIOUG63984-F11|BOLD:ABV2687
Carinostigmus congruus|GMPJA894-21|BIOUG65664-H01|BOLD:ACD9087
Cerceris|GMPJA10144-21|BIOUG66563-E06|BOLD:ACP9123
Cerceris|GMPJA8846-21|BIOUG63985-C05|BOLD:ACP9123
Philanthidae|GMPJA10153-21|BIOUG66563-F03|BOLD:AEJ5559
Philanthidae|GMPJA7008-21|BIOUG66627-G01|BOLD:AEJ5559
Philanthinae|GMPJA3717-21|BIOUG65694-A12|BOLD:AAQ3038
Philanthinae|GMPJA5998-21|BIOUG66617-B01|BOLD:AAQ3038
Cerceris|GMPJA10155-21|BIOUG66563-F05|BOLD:ACD9113
Cerceris|GMPJA10145-21|BIOUG66563-E07|BOLD:ACD9113
Cerceris|GMPJA6216-21|BIOUG66619-D05|BOLD:ACD9113
Cerceris|GMPJA6148-21|BIOUG66618-F08|BOLD:ACD9113
Cerceris|GMPJA6022-21|BIOUG66617-D01|BOLD:ACD9113
Cerceris|GMPJA5991-21|BIOUG66617-A06|BOLD:ACD9113
Bethylidae|GMPJA6154-21|BIOUG66618-G02|BOLD:ABX9693
Bethylidae|GMPJA6069-21|BIOUG66617-G12|BOLD:AEK0025
Bethylidae|GMPJA3726-21|BIOUG65694-B09|BOLD:AEJ8807
Bethylidae|GMPJA9902-21|BIOUG66657-F12|BOLD:ACY5969
Bethylidae|GMPJA4746-21|BIOUG66604-H07|BOLD:ABX9356
Bethylidae|GMPJA9745-21|BIOUG66656-A10|BOLD:ABX9356
Bethylidae|GMPJA9470-21|BIOUG66653-B08|BOLD:AEJ7435
Bethylidae|GMPJA5784-21|BIOUG66614-G12|BOLD:AEJ4015
Bethylidae|GMPJA6116-21|BIOUG66618-C12|BOLD:ACA6592
Bethylidae|GMPJA10708-21|BIOUG70237-D11|BOLD:ACD9013
Bethylidae|GMPJA4704-21|BIOUG66604-E01|BOLD:ACD9013
Bethylidae|GMPJA3770-21|BIOUG65694-F05|BOLD:ACD9013
Bethylidae|GMPJA6171-21|BIOUG66618-H07|BOLD:ACD9013
Bethylidae|GMPJA6169-21|BIOUG66618-H05|BOLD:ACD9013
Bethylidae|GMPJA6157-21|BIOUG66618-G05|BOLD:ACD9013
Bethylidae|GMPJA10686-21|BIOUG70237-C01|BOLD:ADZ9383
Bethylidae|GMPJA6168-21|BIOUG66618-H04|BOLD:ADZ9383
Bethylidae|GMPJA5750-21|BIOUG66614-E02|BOLD:ADZ9383
Tiphiidae|GMPJA6234-21|BIOUG66619-E11|BOLD:ACD9247
Tiphiidae|GMPJA4924-21|BIOUG63984-G07|BOLD:ADU2415
Ammatomus|GMPJA3730-21|BIOUG65694-C01|BOLD:ACD8964
Ammatomus|GMPJA3718-21|BIOUG65694-B01|BOLD:ACD8964
Bembicidae|GMPJA6971-21|BIOUG66627-C12|BOLD:AEJ9345
Harpactus pulchellus|GMPJA2799-21|BIOUG65684-H06|BOLD:ACG1746
Bembecinus|GMPJA6176-21|BIOUG66619-A01|BOLD:ABX8115
Bembecinus|GMPJA6175-21|BIOUG66618-H11|BOLD:AAN8188
Bembecinus|GMPJA6003-21|BIOUG66617-B06|BOLD:AAN8188
Bembecinus|GMPJA4922-21|BIOUG63984-G05|BOLD:AAN8188
Sclerogibbidae|GMPJA6021-21|BIOUG66617-C12|BOLD:AEJ9381
Halictidae|GMPJA6146-21|BIOUG66618-F06|BOLD:AEJ0078
Halictidae|GMPJA6143-21|BIOUG66618-F03|BOLD:AEJ0078
Nomioides|GMPJA6167-21|BIOUG66618-H03|BOLD:ACG1743
Nomioides|GMPJA6156-21|BIOUG66618-G04|BOLD:ACG1743
Nomioides|GMPJA9449-21|BIOUG66652-H10|BOLD:ACG1743
Nomioides|GMPJA6150-21|BIOUG66618-F10|BOLD:ACG1743
Nomioides|GMPJA5985-21|BIOUG66616-H11|BOLD:ACG1743
Nomioides|GMPJA5790-21|BIOUG66614-H06|BOLD:ACG1743
Lipotriches minutula|GMPJA6045-21|BIOUG66617-E12|BOLD:ACW8652
Lasioglossum|GMPJA6081-21|BIOUG66618-A01|BOLD:AAN8625
Halictus|GMPJA6086-21|BIOUG66618-A06|BOLD:ACA2407
Halictinae|GMPJA6149-21|BIOUG66618-F09|BOLD:AAG8315
Halictinae|GMPJA10154-21|BIOUG66563-F04|BOLD:AAG8315
Halictinae|GMPJA10149-21|BIOUG66563-E11|BOLD:AAG8315

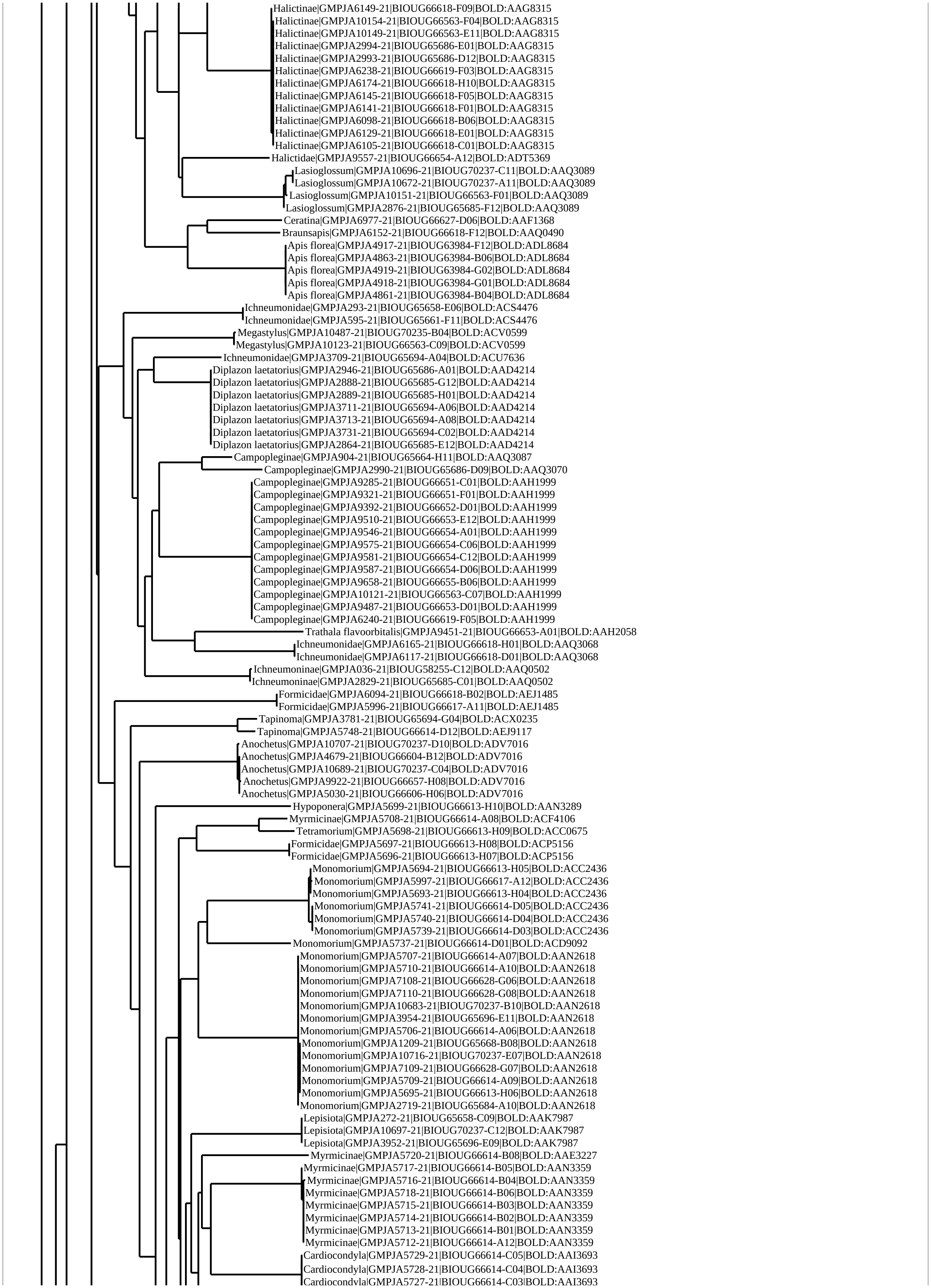
Halictinae|GMPJA6149-21|BIOUG66618-F09|BOLD:AAG8315
Halictinae|GMPJA10154-21|BIOUG66563-F04|BOLD:AAG8315
Halictinae|GMPJA10149-21|BIOUG66563-E11|BOLD:AAG8315
Halictinae|GMPJA2994-21|BIOUG65686-E01|BOLD:AAG8315
Halictinae|GMPJA2993-21|BIOUG65686-D12|BOLD:AAG8315
Halictinae|GMPJA6238-21|BIOUG66619-F03|BOLD:AAG8315
Halictinae|GMPJA6174-21|BIOUG66618-H10|BOLD:AAG8315
Halictinae|GMPJA6145-21|BIOUG66618-F05|BOLD:AAG8315
Halictinae|GMPJA6141-21|BIOUG66618-F01|BOLD:AAG8315
Halictinae|GMPJA6098-21|BIOUG66618-B06|BOLD:AAG8315
Halictinae|GMPJA6129-21|BIOUG66618-E01|BOLD:AAG8315
Halictinae|GMPJA6105-21|BIOUG66618-C01|BOLD:AAG8315
Halictidae|GMPJA9557-21|BIOUG66654-A12|BOLD:ADT5369
Lasioglossum|GMPJA10696-21|BIOUG70237-C11|BOLD:AAQ3089
Lasioglossum|GMPJA10672-21|BIOUG70237-A11|BOLD:AAQ3089
Lasioglossum|GMPJA10151-21|BIOUG66563-F01|BOLD:AAQ3089
Lasioglossum|GMPJA2876-21|BIOUG65685-F12|BOLD:AAQ3089
Ceratina|GMPJA6977-21|BIOUG66627-D06|BOLD:AAF1368
Braunsapis|GMPJA6152-21|BIOUG66618-F12|BOLD:AAQ0490
Apis florea|GMPJA4917-21|BIOUG63984-F12|BOLD:ADL8684
Apis florea|GMPJA4863-21|BIOUG63984-B06|BOLD:ADL8684
Apis florea|GMPJA4919-21|BIOUG63984-G02|BOLD:ADL8684
Apis florea|GMPJA4918-21|BIOUG63984-G01|BOLD:ADL8684
Apis florea|GMPJA4861-21|BIOUG63984-B04|BOLD:ADL8684
Ichneumonidae|GMPJA293-21|BIOUG65658-E06|BOLD:ACS4476
Ichneumonidae|GMPJA595-21|BIOUG65661-F11|BOLD:ACS4476
Megastylus|GMPJA10487-21|BIOUG70235-B04|BOLD:ACV0599
Megastylus|GMPJA10123-21|BIOUG66563-C09|BOLD:ACV0599
Ichneumonidae|GMPJA3709-21|BIOUG65694-A04|BOLD:ACU7636
Diplazon laetatorius|GMPJA2946-21|BIOUG65686-A01|BOLD:AAD4214
Diplazon laetatorius|GMPJA2888-21|BIOUG65685-G12|BOLD:AAD4214
Diplazon laetatorius|GMPJA2889-21|BIOUG65685-H01|BOLD:AAD4214
Diplazon laetatorius|GMPJA3711-21|BIOUG65694-A06|BOLD:AAD4214
Diplazon laetatorius|GMPJA3713-21|BIOUG65694-A08|BOLD:AAD4214
Diplazon laetatorius|GMPJA3731-21|BIOUG65694-C02|BOLD:AAD4214
Diplazon laetatorius|GMPJA2864-21|BIOUG65685-E12|BOLD:AAD4214
Campopleginae|GMPJA904-21|BIOUG65664-H11|BOLD:AAQ3087
Campopleginae|GMPJA2990-21|BIOUG65686-D09|BOLD:AAQ3070
Campopleginae|GMPJA9285-21|BIOUG66651-C01|BOLD:AAH1999
Campopleginae|GMPJA9321-21|BIOUG66651-F01|BOLD:AAH1999
Campopleginae|GMPJA9392-21|BIOUG66652-D01|BOLD:AAH1999
Campopleginae|GMPJA9510-21|BIOUG66653-E12|BOLD:AAH1999
Campopleginae|GMPJA9546-21|BIOUG66654-A01|BOLD:AAH1999
Campopleginae|GMPJA9575-21|BIOUG66654-C06|BOLD:AAH1999
Campopleginae|GMPJA9581-21|BIOUG66654-C12|BOLD:AAH1999
Campopleginae|GMPJA9587-21|BIOUG66654-D06|BOLD:AAH1999
Campopleginae|GMPJA9658-21|BIOUG66655-B06|BOLD:AAH1999
Campopleginae|GMPJA10121-21|BIOUG66563-C07|BOLD:AAH1999
Campopleginae|GMPJA9487-21|BIOUG66653-D01|BOLD:AAH1999
Campopleginae|GMPJA6240-21|BIOUG66619-F05|BOLD:AAH1999
Trathala flavoorbitalis|GMPJA9451-21|BIOUG66653-A01|BOLD:AAH2058
Ichneumonidae|GMPJA6165-21|BIOUG66618-H01|BOLD:AAQ3068
Ichneumonidae|GMPJA6117-21|BIOUG66618-D01|BOLD:AAQ3068
Ichneumoninae|GMPJA036-21|BIOUG58255-C12|BOLD:AAQ0502
Ichneumoninae|GMPJA2829-21|BIOUG65685-C01|BOLD:AAQ0502
Formicidae|GMPJA6094-21|BIOUG66618-B02|BOLD:AEJ1485
Formicidae|GMPJA5996-21|BIOUG66617-A11|BOLD:AEJ1485
Tapinoma|GMPJA3781-21|BIOUG65694-G04|BOLD:ACX0235
Tapinoma|GMPJA5748-21|BIOUG66614-D12|BOLD:AEJ9117
Anochetus|GMPJA10707-21|BIOUG70237-D10|BOLD:ADV7016
Anochetus|GMPJA4679-21|BIOUG66604-B12|BOLD:ADV7016
Anochetus|GMPJA10689-21|BIOUG70237-C04|BOLD:ADV7016
Anochetus|GMPJA9922-21|BIOUG66657-H08|BOLD:ADV7016
Anochetus|GMPJA5030-21|BIOUG66606-H06|BOLD:ADV7016
Hypoponera|GMPJA5699-21|BIOUG66613-H10|BOLD:AAN3289
Myrmicinae|GMPJA5708-21|BIOUG66614-A08|BOLD:ACF4106
Tetramorium|GMPJA5698-21|BIOUG66613-H09|BOLD:ACC0675
Formicidae|GMPJA5697-21|BIOUG66613-H08|BOLD:ACP5156
Formicidae|GMPJA5696-21|BIOUG66613-H07|BOLD:ACP5156
Monomorium|GMPJA5694-21|BIOUG66613-H05|BOLD:ACC2436
Monomorium|GMPJA5997-21|BIOUG66617-A12|BOLD:ACC2436
Monomorium|GMPJA5693-21|BIOUG66613-H04|BOLD:ACC2436
Monomorium|GMPJA5741-21|BIOUG66614-D05|BOLD:ACC2436
Monomorium|GMPJA5740-21|BIOUG66614-D04|BOLD:ACC2436
Monomorium|GMPJA5739-21|BIOUG66614-D03|BOLD:ACC2436
Monomorium|GMPJA5737-21|BIOUG66614-D01|BOLD:ACD9092
Monomorium|GMPJA5707-21|BIOUG66614-A07|BOLD:AAN2618
Monomorium|GMPJA5710-21|BIOUG66614-A10|BOLD:AAN2618
Monomorium|GMPJA7108-21|BIOUG66628-G06|BOLD:AAN2618
Monomorium|GMPJA7110-21|BIOUG66628-G08|BOLD:AAN2618
Monomorium|GMPJA10683-21|BIOUG70237-B10|BOLD:AAN2618
Monomorium|GMPJA3954-21|BIOUG65696-E11|BOLD:AAN2618
Monomorium|GMPJA5706-21|BIOUG66614-A06|BOLD:AAN2618
Monomorium|GMPJA1209-21|BIOUG65668-B08|BOLD:AAN2618
Monomorium|GMPJA10716-21|BIOUG70237-E07|BOLD:AAN2618
Monomorium|GMPJA7109-21|BIOUG66628-G07|BOLD:AAN2618
Monomorium|GMPJA5709-21|BIOUG66614-A09|BOLD:AAN2618
Monomorium|GMPJA5695-21|BIOUG66613-H06|BOLD:AAN2618
Monomorium|GMPJA2719-21|BIOUG65684-A10|BOLD:AAN2618
Lepisiota|GMPJA272-21|BIOUG65658-C09|BOLD:AAK7987
Lepisiota|GMPJA10697-21|BIOUG70237-C12|BOLD:AAK7987
Lepisiota|GMPJA3952-21|BIOUG65696-E09|BOLD:AAK7987
Myrmicinae|GMPJA5720-21|BIOUG66614-B08|BOLD:AAE3227
Myrmicinae|GMPJA5717-21|BIOUG66614-B05|BOLD:AAN3359
Myrmicinae|GMPJA5716-21|BIOUG66614-B04|BOLD:AAN3359
Myrmicinae|GMPJA5718-21|BIOUG66614-B06|BOLD:AAN3359
Myrmicinae|GMPJA5715-21|BIOUG66614-B03|BOLD:AAN3359
Myrmicinae|GMPJA5714-21|BIOUG66614-B02|BOLD:AAN3359
Myrmicinae|GMPJA5713-21|BIOUG66614-B01|BOLD:AAN3359
Myrmicinae|GMPJA5712-21|BIOUG66614-A12|BOLD:AAN3359
Cardiocondyla|GMPJA5729-21|BIOUG66614-C05|BOLD:AAI3693
Cardiocondyla|GMPJA5728-21|BIOUG66614-C04|BOLD:AAI3693
Cardiocondyla|GMPJA5727-21|BIOUG66614-C03|BOLD:AAI3693

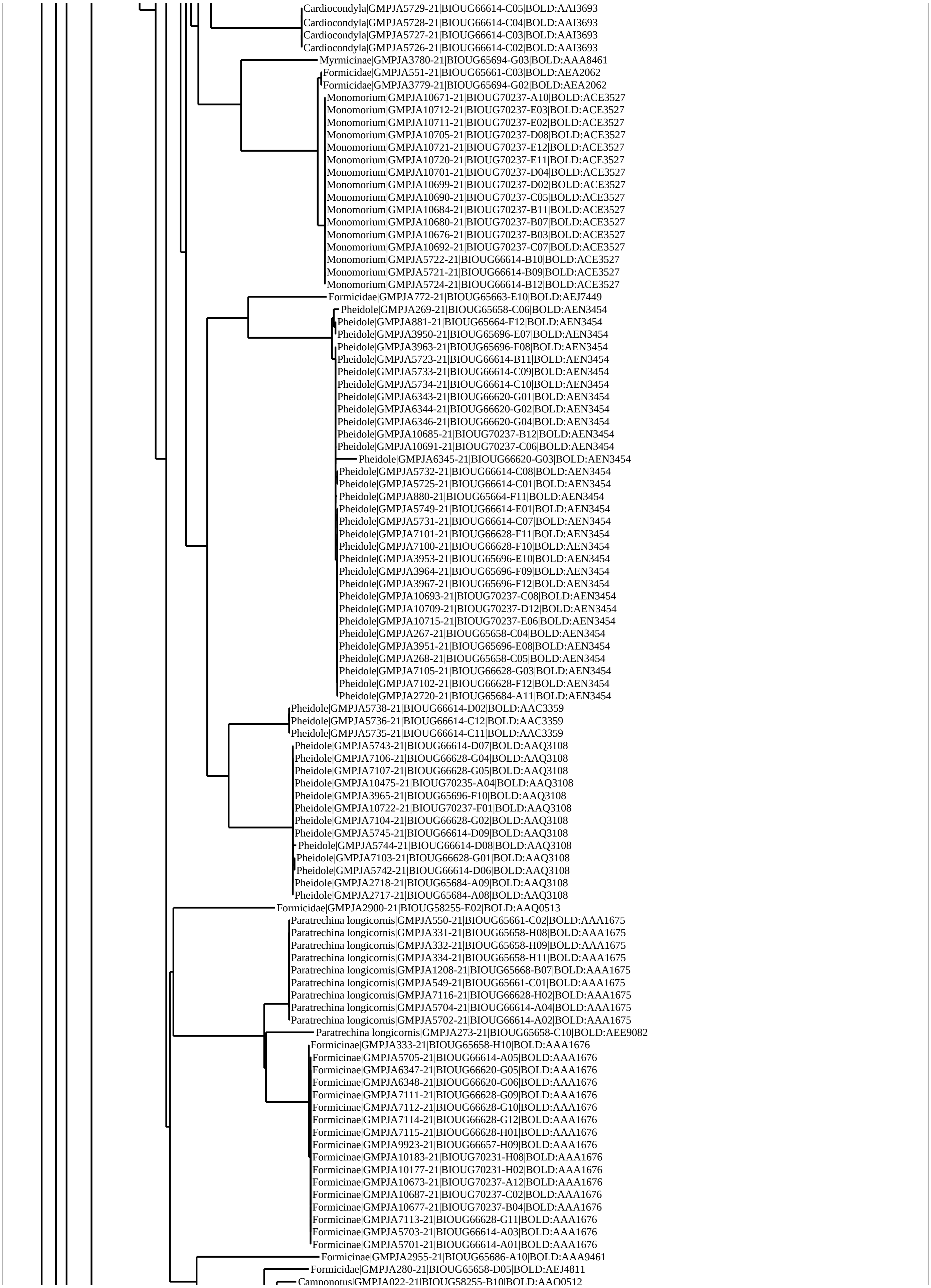
Cardiocondyla|GMPJA5729-21|BIOUG66614-C05|BOLD:AAI3693
Cardiocondyla|GMPJA5728-21|BIOUG66614-C04|BOLD:AAI3693
Cardiocondyla|GMPJA5727-21|BIOUG66614-C03|BOLD:AAI3693
Cardiocondyla|GMPJA5726-21|BIOUG66614-C02|BOLD:AAI3693
Myrmicinae|GMPJA3780-21|BIOUG65694-G03|BOLD:AAA8461
Formicidae|GMPJA551-21|BIOUG65661-C03|BOLD:AEA2062
Formicidae|GMPJA3779-21|BIOUG65694-G02|BOLD:AEA2062
Monomorium|GMPJA10671-21|BIOUG70237-A10|BOLD:ACE3527
Monomorium|GMPJA10712-21|BIOUG70237-E03|BOLD:ACE3527
Monomorium|GMPJA10711-21|BIOUG70237-E02|BOLD:ACE3527
Monomorium|GMPJA10705-21|BIOUG70237-D08|BOLD:ACE3527
Monomorium|GMPJA10721-21|BIOUG70237-E12|BOLD:ACE3527
Monomorium|GMPJA10720-21|BIOUG70237-E11|BOLD:ACE3527
Monomorium|GMPJA10701-21|BIOUG70237-D04|BOLD:ACE3527
Monomorium|GMPJA10699-21|BIOUG70237-D02|BOLD:ACE3527
Monomorium|GMPJA10690-21|BIOUG70237-C05|BOLD:ACE3527
Monomorium|GMPJA10684-21|BIOUG70237-B11|BOLD:ACE3527
Monomorium|GMPJA10680-21|BIOUG70237-B07|BOLD:ACE3527
Monomorium|GMPJA10676-21|BIOUG70237-B03|BOLD:ACE3527
Monomorium|GMPJA10692-21|BIOUG70237-C07|BOLD:ACE3527
Monomorium|GMPJA5722-21|BIOUG66614-B10|BOLD:ACE3527
Monomorium|GMPJA5721-21|BIOUG66614-B09|BOLD:ACE3527
Monomorium|GMPJA5724-21|BIOUG66614-B12|BOLD:ACE3527
Formicidae|GMPJA772-21|BIOUG65663-E10|BOLD:AEJ7449
Pheidole|GMPJA269-21|BIOUG65658-C06|BOLD:AEN3454
Pheidole|GMPJA881-21|BIOUG65664-F12|BOLD:AEN3454
Pheidole|GMPJA3950-21|BIOUG65696-E07|BOLD:AEN3454
Pheidole|GMPJA3963-21|BIOUG65696-F08|BOLD:AEN3454
Pheidole|GMPJA5723-21|BIOUG66614-B11|BOLD:AEN3454
Pheidole|GMPJA5733-21|BIOUG66614-C09|BOLD:AEN3454
Pheidole|GMPJA5734-21|BIOUG66614-C10|BOLD:AEN3454
Pheidole|GMPJA6343-21|BIOUG66620-G01|BOLD:AEN3454
Pheidole|GMPJA6344-21|BIOUG66620-G02|BOLD:AEN3454
Pheidole|GMPJA6346-21|BIOUG66620-G04|BOLD:AEN3454
Pheidole|GMPJA10685-21|BIOUG70237-B12|BOLD:AEN3454
Pheidole|GMPJA10691-21|BIOUG70237-C06|BOLD:AEN3454
Pheidole|GMPJA6345-21|BIOUG66620-G03|BOLD:AEN3454
Pheidole|GMPJA5732-21|BIOUG66614-C08|BOLD:AEN3454
Pheidole|GMPJA5725-21|BIOUG66614-C01|BOLD:AEN3454
Pheidole|GMPJA880-21|BIOUG65664-F11|BOLD:AEN3454
Pheidole|GMPJA5749-21|BIOUG66614-E01|BOLD:AEN3454
Pheidole|GMPJA5731-21|BIOUG66614-C07|BOLD:AEN3454
Pheidole|GMPJA7101-21|BIOUG66628-F11|BOLD:AEN3454
Pheidole|GMPJA7100-21|BIOUG66628-F10|BOLD:AEN3454
Pheidole|GMPJA3953-21|BIOUG65696-E10|BOLD:AEN3454
Pheidole|GMPJA3964-21|BIOUG65696-F09|BOLD:AEN3454
Pheidole|GMPJA3967-21|BIOUG65696-F12|BOLD:AEN3454
Pheidole|GMPJA10693-21|BIOUG70237-C08|BOLD:AEN3454
Pheidole|GMPJA10709-21|BIOUG70237-D12|BOLD:AEN3454
Pheidole|GMPJA10715-21|BIOUG70237-E06|BOLD:AEN3454
Pheidole|GMPJA267-21|BIOUG65658-C04|BOLD:AEN3454
Pheidole|GMPJA3951-21|BIOUG65696-E08|BOLD:AEN3454
Pheidole|GMPJA268-21|BIOUG65658-C05|BOLD:AEN3454
Pheidole|GMPJA7105-21|BIOUG66628-G03|BOLD:AEN3454
Pheidole|GMPJA7102-21|BIOUG66628-F12|BOLD:AEN3454
Pheidole|GMPJA2720-21|BIOUG65684-A11|BOLD:AEN3454
Pheidole|GMPJA5738-21|BIOUG66614-D02|BOLD:AAC3359
Pheidole|GMPJA5736-21|BIOUG66614-C12|BOLD:AAC3359
Pheidole|GMPJA5735-21|BIOUG66614-C11|BOLD:AAC3359
Pheidole|GMPJA5743-21|BIOUG66614-D07|BOLD:AAQ3108
Pheidole|GMPJA7106-21|BIOUG66628-G04|BOLD:AAQ3108
Pheidole|GMPJA7107-21|BIOUG66628-G05|BOLD:AAQ3108
Pheidole|GMPJA10475-21|BIOUG70235-A04|BOLD:AAQ3108
Pheidole|GMPJA3965-21|BIOUG65696-F10|BOLD:AAQ3108
Pheidole|GMPJA10722-21|BIOUG70237-F01|BOLD:AAQ3108
Pheidole|GMPJA7104-21|BIOUG66628-G02|BOLD:AAQ3108
Pheidole|GMPJA5745-21|BIOUG66614-D09|BOLD:AAQ3108
Pheidole|GMPJA5744-21|BIOUG66614-D08|BOLD:AAQ3108
Pheidole|GMPJA7103-21|BIOUG66628-G01|BOLD:AAQ3108
Pheidole|GMPJA5742-21|BIOUG66614-D06|BOLD:AAQ3108
Pheidole|GMPJA2718-21|BIOUG65684-A09|BOLD:AAQ3108
Pheidole|GMPJA2717-21|BIOUG65684-A08|BOLD:AAQ3108
Formicidae|GMPJA2900-21|BIOUG58255-E02|BOLD:AAQ0513
Paratrechina longicornis|GMPJA550-21|BIOUG65661-C02|BOLD:AAA1675
Paratrechina longicornis|GMPJA331-21|BIOUG65658-H08|BOLD:AAA1675
Paratrechina longicornis|GMPJA332-21|BIOUG65658-H09|BOLD:AAA1675
Paratrechina longicornis|GMPJA334-21|BIOUG65658-H11|BOLD:AAA1675
Paratrechina longicornis|GMPJA1208-21|BIOUG65668-B07|BOLD:AAA1675
Paratrechina longicornis|GMPJA549-21|BIOUG65661-C01|BOLD:AAA1675
Paratrechina longicornis|GMPJA7116-21|BIOUG66628-H02|BOLD:AAA1675
Paratrechina longicornis|GMPJA5704-21|BIOUG66614-A04|BOLD:AAA1675
Paratrechina longicornis|GMPJA5702-21|BIOUG66614-A02|BOLD:AAA1675
Paratrechina longicornis|GMPJA273-21|BIOUG65658-C10|BOLD:AEE9082
Formicinae|GMPJA333-21|BIOUG65658-H10|BOLD:AAA1676
Formicinae|GMPJA5705-21|BIOUG66614-A05|BOLD:AAA1676
Formicinae|GMPJA6347-21|BIOUG66620-G05|BOLD:AAA1676
Formicinae|GMPJA6348-21|BIOUG66620-G06|BOLD:AAA1676
Formicinae|GMPJA7111-21|BIOUG66628-G09|BOLD:AAA1676
Formicinae|GMPJA7112-21|BIOUG66628-G10|BOLD:AAA1676
Formicinae|GMPJA7114-21|BIOUG66628-G12|BOLD:AAA1676
Formicinae|GMPJA7115-21|BIOUG66628-H01|BOLD:AAA1676
Formicinae|GMPJA9923-21|BIOUG66657-H09|BOLD:AAA1676
Formicinae|GMPJA10183-21|BIOUG70231-H08|BOLD:AAA1676
Formicinae|GMPJA10177-21|BIOUG70231-H02|BOLD:AAA1676
Formicinae|GMPJA10673-21|BIOUG70237-A12|BOLD:AAA1676
Formicinae|GMPJA10687-21|BIOUG70237-C02|BOLD:AAA1676
Formicinae|GMPJA10677-21|BIOUG70237-B04|BOLD:AAA1676
Formicinae|GMPJA7113-21|BIOUG66628-G11|BOLD:AAA1676
Formicinae|GMPJA5703-21|BIOUG66614-A03|BOLD:AAA1676
Formicinae|GMPJA5701-21|BIOUG66614-A01|BOLD:AAA1676
Formicinae|GMPJA2955-21|BIOUG65686-A10|BOLD:AAA9461
Formicidae|GMPJA280-21|BIOUG65658-D05|BOLD:AEJ4811
Camponotus|GMPJA022-21|BIOUG58255-B10|BOLD:AAO0512

Formicinae|GMPJA2955-21|BIOUG65686-A10|BOLD:AAA9461
Formicidae|GMPJA280-21|BIOUG65658-D05|BOLD:AEJ4811
Camponotus|GMPJA022-21|BIOUG58255-B10|BOLD:AAQ0512
Camponotus|GMPJA10129-21|BIOUG66563-D03|BOLD:AAN2976
Camponotus|GMPJA5687-21|BIOUG66613-G10|BOLD:AAN2976
Camponotus|GMPJA5685-21|BIOUG66613-G08|BOLD:AAN2976
Camponotus|GMPJA10160-21|BIOUG66563-F10|BOLD:AAN2976
Camponotus|GMPJA10157-21|BIOUG66563-F07|BOLD:AAN2976
Camponotus|GMPJA558-21|BIOUG65661-C10|BOLD:AAN2976
Camponotus|GMPJA559-21|BIOUG65661-C11|BOLD:AAN2976
Camponotus|GMPJA563-21|BIOUG65661-D03|BOLD:AAN2976
Camponotus|GMPJA009-21|BIOUG58255-A09|BOLD:AAN2976
Camponotus|GMPJA011-21|BIOUG58255-A11|BOLD:AAN2976
Camponotus|GMPJA026-21|BIOUG58255-C02|BOLD:AAN2976
Camponotus|GMPJA281-21|BIOUG65658-D06|BOLD:AAN2976
Camponotus|GMPJA776-21|BIOUG65663-F02|BOLD:AAN2976
Camponotus|GMPJA561-21|BIOUG65661-D01|BOLD:AAN2976
Camponotus|GMPJA10126-21|BIOUG66563-C12|BOLD:AAN2976
Camponotus|GMPJA7122-21|BIOUG66628-H08|BOLD:AAN2976
Camponotus|GMPJA2723-21|BIOUG65684-B02|BOLD:AAN2976
Camponotus|GMPJA560-21|BIOUG65661-C12|BOLD:AAN2976
Camponotus|GMPJA553-21|BIOUG65661-C05|BOLD:AAN2976
Camponotus|GMPJA554-21|BIOUG65661-C06|BOLD:AAN2976
Camponotus|GMPJA275-21|BIOUG65658-C12|BOLD:AAN2976
Camponotus|GMPJA282-21|BIOUG65658-D07|BOLD:AAN2976
Camponotus|GMPJA10159-21|BIOUG66563-F09|BOLD:AAN2976
Camponotus|GMPJA10125-21|BIOUG66563-C11|BOLD:AAN2976
Camponotus|GMPJA5689-21|BIOUG66613-G12|BOLD:AAN2976
Camponotus|GMPJA5688-21|BIOUG66613-G11|BOLD:AAN2976
Camponotus|GMPJA5686-21|BIOUG66613-G09|BOLD:AAN2976
Camponotus|GMPJA2721-21|BIOUG65684-A12|BOLD:AAN2976
Camponotus|GMPJA4862-21|BIOUG63984-B05|BOLD:AAN2976
Camponotus|GMPJA4927-21|BIOUG63984-G10|BOLD:AAN2976
Camponotus|GMPJA7124-21|BIOUG66628-H10|BOLD:AAN2976
Camponotus|GMPJA4925-21|BIOUG63984-G08|BOLD:AAN2976
Camponotus|GMPJA4926-21|BIOUG63984-G09|BOLD:AAN2976
Camponotus|GMPJA4933-21|BIOUG63984-H04|BOLD:AAN2976
Camponotus|GMPJA6342-21|BIOUG66620-F12|BOLD:AAN2976
Camponotus|GMPJA7117-21|BIOUG66628-H03|BOLD:AAN2976
Camponotus|GMPJA7118-21|BIOUG66628-H04|BOLD:AAN2976
Camponotus|GMPJA7120-21|BIOUG66628-H06|BOLD:AAN2976
Camponotus|GMPJA7121-21|BIOUG66628-H07|BOLD:AAN2976
Camponotus|GMPJA7123-21|BIOUG66628-H09|BOLD:AAN2976
Camponotus|GMPJA7125-21|BIOUG66628-H11|BOLD:AAN2976
Camponotus|GMPJA8837-21|BIOUG63985-B08|BOLD:AAN2976
Camponotus|GMPJA8838-21|BIOUG63985-B09|BOLD:AAN2976
Camponotus|GMPJA2901-21|BIOUG58255-E03|BOLD:AAN2976
Camponotus|GMPJA10158-21|BIOUG66563-F08|BOLD:AAN2976
Camponotus|GMPJA10124-21|BIOUG66563-C10|BOLD:AAN2976
Camponotus|GMPJA10127-21|BIOUG66563-D01|BOLD:AAN2976
Camponotus|GMPJA10161-21|BIOUG66563-F11|BOLD:AAN2976
Camponotus|GMPJA3962-21|BIOUG65696-F07|BOLD:AAN2976
Camponotus|GMPJA555-21|BIOUG65661-C07|BOLD:AAN2976
Camponotus|GMPJA556-21|BIOUG65661-C08|BOLD:AAN2976
Camponotus|GMPJA557-21|BIOUG65661-C09|BOLD:AAN2976
Camponotus|GMPJA562-21|BIOUG65661-D02|BOLD:AAN2976
Camponotus|GMPJA564-21|BIOUG65661-D04|BOLD:AAN2976
Camponotus|GMPJA008-21|BIOUG58255-A08|BOLD:AAN2976
Camponotus|GMPJA010-21|BIOUG58255-A10|BOLD:AAN2976
Camponotus|GMPJA012-21|BIOUG58255-A12|BOLD:AAN2976
Camponotus|GMPJA015-21|BIOUG58255-B03|BOLD:AAN2976
Camponotus|GMPJA016-21|BIOUG58255-B04|BOLD:AAN2976
Camponotus|GMPJA019-21|BIOUG58255-B07|BOLD:AAN2976
Camponotus|GMPJA020-21|BIOUG58255-B08|BOLD:AAN2976
Camponotus|GMPJA021-21|BIOUG58255-B09|BOLD:AAN2976
Camponotus|GMPJA024-21|BIOUG58255-B12|BOLD:AAN2976
Camponotus|GMPJA025-21|BIOUG58255-C01|BOLD:AAN2976
Camponotus|GMPJA037-21|BIOUG58255-D01|BOLD:AAN2976
Camponotus|GMPJA038-21|BIOUG58255-D02|BOLD:AAN2976
Camponotus|GMPJA274-21|BIOUG65658-C11|BOLD:AAN2976
Camponotus|GMPJA276-21|BIOUG65658-D01|BOLD:AAN2976
Camponotus|GMPJA277-21|BIOUG65658-D02|BOLD:AAN2976
Camponotus|GMPJA279-21|BIOUG65658-D04|BOLD:AAN2976
Camponotus|GMPJA283-21|BIOUG65658-D08|BOLD:AAN2976
Camponotus|GMPJA288-21|BIOUG65658-E01|BOLD:AAN2976
Camponotus|GMPJA774-21|BIOUG65663-E12|BOLD:AAN2976
Camponotus|GMPJA777-21|BIOUG65663-F03|BOLD:AAN2976
Camponotus|GMPJA027-21|BIOUG58255-C03|BOLD:AAN2976
Camponotus|GMPJA10128-21|BIOUG66563-D02|BOLD:AAN2976
Camponotus|GMPJA2722-21|BIOUG65684-B01|BOLD:AAN2976
Camponotus|GMPJA017-21|BIOUG58255-B05|BOLD:AAN2976
Camponotus|GMPJA6181-21|BIOUG66619-A06|BOLD:AAN2976
Camponotus|GMPJA6182-21|BIOUG66619-A07|BOLD:AAN2976
Camponotus|GMPJA2906-21|BIOUG58255-E08|BOLD:AAN2976
Camponotus|GMPJA3961-21|BIOUG65696-F06|BOLD:AAN2976
Camponotus|GMPJA4855-21|BIOUG63984-A10|BOLD:AAN2976
Trypoxylon|GMPJA10094-21|BIOUG63985-A12|BOLD:ACA2605
Bethylinae|GMPJA9689-21|BIOUG66655-E01|BOLD:ACO5182
Chrysididae|GMPJA3753-21|BIOUG65694-D12|BOLD:AEJ0327
Chrysididae|GMPJA2948-21|BIOUG65686-A03|BOLD:AEJ9246
Chrysididae|GMPJA9795-21|BIOUG66656-E12|BOLD:ADU5005
Chrysis|GMPJA4756-21|BIOUG66605-A06|BOLD:ACA6030
Chrysis|GMPJA10119-21|BIOUG66563-C05|BOLD:ACA6030
Chrysis|GMPJA10118-21|BIOUG66563-C04|BOLD:ACA6030
Chrysis|GMPJA9404-21|BIOUG66652-E01|BOLD:ACA6030
Chrysidea|GMPJA6173-21|BIOUG66618-H09|BOLD:AAQ0496
Oxybelus|GMPJA9570-21|BIOUG66654-C01|BOLD:ACP4311
Oxybelus|GMPJA3774-21|BIOUG65694-F09|BOLD:ACP4311
Oxybelus|GMPJA9582-21|BIOUG66654-D01|BOLD:ACP4311
Oxybelus|GMPJA9748-21|BIOUG66656-B01|BOLD:ACP4311
Oxybelus|GMPJA9492-21|BIOUG66653-D06|BOLD:ACP4311
Oxybelus|GMPJA9450-21|BIOUG66652-H11|BOLD:ACP4311
Oxybelus|GMPJA6953-21|BIOUG66627-B06|BOLD:ACP4311

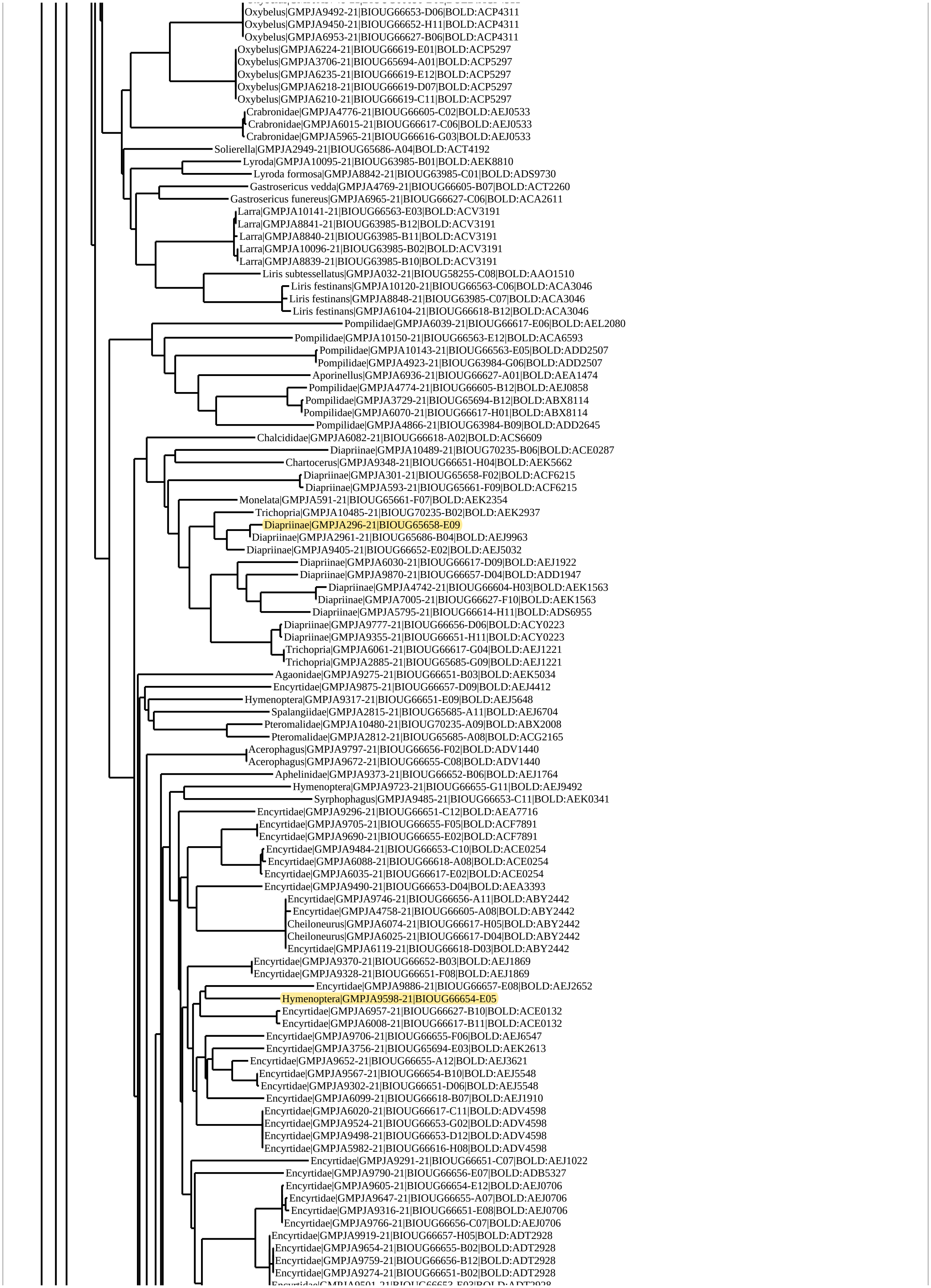
Oxybelus|GMPJA9492-21|BIOUG66653-D06|BOLD:ACP4311
Oxybelus|GMPJA9450-21|BIOUG66652-H11|BOLD:ACP4311
Oxybelus|GMPJA6953-21|BIOUG66627-B06|BOLD:ACP4311
Oxybelus|GMPJA6224-21|BIOUG66619-E01|BOLD:ACP5297
Oxybelus|GMPJA3706-21|BIOUG65694-A01|BOLD:ACP5297
Oxybelus|GMPJA6235-21|BIOUG66619-E12|BOLD:ACP5297
Oxybelus|GMPJA6218-21|BIOUG66619-D07|BOLD:ACP5297
Oxybelus|GMPJA6210-21|BIOUG66619-C11|BOLD:ACP5297
Crabronidae|GMPJA4776-21|BIOUG66605-C02|BOLD:AEJ0533
Crabronidae|GMPJA6015-21|BIOUG66617-C06|BOLD:AEJ0533
Crabronidae|GMPJA5965-21|BIOUG66616-G03|BOLD:AEJ0533
Solierella|GMPJA2949-21|BIOUG65686-A04|BOLD:ACT4192
Lyroda|GMPJA10095-21|BIOUG63985-B01|BOLD:AEK8810
Lyroda formosa|GMPJA8842-21|BIOUG63985-C01|BOLD:ADS9730
Gastrosericus vedda|GMPJA4769-21|BIOUG66605-B07|BOLD:ACT2260
Gastrosericus funereus|GMPJA6965-21|BIOUG66627-C06|BOLD:ACA2611
Larra|GMPJA10141-21|BIOUG66563-E03|BOLD:ACV3191
Larra|GMPJA8841-21|BIOUG63985-B12|BOLD:ACV3191
Larra|GMPJA8840-21|BIOUG63985-B11|BOLD:ACV3191
Larra|GMPJA10096-21|BIOUG63985-B02|BOLD:ACV3191
Larra|GMPJA8839-21|BIOUG63985-B10|BOLD:ACV3191
Liris subtessellatus|GMPJA032-21|BIOUG58255-C08|BOLD:AAO1510
Liris festinans|GMPJA10120-21|BIOUG66563-C06|BOLD:ACA3046
Liris festinans|GMPJA8848-21|BIOUG63985-C07|BOLD:ACA3046
Liris festinans|GMPJA6104-21|BIOUG66618-B12|BOLD:ACA3046
Pompilidae|GMPJA6039-21|BIOUG66617-E06|BOLD:AEL2080
Pompilidae|GMPJA10150-21|BIOUG66563-E12|BOLD:ACA6593
Pompilidae|GMPJA10143-21|BIOUG66563-E05|BOLD:ADD2507
Pompilidae|GMPJA4923-21|BIOUG63984-G06|BOLD:ADD2507
Aporinellus|GMPJA6936-21|BIOUG66627-A01|BOLD:AEA1474
Pompilidae|GMPJA4774-21|BIOUG66605-B12|BOLD:AEJ0858
Pompilidae|GMPJA3729-21|BIOUG65694-B12|BOLD:ABX8114
Pompilidae|GMPJA6070-21|BIOUG66617-H01|BOLD:ABX8114
Pompilidae|GMPJA4866-21|BIOUG63984-B09|BOLD:ADD2645
Chalcididae|GMPJA6082-21|BIOUG66618-A02|BOLD:ACS6609
Diapriinae|GMPJA10489-21|BIOUG70235-B06|BOLD:ACE0287
Chartocerus|GMPJA9348-21|BIOUG66651-H04|BOLD:AEK5662
Diapriinae|GMPJA301-21|BIOUG65658-F02|BOLD:ACF6215
Diapriinae|GMPJA593-21|BIOUG65661-F09|BOLD:ACF6215
Monelata|GMPJA591-21|BIOUG65661-F07|BOLD:AEK2354
Trichopria|GMPJA10485-21|BIOUG70235-B02|BOLD:AEK2937
Diapriinae|GMPJA296-21|BIOUG65658-E09
Diapriinae|GMPJA2961-21|BIOUG65686-B04|BOLD:AEJ9963
Diapriinae|GMPJA9405-21|BIOUG66652-E02|BOLD:AEJ5032
Diapriinae|GMPJA6030-21|BIOUG66617-D09|BOLD:AEJ1922
Diapriinae|GMPJA9870-21|BIOUG66657-D04|BOLD:ADD1947
Diapriinae|GMPJA4742-21|BIOUG66604-H03|BOLD:AEK1563
Diapriinae|GMPJA7005-21|BIOUG66627-F10|BOLD:AEK1563
Diapriinae|GMPJA5795-21|BIOUG66614-H11|BOLD:ADS6955
Diapriinae|GMPJA9777-21|BIOUG66656-D06|BOLD:ACY0223
Diapriinae|GMPJA9355-21|BIOUG66651-H11|BOLD:ACY0223
Trichopria|GMPJA6061-21|BIOUG66617-G04|BOLD:AEJ1221
Trichopria|GMPJA2885-21|BIOUG65685-G09|BOLD:AEJ1221
Agaonidae|GMPJA9275-21|BIOUG66651-B03|BOLD:AEK5034
Encyrtidae|GMPJA9875-21|BIOUG66657-D09|BOLD:AEJ4412
Hymenoptera|GMPJA9317-21|BIOUG66651-E09|BOLD:AEJ5648
Spalangiidae|GMPJA2815-21|BIOUG65685-A11|BOLD:AEJ6704
Pteromalidae|GMPJA10480-21|BIOUG70235-A09|BOLD:ABX2008
Pteromalidae|GMPJA2812-21|BIOUG65685-A08|BOLD:ACG2165
Acerophagus|GMPJA9797-21|BIOUG66656-F02|BOLD:ADV1440
Acerophagus|GMPJA9672-21|BIOUG66655-C08|BOLD:ADV1440
Aphelinidae|GMPJA9373-21|BIOUG66652-B06|BOLD:AEJ1764
Hymenoptera|GMPJA9723-21|BIOUG66655-G11|BOLD:AEJ9492
Syrphophagus|GMPJA9485-21|BIOUG66653-C11|BOLD:AEK0341
Encyrtidae|GMPJA9296-21|BIOUG66651-C12|BOLD:AEA7716
Encyrtidae|GMPJA9705-21|BIOUG66655-F05|BOLD:ACF7891
Encyrtidae|GMPJA9690-21|BIOUG66655-E02|BOLD:ACF7891
Encyrtidae|GMPJA9484-21|BIOUG66653-C10|BOLD:ACE0254
Encyrtidae|GMPJA6088-21|BIOUG66618-A08|BOLD:ACE0254
Encyrtidae|GMPJA6035-21|BIOUG66617-E02|BOLD:ACE0254
Encyrtidae|GMPJA9490-21|BIOUG66653-D04|BOLD:AEA3393
Encyrtidae|GMPJA9746-21|BIOUG66656-A11|BOLD:ABY2442
Encyrtidae|GMPJA4758-21|BIOUG66605-A08|BOLD:ABY2442
Cheiloneurus|GMPJA6074-21|BIOUG66617-H05|BOLD:ABY2442
Cheiloneurus|GMPJA6025-21|BIOUG66617-D04|BOLD:ABY2442
Encyrtidae|GMPJA6119-21|BIOUG66618-D03|BOLD:ABY2442
Encyrtidae|GMPJA9370-21|BIOUG66652-B03|BOLD:AEJ1869
Encyrtidae|GMPJA9328-21|BIOUG66651-F08|BOLD:AEJ1869
Encyrtidae|GMPJA9886-21|BIOUG66657-E08|BOLD:AEJ2652
Hymenoptera|GMPJA9598-21|BIOUG66654-E05
Encyrtidae|GMPJA6957-21|BIOUG66627-B10|BOLD:ACE0132
Encyrtidae|GMPJA6008-21|BIOUG66617-B11|BOLD:ACE0132
Encyrtidae|GMPJA9706-21|BIOUG66655-F06|BOLD:AEJ6547
Encyrtidae|GMPJA3756-21|BIOUG65694-E03|BOLD:AEK2613
Encyrtidae|GMPJA9652-21|BIOUG66655-A12|BOLD:AEJ3621
Encyrtidae|GMPJA9567-21|BIOUG66654-B10|BOLD:AEJ5548
Encyrtidae|GMPJA9302-21|BIOUG66651-D06|BOLD:AEJ5548
Encyrtidae|GMPJA6099-21|BIOUG66618-B07|BOLD:AEJ1910
Encyrtidae|GMPJA6020-21|BIOUG66617-C11|BOLD:ADV4598
Encyrtidae|GMPJA9524-21|BIOUG66653-G02|BOLD:ADV4598
Encyrtidae|GMPJA9498-21|BIOUG66653-D12|BOLD:ADV4598
Encyrtidae|GMPJA5982-21|BIOUG66616-H08|BOLD:ADV4598
Encyrtidae|GMPJA9291-21|BIOUG66651-C07|BOLD:AEJ1022
Encyrtidae|GMPJA9790-21|BIOUG66656-E07|BOLD:ADB5327
Encyrtidae|GMPJA9605-21|BIOUG66654-E12|BOLD:AEJ0706
Encyrtidae|GMPJA9647-21|BIOUG66655-A07|BOLD:AEJ0706
Encyrtidae|GMPJA9316-21|BIOUG66651-E08|BOLD:AEJ0706
Encyrtidae|GMPJA9766-21|BIOUG66656-C07|BOLD:AEJ0706
Encyrtidae|GMPJA9919-21|BIOUG66657-H05|BOLD:ADT2928
Encyrtidae|GMPJA9654-21|BIOUG66655-B02|BOLD:ADT2928
Encyrtidae|GMPJA9759-21|BIOUG66656-B12|BOLD:ADT2928
Encyrtidae|GMPJA9274-21|BIOUG66651-B02|BOLD:ADT2928

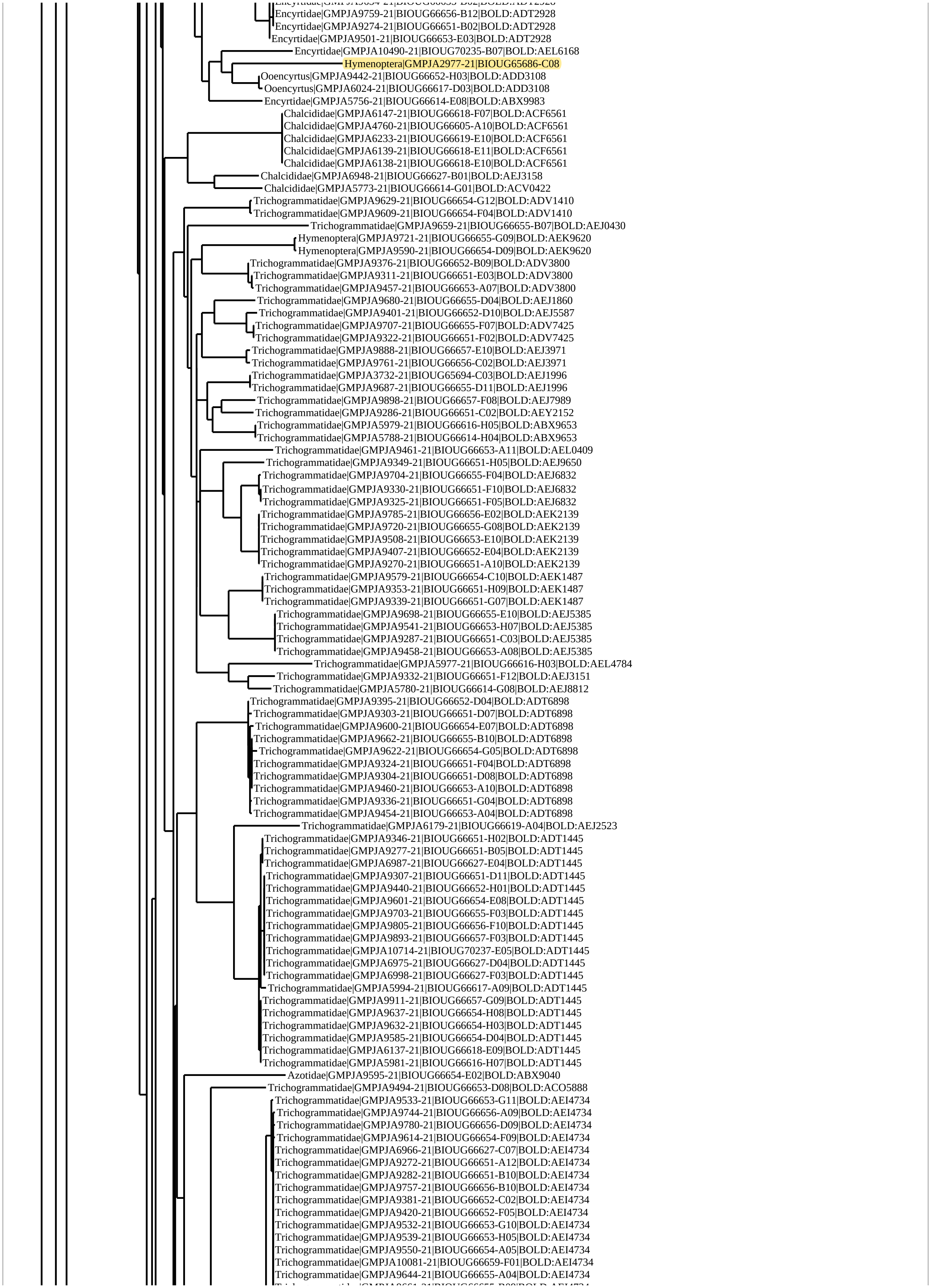
Encyrtidae|GMPJA9759-21|BIOUG66656-B12|BOLD:ADT2928
Encyrtidae|GMPJA9274-21|BIOUG66651-B02|BOLD:ADT2928
Encyrtidae|GMPJA9501-21|BIOUG66653-E03|BOLD:ADT2928
Encyrtidae|GMPJA10490-21|BIOUG70235-B07|BOLD:AEL6168
Hymenoptera|GMPJA2977-21|BIOUG65686-C08
Ooencyrtus|GMPJA9442-21|BIOUG66652-H03|BOLD:ADD3108
Ooencyrtus|GMPJA6024-21|BIOUG66617-D03|BOLD:ADD3108
Encyrtidae|GMPJA5756-21|BIOUG66614-E08|BOLD:ABX9983
Chalcididae|GMPJA6147-21|BIOUG66618-F07|BOLD:ACF6561
Chalcididae|GMPJA4760-21|BIOUG66605-A10|BOLD:ACF6561
Chalcididae|GMPJA6233-21|BIOUG66619-E10|BOLD:ACF6561
Chalcididae|GMPJA6139-21|BIOUG66618-E11|BOLD:ACF6561
Chalcididae|GMPJA6138-21|BIOUG66618-E10|BOLD:ACF6561
Chalcididae|GMPJA6948-21|BIOUG66627-B01|BOLD:AEJ3158
Chalcididae|GMPJA5773-21|BIOUG66614-G01|BOLD:ACV0422
Trichogrammatidae|GMPJA9629-21|BIOUG66654-G12|BOLD:ADV1410
Trichogrammatidae|GMPJA9609-21|BIOUG66654-F04|BOLD:ADV1410
Trichogrammatidae|GMPJA9659-21|BIOUG66655-B07|BOLD:AEJ0430
Hymenoptera|GMPJA9721-21|BIOUG66655-G09|BOLD:AEK9620
Hymenoptera|GMPJA9590-21|BIOUG66654-D09|BOLD:AEK9620
Trichogrammatidae|GMPJA9376-21|BIOUG66652-B09|BOLD:ADV3800
Trichogrammatidae|GMPJA9311-21|BIOUG66651-E03|BOLD:ADV3800
Trichogrammatidae|GMPJA9457-21|BIOUG66653-A07|BOLD:ADV3800
Trichogrammatidae|GMPJA9680-21|BIOUG66655-D04|BOLD:AEJ1860
Trichogrammatidae|GMPJA9401-21|BIOUG66652-D10|BOLD:AEJ5587
Trichogrammatidae|GMPJA9707-21|BIOUG66655-F07|BOLD:ADV7425
Trichogrammatidae|GMPJA9322-21|BIOUG66651-F02|BOLD:ADV7425
Trichogrammatidae|GMPJA9888-21|BIOUG66657-E10|BOLD:AEJ3971
Trichogrammatidae|GMPJA9761-21|BIOUG66656-C02|BOLD:AEJ3971
Trichogrammatidae|GMPJA3732-21|BIOUG65694-C03|BOLD:AEJ1996
Trichogrammatidae|GMPJA9687-21|BIOUG66655-D11|BOLD:AEJ1996
Trichogrammatidae|GMPJA9898-21|BIOUG66657-F08|BOLD:AEJ7989
Trichogrammatidae|GMPJA9286-21|BIOUG66651-C02|BOLD:AEY2152
Trichogrammatidae|GMPJA5979-21|BIOUG66616-H05|BOLD:ABX9653
Trichogrammatidae|GMPJA5788-21|BIOUG66614-H04|BOLD:ABX9653
Trichogrammatidae|GMPJA9461-21|BIOUG66653-A11|BOLD:AEL0409
Trichogrammatidae|GMPJA9349-21|BIOUG66651-H05|BOLD:AEJ9650
Trichogrammatidae|GMPJA9704-21|BIOUG66655-F04|BOLD:AEJ6832
Trichogrammatidae|GMPJA9330-21|BIOUG66651-F10|BOLD:AEJ6832
Trichogrammatidae|GMPJA9325-21|BIOUG66651-F05|BOLD:AEJ6832
Trichogrammatidae|GMPJA9785-21|BIOUG66656-E02|BOLD:AEK2139
Trichogrammatidae|GMPJA9720-21|BIOUG66655-G08|BOLD:AEK2139
Trichogrammatidae|GMPJA9508-21|BIOUG66653-E10|BOLD:AEK2139
Trichogrammatidae|GMPJA9407-21|BIOUG66652-E04|BOLD:AEK2139
Trichogrammatidae|GMPJA9270-21|BIOUG66651-A10|BOLD:AEK2139
Trichogrammatidae|GMPJA9579-21|BIOUG66654-C10|BOLD:AEK1487
Trichogrammatidae|GMPJA9353-21|BIOUG66651-H09|BOLD:AEK1487
Trichogrammatidae|GMPJA9339-21|BIOUG66651-G07|BOLD:AEK1487
Trichogrammatidae|GMPJA9698-21|BIOUG66655-E10|BOLD:AEJ5385
Trichogrammatidae|GMPJA9541-21|BIOUG66653-H07|BOLD:AEJ5385
Trichogrammatidae|GMPJA9287-21|BIOUG66651-C03|BOLD:AEJ5385
Trichogrammatidae|GMPJA9458-21|BIOUG66653-A08|BOLD:AEJ5385
Trichogrammatidae|GMPJA5977-21|BIOUG66616-H03|BOLD:AEL4784
Trichogrammatidae|GMPJA9332-21|BIOUG66651-F12|BOLD:AEJ3151
Trichogrammatidae|GMPJA5780-21|BIOUG66614-G08|BOLD:AEJ8812
Trichogrammatidae|GMPJA9395-21|BIOUG66652-D04|BOLD:ADT6898
Trichogrammatidae|GMPJA9303-21|BIOUG66651-D07|BOLD:ADT6898
Trichogrammatidae|GMPJA9600-21|BIOUG66654-E07|BOLD:ADT6898
Trichogrammatidae|GMPJA9662-21|BIOUG66655-B10|BOLD:ADT6898
Trichogrammatidae|GMPJA9622-21|BIOUG66654-G05|BOLD:ADT6898
Trichogrammatidae|GMPJA9324-21|BIOUG66651-F04|BOLD:ADT6898
Trichogrammatidae|GMPJA9304-21|BIOUG66651-D08|BOLD:ADT6898
Trichogrammatidae|GMPJA9460-21|BIOUG66653-A10|BOLD:ADT6898
Trichogrammatidae|GMPJA9336-21|BIOUG66651-G04|BOLD:ADT6898
Trichogrammatidae|GMPJA9454-21|BIOUG66653-A04|BOLD:ADT6898
Trichogrammatidae|GMPJA6179-21|BIOUG66619-A04|BOLD:AEJ2523
Trichogrammatidae|GMPJA9346-21|BIOUG66651-H02|BOLD:ADT1445
Trichogrammatidae|GMPJA9277-21|BIOUG66651-B05|BOLD:ADT1445
Trichogrammatidae|GMPJA6987-21|BIOUG66627-E04|BOLD:ADT1445
Trichogrammatidae|GMPJA9307-21|BIOUG66651-D11|BOLD:ADT1445
Trichogrammatidae|GMPJA9440-21|BIOUG66652-H01|BOLD:ADT1445
Trichogrammatidae|GMPJA9601-21|BIOUG66654-E08|BOLD:ADT1445
Trichogrammatidae|GMPJA9703-21|BIOUG66655-F03|BOLD:ADT1445
Trichogrammatidae|GMPJA9805-21|BIOUG66656-F10|BOLD:ADT1445
Trichogrammatidae|GMPJA9893-21|BIOUG66657-F03|BOLD:ADT1445
Trichogrammatidae|GMPJA10714-21|BIOUG70237-E05|BOLD:ADT1445
Trichogrammatidae|GMPJA6975-21|BIOUG66627-D04|BOLD:ADT1445
Trichogrammatidae|GMPJA6998-21|BIOUG66627-F03|BOLD:ADT1445
Trichogrammatidae|GMPJA5994-21|BIOUG66617-A09|BOLD:ADT1445
Trichogrammatidae|GMPJA9911-21|BIOUG66657-G09|BOLD:ADT1445
Trichogrammatidae|GMPJA9637-21|BIOUG66654-H08|BOLD:ADT1445
Trichogrammatidae|GMPJA9632-21|BIOUG66654-H03|BOLD:ADT1445
Trichogrammatidae|GMPJA9585-21|BIOUG66654-D04|BOLD:ADT1445
Trichogrammatidae|GMPJA6137-21|BIOUG66618-E09|BOLD:ADT1445
Trichogrammatidae|GMPJA5981-21|BIOUG66616-H07|BOLD:ADT1445
Azotidae|GMPJA9595-21|BIOUG66654-E02|BOLD:ABX9040
Trichogrammatidae|GMPJA9494-21|BIOUG66653-D08|BOLD:ACO5888
Trichogrammatidae|GMPJA9533-21|BIOUG66653-G11|BOLD:AEI4734
Trichogrammatidae|GMPJA9744-21|BIOUG66656-A09|BOLD:AEI4734
Trichogrammatidae|GMPJA9780-21|BIOUG66656-D09|BOLD:AEI4734
Trichogrammatidae|GMPJA9614-21|BIOUG66654-F09|BOLD:AEI4734
Trichogrammatidae|GMPJA6966-21|BIOUG66627-C07|BOLD:AEI4734
Trichogrammatidae|GMPJA9272-21|BIOUG66651-A12|BOLD:AEI4734
Trichogrammatidae|GMPJA9282-21|BIOUG66651-B10|BOLD:AEI4734
Trichogrammatidae|GMPJA9757-21|BIOUG66656-B10|BOLD:AEI4734
Trichogrammatidae|GMPJA9381-21|BIOUG66652-C02|BOLD:AEI4734
Trichogrammatidae|GMPJA9420-21|BIOUG66652-F05|BOLD:AEI4734
Trichogrammatidae|GMPJA9532-21|BIOUG66653-G10|BOLD:AEI4734
Trichogrammatidae|GMPJA9539-21|BIOUG66653-H05|BOLD:AEI4734
Trichogrammatidae|GMPJA9550-21|BIOUG66654-A05|BOLD:AEI4734
Trichogrammatidae|GMPJA10081-21|BIOUG66659-F01|BOLD:AEI4734
Trichogrammatidae|GMPJA9644-21|BIOUG66655-A04|BOLD:AEI4734

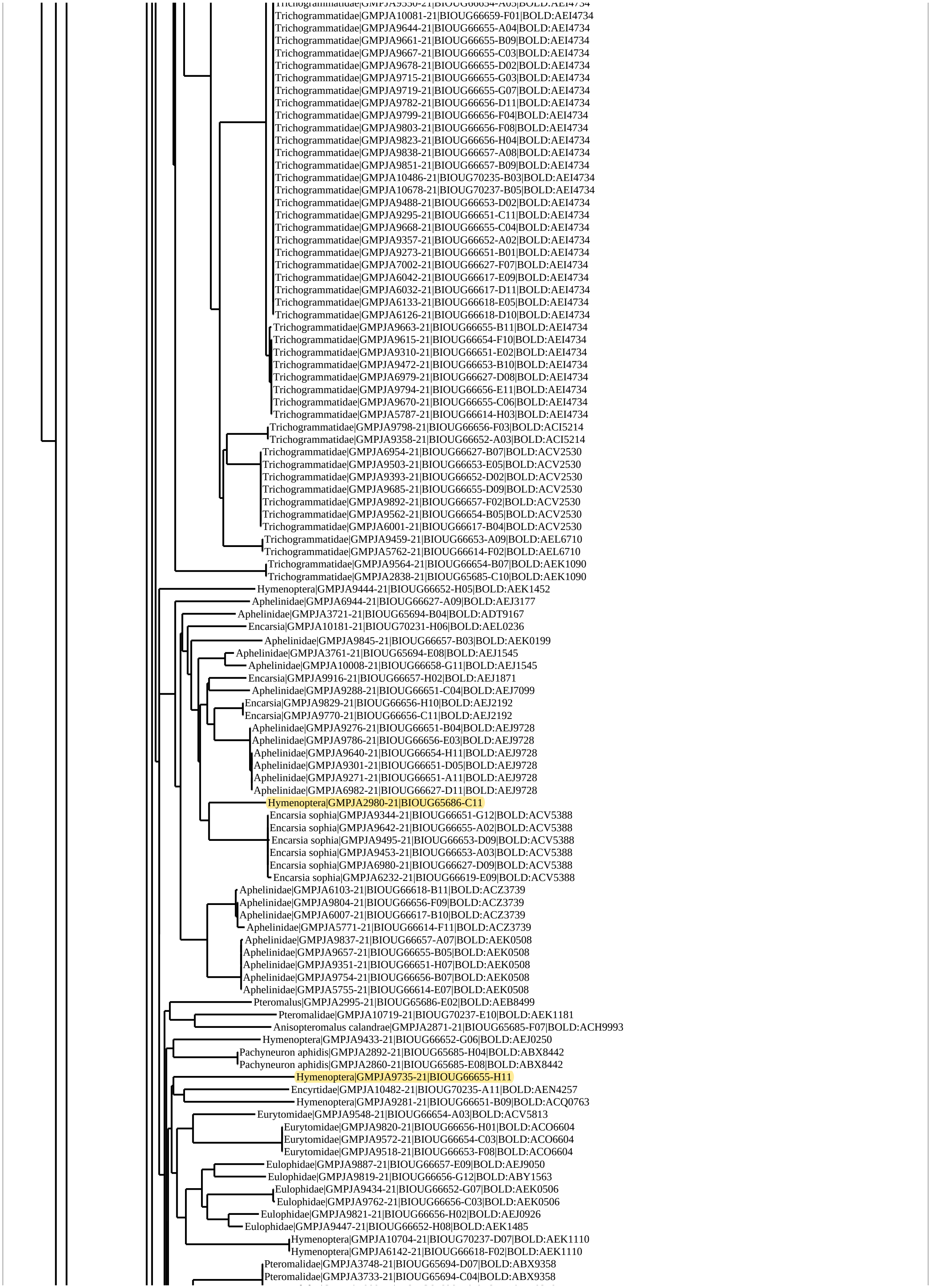
Trichogrammatidae|GMPJA95[illegible]-21|BIOUG66654-A05|BOLD:AEI4734
Trichogrammatidae|GMPJA10081-21|BIOUG66659-F01|BOLD:AEI4734
Trichogrammatidae|GMPJA9644-21|BIOUG66655-A04|BOLD:AEI4734
Trichogrammatidae|GMPJA9661-21|BIOUG66655-B09|BOLD:AEI4734
Trichogrammatidae|GMPJA9667-21|BIOUG66655-C03|BOLD:AEI4734
Trichogrammatidae|GMPJA9678-21|BIOUG66655-D02|BOLD:AEI4734
Trichogrammatidae|GMPJA9715-21|BIOUG66655-G03|BOLD:AEI4734
Trichogrammatidae|GMPJA9719-21|BIOUG66655-G07|BOLD:AEI4734
Trichogrammatidae|GMPJA9782-21|BIOUG66656-D11|BOLD:AEI4734
Trichogrammatidae|GMPJA9799-21|BIOUG66656-F04|BOLD:AEI4734
Trichogrammatidae|GMPJA9803-21|BIOUG66656-F08|BOLD:AEI4734
Trichogrammatidae|GMPJA9823-21|BIOUG66656-H04|BOLD:AEI4734
Trichogrammatidae|GMPJA9838-21|BIOUG66657-A08|BOLD:AEI4734
Trichogrammatidae|GMPJA9851-21|BIOUG66657-B09|BOLD:AEI4734
Trichogrammatidae|GMPJA10486-21|BIOUG70235-B03|BOLD:AEI4734
Trichogrammatidae|GMPJA10678-21|BIOUG70237-B05|BOLD:AEI4734
Trichogrammatidae|GMPJA9488-21|BIOUG66653-D02|BOLD:AEI4734
Trichogrammatidae|GMPJA9295-21|BIOUG66651-C11|BOLD:AEI4734
Trichogrammatidae|GMPJA9668-21|BIOUG66655-C04|BOLD:AEI4734
Trichogrammatidae|GMPJA9357-21|BIOUG66652-A02|BOLD:AEI4734
Trichogrammatidae|GMPJA9273-21|BIOUG66651-B01|BOLD:AEI4734
Trichogrammatidae|GMPJA7002-21|BIOUG66627-F07|BOLD:AEI4734
Trichogrammatidae|GMPJA6042-21|BIOUG66617-E09|BOLD:AEI4734
Trichogrammatidae|GMPJA6032-21|BIOUG66617-D11|BOLD:AEI4734
Trichogrammatidae|GMPJA6133-21|BIOUG66618-E05|BOLD:AEI4734
Trichogrammatidae|GMPJA6126-21|BIOUG66618-D10|BOLD:AEI4734
Trichogrammatidae|GMPJA9663-21|BIOUG66655-B11|BOLD:AEI4734
Trichogrammatidae|GMPJA9615-21|BIOUG66654-F10|BOLD:AEI4734
Trichogrammatidae|GMPJA9310-21|BIOUG66651-E02|BOLD:AEI4734
Trichogrammatidae|GMPJA9472-21|BIOUG66653-B10|BOLD:AEI4734
Trichogrammatidae|GMPJA6979-21|BIOUG66627-D08|BOLD:AEI4734
Trichogrammatidae|GMPJA9794-21|BIOUG66656-E11|BOLD:AEI4734
Trichogrammatidae|GMPJA9670-21|BIOUG66655-C06|BOLD:AEI4734
Trichogrammatidae|GMPJA5787-21|BIOUG66614-H03|BOLD:AEI4734
Trichogrammatidae|GMPJA9798-21|BIOUG66656-F03|BOLD:ACI5214
Trichogrammatidae|GMPJA9358-21|BIOUG66652-A03|BOLD:ACI5214
Trichogrammatidae|GMPJA6954-21|BIOUG66627-B07|BOLD:ACV2530
Trichogrammatidae|GMPJA9503-21|BIOUG66653-E05|BOLD:ACV2530
Trichogrammatidae|GMPJA9393-21|BIOUG66652-D02|BOLD:ACV2530
Trichogrammatidae|GMPJA9685-21|BIOUG66655-D09|BOLD:ACV2530
Trichogrammatidae|GMPJA9892-21|BIOUG66657-F02|BOLD:ACV2530
Trichogrammatidae|GMPJA9562-21|BIOUG66654-B05|BOLD:ACV2530
Trichogrammatidae|GMPJA6001-21|BIOUG66617-B04|BOLD:ACV2530
Trichogrammatidae|GMPJA9459-21|BIOUG66653-A09|BOLD:AEL6710
Trichogrammatidae|GMPJA5762-21|BIOUG66614-F02|BOLD:AEL6710
Trichogrammatidae|GMPJA9564-21|BIOUG66654-B07|BOLD:AEK1090
Trichogrammatidae|GMPJA2838-21|BIOUG65685-C10|BOLD:AEK1090
Hymenoptera|GMPJA9444-21|BIOUG66652-H05|BOLD:AEK1452
Aphelinidae|GMPJA6944-21|BIOUG66627-A09|BOLD:AEJ3177
Aphelinidae|GMPJA3721-21|BIOUG65694-B04|BOLD:ADT9167
Encarsia|GMPJA10181-21|BIOUG70231-H06|BOLD:AEL0236
Aphelinidae|GMPJA9845-21|BIOUG66657-B03|BOLD:AEK0199
Aphelinidae|GMPJA3761-21|BIOUG65694-E08|BOLD:AEJ1545
Aphelinidae|GMPJA10008-21|BIOUG66658-G11|BOLD:AEJ1545
Encarsia|GMPJA9916-21|BIOUG66657-H02|BOLD:AEJ1871
Aphelinidae|GMPJA9288-21|BIOUG66651-C04|BOLD:AEJ7099
Encarsia|GMPJA9829-21|BIOUG66656-H10|BOLD:AEJ2192
Encarsia|GMPJA9770-21|BIOUG66656-C11|BOLD:AEJ2192
Aphelinidae|GMPJA9276-21|BIOUG66651-B04|BOLD:AEJ9728
Aphelinidae|GMPJA9786-21|BIOUG66656-E03|BOLD:AEJ9728
Aphelinidae|GMPJA9640-21|BIOUG66654-H11|BOLD:AEJ9728
Aphelinidae|GMPJA9301-21|BIOUG66651-D05|BOLD:AEJ9728
Aphelinidae|GMPJA9271-21|BIOUG66651-A11|BOLD:AEJ9728
Aphelinidae|GMPJA6982-21|BIOUG66627-D11|BOLD:AEJ9728
Hymenoptera|GMPJA2980-21|BIOUG65686-C11
Encarsia sophia|GMPJA9344-21|BIOUG66651-G12|BOLD:ACV5388
Encarsia sophia|GMPJA9642-21|BIOUG66655-A02|BOLD:ACV5388
Encarsia sophia|GMPJA9495-21|BIOUG66653-D09|BOLD:ACV5388
Encarsia sophia|GMPJA9453-21|BIOUG66653-A03|BOLD:ACV5388
Encarsia sophia|GMPJA6980-21|BIOUG66627-D09|BOLD:ACV5388
Encarsia sophia|GMPJA6232-21|BIOUG66619-E09|BOLD:ACV5388
Aphelinidae|GMPJA6103-21|BIOUG66618-B11|BOLD:ACZ3739
Aphelinidae|GMPJA9804-21|BIOUG66656-F09|BOLD:ACZ3739
Aphelinidae|GMPJA6007-21|BIOUG66617-B10|BOLD:ACZ3739
Aphelinidae|GMPJA5771-21|BIOUG66614-F11|BOLD:ACZ3739
Aphelinidae|GMPJA9837-21|BIOUG66657-A07|BOLD:AEK0508
Aphelinidae|GMPJA9657-21|BIOUG66655-B05|BOLD:AEK0508
Aphelinidae|GMPJA9351-21|BIOUG66651-H07|BOLD:AEK0508
Aphelinidae|GMPJA9754-21|BIOUG66656-B07|BOLD:AEK0508
Aphelinidae|GMPJA5755-21|BIOUG66614-E07|BOLD:AEK0508
Pteromalus|GMPJA2995-21|BIOUG65686-E02|BOLD:AEB8499
Pteromalidae|GMPJA10719-21|BIOUG70237-E10|BOLD:AEK1181
Anisopteromalus calandrae|GMPJA2871-21|BIOUG65685-F07|BOLD:ACH9993
Hymenoptera|GMPJA9433-21|BIOUG66652-G06|BOLD:AEJ0250
Pachyneuron aphidis|GMPJA2892-21|BIOUG65685-H04|BOLD:ABX8442
Pachyneuron aphidis|GMPJA2860-21|BIOUG65685-E08|BOLD:ABX8442
Hymenoptera|GMPJA9735-21|BIOUG66655-H11
Encyrtidae|GMPJA10482-21|BIOUG70235-A11|BOLD:AEN4257
Hymenoptera|GMPJA9281-21|BIOUG66651-B09|BOLD:ACQ0763
Eurytomidae|GMPJA9548-21|BIOUG66654-A03|BOLD:ACV5813
Eurytomidae|GMPJA9820-21|BIOUG66656-H01|BOLD:ACO6604
Eurytomidae|GMPJA9572-21|BIOUG66654-C03|BOLD:ACO6604
Eurytomidae|GMPJA9518-21|BIOUG66653-F08|BOLD:ACO6604
Eulophidae|GMPJA9887-21|BIOUG66657-E09|BOLD:AEJ9050
Eulophidae|GMPJA9819-21|BIOUG66656-G12|BOLD:ABY1563
Eulophidae|GMPJA9434-21|BIOUG66652-G07|BOLD:AEK0506
Eulophidae|GMPJA9762-21|BIOUG66656-C03|BOLD:AEK0506
Eulophidae|GMPJA9821-21|BIOUG66656-H02|BOLD:AEJ0926
Eulophidae|GMPJA9447-21|BIOUG66652-H08|BOLD:AEK1485
Hymenoptera|GMPJA10704-21|BIOUG70237-D07|BOLD:AEK1110
Hymenoptera|GMPJA6142-21|BIOUG66618-F02|BOLD:AEK1110
Pteromalidae|GMPJA3748-21|BIOUG65694-D07|BOLD:ABX9358
Pteromalidae|GMPJA3733-21|BIOUG65694-C04|BOLD:ABX9358

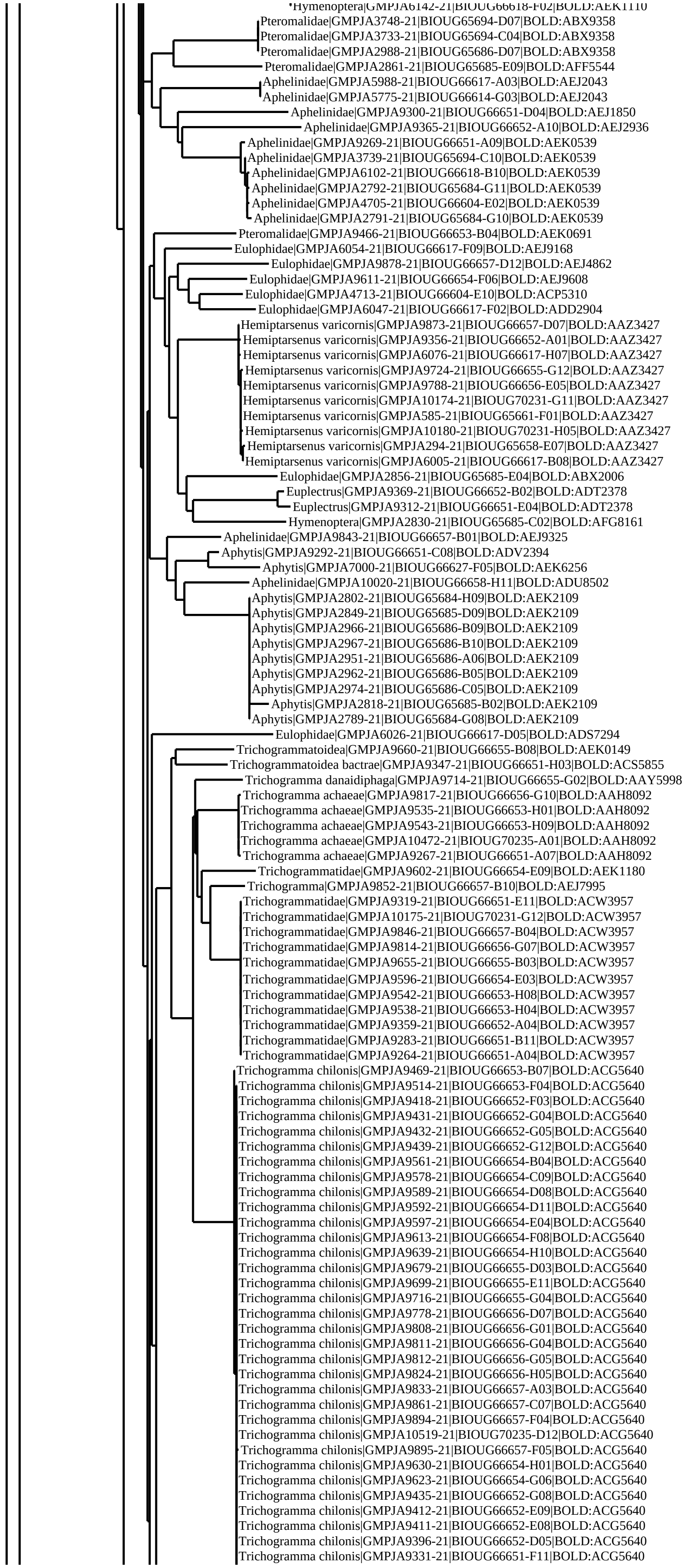
Hymenoptera|GMPJA6142-21|BIOUG66618-F02|BOLD:AEK1110
Pteromalidae|GMPJA3748-21|BIOUG65694-D07|BOLD:ABX9358
Pteromalidae|GMPJA3733-21|BIOUG65694-C04|BOLD:ABX9358
Pteromalidae|GMPJA2988-21|BIOUG65686-D07|BOLD:ABX9358
Pteromalidae|GMPJA2861-21|BIOUG65685-E09|BOLD:AFF5544
Aphelinidae|GMPJA5988-21|BIOUG66617-A03|BOLD:AEJ2043
Aphelinidae|GMPJA5775-21|BIOUG66614-G03|BOLD:AEJ2043
Aphelinidae|GMPJA9300-21|BIOUG66651-D04|BOLD:AEJ1850
Aphelinidae|GMPJA9365-21|BIOUG66652-A10|BOLD:AEJ2936
Aphelinidae|GMPJA9269-21|BIOUG66651-A09|BOLD:AEK0539
Aphelinidae|GMPJA3739-21|BIOUG65694-C10|BOLD:AEK0539
Aphelinidae|GMPJA6102-21|BIOUG66618-B10|BOLD:AEK0539
Aphelinidae|GMPJA2792-21|BIOUG65684-G11|BOLD:AEK0539
Aphelinidae|GMPJA4705-21|BIOUG66604-E02|BOLD:AEK0539
Aphelinidae|GMPJA2791-21|BIOUG65684-G10|BOLD:AEK0539
Pteromalidae|GMPJA9466-21|BIOUG66653-B04|BOLD:AEK0691
Eulophidae|GMPJA6054-21|BIOUG66617-F09|BOLD:AEJ9168
Eulophidae|GMPJA9878-21|BIOUG66657-D12|BOLD:AEJ4862
Eulophidae|GMPJA9611-21|BIOUG66654-F06|BOLD:AEJ9608
Eulophidae|GMPJA4713-21|BIOUG66604-E10|BOLD:ACP5310
Eulophidae|GMPJA6047-21|BIOUG66617-F02|BOLD:ADD2904
Hemiptarsenus varicornis|GMPJA9873-21|BIOUG66657-D07|BOLD:AAZ3427
Hemiptarsenus varicornis|GMPJA9356-21|BIOUG66652-A01|BOLD:AAZ3427
Hemiptarsenus varicornis|GMPJA6076-21|BIOUG66617-H07|BOLD:AAZ3427
Hemiptarsenus varicornis|GMPJA9724-21|BIOUG66655-G12|BOLD:AAZ3427
Hemiptarsenus varicornis|GMPJA9788-21|BIOUG66656-E05|BOLD:AAZ3427
Hemiptarsenus varicornis|GMPJA10174-21|BIOUG70231-G11|BOLD:AAZ3427
Hemiptarsenus varicornis|GMPJA585-21|BIOUG65661-F01|BOLD:AAZ3427
Hemiptarsenus varicornis|GMPJA10180-21|BIOUG70231-H05|BOLD:AAZ3427
Hemiptarsenus varicornis|GMPJA294-21|BIOUG65658-E07|BOLD:AAZ3427
Hemiptarsenus varicornis|GMPJA6005-21|BIOUG66617-B08|BOLD:AAZ3427
Eulophidae|GMPJA2856-21|BIOUG65685-E04|BOLD:ABX2006
Euplectrus|GMPJA9369-21|BIOUG66652-B02|BOLD:ADT2378
Euplectrus|GMPJA9312-21|BIOUG66651-E04|BOLD:ADT2378
Hymenoptera|GMPJA2830-21|BIOUG65685-C02|BOLD:AFG8161
Aphelinidae|GMPJA9843-21|BIOUG66657-B01|BOLD:AEJ9325
Aphytis|GMPJA9292-21|BIOUG66651-C08|BOLD:ADV2394
Aphytis|GMPJA7000-21|BIOUG66627-F05|BOLD:AEK6256
Aphelinidae|GMPJA10020-21|BIOUG66658-H11|BOLD:ADU8502
Aphytis|GMPJA2802-21|BIOUG65684-H09|BOLD:AEK2109
Aphytis|GMPJA2849-21|BIOUG65685-D09|BOLD:AEK2109
Aphytis|GMPJA2966-21|BIOUG65686-B09|BOLD:AEK2109
Aphytis|GMPJA2967-21|BIOUG65686-B10|BOLD:AEK2109
Aphytis|GMPJA2951-21|BIOUG65686-A06|BOLD:AEK2109
Aphytis|GMPJA2962-21|BIOUG65686-B05|BOLD:AEK2109
Aphytis|GMPJA2974-21|BIOUG65686-C05|BOLD:AEK2109
Aphytis|GMPJA2818-21|BIOUG65685-B02|BOLD:AEK2109
Aphytis|GMPJA2789-21|BIOUG65684-G08|BOLD:AEK2109
Eulophidae|GMPJA6026-21|BIOUG66617-D05|BOLD:ADS7294
Trichogrammatoidea|GMPJA9660-21|BIOUG66655-B08|BOLD:AEK0149
Trichogrammatoidea bactrae|GMPJA9347-21|BIOUG66651-H03|BOLD:ACS5855
Trichogramma danaidiphaga|GMPJA9714-21|BIOUG66655-G02|BOLD:AAY5998
Trichogramma achaeae|GMPJA9817-21|BIOUG66656-G10|BOLD:AAH8092
Trichogramma achaeae|GMPJA9535-21|BIOUG66653-H01|BOLD:AAH8092
Trichogramma achaeae|GMPJA9543-21|BIOUG66653-H09|BOLD:AAH8092
Trichogramma achaeae|GMPJA10472-21|BIOUG70235-A01|BOLD:AAH8092
Trichogramma achaeae|GMPJA9267-21|BIOUG66651-A07|BOLD:AAH8092
Trichogrammatidae|GMPJA9602-21|BIOUG66654-E09|BOLD:AEK1180
Trichogramma|GMPJA9852-21|BIOUG66657-B10|BOLD:AEJ7995
Trichogrammatidae|GMPJA9319-21|BIOUG66651-E11|BOLD:ACW3957
Trichogrammatidae|GMPJA10175-21|BIOUG70231-G12|BOLD:ACW3957
Trichogrammatidae|GMPJA9846-21|BIOUG66657-B04|BOLD:ACW3957
Trichogrammatidae|GMPJA9814-21|BIOUG66656-G07|BOLD:ACW3957
Trichogrammatidae|GMPJA9655-21|BIOUG66655-B03|BOLD:ACW3957
Trichogrammatidae|GMPJA9596-21|BIOUG66654-E03|BOLD:ACW3957
Trichogrammatidae|GMPJA9542-21|BIOUG66653-H08|BOLD:ACW3957
Trichogrammatidae|GMPJA9538-21|BIOUG66653-H04|BOLD:ACW3957
Trichogrammatidae|GMPJA9359-21|BIOUG66652-A04|BOLD:ACW3957
Trichogrammatidae|GMPJA9283-21|BIOUG66651-B11|BOLD:ACW3957
Trichogrammatidae|GMPJA9264-21|BIOUG66651-A04|BOLD:ACW3957
Trichogramma chilonis|GMPJA9469-21|BIOUG66653-B07|BOLD:ACG5640
Trichogramma chilonis|GMPJA9514-21|BIOUG66653-F04|BOLD:ACG5640
Trichogramma chilonis|GMPJA9418-21|BIOUG66652-F03|BOLD:ACG5640
Trichogramma chilonis|GMPJA9431-21|BIOUG66652-G04|BOLD:ACG5640
Trichogramma chilonis|GMPJA9432-21|BIOUG66652-G05|BOLD:ACG5640
Trichogramma chilonis|GMPJA9439-21|BIOUG66652-G12|BOLD:ACG5640
Trichogramma chilonis|GMPJA9561-21|BIOUG66654-B04|BOLD:ACG5640
Trichogramma chilonis|GMPJA9578-21|BIOUG66654-C09|BOLD:ACG5640
Trichogramma chilonis|GMPJA9589-21|BIOUG66654-D08|BOLD:ACG5640
Trichogramma chilonis|GMPJA9592-21|BIOUG66654-D11|BOLD:ACG5640
Trichogramma chilonis|GMPJA9597-21|BIOUG66654-E04|BOLD:ACG5640
Trichogramma chilonis|GMPJA9613-21|BIOUG66654-F08|BOLD:ACG5640
Trichogramma chilonis|GMPJA9639-21|BIOUG66654-H10|BOLD:ACG5640
Trichogramma chilonis|GMPJA9679-21|BIOUG66655-D03|BOLD:ACG5640
Trichogramma chilonis|GMPJA9699-21|BIOUG66655-E11|BOLD:ACG5640
Trichogramma chilonis|GMPJA9716-21|BIOUG66655-G04|BOLD:ACG5640
Trichogramma chilonis|GMPJA9778-21|BIOUG66656-D07|BOLD:ACG5640
Trichogramma chilonis|GMPJA9808-21|BIOUG66656-G01|BOLD:ACG5640
Trichogramma chilonis|GMPJA9811-21|BIOUG66656-G04|BOLD:ACG5640
Trichogramma chilonis|GMPJA9812-21|BIOUG66656-G05|BOLD:ACG5640
Trichogramma chilonis|GMPJA9824-21|BIOUG66656-H05|BOLD:ACG5640
Trichogramma chilonis|GMPJA9833-21|BIOUG66657-A03|BOLD:ACG5640
Trichogramma chilonis|GMPJA9861-21|BIOUG66657-C07|BOLD:ACG5640
Trichogramma chilonis|GMPJA9894-21|BIOUG66657-F04|BOLD:ACG5640
Trichogramma chilonis|GMPJA10519-21|BIOUG70235-D12|BOLD:ACG5640
Trichogramma chilonis|GMPJA9895-21|BIOUG66657-F05|BOLD:ACG5640
Trichogramma chilonis|GMPJA9630-21|BIOUG66654-H01|BOLD:ACG5640
Trichogramma chilonis|GMPJA9623-21|BIOUG66654-G06|BOLD:ACG5640
Trichogramma chilonis|GMPJA9435-21|BIOUG66652-G08|BOLD:ACG5640
Trichogramma chilonis|GMPJA9412-21|BIOUG66652-E09|BOLD:ACG5640
Trichogramma chilonis|GMPJA9411-21|BIOUG66652-E08|BOLD:ACG5640
Trichogramma chilonis|GMPJA9396-21|BIOUG66652-D05|BOLD:ACG5640
Trichogramma chilonis|GMPJA9331-21|BIOUG66651-F11|BOLD:ACG5640

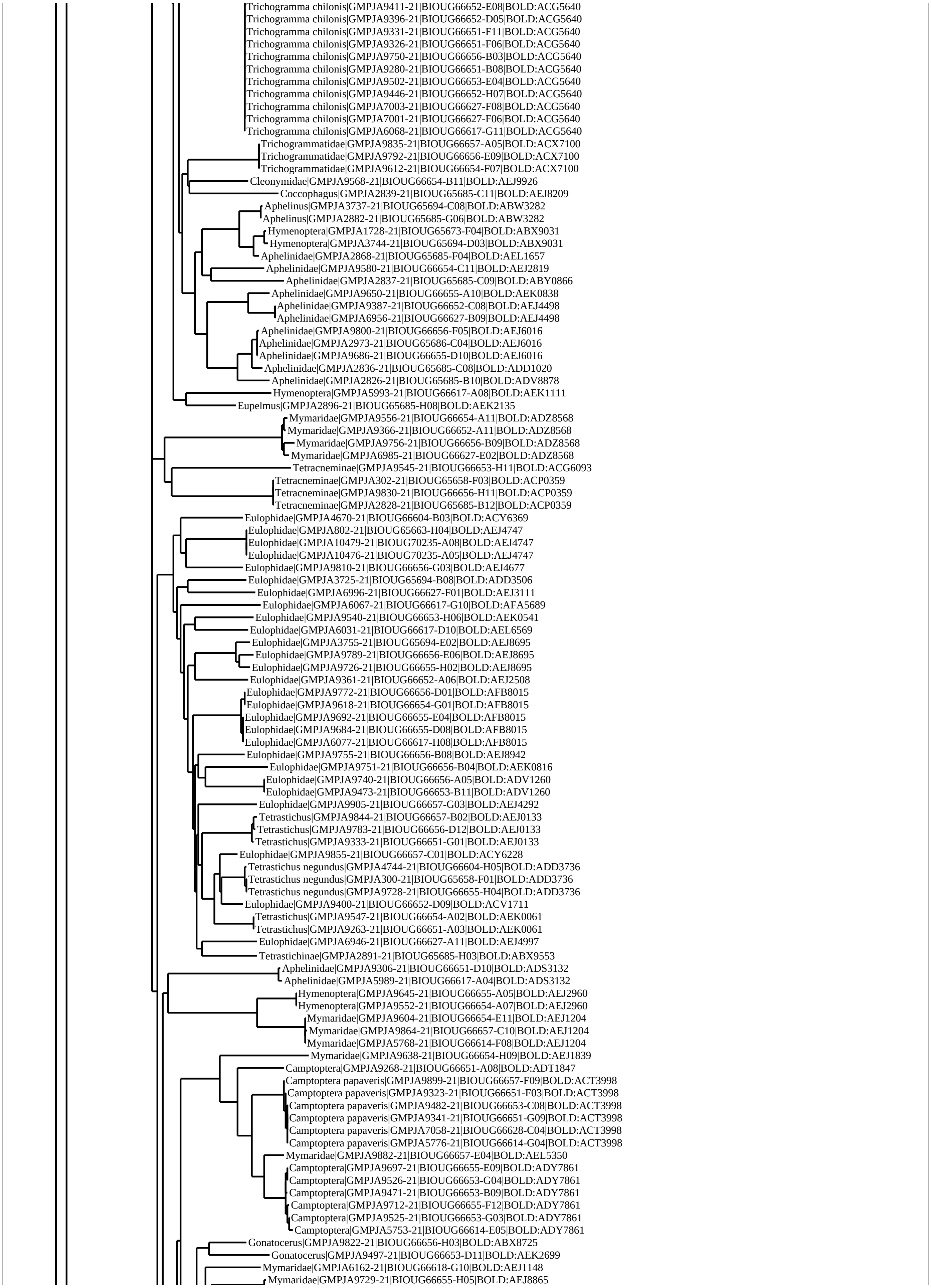
Trichogramma chilonis|GMPJA9411-21|BIOUG66652-E08|BOLD:ACG5640
Trichogramma chilonis|GMPJA9396-21|BIOUG66652-D05|BOLD:ACG5640
Trichogramma chilonis|GMPJA9331-21|BIOUG66651-F11|BOLD:ACG5640
Trichogramma chilonis|GMPJA9326-21|BIOUG66651-F06|BOLD:ACG5640
Trichogramma chilonis|GMPJA9750-21|BIOUG66656-B03|BOLD:ACG5640
Trichogramma chilonis|GMPJA9280-21|BIOUG66651-B08|BOLD:ACG5640
Trichogramma chilonis|GMPJA9502-21|BIOUG66653-E04|BOLD:ACG5640
Trichogramma chilonis|GMPJA9446-21|BIOUG66652-H07|BOLD:ACG5640
Trichogramma chilonis|GMPJA7003-21|BIOUG66627-F08|BOLD:ACG5640
Trichogramma chilonis|GMPJA7001-21|BIOUG66627-F06|BOLD:ACG5640
Trichogramma chilonis|GMPJA6068-21|BIOUG66617-G11|BOLD:ACG5640
Trichogrammatidae|GMPJA9835-21|BIOUG66657-A05|BOLD:ACX7100
Trichogrammatidae|GMPJA9792-21|BIOUG66656-E09|BOLD:ACX7100
Trichogrammatidae|GMPJA9612-21|BIOUG66654-F07|BOLD:ACX7100
Cleonymidae|GMPJA9568-21|BIOUG66654-B11|BOLD:AEJ9926
Coccophagus|GMPJA2839-21|BIOUG65685-C11|BOLD:AEJ8209
Aphelinus|GMPJA3737-21|BIOUG65694-C08|BOLD:ABW3282
Aphelinus|GMPJA2882-21|BIOUG65685-G06|BOLD:ABW3282
Hymenoptera|GMPJA1728-21|BIOUG65673-F04|BOLD:ABX9031
Hymenoptera|GMPJA3744-21|BIOUG65694-D03|BOLD:ABX9031
Aphelinidae|GMPJA2868-21|BIOUG65685-F04|BOLD:AEL1657
Aphelinidae|GMPJA9580-21|BIOUG66654-C11|BOLD:AEJ2819
Aphelinidae|GMPJA2837-21|BIOUG65685-C09|BOLD:ABY0866
Aphelinidae|GMPJA9650-21|BIOUG66655-A10|BOLD:AEK0838
Aphelinidae|GMPJA9387-21|BIOUG66652-C08|BOLD:AEJ4498
Aphelinidae|GMPJA6956-21|BIOUG66627-B09|BOLD:AEJ4498
Aphelinidae|GMPJA9800-21|BIOUG66656-F05|BOLD:AEJ6016
Aphelinidae|GMPJA2973-21|BIOUG65686-C04|BOLD:AEJ6016
Aphelinidae|GMPJA9686-21|BIOUG66655-D10|BOLD:AEJ6016
Aphelinidae|GMPJA2836-21|BIOUG65685-C08|BOLD:ADD1020
Aphelinidae|GMPJA2826-21|BIOUG65685-B10|BOLD:ADV8878
Hymenoptera|GMPJA5993-21|BIOUG66617-A08|BOLD:AEK1111
Eupelmus|GMPJA2896-21|BIOUG65685-H08|BOLD:AEK2135
Mymaridae|GMPJA9556-21|BIOUG66654-A11|BOLD:ADZ8568
Mymaridae|GMPJA9366-21|BIOUG66652-A11|BOLD:ADZ8568
Mymaridae|GMPJA9756-21|BIOUG66656-B09|BOLD:ADZ8568
Mymaridae|GMPJA6985-21|BIOUG66627-E02|BOLD:ADZ8568
Tetracneminae|GMPJA9545-21|BIOUG66653-H11|BOLD:ACG6093
Tetracneminae|GMPJA302-21|BIOUG65658-F03|BOLD:ACP0359
Tetracneminae|GMPJA9830-21|BIOUG66656-H11|BOLD:ACP0359
Tetracneminae|GMPJA2828-21|BIOUG65685-B12|BOLD:ACP0359
Eulophidae|GMPJA4670-21|BIOUG66604-B03|BOLD:ACY6369
Eulophidae|GMPJA802-21|BIOUG65663-H04|BOLD:AEJ4747
Eulophidae|GMPJA10479-21|BIOUG70235-A08|BOLD:AEJ4747
Eulophidae|GMPJA10476-21|BIOUG70235-A05|BOLD:AEJ4747
Eulophidae|GMPJA9810-21|BIOUG66656-G03|BOLD:AEJ4677
Eulophidae|GMPJA3725-21|BIOUG65694-B08|BOLD:ADD3506
Eulophidae|GMPJA6996-21|BIOUG66627-F01|BOLD:AEJ3111
Eulophidae|GMPJA6067-21|BIOUG66617-G10|BOLD:AFA5689
Eulophidae|GMPJA9540-21|BIOUG66653-H06|BOLD:AEK0541
Eulophidae|GMPJA6031-21|BIOUG66617-D10|BOLD:AEL6569
Eulophidae|GMPJA3755-21|BIOUG65694-E02|BOLD:AEJ8695
Eulophidae|GMPJA9789-21|BIOUG66656-E06|BOLD:AEJ8695
Eulophidae|GMPJA9726-21|BIOUG66655-H02|BOLD:AEJ8695
Eulophidae|GMPJA9361-21|BIOUG66652-A06|BOLD:AEJ2508
Eulophidae|GMPJA9772-21|BIOUG66656-D01|BOLD:AFB8015
Eulophidae|GMPJA9618-21|BIOUG66654-G01|BOLD:AFB8015
Eulophidae|GMPJA9692-21|BIOUG66655-E04|BOLD:AFB8015
Eulophidae|GMPJA9684-21|BIOUG66655-D08|BOLD:AFB8015
Eulophidae|GMPJA6077-21|BIOUG66617-H08|BOLD:AFB8015
Eulophidae|GMPJA9755-21|BIOUG66656-B08|BOLD:AEJ8942
Eulophidae|GMPJA9751-21|BIOUG66656-B04|BOLD:AEK0816
Eulophidae|GMPJA9740-21|BIOUG66656-A05|BOLD:ADV1260
Eulophidae|GMPJA9473-21|BIOUG66653-B11|BOLD:ADV1260
Eulophidae|GMPJA9905-21|BIOUG66657-G03|BOLD:AEJ4292
Tetrastichus|GMPJA9844-21|BIOUG66657-B02|BOLD:AEJ0133
Tetrastichus|GMPJA9783-21|BIOUG66656-D12|BOLD:AEJ0133
Tetrastichus|GMPJA9333-21|BIOUG66651-G01|BOLD:AEJ0133
Eulophidae|GMPJA9855-21|BIOUG66657-C01|BOLD:ACY6228
Tetrastichus negundus|GMPJA4744-21|BIOUG66604-H05|BOLD:ADD3736
Tetrastichus negundus|GMPJA300-21|BIOUG65658-F01|BOLD:ADD3736
Tetrastichus negundus|GMPJA9728-21|BIOUG66655-H04|BOLD:ADD3736
Eulophidae|GMPJA9400-21|BIOUG66652-D09|BOLD:ACV1711
Tetrastichus|GMPJA9547-21|BIOUG66654-A02|BOLD:AEK0061
Tetrastichus|GMPJA9263-21|BIOUG66651-A03|BOLD:AEK0061
Eulophidae|GMPJA6946-21|BIOUG66627-A11|BOLD:AEJ4997
Tetrastichinae|GMPJA2891-21|BIOUG65685-H03|BOLD:ABX9553
Aphelinidae|GMPJA9306-21|BIOUG66651-D10|BOLD:ADS3132
Aphelinidae|GMPJA5989-21|BIOUG66617-A04|BOLD:ADS3132
Hymenoptera|GMPJA9645-21|BIOUG66655-A05|BOLD:AEJ2960
Hymenoptera|GMPJA9552-21|BIOUG66654-A07|BOLD:AEJ2960
Mymaridae|GMPJA9604-21|BIOUG66654-E11|BOLD:AEJ1204
Mymaridae|GMPJA9864-21|BIOUG66657-C10|BOLD:AEJ1204
Mymaridae|GMPJA5768-21|BIOUG66614-F08|BOLD:AEJ1204
Mymaridae|GMPJA9638-21|BIOUG66654-H09|BOLD:AEJ1839
Camptoptera|GMPJA9268-21|BIOUG66651-A08|BOLD:ADT1847
Camptoptera papaveris|GMPJA9899-21|BIOUG66657-F09|BOLD:ACT3998
Camptoptera papaveris|GMPJA9323-21|BIOUG66651-F03|BOLD:ACT3998
Camptoptera papaveris|GMPJA9482-21|BIOUG66653-C08|BOLD:ACT3998
Camptoptera papaveris|GMPJA9341-21|BIOUG66651-G09|BOLD:ACT3998
Camptoptera papaveris|GMPJA7058-21|BIOUG66628-C04|BOLD:ACT3998
Camptoptera papaveris|GMPJA5776-21|BIOUG66614-G04|BOLD:ACT3998
Mymaridae|GMPJA9882-21|BIOUG66657-E04|BOLD:AEL5350
Camptoptera|GMPJA9697-21|BIOUG66655-E09|BOLD:ADY7861
Camptoptera|GMPJA9526-21|BIOUG66653-G04|BOLD:ADY7861
Camptoptera|GMPJA9471-21|BIOUG66653-B09|BOLD:ADY7861
Camptoptera|GMPJA9712-21|BIOUG66655-F12|BOLD:ADY7861
Camptoptera|GMPJA9525-21|BIOUG66653-G03|BOLD:ADY7861
Camptoptera|GMPJA5753-21|BIOUG66614-E05|BOLD:ADY7861
Gonatocerus|GMPJA9822-21|BIOUG66656-H03|BOLD:ABX8725
Gonatocerus|GMPJA9497-21|BIOUG66653-D11|BOLD:AEK2699
Mymaridae|GMPJA6162-21|BIOUG66618-G10|BOLD:AEJ1148
Mymaridae|GMPJA9729-21|BIOUG66655-H05|BOLD:AEJ8865

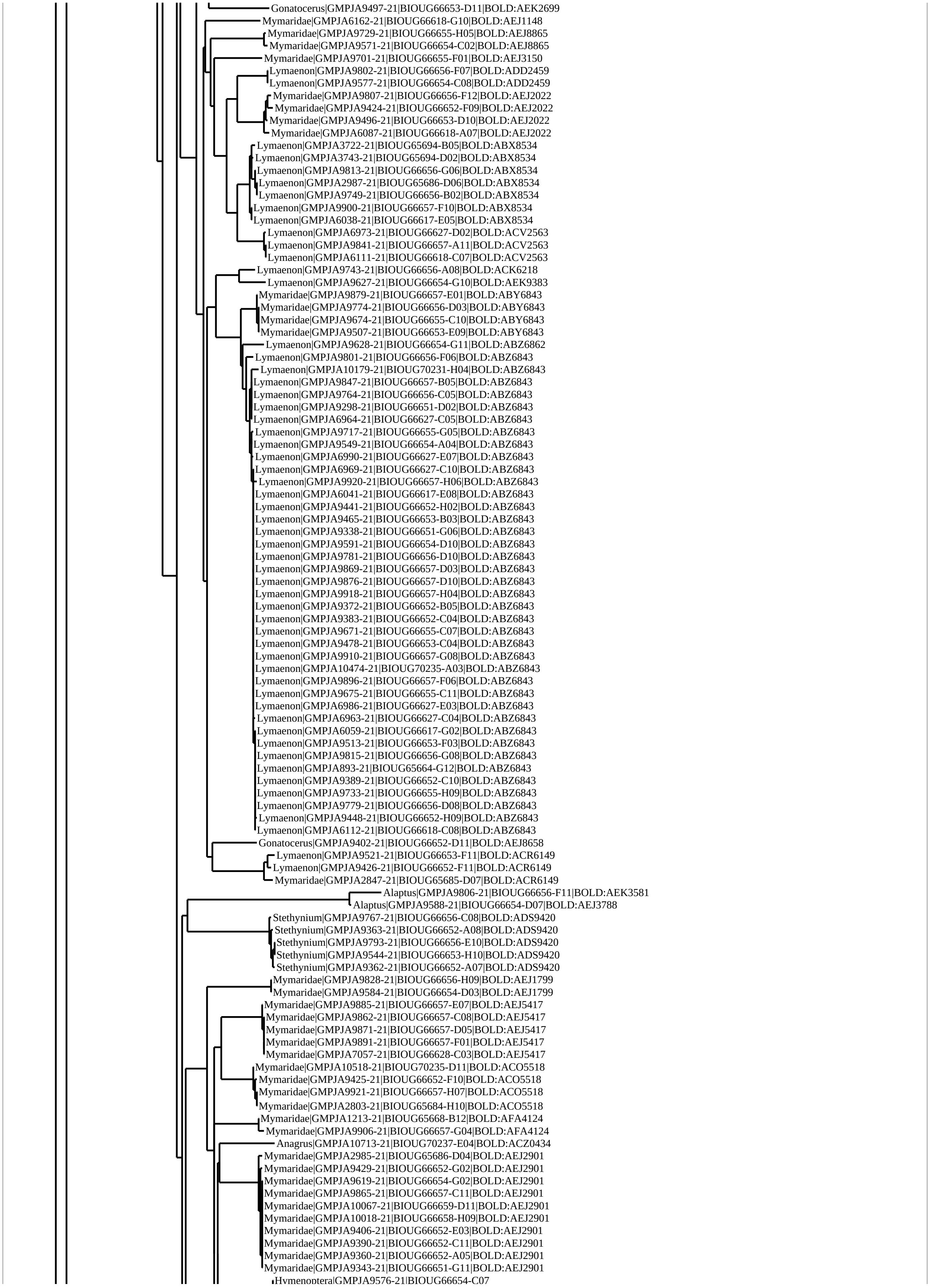
Gonatocerus|GMPJA9497-21|BIOUG66653-D11|BOLD:AEK2699
Mymaridae|GMPJA6162-21|BIOUG66618-G10|BOLD:AEJ1148
Mymaridae|GMPJA9729-21|BIOUG66655-H05|BOLD:AEJ8865
Mymaridae|GMPJA9571-21|BIOUG66654-C02|BOLD:AEJ8865
Mymaridae|GMPJA9701-21|BIOUG66655-F01|BOLD:AEJ3150
Lymaenon|GMPJA9802-21|BIOUG66656-F07|BOLD:ADD2459
Lymaenon|GMPJA9577-21|BIOUG66654-C08|BOLD:ADD2459
Mymaridae|GMPJA9807-21|BIOUG66656-F12|BOLD:AEJ2022
Mymaridae|GMPJA9424-21|BIOUG66652-F09|BOLD:AEJ2022
Mymaridae|GMPJA9496-21|BIOUG66653-D10|BOLD:AEJ2022
Mymaridae|GMPJA6087-21|BIOUG66618-A07|BOLD:AEJ2022
Lymaenon|GMPJA3722-21|BIOUG65694-B05|BOLD:ABX8534
Lymaenon|GMPJA3743-21|BIOUG65694-D02|BOLD:ABX8534
Lymaenon|GMPJA9813-21|BIOUG66656-G06|BOLD:ABX8534
Lymaenon|GMPJA2987-21|BIOUG65686-D06|BOLD:ABX8534
Lymaenon|GMPJA9749-21|BIOUG66656-B02|BOLD:ABX8534
Lymaenon|GMPJA9900-21|BIOUG66657-F10|BOLD:ABX8534
Lymaenon|GMPJA6038-21|BIOUG66617-E05|BOLD:ABX8534
Lymaenon|GMPJA6973-21|BIOUG66627-D02|BOLD:ACV2563
Lymaenon|GMPJA9841-21|BIOUG66657-A11|BOLD:ACV2563
Lymaenon|GMPJA6111-21|BIOUG66618-C07|BOLD:ACV2563
Lymaenon|GMPJA9743-21|BIOUG66656-A08|BOLD:ACK6218
Lymaenon|GMPJA9627-21|BIOUG66654-G10|BOLD:AEK9383
Mymaridae|GMPJA9879-21|BIOUG66657-E01|BOLD:ABY6843
Mymaridae|GMPJA9774-21|BIOUG66656-D03|BOLD:ABY6843
Mymaridae|GMPJA9674-21|BIOUG66655-C10|BOLD:ABY6843
Mymaridae|GMPJA9507-21|BIOUG66653-E09|BOLD:ABY6843
Lymaenon|GMPJA9628-21|BIOUG66654-G11|BOLD:ABZ6862
Lymaenon|GMPJA9801-21|BIOUG66656-F06|BOLD:ABZ6843
Lymaenon|GMPJA10179-21|BIOUG70231-H04|BOLD:ABZ6843
Lymaenon|GMPJA9847-21|BIOUG66657-B05|BOLD:ABZ6843
Lymaenon|GMPJA9764-21|BIOUG66656-C05|BOLD:ABZ6843
Lymaenon|GMPJA9298-21|BIOUG66651-D02|BOLD:ABZ6843
Lymaenon|GMPJA6964-21|BIOUG66627-C05|BOLD:ABZ6843
Lymaenon|GMPJA9717-21|BIOUG66655-G05|BOLD:ABZ6843
Lymaenon|GMPJA9549-21|BIOUG66654-A04|BOLD:ABZ6843
Lymaenon|GMPJA6990-21|BIOUG66627-E07|BOLD:ABZ6843
Lymaenon|GMPJA6969-21|BIOUG66627-C10|BOLD:ABZ6843
Lymaenon|GMPJA9920-21|BIOUG66657-H06|BOLD:ABZ6843
Lymaenon|GMPJA6041-21|BIOUG66617-E08|BOLD:ABZ6843
Lymaenon|GMPJA9441-21|BIOUG66652-H02|BOLD:ABZ6843
Lymaenon|GMPJA9465-21|BIOUG66653-B03|BOLD:ABZ6843
Lymaenon|GMPJA9338-21|BIOUG66651-G06|BOLD:ABZ6843
Lymaenon|GMPJA9591-21|BIOUG66654-D10|BOLD:ABZ6843
Lymaenon|GMPJA9781-21|BIOUG66656-D10|BOLD:ABZ6843
Lymaenon|GMPJA9869-21|BIOUG66657-D03|BOLD:ABZ6843
Lymaenon|GMPJA9876-21|BIOUG66657-D10|BOLD:ABZ6843
Lymaenon|GMPJA9918-21|BIOUG66657-H04|BOLD:ABZ6843
Lymaenon|GMPJA9372-21|BIOUG66652-B05|BOLD:ABZ6843
Lymaenon|GMPJA9383-21|BIOUG66652-C04|BOLD:ABZ6843
Lymaenon|GMPJA9671-21|BIOUG66655-C07|BOLD:ABZ6843
Lymaenon|GMPJA9478-21|BIOUG66653-C04|BOLD:ABZ6843
Lymaenon|GMPJA9910-21|BIOUG66657-G08|BOLD:ABZ6843
Lymaenon|GMPJA10474-21|BIOUG70235-A03|BOLD:ABZ6843
Lymaenon|GMPJA9896-21|BIOUG66657-F06|BOLD:ABZ6843
Lymaenon|GMPJA9675-21|BIOUG66655-C11|BOLD:ABZ6843
Lymaenon|GMPJA6986-21|BIOUG66627-E03|BOLD:ABZ6843
Lymaenon|GMPJA6963-21|BIOUG66627-C04|BOLD:ABZ6843
Lymaenon|GMPJA6059-21|BIOUG66617-G02|BOLD:ABZ6843
Lymaenon|GMPJA9513-21|BIOUG66653-F03|BOLD:ABZ6843
Lymaenon|GMPJA9815-21|BIOUG66656-G08|BOLD:ABZ6843
Lymaenon|GMPJA893-21|BIOUG65664-G12|BOLD:ABZ6843
Lymaenon|GMPJA9389-21|BIOUG66652-C10|BOLD:ABZ6843
Lymaenon|GMPJA9733-21|BIOUG66655-H09|BOLD:ABZ6843
Lymaenon|GMPJA9779-21|BIOUG66656-D08|BOLD:ABZ6843
Lymaenon|GMPJA9448-21|BIOUG66652-H09|BOLD:ABZ6843
Lymaenon|GMPJA6112-21|BIOUG66618-C08|BOLD:ABZ6843
Gonatocerus|GMPJA9402-21|BIOUG66652-D11|BOLD:AEJ8658
Lymaenon|GMPJA9521-21|BIOUG66653-F11|BOLD:ACR6149
Lymaenon|GMPJA9426-21|BIOUG66652-F11|BOLD:ACR6149
Mymaridae|GMPJA2847-21|BIOUG65685-D07|BOLD:ACR6149
Alaptus|GMPJA9806-21|BIOUG66656-F11|BOLD:AEK3581
Alaptus|GMPJA9588-21|BIOUG66654-D07|BOLD:AEJ3788
Stethynium|GMPJA9767-21|BIOUG66656-C08|BOLD:ADS9420
Stethynium|GMPJA9363-21|BIOUG66652-A08|BOLD:ADS9420
Stethynium|GMPJA9793-21|BIOUG66656-E10|BOLD:ADS9420
Stethynium|GMPJA9544-21|BIOUG66653-H10|BOLD:ADS9420
Stethynium|GMPJA9362-21|BIOUG66652-A07|BOLD:ADS9420
Mymaridae|GMPJA9828-21|BIOUG66656-H09|BOLD:AEJ1799
Mymaridae|GMPJA9584-21|BIOUG66654-D03|BOLD:AEJ1799
Mymaridae|GMPJA9885-21|BIOUG66657-E07|BOLD:AEJ5417
Mymaridae|GMPJA9862-21|BIOUG66657-C08|BOLD:AEJ5417
Mymaridae|GMPJA9871-21|BIOUG66657-D05|BOLD:AEJ5417
Mymaridae|GMPJA9891-21|BIOUG66657-F01|BOLD:AEJ5417
Mymaridae|GMPJA7057-21|BIOUG66628-C03|BOLD:AEJ5417
Mymaridae|GMPJA10518-21|BIOUG70235-D11|BOLD:ACO5518
Mymaridae|GMPJA9425-21|BIOUG66652-F10|BOLD:ACO5518
Mymaridae|GMPJA9921-21|BIOUG66657-H07|BOLD:ACO5518
Mymaridae|GMPJA2803-21|BIOUG65684-H10|BOLD:ACO5518
Mymaridae|GMPJA1213-21|BIOUG65668-B12|BOLD:AFA4124
Mymaridae|GMPJA9906-21|BIOUG66657-G04|BOLD:AFA4124
Anagrus|GMPJA10713-21|BIOUG70237-E04|BOLD:ACZ0434
Mymaridae|GMPJA2985-21|BIOUG65686-D04|BOLD:AEJ2901
Mymaridae|GMPJA9429-21|BIOUG66652-G02|BOLD:AEJ2901
Mymaridae|GMPJA9619-21|BIOUG66654-G02|BOLD:AEJ2901
Mymaridae|GMPJA9865-21|BIOUG66657-C11|BOLD:AEJ2901
Mymaridae|GMPJA10067-21|BIOUG66659-D11|BOLD:AEJ2901
Mymaridae|GMPJA10018-21|BIOUG66658-H09|BOLD:AEJ2901
Mymaridae|GMPJA9406-21|BIOUG66652-E03|BOLD:AEJ2901
Mymaridae|GMPJA9390-21|BIOUG66652-C11|BOLD:AEJ2901
Mymaridae|GMPJA9360-21|BIOUG66652-A05|BOLD:AEJ2901
Mymaridae|GMPJA9343-21|BIOUG66651-G11|BOLD:AEJ2901
Hymenoptera|GMPJA9576-21|BIOUG66654-C07

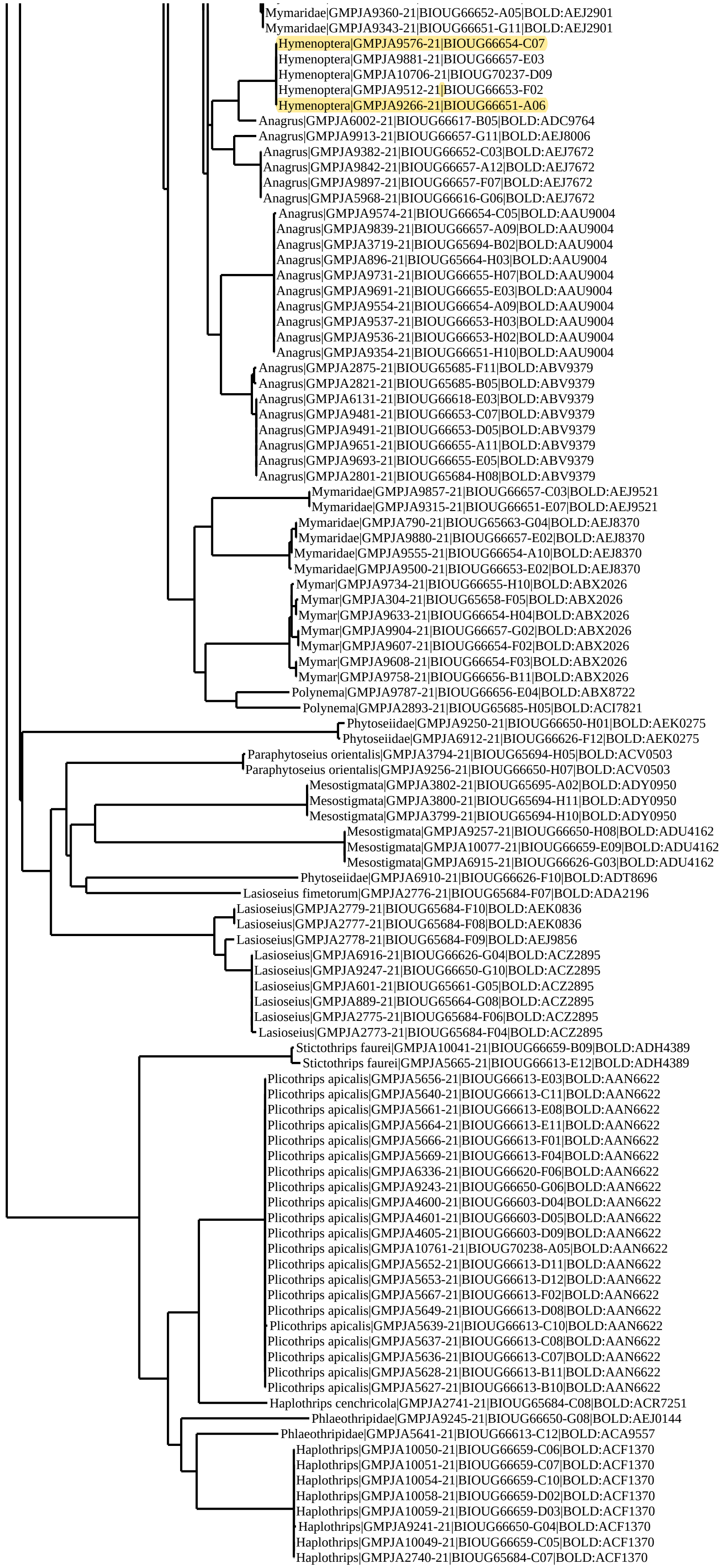
Mymaridae|GMPJA9360-21|BIOUG66652-A05|BOLD:AEJ2901
Mymaridae|GMPJA9343-21|BIOUG66651-G11|BOLD:AEJ2901
Hymenoptera|GMPJA9576-21|BIOUG66654-C07
Hymenoptera|GMPJA9881-21|BIOUG66657-E03
Hymenoptera|GMPJA10706-21|BIOUG70237-D09
Hymenoptera|GMPJA9512-21|BIOUG66653-F02
Hymenoptera|GMPJA9266-21|BIOUG66651-A06
Anagrus|GMPJA6002-21|BIOUG66617-B05|BOLD:ADC9764
Anagrus|GMPJA9913-21|BIOUG66657-G11|BOLD:AEJ8006
Anagrus|GMPJA9382-21|BIOUG66652-C03|BOLD:AEJ7672
Anagrus|GMPJA9842-21|BIOUG66657-A12|BOLD:AEJ7672
Anagrus|GMPJA9897-21|BIOUG66657-F07|BOLD:AEJ7672
Anagrus|GMPJA5968-21|BIOUG66616-G06|BOLD:AEJ7672
Anagrus|GMPJA9574-21|BIOUG66654-C05|BOLD:AAU9004
Anagrus|GMPJA9839-21|BIOUG66657-A09|BOLD:AAU9004
Anagrus|GMPJA3719-21|BIOUG65694-B02|BOLD:AAU9004
Anagrus|GMPJA896-21|BIOUG65664-H03|BOLD:AAU9004
Anagrus|GMPJA9731-21|BIOUG66655-H07|BOLD:AAU9004
Anagrus|GMPJA9691-21|BIOUG66655-E03|BOLD:AAU9004
Anagrus|GMPJA9554-21|BIOUG66654-A09|BOLD:AAU9004
Anagrus|GMPJA9537-21|BIOUG66653-H03|BOLD:AAU9004
Anagrus|GMPJA9536-21|BIOUG66653-H02|BOLD:AAU9004
Anagrus|GMPJA9354-21|BIOUG66651-H10|BOLD:AAU9004
Anagrus|GMPJA2875-21|BIOUG65685-F11|BOLD:ABV9379
Anagrus|GMPJA2821-21|BIOUG65685-B05|BOLD:ABV9379
Anagrus|GMPJA6131-21|BIOUG66618-E03|BOLD:ABV9379
Anagrus|GMPJA9481-21|BIOUG66653-C07|BOLD:ABV9379
Anagrus|GMPJA9491-21|BIOUG66653-D05|BOLD:ABV9379
Anagrus|GMPJA9651-21|BIOUG66655-A11|BOLD:ABV9379
Anagrus|GMPJA9693-21|BIOUG66655-E05|BOLD:ABV9379
Anagrus|GMPJA2801-21|BIOUG65684-H08|BOLD:ABV9379
Mymaridae|GMPJA9857-21|BIOUG66657-C03|BOLD:AEJ9521
Mymaridae|GMPJA9315-21|BIOUG66651-E07|BOLD:AEJ9521
Mymaridae|GMPJA790-21|BIOUG65663-G04|BOLD:AEJ8370
Mymaridae|GMPJA9880-21|BIOUG66657-E02|BOLD:AEJ8370
Mymaridae|GMPJA9555-21|BIOUG66654-A10|BOLD:AEJ8370
Mymaridae|GMPJA9500-21|BIOUG66653-E02|BOLD:AEJ8370
Mymar|GMPJA9734-21|BIOUG66655-H10|BOLD:ABX2026
Mymar|GMPJA304-21|BIOUG65658-F05|BOLD:ABX2026
Mymar|GMPJA9633-21|BIOUG66654-H04|BOLD:ABX2026
Mymar|GMPJA9904-21|BIOUG66657-G02|BOLD:ABX2026
Mymar|GMPJA9607-21|BIOUG66654-F02|BOLD:ABX2026
Mymar|GMPJA9608-21|BIOUG66654-F03|BOLD:ABX2026
Mymar|GMPJA9758-21|BIOUG66656-B11|BOLD:ABX2026
Polynema|GMPJA9787-21|BIOUG66656-E04|BOLD:ABX8722
Polynema|GMPJA2893-21|BIOUG65685-H05|BOLD:ACI7821
Phytoseiidae|GMPJA9250-21|BIOUG66650-H01|BOLD:AEK0275
Phytoseiidae|GMPJA6912-21|BIOUG66626-F12|BOLD:AEK0275
Paraphytoseius orientalis|GMPJA3794-21|BIOUG65694-H05|BOLD:ACV0503
Paraphytoseius orientalis|GMPJA9256-21|BIOUG66650-H07|BOLD:ACV0503
Mesostigmata|GMPJA3802-21|BIOUG65695-A02|BOLD:ADY0950
Mesostigmata|GMPJA3800-21|BIOUG65694-H11|BOLD:ADY0950
Mesostigmata|GMPJA3799-21|BIOUG65694-H10|BOLD:ADY0950
Mesostigmata|GMPJA9257-21|BIOUG66650-H08|BOLD:ADU4162
Mesostigmata|GMPJA10077-21|BIOUG66659-E09|BOLD:ADU4162
Mesostigmata|GMPJA6915-21|BIOUG66626-G03|BOLD:ADU4162
Phytoseiidae|GMPJA6910-21|BIOUG66626-F10|BOLD:ADT8696
Lasioseius fimetorum|GMPJA2776-21|BIOUG65684-F07|BOLD:ADA2196
Lasioseius|GMPJA2779-21|BIOUG65684-F10|BOLD:AEK0836
Lasioseius|GMPJA2777-21|BIOUG65684-F08|BOLD:AEK0836
Lasioseius|GMPJA2778-21|BIOUG65684-F09|BOLD:AEJ9856
Lasioseius|GMPJA6916-21|BIOUG66626-G04|BOLD:ACZ2895
Lasioseius|GMPJA9247-21|BIOUG66650-G10|BOLD:ACZ2895
Lasioseius|GMPJA601-21|BIOUG65661-G05|BOLD:ACZ2895
Lasioseius|GMPJA889-21|BIOUG65664-G08|BOLD:ACZ2895
Lasioseius|GMPJA2775-21|BIOUG65684-F06|BOLD:ACZ2895
Lasioseius|GMPJA2773-21|BIOUG65684-F04|BOLD:ACZ2895
Stictothrips faurei|GMPJA10041-21|BIOUG66659-B09|BOLD:ADH4389
Stictothrips faurei|GMPJA5665-21|BIOUG66613-E12|BOLD:ADH4389
Plicothrips apicalis|GMPJA5656-21|BIOUG66613-E03|BOLD:AAN6622
Plicothrips apicalis|GMPJA5640-21|BIOUG66613-C11|BOLD:AAN6622
Plicothrips apicalis|GMPJA5661-21|BIOUG66613-E08|BOLD:AAN6622
Plicothrips apicalis|GMPJA5664-21|BIOUG66613-E11|BOLD:AAN6622
Plicothrips apicalis|GMPJA5666-21|BIOUG66613-F01|BOLD:AAN6622
Plicothrips apicalis|GMPJA5669-21|BIOUG66613-F04|BOLD:AAN6622
Plicothrips apicalis|GMPJA6336-21|BIOUG66620-F06|BOLD:AAN6622
Plicothrips apicalis|GMPJA9243-21|BIOUG66650-G06|BOLD:AAN6622
Plicothrips apicalis|GMPJA4600-21|BIOUG66603-D04|BOLD:AAN6622
Plicothrips apicalis|GMPJA4601-21|BIOUG66603-D05|BOLD:AAN6622
Plicothrips apicalis|GMPJA4605-21|BIOUG66603-D09|BOLD:AAN6622
Plicothrips apicalis|GMPJA10761-21|BIOUG70238-A05|BOLD:AAN6622
Plicothrips apicalis|GMPJA5652-21|BIOUG66613-D11|BOLD:AAN6622
Plicothrips apicalis|GMPJA5653-21|BIOUG66613-D12|BOLD:AAN6622
Plicothrips apicalis|GMPJA5667-21|BIOUG66613-F02|BOLD:AAN6622
Plicothrips apicalis|GMPJA5649-21|BIOUG66613-D08|BOLD:AAN6622
Plicothrips apicalis|GMPJA5639-21|BIOUG66613-C10|BOLD:AAN6622
Plicothrips apicalis|GMPJA5637-21|BIOUG66613-C08|BOLD:AAN6622
Plicothrips apicalis|GMPJA5636-21|BIOUG66613-C07|BOLD:AAN6622
Plicothrips apicalis|GMPJA5628-21|BIOUG66613-B11|BOLD:AAN6622
Plicothrips apicalis|GMPJA5627-21|BIOUG66613-B10|BOLD:AAN6622
Haplothrips cenchricola|GMPJA2741-21|BIOUG65684-C08|BOLD:ACR7251
Phlaeothripidae|GMPJA9245-21|BIOUG66650-G08|BOLD:AEJ0144
Phlaeothripidae|GMPJA5641-21|BIOUG66613-C12|BOLD:ACA9557
Haplothrips|GMPJA10050-21|BIOUG66659-C06|BOLD:ACF1370
Haplothrips|GMPJA10051-21|BIOUG66659-C07|BOLD:ACF1370
Haplothrips|GMPJA10054-21|BIOUG66659-C10|BOLD:ACF1370
Haplothrips|GMPJA10058-21|BIOUG66659-D02|BOLD:ACF1370
Haplothrips|GMPJA10059-21|BIOUG66659-D03|BOLD:ACF1370
Haplothrips|GMPJA9241-21|BIOUG66650-G04|BOLD:ACF1370
Haplothrips|GMPJA10049-21|BIOUG66659-C05|BOLD:ACF1370
Haplothrips|GMPJA2740-21|BIOUG65684-C07|BOLD:ACF1370
