# Supplementary material for: Assessing arthropod biodiversity with DNA barcoding in Jinnah Garden, Lahore, Pakistan
Source: PeerJ. 2024 May 31;12:e17420. doi: 10.7717/peerj.17420 (PMC11146329; doi:10.7717/peerj.17420)
Supplement: Supplemental Information 6 [file peerj-12-17420-s006.pdf]

Diversity Measures Result - DS-GMPJA

Alpha Diversity for All Data (1361 Observed Units - Barcode Cluster (BIN))

Preston

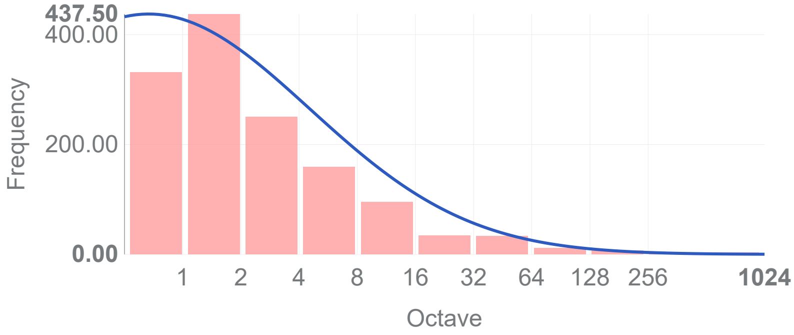

Est. Species: 2785 (Log Normal - **Mode:** -0.58, **Stdev:** 2.79, **Veiled:** 1424).

Alpha Indices

●	Fisher's Alpha Index	446.28
●	Chao-1 Index	2389.74
●	Simpson Index of Diversity (1-D)	0.989
●	Shannon Index	5.77

Alpha Diversity per Site **Site Summary** **Data**

Map of Sites or Clusters

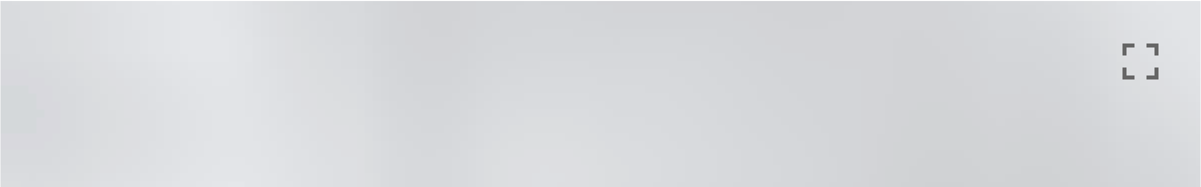

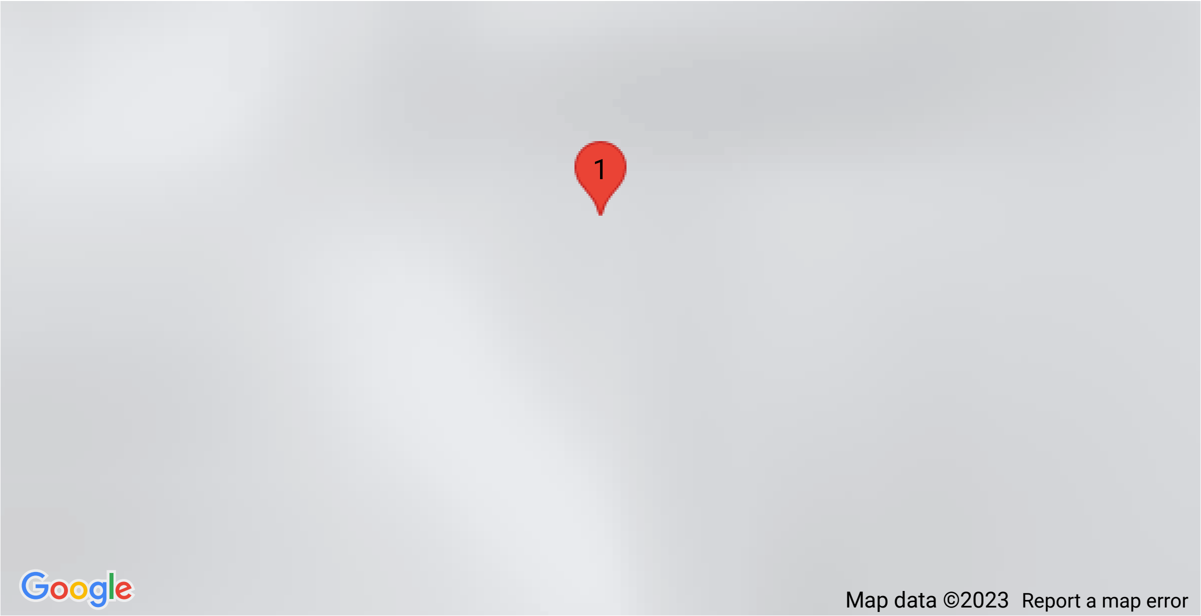

1: null

Analysis description

Alpha Diversity and Beta Diversity are used to (1) characterize the numbers of species based on their observed counts, and (2) estimate the total number of species as a function of unique observations and sample size. Alpha Diversity describes the *intrasite* diversity, and Beta Diversity describes the *intersite* diversity.

Diversity Quantities Computed: Alpha diversity
Number of Sites: 1
Features: Barcode Cluster (BIN), Site Code (2)
Group By: Geography: GPS
Minimum Group Size: 100
Filters Applied: Records with BINs

Filter Summary

Data Summary

Copyright BOLD © 2014-2023
